# CRISPR elements provide a new framework for the genealogy of the citrus canker pathogen *Xanthomonas citri* pv. *citri*

**DOI:** 10.1186/s12864-019-6267-z

**Published:** 2019-12-02

**Authors:** Kwanho Jeong, Alejandra Muñoz-Bodnar, Nathalia Arias Rojas, Lucie Poulin, Luis Miguel Rodriguez-R, Lionel Gagnevin, Christian Vernière, Olivier Pruvost, Ralf Koebnik

**Affiliations:** 1 IRD, Cirad, Université de Montpellier, IPME, Montpellier, France; 2 Present address: Current address: Department of Plant Pathology, University of Florida, Gainesville, FL 32611 USA; 3 Present address: Laboratoire de Biologie et de Pathologie Végétales, Université de Nantes, Nantes, France; 4 Present address: Department of Civil and Environmental Engineering, Georgia Institute of Technology, Atlanta, GA 30332 USA; 5 CIRAD, UMR PVBMT, 97410 Saint Pierre, La Réunion France; 6 CIRAD, UMR BGPI, 34398 Montpellier, France

**Keywords:** Molecular typing, Genetic diversity, Clustered regularly interspaced short palindromic repeats, Variable numbers of tandem repeats, Spoligotyping, Epidemiology, Phylogeny, Evolution, *Xanthomonas citri* pv. *citri*

## Abstract

**Background:**

Xanthomonads are an important clade of Gram-negative bacteria infecting a plethora of economically important host plants, including citrus. Knowledge about the pathogen’s diversity and population structure are prerequisite for epidemiological surveillance and efficient disease management. Rapidly evolving genetic loci, such as Clustered Regularly Interspaced Short Palindromic Repeats (CRISPR), are of special interest to develop new molecular typing tools.

**Results:**

We analyzed CRISPR loci of 56 *Xanthomonas citri* pv. *citri* strains of world-wide origin, a regulated pathogen causing Asiatic citrus canker in several regions of the world. With one exception, 23 unique sequences built up the repertoire of spacers, suggesting that this set of strains originated from a common ancestor that already harbored these 23 spacers. One isolate originating from Pakistan contained a string of 14 additional, probably more recently acquired spacers indicating that this genetic lineage has or had until recently the capacity to acquire new spacers. Comparison of CRISPR arrays with previously obtained molecular typing data, such as amplified fragment length polymorphisms (AFLP), variable-number of tandem-repeats (VNTR) and genome-wide single-nucleotide polymorphisms (SNP), demonstrated that these methods reveal similar evolutionary trajectories. Notably, genome analyses allowed to generate a model for CRISPR array evolution in *X. citri* pv. *citri*, which provides a new framework for the genealogy of the citrus canker pathogen.

**Conclusions:**

CRISPR-based typing will further improve the accuracy of the genetic identification of *X. citri* pv. *citri* outbreak strains in molecular epidemiology analyses, especially when used concomitantly with another genotyping method.

## Background

Xanthomonads are a large genus of Gram-negative, plant-associated gamma-proteobacteria that shows a high degree of host plant specificity. Pathogenic members of the genus cause diseases on over 300 host plants [1]. Many of these bacteria cause significant yield losses of economically important crops, such as cereals, solanaceous and brassicaceous plants [2]. They cause a variety of symptoms, including necrosis, cankers, spots, and blight, and they affect different parts of the plant, including leaves, stems, and fruits [3]. One of the most important diseases caused by *Xanthomonas* is citrus canker, which results in significant yield losses on susceptible citrus species [4, 5]. Citrus canker does not only reduce fruit quality and yield but also triggers immediate quarantine restrictions, thus increasing its impact on economy by disrupting trade and implementation of costly eradication programs [5, 6].

Citrus canker is commonly used as a generic term that includes two diseases of citrus caused by strains of *Xanthomonas citri*. Asiatic citrus canker, which is caused by *X. citri* pv. *citri* (synonyms, *X. citri* subsp. *citri* and *X. axonopodis* pv. *citri*), is prevalent worldwide and causes major outbreaks. South American citrus canker, which is caused by *X. citri* pv. *aurantifolii* (synonym, *Xanthomonas fuscans* subsp. *aurantifolii*), is geographically restricted to a few South American countries with minor agricultural significance and is very uncommonly isolated from naturally infected citrus [5]. Two other xanthomonads, *X. citri* pv. *bilvae* and *Xanthomonas euvesicatoria* pv. *citrumelonis*, were reported as citrus pathogens but they produce necrotic spots rather than canker-like lesions and are considered minor pathogens [7–10]. Both canker-causing pathovars were further subdivided into pathotypes (i.e. groups of strains differing in host range within the *Citrus* genus). Three (A, A* and A^w^) and two (B and C) pathotypes are recognized within *X. citri* pv. *citri* and *X. citri* pv. *aurantifolii*, respectively [11–13].

Due to the enormous economic impact, molecular DNA-based methods were developed to rapidly identify and type strains of bacteria associated with citrus canker, including RFLP (restriction fragment length polymorphism), AFLP (amplified fragment length polymorphism), and rep-PCR (repetitive element-polymerase chain reaction) [14–17]. However, these approaches suffered from technical challenges, problematic reproducibility and/or limited comparability. An accurate understanding of the phylogeny and evolution and proper identification of *X. citri* pv. *citri* strains was achieved through a genome sequencing approach, referred to as next generation sequencing (NGS), which facilitated the genome-wide analysis of evolutionary events in a set of 43 *X. citri* pv. *citri* strains [18]. However, robust and high-resolution genotyping methods, which are less costly, easy to perform and which offer good reproducibility and portability are still required for routine outbreak investigations. Two robust genotyping methods targeting tandem repeats (MLVA; multilocus variable-number of tandem-repeats [VNTR] analysis) suitable for analyses at different evolutionary scales have been developed for *X. citri* pv. *citri* [19–21]. Minisatellite-based typing (MLVA-31) and microsatellite-based typing (MLVA-14) are suited for global and local epidemiological analyses, respectively.

**Fig. 1:**
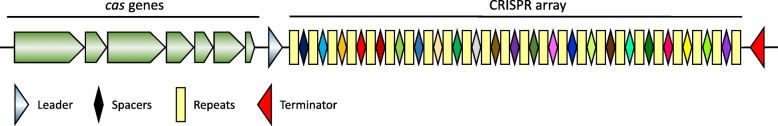
Schematic representation of the *X. citri* pv. *citri* CRISPR/Cas locus. Conserved repeats are shown as yellow rectangles, spacers are represented by diamonds in different colors and the leader with the presumed promoter and the terminator region are represented by a blue and a red triangle, respectively. Genes of the *cas* gene cluster are schematically represented by green arrows. Genetic elements are not drawn to scale

Clustered regularly interspaced short palindromic repeats (CRISPRs) constitute a family of DNA repeat sequences, which are widely distributed among *Archaea* and *Bacteria* [22–24]. This genetic locus consists of highly conserved DNA repeats that are interspersed by unique, similarly sized spacers, which are acquired from alien DNA elements such as bacteriophages or conjugative plasmids (Fig. 1). CRISPR repeats and spacers form rapidly evolving arrays that can contain up to 100 or even more spacer/repeat units [25, 26]. Typically, CRISPR loci are associated with a conserved *cas* (CRISPR-associated sequence) gene cluster [27], which functions in the acquisition of new spacers and in the protection against subsequent phage infection. Among the *cas* genes, *cas1* is the only gene which is present in almost all known CRISPR/Cas systems and can therefore be considered as the best marker for CRISPR/Cas systems [28, 29]. Once integrated into the CRISPR array, newly acquired spacers interfere with subsequent infection by DNA elements that carry a matching sequence in their genetic repertoire. Thus, CRISPR/Cas systems function as an adaptive microbial immune system. Notably, new spacers become almost always introduced at the same side of the locus close to the leader sequence; thus, the CRISPR array grows at the proximal end [30–32].

Making use of the polymorphisms in the CRISPR locus, a typing method has been developed for mycobacteria called “spoligotyping” (for spacer oligonucleotide typing) [33, 34]. Spoligotyping is a technique for the identification and analysis of polymorphisms in certain types of spacer/repeat units of CRISPR loci. A PCR-based reverse-line hybridization blotting technique is used to monitor the genetic diversity at CRISPR loci. This method turned out to be extremely useful for routine assays in clinical laboratories as well as for molecular epidemiology, evolutionary and population genetics since it is a fast, robust and cost-effective genotyping method complementary to more traditional fingerprinting techniques. More recently, a new spoligotyping method based on microbeads was proposed for *Mycobacterium tuberculosis* and *Salmonella enterica* [35, 36], thus further increasing the throughput and the amount of data that can be queried in internet-accessible databases [37, 38].

CRISPR-based molecular typing did not stay restricted to human pathogens such as *Corynebacterium diptheriae*, *Escherichia coli*, *Legionella pneumophila*, *M. tuberculosis*, *Porphyromonas gingivalis*, *S. enterica*, group A *Streptococcus* and *Yersinia pestis* [39]. Polymorphisms in CRISPR arrays were first reported for rice-pathogenic xanthomonads [40, 41]. It was noted that the CRISPR region of rice-pathogenic *Xanthomonas oryzae* evolves very rapidly and thus provides one of the most striking records of differentiation among bacterial isolates originating from different geographic areas. However, the first applications for plant-pathogenic bacteria were reported for *Erwinia amylovora*, the causal agent of fire blight, which can affect most members of the *Rosaceae* family [42, 43]. CRISPR array polymorphisms in this highly homogeneous species allowed clustering representative strains from a worldwide collection into well-defined, evolutionary related groups that reflected their geographic origins and the host plants from which they were isolated. Recently, CRISPR typing combined with VNTR analysis was applied for the first time to strains of *Xanthomonas* infecting strawberry [44]. Importantly, CRISPR spacer analysis and MLVA of strawberry-infecting *Xanthomonas fragariae* displayed a congruent population structure, in which two major groups and a total of four subgroups were revealed. Results from this work suggested that the two main groups are responsible for the worldwide expansion of the angular leaf spot disease on strawberry plants.

Here, we describe the CRISPR loci from a representative set of *X. citri* pv. *citri* strains in order to develop a robust and cost-effective molecular typing method that complements other typing tools, such as MLVA. Since CRISPR loci offer the advantage of building evolutionary scenarios based on time-resolved acquisition and loss of spacers, analysis of *X. citri* pv. *citri* CRISPR arrays give new insight into the phylogeny and worldwide epidemic of this important plant pathogen.

## Results

### PCR screening of *X. citri* strains for the presence of the *cas1* gene

In order to elucidate whether CRISPR/Cas loci are widespread among strains of *X. citri* pv. *citri*, we first screened our strain collection (*n* = 56) as well as a citrus-pathogenic *X. citri* pv. *bilvae* strain for the presence of *cas1*, the most conserved *cas* gene, by conventional PCR using *cas1*-specific primers. A DNA fragment of approximately 220 bp corresponding to the *cas1* gene was amplified from all 56 *X. citri* pv. *citri* strains (Additional file 1: Figure S1), indicating that these strains may possess a CRISPR/Cas locus of potential use for molecular typing. However, the *X. citri* pv. *bilvae* strain (NCPPB 3213) was negative in the PCR screen, suggesting that the *cas1* gene may not be conserved in the pathovar *bilvae* (Additional file 1: Figure S1).

### PCR screening of *X. citri* strains for the presence of a CRISPR locus

All 57 strains were then subjected to PCR amplification of the complete CRISPR locus, using leader- and terminator-specific primers. As expected, PCR products were obtained for all of the *X. citri* pv. *citri* strains, most of which varied in size between 500 bp and 1400 bp depending on the strain (Additional file 2: Figure S2). These different sizes, probably corresponding to different numbers of spacer/repeat units, indicated that differential deletion and/or acquisition events had occurred. However, for five *X. citri* pv. *citri* strains, a weak signal corresponding to a DNA fragment of approximately 3500 bp was detected, indicating the presence of an exceptionally large CRISPR locus (Additional file 2: Figure S2, lanes 19, 20, 33, 49 and 50).

On the other hand, no DNA amplification occurred when using DNA of the *X. citri* pv. *bilvae* strain NCPPB 3213, which was also negative for *cas1* (Additional file 1: Figure S1). This result suggested that either NCPPB 3213 does not have a CRISPR/Cas system or that the leader and/or terminator sequences are too distant and do not allow annealing of the used PCR primer(s). We therefore scrutinized the draft genome sequence of strain NCPPB 3213 (NCBI BioProject PRJEB7165) for the presence of *cas* genes or the CRISPR array, using the CRISPRCasFinder website. This search did not provide evidence that this strain of *X. citri* pv. *bilvae* would possess this type of CRISPR/Cas immunity system. For these reasons, strain NCPPB 3213 was excluded from further analyses.

In summary, these results suggest that most, if not all, *X. citri* pv. *citri* strains possess a CRISPR/Cas system, which evolved sufficient diversity due to the acquisition and/or loss of spacer/repeat units, thus allowing the development of a spacer-based typing scheme.

### PCR screening of *X. citri* strains for the presence of an IS element in CRISPR loci

For five strains of *X. citri* pv. *citri* (LB302, LB305, LG097, LG115, and NCPPB 3608), a DNA fragment of large molecular mass was weakly amplified using primers flanking the CRISPR array. Because we had access to draft genome sequences of most of these strains, we checked for the presence of CRISPRs loci using CRISPRCasFinder. For each strain, two contigs were predicted to contain an array of spacers and repeats, with one contig harboring four to five repeats of the leader-proximal end (spacers Xcc_23 to Xcc_20) and another contig harboring 16 to 20 repeats of the terminator-proximal end (spacers Xcc_20 to Xcc_01) (Additional file 3: Figure S3, Additional file 4: Figure S4 and Additional file 5: Figure S5). Notably, all spacer/repeat arrays were found at the ends of the contigs, suggesting that genome assembly was not complete due to the repetitive character of the sequence or due to other factors. Indeed, scrutiny of the contig ends allowed to identify a short inverted repeat, as typically found at the extremities of an IS element. When analyzing the draft genome sequence of NCPPB 3608, we found these inverted repeats 42 times, always located at the end of contigs, further supporting the hypothesis of an IS element insertion in the CRISPR locus (Additional file 3: Figure S3, Additional file 4: Figure S4 and Additional file 5: Figure S5). BLASTN searches identified similar inverted repeats at the extremities of annotated IS elements in the genome of *Ralstonia solanacearum* strain Po82 (GenBank accession number CP002820). The *IS Finder* database identified this IS element as IS*Rso19*, which belongs to the IS family IS*21*.

Using the full-length IS*Rso19* element as a query, we found a single contig in the draft genome of NCPPB 3608 with 72% sequence identity, CCWG01000056.1, encompasing most of the IS element. Based on sequence information from the *X. citri* pv. *citri* and *R. solanacearum* IS elements, we designed PCR primers to amplify the flanking spacer/repeat units. All five strains that resulted in PCR amplification of a large band of weak intensity (LB302, LB305, LG097, LG115 and NCPPB 3608) were evaluated for the presence of the IS element in the CRISPR locus (Additional file 6: Figure S6). PCR with primer combinations Leader_fw and IS-1_rev and IS-2_fw and Spacer#18_rev resulted in the amplification of a DNA fragment of approximately 800 bp and 750 bp, respectively, for strains LB302, LB305, LG115 and NCPPB 3608. In contrast, the amplicon of strain LG097 was slightly larger with primer combination Leader_fw and IS-1_rev and no specific amplification occured with primer combination IS-2_fw and Spacer#18_rev (Additional file 6: Figure S6). These results suggested that strains LB302, LB305, LG115 and NCPPB 3608 contain an IS element between spacers Xcc_23 and Xcc_18 while strain LG097 might not possess spacer Xcc_18.

Sequencing of these DNA fragments confirmed that strains LB302, LB305, LG115 and NCPPB 3608 contain an IS element at exactly the same position between spacers Xcc_21 and Xcc_20 (Additional file 3: Figure S3 and Additional file 4: Figure S4). Sequencing of the amplicon from strain LG097 revealed the presence of spacers Xcc_23, Xcc_22, Xcc_20, Xcc_19 and Xcc_18 (except for 4 bp at the site of the IS element insertion) between the leader region and the IS element (Additional file 5: Figure S5). To amplify the opposite site of the IS element insertion in LG097, we performed a PCR with primers IS-2_fw and Terminator_rev. DNA sequencing confirmed that an IS element had inserted in spacer Xcc_18 in strain LG097 (Additional file 5: Figure S5).

### Analysis of CRISPR spacers and spoligotypes

**Fig. 2:**
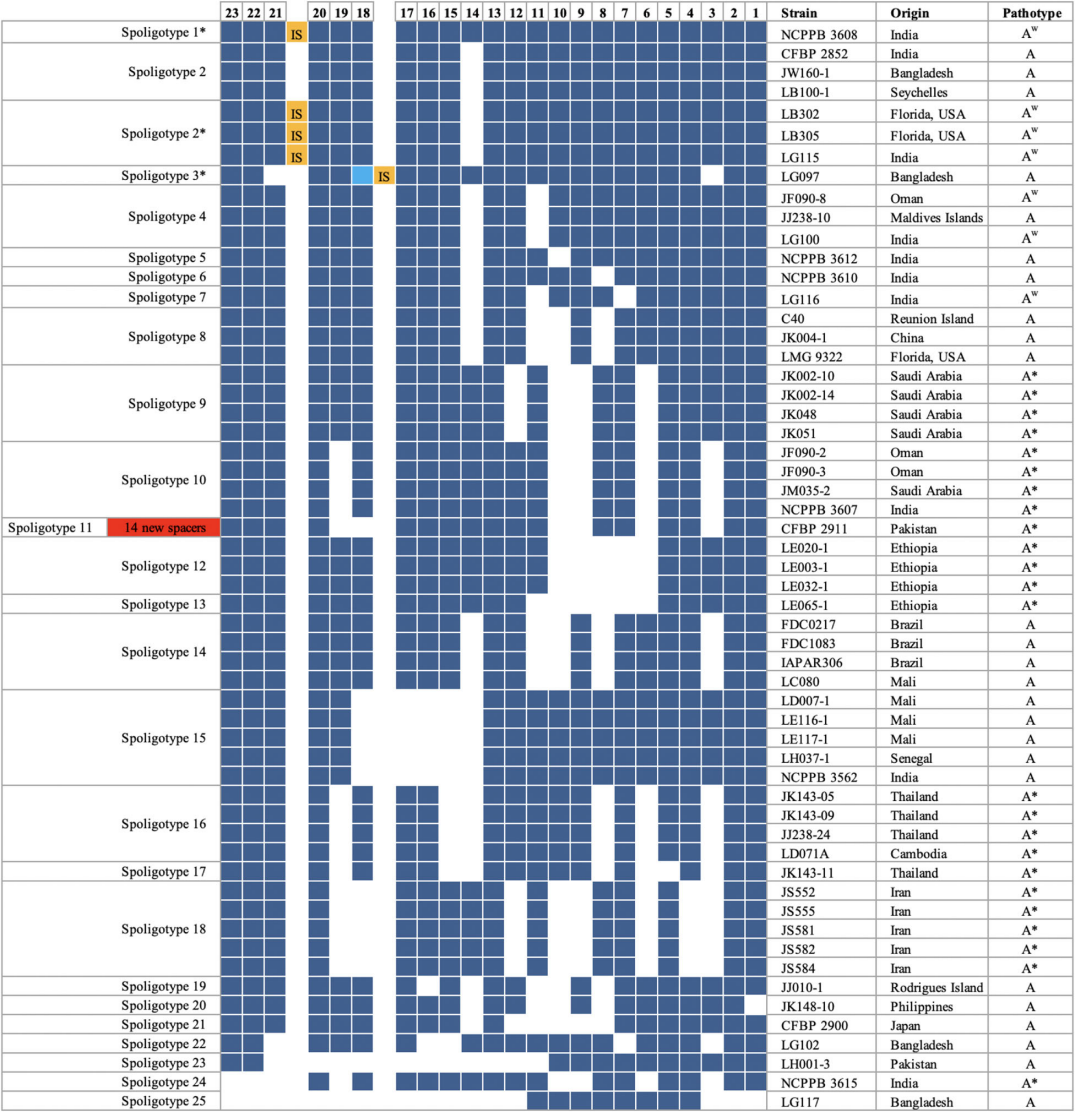
Spoligotypes of 56 *X. citri* pv. *citri* strains. CRISPR arrays are oriented with the leader-proximal spacers on the left side. Identical spacers within the same block are vertically aligned. Detected CRISPR spacers are represented by deep blue boxes, with the identifier of spacers indicated by numbers in the first row. White boxes indicate the absence of the corresponding spacer. Orange boxes indicate the presence of IS elements and the light blue box indicates a variant of spacer Xcc_18 with a deletion of 4 bp due to the IS element insertion. 14 unique spacers are shown as red box for strain CFBP 2911. Spoligotype 2* is identical to spoligogtype 2, but contains an IS element between spacers Xcc_20 and Xcc_21

CRISPR loci from all 56 *X. citri* pv. *citri* strains were completely sequenced and patterns of presence and absence of spacers were analyzed. Altogether, 25 different patterns (spoligotypes) were found (Fig. 2). A total of 37 distinct spacers were identified among the 56 *X. citri* pv. *citri* strains. Most strains contain between 8 and 23 spacer/repeat units, corresponding to spacers Xcc_01 to Xcc_23. Strain CFBP 2911 was exceptional in that it contains 14 unique spacers (Xcc_24 to Xcc_37), bringing the total number of spacer/repeat units of this strain to 31 (Fig. 2). This strain was the only one that contains spacers Xcc_24 to Xcc_37. The size of spacers varies between 34 bp and 37 bp (Table 1). Except for strain CFBP 2911, spacer Xcc_23 was likely the most recently acquired spacer, which is conserved in most of the 56 strains (except for LG117 and NCPPB 3615). Most of the 25 spoligotype patterns likely evolved by the deletion of a single spacer/repeat unit although simultaneous deletion of adjacent spacer/repeat units probably occurred as well, as suggested by the absence of intermediate CRISPR structures (Fig. 2). Deletion of spacer/repeat units appeared to be random.

**Table 1:** List of spacer sequences of *Xanthomonas citri* pv. *citri* identified in the present study and homologous sequences in other organisms

Name	Sequence (5′ → 3′)	Bacteriophage-related homologs
Xcc_37 *	aggtatggattgcccgccatagggcggatgttgtcg	(Phage from *Xanthomonas*)
Xcc_36 *	tcgctaatcgccaaattgctggagattggccgcgg	Phage from *Xanthomonas*
Xcc_35 *	accatcgaagccgagtacaatggcatgtacgtggag	Phages from *Xanthomonas* and *Xylella*
Xcc_34 *	ctcatgtactcaaccgtaaactcacgcacgacacg	[Phage from *Xanthomonas*]
Xcc_33 *	accaacgcactggcccgccgagctgacatccacag	Phage from *Xanthomonas*
Xcc_32 *	atctgcttgtctagttccaaaatcgccttaaccgg	[Phage from *Xanthomonas*]
Xcc_31 *	atcgacggcggcggcatggtgtgggactgccagctg	Phages from *Xanthomonas* and *Xylella*, prophage in *Ralstonia*; (Phages from *Burkholderia*, *Ralstonia* and *Xylella*)
Xcc_30 *	atcgccagcaagcccatgagcaagggcggctgcgg	Phages from *Xanthomonas*
Xcc_29 *	ctcatcaccaccctggagaacgcagcggaaagatgg	No
Xcc_28 *	gagttcgagggcaagaagaagacgcaggatgaaggg	Phages from *Xanthomonas*; (Phages from *Caulobacter* and *Xylella*)
Xcc_27 *	ttgcgtataccatccggcccgaacttctccgagg	Phages from *Xanthomonas*; (Phage from *Xanthomonas*)
Xcc_26 *	tattaggagacaatatgaatactgcacctaacatg	No
Xcc_25 *	tgtagattcggcgaattggatgacaggcgaccgg	Phage from *Xanthomonas*
Xcc_24 *	tcttaagagaagctcggatcgtggtttcaaggtcg	No
Xcc_23	aaatgctttcgacgcgcataaagcgctggcgcaggag	No
Xcc_22	ctgttcaagctccgccgcctgatccgcttgccgag	Filamentous phage in *X. citri* pv. *vignicola*
Xcc_21	ctcgggtttcgggatgtgcttcagatctgcgtcg	No
Xcc_20	cgctgcacggatgcgccaggcggcgaggcgatcat	Prophage in *X. citri* pv. *vignicola*
Xcc_19	tcgagcgcatcgatgacggtcacccatcccccaatg	No
Xcc_18	gtgccaccgacagcgacgcacgtggacctgcagatc	No
Xcc_17	ctctctcacgccgcgcgtgcgagatcctgcgtgc	No
Xcc_16	gcagactgccgaggccggcatgctggaggggcgcct	Prophage in *X. citri* pv. *phaseoli*
Xcc_15	gggttaacaacgccttgaaacggctttgccgcgacgc	No
Xcc_14	acgtcttggacctgggtgtggttgctgagatagtca	No
Xcc_13	gccatcatgctttgaatgcgcttacccacggcgaa	No
Xcc_12	gcggatatgtgattagacccttttacgactttcag	No
Xcc_11	atgtcgaaaacgatggccttgacgtcatcgtctgc	(Phage from *Achromobacter*); [Phage from *Streptomyces*]
Xcc_10	ttcgctggcatcggtggatggagccttgcgcttc	(Uncultured Mediterranean bacteriophage)
Xcc_9	tcattgaacccaaggaccacttcgcagggcgact	No
Xcc_8	ttgaccacatgttctctctgtgggaggaaggcac	No
Xcc_7	tgtcgagcgcgcactgctgccgcgatggccggaa	No
Xcc_6	ggctgggagcgttacaagtttgagcagcccgtag	No
Xcc_5	tggttcagggctggaaagacttggatgcccgcatc	No
Xcc_4	ctgactatccctgcataggccacgacctgcgagg	No
Xcc_3	aagaagaccagtctgcggcgtcgcggcatcctgggg	No
Xcc_2	ctgagttcgtcgccgtcccggtcgtctgacgcgt	[Phage from *Microbacterium*]
Xcc_1	catgccatatgcggcgagatcgcacagcagaaggaa	Prophage in *X. citri* pv. *vignicola*

*, these spacers were only detected in strain CFBP 2911

Homologs are indicated in round brackets when they match with less stringent search criteria (E-value between 0.1 and 1) (Additional file 7: Table S1). Homologs in square brackets indicate that these are matches with E-values > 1 (see Discussion)

In order to decipher the origin of the 37 spacers, the NCBI GenBank was queried for similar sequences using the BLASTN algorithm. As expected, spacers Xcc_23 to Xcc_01 had hits in several genome sequences of *X. citri* pv. *citri*, reflecting their high conservation in this pathovar of the species *X. citri*.

Using stringent thresholds (E-value smaller than 0.1 and at least 90% coverage of the query sequence), we found significant matches between eight spacers and sequences from *Xanthomonas*-specific bacteriophages, which were however restricted to the 14 unique spacers of strain CFBP 2911 (Table 1; Additional file 7: Table S1). The other six spacers among the 14 unique CFBP 2911 spacers did not have any significant hit. Among the *Xanthomonas* bacteriophages, we found one that had been shown to cause lytic infections of some strains of *X. citri* pv. *citri* (bacteriophage CP1, GenBank accession number AB720063) [45]. Bacteriophage phi Xc10 (GenBank accession number MF375456) can infect *X. citri* pv. *citri*, but also *Xanthomonas citri* pv. *glycines* and *Xanthomonas campestris* pv. *campestris*. Three bacteriophages, f30-Xaj (GenBank accession number KU595433), f20-Xaj (GenBank accession number KU595432) and XAJ24 (GenBank accession number KU197013), were isolated from walnut trees and have lytic activity against *Xanthomonas arboricola* pv. *juglandis* [46, 47]. All five bacteriophages belong to the order of *Causovirales*, with CP1 being a member of the *Siphoviridae* and the others being members of the *Podoviridae*. Spacer Xcc_35 was also similar to a virulent bacteriophage for *Xylella fastidiosa* (bacteriophage Prado; *Caudovirales*; *Podoviridae*; GenBank accession number KF626667) with a host range that includes *Xanthomonas* spp. [48]. Spacer Xcc_31 was also similar to a sequence in the genome of the *Ralstonia*-related blood disease bacterium R229 (GenBank accession number FR854082), which likely belongs to an integrated prophage and encodes a DNA polymerase A (GenBank accession number CCA83269.1) (Additional file 7: Table S1).

Among the conserved 23 spacers, only four had significant matches in the non-redundant GenBank database, all of which corresponded to sequences from other *Xanthomonas* species or pathovars (Additional file 7: Table S1). Spacers Xcc_22, Xcc_20 and Xcc_01 were similar to sequences in the *X. citri* pv. *vignicola* strain CFBP 7113. Notably, spacer Xcc_22 matched to locus XcvCFBP7113P_11110, which has been annotated to encode a hypothetical protein. However, BLASTP search of the coding sequence revealed 80% sequence identity with protein I of the *Xanthomonas campestris* filamentous bacteriophage ΦLf (GenBank accession number AAB88261) [49]. Spacer Xcc_01 matched to locus XcvCFBP7113P_16810 (annotated as hypothetical protein with similarity to the Pfam domain NinB [PF05772; E-value 8.2e-30], which corresponds to the DNA recombination protein NinB of bacteriophage lambda) and spacer Xcc_20 matched to the intergenic region between loci XcvCFBP7113P_16630 and XcvCFBP7113P_16635. All these loci belong to a 29-kb region (GenBank accession number CP022270; 3,740,909 to 3,769,866) that likely corresponds to (remnants of) a prophage. A similar region with 74% sequence identity over the whole length is present in the genomes of the *X. citri* pv. *phaseoli* var. *fuscans* strains (e.g. strain CFBP 6988R, GenBank accession number CP020979, 3,315,711–3,346,400). Interestingly, spacer Xcc_16 matches to a sequence motif in this region (e.g. locus XcfCFBP6988P_14885 in strain CFBP 6988R, annotated as hypothetical protein). Thus, all spacers that had a hit in the GenBank database derived from bacteriophage or prophage sequences.

### Comparison of evolutionary distance trees derived from AFLP and CRISPR genotyping

**Fig. 3:**
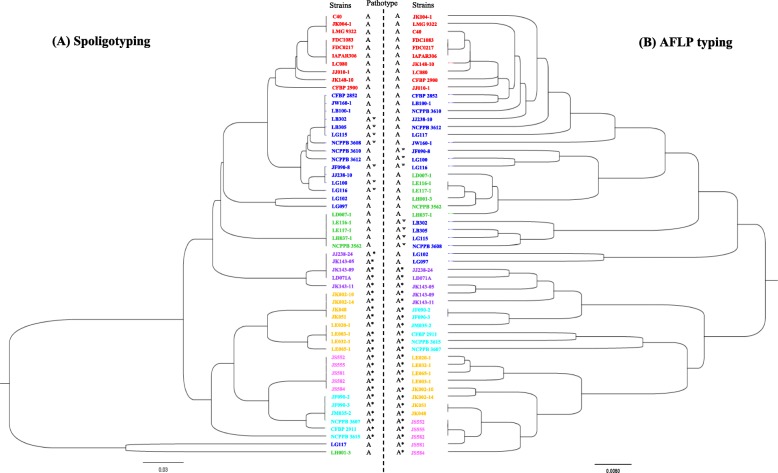
Comparison of phylogenetic analyses based on CRISPR data (**a**) and AFLP data (**b**) for 56 strains of *X. citri* pv. *citri.* AFLP data were taken from previous work [17]. AFLP and CRISPR data were converted into a binary array according to the presence or absence of each marker (except for the 14 unique spacers of strain CFBP 2911) and clustering was inferred using the UPGMA method. Different colors of characters indicate different clusters and the same strains are represented by the same color in both panels

We analyzed the distances of 56 *X. citri* pv. *citri* strains based on information about the CRISPR locus, which was obtained by conventional PCR and DNA sequencing, and compared them with those from AFLP analyses (Fig. 3). In general, there was a fairly good congruence between the two methods, except for strains LG117 and LH001–3. The 25 spoligotypes of the 56 *X. citri* pv. *citri* strains were classified in 7 groups and 2 singletons. In contrast, AFLP generated 49 haplotypes for the same set of strains (Fig. 3). Both genotyping methods accurately classified strains with respect to the two major pathotypes, A and A*, with the few A^w^ strains strongly linked to the A strains (Fig. 3). However, spoligotypes were found to lack resolution for accurate identification of A^w^ strains, as in several cases, A and A^w^ strains were found to share identical patterns. Distinction between these strains was only possible by additional evidence. For instance, the presence of an IS element could distinguish some A^w^ strains (LB302, LB305, LG115) from A strains (CFBP 2852, JW160–1, LB100–1).

### Comparison of the discriminatory power of CRISPR typing with other genotyping methods

To define the advantage of CRISPR typing tool, we compared the discriminatory power of CRISPR typing with other genotyping methods that have been applied to *X. citri* pv. *citri* previously [17, 19, 21]. The Mantel pairwise correlation results revealed the highest value between MLVA-31 data and AFLP data (*r* = 0.590; *P* <  0.001) (Table 2). When comparing the CRISPR typing method with the other methods, the best correlation was found with AFLP typing, showing a relatively high and significant value (*r* = 0.467; P <  0.001) (Table 2). Globally, the genetic distances derived from the four typing methods were highly significantly congruent in almost all cases (P <  0.001), while the distances between microsatellite (MLVA-14) and CRISPR data were less significantly congruent (*P* = 0.021) (Table 2).

**Table 2:** Mantel test results for the pairwise correlations of genetic distances among 56 strains of *Xanthomonas citri* pv. *citri* obtained for four different genotyping methods. Mantel coefficients above the diagonal, *P* values of Mantel correlation coefficients below the diagonal

Genotyping methods	AFLP^a^	CRISPR	MLVA-14^a^	MLVA-31^a^
AFLP	–	0.467	0.397	0.590
CRISPR	< 0.001	–	0.152	0.333
MLVA-14	< 0.001	0.021	–	0.509
MLVA-31	< 0.001	< 0.001	< 0.001	–

^a^ Data for MLVA-14, MLVA-31 and AFLP analyses were taken from previously published datasets [17, 19, 21]

## Discussion

The CRISPR locus is an important genetic locus that can be used for bacterial typing in molecular epidemiology analyses [39]. Whereas CRISPR-based typing and comparison of strains has become an established technique for human pathogens, it has remained largely unexplored for plant pathogens [50]. To the best of our knowledge, only a few studies have been published, mostly on a single plant pathogen, *E. amylovora* [42, 43, 51]. Very recently, two CRISPR loci, one of which displayed sufficient complexity for being used as a strain subtyping technique, were reported from *X. fragariae* [44]. CRISPR data, analyzed from a collection of 55 *X. fragariae* strains, yielded a genetic structure in agreement with that derived from MLVA data targeting 27 microsatellites and 9 minisatellites.

### Presence of CRISPR loci in citrus-infecting xanthomonads

In the present study, we analyzed 57 strains of *X. citri* for the presence of CRISPR loci. Our results demonstrated that both the *cas1* gene and the CRISPR array are conserved in all 56 strains of *X. citri* pv. *citri*. However, our PCR screen failed to amplify a *cas1* gene or a CRISPR array in the *X. citri* pv. *bilvae* strain NCPPB 3213. We conclude that at least this *X. citri* pv. *bilvae* strain does not have a *X. citri* pv. *citri*-type CRISPR/Cas system, which is supported by the absence of CRISPR-related sequences in its draft genome sequence. Notably, other xanthomonads infecting citrus, such as *Xanthomonas citri* pv. *aurantifolii* (strains 1566, FDC1559, FDC1561, FDC1609, ICPB 10535, ICPB 11122) and *Xanthomonas euvesicatoria* pv. *citrumelo* (strain F1, synonymous to FL1195) do also not have CRISPR loci, as indicated by the absence of *cas* genes and CRISPR arrays in the genome sequences. Hence, CRISPR loci appear to be restricted to *X. citri* pv. *citri* among citrus-infecting xanthomonads and our results demonstrate that the *cas1* gene is a useful diagnostic marker for the presence or absence of the CRISPR/Cas system and could be used to differentiate citrus pathogens of the genus *Xanthomonas*.

### CRISPRs in *X. citri* pv. *citri* are adapted for a simple genotyping based-PCR tool

Compared to other strains of *Xanthomonas*, such as *X. oryzae* pv. *oryzae* [40, 41], the CRISPR locus of *X. citri* pv. *citri* is rather small. Most strains of *X. citri* pv. *citri* have only 23 or fewer spacers while strains of *X. oryzae* pv. *oryzae* have been found to possess between 37 (Xo604) and 77 spacers (Xo21). Consequently, the small size of the *X. citri* pv. *citri* CRISPR loci allowed using simple conventional PCR to resolve the genetic diversity of different *X. citri* pv. *citri* strains. The PCR screening revealed considerable size variation of CRISPR loci among strains of *X. citri* pv. *citri*, suggesting that these loci consist of different numbers of spacer/repeat units due to deletion or acquisition of spacers based on their evolutionary history. Analysis of spoligotypes showed that most *X. citri* pv. *citri* strains share 23 or fewer spacers except for CFBP 2911, and that the leader-proximal spacer, which corresponds to the most recently acquired spacer, is conserved in most *X. citri* pv. *citri* strains (Fig. 2). This means that these strains only differ due to loss of one or more of the 23 spacers. The fact that 23 unique sequences built up the repertoire of spacers suggests that this set of strains originated from a common ancestor that harbored all the 23 spacers. Strain CFBP 2852 represents the oldest isolate in our set of strains (Table 3), yet it lacks already spacer Xcc_14. It would be interesting to go further back in time by analyzing herbarium specimen that date back to the beginning of the twentieth century and to analyse their repertoire of spacers [52].

**Table 3:** Origin and relevant characteristics of strains used in this study. Pathotype b indicates that this strain belongs to the pathovar *Xanthomonas citri* pv. *bilvae*. Pathotype A: wide host range on *Citrus* and other related genera, worldwide distribution. Pathotype A*: narrow host range: limes (*Citrus aurantifolia*) and alemow (*Citrus macrophylla*), limited areas of distribution. Pathotype A^w^: narrow host range, limes (*C. aurantifolia*), hypersensitive response on grapefruit

No.	Strain	Pathotype	Geographic origin	Isolation host	Year isolated	GenBank acc. no.
1	NCPPB 3213	b	India	N/A	1982	CDHI01
2	IAPAR306	A	Brazil	*Citrus sinensis*	1997	AE008923
3	C40	A	Reunion Island	*C. sinensis*	1988	CCWX01
4	CFBP 2852	A	India	*Citrus* sp.	< 1958	CCWI01
5	FDC217	A	Brazil	*C. sinensis*	2003	CCWY01
6	FDC1083	A	Brazil	*Citrus reticulata*	1980	CCVZ01
7	JJ238–10	A	Maldives Islands	*Citrus aurantifolia*	1987	CCWC01
8	JW160–1	A	Bangladesh	*C. aurantifolia*	2000	CCWH01
9	LMG 9322	A	Florida, USA	*C. aurantifolia*	1989	CCVY01
10	NCPPB 3562	A	India	*Citrus limon*	1988	CCXZ01
11	LC080	A	Mali	*C. reticulata* x *C. sinensis*	2006	CCWJ01
12	CFBP 2911	A*	Pakistan	*Citrus* sp.	1984	CCWD01
13	JF090–2	A*	Oman	*C. aurantifolia*	1986	CCWA01
14	JF090–8	A^w^	Oman	*C. aurantifolia*	1986	CCWB01
15	JJ238–24	A*	Thailand	*C. aurantifolia*	1989	CCVX01
16	JK002–10	A*	Saudi Arabia	*C. aurantifolia*	1988	CCWV01
17	JS584	A*	Iran	*Citrus* sp.	1997	CCWF01
18	LD007–1	A	Mali	*C. aurantifolia*	2007	CDAL01
19	NCPPB 3608	A^w^	India	*C. aurantifolia*	1988	CCWG01
20	LB305 = X2003–3218	A^w^	Florida, USA	*Citrus* sp.	2003	CCWL01
21	LE020–1	A*	Ethiopia	*C. aurantifolia*	2008	CCWK01
22	LH001–3	A	Pakistan	*Citrus* sp.	2010	N/A
23	LH037–1	A	Senegal	*Citrus paradisi*	2010	CDAS01
24	LG117	A	Bangladesh	*Citrus* sp.	2009	CDAX01
25	LB100–1	A	Seychelles	*C. sinensis* x *Poncirus trifoliata*	2005	CDAV01
26	JJ010–1	A	Rodrigues Island	*C. aurantifolia*	1985	CDDV01
27	JK004–1	A	China	*Citrus* sp.	< 1989	CDMR01
28	JK148–10	A	Philippines	*Citrus madurensis*	1990	N/A
29	CFBP 2900	A	Japan	*Citrus* sp.	< 1976	N/A
30	JS582	A*	Iran	*Citrus latifolia*	1997	CDAP01
31	JS555	A*	Iran	*C. paradisi*	1997	N/A
32	JS552	A*	Iran	*C. sinensis*	1997	N/A
33	LG115	A^w^	India	*C. aurantifolia*	2007	CDAY01
34	LG116	A^w^	India	*C. aurantifolia*	2006	N/A
35	LG100	A^w^	India	*C. aurantifolia*	2006	N/A
36	JK051	A*	Saudi Arabia	*C. aurantifolia*	1988	N/A
37	JK002–14	A*	Saudi Arabia	*C. aurantifolia*	1988	N/A
38	JM035–2	A*	Saudi Arabia	*C. aurantifolia*	< 1990	CDMS01
39	JK143–05	A*	Thailand	*C. aurantifolia*	< 1990	N/A
40	JK143–09	A*	Thailand	*Citrus* sp.	< 1990	CDMQ01
41	JK143–11	A*	Thailand	*Citrus* sp.	< 1990	CDMO01
42	LD071A	A*	Cambodia	*Citrus* sp.	2007	CCWE01
43	NCPPB 3607	A*	India	*C. aurantifolia*	1988	CDAT01
44	NCPPB 3615	A*	India	*C. aurantifolia*	1989	CDAM01
45	LE116–1	A	Mali	*C. aurantifolia*	2008	CDHD01
46	LE117–1	A	Mali	*Citrus* sp.	2008	N/A
47	LE003–1	A*	Ethiopia	*C. aurantifolia*	2008	CDAI01
48	LE065–1	A*	Ethiopia	*C. aurantifolia*	2008	N/A
49	LB302	A^w^	Florida, USA	*Citrus* sp.	2002	CDAU01
50	LG097	A	Bangladesh	*C. limon*	2006	CDAK01
51	LG102	A	Bangladesh	*Citrus* sp.	2006	CDAN01
52	JS581	A*	Iran	*C. limon*	1997	CDAW01
53	JK048	A*	Saudi Arabia	*C. aurantifolia*	1988	CDAJ01
54	NCPPB 3612	A	India	*C. aurantifolia*	1988	CDAQ01
55	NCPPB 3610	A	India	*P. trifoliata*	1988	CDAO01
56	LE032–1	A*	Ethiopia	*C. aurantifolia*	2008	N/A
57	JF090–3	A*	Oman	*C. aurantifolia*	1986	N/A

N/A, not available

### Correlations among different DNA-based typing methods

Correlation analyses of AFLP versus CRISPR or minisatellite-based typing (MLVA-31) data revealed a fairly good congruence between these methods. We found more AFLP haplotypes (49 haplotypes) and MLVA-31 haplotypes (37 haplotypes) than CRISPR spoligotypes (25 haplotypes). Hence, the AFLP method appears to better resolve the genetic diversity among strains of *X. citri* pv. *citri* than the two other methods but suffers from technical limitations making interlaboratory comparisons difficult to achieve, a characteristic that precludes a wide use for epidemiosurveillance [17].

In general, strains belonging to a certain spoligotype clade in the CRISPR tree also cluster together in the AFLP tree (Fig. 3). Exceptions were two strains, LH001–3 (spoligotype 23) and LG117 (spoligotype 25), with exceptionally small numbers of spacers, 12 and 8, respectively, which might explain their misplacement in comparison to the AFLP, MLVA-31 and SNP analyses [17–19]. For instance, strain LH001–3 clusters with strains LD007–1, LE116–1, LE117–1, LH37–1 and NCPPB 3562 in the AFLP analysis. The latter five strains belong to spoligotype 15. Strikingly, a single recombinational event, leading to a deletion of spacers Xcc_11 to Xcc_21, could transform spoligotype 15 into spoligotype 23 of strain LH001–3. Evolutionary speaking, such a scenario would place both strains close to each other. Similarly, both AFLP and spoligotyping cluster strains CFBP 2852, JW160–1, LB100–1 (spoligotype 2), JF090–8, JJ238–10, LG100 (spoligotype 4), NCPPB 3612 (spoligotype 5), NCPPB 3610 (spoligotype 6), and LG116 (spoligotype 7). In addition, AFLP contains as well strain LG117 in this cluster. Again, just two recombinational events, deleting spacers Xcc_01 to Xcc_03 and spacers Xcc_12 to Xcc_23, could transform spoligotype 2 into spoligotype 25 of strain LG117.

Indeed, the used algorithm considers binary information about presence or absence of individual spacers and no software is publicly available to consider the minimal number of necessary mutations for tree construction based on spoligotype data. For example, strain NCPPB 3562 contains spacers Xcc_01 to Xcc_13 and spacers Xcc_19 to Xcc_23. In contrast, strain LH001–3 contains only spacers Xcc_01 to Xcc_10 and spacers Xcc_22 to Xcc_23, i.e. this strain lacks six spacers in comparison to strain NCPPB 3562 (Xcc_11, Xcc_12, Xcc_13, Xcc_19, Xcc_20, Xcc_21), thus resulting in a large apparent distance, which does not necessarily refect the ‘true’ evolutionary distance. However, incorrect placement of a small number of strains is a common feature of many genotyping techniques. This was observed for a few host-restricted strains (JF090–8 and a few relatives), which clustered with pathotype A genetic lineage 2 strains when assayed by minisatellite-based typing (MLVA-31), whereas SNP analysis from complete genome data unambiguously assigned them to pathotype A^w^ [18, 19]. These strains had been previously erroneously assigned to pathotype A*, as they had a Mexican lime-restricted host range and AFLP-based methods did not show any close genetic relatedness to other host-restricted A* or A^w^ strains [17]. This incorrect placement of a few strains both by spoligotyping and minisatellite-based typing may explain the lower Mantel value between these two techniques, as compared to the values obtained for each of these techniques when compared to AFLP (Table 2).

### Distinguishing pathotypes A and A*

Interestingly, pathotype A and pathotype A* strains of our dataset with different citrus host range can be distinguished from each other by the presence or absence of spacer Xcc_06, which corresponds to the first deletion event in the evolution of pathotype A* spoligotypes. Knowledge of pathotype is of importance for disease management and has consequences for regulation measures. However, conventional determination of pathotypes is laborious, as it requires assaying citrus plants. Moreover, some PCR-based techniques failed to accurately identify pathotype A* strains [53, 54]. Apart from whole genome sequence data, the most straightforward method for distinguishing pathotype A* from another *X. citri* pv. *citri* pathotype is currently MLVA-31 (or its derivative MLVA-12) targeting minisatellites [18, 19].

We suggest to consider spacer Xcc_06 as a first line of evidence for the identification of pathotype A* strains using a PCR combining a spacer Xcc_06-specific primer and a primer annealing to the conserved terminator region, which would be a highly discriminatory assay. Analysis of publicly available genomic resources further confirmed the interest of spacer Xcc_06 as a diagnostic marker. Yet, it cannot be ruled out that hitherto undiscovered spoligotypes exist that could undermine such a diagnostic PCR. It is therefore necessary to sequence more CRISPR arrays or genomes, which would (i) help in estimating the discriminatory power of such an approach at a given geographical scale and (ii) allow designing complementary PCR schemes, if necessary.

### Origin of spacers

CRISPR arrays represent a signature of the long history of interactions between bacteria and bacteriophages or other extrachromosomal genetic elements. To understand the evolution of CRISPR loci, it is of interest to know from where the spacer sequences derive. To elucidate their origin, we performed BLASTN searches against the NCBI GenBank. In addition to the hits in the CRISPR loci of completely sequenced *X. citri* pv. *citri* strains, we found significant hits between spacer sequences and five *Xanthomonas* bacteriophages, a finding that supports the principal mechanism of CRISPR immune system in bacteria. Homologies with *Xanthomonas* bacteriophage CP1 (GenBank accession number AB720063) were found for spacers Xcc_36, Xcc_28 and Xcc_25 (Additional file 7: Table S1). Four bacteriophages (CP1, CP2, CP115 and CP122) have been used for classification of *X. citri* pv. *citri* strains based on their sensitivity to phage for quarantine purposes [55, 56]. Strains from *X. citri* pv. *citri* were variable in their sensitivity to bacteriophages CP1 and CP2 [55, 57]. The studies of genomic analysis of bacteriophage CP1 and CP2 have reported that the CP1 DNA sequence was detected in the genome sequence of *X. campestris* bacteriophage phiL7 (GenBank accession number EU717894), *X. oryzae* bacteriophage OP1 (GenBank accession number AP008979) and *Xanthomonas* bacteriophage Xp10 (GenBank accession number AY299121) [45]. In addition, a sequence in the genomic contig of the *Ralstonia*-related blood disease bacterium R229 (GenBank accession number FR854082) was related to spacer Xcc_31; this sequence encodes a DNA-dependent DNA polymerase with homology to DNA polymerases of the *Xanthomonas*-specific bacteriophages phiL7, OP1 and Xp10 [58–60]. Possibly, the genomic sequence of the blood disease bacterium R229 corresponds to a prophage with similarity to *Xanthomonas*-specific bacteriophages. Therefore, spacer Xcc_31 was likely acquired from a bacteriophage. *Xanthomonas* bacteriophages f20-Xaj and f30-Xaj also matched with several spacers of the 14 unique spacers of strain CFBP 2911 (Additional file 7: Table S1). Those two bacteriophages are closely related to each other and belong to the same clade as *X. citri* pv. *citri* bacteriophage CP2 [47]. Taken together, this evidence supports the hypothesis that the aforementioned spacers have been acquired from alien DNA most likely derived from bacteriophage CP1 and CP2, which were originally isolated from *X. citri* pv. *citri* strains [61].

Using less stringent thresholds (E-value smaller than 1 and no minimum criterium with respect to coverage of the query sequence), we also found a match for spacer Xcc_37 in the *Xanthomonas* bacteriophage CP1, and a few more bacteriophage-related matches for spacers Xcc_31, Xcc_28, Xcc_27, Xcc_11, and Xcc_10 (Additional file 7: Table S1). With even less stringent criteria there are also matches with *Xanthomonas*-specific bacteriophages for spacers Xcc_34 (bacteriophage CP1), Xcc_32 (bacteriophage CP1), Xcc_11 (*Streptomyces* phage Yaboi), and Xcc_2 (*Microbacterium* phage MementoMori) (data not shown). However, as demonstrated in Additional file 7: Table S1, relaxing the threshold results in an increased number of matches in genomes of diverse bacteria. Therefore, we cannot conclude that these are bona fide homologs the sequences of which have been altered with the long time since these spacers were acquired, or if these are merely false positives.

Only four of the 23 older spacers matched to sequences in GenBank that did not correspond to the CRISPR arrays of *X. citri* pv. *citri*. In all four cases, homology to sequences from integrated prophages or from a filamentous bacteriophage was observed. It was surprising that none of the older and conserved 23 spacers matched to a sequence from a bacteriophage genome whereas all the observed hits of the CFBP 2911-specific spacers corresponded to sequences from bacteriophages that have been isolated over the last 50 years. It is not clear whether this observation is merely due to sampling effects or if it reflects the fact that the sources for the 23 old spacers got extinct and only a few of the homologous sequences were vertically inherited and thus preserved in the form of prophages or remnants thereof.

### Multiple genetic events have contributed to the CRISPR array diversity within *X. citri* pv. *citri*

It is interesting to note that these strains did not acquire new spacers after spacer Xcc_23. Only strain CFBP 2911 acquired 14 new spacers next to the leader sequence, which are not present in any other *X. citri* pv. *citri* strain that we have analysed (Fig. 2). This finding can be explained by three scenarios. The first explanation that these 14 new spacers were deleted in all *X. citri* pv*. citri* strains but CFBP 2911 is very unlikely because CFBP 2911 does not represent an ancestral clade at the root of *X. citri* pv. *citri* phylogeny [18]. Second, it is possible but unlikely as well that none of the 56 strains except for CFBP 2911 was challenged by alien DNA elements, such as bacteriophages or plasmids, since they had acquired spacer Xcc_23. We favor the third hypothesis that the CRISPR immunity system was mutationally inactivated in its ability to acquire new spacers in the ancestor of all 56 *X. citri* pv. *citri* strains in our dataset, yet the CRISPR/Cas system was maintained during evolution as a mechanism of protection against bacteriophage infection. Possibly, a revertant evolved which regained the function of spacer acquisition, giving rise to strain CFBP 2911. Given the important role of the Cas proteins for spacer acquisition in the CRISPR/Cas system, we compared the sequences of the *cas* gene cluster of strain CFBP 2911 with those of other strains. However, we did not find any differences in the Cas protein sequences between CFBP 2911 and the other strains that could explain the regained CRISPR/Cas activity in strain CFBP 2911 (Additional file 8: Figure S7). Interestingly, *csd1*/*cas8c* genes of the majority of strains suffer from a frame-shift mutation due to a short tandem repeat of two base pairs (AG). Yet, strain CFBP 2911 is not the only one that has an intact copy of this gene. Therefore, the reason why strain CFBP 2911 acquired 14 extra spacers is still unclear. For further insight it would be interesting to analyze more strains from the same region as CFBP 2911 (i.e. Pakistan) by assuming that they might have undergone the same evolutionary event(s).

In addition, we found two cases of IS element insertions in CRISPR loci of *X. citri* pv. *citri*. One insertion occurred in the repeat between spacers Xcc_20 and Xcc_21 (LB302, LB305, LG115 and NCPPB 3608,) and another insertion had occurred in spacer Xcc_18 (LG097) (Fig. 2, Additional file 3: Figure S3, Additional file 4: Figure S4 and Additional file 5: Figure S5). The first four strains originated from India (LG115, NCPPB 3608) and Florida (LB302, LB305). Notably, these strains were all assigned to pathotype A^w^ and genetic lineage 3 based on minisatellite typing [19]. Interestingly, the spacer Xcc_14 was deleted from strains, LB302, LB305 and LG115 whereas NCPPB 3608, probably representing the ancestral spoligotype of our dataset, had all 23 spacers. Our results thus further confirm an Indian origin of the A^w^ strains from Florida, in agreement with outbreak investigation and previously produced genotyping data [18, 19, 62]. Insertion of IS elements can therefore be another source of polymorphism as frequently observed in the CRISPR locus of *M. tuberculosis* [63, 64]. Depending on the spoligotyping scheme, insertion of an IS element into either the direct repeat or spacer sequences can influence the spoligotype pattern, resulting in apparent deletion of CRISPR sequence [65]. In such cases, binary data of the spoligotype might be unable to provide sufficient information to accurately establish genotypic relationships among bacterial isolates. This limitation needs to be considered when using spoligotyping data for molecular epidemiological strain tracking and phylogenetic analyses of pathogens [65].

### Genealogy of CRISPR spoligotypes

**Fig. 4:**
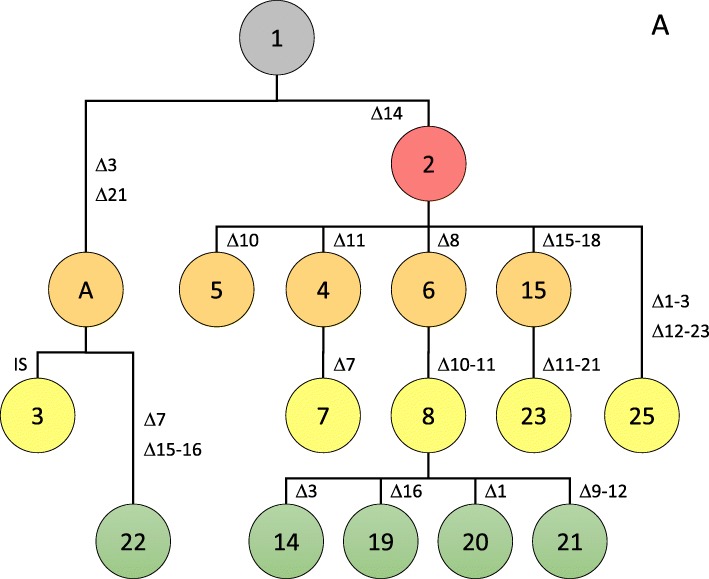
Genealogy of spoligotypes from pathotype A strains*.* Postulated mutational events leading to the observed spoligotypes are indicated, starting from the ancestral spoligotype with all 23 spacers (Fig. 2) shown in grey on the top, with the colors indicating the number of events (from one to four events, colored in salmon, orange, yellow, and green, respectively). Numbers of observed haplotypes are indicated in the circles. Characters indicate postulated intermediate haplotypes that were not observed among the 56 analysed strains

**Fig. 5:**
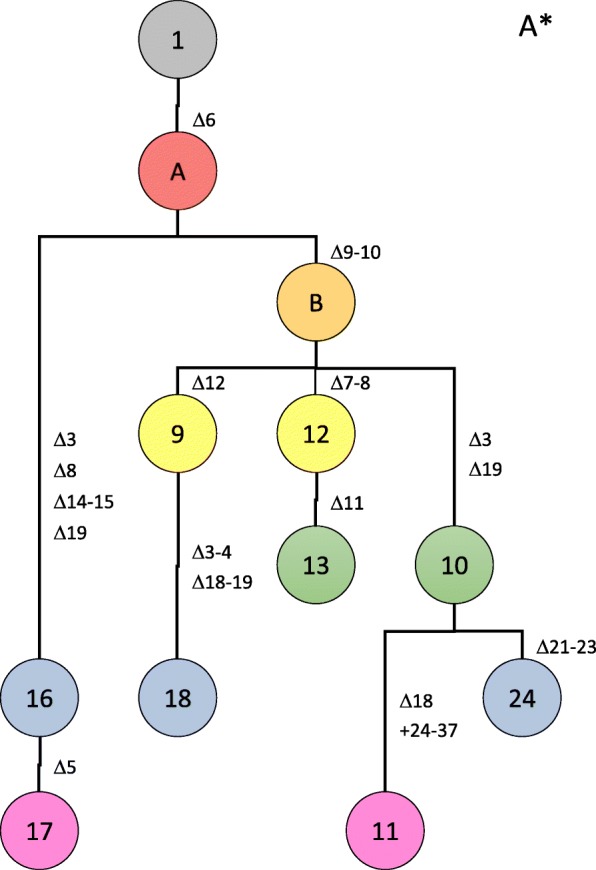
Genealogy of spoligotypes from pathotype A* strains. Postulated mutational events leading to the observed spoligotypes are indicated, starting from the ancestral spoligotype with all 23 spacers (Fig. 2) shown in grey on the top, with the colors indicating the number of events (from one to six events, colored in salmon, orange, yellow, green, blue and purple, respectively). Numbers of observed haplotypes are indicated in the circles. Characters indicate postulated intermediate haplotypes that were not observed among the 56 analysed strains

Since the CRISPR array of all strains originated from a conserved array of 23 spacers, one can use this information to establish an evolutionary trajectory among the observed spoligotypes. To building such an evolutionary pathway one could assume to minimize the number of mutational events that are necessary to connect all spoligotypes with each other. Yet, without additional information it is impossible to be absolutely certain about a given scenario because several alternatives might exist with a similar number of postulated mutation (deletion) events. Here, we took advantage of the availability of genome sequence data for 42 out of the 56 *X. citri* pv. *citri* strains, which were used to build a robust phylogenetic tree based on whole genome alignment upon removal of regions with signs of recombination [18]. These data provided information about the evolutionary relationships among 21 spoligotypes. Only spoligotypes 7, 13, 20, 21, and 23 were not covered by full genome data. In these cases, information was taken from global studies using AFLP and MLVA data [17, 19]. Based on these phylogenetic datasets, which can be considered as evolutionary neutral, we were able to manually build trees for all observed spoligotypes, with one tree representing pathotype A and another tree representing pathotype A* strains (Figs. 4 and 5). Future work including strains representing a larger temporal scale, e.g. from herbarium specimen [52], together with approaches to build time-calibrated phylogenies will help to assess the speed of the molecular CRISPR clock [66].

The phylogenetic trees for pathotype A and A* strains demonstrate the utility and power of spoligotyping in order to assess the genealogy of bacterial strains. The pathotype A strains fall into two clades that are distinguished by three early deletion events (Fig. 4). One clade consists of two strains from Bangladesh, LG097 and LG102 (spoligotypes 3 and 22, respectively). These two spoligotypes likely derived from a hypothetical intermediate spoligotype (missing link labelled “A” in Fig. 4) that lacks spacers Xcc_03 and Xcc_21. The second clade consists of strains that all lack spacer Xcc_14. Loss of spacer Xcc_14, which is represented by strains from India, Bangladesh and the Seychelles (spoligotype 2), can thus be considered as an early event in the evolution of this clade, possibly in connection with the Indian subcontinent being regarded as a likely area of origin of *X. citri* pv. *citri* [19, 67]. Interestingly, this clade contains two spoligotypes that correspond to strains from West Africa, spoligotype 15 (which also contains strain NCPPB 3562 from India) and spoligotype 14 (which also contains three strains from Brazil, FDC2017, FDC1083 and IAPAR306). Since all the West African strains were isolated after 2005 while the other strains have been isolated up to 25 years earlier it is temping to speculate that *X. citri* pv. *citri* has been introduced in West Africa at least two times, once from the Indian subcontinent and once from South America. Strikingly, this observation is backed by (i) mini- and microsatellite data where spoligotype 15 corresponds to DAPC cluster 2 and spoligotype 14 corresponds to DAPC cluster 1 [21], and (ii) by whole genome data [18].

The pathotype A* strains fall into two clades that are distinguished by the presence or absence of spacers Xcc_9 and Xcc_10 (Fig. 5). One clade is restricted to strains from Cambodia and Thailand (spoligotypes 16 and 17), which result from an evolutionary pathway that involved at least four spacer/repeat deletion events (spacers Xcc_03, Xcc_08, Xcc_14/Xcc_15, Xcc_19). The other clade shows as well a strong geographic structuring. Spoligotype 18, which only contains strain from Iran, probably evolved by two deletion events (spacers Xcc_03/Xcc_04 and spacers Xcc_18/Xcc_19) from spoligotype 9, which only contains strains from Saudi Arabia. Spoligotypes 12 and 13 are restricted to strains from Ethiopia while spoligotypes 11 and 24 correspond to strains from Pakistan and India, respectively, with their ancestral spoligotype 10 consisting of strains from India, Oman and Saudi Arabia. These findings are consistent with previous minisatellite and whole genome sequence analyses [18, 19].

Five of the seven pathotype A^w^ strains have been sequenced and allow as well their phylogenetic reconstruction [18]. Strain JF090–8 from Oman (1986) diverged early and its spoligotype 4 can be considered as the ancestor of spoligotype 7, which underwent a subsequent deletion of spacer Xcc_7 (strain LG116 from India, 2006). Spoligotype 1*, as represented by strain NCPPB 3608 from India (1988) and which contains all 23 ancestral spacers, can be considered as the founder of a distinct clade which is characterized by the acquisition of an IS element between spacers Xcc_20 and Xcc_21. Genomic data indicate that strains LG115 (India, 2007), LB302 (Florida, USA, 2002) and LB305 (Florida, USA, 2003), corresponding to spoligotype 2*, are descendants of a spoligotype-1* strain that underwent a deletion of spacer Xcc_14 [18]. Therefore and because of their geographic separation it is likely that the deletion of spacer Xcc_14 in spoligotypes 2* and 4 were independent events; hence, effects of homoplasy need to be considered when drawing conclusions from spoligotyping. Nevertheless, we conclude that CRISPR elements provide a new and useful framework for the genealogy of the citrus canker pathogen *X. citri* pv. *citri*.

## Conclusions

This study provides the necessary information to set up a spoligotyping scheme and a spoligotyping database for *X. citri* pv. *citri*, similar to the well-established spoligotyping scheme for *M. tuberculosis* [37]. It demonstrated the advantages and disadvantages of a CRISPR-based typing method. In order to facilitate future work and comparisons we have deposited all CRISPR typing data in the MLVAbank under the name “Xanthomonas_citri_CRISPR” (http://www.biopred.net/MLVA/) [68]. In accordance with previous studies [28, 42], we confirmed that CRISPR-based typing can be an efficient and robust method to study the evolution of bacterial isolates and to resolve the phylogenetic relationship among strains. We confirmed that CRISPR loci can differ among strains due to bacteriophage exposure, IS element insertion and intra-locus recombination leading to loss of spacer/repeat units, thus giving a valuable typing tool as well [42]. Moreover, the CRISPR-based typing method is easier to perform and more reproducible than AFLP and rep-PCR methods since it can be performed with a simple conventional PCR approach and results in robust binary data.

Genotyping-based surveillance is informative for assessing the geographical expansion of plant pathogenic bacteria, their prevalence, and to identify new strains, especially in the case of regulated pathogens such as *X. citri* pv. *citri*. We therefore consider our new typing method as a valuable tool for further studies and conclude that, if complete genome sequence data cannot be made available, a combined use of minisatellite and CRISPR-based typing, two techniques combining overall fairly good phylogenetic signals, discriminatory power and portability, should be preferred for placing strains associated with new outbreaks in the global diversity of *X. citri* pv. *citri*. The correct identification of outbreak strains is a critical issue, as there are marked differences in biological features (e.g., host range) and agricultural significance among genetic lineages (in relation with the pathotype classification), which affect the options possibly taken in terms of disease management [5, 62].

## Methods

### Isolation of genomic DNA

The collection of 56 *X. citri* pv. *citri* strains used in this study is representative of the worldwide genetic and pathological diversity of *X. citri* pv. *citri* [18]. The strains originated from Asia (Bangladesh, Cambodia, China, India, Iran, Japan, Oman, Pakistan, Philippines, Saudi Arabia and Thailand), Africa (Ethiopia, Mali and Senegal), North America (Florida-USA), South America (Brazil) and some islands in the Indian Ocean (Maldives, Reunion Island, Rodrigues and Seychelles) (Table 3). Genomic DNA of *X. citri* pv. *citri* and one strain of *X. citri* pv. *bilvae* were extracted as previously described [18]. The concentration of genomic DNA samples was approximately 500 ng/μl. Each DNA was diluted to 20 ng/μl. DNA quantification was done using a nanodrop device (spectrophotometer ND 1000; Labtech France). Purity of DNA was confirmed by 1.0% agarose gel electrophoresis, stained with ethidium bromide and visualized on a UV transilluminator.

### Genomic information

Genomic information for 86 *X. citri* pv. *citri* strains is publicly available (without counting doublets), including 31 complete genome sequences (https://www.ncbi.nlm.nih.gov/assembly/?term=Xanthomonas%20citri%20pv.%20citri; queried on July 30, 2019). In this study, we have accessed all genome sequences (Additional file 9: Table S2; Additional file 10: Table S3) to screen for the presence of CRISPR loci. Among them, we used the draft genomes of 42 strains out of the 56 strains tested in this study to confirm our PCR amplification data (Additional file 10: Table S3) [18].

### PCR amplification

A primer pair targeting the *cas1* gene of several *Xanthomonas* species (*Xanthomonas albilineans*, *X. citri* pv. *citri*, *X. oryzae*), resulting in an amplicon of 221 bp, was designed and used to evaluate the presence of the CRISPR/Cas system in strains of *X. citri* pv. *citri* (Table 4). PCR primers corresponding to the leader and terminator regions of the CRISPR locus were designed based on five genome sequences of *X. citri* pv. *citri* and expected to amplify the whole of CRISPR array (Table 4; Additional file 11: Figure S8A). In cases where we could not amplify and/or sequence the full-length CRISPR array, we designed PCR primers corresponding to internal regions of the CRISPR array. Specifically, we designed two forward primers targeting spacers Xcc_21 and Xcc_19 and two reverse primers targeting spacers Xcc_18 and Xcc_02, counting from the terminator of the CRISPR locus (Table 4; Additional file 11: Figure S8B).

**Table 4:** List of oligonucleotides

Name	Sequence (5′ → 3′)	Purpose
Cas1_fw	GCGCGCGGCTGGCGCGA	Detection of *cas1* gene
Cas1_rev	CGGCGATTGCGTCCGCC	
Leader_fw	TCACGGGGTCCGCATGAC	PCR amplification of CRISPR array
Terminator_rev	CTCGTCAGCGTCCGGCTG	
Spacer#19_fw	CGAGCGCATCGATGACGG	PCR amplification of internal region of the CRISPR array
Spacer#21_fw	TCGGGTTTCGGGATGTGC	
Spacer#02_rev	CCGGGACGGCGACGAAC	
Spacer#18_rev	CGTCGCTGTCGGTGGCAC	
IS-1_rev	ACCAGCGCCAGCAGCGG	PCR amplification of internal region of the CRISPR array next to an IS element
IS-2_fw	GCCGACCTGATGATGCA	

PCR results on CRISPR loci indicated the presence of an insertion sequence (IS) element within the CRISPR array of a few strains, including NCPPB 3608. Based on genome sequence data, we designed specific primers corresponding to conserved regions of the IS element (Table 4). Several primer combinations were used to determine the position of the IS element and to elucidate the presence and order of CRISPR spacers, e.g., combinations Leader_fw and IS-1_rev, IS-2_fw and Spacer#18_rev, IS-2_fw and Terminator_rev (Additional file 12: Figure S9).

PCR amplifications were performed with a 2720 thermal cycler version 2.08 (Applied Biosystems, USA) in a final volume of 25 μl containing 10 mM Tris-HCl (pH 8.5), 50 mM KCl, 1.5 mM MgCl_2_, 0.01% gelatine, 0.2 mM of each dNTP, 10 μM of each primer, and 0.25 units of GoTaq*®* DNA polymerase (Promega, France). Approximately 20 ng of genomic DNA were added to the PCR mixture. All PCR protocols included an initial denaturation step of 1 min at 95 °C, 30 cycles of a denaturation step of 2 min at 94 °C, an annealing step of 30 s at 55 °C, an elongation step of 2 min at 72 °C and a final extension step of 2 min at 72 °C.

### DNA purification and sequencing

If required, PCR amplicons were purified using the commercial QIAquick Gel Extraction kit (QIAGEN, France). All PCR products were sequenced by Beckman Genomic Inc. (UK), with primers used for PCR amplification. In cases were the PCR amplicon could not be completely sequenced by the PCR primers, amplicons were re-sequenced using internal primers corresponding to the spacers Xcc_21, Xcc_19, Xcc_18 and Xcc_02, depending on the missing CRISPR regions.

### Bioinformatic and statistical analyses

The obtained DNA sequences were edited and assembled using the CAP 3 program [69], using default parameters. CRISPR spacers and repeats were identified using CRISPRCasFinder (https://crisprcas.i2bc.paris-saclay.fr/CrisprCasFinder/Index) [25] (Table 1). CRISPR spacers and repeats were annotated on the *X. citri* pv. *citri* sequences using Artemis Release 17.0.1 (http://www.sanger.ac.uk/science/tools/artemis/) [70]. To compare the CRISPR loci of the 56 *X. citri* pv. *citri* strains, we represented the spacers by different colors, an approach that was called “spacers crawling” in a similar study on *E. amylovora* [42]. To elucidate the origin of spacer sequences, we performed BLASTN searches in the non-redundant NCBI database (https://blast.ncbi.nlm.nih.gov/Blast.cgi), using the following parameters: E-value threshold 0.1, word size 7, default mismatch and gap penalties (i.e. match/mismatch scores: 2, − 3; gap costs: 5 for existence, 2 for extension), no filter for low complexity regions. Only hits with at least 90% coverage were retrieved. Hits in eukaryotic organisms were excluded. For IS element identification, the *IS-Finder* database (https://www-is.biotoul.fr/) was used.

In order to compare the resolution and discriminatory power of CRISPR typing with other typing methods, MLVA-14 (microsatellite), MLVA-31 (minisatellite) and AFLP genotyping data were retrieved from our previous studies [17, 19, 21]. The discriminatory power of MLVA-14, MLVA-31, AFLP and CRISPR was calculated based on Hunter’s index (D) [71]. Distance matrices of each genotyping method were compared with a Mantel test using the ‘CADM.Post’ functions of ‘APE’ package (9999 permutations) in *R* [72].

### Comparison of distance tree analysis between AFLP and CRISPR typing

AFLP data used for comparison were derived from a previous study [17]. In order to produce a distance tree for 56 strains, a presence/absence matrix was produced based on the distribution of the polymorphism of fragment for AFLP and the distribution of CRISPR spacers for CRISPR typing, respectively. These matrices were analyzed with the DendroUPGMA program (http://genomes.urv.es/UPGMA/) using the Dice similarity coefficient and the UPGMA (Unweighted Pair Group Method with Arithmetic Mean) method [73]. The FigTree program was used to visualize the distance tree map (version 1.4.2; http://tree.bio.ed.ac.uk/software/figtree/).

## Supplementary information

**Additional file 1: Figure S1.** PCR amplification of a 220-bp *cas1* gene fragment from strains of *X. citri* pv. *citri*. M, molecular weight marker (1-kb ladder, Promega); n, negative control (PCR reaction without template DNA). A, strains no. 1–22 of Table 3; B, strains no. 23–44 of Table 3; C, strains no. 45–57 of Table 3. The red box indicates *X. citri* pv. *bilvae* strain NCPPB 3213.

**Additional file 2: Figure S2.** PCR amplification of CRISPR arrays from *X. citri* pv. *citri.* M, molecular weight marker (λ DNA/EcoRI + HindIII, Promega); n, negative control (PCR reaction without template DNA). A, strains no. 1–20 of Table 3; B, strains no. 21–40 of Table 3; C, strains no. 41–57 of Table 3.

**Additional file 3: Figure S3.** Structure of the CRISPR array of *X. citri pv. citri* strain NCPPB 3608. Red characters indicate direct repeat sequences, with SNPs underlined. Blue characters indicate spacer sequences. 6 bp (tgaaac) in green boxes represent the target site duplication. Pink boxes represent the inverted repeats (28 bp). Blue boxes represent base pairs that do not match within the inverted repeats.

**Additional file 4: Figure S4.** Structure of the CRISPR array of *X. citri pv. citri* strains LB302, LB305 and LG115. Red characters indicate direct repeat sequences, with SNPs underlined. Blue characters indicate spacer sequences. 6 bp (tgaaac) in green boxes represent the target site duplication. Pink boxes represent the inverted repeats (28 bp). Blue boxes represent base pairs that do not match within the inverted repeats.

**Additional file 5: Figure S5.** Structure of the CRISPR array of *X. citri pv. citri* strain LG097. Red characters indicate direct repeat sequences, with SNPs underlined. Blue characters indicate spacer sequences. 6 bp (cctgca) in green boxes represent the target site duplication. Pink boxes represent the inverted repeats (28 bp). Blue boxes represent base pairs that do not match within the inverted repeats. Spacer Xcc_18*: 4 bp, indicated by dashes, are deleted due to the IS element insertion.

**Additional file 6: Figure S6.** PCR amplification of spacer/repeat units next to the IS element of five *X. citri* pv. *citri* strains*.* M, molecular weight marker (1-kb ladder, Promega); n, negative control (PCR reaction without template DNA). Lanes 2–7, primer combination Leader_fw and IS-1_rev; lanes 9–14, primer combination IS-2_fw and Spacer#18_rev.

**Additional file 7: Table S1.** BLASTN database searches for spacer-related sequences at NCBI GenBank.

**Additional file 8: Figure S7.** Multiple sequence alignments of the seven *cas* gene open reading frames, which were retrieved from the corresponding GenBank files (Table 3).

**Additional file 9: Table S2.** List of full-length genome resources used in this study.

**Additional file 10: Table S3.** List of draft genome resources used in this study.

**Additional file 11: Figure S8.** Primer design for PCR amplification of the CRISPR array from *X. citri* pv. *citri*. A, amplification of the full-length CRISPR arrays using primers Leader_fw and Terminator_rev. B, amplification to internal regions of the CRISPR arrays using spacer-specific primers. Forward primers are shown as red rectangles with an arrow, reverse primers are represented by blue rectangles with an arrow.

**Additional file 12: Figure S9.** Primer design for PCR amplification of CRISPR regions adjacent to an IS element. A, primer combinations used for strains LB302, LB305, LG115 and NCPPB 3608; B, primer combinations used for strain LG097. Forward primers are shown as red rectangles with an arrow, reverse primers are represented by blue rectangles with an arrow. The yellow triangle indicates the leader sequence and the red triangle indicates the terminator sequence. Deep blue rectangles indicate CRISPR repeats. CRISPR spacers are represented by green diamonds. Spacers are numbered from 1 to 23, starting with the terminator proximal spacer, which presumably represents the oldest spacer. Orange rectangles indicate IS elements.

## Data Availability

All CRISPR typing data have been deposited in the MLVAbank under the name “Xanthomonas_citri_CRISPR” (http://www.biopred.net/MLVA/).

## Abbreviations

AFLP: Amplified fragment length polymorphism
BLAST: Basic local alignment search tool
CRISPR: Clustered Regularly Interspaced Short Palindromic Repeats
DAPC: Discriminant analysis of principal components
MLVA: Multilocus VNTR analysis
NCBI: National Center for Biotechnology Information
NGS: Next generation sequencing
rep-PCR: Repetitive element-polymerase chain reaction
RFLP: Restriction fragment length polymorphism
SNP: Single-nucleotide polymorphism
UPGMA: Unweighted pair group method with arithmetic mean
VNTR: Variable-number of tandem-repeats
