# Supplementary material for: CRISPR elements provide a new framework for the genealogy of the citrus canker pathogen Xanthomonas citri pv. citri
Source: BMC Genomics. 2019 Dec 2;20:917. doi: 10.1186/s12864-019-6267-z (PMC6889575; doi:10.1186/s12864-019-6267-z)
Supplement: Supplementary file 1 — Additional file 1: Figure S1. PCR amplification of a 220-bp cas1 gene fragment from strains of X. citri pv. citri. M, molecular weight marker (1-kb ladder, Promega); n, negative control (PCR reaction without template DNA). A, strains no. 1–22 of Table 3; B, strains no. 23–44 of Table 3; C, strains no. 45–57 of Table 3. The red box indicates X. citri pv. bilvae strain NCPPB 3213. [file 12864_2019_6267_MOESM1_ESM.pptx]

## Slide 1
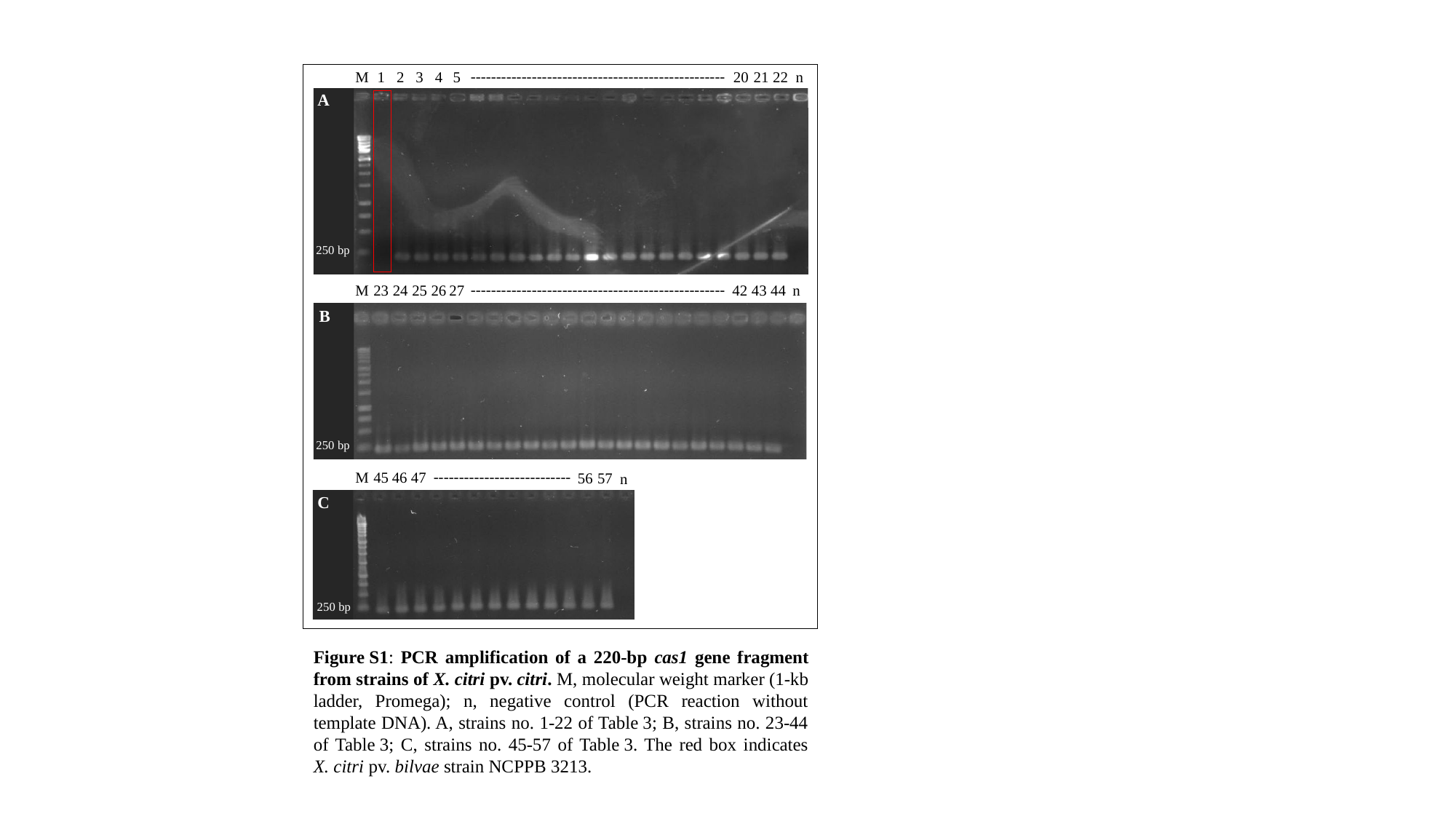

--------------------------------------------------
n
22
5
21
4
20
3
2
1
M
250 bp
--------------------------------------------------
n
44
27
43
26
42
25
24
23
M
250 bp
n
---------------------------
47
46
45
M
56
57
250 bp
A
B
C
Figure S1: PCR amplification of a 220-bp cas1 gene fragment from strains of X. citri pv. citri. M, molecular weight marker (1-kb ladder, Promega); n, negative control (PCR reaction without template DNA). A, strains no. 1-22 of Table 3; B, strains no. 23-44 of Table 3; C, strains no. 45-57 of Table 3. The red box indicates X. citri pv. bilvae strain NCPPB 3213.
