# Supplementary material for: CRISPR elements provide a new framework for the genealogy of the citrus canker pathogen Xanthomonas citri pv. citri
Source: BMC Genomics. 2019 Dec 2;20:917. doi: 10.1186/s12864-019-6267-z (PMC6889575; doi:10.1186/s12864-019-6267-z)
Supplement: Supplementary file 2 — Additional file 2: Figure S2. PCR amplification of CRISPR arrays from X. citri pv. citri. M, molecular weight marker (λ DNA/EcoRI + HindIII, Promega); n, negative control (PCR reaction without template DNA). A, strains no. 1–20 of Table 3; B, strains no. 21–40 of Table 3; C, strains no. 41–57 of Table 3. [file 12864_2019_6267_MOESM2_ESM.pptx]

## Slide 1
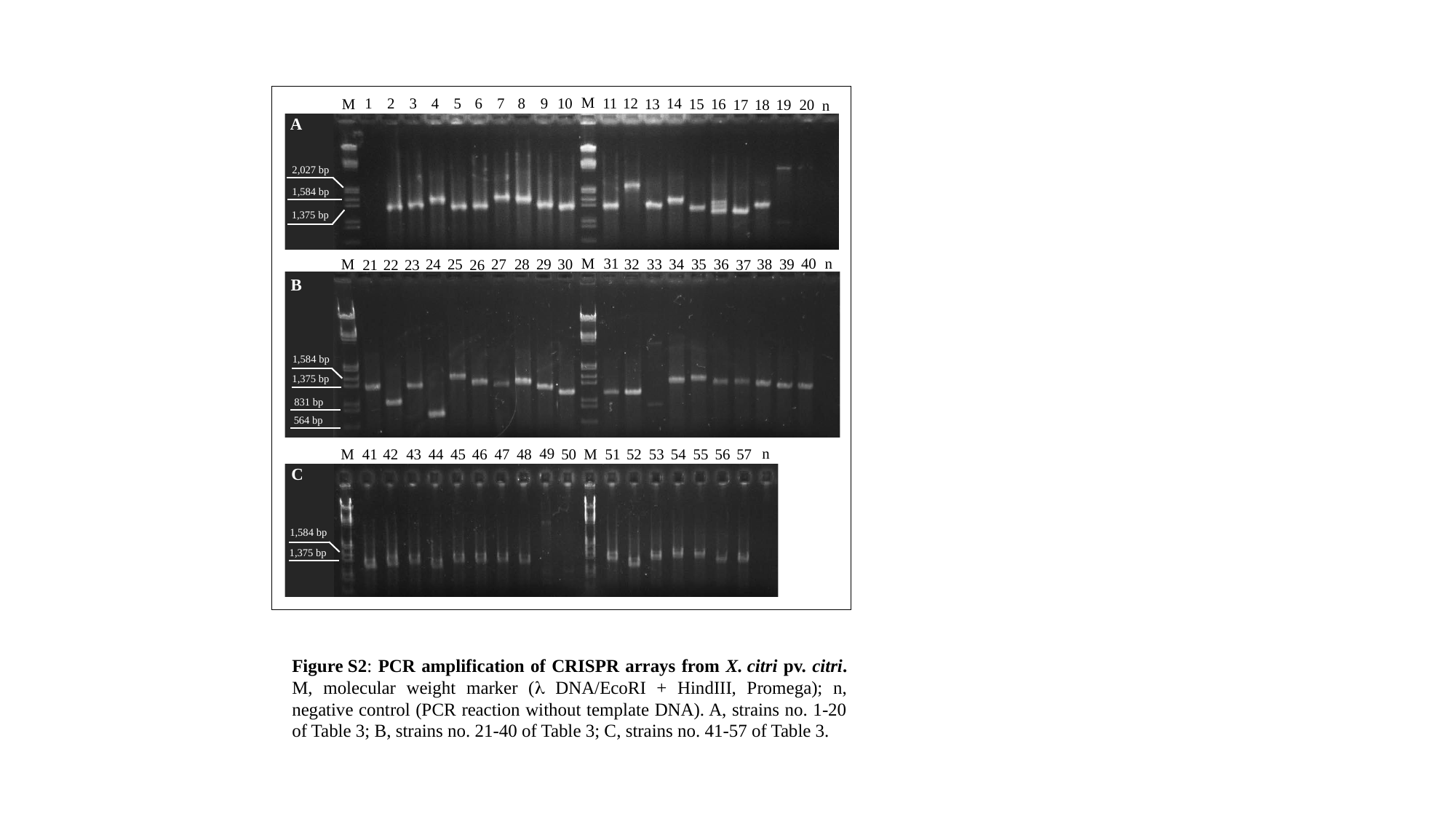

M
11
8
14
7
3
4
5
9
10
12
1
2
6
13
16
15
M
17
20
18
19
n
M
31
40
n
29
30
32
M
28
33
38
39
34
36
35
27
24
25
21
22
26
37
23
49
n
M
41
42
47
55
56
51
52
54
57
53
46
M
43
44
48
45
50
A
2,027 bp
1,584 bp
1,375 bp
B
1,584 bp
1,375 bp
831 bp
564 bp
C
1,584 bp
1,375 bp
Figure S2: PCR amplification of CRISPR arrays from X. citri pv. citri. M, molecular weight marker (l DNA/EcoRI + HindIII, Promega); n, negative control (PCR reaction without template DNA). A, strains no. 1-20 of Table 3; B, strains no. 21-40 of Table 3; C, strains no. 41-57 of Table 3.
