# Supplementary material for: CRISPR elements provide a new framework for the genealogy of the citrus canker pathogen Xanthomonas citri pv. citri
Source: BMC Genomics. 2019 Dec 2;20:917. doi: 10.1186/s12864-019-6267-z (PMC6889575; doi:10.1186/s12864-019-6267-z)
Supplement: Supplementary file 3 — Additional file 3: Figure S3. Structure of the CRISPR array of X. citri pv. citri strain NCPPB 3608. Red characters indicate direct repeat sequences, with SNPs underlined. Blue characters indicate spacer sequences. 6 bp (tgaaac) in green boxes represent the target site duplication. Pink boxes represent the inverted repeats (28 bp). Blue boxes represent base pairs that do not match within the inverted repeats. [file 12864_2019_6267_MOESM3_ESM.pptx]

## Slide 1
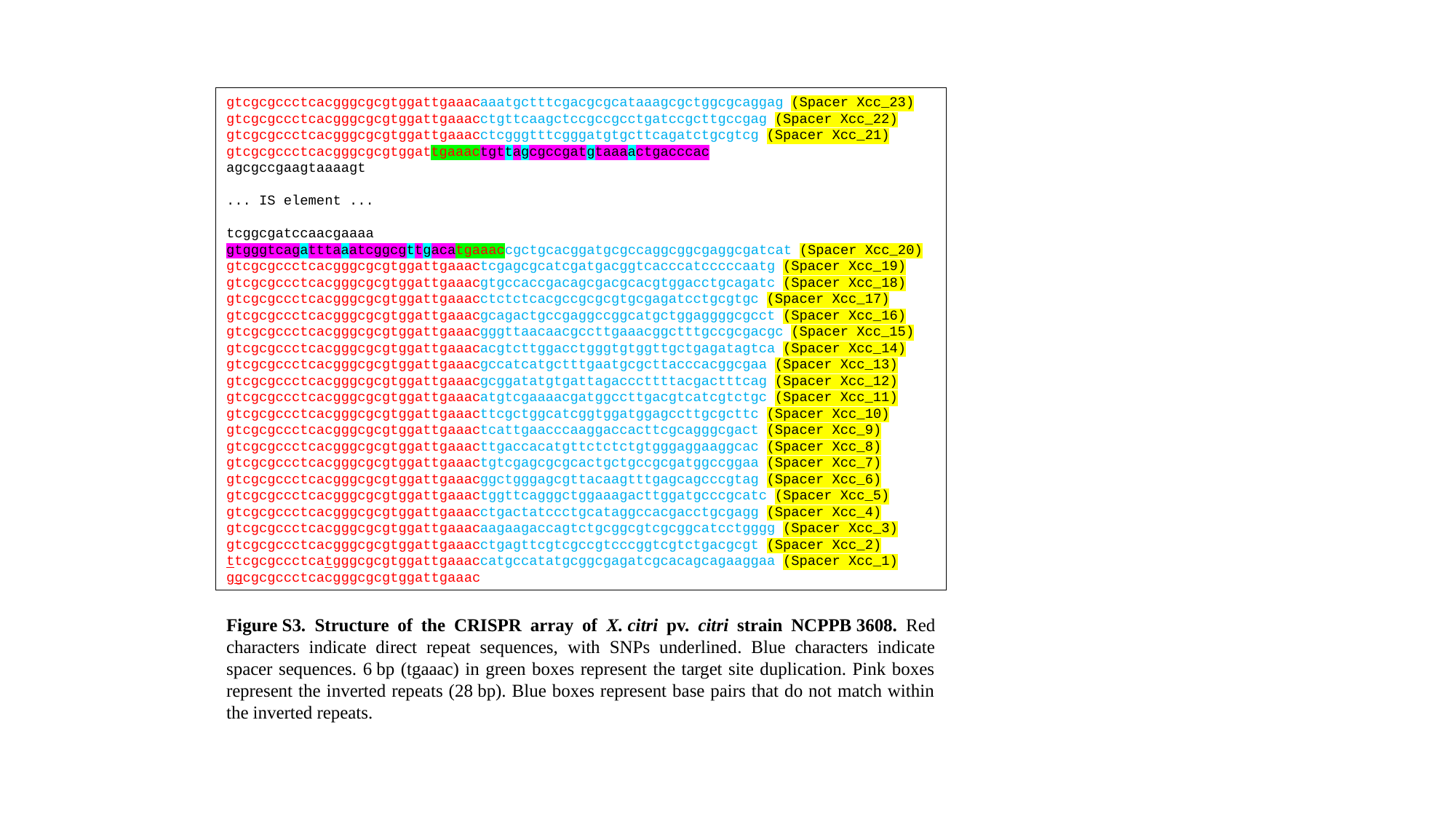

gtcgcgccctcacgggcgcgtggattgaaacaaatgctttcgacgcgcataaagcgctggcgcaggag (Spacer Xcc_23) gtcgcgccctcacgggcgcgtggattgaaacctgttcaagctccgccgcctgatccgcttgccgag (Spacer Xcc_22) gtcgcgccctcacgggcgcgtggattgaaacctcgggtttcgggatgtgcttcagatctgcgtcg (Spacer Xcc_21)
gtcgcgccctcacgggcgcgtggattgaaactgttagcgccgatgtaaaactgacccac
agcgccgaagtaaaagt
... IS element ...
tcggcgatccaacgaaaa
gtgggtcagatttaaatcggcgttgacatgaaaccgctgcacggatgcgccaggcggcgaggcgatcat (Spacer Xcc_20)
gtcgcgccctcacgggcgcgtggattgaaactcgagcgcatcgatgacggtcacccatcccccaatg (Spacer Xcc_19) gtcgcgccctcacgggcgcgtggattgaaacgtgccaccgacagcgacgcacgtggacctgcagatc (Spacer Xcc_18)
gtcgcgccctcacgggcgcgtggattgaaacctctctcacgccgcgcgtgcgagatcctgcgtgc (Spacer Xcc_17)
gtcgcgccctcacgggcgcgtggattgaaacgcagactgccgaggccggcatgctggaggggcgcct (Spacer Xcc_16)
gtcgcgccctcacgggcgcgtggattgaaacgggttaacaacgccttgaaacggctttgccgcgacgc (Spacer Xcc_15)
gtcgcgccctcacgggcgcgtggattgaaacacgtcttggacctgggtgtggttgctgagatagtca (Spacer Xcc_14)
gtcgcgccctcacgggcgcgtggattgaaacgccatcatgctttgaatgcgcttacccacggcgaa (Spacer Xcc_13)
gtcgcgccctcacgggcgcgtggattgaaacgcggatatgtgattagacccttttacgactttcag (Spacer Xcc_12)
gtcgcgccctcacgggcgcgtggattgaaacatgtcgaaaacgatggccttgacgtcatcgtctgc (Spacer Xcc_11)
gtcgcgccctcacgggcgcgtggattgaaacttcgctggcatcggtggatggagccttgcgcttc (Spacer Xcc_10)
gtcgcgccctcacgggcgcgtggattgaaactcattgaacccaaggaccacttcgcagggcgact (Spacer Xcc_9)
gtcgcgccctcacgggcgcgtggattgaaacttgaccacatgttctctctgtgggaggaaggcac (Spacer Xcc_8)
gtcgcgccctcacgggcgcgtggattgaaactgtcgagcgcgcactgctgccgcgatggccggaa (Spacer Xcc_7)
gtcgcgccctcacgggcgcgtggattgaaacggctgggagcgttacaagtttgagcagcccgtag (Spacer Xcc_6)
gtcgcgccctcacgggcgcgtggattgaaactggttcagggctggaaagacttggatgcccgcatc (Spacer Xcc_5)
gtcgcgccctcacgggcgcgtggattgaaacctgactatccctgcataggccacgacctgcgagg (Spacer Xcc_4)
gtcgcgccctcacgggcgcgtggattgaaacaagaagaccagtctgcggcgtcgcggcatcctgggg (Spacer Xcc_3)
gtcgcgccctcacgggcgcgtggattgaaacctgagttcgtcgccgtcccggtcgtctgacgcgt (Spacer Xcc_2)
ttcgcgccctcatgggcgcgtggattgaaaccatgccatatgcggcgagatcgcacagcagaaggaa (Spacer Xcc_1)
ggcgcgccctcacgggcgcgtggattgaaac
Figure S3. Structure of the CRISPR array of X. citri pv. citri strain NCPPB 3608. Red characters indicate direct repeat sequences, with SNPs underlined. Blue characters indicate spacer sequences. 6 bp (tgaaac) in green boxes represent the target site duplication. Pink boxes represent the inverted repeats (28 bp). Blue boxes represent base pairs that do not match within the inverted repeats.
