# Supplementary material for: CRISPR elements provide a new framework for the genealogy of the citrus canker pathogen Xanthomonas citri pv. citri
Source: BMC Genomics. 2019 Dec 2;20:917. doi: 10.1186/s12864-019-6267-z (PMC6889575; doi:10.1186/s12864-019-6267-z)
Supplement: Supplementary file 4 — Additional file 4: Figure S4. Structure of the CRISPR array of X. citri pv. citri strains LB302, LB305 and LG115. Red characters indicate direct repeat sequences, with SNPs underlined. Blue characters indicate spacer sequences. 6 bp (tgaaac) in green boxes represent the target site duplication. Pink boxes represent the inverted repeats (28 bp). Blue boxes represent base pairs that do not match within the inverted repeats. [file 12864_2019_6267_MOESM4_ESM.pptx]

## Slide 1
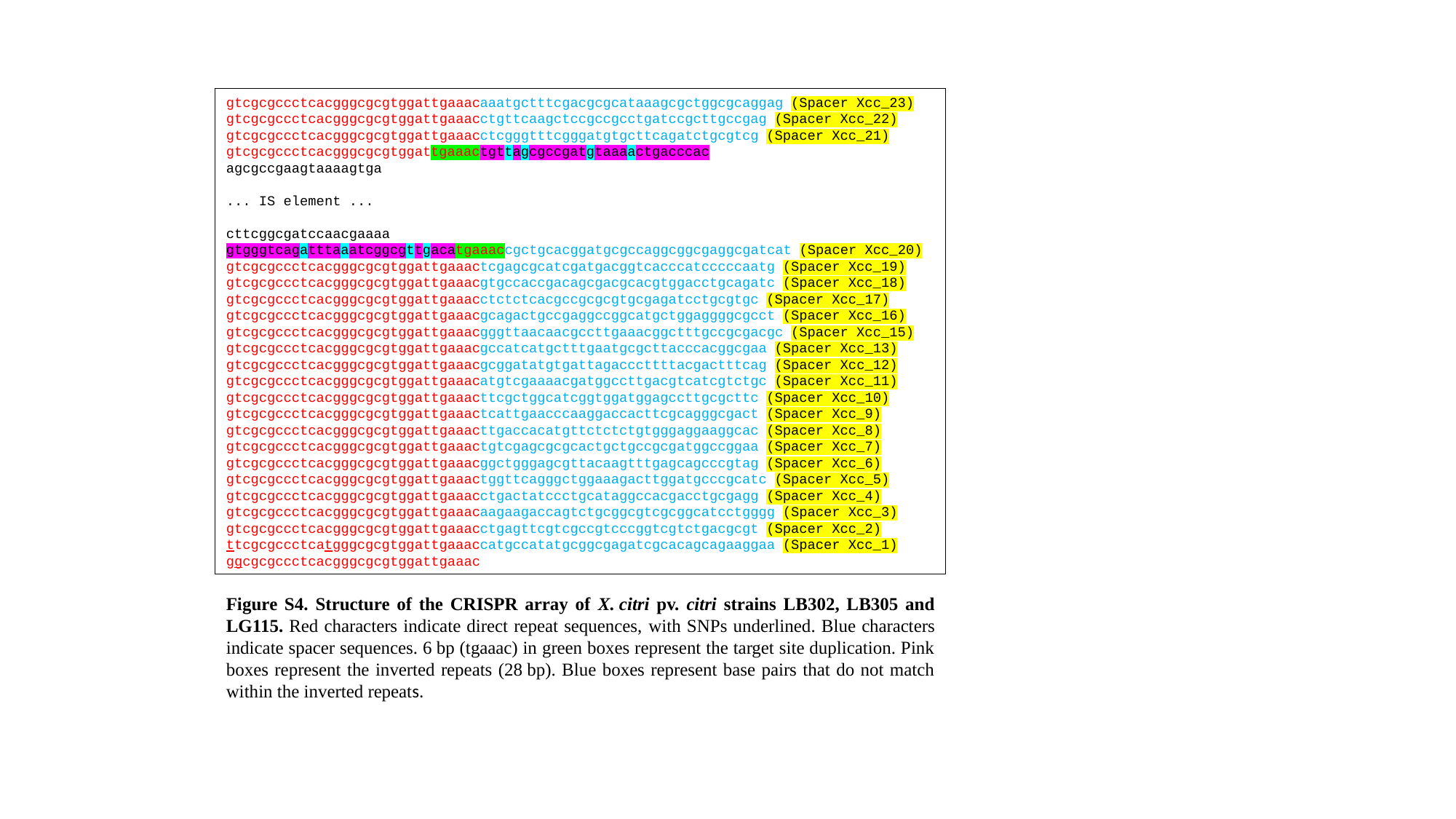

gtcgcgccctcacgggcgcgtggattgaaacaaatgctttcgacgcgcataaagcgctggcgcaggag (Spacer Xcc_23)
gtcgcgccctcacgggcgcgtggattgaaacctgttcaagctccgccgcctgatccgcttgccgag (Spacer Xcc_22)
gtcgcgccctcacgggcgcgtggattgaaacctcgggtttcgggatgtgcttcagatctgcgtcg (Spacer Xcc_21)
gtcgcgccctcacgggcgcgtggattgaaactgttagcgccgatgtaaaactgacccac
agcgccgaagtaaaagtga
... IS element ...
cttcggcgatccaacgaaaa
gtgggtcagatttaaatcggcgttgacatgaaaccgctgcacggatgcgccaggcggcgaggcgatcat (Spacer Xcc_20)
gtcgcgccctcacgggcgcgtggattgaaactcgagcgcatcgatgacggtcacccatcccccaatg (Spacer Xcc_19)
gtcgcgccctcacgggcgcgtggattgaaacgtgccaccgacagcgacgcacgtggacctgcagatc (Spacer Xcc_18)
gtcgcgccctcacgggcgcgtggattgaaacctctctcacgccgcgcgtgcgagatcctgcgtgc (Spacer Xcc_17)
gtcgcgccctcacgggcgcgtggattgaaacgcagactgccgaggccggcatgctggaggggcgcct (Spacer Xcc_16)
gtcgcgccctcacgggcgcgtggattgaaacgggttaacaacgccttgaaacggctttgccgcgacgc (Spacer Xcc_15)
gtcgcgccctcacgggcgcgtggattgaaacgccatcatgctttgaatgcgcttacccacggcgaa (Spacer Xcc_13)
gtcgcgccctcacgggcgcgtggattgaaacgcggatatgtgattagacccttttacgactttcag (Spacer Xcc_12)
gtcgcgccctcacgggcgcgtggattgaaacatgtcgaaaacgatggccttgacgtcatcgtctgc (Spacer Xcc_11)
gtcgcgccctcacgggcgcgtggattgaaacttcgctggcatcggtggatggagccttgcgcttc (Spacer Xcc_10)
gtcgcgccctcacgggcgcgtggattgaaactcattgaacccaaggaccacttcgcagggcgact (Spacer Xcc_9)
gtcgcgccctcacgggcgcgtggattgaaacttgaccacatgttctctctgtgggaggaaggcac (Spacer Xcc_8)
gtcgcgccctcacgggcgcgtggattgaaactgtcgagcgcgcactgctgccgcgatggccggaa (Spacer Xcc_7)
gtcgcgccctcacgggcgcgtggattgaaacggctgggagcgttacaagtttgagcagcccgtag (Spacer Xcc_6)
gtcgcgccctcacgggcgcgtggattgaaactggttcagggctggaaagacttggatgcccgcatc (Spacer Xcc_5)
gtcgcgccctcacgggcgcgtggattgaaacctgactatccctgcataggccacgacctgcgagg (Spacer Xcc_4)
gtcgcgccctcacgggcgcgtggattgaaacaagaagaccagtctgcggcgtcgcggcatcctgggg (Spacer Xcc_3)
gtcgcgccctcacgggcgcgtggattgaaacctgagttcgtcgccgtcccggtcgtctgacgcgt (Spacer Xcc_2)
ttcgcgccctcatgggcgcgtggattgaaaccatgccatatgcggcgagatcgcacagcagaaggaa (Spacer Xcc_1)
ggcgcgccctcacgggcgcgtggattgaaac
Figure S4. Structure of the CRISPR array of X. citri pv. citri strains LB302, LB305 and LG115. Red characters indicate direct repeat sequences, with SNPs underlined. Blue characters indicate spacer sequences. 6 bp (tgaaac) in green boxes represent the target site duplication. Pink boxes represent the inverted repeats (28 bp). Blue boxes represent base pairs that do not match within the inverted repeats.
