# Supplementary material for: CRISPR elements provide a new framework for the genealogy of the citrus canker pathogen Xanthomonas citri pv. citri
Source: BMC Genomics. 2019 Dec 2;20:917. doi: 10.1186/s12864-019-6267-z (PMC6889575; doi:10.1186/s12864-019-6267-z)
Supplement: Supplementary file 5 — Additional file 5: Figure S5. Structure of the CRISPR array of X. citri pv. citri strain LG097. Red characters indicate direct repeat sequences, with SNPs underlined. Blue characters indicate spacer sequences. 6 bp (cctgca) in green boxes represent the target site duplication. Pink boxes represent the inverted repeats (28 bp). Blue boxes represent base pairs that do not match within the inverted repeats. Spacer Xcc_18*: 4 bp, indicated by dashes, are deleted due to the IS element insertion. [file 12864_2019_6267_MOESM5_ESM.pptx]

## Slide 1
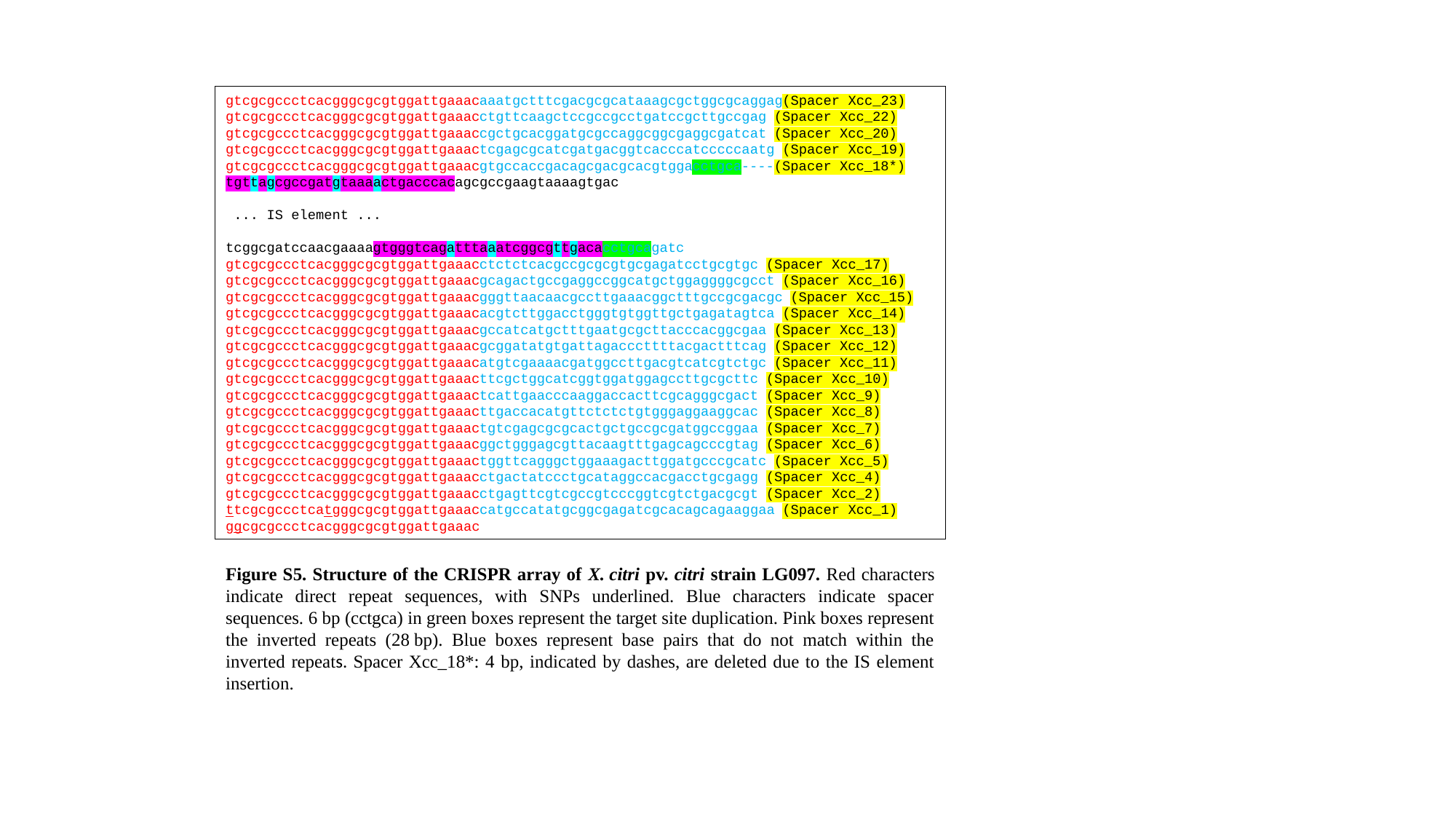

gtcgcgccctcacgggcgcgtggattgaaacaaatgctttcgacgcgcataaagcgctggcgcaggag(Spacer Xcc_23)
gtcgcgccctcacgggcgcgtggattgaaacctgttcaagctccgccgcctgatccgcttgccgag (Spacer Xcc_22)
gtcgcgccctcacgggcgcgtggattgaaaccgctgcacggatgcgccaggcggcgaggcgatcat (Spacer Xcc_20)
gtcgcgccctcacgggcgcgtggattgaaactcgagcgcatcgatgacggtcacccatcccccaatg (Spacer Xcc_19)
gtcgcgccctcacgggcgcgtggattgaaacgtgccaccgacagcgacgcacgtggacctgca----(Spacer Xcc_18*)
tgttagcgccgatgtaaaactgacccacagcgccgaagtaaaagtgac
 ... IS element ...
tcggcgatccaacgaaaagtgggtcagatttaaatcggcgttgacacctgcagatc
gtcgcgccctcacgggcgcgtggattgaaacctctctcacgccgcgcgtgcgagatcctgcgtgc (Spacer Xcc_17)
gtcgcgccctcacgggcgcgtggattgaaacgcagactgccgaggccggcatgctggaggggcgcct (Spacer Xcc_16)
gtcgcgccctcacgggcgcgtggattgaaacgggttaacaacgccttgaaacggctttgccgcgacgc (Spacer Xcc_15)
gtcgcgccctcacgggcgcgtggattgaaacacgtcttggacctgggtgtggttgctgagatagtca (Spacer Xcc_14)
gtcgcgccctcacgggcgcgtggattgaaacgccatcatgctttgaatgcgcttacccacggcgaa (Spacer Xcc_13)
gtcgcgccctcacgggcgcgtggattgaaacgcggatatgtgattagacccttttacgactttcag (Spacer Xcc_12)
gtcgcgccctcacgggcgcgtggattgaaacatgtcgaaaacgatggccttgacgtcatcgtctgc (Spacer Xcc_11)
gtcgcgccctcacgggcgcgtggattgaaacttcgctggcatcggtggatggagccttgcgcttc (Spacer Xcc_10)
gtcgcgccctcacgggcgcgtggattgaaactcattgaacccaaggaccacttcgcagggcgact (Spacer Xcc_9)
gtcgcgccctcacgggcgcgtggattgaaacttgaccacatgttctctctgtgggaggaaggcac (Spacer Xcc_8)
gtcgcgccctcacgggcgcgtggattgaaactgtcgagcgcgcactgctgccgcgatggccggaa (Spacer Xcc_7)
gtcgcgccctcacgggcgcgtggattgaaacggctgggagcgttacaagtttgagcagcccgtag (Spacer Xcc_6)
gtcgcgccctcacgggcgcgtggattgaaactggttcagggctggaaagacttggatgcccgcatc (Spacer Xcc_5)
gtcgcgccctcacgggcgcgtggattgaaacctgactatccctgcataggccacgacctgcgagg (Spacer Xcc_4)
gtcgcgccctcacgggcgcgtggattgaaacctgagttcgtcgccgtcccggtcgtctgacgcgt (Spacer Xcc_2)
ttcgcgccctcatgggcgcgtggattgaaaccatgccatatgcggcgagatcgcacagcagaaggaa (Spacer Xcc_1)
ggcgcgccctcacgggcgcgtggattgaaac
Figure S5. Structure of the CRISPR array of X. citri pv. citri strain LG097. Red characters indicate direct repeat sequences, with SNPs underlined. Blue characters indicate spacer sequences. 6 bp (cctgca) in green boxes represent the target site duplication. Pink boxes represent the inverted repeats (28 bp). Blue boxes represent base pairs that do not match within the inverted repeats. Spacer Xcc_18*: 4 bp, indicated by dashes, are deleted due to the IS element insertion.
