# Supplementary material for: CRISPR elements provide a new framework for the genealogy of the citrus canker pathogen Xanthomonas citri pv. citri
Source: BMC Genomics. 2019 Dec 2;20:917. doi: 10.1186/s12864-019-6267-z (PMC6889575; doi:10.1186/s12864-019-6267-z)
Supplement: Supplementary file 6 — Additional file 6: Figure S6. PCR amplification of spacer/repeat units next to the IS element of five X. citri pv. citri strains. M, molecular weight marker (1-kb ladder, Promega); n, negative control (PCR reaction without template DNA). Lanes 2–7, primer combination Leader_fw and IS-1_rev; lanes 9–14, primer combination IS-2_fw and Spacer#18_rev. [file 12864_2019_6267_MOESM6_ESM.pptx]

## Slide 1
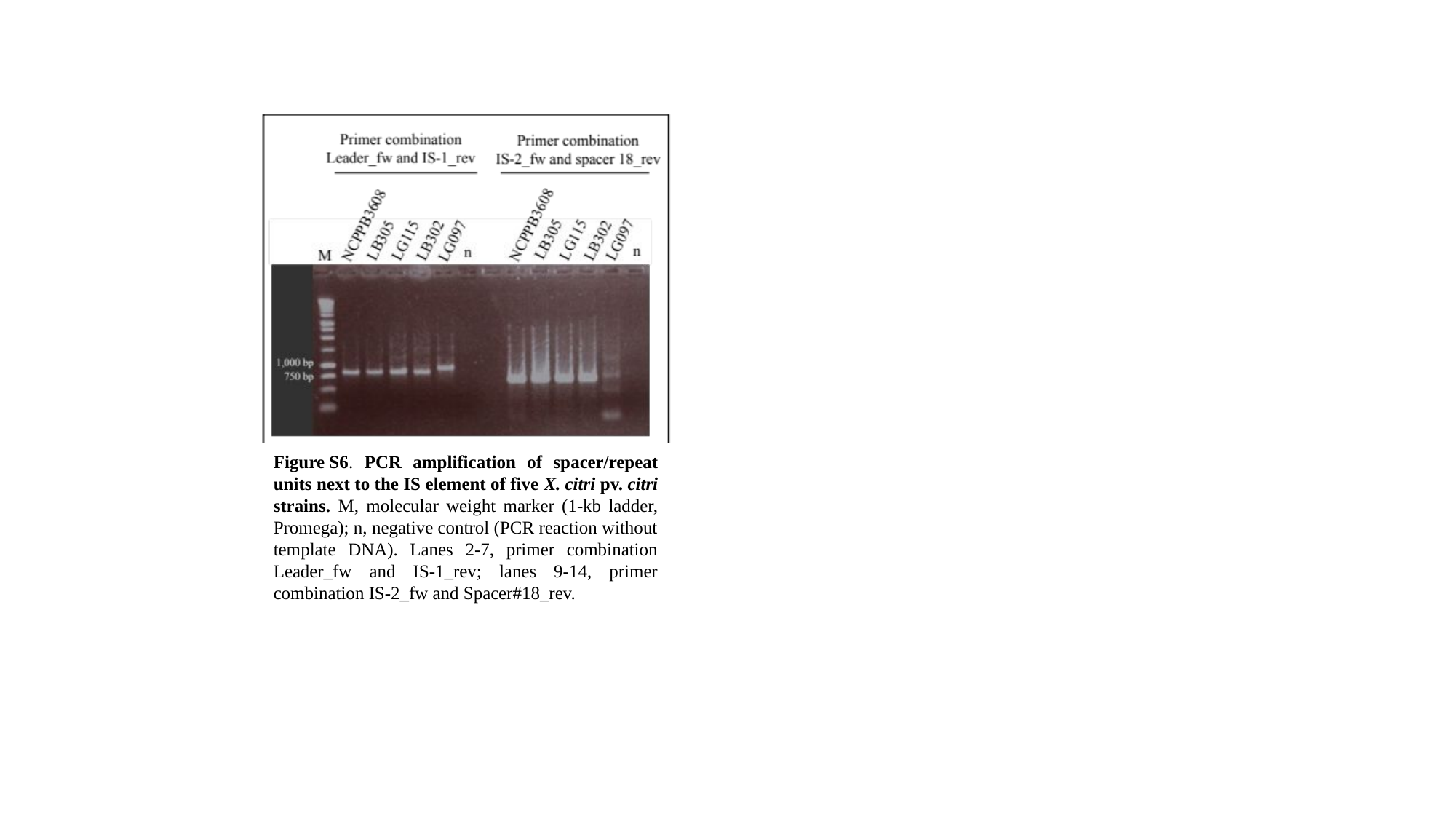

Figure S6. PCR amplification of spacer/repeat units next to the IS element of five X. citri pv. citri strains. M, molecular weight marker (1-kb ladder, Promega); n, negative control (PCR reaction without template DNA). Lanes 2-7, primer combination Leader_fw and IS-1_rev; lanes 9-14, primer combination IS-2_fw and Spacer#18_rev.
