# Supplementary material for: CRISPR elements provide a new framework for the genealogy of the citrus canker pathogen Xanthomonas citri pv. citri
Source: BMC Genomics. 2019 Dec 2;20:917. doi: 10.1186/s12864-019-6267-z (PMC6889575; doi:10.1186/s12864-019-6267-z)
Supplement: Supplementary file 7 — Additional file 7: Table S1. BLASTN database searches for spacer-related sequences at NCBI GenBank. [file 12864_2019_6267_MOESM7_ESM.pdf]

Table S1. BLASTN database searches for spacer-related sequences at NCBI GenBank.

Spacer	Annotation	Acc. No.	E-value	Identity (%)
Xcc_37	*Xanthomonas* phage CP1 DNA, complete genome	AB720063	0.40	27/30 (90%)
Xcc_36	*Xanthomonas* phage CP1 DNA, complete genome	AB720063	5e-06	33/34 (97%)
	Bradymonas sediminis strain FA350 chromosome, complete genome	CP030032	0.40	25/27 (93%)
Xcc_35	*Xanthomonas* phage phi Xc10, complete genome	MF375456	1e-06	34/35 (97%)
	Xanthomonas phage f30-Xaj, complete genome	KU595433	2e-05	33/35 (94%)
	Xanthomonas phage f20-Xaj, complete genome	KU595432	2e-05	33/35 (94%)
	Xylella phage Prado, complete genome	KF626667	8e-04	32/35 (91%)
	Xanthomonas phage XAJ24, complete genome	KU197013	0.009	31/35 (89%)
	blood disease bacterium R229, genomic contig 00006-1627	FR854082	0.40	30/35 (86%)
Xcc_34	No significant hit			
Xcc_33	*Xanthomonas* phage phi Xc10, complete genome	MF375456	0.003	31/34 (91%)
Xcc_32	No significant hit			
Xcc_31	*Xanthomonas* phage f30-Xaj, complete genome	KU595433	3e-08	35/35 (100%)
	Xanthomonas phage f20-Xaj, complete genome	KU595432	3e-08	35/35 (100%)
	Xanthomonas phage phi Xc10, complete genome	MF375456	1e-06	34/35 (97%)
	Xylella phage Prado, complete genome	KF626667	2e-05	33/35 (94%)
	Xanthomonas phage XAJ24, complete genome	KU197013	8e-04	32/35 (91%)
	blood disease bacterium R229, genomic contig 00006-1627	FR854082	0.003	31/34 (91%)
	Ralstonia solanacearum genome assembly 9 genomes, chromosome : I	LN899819	0.003	29/31 (94%)
	Xylella phage Paz, complete genome	KF626666	0.009	28/30 (93%)
	Burkholderia phage JG068, complete genome	KC853746	0.033	26/27 (96%)
	Actinopolymorpha singaporensis strain DSM 22024 genome assembly, chromosome: I	LT629732	0.11	25/26 (96%)
	Streptomyces dengpaensis strain XZHG99 chromosome, complete genome	CP026652	0.40	24/25 (96%)
	Xylella taiwanensis strain PLS235 chromosome, complete genome	CP011800	0.40	22/22 (100%)
	Ralstonia phage RSJ5 DNA, complete genome	AB983711	0.40	25/27 (93%)
	Ralstonia phage RSJ2 DNA, complete genome	AB920995	0.40	25/27 (93%)
Xcc_30	*Xanthomonas* phage phi Xc10, complete genome	MF375456	0.003	31/34 (91%)
	Xanthomonas phage f20-Xaj, complete genome	KU595432	0.003	31/34 (91%)
	Actinomadura fulva subsp. *indica* genomic DNA, fluvirucin biosynthetic genes, complete sequence, strain: ATCC 53714	LC095592	0.11	28/31 (90%)
	Pseudomonas sp. MRSN12121, complete genome	CP010892	0.40	28/32 (88%)
	Pseudogulbenkiania sp. NH8B DNA, complete genome	AP012224	0.40	28/32 (88%)
	Cyclobacterium marinum DSM 745, complete genome	CP002955	0.40	25/27 (93%)
Xcc_29	No significant hit			
Xcc_28	*Xanthomonas* phage CP1 DNA, complete genome	AB720063	1e-07	34/34 (100%)
	Xanthomonas phage phi Xc10, complete genome	MF375456	1e-07	34/34 (100%)
	Xanthomonas phage XAJ24, complete genome	KU197013	6e-05	32/34 (94%)
	Xanthomonas phage f30-Xaj, complete genome	KU595433	0.003	31/34 (91%)
	Xanthomonas phage f20-Xaj, complete genome	KU595432	0.003	31/34 (91%)

Spacer	Annotation	Acc. No.	E-value	Identity (%)
	Caulobacter phage Percy, complete genome	KT381879	0.11	23/23 (100%)
	Xylella phage Prado, complete genome	KF626667	0.11	23/23 (100%)
	Ralstonia solanacearum genome assembly 9 genomes, chromosome : I	LN899819	0.40	28/32 (88%)
Xcc_27	*Xanthomonas* phage XAJ24, complete genome	KU197013	2e-04	31/33 (94%)
	Xanthomonas phage f30-Xaj, complete genome	KU595433	0.40	28/32 (88%)
Xcc_26	No significant hit			
Xcc_25	*Xanthomonas* phage CP1 DNA, complete genome	AB720063	1e-06	32/32 (100%)
Xcc_24	No significant hit			
Xcc_23	Perfectly matched to 31 *Xcc* genomes		3e-09	37/37 (100%)
Xcc_22	Perfectly matched to 32 *Xcc* genomes		3e-08	35/35 (100%)
	Xanthomonas citri pv. *vignicola* strain CFBP 7113, complete genome	CP022270	1e-07	34/34 (100%)
	Deinococcus sp. S14-83 strain S14-83T plasmid unnamed2, complete sequence	CP034186	0.40	27/29 (93%)
	Sphingopyxis sp. QXT-31, complete genome	CP019449	0.40	25/27 (93%)
Xcc_21	Perfectly matched to 28 *Xcc* genomes		1e-07	34/34 (100%)
Xcc_20	Perfectly matched to 31 *Xcc* genomes		3e-08	35/35 (100%)
	*Xanthomonas cit*ri pv. *vignicola* strain CFBP 7113, complete genome	CP022270	0.009	31/35 (89%)
	Xanthomonas citri pv. *fuscans* CFBP 6988 chromosome	CP026331	0.40	30/35 (86%)
	Xanthomonas citri pv. *phaseoli* var. *fuscans* strain CFBP 6991 chromosome, complete genome	CP021015	0.40	30/35 (86%)
	Xanthomonas citri pv. *phaseoli* var. *fuscans* strain CFBP 6990 chromosome, complete genome	CP020983	0.40	30/35 (86%)
	Xanthomonas citri pv. *phaseoli* var. *fuscans* strain CFBP 6989 chromosome, complete genome	CP020981	0.40	30/35 (86%)
	Xanthomonas citri pv. *phaseoli* var. *fuscans* strain CFBP 6988R chromosome, complete genome	CP020979	0.40	30/35 (86%)
	Bradyrhizobium sp. CCBAU 51670 chromosome, complete genome	CP030053	0.40	22/22 (100%)
	Bradyrhizobium sp. ORS 3257 isolate ORS3257 genome assembly, chromosome: BRAD3257	LS398110	0.40	22/22 (100%)
	Bradyrhizobium sp. 3 85S1MB chromosome, complete genome	CP029427	0.40	22/22 (100%)
Xcc_19	Perfectly matched to 30 *Xcc* genomes		1e-08	36/36 (100%)
	Xanthomonas citri subsp. *citri* strain jx4, complete genome	CP009013	0.003	28/29 (97%)
	Streptomyces sporoclivatus NBRC 100767 DNA, complete genome	AP019620	0.40	28/32 (88%)
	Enterobacter cancerogenus strain CR-Eb1 chromosome, complete genome	CP025225	0.40	25/27 (93%)
Xcc_18	Perfectly matched to 31 *Xcc* genomes		1e-08	36/36 (100%)
	Pelobacter propionicus DSM 2379, complete genome	CP000482	0.11	30/33 (91%)
	Herbaspirillum rubrisubalbicans strain DSM 11543 chromosome, complete genome	CP024996	0.40	30/34 (88%)
	Herbaspirillum rubrisubalbicans M1, complete genome	CP013737	0.40	30/34 (88%)
Xcc_17	Perfectly matched to 31 *Xcc* genomes		1e-07	34/34 (100%)
	Sphingopyxis sp. PAMC25046 chromosome, complete genome	CP039250	0.40	27/30 (90%)

Spacer	Annotation	Acc. No.	E-value	Identity (%)
	Myxococcus macrosporus DSM 14697 chromosome, complete genome	CP022203	0.40	25/27 (93%)
	Myxococcus macrosporus strain HW-1, complete genome	CP002830	0.40	25/27 (93%)
Xcc_16	Perfectly matched to 31 *Xcc* genomes		1e-08	36/36 (100%)
	Xanthomonas citri pv. *fuscans* CFBP 6988 chromosome	CP026331	6e-05	32/34 (94%)
	Xanthomonas citri pv. *phaseoli* var. *fuscans* strain CFBP 6991 chromosome, complete genome	CP021015	6e-05	32/34 (94%)
	Xanthomonas citri pv. *phaseoli* var. *fuscans* strain CFBP 6990 chromosome, complete genome	CP020983	6e-05	32/34 (94%)
	Xanthomonas citri pv. *phaseoli* var. *fuscans* strain CFBP 6989 chromosome, complete genome	CP020981	6e-05	32/34 (94%)
	Xanthomonas citri pv. *phaseoli* var. *fuscans* strain CFBP 6988R chromosome, complete genome	CP020979	6e-05	32/34 (94%)
	Paracoccus sp. BM15 chromosome, complete genome	CP025408	0.11	25/26 (96%)
	Mesorhizobium sp. M8A.F.Ca.ET.057.01.1.1 chromosome	CP034454	0.11	26/28 (93%)
	Burkholderia thailandensis strain FDAARGOS_242 chromosome 1, complete sequence	CP022217	0.11	26/28 (93%)
	Burkholderia thailandensis strain FDAARGOS_241 chromosome 1, complete sequence	CP022214	0.11	26/28 (93%)
	Burkholderia thailandensis strain FDAARGOS_238 chromosome 1, complete sequence	CP020392	0.11	26/28 (93%)
	Burkholderia thailandensis strain FDAARGOS_237 chromosome 1, complete sequence	CP020390	0.11	26/28 (93%)
	Burkholderia thailandensis strain 2002721643 chromosome 1, complete sequence	CP013411	0.11	26/28 (93%)
	Burkholderia thailandensis strain 2002721121 chromosome 1, complete sequence	CP013409	0.11	26/28 (93%)
	Burkholderia thailandensis strain MSMB59 chromosome 1, complete sequence	CP013407	0.11	26/28 (93%)
	Burkholderia thailandensis strain 2003015869 chromosome 1, complete sequence	CP013360	0.11	26/28 (93%)
	Azospirillum thiophilum strain BV-S chromosome 8, complete sequence	CP012408	0.11	26/28 (93%)
	Streptomyces leeuwenhoekii genome assembly sleC34, chromosome	LN831790	0.11	26/28 (93%)
	Burkholderia thailandensis 34 chromosome 1, complete sequence	CP010017	0.11	26/28 (93%)
	Burkholderia thailandensis 2002721643 chromosome I, complete sequence	CP009601	0.11	26/28 (93%)
	Burkholderia thailandensis strain 2003015869 chromosome 1, complete sequence	CP008914	0.11	26/28 (93%)
	Streptomyces cyaneogriseus subsp. *noncyanogenus* strain NMWT 1, complete genome	CP010849	0.11	26/28 (93%)
	Burkholderia thailandensis E254 chromosome 1, complete sequence	CP004381	0.11	26/28 (93%)
	Burkholderia thailandensis MSMB59 chromosome 1, complete sequence	CP004385	0.11	26/28 (93%)
	Burkholderia thailandensis E264 chromosome 1, complete sequence	CP008785	0.11	26/28 (93%)
	Burkholderia thailandensis USAMRU Malaysia #20 chromosome 1, complete sequence	CP004383	0.11	26/28 (93%)
	Burkholderia thailandensis E444 chromosome 1,	CP004117	0.11	26/28 (93%)

Spacer	Annotation	Acc. No.	E-value	Identity (%)
	complete sequence			
	Burkholderia thailandensis 2002721723 chromosome 1, complete sequence	CP004097	0.11	26/28 (93%)
	Burkholderia thailandensis H0587 chromosome 1, complete sequence	CP004089	0.11	26/28 (93%)
	Burkholderia thailandensis E264 chromosome I, complete sequence	CP000086	0.11	26/28 (93%)
	Thermobispora bispora DSM 43833, complete genome	CP001874	0.40	30/35 (86%)
	Thioalkalivibrio versutus strain D301, complete genome	CP011367	0.40	25/27 (93%)
	Bordetella petrii strain DSM 12804, complete genome	AM902716	0.40	25/27 (93%)
Xcc_15	Perfectly matched to 31 *Xcc* genomes		3e-09	37/37 (100%)
Xcc_14	Perfectly matched to two *Xcc* genomes		1e-08	36/36 (100%)
Xcc_13	Perfectly matched to 29 *Xcc* genomes		3e-08	35/35 (100%)
	Xanthomonas citri subsp. *citri* strain UI7, complete genome	CP008989	1e-06	34/35 (97%)
	Xanthomonas citri subsp. *citri* UI6, complete genome	CP008992	1e-06	34/35 (97%)
Xcc_12	Perfectly matched to 30 *Xcc* genomes		3e-08	35/35 (100%)
Xcc_11	Perfectly matched to seven *Xcc* genomes		3e-08	35/35 (100%)
	Pseudomonas citronellolis strain SJTE-3, complete genome	CP015878	0.11	26/28 (93%)
	Pseudomonas aeruginosa strain N15-01092, complete sequence	CP012901	0.11	26/28 (93%)
	Pseudomonas aeruginosa strain PSE305, genome	HG974234	0.11	26/28 (93%)
	Achromobacter phage phiAxp-2, complete genome	KT321316	0.40	30/35 (86%)
Xcc_10	Perfectly matched to eight *Xcc* genomes		1e-07	34/34 (100%)
	Herbaspirillum robiniae strain AA6 chromosome, complete genome	CP018845	0.40	27/30 (90%)
	Uncultured Mediterranean phage uvDeep-CGR2-AD3-C191, complete genome	KT997870	0.40	28/32 (88%)
	Ramlibacter tataouinensis strain 5-10, complete genome	CP010951	0.40	22/22 (100%)
Xcc_9	Perfectly matched to 30 *Xcc* genomes		1e-07	34/34 (100%)
Xcc_8	Perfectly matched to eight *Xcc* genomes		1e-07	34/34 (100%)
Xcc_7	Perfectly matched to 33 *Xcc* genomes		1e-07	34/34 (100%)
	Rhodococcus sp. NJ-530 chromosome, complete genome	CP034152	0.11	26/28 (93%)
	Rhodococcus sp. djl-6-2 chromosome, complete genome	CP025959	0.11	26/28 (93%)
	Xanthomonas citri pv. *glycines* str. 12-2 chromosome, complete genome	CP015972	0.11	26/28 (93%)
	Rhodococcus sp. BH4, complete genome	CP014941	0.11	26/28 (93%)
	Rhodococcus sp. YL-1, complete genome	CP017299	0.11	26/28 (93%)
	Rhodococcus sp. 008, complete genome	CP012749	0.11	26/28 (93%)
	Alloactinosynnema sp. L-07 genome assembly *Alloactinosynnema* sp. L-07, chromosome : I	LN850107	0.40	24/25 (96%)
	Pseudomonas putida strain B4 chromosome, complete genome	CP022561	0.40	22/22 (100%)
	Pseudomonas putida strain NCTC13186 genome assembly, chromosome: 1	LR134299	0.40	22/22 (100%)

Spacer	Annotation	Acc. No.	E-value	Identity (%)
	Pseudomonas sp. SWI36 chromosome, complete genome	CP026675	0.40	22/22 (100%)
	Pseudomonas sp. XWY-1 chromosome, complete genome	CP026332	0.40	22/22 (100%)
	Pseudomonas putida strain KT2440 genome assembly, chromosome: I	LT799039	0.40	22/22 (100%)
	Pseudomonas putida strain N1R genome assembly, chromosome: I	LT707061	0.40	22/22 (100%)
	Pseudomonas putida JB, complete genome	CP016212	0.40	22/22 (100%)
	Pseudomonas putida SJTE-1, complete genome	CP015876	0.40	22/22 (100%)
	Pseudomonas putida KT2440 complete genome	AE015451	0.40	22/22 (100%)
	Pseudomonas putida S12, complete genome	CP009974	0.40	22/22 (100%)
	Nocardia nova SH22a, complete genome	CP006850	0.40	25/27 (93%)
	Pseudomonas putida DOT-T1E, complete genome	CP003734	0.40	22/22 (100%)
	Pseudomonas putida ND6, complete genome	CP003588	0.40	22/22 (100%)
	Pseudomonas putida BIRD-1, complete genome	CP002290	0.40	22/22 (100%)
Xcc_6	Perfectly matched to 32 *Xcc* genomes		1e-07	34/34 (100%)
Xcc_5	Perfectly matched to 33 *Xcc* genomes		3e-08	35/35 (100%)
Xcc_4	Perfectly matched to 33 *Xcc* genomes		1e-07	34/34 (100%)
Xcc_3	Perfectly matched to 15 *Xcc* genomes		1e-08	36/36 (100%)
	Synechococcus sp. SynAce01, complete genome	CP018091	0.11	23/23 (100%)
	Xanthomonas vasicola pv. *vasculorum* strain Xv1601 chromosome, complete genome	CP025272	0.40	30/35 (86%)
	Xanthomonas vasicola strain NCPPB 1060 chromosome, complete genome	CP034649	0.40	30/35 (86%)
	Xanthomonas citri pv. vignicola strain CFBP 7113, complete genome	CP022270	0.40	27/30 (90%)
	Xanthomonas citri pv. *phaseoli* var. *fuscans* strain CFBP 6994R chromosome, complete genome	CP020987	0.40	28/32 (88%)
	Rhodopseudomonas palustris BisB18, complete genome	CP000301	0.40	22/22 (100%)
Xcc_2	Perfectly matched to 33 *Xcc* genomes		1e-07	34/34 (100%)
	Halobiforma lacisalsi AJ5, complete genome	CP019285	0.11	26/28 (93%)
	Streptomyces sp. WAC 01529 chromosome, complete genome	CP029617	0.40	22/22 (100%)
	Uncultured *Acidobacteria* bacterium DNA, fosmid clone: JFF010_C03	AP011669	0.40	22/22 (100%)
Xcc_1	Perfectly matched to 33 *Xcc* genomes		1e-08	36/36 (100%)
	Xanthomonas citri pv. *vignicola* strain CFBP 7113, complete genome	CP022270	0.003	32/36 (89%)
	Xanthomonas citri pv. *fuscans* CFBP 6988 chromosome	CP026331	0.11	31/36 (86%)
	Xanthomonas citri pv. *phaseoli* var. *fuscans* strain CFBP 6991 chromosome, complete genome	CP021015	0.11	31/36 (86%)
	Xanthomonas citri pv. *phaseoli* var. *fuscans* strain CFBP 6990 chromosome, complete genome	CP020983	0.11	31/36 (86%)
	Xanthomonas citri pv. *phaseoli* var. *fuscans* strain CFBP 6989 chromosome, complete genome	CP020981	0.11	31/36 (86%)
	Xanthomonas citri pv. *phaseoli* var. *fuscans* strain CFBP 6988R chromosome, complete genome	CP020979	0.11	31/36 (86%)
	Ruminococcus champanellensis type strain 18P13T draft genome	FP929052	0.11	23/23 (100%)

Database at http://www.ncbi.nlm.nih.gov queried on June 12, 2019. Yellow cells indicate identical spacers in completely sequenced strains of *X. citri* pv. *citri*. Orange cells indicate spacers with single-nucleotide polymorphisms in completely sequenced strains of *X. citri* pv. *citri*. Green cells indicate bacteriophage-related sequences. All hits that do not fit the stringent search criteria (E-value < 0.1, at least 90% coverage of the query sequence) with E-values < 1 are given in red. Only hits in in non-eukaryotic organisms are listed.
