# Supplementary material for: CRISPR elements provide a new framework for the genealogy of the citrus canker pathogen Xanthomonas citri pv. citri
Source: BMC Genomics. 2019 Dec 2;20:917. doi: 10.1186/s12864-019-6267-z (PMC6889575; doi:10.1186/s12864-019-6267-z)
Supplement: Supplementary file 8 — Additional file 8: Figure S7. Multiple sequence alignments of the seven cas gene open reading frames, which were retrieved from the corresponding GenBank files (Table 3). [file 12864_2019_6267_MOESM8_ESM.pdf]

Cas3 (effector nuclease-helicase)

```
LG97            ATGGACTTCTTTGCGCATTCGACCAAGCGGCAGGATCGCAGCGATTGGCAACGGCTGGCC
NCPPB3612       ATGGACTTCTTTGCGCATTCGACCAAGCGGCAGGATCGCAGCGATTGGCAACGGCTGGCC
C40             ATGGACTTCTTTGCGCATTCGACCAAGCGGCAGGATCGCAGCGATTGGCAACGGCTGGCC
CFBP2852        ATGGACTTCTTTGCGCATTCGACCAAGCGGCAGGATCGCAGCGATTGGCAACGGCTGGCC
CFBP2911        ATGGACTTCTTTGCGCATTCGACCAAGCGGCAGGATCGCAGCGATTGGCAACGGCTGGCC
FDC217          ATGGACTTCTTTGCGCATTCGACCAAGCGGCAGGATCGCAGCGATTGGCAACGGCTGGCC
FDC1083         ATGGACTTCTTTGCGCATTCGACCAAGCGGCAGGATCGCAGCGATTGGCAACGGCTGGCC
JF90-8          ATGGACTTCTTTGCGCATTCGACCAAGCGGCAGGATCGCAGCGATTGGCAACGGCTGGCC
JJ10-1          ATGGACTTCTTTGCGCATTCGACCAAGCGGCAGGATCGCAGCGATTGGCAACGGCTGGCC
JJ238-10        ATGGACTTCTTTGCGCATTCGACCAAGCGGCAGGATCGCAGCGATTGGCAACGGCTGGCC
JJ238-24        ATGGACTTCTTTGCGCATTCGACCAAGCGGCAGGATCGCAGCGATTGGCAACGGCTGGCC
JK2-10          ATGGACTTCTTTGCGCATTCGACCAAGCGGCAGGATCGCAGCGATTGGCAACGGCTGGCC
JK4-1           ATGGACTTCTTTGCGCATTCGACCAAGCGGCAGGATCGCAGCGATTGGCAACGGCTGGCC
JK143-11        ATGGACTTCTTTGCGCATTCGACCAAGCGGCAGGATCGCAGCGATTGGCAACGGCTGGCC
JS581           ATGGACTTCTTTGCGCATTCGACCAAGCGGCAGGATCGCAGCGATTGGCAACGGCTGGCC
JS584           ATGGACTTCTTTGCGCATTCGACCAAGCGGCAGGATCGCAGCGATTGGCAACGGCTGGCC
JW160-1         ATGGACTTCTTTGCGCATTCGACCAAGCGGCAGGATCGCAGCGATTGGCAACGGCTGGCC
LB100-1         ATGGACTTCTTTGCGCATTCGACCAAGCGGCAGGATCGCAGCGATTGGCAACGGCTGGCC
LC80            ATGGACTTCTTTGCGCATTCGACCAAGCGGCAGGATCGCAGCGATTGGCAACGGCTGGCC
LD71a           ATGGACTTCTTTGCGCATTCGACCAAGCGGCAGGATCGCAGCGATTGGCAACGGCTGGCC
LE20-1          ATGGACTTCTTTGCGCATTCGACCAAGCGGCAGGATCGCAGCGATTGGCAACGGCTGGCC
LG115           ATGGACTTCTTTGCGCATTCGACCAAGCGGCAGGATCGCAGCGATTGGCAACGGCTGGCC
LG117           ATGGACTTCTTTGCGCATTCGACCAAGCGGCAGGATCGCAGCGATTGGCAACGGCTGGCC
LH37-1          ATGGACTTCTTTGCGCATTCGACCAAGCGGCAGGATCGCAGCGATTGGCAACGGCTGGCC
LMG9322         ATGGACTTCTTTGCGCATTCGACCAAGCGGCAGGATCGCAGCGATTGGCAACGGCTGGCC
NCPPB3562       ATGGACTTCTTTGCGCATTCGACCAAGCGGCAGGATCGCAGCGATTGGCAACGGCTGGCC
NCPPB3607       ATGGACTTCTTTGCGCATTCGACCAAGCGGCAGGATCGCAGCGATTGGCAACGGCTGGCC
NCPPB3608       ATGGACTTCTTTGCGCATTCGACCAAGCGGCAGGATCGCAGCGATTGGCAACGGCTGGCC
X2003-3218      ATGGACTTCTTTGCGCATTCGACCAAGCGGCAGGATCGCAGCGATTGGCAACGGCTGGCC
JF90-2          ATGGACTTCTTTGCGCATTCGACCAAGCGGCAGGATCGCAGCGATTGGCAACGGCTGGCC
JM35            ATGGACTTCTTTGCGCATTCGACCAAGCGGCAGGATCGCAGCGATTGGCAACGGCTGGCC
                ************************************************************
```

```
LG97            GATCACCTACTCGCCGTCGGCGAACGCGCCGCCAGGAATGCCGCACCGTTTGGAGGCCAA
NCPPB3612       GATCACCTACTCGCCGTCGGCGAACGCGCCGCCAGGAATGCCGCACCGTTTGGAGGCCAA
C40             GATCACCTACTCGCCGTCGGCGAACGCGCCGCCAGGAATGCCGCACCGTTTGGAGGCCAA
CFBP2852        GATCACCTACTCGCCGTCGGCGAACGCGCCGCCAGGAATGCCGCACCGTTTGGAGGCCAA
CFBP2911        GATCACCTACTCGCCGTCGGCGAACGCGCCGCCAGGAATGCCGCACCGTTTGGAGGCCAA
FDC217          GATCACCTACTCGCCGTCGGCGAACGCGCCGCCAGGAATGCCGCACCGTTTGGAGGCCAA
FDC1083         GATCACCTACTCGCCGTCGGCGAACGCGCCGCCAGGAATGCCGCACCGTTTGGAGGCCAA
JF90-8          GATCACCTACTCGCCGTCGGCGAACGCGCCGCCAGGAATGCCGCACCGTTTGGAGGCCAA
JJ10-1          GATCACCTACTCGCCGTCGGCGAACGCGCCGCCAGGAATGCCGCACCGTTTGGAGGCCAA
JJ238-10        GATCACCTACTCGCCGTCGGCGAACGCGCCGCCAGGAATGCCGCACCGTTTGGAGGCCAA
JJ238-24        GATCACCTACTCGCCGTCGGCGAACGCGCCGCCAGGAATGCCGCACCGTTTGGAGGCCAA
JK2-10          GATCACCTACTCGCCGTCGGCGAACGCGCCGCCAGGAATGCCGCACCGTTTGGAGGCCAA
JK4-1           GATCACCTACTCGCCGTCGGCGAACGCGCCGCCAGGAATGCCGCACCGTTTGGAGGCCAA
JK143-11        GATCACCTACTCGCCGTCGGCGAACGCGCCGCCAGGAATGCCGCACCGTTTGGAGGCCAA
JS581           GATCACCTACTCGCCGTCGGCGAACGCGCCGCCAGGAATGCCGCACCGTTTGGAGGCCAA
JS584           GATCACCTACTCGCCGTCGGCGAACGCGCCGCCAGGAATGCCGCACCGTTTGGAGGCCAA
JW160-1         GATCACCTACTCGCCGTCGGCGAACGCGCCGCCAGGAATGCCGCACCGTTTGGAGGCCAA
LB100-1         GATCACCTACTCGCCGTCGGCGAACGCGCCGCCAGGAATGCCGCACCGTTTGGAGGCCAA
LC80            GATCACCTACTCGCCGTCGGCGAACGCGCCGCCAGGAATGCCGCACCGTTTGGAGGCCAA
LD71a           GATCACCTACTCGCCGTCGGCGAACGCGCCGCCAGGAATGCCGCACCGTTTGGAGGCCAA
LE20-1          GATCACCTACTCGCCGTCGGCGAACGCGCCGCCAGGAATGCCGCACCGTTTGGAGGCCAA
LG115           GATCACCTACTCGCCGTCGGCGAACGCGCCGCCAGGAATGCCGCACCGTTTGGAGGCCAA
LG117           GATCACCTACTCGCCGTCGGCGAACGCGCCGCCAGGAATGCCGCACCGTTTGGAGGCCAA
LH37-1          GATCACCTACTCGCCGTCGGCGAACGCGCCGCCAGGAATGCCGCACCGTTTGGAGGCCAA
LMG9322         GATCACCTACTCGCCGTCGGCGAACGCGCCGCCAGGAATGCCGCACCGTTTGGAGGCCAA
NCPPB3562       GATCACCTACTCGCCGTCGGCGAACGCGCCGCCAGGAATGCCGCACCGTTTGGAGGCCAA
NCPPB3607       GATCACCTACTCGCCGTCGGCGAACGCGCCGCCAGGAATGCCGCACCGTTTGGAGGCCAA
NCPPB3608       GATCACCTACTCGCCGTCGGCGAACGCGCCGCCAGGAATGCCGCACCGTTTGGAGGCCAA
X2003-3218      GATCACCTACTCGCCGTCGGCGAACGCGCCGCCAGGAATGCCGCACCGTTTGGAGGCCAA
JF90-2          GATCACCTACTCGCCGTCGGCGAACGCGCCGCCAGGAATGCCGCACCGTTTGGAGGCCAA
JM35            GATCACCTACTCGCCGTCGGCGAACGCGCCGCCAGGAATGCCGCACCGTTTGGAGGCCAA
                ************************************************************
```

```
LG97            CTTCTGGCCAGTCTGGCCGGACAACTGCACGACCTCGGCAAGTACACACAGAAATTTCAG
NCPPB3612       CTTCTGGCCAGTCTGGCCGGACAACTGCACGACCTCGGCAAGTACACACAGAAATTTCAG
C40             CTTCTGGCCAGTCTGGCCGGACAACTGCACGACCTCGGCAAGTACACACAGAAATTTCAG
CFBP2852        CTTCTGGCCAGTCTGGCCGGACAACTGCACGACCTCGGCAAGTACACACAGAAATTTCAG
CFBP2911        CTTCTGGCCAGTCTGGCCGGACAACTGCACGACCTCGGCAAGTACACACAGAAATTTCAG
FDC217          CTTCTGGCCAGTCTGGCCGGACAACTGCACGACCTCGGCAAGTACACACAGAAATTTCAG
FDC1083         CTTCTGGCCAGTCTGGCCGGACAACTGCACGACCTCGGCAAGTACACACAGAAATTTCAG
JF90-8          CTTCTGGCCAGTCTGGCCGGACAACTGCACGACCTCGGCAAGTACACACAGAAATTTCAG
JJ10-1          CTTCTGGCCAGTCTGGCCGGACAACTGCACGACCTCGGCAAGTACACACAGAAATTTCAG
JJ238-10        CTTCTGGCCAGTCTGGCCGGACAACTGCACGACCTCGGCAAGTACACACAGAAATTTCAG
JJ238-24        CTTCTGGCCAGTCTGGCCGGACAACTGCACGACCTCGGCAAGTACACACAGAAATTTCAG
JK2-10          CTTCTGGCCAGTCTGGCCGGACAACTGCACGACCTCGGCAAGTACACACAGAAATTTCAG
JK4-1           CTTCTGGCCAGTCTGGCCGGACAACTGCACGACCTCGGCAAGTACACACAGAAATTTCAG
JK143-11        CTTCTGGCCAGTCTGGCCGGACAACTGCACGACCTCGGCAAGTACACACAGAAATTTCAG
JS581           CTTCTGGCCAGTCTGGCCGGACAACTGCACGACCTCGGCAAGTACACACAGAAATTTCAG
JS584           CTTCTGGCCAGTCTGGCCGGACAACTGCACGACCTCGGCAAGTACACACAGAAATTTCAG
JW160-1         CTTCTGGCCAGTCTGGCCGGACAACTGCACGACCTCGGCAAGTACACACAGAAATTTCAG
LB100-1         CTTCTGGCCAGTCTGGCCGGACAACTGCACGACCTCGGCAAGTACACACAGAAATTTCAG
LC80            CTTCTGGCCAGTCTGGCCGGACAACTGCACGACCTCGGCAAGTACACACAGAAATTTCAG
LD71a           CTTCTGGCCAGTCTGGCCGGACAACTGCACGACCTCGGCAAGTACACACAGAAATTTCAG
LE20-1          CTTCTGGCCAGTCTGGCCGGACAACTGCACGACCTCGGCAAGTACACACAGAAATTTCAG
LG115           CTTCTGGCCAGTCTGGCCGGACAACTGCACGACCTCGGCAAGTACACACAGAAATTTCAG
LG117           CTTCTGGCCAGTCTGGCCGGACAACTGCACGACCTCGGCAAGTACACACAGAAATTTCAG
LH37-1          CTTCTGGCCAGTCTGGCCGGACAACTGCACGACCTCGGCAAGTACACACAGAAATTTCAG
LMG9322         CTTCTGGCCAGTCTGGCCGGACAACTGCACGACCTCGGCAAGTACACACAGAAATTTCAG
NCPPB3562       CTTCTGGCCAGTCTGGCCGGACAACTGCACGACCTCGGCAAGTACACACAGAAATTTCAG
NCPPB3607       CTTCTGGCCAGTCTGGCCGGACAACTGCACGACCTCGGCAAGTACACACAGAAATTTCAG
NCPPB3608       CTTCTGGCCAGTCTGGCCGGACAACTGCACGACCTCGGCAAGTACACACAGAAATTTCAG
X2003-3218      CTTCTGGCCAGTCTGGCCGGACAACTGCACGACCTCGGCAAGTACACACAGAAATTTCAG
JF90-2          CTTCTGGCCAGTCTGGCCGGACAACTGCACGACCTCGGCAAGTACACACAGAAATTTCAG
JM35            CTTCTGGCCAGTCTGGCCGGACAACTGCACGACCTCGGCAAGTACACACAGAAATTTCAG
                ************************************************************

LG97            GGCCGGCTGGACGGTGACCCGGCCCGTGTCGACCATGCGACCTGGGGCGCTCGGATCGCC
NCPPB3612       GGCCGGCTGGACGGTGACCCGGCCCGTGTCGACCATGCGACCTGGGGCGCTCGGATCGCC
C40             GGCCGGCTGGACGGTGACCCGGCCCGTGTCGACCATGCGACCTGGGGCGCTCGGATCGCC
CFBP2852        GGCCGGCTGGACGGTGACCCGGCCCGTGTCGACCATGCGACCTGGGGCGCTCGGATCGCC
CFBP2911        GGCCGGCTGGACGGTGACCCGGCCCGTGTCGACCATGCGACCTGGGGCGCTCGGATCGCC
FDC217          GGCCGGCTGGACGGTGACCCGGCCCGTGTCGACCATGCGACCTGGGGCGCTCGGATCGCC
FDC1083         GGCCGGCTGGACGGTGACCCGGCCCGTGTCGACCATGCGACCTGGGGCGCTCGGATCGCC
JF90-8          GGCCGGCTGGACGGTGACCCGGCCCGTGTCGACCATGCGACCTGGGGCGCTCGGATCGCC
JJ10-1          GGCCGGCTGGACGGTGACCCGGCCCGTGTCGACCATGCGACCTGGGGCGCTCGGATCGCC
JJ238-10        GGCCGGCTGGACGGTGACCCGGCCCGTGTCGACCATGCGACCTGGGGCGCTCGGATCGCC
JJ238-24        GGCCGGCTGGACGGTGACCCGGCCCGTGTCGACCATGCGACCTGGGGCGCTCGGATCGCC
JK2-10          GGCCGGCTGGACGGTGACCCGGCCCGTGTCGACCATGCGACCTGGGGCGCTCGGATCGCC
JK4-1           GGCCGGCTGGACGGTGACCCGGCCCGTGTCGACCATGCGACCTGGGGCGCTCGGATCGCC
JK143-11        GGCCGGCTGGACGGTGACCCGGCCCGTGTCGACCATGCGACCTGGGGCGCTCGGATCGCC
JS581           GGCCGGCTGGACGGTGACCCGGCCCGTGTCGACCATGCGACCTGGGGCGCTCGGATCGCC
JS584           GGCCGGCTGGACGGTGACCCGGCCCGTGTCGACCATGCGACCTGGGGCGCTCGGATCGCC
JW160-1         GGCCGGCTGGACGGTGACCCGGCCCGTGTCGACCATGCGACCTGGGGCGCTCGGATCGCC
LB100-1         GGCCGGCTGGACGGTGACCCGGCCCGTGTCGACCATGCGACCTGGGGCGCTCGGATCGCC
LC80            GGCCGGCTGGACGGTGACCCGGCCCGTGTCGACCATGCGACCTGGGGCGCTCGGATCGCC
LD71a           GGCCGGCTGGACGGTGACCCGGCCCGTGTCGACCATGCGACCTGGGGCGCTCGGATCGCC
LE20-1          GGCCGGCTGGACGGTGACCCGGCCCGTGTCGACCATGCGACCTGGGGCGCTCGGATCGCC
LG115           GGCCGGCTGGACGGTGACCCGGCCCGTGTCGACCATGCGACCTGGGGCGCTCGGATCGCC
LG117           GGCCGGCTGGACGGTGACCCGGCCCGTGTCGACCATGCGACCTGGGGCGCTCGGATCGCC
LH37-1          GGCCGGCTGGACGGTGACCCGGCCCGTGTCGACCATGCGACCTGGGGCGCTCGGATCGCC
LMG9322         GGCCGGCTGGACGGTGACCCGGCCCGTGTCGACCATGCGACCTGGGGCGCTCGGATCGCC
NCPPB3562       GGCCGGCTGGACGGTGACCCGGCCCGTGTCGACCATGCGACCTGGGGCGCTCGGATCGCC
NCPPB3607       GGCCGGCTGGACGGTGACCCGGCCCGTGTCGACCATGCGACCTGGGGCGCTCGGATCGCC
NCPPB3608       GGCCGGCTGGACGGTGACCCGGCCCGTGTCGACCATGCGACCTGGGGCGCTCGGATCGCC
X2003-3218      GGCCGGCTGGACGGTGACCCGGCCCGTGTCGACCATGCGACCTGGGGCGCTCGGATCGCC
JF90-2          GGCCGGCTGGACGGTGACCCGGCCCGTGTCGACCATGCGACCTGGGGCGCTCGGATCGCC
JM35            GGCCGGCTGGACGGTGACCCGGCCCGTGTCGACCATGCGACCTGGGGCGCTCGGATCGCC
                ************************************************************
```

```
LG97            TGCGAGCGCTATGGTACCGCCGGCACCTTGCTCGCGTACGGCATCGCCGGCCATCACGCC
NCPPB3612       TGCGAGCGCTATGGTACCGCCGGCACCTTGCTCGCGTACGGCATCGCCGGCCATCACGCC
C40             TGCGAGCGCTATGGTACCGCCGGCACCTTGCTCGCGTACGGCATCGCCGGCCATCACGCC
CFBP2852        TGCGAGCGCTATGGTACCGCCGGCACCTTGCTCGCGTACGGCATCGCCGGCCATCACGCC
CFBP2911        TGCGAGCGCTATGGTACCGCCGGCACCTTGCTCGCGTACGGCATCGCCGGCCATCACGCC
FDC217          TGCGAGCGCTATGGTACCGCCGGCACCTTGCTCGCGTACGGCATCGCCGGCCATCACGCC
FDC1083         TGCGAGCGCTATGGTACCGCCGGCACCTTGCTCGCGTACGGCATCGCCGGCCATCACGCC
JF90-8          TGCGAGCGCTATGGTACCGCCGGCACCTTGCTCGCGTACGGCATCGCCGGCCATCACGCC
JJ10-1          TGCGAGCGCTATGGTACCGCCGGCACCTTGCTCGCGTACGGCATCGCCGGCCATCACGCC
JJ238-10        TGCGAGCGCTATGGTACCGCCGGCACCTTGCTCGCGTACGGCATCGCCGGCCATCACGCC
JJ238-24        TGCGAGCGCTATGGTACCGCCGGCACCTTGCTCGCGTACGGCATCGCCGGCCATCACGCC
JK2-10          TGCGAGCGCTATGGTACCGCCGGCACCTTGCTCGCGTACGGCATCGCCGGCCATCACGCC
JK4-1           TGCGAGCGCTATGGTACCGCCGGCACCTTGCTCGCGTACGGCATCGCCGGCCATCACGCC
JK143-11        TGCGAGCGCTATGGTACCGCCGGCACCTTGCTCGCGTACGGCATCGCCGGCCATCACGCC
JS581           TGCGAGCGCTATGGTACCGCCGGCACCTTGCTCGCGTACGGCATCGCCGGCCATCACGCC
JS584           TGCGAGCGCTATGGTACCGCCGGCACCTTGCTCGCGTACGGCATCGCCGGCCATCACGCC
JW160-1         TGCGAGCGCTATGGTACCGCCGGCACCTTGCTCGCGTACGGCATCGCCGGCCATCACGCC
LB100-1         TGCGAGCGCTATGGTACCGCCGGCACCTTGCTCGCGTACGGCATCGCCGGCCATCACGCC
LC80            TGCGAGCGCTATGGTACCGCCGGCACCTTGCTCGCGTACGGCATCGCCGGCCATCACGCC
LD71a           TGCGAGCGCTATGGTACCGCCGGCACCTTGCTCGCGTACGGCATCGCCGGCCATCACGCC
LE20-1          TGCGAGCGCTATGGTACCGCCGGCACCTTGCTCGCGTACGGCATCGCCGGCCATCACGCC
LG115           TGCGAGCGCTATGGTACCGCCGGCACCTTGCTCGCGTACGGCATCGCCGGCCATCACGCC
LG117           TGCGAGCGCTATGGTACCGCCGGCACCTTGCTCGCGTACGGCATCGCCGGCCATCACGCC
LH37-1          TGCGAGCGCTATGGTACCGCCGGCACCTTGCTCGCGTACGGCATCGCCGGCCATCACGCC
LMG9322         TGCGAGCGCTATGGTACCGCCGGCACCTTGCTCGCGTACGGCATCGCCGGCCATCACGCC
NCPPB3562       TGCGAGCGCTATGGTACCGCCGGCACCTTGCTCGCGTACGGCATCGCCGGCCATCACGCC
NCPPB3607       TGCGAGCGCTATGGTACCGCCGGCACCTTGCTCGCGTACGGCATCGCCGGCCATCACGCC
NCPPB3608       TGCGAGCGCTATGGTACCGCCGGCACCTTGCTCGCGTACGGCATCGCCGGCCATCACGCC
X2003-3218      TGCGAGCGCTATGGTACCGCCGGCACCTTGCTCGCGTACGGCATCGCCGGCCATCACGCC
JF90-2          TGCGAGCGCTATGGTACCGCCGGCACCTTGCTCGCGTACGGCATCGCCGGCCATCACGCC
JM35            TGCGAGCGCTATGGTACCGCCGGCACCTTGCTCGCGTACGGCATCGCCGGCCATCACGCC
                ************************************************************

LG97            GGCCTAGCCGATGGACGACCAGGACACGCAGCGCAATCACGCCCTTCGCTGCACGAGCGC
NCPPB3612       GGCCTAGCCGATGGACGACCAGGACACGCAGCGCAATCACGCCCTTCGCTGCACGAGCGC
C40             GGCCTAGCCGATGGACGACCAGGACACGCAGCGCAATCACGCCCTTCGCTGCACGAGCGC
CFBP2852        GGCCTAGCCGATGGACGACCAGGACACGCAGCGCAATCACGCCCTTCGCTGCACGAGCGC
CFBP2911        GGCCTAGCCGATGGACGACCAGGACACGCAGCGCAATCACGCCCTTCGCTGCACGAGCGC
FDC217          GGCCTAGCCGATGGACGACCAGGACACGCAGCGCAATCACGCCCTTCGCTGCACGAGCGC
FDC1083         GGCCTAGCCGATGGACGACCAGGACACGCAGCGCAATCACGCCCTTCGCTGCACGAGCGC
JF90-8          GGCCTAGCCGATGGACGACCAGGACACGCAGCGCAATCACGCCCTTCGCTGCACGAGCGC
JJ10-1          GGCCTAGCCGATGGACGACCAGGACACGCAGCGCAATCACGCCCTTCGCTGCACGAGCGC
JJ238-10        GGCCTAGCCGATGGACGACCAGGACACGCAGCGCAATCACGCCCTTCGCTGCACGAGCGC
JJ238-24        GGCCTAGCCGATGGACGACCAGGACACGCAGCGCAATCACGCCCTTCGCTGCACGAGCGC
JK2-10          GGCCTAGCCGATGGACGACCAGGACACGCAGCGCAATCACGCCCTTCGCTGCACGAGCGC
JK4-1           GGCCTAGCCGATGGACGACCAGGACACGCAGCGCAATCACGCCCTTCGCTGCACGAGCGC
JK143-11        GGCCTAGCCGATGGACGACCAGGACACGCAGCGCAATCACGCCCTTCGCTGCACGAGCGC
JS581           GGCCTAGCCGATGGACGACCAGGACACGCAGCGCAATCACGCCCTTCGCTGCACGAGCGC
JS584           GGCCTAGCCGATGGACGACCAGGACACGCAGCGCAATCACGCCCTTCGCTGCACGAGCGC
JW160-1         GGCCTAGCCGATGGACGACCAGGACACGCAGCGCAATCACGCCCTTCGCTGCACGAGCGC
LB100-1         GGCCTAGCCGATGGACGACCAGGACACGCAGCGCAATCACGCCCTTCGCTGCACGAGCGC
LC80            GGCCTAGCCGATGGACGACCAGGACACGCAGCGCAATCACGCCCTTCGCTGCACGAGCGC
LD71a           GGCCTAGCCGATGGACGACCAGGACACGCAGCGCAATCACGCCCTTCGCTGCACGAGCGC
LE20-1          GGCCTAGCCGATGGACGACCAGGACACGCAGCGCAATCACGCCCTTCGCTGCACGAGCGC
LG115           GGCCTAGCCGATGGACGACCAGGACACGCAGCGCAATCACGCCCTTCGCTGCACGAGCGC
LG117           GGCCTAGCCGATGGACGACCAGGACACGCAGCGCAATCACGCCCTTCGCTGCACGAGCGC
LH37-1          GGCCTAGCCGATGGACGACCAGGACACGCAGCGCAATCACGCCCTTCGCTGCACGAGCGC
LMG9322         GGCCTAGCCGATGGACGACCAGGACACGCAGCGCAATCACGCCCTTCGCTGCACGAGCGC
NCPPB3562       GGCCTAGCCGATGGACGACCAGGACACGCAGCGCAATCACGCCCTTCGCTGCACGAGCGC
NCPPB3607       GGCCTAGCCGATGGACGACCAGGACACGCAGCGCAATCACGCCCTTCGCTGCACGAGCGC
NCPPB3608       GGCCTAGCCGATGGACGACCAGGACACGCAGCGCAATCACGCCCTTCGCTGCACGAGCGC
X2003-3218      GGCCTAGCCGATGGACGACCAGGACACGCAGCGCAATCACGCCCTTCGCTGCACGAGCGC
JF90-2          GGCCTAGCCGATGGACGACCAGGACACGCAGCGCAATCACGCCCTTCGCTGCACGAGCGC
JM35            GGCCTAGCCGATGGACGACCAGGACACGCAGCGCAATCACGCCCTTCGCTGCACGAGCGC
                ************************************************************
```

```
LG97            CTATCGGAGGACTACCGCGAACGGGAACTGCCACCGCTGCTGTCGGACTGGGAACGGGAG
NCPPB3612       CTATCGGAGGACTACCGCGAACGGGAACTGCCACCGCTGCTGTCGGACTGGGAACGGGAG
C40             CTATCGGAGGACTACCGCGAACGGGAACTGCCACCGCTGCTGTCGGACTGGGAACGGGAG
CFBP2852        CTATCGGAGGACTACCGCGAACGGGAACTGCCACCGCTGCTGTCGGACTGGGAACGGGAG
CFBP2911        CTATCGGAGGACTACCGCGAACGGGAACTGCCACCGCTGCTGTCGGACTGGGAACGGGAG
FDC217          CTATCGGAGGACTACCGCGAACGGGAACTGCCACCGCTGCTGTCGGACTGGGAACGGGAG
FDC1083         CTATCGGAGGACTACCGCGAACGGGAACTGCCACCGCTGCTGTCGGACTGGGAACGGGAG
JF90-8          CTATCGGAGGACTACCGCGAACGGGAACTGCCACCGCTGCTGTCGGACTGGGAACGGGAG
JJ10-1          CTATCGGAGGACTACCGCGAACGGGAACTGCCACCGCTGCTGTCGGACTGGGAACGGGAG
JJ238-10        CTATCGGAGGACTACCGCGAACGGGAACTGCCACCGCTGCTGTCGGACTGGGAACGGGAG
JJ238-24        CTATCGGAGGACTACCGCGAACGGGAACTGCCACCGCTGCTGTCGGACTGGGAACGGGAG
JK2-10          CTATCGGAGGACTACCGCGAACGGGAACTGCCACCGCTGCTGTCGGACTGGGAACGGGAG
JK4-1           CTATCGGAGGACTACCGCGAACGGGAACTGCCACCGCTGCTGTCGGACTGGGAACGGGAG
JK143-11        CTATCGGAGGACTACCGCGAACGGGAACTGCCACCGCTGCTGTCGGACTGGGAACGGGAG
JS581           CTATCGGAGGACTACCGCGAACGGGAACTGCCACCGCTGCTGTCGGACTGGGAACGGGAG
JS584           CTATCGGAGGACTACCGCGAACGGGAACTGCCACCGCTGCTGTCGGACTGGGAACGGGAG
JW160-1         CTATCGGAGGACTACCGCGAACGGGAACTGCCACCGCTGCTGTCGGACTGGGAACGGGAG
LB100-1         CTATCGGAGGACTACCGCGAACGGGAACTGCCACCGCTGCTGTCGGACTGGGAACGGGAG
LC80            CTATCGGAGGACTACCGCGAACGGGAACTGCCACCGCTGCTGTCGGACTGGGAACGGGAG
LD71a           CTATCGGAGGACTACCGCGAACGGGAACTGCCACCGCTGCTGTCGGACTGGGAACGGGAG
LE20-1          CTATCGGAGGACTACCGCGAACGGGAACTGCCACCGCTGCTGTCGGACTGGGAACGGGAG
LG115           CTATCGGAGGACTACCGCGAACGGGAACTGCCACCGCTGCTGTCGGACTGGGAACGGGAG
LG117           CTATCGGAGGACTACCGCGAACGGGAACTGCCACCGCTGCTGTCGGACTGGGAACGGGAG
LH37-1          CTATCGGAGGACTACCGCGAACGGGAACTGCCACCGCTGCTGTCGGACTGGGAACGGGAG
LMG9322         CTATCGGAGGACTACCGCGAACGGGAACTGCCACCGCTGCTGTCGGACTGGGAACGGGAG
NCPPB3562       CTATCGGAGGACTACCGCGAACGGGAACTGCCACCGCTGCTGTCGGACTGGGAACGGGAG
NCPPB3607       CTATCGGAGGACTACCGCGAACGGGAACTGCCACCGCTGCTGTCGGACTGGGAACGGGAG
NCPPB3608       CTATCGGAGGACTACCGCGAACGGGAACTGCCACCGCTGCTGTCGGACTGGGAACGGGAG
X2003-3218      CTATCGGAGGACTACCGCGAACGGGAACTGCCACCGCTGCTGTCGGACTGGGAACGGGAG
JF90-2          CTATCGGAGGACTACCGCGAACGGGAACTGCCACCGCTGCTGTCGGACTGGGAACGGGAG
JM35            CTATCGGAGGACTACCGCGAACGGGAACTGCCACCGCTGCTGTCGGACTGGGAACGGGAG
                ************************************************************

LG97            TTGTCACTCCCGGCCAAGCTGGCGTTGCCGGACGGCTTTATTGGTCATCCGCGCCAGGAG
NCPPB3612       TTGTCACTCCCGGCCAAGCTGGCGTTGCCGGACGGCTTTATTGGTCATCCGCGCCAGGAG
C40             TTGTCACTCCCGGCCAAGCTGGCGTTGCCGGACGGCTTTATTGGTCATCCGCGCCAGGAG
CFBP2852        TTGTCACTCCCGGCCAAGCTGGCGTTGCCGGACGGCTTTATTGGTCATCCGCGCCAGGAG
CFBP2911        TTGTCACTCCCGGCCAAGCTGGCGTTGCCGGACGGCTTTATTGGTCATCCGCGCCAGGAG
FDC217          TTGTCACTCCCGGCCAAGCTGGCGTTGCCGGACGGCTTTATTGGTCATCCGCGCCAGGAG
FDC1083         TTGTCACTCCCGGCCAAGCTGGCGTTGCCGGACGGCTTTATTGGTCATCCGCGCCAGGAG
JF90-8          TTGTCACTCCCGGCCAAGCTGGCGTTGCCGGACGGCTTTATTGGTCATCCGCGCCAGGAG
JJ10-1          TTGTCACTCCCGGCCAAGCTGGCGTTGCCGGACGGCTTTATTGGTCATCCGCGCCAGGAG
JJ238-10        TTGTCACTCCCGGCCAAGCTGGCGTTGCCGGACGGCTTTATTGGTCATCCGCGCCAGGAG
JJ238-24        TTGTCACTCCCGGCCAAGCTGGCGTTGCCGGACGGCTTTATTGGTCATCCGCGCCAGGAG
JK2-10          TTGTCACTCCCGGCCAAGCTGGCGTTGCCGGACGGCTTTATTGGTCATCCGCGCCAGGAG
JK4-1           TTGTCACTCCCGGCCAAGCTGGCGTTGCCGGACGGCTTTATTGGTCATCCGCGCCAGGAG
JK143-11        TTGTCACTCCCGGCCAAGCTGGCGTTGCCGGACGGCTTTATTGGTCATCCGCGCCAGGAG
JS581           TTGTCACTCCCGGCCAAGCTGGCGTTGCCGGACGGCTTTATTGGTCATCCGCGCCAGGAG
JS584           TTGTCACTCCCGGCCAAGCTGGCGTTGCCGGACGGCTTTATTGGTCATCCGCGCCAGGAG
JW160-1         TTGTCACTCCCGGCCAAGCTGGCGTTGCCGGACGGCTTTATTGGTCATCCGCGCCAGGAG
LB100-1         TTGTCACTCCCGGCCAAGCTGGCGTTGCCGGACGGCTTTATTGGTCATCCGCGCCAGGAG
LC80            TTGTCACTCCCGGCCAAGCTGGCGTTGCCGGACGGCTTTATTGGTCATCCGCGCCAGGAG
LD71a           TTGTCACTCCCGGCCAAGCTGGCGTTGCCGGACGGCTTTATTGGTCATCCGCGCCAGGAG
LE20-1          TTGTCACTCCCGGCCAAGCTGGCGTTGCCGGACGGCTTTATTGGTCATCCGCGCCAGGAG
LG115           TTGTCACTCCCGGCCAAGCTGGCGTTGCCGGACGGCTTTATTGGTCATCCGCGCCAGGAG
LG117           TTGTCACTCCCGGCCAAGCTGGCGTTGCCGGACGGCTTTATTGGTCATCCGCGCCAGGAG
LH37-1          TTGTCACTCCCGGCCAAGCTGGCGTTGCCGGACGGCTTTATTGGTCATCCGCGCCAGGAG
LMG9322         TTGTCACTCCCGGCCAAGCTGGCGTTGCCGGACGGCTTTATTGGTCATCCGCGCCAGGAG
NCPPB3562       TTGTCACTCCCGGCCAAGCTGGCGTTGCCGGACGGCTTTATTGGTCATCCGCGCCAGGAG
NCPPB3607       TTGTCACTCCCGGCCAAGCTGGCGTTGCCGGACGGCTTTATTGGTCATCCGCGCCAGGAG
NCPPB3608       TTGTCACTCCCGGCCAAGCTGGCGTTGCCGGACGGCTTTATTGGTCATCCGCGCCAGGAG
X2003-3218      TTGTCACTCCCGGCCAAGCTGGCGTTGCCGGACGGCTTTATTGGTCATCCGCGCCAGGAG
JF90-2          TTGTCACTCCCGGCCAAGCTGGCGTTGCCGGACGGCTTTATTGGTCATCCGCGCCAGGAG
JM35            TTGTCACTCCCGGCCAAGCTGGCGTTGCCGGACGGCTTTATTGGTCATCCGCGCCAGGAG
                ************************************************************
```

```
LG97            CGTCGCCCTTTTCAGCTCACGGTCCTGACGCGGATGATCTTCTCCTGCCTGGTCGATTCC
NCPPB3612       CGTCGCCCTTTTCAGCTCACGGTCCTGACGCGGATGATCTTCTCCTGCCTGGTCGATTCC
C40             CGTCGCCCTTTTCAGCTCACGGTCCTGACGCGGATGATCTTCTCCTGCCTGGTCGATTCC
CFBP2852        CGTCGCCCTTTTCAGCTCACGGTCCTGACGCGGATGATCTTCTCCTGCCTGGTCGATTCC
CFBP2911        CGTCGCCCTTTTCAGCTCACGGTCCTGACGCGGATGATCTTCTCCTGCCTGGTCGATTCC
FDC217          CGTCGCCCTTTTCAGCTCACGGTCCTGACGCGGATGATCTTCTCCTGCCTGGTCGATTCC
FDC1083         CGTCGCCCTTTTCAGCTCACGGTCCTGACGCGGATGATCTTCTCCTGCCTGGTCGATTCC
JF90-8          CGTCGCCCTTTTCAGCTCACGGTCCTGACGCGGATGATCTTCTCCTGCCTGGTCGATTCC
JJ10-1          CGTCGCCCTTTTCAGCTCACGGTCCTGACGCGGATGATCTTCTCCTGCCTGGTCGATTCC
JJ238-10        CGTCGCCCTTTTCAGCTCACGGTCCTGACGCGGATGATCTTCTCCTGCCTGGTCGATTCC
JJ238-24        CGTCGCCCTTTTCAGCTCACGGTCCTGACGCGGATGATCTTCTCCTGCCTGGTCGATTCC
JK2-10          CGTCGCCCTTTTCAGCTCACGGTCCTGACGCGGATGATCTTCTCCTGCCTGGTCGATTCC
JK4-1           CGTCGCCCTTTTCAGCTCACGGTCCTGACGCGGATGATCTTCTCCTGCCTGGTCGATTCC
JK143-11        CGTCGCCCTTTTCAGCTCACGGTCCTGACGCGGATGATCTTCTCCTGCCTGGTCGATTCC
JS581           CGTCGCCCTTTTCAGCTCACGGTCCTGACGCGGATGATCTTCTCCTGCCTGGTCGATTCC
JS584           CGTCGCCCTTTTCAGCTCACGGTCCTGACGCGGATGATCTTCTCCTGCCTGGTCGATTCC
JW160-1         CGTCGCCCTTTTCAGCTCACGGTCCTGACGCGGATGATCTTCTCCTGCCTGGTCGATTCC
LB100-1         CGTCGCCCTTTTCAGCTCACGGTCCTGACGCGGATGATCTTCTCCTGCCTGGTCGATTCC
LC80            CGTCGCCCTTTTCAGCTCACGGTCCTGACGCGGATGATCTTCTCCTGCCTGGTCGATTCC
LD71a           CGTCGCCCTTTTCAGCTCACGGTCCTGACGCGGATGATCTTCTCCTGCCTGGTCGATTCC
LE20-1          CGTCGCCCTTTTCAGCTCACGGTCCTGACGCGGATGATCTTCTCCTGCCTGGTCGATTCC
LG115           CGTCGCCCTTTTCAGCTCACGGTCCTGACGCGGATGATCTTCTCCTGCCTGGTCGATTCC
LG117           CGTCGCCCTTTTCAGCTCACGGTCCTGACGCGGATGATCTTCTCCTGCCTGGTCGATTCC
LH37-1          CGTCGCCCTTTTCAGCTCACGGTCCTGACGCGGATGATCTTCTCCTGCCTGGTCGATTCC
LMG9322         CGTCGCCCTTTTCAGCTCACGGTCCTGACGCGGATGATCTTCTCCTGCCTGGTCGATTCC
NCPPB3562       CGTCGCCCTTTTCAGCTCACGGTCCTGACGCGGATGATCTTCTCCTGCCTGGTCGATTCC
NCPPB3607       CGTCGCCCTTTTCAGCTCACGGTCCTGACGCGGATGATCTTCTCCTGCCTGGTCGATTCC
NCPPB3608       CGTCGCCCTTTTCAGCTCACGGTCCTGACGCGGATGATCTTCTCCTGCCTGGTCGATTCC
X2003-3218      CGTCGCCCTTTTCAGCTCACGGTCCTGACGCGGATGATCTTCTCCTGCCTGGTCGATTCC
JF90-2          CGTCGCCCTTTTCAGCTCACGGTCCTGACGCGGATGATCTTCTCCTGCCTGGTCGATTCC
JM35            CGTCGCCCTTTTCAGCTCACGGTCCTGACGCGGATGATCTTCTCCTGCCTGGTCGATTCC
                ************************************************************

LG97            GACTTCACCGAGACCGACGATTTCTATCGCCGCCTGGAAGGGCGCCAGTCGCGTGCGGAG
NCPPB3612       GACTTCACCGAGACCGACGATTTCTATCGCCGCCTGGAAGGGCGCCAGTCGCGTGCGGAG
C40             GACTTCACCGAGACCGACGATTTCTATCGCCGCCTGGAAGGGCGCCAGTCGCGTGCGGAG
CFBP2852        GACTTCACCGAGACCGACGATTTCTATCGCCGCCTGGAAGGGCGCCAGTCGCGTGCGGAG
CFBP2911        GACTTCACCGAGACCGACGATTTCTATCGCCGCCTGGAAGGGCGCCAGTCGCGTGCGGAG
FDC217          GACTTCACCGAGACCGACGATTTCTATCGCCGCCTGGAAGGGCGCCAGTCGCGTGCGGAG
FDC1083         GACTTCACCGAGACCGACGATTTCTATCGCCGCCTGGAAGGGCGCCAGTCGCGTGCGGAG
JF90-8          GACTTCACCGAGACCGACGATTTCTATCGCCGCCTGGAAGGGCGCCAGTCGCGTGCGGAG
JJ10-1          GACTTCACCGAGACCGACGATTTCTATCGCCGCCTGGAAGGGCGCCAGTCGCGTGCGGAG
JJ238-10        GACTTCACCGAGACCGACGATTTCTATCGCCGCCTGGAAGGGCGCCAGTCGCGTGCGGAG
JJ238-24        GACTTCACCGAGACCGACGATTTCTATCGCCGCCTGGAAGGGCGCCAGTCGCGTGCGGAG
JK2-10          GACTTCACCGAGACCGACGATTTCTATCGCCGCCTGGAAGGGCGCCAGTCGCGTGCGGAG
JK4-1           GACTTCACCGAGACCGACGATTTCTATCGCCGCCTGGAAGGGCGCCAGTCGCGTGCGGAG
JK143-11        GACTTCACCGAGACCGACGATTTCTATCGCCGCCTGGAAGGGCGCCAGTCGCGTGCGGAG
JS581           GACTTCACCGAGACCGACGATTTCTATCGCCGCCTGGAAGGGCGCCAGTCGCGTGCGGAG
JS584           GACTTCACCGAGACCGACGATTTCTATCGCCGCCTGGAAGGGCGCCAGTCGCGTGCGGAG
JW160-1         GACTTCACCGAGACCGACGATTTCTATCGCCGCCTGGAAGGGCGCCAGTCGCGTGCGGAG
LB100-1         GACTTCACCGAGACCGACGATTTCTATCGCCGCCTGGAAGGGCGCCAGTCGCGTGCGGAG
LC80            GACTTCACCGAGACCGACGATTTCTATCGCCGCCTGGAAGGGCGCCAGTCGCGTGCGGAG
LD71a           GACTTCACCGAGACCGACGATTTCTATCGCCGCCTGGAAGGGCGCCAGTCGCGTGCGGAG
LE20-1          GACTTCACCGAGACCGACGATTTCTATCGCCGCCTGGAAGGGCGCCAGTCGCGTGCGGAG
LG115           GACTTCACCGAGACCGACGATTTCTATCGCCGCCTGGAAGGGCGCCAGTCGCGTGCGGAG
LG117           GACTTCACCGAGACCGACGATTTCTATCGCCGCCTGGAAGGGCGCCAGTCGCGTGCGGAG
LH37-1          GACTTCACCGAGACCGACGATTTCTATCGCCGCCTGGAAGGGCGCCAGTCGCGTGCGGAG
LMG9322         GACTTCACCGAGACCGACGATTTCTATCGCCGCCTGGAAGGGCGCCAGTCGCGTGCGGAG
NCPPB3562       GACTTCACCGAGACCGACGATTTCTATCGCCGCCTGGAAGGGCGCCAGTCGCGTGCGGAG
NCPPB3607       GACTTCACCGAGACCGACGATTTCTATCGCCGCCTGGAAGGGCGCCAGTCGCGTGCGGAG
NCPPB3608       GACTTCACCGAGACCGACGATTTCTATCGCCGCCTGGAAGGGCGCCAGTCGCGTGCGGAG
X2003-3218      GACTTCACCGAGACCGACGATTTCTATCGCCGCCTGGAAGGGCGCCAGTCGCGTGCGGAG
JF90-2          GACTTCACCGAGACCGACGATTTCTATCGCCGCCTGGAAGGGCGCCAGTCGCGTGCGGAG
JM35            GACTTCACCGAGACCGACGATTTCTATCGCCGCCTGGAAGGGCGCCAGTCGCGTGCGGAG
                ************************************************************
```

```
LG97            GACACCGGCACGCGTCCGGCCCTGCCAGAATTACGGCAATGCCTTGATGCGCACCTGTCC
NCPPB3612       GACACCGGCACGCGTCCGGCCCTGCCAGAATTACGGCAATGCCTTGATGCGCACCTGTCC
C40             GACACCGGCACGCGTCCGGCCCTGCCAGAATTACGGCAATGCCTTGATGCGCACCTGTCC
CFBP2852        GACACCGGCACGCGTCCGGCCCTGCCAGAATTACGGCAATGCCTTGATGCGCACCTGTCC
CFBP2911        GACACCGGCACGCGTCCGGCCCTGCCAGAATTACGGCAATGCCTTGATGCGCACCTGTCC
FDC217          GACACCGGCACGCGTCCGGCCCTGCCAGAATTACGGCAATGCCTTGATGCGCACCTGTCC
FDC1083         GACACCGGCACGCGTCCGGCCCTGCCAGAATTACGGCAATGCCTTGATGCGCACCTGTCC
JF90-8          GACACCGGCACGCGTCCGGCCCTGCCAGAATTACGGCAATGCCTTGATGCGCACCTGTCC
JJ10-1          GACACCGGCACGCGTCCGGCCCTGCCAGAATTACGGCAATGCCTTGATGCGCACCTGTCC
JJ238-10        GACACCGGCACGCGTCCGGCCCTGCCAGAATTACGGCAATGCCTTGATGCGCACCTGTCC
JJ238-24        GACACCGGCACGCGTCCGGCCCTGCCAGAATTACGGCAATGCCTTGATGCGCACCTGTCC
JK2-10          GACACCGGCACGCGTCCGGCCCTGCCAGAATTACGGCAATGCCTTGATGCGCACCTGTCC
JK4-1           GACACCGGCACGCGTCCGGCCCTGCCAGAATTACGGCAATGCCTTGATGCGCACCTGTCC
JK143-11        GACACCGGCACGCGTCCGGCCCTGCCAGAATTACGGCAATGCCTTGATGCGCACCTGTCC
JS581           GACACCGGCACGCGTCCGGCCCTGCCAGAATTACGGCAATGCCTTGATGCGCACCTGTCC
JS584           GACACCGGCACGCGTCCGGCCCTGCCAGAATTACGGCAATGCCTTGATGCGCACCTGTCC
JW160-1         GACACCGGCACGCGTCCGGCCCTGCCAGAATTACGGCAATGCCTTGATGCGCACCTGTCC
LB100-1         GACACCGGCACGCGTCCGGCCCTGCCAGAATTACGGCAATGCCTTGATGCGCACCTGTCC
LC80            GACACCGGCACGCGTCCGGCCCTGCCAGAATTACGGCAATGCCTTGATGCGCACCTGTCC
LD71a           GACACCGGCACGCGTCCGGCCCTGCCAGAATTACGGCAATGCCTTGATGCGCACCTGTCC
LE20-1          GACACCGGCACGCGTCCGGCCCTGCCAGAATTACGGCAATGCCTTGATGCGCACCTGTCC
LG115           GACACCGGCACGCGTCCGGCCCTGCCAGAATTACGGCAATGCCTTGATGCGCACCTGTCC
LG117           GACACCGGCACGCGTCCGGCCCTGCCAGAATTACGGCAATGCCTTGATGCGCACCTGTCC
LH37-1          GACACCGGCACGCGTCCGGCCCTGCCAGAATTACGGCAATGCCTTGATGCGCACCTGTCC
LMG9322         GACACCGGCACGCGTCCGGCCCTGCCAGAATTACGGCAATGCCTTGATGCGCACCTGTCC
NCPPB3562       GACACCGGCACGCGTCCGGCCCTGCCAGAATTACGGCAATGCCTTGATGCGCACCTGTCC
NCPPB3607       GACACCGGCACGCGTCCGGCCCTGCCAGAATTACGGCAATGCCTTGATGCGCACCTGTCC
NCPPB3608       GACACCGGCACGCGTCCGGCCCTGCCAGAATTACGGCAATGCCTTGATGCGCACCTGTCC
X2003-3218      GACACCGGCACGCGTCCGGCCCTGCCAGAATTACGGCAATGCCTTGATGCGCACCTGTCC
JF90-2          GACACCGGCACGCGTCCGGCCCTGCCAGAATTACGGCAATGCCTTGATGCGCACCTGTCC
JM35            GACACCGGCACGCGTCCGGCCCTGCCAGAATTACGGCAATGCCTTGATGCGCACCTGTCC
                ************************************************************

LG97            ACGTTGCCCACCGAGGGCGGCATCAACCCCTTGCGCGCGCAGATCCTGAGCGAAGTTCGT
NCPPB3612       ACGTTGCCCACCGAGGGCGGCATCAACCCCTTGCGCGCGCAGATCCTGAGCGAAGTTCGT
C40             ACGTTGCCCACCGAGGGCGGCATCAACCCCTTGCGCGCGCAGATCCTGAGCGAAGTTCGT
CFBP2852        ACGTTGCCCACCGAGGGCGGCATCAACCCCTTGCGCGCGCAGATCCTGAGCGAAGTTCGT
CFBP2911        ACGTTGCCCACCGAGGGCGGCATCAACCCCTTGCGCGCGCAGATCCTGAGCGAAGTTCGT
FDC217          ACGTTGCCCACCGAGGGCGGCATCAACCCCTTGCGCGCGCAGATCCTGAGCGAAGTTCGT
FDC1083         ACGTTGCCCACCGAGGGCGGCATCAACCCCTTGCGCGCGCAGATCCTGAGCGAAGTTCGT
JF90-8          ACGTTGCCCACCGAGGGCGGCATCAACCCCTTGCGCGCGCAGATCCTGAGCGAAGTTCGT
JJ10-1          ACGTTGCCCACCGAGGGCGGCATCAACCCCTTGCGCGCGCAGATCCTGAGCGAAGTTCGT
JJ238-10        ACGTTGCCCACCGAGGGCGGCATCAACCCCTTGCGCGCGCAGATCCTGAGCGAAGTTCGT
JJ238-24        ACGTTGCCCACCGAGGGCGGCATCAACCCCTTGCGCGCGCAGATCCTGAGCGAAGTTCGT
JK2-10          ACGTTGCCCACCGAGGGCGGCATCAACCCCTTGCGCGCGCAGATCCTGAGCGAAGTTCGT
JK4-1           ACGTTGCCCACCGAGGGCGGCATCAACCCCTTGCGCGCGCAGATCCTGAGCGAAGTTCGT
JK143-11        ACGTTGCCCACCGAGGGCGGCATCAACCCCTTGCGCGCGCAGATCCTGAGCGAAGTTCGT
JS581           ACGTTGCCCACCGAGGGCGGCATCAACCCCTTGCGCGCGCAGATCCTGAGCGAAGTTCGT
JS584           ACGTTGCCCACCGAGGGCGGCATCAACCCCTTGCGCGCGCAGATCCTGAGCGAAGTTCGT
JW160-1         ACGTTGCCCACCGAGGGCGGCATCAACCCCTTGCGCGCGCAGATCCTGAGCGAAGTTCGT
LB100-1         ACGTTGCCCACCGAGGGCGGCATCAACCCCTTGCGCGCGCAGATCCTGAGCGAAGTTCGT
LC80            ACGTTGCCCACCGAGGGCGGCATCAACCCCTTGCGCGCGCAGATCCTGAGCGAAGTTCGT
LD71a           ACGTTGCCCACCGAGGGCGGCATCAACCCCTTGCGCGCGCAGATCCTGAGCGAAGTTCGT
LE20-1          ACGTTGCCCACCGAGGGCGGCATCAACCCCTTGCGCGCGCAGATCCTGAGCGAAGTTCGT
LG115           ACGTTGCCCACCGAGGGCGGCATCAACCCCTTGCGCGCGCAGATCCTGAGCGAAGTTCGT
LG117           ACGTTGCCCACCGAGGGCGGCATCAACCCCTTGCGCGCGCAGATCCTGAGCGAAGTTCGT
LH37-1          ACGTTGCCCACCGAGGGCGGCATCAACCCCTTGCGCGCGCAGATCCTGAGCGAAGTTCGT
LMG9322         ACGTTGCCCACCGAGGGCGGCATCAACCCCTTGCGCGCGCAGATCCTGAGCGAAGTTCGT
NCPPB3562       ACGTTGCCCACCGAGGGCGGCATCAACCCCTTGCGCGCGCAGATCCTGAGCGAAGTTCGT
NCPPB3607       ACGTTGCCCACCGAGGGCGGCATCAACCCCTTGCGCGCGCAGATCCTGAGCGAAGTTCGT
NCPPB3608       ACGTTGCCCACCGAGGGCGGCATCAACCCCTTGCGCGCGCAGATCCTGAGCGAAGTTCGT
X2003-3218      ACGTTGCCCACCGAGGGCGGCATCAACCCCTTGCGCGCGCAGATCCTGAGCGAAGTTCGT
JF90-2          ACGTTGCCCACCGAGGGCGGCATCAACCCCTTGCGCGCGCAGATCCTGAGCGAAGTTCGT
JM35            ACGTTGCCCACCGAGGGCGGCATCAACCCCTTGCGCGCGCAGATCCTGAGCGAAGTTCGT
                ************************************************************
```

```
LG97            GCCAAGGCCGGCATGCGTCCTGGTCTGTTCTCCCTCACCGTACCCACCGGCGGCGGCAAG
NCPPB3612       GCCAAGGCCGGCATGCGTCCTGGTCTGTTCTCCCTCACCGTACCCACCGGCGGCGGCAAG
C40             GCCAAGGCCGGCATGCGTCCTGGTCTGTTCTCCCTCACCGTACCCACCGGCGGCGGCAAG
CFBP2852        GCCAAGGCCGGCATGCGTCCTGGTCTGTTCTCCCTCACCGTACCCACCGGCGGCGGCAAG
CFBP2911        GCCAAGGCCGGCATGCGTCCTGGTCTGTTCTCCCTCACCGTACCCACCGGCGGCGGCAAG
FDC217          GCCAAGGCCGGCATGCGTCCTGGTCTGTTCTCCCTCACCGTACCCACCGGCGGCGGCAAG
FDC1083         GCCAAGGCCGGCATGCGTCCTGGTCTGTTCTCCCTCACCGTACCCACCGGCGGCGGCAAG
JF90-8          GCCAAGGCCGGCATGCGTCCTGGTCTGTTCTCCCTCACCGTACCCACCGGCGGCGGCAAG
JJ10-1          GCCAAGGCCGGCATGCGTCCTGGTCTGTTCTCCCTCACCGTACCCACCGGCGGCGGCAAG
JJ238-10        GCCAAGGCCGGCATGCGTCCTGGTCTGTTCTCCCTCACCGTACCCACCGGCGGCGGCAAG
JJ238-24        GCCAAGGCCGGCATGCGTCCTGGTCTGTTCTCCCTCACCGTACCCACCGGCGGCGGCAAG
JK2-10          GCCAAGGCCGGCATGCGTCCTGGTCTGTTCTCCCTCACCGTACCCACCGGCGGCGGCAAG
JK4-1           GCCAAGGCCGGCATGCGTCCTGGTCTGTTCTCCCTCACCGTACCCACCGGCGGCGGCAAG
JK143-11        GCCAAGGCCGGCATGCGTCCTGGTCTGTTCTCCCTCACCGTACCCACCGGCGGCGGCAAG
JS581           GCCAAGGCCGGCATGCGTCCTGGTCTGTTCTCCCTCACCGTACCCACCGGCGGCGGCAAG
JS584           GCCAAGGCCGGCATGCGTCCTGGTCTGTTCTCCCTCACCGTACCCACCGGCGGCGGCAAG
JW160-1         GCCAAGGCCGGCATGCGTCCTGGTCTGTTCTCCCTCACCGTACCCACCGGCGGCGGCAAG
LB100-1         GCCAAGGCCGGCATGCGTCCTGGTCTGTTCTCCCTCACCGTACCCACCGGCGGCGGCAAG
LC80            GCCAAGGCCGGCATGCGTCCTGGTCTGTTCTCCCTCACCGTACCCACCGGCGGCGGCAAG
LD71a           GCCAAGGCCGGCATGCGTCCTGGTCTGTTCTCCCTCACCGTACCCACCGGCGGCGGCAAG
LE20-1          GCCAAGGCCGGCATGCGTCCTGGTCTGTTCTCCCTCACCGTACCCACCGGCGGCGGCAAG
LG115           GCCAAGGCCGGCATGCGTCCTGGTCTGTTCTCCCTCACCGTACCCACCGGCGGCGGCAAG
LG117           GCCAAGGCCGGCATGCGTCCTGGTCTGTTCTCCCTCACCGTACCCACCGGCGGCGGCAAG
LH37-1          GCCAAGGCCGGCATGCGTCCTGGTCTGTTCTCCCTCACCGTACCCACCGGCGGCGGCAAG
LMG9322         GCCAAGGCCGGCATGCGTCCTGGTCTGTTCTCCCTCACCGTACCCACCGGCGGCGGCAAG
NCPPB3562       GCCAAGGCCGGCATGCGTCCTGGTCTGTTCTCCCTCACCGTACCCACCGGCGGCGGCAAG
NCPPB3607       GCCAAGGCCGGCATGCGTCCTGGTCTGTTCTCCCTCACCGTACCCACCGGCGGCGGCAAG
NCPPB3608       GCCAAGGCCGGCATGCGTCCTGGTCTGTTCTCCCTCACCGTACCCACCGGCGGCGGCAAG
X2003-3218      GCCAAGGCCGGCATGCGTCCTGGTCTGTTCTCCCTCACCGTACCCACCGGCGGCGGCAAG
JF90-2          GCCAAGGCCGGCATGCGTCCTGGTCTGTTCTCCCTCACCGTACCCACCGGCGGCGGCAAG
JM35            GCCAAGGCCGGCATGCGTCCTGGTCTGTTCTCCCTCACCGTACCCACCGGCGGCGGCAAG
                ************************************************************

LG97            ACGCTGGCTTCACTGGCATTCGCCCTGGACCATGCCATCGCCCATGGATTGCGCCGGGTG
NCPPB3612       ACGCTGGCTTCACTGGCATTCGCCCTGGACCATGCCATCGCCCATGGATTGCGCCGGGGG
C40             ACGCTGGCTTCACTGGCATTCGCCCTGGACCATGCCATCGCCCATGGATTGCGCCGGGTG
CFBP2852        ACGCTGGCTTCACTGGCATTCGCCCTGGACCATGCCATCGCCCATGGATTGCGCCGGGTG
CFBP2911        ACGCTGGCTTCACTGGCATTCGCCCTGGACCATGCCATCGCCCATGGATTGCGCCGGGTG
FDC217          ACGCTGGCTTCACTGGCATTCGCCCTGGACCATGCCATCGCCCATGGATTGCGCCGGGTG
FDC1083         ACGCTGGCTTCACTGGCATTCGCCCTGGACCATGCCATCGCCCATGGATTGCGCCGGGTG
JF90-8          ACGCTGGCTTCACTGGCATTCGCCCTGGACCATGCCATCGCCCATGGATTGCGCCGGGTG
JJ10-1          ACGCTGGCTTCACTGGCATTCGCCCTGGACCATGCCATCGCCCATGGATTGCGCCGGGTG
JJ238-10        ACGCTGGCTTCACTGGCATTCGCCCTGGACCATGCCATCGCCCATGGATTGCGCCGGGTG
JJ238-24        ACGCTGGCTTCACTGGCATTCGCCCTGGACCATGCCATCGCCCATGGATTGCGCCGGGTG
JK2-10          ACGCTGGCTTCACTGGCATTCGCCCTGGACCATGCCATCGCCCATGGATTGCGCCGGGTG
JK4-1           ACGCTGGCTTCACTGGCATTCGCCCTGGACCATGCCATCGCCCATGGATTGCGCCGGGTG
JK143-11        ACGCTGGCTTCACTGGCATTCGCCCTGGACCATGCCATCGCCCATGGATTGCGCCGGGTG
JS581           ACGCTGGCTTCACTGGCATTCGCCCTGGACCATGCCATCGCCCATGGATTGCGCCGGGTG
JS584           ACGCTGGCTTCACTGGCATTCGCCCTGGACCATGCCATCGCCCATGGATTGCGCCGGGTG
JW160-1         ACGCTGGCTTCACTGGCATTCGCCCTGGACCATGCCATCGCCCATGGATTGCGCCGGGTG
LB100-1         ACGCTGGCTTCACTGGCATTCGCCCTGGACCATGCCATCGCCCATGGATTGCGCCGGGTG
LC80            ACGCTGGCTTCACTGGCATTCGCCCTGGACCATGCCATCGCCCATGGATTGCGCCGGGTG
LD71a           ACGCTGGCTTCACTGGCATTCGCCCTGGACCATGCCATCGCCCATGGATTGCGCCGGGTG
LE20-1          ACGCTGGCTTCACTGGCATTCGCCCTGGACCATGCCATCGCCCATGGATTGCGCCGGGTG
LG115           ACGCTGGCTTCACTGGCATTCGCCCTGGACCATGCCATCGCCCATGGATTGCGCCGGGTG
LG117           ACGCTGGCTTCACTGGCATTCGCCCTGGACCATGCCATCGCCCATGGATTGCGCCGGGTG
LH37-1          ACGCTGGCTTCACTGGCATTCGCCCTGGACCATGCCATCGCCCATGGATTGCGCCGGGTG
LMG9322         ACGCTGGCTTCACTGGCATTCGCCCTGGACCATGCCATCGCCCATGGATTGCGCCGGGTG
NCPPB3562       ACGCTGGCTTCACTGGCATTCGCCCTGGACCATGCCATCGCCCATGGATTGCGCCGGGTG
NCPPB3607       ACGCTGGCTTCACTGGCATTCGCCCTGGACCATGCCATCGCCCATGGATTGCGCCGGGTG
NCPPB3608       ACGCTGGCTTCACTGGCATTCGCCCTGGACCATGCCATCGCCCATGGATTGCGCCGGGTG
X2003-3218      ACGCTGGCTTCACTGGCATTCGCCCTGGACCATGCCATCGCCCATGGATTGCGCCGGGTG
JF90-2          ACGCTGGCTTCACTGGCATTCGCCCTGGACCATGCCATCGCCCATGGATTGCGCCGGGTG
JM35            ACGCTGGCTTCACTGGCATTCGCCCTGGACCATGCCATCGCCCATGGATTGCGCCGGGTG
                ********************************************************** *
```

```
LG97            ATCTACGTGATTCCGTTCACCAGCATCGTCGAACAGACCGTACAGGTGTTTCGGCACGCA
NCPPB3612       ATCTACGTGATTCCGTTCACCAGCATCGTCGAACAGACCGTACAGGTGTTTCGGCACGCA
C40             ATCTACGTGATTCCGTTCACCAGCATCGTCGAACAGACCGTACAGGTGTTTCGGCACGCA
CFBP2852        ATCTACGTGATTCCGTTCACCAGCATCGTCGAACAGACCGTACAGGTGTTTCGGCACGCA
CFBP2911        ATCTACGTGATTCCGTTCACCAGCATCGTCGAACAGACCGTACAGGTGTTTCGGCACGCA
FDC217          ATCTACGTGATTCCGTTCACCAGCATCGTCGAACAGACCGTACAGGTGTTTCGGCACGCA
FDC1083         ATCTACGTGATTCCGTTCACCAGCATCGTCGAACAGACCGTACAGGTGTTTCGGCACGCA
JF90-8          ATCTACGTGATTCCGTTCACCAGCATCGTCGAACAGACCGTACAGGTGTTTCGGCACGCA
JJ10-1          ATCTACGTGATTCCGTTCACCAGCATCGTCGAACAGACCGTACAGGTGTTTCGGCACGCA
JJ238-10        ATCTACGTGATTCCGTTCACCAGCATCGTCGAACAGACCGTACAGGTGTTTCGGCACGCA
JJ238-24        ATCTACGTGATTCCGTTCACCAGCATCGTCGAACAGACCGTACAGGTGTTTCGGCACGCA
JK2-10          ATCTACGTGATTCCGTTCACCAGCATCGTCGAACAGACCGTACAGGTGTTTCGGCACGCA
JK4-1           ATCTACGTGATTCCGTTCACCAGCATCGTCGAACAGACCGTACAGGTGTTTCGGCACGCA
JK143-11        ATCTACGTGATTCCGTTCACCAGCATCGTCGAACAGACCGTACAGGTGTTTCGGCACGCA
JS581           ATCTACGTGATTCCGTTCACCAGCATCGTCGAACAGACCGTACAGGTGTTTCGGCACGCA
JS584           ATCTACGTGATTCCGTTCACCAGCATCGTCGAACAGACCGTACAGGTGTTTCGGCACGCA
JW160-1         ATCTACGTGATTCCGTTCACCAGCATCGTCGAACAGACCGTACAGGTGTTTCGGCACGCA
LB100-1         ATCTACGTGATTCCGTTCACCAGCATCGTCGAACAGACCGTACAGGTGTTTCGGCACGCA
LC80            ATCTACGTGATTCCGTTCACCAGCATCGTCGAACAGACCGTACAGGTGTTTCGGCACGCA
LD71a           ATCTACGTGATTCCGTTCACCAGCATCGTCGAACAGACCGTACAGGTGTTTCGGCACGCA
LE20-1          ATCTACGTGATTCCGTTCACCAGCATCGTCGAACAGACCGTACAGGTGTTTCGGCACGCA
LG115           ATCTACGTGATTCCGTTCACCAGCATCGTCGAACAGACCGTACAGGTGTTTCGGCACGCA
LG117           ATCTACGTGATTCCGTTCACCAGCATCGTCGAACAGACCGTACAGGTGTTTCGGCACGCA
LH37-1          ATCTACGTGATTCCGTTCACCAGCATCGTCGAACAGACCGTACAGGTGTTTCGGCACGCA
LMG9322         ATCTACGTGATTCCGTTCACCAGCATCGTCGAACAGACCGTACAGGTGTTTCGGCACGCA
NCPPB3562       ATCTACGTGATTCCGTTCACCAGCATCGTCGAACAGACCGTACAGGTGTTTCGGCACGCA
NCPPB3607       ATCTACGTGATTCCGTTCACCAGCATCGTCGAACAGACCGTACAGGTGTTTCGGCACGCA
NCPPB3608       ATCTACGTGATTCCGTTCACCAGCATCGTCGAACAGACCGTACAGGTGTTTCGGCACGCA
X2003-3218      ATCTACGTGATTCCGTTCACCAGCATCGTCGAACAGACCGTACAGGTGTTTCGGCACGCA
JF90-2          ATCTACGTGATTCCGTTCACCAGCATCGTCGAACAGACCGTACAGGTGTTTCGGCACGCA
JM35            ATCTACGTGATTCCGTTCACCAGCATCGTCGAACAGACCGTACAGGTGTTTCGGCACGCA
                ************************************************************

LG97            TTGGGCGTGCAGGAGCGCGACGACATCGTGCTGGAGCACCACAGCGCCTTCTTCGACGAT
NCPPB3612       TTGGGCGTGCAGGAGCGCGACGACATCGTGCTGGAGCACCACAGCGCCTTCTTCGACGAT
C40             TTGGGCGTGCAGGAGCGCGACGACATCGTGCTGGAGCACCACAGCGCCTTCTTCGACGAT
CFBP2852        TTGGGCGTGCAGGAGCGCGACGACATCGTGCTGGAGCACCACAGCGCCTTCTTCGACGAT
CFBP2911        TTGGGCGTGCAGGAGCGCGACGACATCGTGCTGGAGCACCACAGCGCCTTCTTCGACGAT
FDC217          TTGGGCGTGCAGGAGCGCGACGACATCGTGCTGGAGCACCACAGCGCCTTCTTCGACGAT
FDC1083         TTGGGCGTGCAGGAGCGCGACGACATCGTGCTGGAGCACCACAGCGCCTTCTTCGACGAT
JF90-8          TTGGGCGTGCAGGAGCGCGACGACATCGTGCTGGAGCACCACAGCGCCTTCTTCGACGAT
JJ10-1          TTGGGCGTGCAGGAGCGCGACGACATCGTGCTGGAGCACCACAGCGCCTTCTTCGACGAT
JJ238-10        TTGGGCGTGCAGGAGCGCGACGACATCGTGCTGGAGCACCACAGCGCCTTCTTCGACGAT
JJ238-24        TTGGGCGTGCAGGAGCGCGACGACATCGTGCTGGAGCACCACAGCGCCTTCTTCGACGAT
JK2-10          TTGGGCGTGCAGGAGCGCGACGACATCGTGCTGGAGCACCACAGCGCCTTCTTCGACGAT
JK4-1           TTGGGCGTGCAGGAGCGCGACGACATCGTGCTGGAGCACCACAGCGCCTTCTTCGACGAT
JK143-11        TTGGGCGTGCAGGAGCGCGACGACATCGTGCTGGAGCACCACAGCGCCTTCTTCGACGAT
JS581           TTGGGCGTGCAGGAGCGCGACGACATCGTGCTGGAGCACCACAGCGCCTTCTTCGACGAT
JS584           TTGGGCGTGCAGGAGCGCGACGACATCGTGCTGGAGCACCACAGCGCCTTCTTCGACGAT
JW160-1         TTGGGCGTGCAGGAGCGCGACGACATCGTGCTGGAGCACCACAGCGCCTTCTTCGACGAT
LB100-1         TTGGGCGTGCAGGAGCGCGACGACATCGTGCTGGAGCACCACAGCGCCTTCTTCGACGAT
LC80            TTGGGCGTGCAGGAGCGCGACGACATCGTGCTGGAGCACCACAGCGCCTTCTTCGACGAT
LD71a           TTGGGCGTGCAGGAGCGCGACGACATCGTGCTGGAGCACCACAGCGCCTTCTTCGACGAT
LE20-1          TTGGGCGTGCAGGAGCGCGACGACATCGTGCTGGAGCACCACAGCGCCTTCTTCGACGAT
LG115           TTGGGCGTGCAGGAGCGCGACGACATCGTGCTGGAGCACCACAGCGCCTTCTTCGACGAT
LG117           TTGGGCGTGCAGGAGCGCGACGACATCGTGCTGGAGCACCACAGCGCCTTCTTCGACGAT
LH37-1          TTGGGCGTGCAGGAGCGCGACGACATCGTGCTGGAGCACCACAGCGCCTTCTTCGACGAT
LMG9322         TTGGGCGTGCAGGAGCGCGACGACATCGTGCTGGAGCACCACAGCGCCTTCTTCGACGAT
NCPPB3562       TTGGGCGTGCAGGAGCGCGACGACATCGTGCTGGAGCACCACAGCGCCTTCTTCGACGAT
NCPPB3607       TTGGGCGTGCAGGAGCGCGACGACATCGTGCTGGAGCACCACAGCGCCTTCTTCGACGAT
NCPPB3608       TTGGGCGTGCAGGAGCGCGACGACATCGTGCTGGAGCACCACAGCGCCTTCTTCGACGAT
X2003-3218      TTGGGCGTGCAGGAGCGCGACGACATCGTGCTGGAGCACCACAGCGCCTTCTTCGACGAT
JF90-2          TTGGGCGTGCAGGAGCGCGACGACATCGTGCTGGAGCACCACAGCGCCTTCTTCGACGAT
JM35            TTGGGCGTGCAGGAGCGCGACGACATCGTGCTGGAGCACCACAGCGCCTTCTTCGACGAT
                ************************************************************
```

```
LG97            CCGACCAAGGCACCGCAATCCATCGACAAGCGCAAATTGGCCATGGAGAACTGGGATGCG
NCPPB3612       CCGACCAAGGCACCGCAATCCATCGACAAGCGCAAATTGGCCATGGAGAACTGGGATGCG
C40             CCGACCAAGGCACCGCAATCCATCGACAAGCGCAAATTGGCCATGGAGAACTGGGATGCG
CFBP2852        CCGACCAAGGCACCGCAATCCATCGACAAGCGCAAATTGGCCATGGAGAACTGGGATGCG
CFBP2911        CCGACCAAGGCACCGCAATCCATCGACAAGCGCAAATTGGCCATGGAGAACTGGGATGCG
FDC217          CCGACCAAGGCACCGCAATCCATCGACAAGCGCAAATTGGCCATGGAGAACTGGGATGCG
FDC1083         CCGACCAAGGCACCGCAATCCATCGACAAGCGCAAATTGGCCATGGAGAACTGGGATGCG
JF90-8          CCGACCAAGGCACCGCAATCCATCGACAAGCGCAAATTGGCCATGGAGAACTGGGATGCG
JJ10-1          CCGACCAAGGCACCGCAATCCATCGACAAGCGCAAATTGGCCATGGAGAACTGGGATGCG
JJ238-10        CCGACCAAGGCACCGCAATCCATCGACAAGCGCAAATTGGCCATGGAGAACTGGGATGCG
JJ238-24        CCGACCAAGGCACCGCAATCCATCGACAAGCGCAAATTGGCCATGGAGAACTGGGATGCG
JK2-10          CCGACCAAGGCACCGCAATCCATCGACAAGCGCAAATTGGCCATGGAGAACTGGGATGCG
JK4-1           CCGACCAAGGCACCGCAATCCATCGACAAGCGCAAATTGGCCATGGAGAACTGGGATGCG
JK143-11        CCGACCAAGGCACCGCAATCCATCGACAAGCGCAAATTGGCCATGGAGAACTGGGATGCG
JS581           CCGACCAAGGCACCGCAATCCATCGACAAGCGCAAATTGGCCATGGAGAACTGGGATGCG
JS584           CCGACCAAGGCACCGCAATCCATCGACAAGCGCAAATTGGCCATGGAGAACTGGGATGCG
JW160-1         CCGACCAAGGCACCGCAATCCATCGACAAGCGCAAATTGGCCATGGAGAACTGGGATGCG
LB100-1         CCGACCAAGGCACCGCAATCCATCGACAAGCGCAAATTGGCCATGGAGAACTGGGATGCG
LC80            CCGACCAAGGCACCGCAATCCATCGACAAGCGCAAATTGGCCATGGAGAACTGGGATGCG
LD71a           CCGACCAAGGCACCGCAATCCATCGACAAGCGCAAATTGGCCATGGAGAACTGGGATGCG
LE20-1          CCGACCAAGGCACCGCAATCCATCGACAAGCGCAAATTGGCCATGGAGAACTGGGATGCG
LG115           CCGACCAAGGCACCGCAATCCATCGACAAGCGCAAATTGGCCATGGAGAACTGGGATGCG
LG117           CCGACCAAGGCACCGCAATCCATCGACAAGCGCAAATTGGCCATGGAGAACTGGGATGCG
LH37-1          CCGACCAAGGCACCGCAATCCATCGACAAGCGCAAATTGGCCATGGAGAACTGGGATGCG
LMG9322         CCGACCAAGGCACCGCAATCCATCGACAAGCGCAAATTGGCCATGGAGAACTGGGATGCG
NCPPB3562       CCGACCAAGGCACCGCAATCCATCGACAAGCGCAAATTGGCCATGGAGAACTGGGATGCG
NCPPB3607       CCGACCAAGGCACCGCAATCCATCGACAAGCGCAAATTGGCCATGGAGAACTGGGATGCG
NCPPB3608       CCGACCAAGGCACCGCAATCCATCGACAAGCGCAAATTGGCCATGGAGAACTGGGATGCG
X2003-3218      CCGACCAAGGCACCGCAATCCATCGACAAGCGCAAATTGGCCATGGAGAACTGGGATGCG
JF90-2          CCGACCAAGGCACCGCAATCCATCGACAAGCGCAAATTGGCCATGGAGAACTGGGATGCG
JM35            CCGACCAAGGCACCGCAATCCATCGACAAGCGCAAATTGGCCATGGAGAACTGGGATGCG
                ************************************************************

LG97            CCGATCGTCGTTACCACCGCAGTGCAGTTCTTCGAGAGCCTGTTCGCCGATCGTCCTTCC
NCPPB3612       CCGATCGTCGTTACCACCGCAGTGCAGTTCTTCGAGAGCCTGTTCGCCGATCGTCCTTCC
C40             CCGATCGTCGTTACCACCGCAGTGCAGTTCTTCGAGAGCCTGTTCGCCGATCGTCCTTCC
CFBP2852        CCGATCGTCGTTACCACCGCAGTGCAGTTCTTCGAGAGCCTGTTCGCCGATCGTCCTTCC
CFBP2911        CCGATCGTCGTTACCACCGCAGTGCAGTTCTTCGAGAGCCTGTTCGCCGATCGTCCTTCC
FDC217          CCGATCGTCGTTACCACCGCAGTGCAGTTCTTCGAGAGCCTGTTCGCCGATCGTCCTTCC
FDC1083         CCGATCGTCGTTACCACCGCAGTGCAGTTCTTCGAGAGCCTGTTCGCCGATCGTCCTTCC
JF90-8          CCGATCGTCGTTACCACCGCAGTGCAGTTCTTCGAGAGCCTGTTCGCCGATCGTCCTTCC
JJ10-1          CCGATCGTCGTTACCACCGCAGTGCAGTTCTTCGAGAGCCTGTTCGCCGATCGTCCTTCC
JJ238-10        CCGATCGTCGTTACCACCGCAGTGCAGTTCTTCGAGAGCCTGTTCGCCGATCGTCCTTCC
JJ238-24        CCGATCGTCGTTACCACCGCAGTGCAGTTCTTCGAGAGCCTGTTCGCCGATCGTCCTTCC
JK2-10          CCGATCGTCGTTACCACCGCAGTGCAGTTCTTCGAGAGCCTGTTCGCCGATCGTCCTTCC
JK4-1           CCGATCGTCGTTACCACCGCAGTGCAGTTCTTCGAGAGCCTGTTCGCCGATCGTCCTTCC
JK143-11        CCGATCGTCGTTACCACCGCAGTGCAGTTCTTCGAGAGCCTGTTCGCCGATCGTCCTTCC
JS581           CCGATCGTCGTTACCACCGCAGTGCAGTTCTTCGAGAGCCTGTTCGCCGATCGTCCTTCC
JS584           CCGATCGTCGTTACCACCGCAGTGCAGTTCTTCGAGAGCCTGTTCGCCGATCGTCCTTCC
JW160-1         CCGATCGTCGTTACCACCGCAGTGCAGTTCTTCGAGAGCCTGTTCGCCGATCGTCCTTCC
LB100-1         CCGATCGTCGTTACCACCGCAGTGCAGTTCTTCGAGAGCCTGTTCGCCGATCGTCCTTCC
LC80            CCGATCGTCGTTACCACCGCAGTGCAGTTCTTCGAGAGCCTGTTCGCCGATCGTCCTTCC
LD71a           CCGATCGTCGTTACCACCGCAGTGCAGTTCTTCGAGAGCCTGTTCGCCGATCGTCCTTCC
LE20-1          CCGATCGTCGTTACCACCGCAGTGCAGTTCTTCGAGAGCCTGTTCGCCGATCGTCCTTCC
LG115           CCGATCGTCGTTACCACCGCAGTGCAGTTCTTCGAGAGCCTGTTCGCCGATCGTCCTTCC
LG117           CCGATCGTCGTTACCACCGCAGTGCAGTTCTTCGAGAGCCTGTTCGCCGATCGTCCTTCC
LH37-1          CCGATCGTCGTTACCACCGCAGTGCAGTTCTTCGAGAGCCTGTTCGCCGATCGTCCTTCC
LMG9322         CCGATCGTCGTTACCACCGCAGTGCAGTTCTTCGAGAGCCTGTTCGCCGATCGTCCTTCC
NCPPB3562       CCGATCGTCGTTACCACCGCAGTGCAGTTCTTCGAGAGCCTGTTCGCCGATCGTCCTTCC
NCPPB3607       CCGATCGTCGTTACCACCGCAGTGCAGTTCTTCGAGAGCCTGTTCGCCGATCGTCCTTCC
NCPPB3608       CCGATCGTCGTTACCACCGCAGTGCAGTTCTTCGAGAGCCTGTTCGCCGATCGTCCTTCC
X2003-3218      CCGATCGTCGTTACCACCGCAGTGCAGTTCTTCGAGAGCCTGTTCGCCGATCGTCCTTCC
JF90-2          CCGATCGTCGTTACCACCGCAGTGCAGTTCTTCGAGAGCCTGTTCGCCGATCGTCCTTCC
JM35            CCGATCGTCGTTACCACCGCAGTGCAGTTCTTCGAGAGCCTGTTCGCCGATCGTCCTTCC
                ************************************************************
```

```
LG97            CGCTGCCGCAAGCTGCACAACATCGCCGGCAGCGTGGTGGTTCTGGACGAAGCGCAGACC
NCPPB3612       CGCTGCCGCAAGCTGCACAACATCGCCGGCAGCGTGGTGGTTCTGGACGAAGCGCAGACC
C40             CGCTGCCGCAAGCTGCACAACATCGCCGGCAGCGTGGTGGTTCTGGACGAAGCGCAGACC
CFBP2852        CGCTGCCGCAAGCTGCACAACATCGCCGGCAGCGTGGTGGTTCTGGACGAAGCGCAGACC
CFBP2911        CGCTGCCGCAAGCTGCACAACATCGCCGGCAGCGTGGTGGTTCTGGACGAAGCGCAGACC
FDC217          CGCTGCCGCAAGCTGCACAACATCGCCGGCAGCGTGGTGGTTCTGGACGAAGCGCAGACC
FDC1083         CGCTGCCGCAAGCTGCACAACATCGCCGGCAGCGTGGTGGTTCTGGACGAAGCGCAGACC
JF90-8          CGCTGCCGCAAGCTGCACAACATCGCCGGCAGCGTGGTGGTTCTGGACGAAGCGCAGACC
JJ10-1          CGCTGCCGCAAGCTGCACAACATCGCCGGCAGCGTGGTGGTTCTGGACGAAGCGCAGACC
JJ238-10        CGCTGCCGCAAGCTGCACAACATCGCCGGCAGCGTGGTGGTTCTGGACGAAGCGCAGACC
JJ238-24        CGCTGCCGCAAGCTGCACAACATCGCCGGCAGCGTGGTGGTTCTGGACGAAGCGCAGACC
JK2-10          CGCTGCCGCAAGCTGCACAACATCGCCGGCAGCGTGGTGGTTCTGGACGAAGCGCAGACC
JK4-1           CGCTGCCGCAAGCTGCACAACATCGCCGGCAGCGTGGTGGTTCTGGACGAAGCGCAGACC
JK143-11        CGCTGCCGCAAGCTGCACAACATCGCCGGCAGCGTGGTGGTTCTGGACGAAGCGCAGACC
JS581           CGCTGCCGCAAGCTGCACAACATCGCCGGCAGCGTGGTGGTTCTGGACGAAGCGCAGACC
JS584           CGCTGCCGCAAGCTGCACAACATCGCCGGCAGCGTGGTGGTTCTGGACGAAGCGCAGACC
JW160-1         CGCTGCCGCAAGCTGCACAACATCGCCGGCAGCGTGGTGGTTCTGGACGAAGCGCAGACC
LB100-1         CGCTGCCGCAAGCTGCACAACATCGCCGGCAGCGTGGTGGTTCTGGACGAAGCGCAGACC
LC80            CGCTGCCGCAAGCTGCACAACATCGCCGGCAGCGTGGTGGTTCTGGACGAAGCGCAGACC
LD71a           CGCTGCCGCAAGCTGCACAACATCGCCGGCAGCGTGGTGGTTCTGGACGAAGCGCAGACC
LE20-1          CGCTGCCGCAAGCTGCACAACATCGCCGGCAGCGTGGTGGTTCTGGACGAAGCGCAGACC
LG115           CGCTGCCGCAAGCTGCACAACATCGCCGGCAGCGTGGTGGTTCTGGACGAAGCGCAGACC
LG117           CGCTGCCGCAAGCTGCACAACATCGCCGGCAGCGTGGTGGTTCTGGACGAAGCGCAGACC
LH37-1          CGCTGCCGCAAGCTGCACAACATCGCCGGCAGCGTGGTGGTTCTGGACGAAGCGCAGACC
LMG9322         CGCTGCCGCAAGCTGCACAACATCGCCGGCAGCGTGGTGGTTCTGGACGAAGCGCAGACC
NCPPB3562       CGCTGCCGCAAGCTGCACAACATCGCCGGCAGCGTGGTGGTTCTGGACGAAGCGCAGACC
NCPPB3607       CGCTGCCGCAAGCTGCACAACATCGCCGGCAGCGTGGTGGTTCTGGACGAAGCGCAGACC
NCPPB3608       CGCTGCCGCAAGCTGCACAACATCGCCGGCAGCGTGGTGGTTCTGGACGAAGCGCAGACC
X2003-3218      CGCTGCCGCAAGCTGCACAACATCGCCGGCAGCGTGGTGGTTCTGGACGAAGCGCAGACC
JF90-2          CGCTGCCGCAAGCTGCACAACATCGCCGGCAGCGTGGTGGTTCTGGACGAAGCGCAGACC
JM35            CGCTGCCGCAAGCTGCACAACATCGCCGGCAGCGTGGTGGTTCTGGACGAAGCGCAGACC
                ************************************************************

LG97            CTGCCGCACGCATTGCTGCGCCCGTGCGTGGCGCTACTGGACGAACTGGCGCGCAACTAC
NCPPB3612       CTGCCGCACGCATTGCTGCGCCCGTGCGTGGCGCTACTGGACGAACTGGCGCGCAACTAC
C40             CTGCCGCACGCATTGCTGCGCCCGTGCGTGGCGCTACTGGACGAACTGGCGCGCAACTAC
CFBP2852        CTGCCGCACGCATTGCTGCGCCCGTGCGTGGCGCTACTGGACGAACTGGCGCGCAACTAC
CFBP2911        CTGCCGCACGCATTGCTGCGCCCGTGCGTGGCGCTACTGGACGAACTGGCGCGCAACTAC
FDC217          CTGCCGCACGCATTGCTGCGCCCGTGCGTGGCGCTACTGGACGAACTGGCGCGCAACTAC
FDC1083         CTGCCGCACGCATTGCTGCGCCCGTGCGTGGCGCTACTGGACGAACTGGCGCGCAACTAC
JF90-8          CTGCCGCACGCATTGCTGCGCCCGTGCGTGGCGCTACTGGACGAACTGGCGCGCAACTAC
JJ10-1          CTGCCGCACGCATTGCTGCGCCCGTGCGTGGCGCTACTGGACGAACTGGCGCGCAACTAC
JJ238-10        CTGCCGCACGCATTGCTGCGCCCGTGCGTGGCGCTACTGGACGAACTGGCGCGCAACTAC
JJ238-24        CTGCCGCACGCATTGCTGCGCCCGTGCGTGGCGCTACTGGACGAACTGGCGCGCAACTAC
JK2-10          CTGCCGCACGCATTGCTGCGCCCGTGCGTGGCGCTACTGGACGAACTGGCGCGCAACTAC
JK4-1           CTGCCGCACGCATTGCTGCGCCCGTGCGTGGCGCTACTGGACGAACTGGCGCGCAACTAC
JK143-11        CTGCCGCACGCATTGCTGCGCCCGTGCGTGGCGCTACTGGACGAACTGGCGCGCAACTAC
JS581           CTGCCGCACGCATTGCTGCGCCCGTGCGTGGCGCTACTGGACGAACTGGCGCGCAACTAC
JS584           CTGCCGCACGCATTGCTGCGCCCGTGCGTGGCGCTACTGGACGAACTGGCGCGCAACTAC
JW160-1         CTGCCGCACGCATTGCTGCGCCCGTGCGTGGCGCTACTGGACGAACTGGCGCGCAACTAC
LB100-1         CTGCCGCACGCATTGCTGCGCCCGTGCGTGGCGCTACTGGACGAACTGGCGCGCAACTAC
LC80            CTGCCGCACGCATTGCTGCGCCCGTGCGTGGCGCTACTGGACGAACTGGCGCGCAACTAC
LD71a           CTGCCGCACGCATTGCTGCGCCCGTGCGTGGCGCTACTGGACGAACTGGCGCGCAACTAC
LE20-1          CTGCCGCACGCATTGCTGCGCCCGTGCGTGGCGCTACTGGACGAACTGGCGCGCAACTAC
LG115           CTGCCGCACGCATTGCTGCGCCCGTGCGTGGCGCTACTGGACGAACTGGCGCGCAACTAC
LG117           CTGCCGCACGCATTGCTGCGCCCGTGCGTGGCGCTACTGGACGAACTGGCGCGCAACTAC
LH37-1          CTGCCGCACGCATTGCTGCGCCCGTGCGTGGCGCTACTGGACGAACTGGCGCGCAACTAC
LMG9322         CTGCCGCACGCATTGCTGCGCCCGTGCGTGGCGCTACTGGACGAACTGGCGCGCAACTAC
NCPPB3562       CTGCCGCACGCATTGCTGCGCCCGTGCGTGGCGCTACTGGACGAACTGGCGCGCAACTAC
NCPPB3607       CTGCCGCACGCATTGCTGCGCCCGTGCGTGGCGCTACTGGACGAACTGGCGCGCAACTAC
NCPPB3608       CTGCCGCACGCATTGCTGCGCCCGTGCGTGGCGCTACTGGACGAACTGGCGCGCAACTAC
X2003-3218      CTGCCGCACGCATTGCTGCGCCCGTGCGTGGCGCTACTGGACGAACTGGCGCGCAACTAC
JF90-2          CTGCCGCACGCATTGCTGCGCCCGTGCGTGGCGCTACTGGACGAACTGGCGCGCAACTAC
JM35            CTGCCGCACGCATTGCTGCGCCCGTGCGTGGCGCTACTGGACGAACTGGCGCGCAACTAC
                ************************************************************
```

```
LG97            CGGGTCAGTCCGGTGTTGTGCACCGCCACGCAACCCACGCTGCGCCTGGACCAGGGCTTC
NCPPB3612       CGGGTCAGTCCGGTGTTGTGCACCGCCACGCAACCCACGCTGCGCCTGGACCAGGGCTTC
C40             CGGGTCAGTCCGGTGTTGTGCACCGCCACGCAACCCACGCTGCGCCTGGACCAGGGCTTC
CFBP2852        CGGGTCAGTCCGGTGTTGTGCACCGCCACGCAACCCACGCTGCGCCTGGACCAGGGCTTC
CFBP2911        CGGGTCAGTCCGGTGTTGTGCACCGCCACGCAACCCACGCTGCGCCTGGACCAGGGCTTC
FDC217          CGGGTCAGTCCGGTGTTGTGCACCGCCACGCAACCCACGCTGCGCCTGGACCAGGGCTTC
FDC1083         CGGGTCAGTCCGGTGTTGTGCACCGCCACGCAACCCACGCTGCGCCTGGACCAGGGCTTC
JF90-8          CGGGTCAGTCCGGTGTTGTGCACCGCCACGCAACCCACGCTGCGCCTGGACCAGGGCTTC
JJ10-1          CGGGTCAGTCCGGTGTTGTGCACCGCCACGCAACCCACGCTGCGCCTGGACCAGGGCTTC
JJ238-10        CGGGTCAGTCCGGTGTTGTGCACCGCCACGCAACCCACGCTGCGCCTGGACCAGGGCTTC
JJ238-24        CGGGTCAGTCCGGTGTTGTGCACCGCCACGCAACCCACGCTGCGCCTGGACCAGGGCTTC
JK2-10          CGGGTCAGTCCGGTGTTGTGCACCGCCACGCAACCCACGCTGCGCCTGGACCAGGGCTTC
JK4-1           CGGGTCAGTCCGGTGTTGTGCACCGCCACGCAACCCACGCTGCGCCTGGACCAGGGCTTC
JK143-11        CGGGTCAGTCCGGTGTTGTGCACCGCCACGCAACCCACGCTGCGCCTGGACCAGGGCTTC
JS581           CGGGTCAGTCCGGTGTTGTGCACCGCCACGCAACCCACGCTGCGCCTGGACCAGGGCTTC
JS584           CGGGTCAGTCCGGTGTTGTGCACCGCCACGCAACCCACGCTGCGCCTGGACCAGGGCTTC
JW160-1         CGGGTCAGTCCGGTGTTGTGCACCGCCACGCAACCCACGCTGCGCCTGGACCAGGGCTTC
LB100-1         CGGGTCAGTCCGGTGTTGTGCACCGCCACGCAACCCACGCTGCGCCTGGACCAGGGCTTC
LC80            CGGGTCAGTCCGGTGTTGTGCACCGCCACGCAACCCACGCTGCGCCTGGACCAGGGCTTC
LD71a           CGGGTCAGTCCGGTGTTGTGCACCGCCACGCAACCCACGCTGCGCCTGGACCAGGGCTTC
LE20-1          CGGGTCAGTCCGGTGTTGTGCACCGCCACGCAACCCACGCTGCGCCTGGACCAGGGCTTC
LG115           CGGGTCAGTCCGGTGTTGTGCACCGCCACGCAACCCACGCTGCGCCTGGACCAGGGCTTC
LG117           CGGGTCAGTCCGGTGTTGTGCACCGCCACGCAACCCACGCTGCGCCTGGACCAGGGCTTC
LH37-1          CGGGTCAGTCCGGTGTTGTGCACCGCCACGCAACCCACGCTGCGCCTGGACCAGGGCTTC
LMG9322         CGGGTCAGTCCGGTGTTGTGCACCGCCACGCAACCCACGCTGCGCCTGGACCAGGGCTTC
NCPPB3562       CGGGTCAGTCCGGTGTTGTGCACCGCCACGCAACCCACGCTGCGCCTGGACCAGGGCTTC
NCPPB3607       CGGGTCAGTCCGGTGTTGTGCACCGCCACGCAACCCACGCTGCGCCTGGACCAGGGCTTC
NCPPB3608       CGGGTCAGTCCGGTGTTGTGCACCGCCACGCAACCCACGCTGCGCCTGGACCAGGGCTTC
X2003-3218      CGGGTCAGTCCGGTGTTGTGCACCGCCACGCAACCCACGCTGCGCCTGGACCAGGGCTTC
JF90-2          CGGGTCAGTCCGGTGTTGTGCACCGCCACGCAACCCACGCTGCGCCTGGACCAGGGCTTC
JM35            CGGGTCAGTCCGGTGTTGTGCACCGCCACGCAACCCACGCTGCGCCTGGACCAGGGCTTC
                ************************************************************

LG97            GTTGGGGGTCTGGACCAGGTGCGAGAGCTGGTGGACGACCCACCGGCGCTGTATGCAAGG
NCPPB3612       GTTGGGGGTCTGGACCAGGTGCGAGAGCTGGTGGACGACCCACCGGCGCTGTATGCAAGG
C40             GTTGGGGGTCTGGACCAGGTGCGAGAGCTGGTGGACGACCCACCGGCGCTGTATGCAAGG
CFBP2852        GTTGGGGGTCTGGACCAGGTGCGAGAGCTGGTGGACGACCCACCGGCGCTGTATGCAAGG
CFBP2911        GTTGGGGGTCTGGACCAGGTGCGAGAGCTGGTGGACGACCCACCGGCGCTGTATGCAAGG
FDC217          GTTGGGGGTCTGGACCAGGTGCGAGAGCTGGTGGACGACCCACCGGCGCTGTATGCAAGG
FDC1083         GTTGGGGGTCTGGACCAGGTGCGAGAGCTGGTGGACGACCCACCGGCGCTGTATGCAAGG
JF90-8          GTTGGGGGTCTGGACCAGGTGCGAGAGCTGGTGGACGACCCACCGGCGCTGTATGCAAGG
JJ10-1          GTTGGGGGTCTGGACCAGGTGCGAGAGCTGGTGGACGACCCACCGGCGCTGTATGCAAGG
JJ238-10        GTTGGGGGTCTGGACCAGGTGCGAGAGCTGGTGGACGACCCACCGGCGCTGTATGCAAGG
JJ238-24        GTTGGGGGTCTGGACCAGGTGCGAGAGCTGGTGGACGACCCACCGGCGCTGTATGCAAGG
JK2-10          GTTGGGGGTCTGGACCAGGTGCGAGAGCTGGTGGACGACCCACCGGCGCTGTATGCAAGG
JK4-1           GTTGGGGGTCTGGACCAGGTGCGAGAGCTGGTGGACGACCCACCGGCGCTGTATGCAAGG
JK143-11        GTTGGGGGTCTGGACCAGGTGCGAGAGCTGGTGGACGACCCACCGGCGCTGTATGCAAGG
JS581           GTTGGGGGTCTGGACCAGGTGCGAGAGCTGGTGGACGACCCACCGGCGCTGTATGCAAGG
JS584           GTTGGGGGTCTGGACCAGGTGCGAGAGCTGGTGGACGACCCACCGGCGCTGTATGCAAGG
JW160-1         GTTGGGGGTCTGGACCAGGTGCGAGAGCTGGTGGACGACCCACCGGCGCTGTATGCAAGG
LB100-1         GTTGGGGGTCTGGACCAGGTGCGAGAGCTGGTGGACGACCCACCGGCGCTGTATGCAAGG
LC80            GTTGGGGGTCTGGACCAGGTGCGAGAGCTGGTGGACGACCCACCGGCGCTGTATGCAAGG
LD71a           GTTGGGGGTCTGGACCAGGTGCGAGAGCTGGTGGACGACCCACCGGCGCTGTATGCAAGG
LE20-1          GTTGGGGGTCTGGACCAGGTGCGAGAGCTGGTGGACGACCCACCGGCGCTGTATGCAAGG
LG115           GTTGGGGGTCTGGACCAGGTGCGAGAGCTGGTGGACGACCCACCGGCGCTGTATGCAAGG
LG117           GTTGGGGGTCTGGACCAGGTGCGAGAGCTGGTGGACGACCCACCGGCGCTGTATGCAAGG
LH37-1          GTTGGGGGTCTGGACCAGGTGCGAGAGCTGGTGGACGACCCACCGGCGCTGTATGCAAGG
LMG9322         GTTGGGGGTCTGGACCAGGTGCGAGAGCTGGTGGACGACCCACCGGCGCTGTATGCAAGG
NCPPB3562       GTTGGGGGTCTGGACCAGGTGCGAGAGCTGGTGGACGACCCACCGGCGCTGTATGCAAGG
NCPPB3607       GTTGGGGGTCTGGACCAGGTGCGAGAGCTGGTGGACGACCCACCGGCGCTGTATGCAAGG
NCPPB3608       GTTGGGGGTCTGGACCAGGTGCGAGAGCTGGTGGACGACCCACCGGCGCTGTATGCAAGG
X2003-3218      GTTGGGGGTCTGGACCAGGTGCGAGAGCTGGTGGACGACCCACCGGCGCTGTATGCAAGG
JF90-2          GTTGGGGGTCTGGACCAGGTGCGAGAGCTGGTGGACGACCCACCGGCGCTGTATGCAAGG
JM35            GTTGGGGGTCTGGACCAGGTGCGAGAGCTGGTGGACGACCCACCGGCGCTGTATGCAAGG
                ************************************************************
```

```
LG97            CTACGACGCGTCCAGGTGCGCCACGTCGGCACGCTCGACGACGCTGCGCTGACCGACCAC
NCPPB3612       CTACGACGCGTCCAGGTGCGCCACGTCGGCACGCTCGACGACGCTGCGCTGACCGACCAC
C40             CTACGACGCGTCCAGGTGCGCCACGTCGGCACGCTCGACGACGCTGCGCTGACCGACCAC
CFBP2852        CTACGACGCGTCCAGGTGCGCCACGTCGGCACGCTCGACGACGCTGCGCTGACCGACCAC
CFBP2911        CTACGACGCGTCCAGGTGCGCCACGTCGGCACGCTCGACGACGCTGCGCTGACCGACCAC
FDC217          CTACGACGCGTCCAGGTGCGCCACGTCGGCACGCTCGACGACGCTGCGCTGACCGACCAC
FDC1083         CTACGACGCGTCCAGGTGCGCCACGTCGGCACGCTCGACGACGCTGCGCTGACCGACCAC
JF90-8          CTACGACGCGTCCAGGTGCGCCACGTCGGCACGCTCGACGACGCTGCGCTGACCGACCAC
JJ10-1          CTACGACGCGTCCAGGTGCGCCACGTCGGCACGCTCGACGACGCTGCGCTGACCGACCAC
JJ238-10        CTACGACGCGTCCAGGTGCGCCACGTCGGCACGCTCGACGACGCTGCGCTGACCGACCAC
JJ238-24        CTACGACGCGTCCAGGTGCGCCACGTCGGCACGCTCGACGACGCTGCGCTGACCGACCAC
JK2-10          CTACGACGCGTCCAGGTGCGCCACGTCGGCACGCTCGACGACGCTGCGCTGACCGACCAC
JK4-1           CTACGACGCGTCCAGGTGCGCCACGTCGGCACGCTCGACGACGCTGCGCTGACCGACCAC
JK143-11        CTACGACGCGTCCAGGTGCGCCACGTCGGCACGCTCGACGACGCTGCGCTGACCGACCAC
JS581           CTACGACGCGTCCAGGTGCGCCACGTCGGCACGCTCGACGACGCTGCGCTGACCGACCAC
JS584           CTACGACGCGTCCAGGTGCGCCACGTCGGCACGCTCGACGACGCTGCGCTGACCGACCAC
JW160-1         CTACGACGCGTCCAGGTGCGCCACGTCGGCACGCTCGACGACGCTGCGCTGACCGACCAC
LB100-1         CTACGACGCGTCCAGGTGCGCCACGTCGGCACGCTCGACGACGCTGCGCTGACCGACCAC
LC80            CTACGACGCGTCCAGGTGCGCCACGTCGGCACGCTCGACGACGCTGCGCTGACCGACCAC
LD71a           CTACGACGCGTCCAGGTGCGCCACGTCGGCACGCTCGACGACGCTGCGCTGACCGACCAC
LE20-1          CTACGACGCGTCCAGGTGCGCCACGTCGGCACGCTCGACGACGCTGCGCTGACCGACCAC
LG115           CTACGACGCGTCCAGGTGCGCCACGTCGGCACGCTCGACGACGCTGCGCTGACCGACCAC
LG117           CTACGACGCGTCCAGGTGCGCCACGTCGGCACGCTCGACGACGCTGCGCTGACCGACCAC
LH37-1          CTACGACGCGTCCAGGTGCGCCACGTCGGCACGCTCGACGACGCTGCGCTGACCGACCAC
LMG9322         CTACGACGCGTCCAGGTGCGCCACGTCGGCACGCTCGACGACGCTGCGCTGACCGACCAC
NCPPB3562       CTACGACGCGTCCAGGTGCGCCACGTCGGCACGCTCGACGACGCTGCGCTGACCGACCAC
NCPPB3607       CTACGACGCGTCCAGGTGCGCCACGTCGGCACGCTCGACGACGCTGCGCTGACCGACCAC
NCPPB3608       CTACGACGCGTCCAGGTGCGCCACGTCGGCACGCTCGACGACGCTGCGCTGACCGACCAC
X2003-3218      CTACGACGCGTCCAGGTGCGCCACGTCGGCACGCTCGACGACGCTGCGCTGACCGACCAC
JF90-2          CTACGACGCGTCCAGGTGCGCCACGTCGGCACGCTCGACGACGCTGCGCTGACCGACCAC
JM35            CTACGACGCGTCCAGGTGCGCCACGTCGGCACGCTCGACGACGCTGCGCTGACCGACCAC
                ************************************************************

LG97            CTCCGGCAACGCGAACAGGTGCTGTGCATCGTCAACAATCGTCGCCACGCGCGGCACCTG
NCPPB3612       CTCCGGCAACGCGAACAGGTGCTGTGCATCGTCAACAATCGTCGCCACGCGCGGCACCTG
C40             CTCCGGCAACGCGAACAGGTGCTGTGCATCGTCAACAATCGTCGCCACGCGCGGCACCTG
CFBP2852        CTCCGGCAACGCGAACAGGTGCTGTGCATCGTCAACAATCGTCGCCACGCGCGGCACCTG
CFBP2911        CTCCGGCAACGCGAACAGGTGCTGTGCATCGTCAACAATCGTCGCCACGCGCGGCACCTG
FDC217          CTCCGGCAACGCGAACAGGTGCTGTGCATCGTCAACAATCGTCGCCACGCGCGGCACCTG
FDC1083         CTCCGGCAACGCGAACAGGTGCTGTGCATCGTCAACAATCGTCGCCACGCGCGGCACCTG
JF90-8          CTCCGGCAACGCGAACAGGTGCTGTGCATCGTCAACAATCGTCGCCACGCGCGGCACCTG
JJ10-1          CTCCGGCAACGCGAACAGGTGCTGTGCATCGTCAACAATCGTCGCCACGCGCGGCACCTG
JJ238-10        CTCCGGCAACGCGAACAGGTGCTGTGCATCGTCAACAATCGTCGCCACGCGCGGCACCTG
JJ238-24        CTCCGGCAACGCGAACAGGTGCTGTGCATCGTCAACAATCGTCGCCACGCGCGGCACCTG
JK2-10          CTCCGGCAACGCGAACAGGTGCTGTGCATCGTCAACAATCGTCGCCACGCGCGGCACCTG
JK4-1           CTCCGGCAACGCGAACAGGTGCTGTGCATCGTCAACAATCGTCGCCACGCGCGGCACCTG
JK143-11        CTCCGGCAACGCGAACAGGTGCTGTGCATCGTCAACAATCGTCGCCACGCGCGGCACCTG
JS581           CTCCGGCAACGCGAACAGGTGCTGTGCATCGTCAACAATCGTCGCCACGCGCGGCACCTG
JS584           CTCCGGCAACGCGAACAGGTGCTGTGCATCGTCAACAATCGTCGCCACGCGCGGCACCTG
JW160-1         CTCCGGCAACGCGAACAGGTGCTGTGCATCGTCAACAATCGTCGCCACGCGCGGCACCTG
LB100-1         CTCCGGCAACGCGAACAGGTGCTGTGCATCGTCAACAATCGTCGCCACGCGCGGCACCTG
LC80            CTCCGGCAACGCGAACAGGTGCTGTGCATCGTCAACAATCGTCGCCACGCGCGGCACCTG
LD71a           CTCCGGCAACGCGAACAGGTGCTGTGCATCGTCAACAATCGTCGCCACGCGCGGCACCTG
LE20-1          CTCCGGCAACGCGAACAGGTGCTGTGCATCGTCAACAATCGTCGCCACGCGCGGCACCTG
LG115           CTCCGGCAACGCGAACAGGTGCTGTGCATCGTCAACAATCGTCGCCACGCGCGGCACCTG
LG117           CTCCGGCAACGCGAACAGGTGCTGTGCATCGTCAACAATCGTCGCCACGCGCGGCACCTG
LH37-1          CTCCGGCAACGCGAACAGGTGCTGTGCATCGTCAACAATCGTCGCCACGCGCGGCACCTG
LMG9322         CTCCGGCAACGCGAACAGGTGCTGTGCATCGTCAACAATCGTCGCCACGCGCGGCACCTG
NCPPB3562       CTCCGGCAACGCGAACAGGTGCTGTGCATCGTCAACAATCGTCGCCACGCGCGGCACCTG
NCPPB3607       CTCCGGCAACGCGAACAGGTGCTGTGCATCGTCAACAATCGTCGCCACGCGCGGCACCTG
NCPPB3608       CTCCGGCAACGCGAACAGGTGCTGTGCATCGTCAACAATCGTCGCCACGCGCGGCACCTG
X2003-3218      CTCCGGCAACGCGAACAGGTGCTGTGCATCGTCAACAATCGTCGCCACGCGCGGCACCTG
JF90-2          CTCCGGCAACGCGAACAGGTGCTGTGCATCGTCAACAATCGTCGCCACGCGCGGCACCTG
JM35            CTCCGGCAACGCGAACAGGTGCTGTGCATCGTCAACAATCGTCGCCACGCGCGGCACCTG
                ************************************************************
```

```
LG97            TTCGACGCGATCGCCGACCGACCCGGTGCACGGCACTTGACCACGCTGATGCACGCGCGC
NCPPB3612       TTCGACGCGATCGCCGACCGACCCGGTGCACGGCACTTGACCACGCTGATGCACGCGCGC
C40             TTCGACGCGATCGCCGACCGACCCGGTGCACGGCACTTGACCACGCTGATGCACGCGCGC
CFBP2852        TTCGACGCGATCGCCGACCGACCCGGTGCACGGCACTTGACCACGCTGATGCACGCGCGC
CFBP2911        TTCGACGCGATCGCCGACCGACCCGGTGCACGGCACTTGACCACGCTGATGCACGCGCGC
FDC217          TTCGACGCGATCGCCGACCGACCCGGTGCACGGCACTTGACCACGCTGATGCACGCGCGC
FDC1083         TTCGACGCGATCGCCGACCGACCCGGTGCACGGCACTTGACCACGCTGATGCACGCGCGC
JF90-8          TTCGACGCGATCGCCGACCGACCCGGTGCACGGCACTTGACCACGCTGATGCACGCGCGC
JJ10-1          TTCGACGCGATCGCCGACCGACCCGGTGCACGGCACTTGACCACGCTGATGCACGCGCGC
JJ238-10        TTCGACGCGATCGCCGACCGACCCGGTGCACGGCACTTGACCACGCTGATGCACGCGCGC
JJ238-24        TTCGACGCGATCGCCGACCGACCCGGTGCACGGCACTTGACCACGCTGATGCACGCGCGC
JK2-10          TTCGACGCGATCGCCGACCGACCCGGTGCACGGCACTTGACCACGCTGATGCACGCGCGC
JK4-1           TTCGACGCGATCGCCGACCGACCCGGTGCACGGCACTTGACCACGCTGATGCACGCGCGC
JK143-11        TTCGACGCGATCGCCGACCGACCCGGTGCACGGCACTTGACCACGCTGATGCACGCGCGC
JS581           TTCGACGCGATCGCCGACCGACCCGGTGCACGGCACTTGACCACGCTGATGCACGCGCGC
JS584           TTCGACGCGATCGCCGACCGACCCGGTGCACGGCACTTGACCACGCTGATGCACGCGCGC
JW160-1         TTCGACGCGATCGCCGACCGACCCGGTGCACGGCACTTGACCACGCTGATGCACGCGCGC
LB100-1         TTCGACGCGATCGCCGACCGACCCGGTGCACGGCACTTGACCACGCTGATGCACGCGCGC
LC80            TTCGACGCGATCGCCGACCGACCCGGTGCACGGCACTTGACCACGCTGATGCACGCGCGC
LD71a           TTCGACGCGATCGCCGACCGACCCGGTGCACGGCACTTGACCACGCTGATGCACGCGCGC
LE20-1          TTCGACGCGATCGCCGACCGACCCGGTGCACGGCACTTGACCACGCTGATGCACGCGCGC
LG115           TTCGACGCGATCGCCGACCGACCCGGTGCACGGCACTTGACCACGCTGATGCACGCGCGC
LG117           TTCGACGCGATCGCCGACCGACCCGGTGCACGGCACTTGACCACGCTGATGCACGCGCGC
LH37-1          TTCGACGCGATCGCCGACCGACCCGGTGCACGGCACTTGACCACGCTGATGCACGCGCGC
LMG9322         TTCGACGCGATCGCCGACCGACCCGGTGCACGGCACTTGACCACGCTGATGCACGCGCGC
NCPPB3562       TTCGACGCGATCGCCGACCGACCCGGTGCACGGCACTTGACCACGCTGATGCACGCGCGC
NCPPB3607       TTCGACGCGATCGCCGACCGACCCGGTGCACGGCACTTGACCACGCTGATGCACGCGCGC
NCPPB3608       TTCGACGCGATCGCCGACCGACCCGGTGCACGGCACTTGACCACGCTGATGCACGCGCGC
X2003-3218      TTCGACGCGATCGCCGACCGACCCGGTGCACGGCACTTGACCACGCTGATGCACGCGCGC
JF90-2          TTCGACGCGATCGCCGACCGACCCGGTGCACGGCACTTGACCACGCTGATGCACGCGCGC
JM35            TTCGACGCGATCGCCGACCGACCCGGTGCACGGCACTTGACCACGCTGATGCACGCGCGC
                ************************************************************

LG97            CACCGCAGCGCGGTGCTGGCCGAACTGCGCGAAGACCTGAAAGCAGGCCGGCCATGCCGG
NCPPB3612       CACCGCAGCGCGGTGCTGGCCGAACTGCGCGAAGACCTGAAAGCAGGCCGGCCATGCCGG
C40             CACCGCAGCGCGGTGCTGGCCGAACTGCGCGAAGACCTGAAAGCAGGCCGGCCATGCCGG
CFBP2852        CACCGCAGCGCGGTGCTGGCCGAACTGCGCGAAGACCTGAAAGCAGGCCGGCCATGCCGG
CFBP2911        CACCGCAGCGCGGTGCTGGCCGAACTGCGCGAAGACCTGAAAGCAGGCCGGCCATGCCGG
FDC217          CACCGCAGCGCGGTGCTGGCCGAACTGCGCGAAGACCTGAAAGCAGGCCGGCCATGCCGG
FDC1083         CACCGCAGCGCGGTGCTGGCCGAACTGCGCGAAGACCTGAAAGCAGGCCGGCCATGCCGG
JF90-8          CACCGCAGCGCGGTGCTGGCCGAACTGCGCGAAGACCTGAAAGCAGGCCGGCCATGCCGG
JJ10-1          CACCGCAGCGCGGTGCTGGCCGAACTGCGCGAAGACCTGAAAGCAGGCCGGCCATGCCGG
JJ238-10        CACCGCAGCGCGGTGCTGGCCGAACTGCGCGAAGACCTGAAAGCAGGCCGGCCATGCCGG
JJ238-24        CACCGCAGCGCGGTGCTGGCCGAACTGCGCGAAGACCTGAAAGCAGGCCGGCCATGCCGG
JK2-10          CACCGCAGCGCGGTGCTGGCCGAACTGCGCGAAGACCTGAAAGCAGGCCGGCCATGCCGG
JK4-1           CACCGCAGCGCGGTGCTGGCCGAACTGCGCGAAGACCTGAAAGCAGGCCGGCCATGCCGG
JK143-11        CACCGCAGCGCGGTGCTGGCCGAACTGCGCGAAGACCTGAAAGCAGGCCGGCCATGCCGG
JS581           CACCGCAGCGCGGTGCTGGCCGAACTGCGCGAAGACCTGAAAGCAGGCCGGCCATGCCGG
JS584           CACCGCAGCGCGGTGCTGGCCGAACTGCGCGAAGACCTGAAAGCAGGCCGGCCATGCCGG
JW160-1         CACCGCAGCGCGGTGCTGGCCGAACTGCGCGAAGACCTGAAAGCAGGCCGGCCATGCCGG
LB100-1         CACCGCAGCGCGGTGCTGGCCGAACTGCGCGAAGACCTGAAAGCAGGCCGGCCATGCCGG
LC80            CACCGCAGCGCGGTGCTGGCCGAACTGCGCGAAGACCTGAAAGCAGGCCGGCCATGCCGG
LD71a           CACCGCAGCGCGGTGCTGGCCGAACTGCGCGAAGACCTGAAAGCAGGCCGGCCATGCCGG
LE20-1          CACCGCAGCGCGGTGCTGGCCGAACTGCGCGAAGACCTGAAAGCAGGCCGGCCATGCCGG
LG115           CACCGCAGCGCGGTGCTGGCCGAACTGCGCGAAGACCTGAAAGCAGGCCGGCCATGCCGG
LG117           CACCGCAGCGCGGTGCTGGCCGAACTGCGCGAAGACCTGAAAGCAGGCCGGCCATGCCGG
LH37-1          CACCGCAGCGCGGTGCTGGCCGAACTGCGCGAAGACCTGAAAGCAGGCCGGCCATGCCGG
LMG9322         CACCGCAGCGCGGTGCTGGCCGAACTGCGCGAAGACCTGAAAGCAGGCCGGCCATGCCGG
NCPPB3562       CACCGCAGCGCGGTGCTGGCCGAACTGCGCGAAGACCTGAAAGCAGGCCGGCCATGCCGG
NCPPB3607       CACCGCAGCGCGGTGCTGGCCGAACTGCGCGAAGACCTGAAAGCAGGCCGGCCATGCCGG
NCPPB3608       CACCGCAGCGCGGTGCTGGCCGAACTGCGCGAAGACCTGAAAGCAGGCCGGCCATGCCGG
X2003-3218      CACCGCAGCGCGGTGCTGGCCGAACTGCGCGAAGACCTGAAAGCAGGCCGGCCATGCCGG
JF90-2          CACCGCAGCGCGGTGCTGGCCGAACTGCGCGAAGACCTGAAAGCAGGCCGGCCATGCCGG
JM35            CACCGCAGCGCGGTGCTGGCCGAACTGCGCGAAGACCTGAAAGCAGGCCGGCCATGCCGG
                ************************************************************
```

```
LG97            CTGGTCGCCACGAGCTTGATAGAAGCGGGGGTGGACGTGGACTTCCCAACGGTGCTGCGC
NCPPB3612       CTGGTCGCCACGAGCTTGATAGAAGCGGGGGTGGACGTGGACTTCCCAACGGTGCTGCGC
C40             CTGGTCGCCACGAGCTTGATAGAAGCGGGGGTGGACGTGGACTTCCCAACGGTGCTGCGC
CFBP2852        CTGGTCGCCACGAGCTTGATAGAAGCGGGGGTGGACGTGGACTTCCCAACGGTGCTGCGC
CFBP2911        CTGGTCGCCACGAGCTTGATAGAAGCGGGGGTGGACGTGGACTTCCCAACGGTGCTGCGC
FDC217          CTGGTCGCCACGAGCTTGATAGAAGCGGGGGTGGACGTGGACTTCCCAACGGTGCTGCGC
FDC1083         CTGGTCGCCACGAGCTTGATAGAAGCGGGGGTGGACGTGGACTTCCCAACGGTGCTGCGC
JF90-8          CTGGTCGCCACGAGCTTGATAGAAGCGGGGGTGGACGTGGACTTCCCAACGGTGCTGCGC
JJ10-1          CTGGTCGCCACGAGCTTGATAGAAGCGGGGGTGGACGTGGACTTCCCAACGGTGCTGCGC
JJ238-10        CTGGTCGCCACGAGCTTGATAGAAGCGGGGGTGGACGTGGACTTCCCAACGGTGCTGCGC
JJ238-24        CTGGTCGCCACGAGCTTGATAGAAGCGGGGGTGGACGTGGACTTCCCAACGGTGCTGCGC
JK2-10          CTGGTCGCCACGAGCTTGATAGAAGCGGGGGTGGACGTGGACTTCCCAACGGTGCTGCGC
JK4-1           CTGGTCGCCACGAGCTTGATAGAAGCGGGGGTGGACGTGGACTTCCCAACGGTGCTGCGC
JK143-11        CTGGTCGCCACGAGCTTGATAGAAGCGGGGGTGGACGTGGACTTCCCAACGGTGCTGCGC
JS581           CTGGTCGCCACGAGCTTGATAGAAGCGGGGGTGGACGTGGACTTCCCAACGGTGCTGCGC
JS584           CTGGTCGCCACGAGCTTGATAGAAGCGGGGGTGGACGTGGACTTCCCAACGGTGCTGCGC
JW160-1         CTGGTCGCCACGAGCTTGATAGAAGCGGGGGTGGACGTGGACTTCCCAACGGTGCTGCGC
LB100-1         CTGGTCGCCACGAGCTTGATAGAAGCGGGGGTGGACGTGGACTTCCCAACGGTGCTGCGC
LC80            CTGGTCGCCACGAGCTTGATAGAAGCGGGGGTGGACGTGGACTTCCCAACGGTGCTGCGC
LD71a           CTGGTCGCCACGAGCTTGATAGAAGCGGGGGTGGACGTGGACTTCCCAACGGTGCTGCGC
LE20-1          CTGGTCGCCACGAGCTTGATAGAAGCGGGGGTGGACGTGGACTTCCCAACGGTGCTGCGC
LG115           CTGGTCGCCACGAGCTTGATAGAAGCGGGGGTGGACGTGGACTTCCCAACGGTGCTGCGC
LG117           CTGGTCGCCACGAGCTTGATAGAAGCGGGGGTGGACGTGGACTTCCCAACGGTGCTGCGC
LH37-1          CTGGTCGCCACGAGCTTGATAGAAGCGGGGGTGGACGTGGACTTCCCAACGGTGCTGCGC
LMG9322         CTGGTCGCCACGAGCTTGATAGAAGCGGGGGTGGACGTGGACTTCCCAACGGTGCTGCGC
NCPPB3562       CTGGTCGCCACGAGCTTGATAGAAGCGGGGGTGGACGTGGACTTCCCAACGGTGCTGCGC
NCPPB3607       CTGGTCGCCACGAGCTTGATAGAAGCGGGGGTGGACGTGGACTTCCCAACGGTGCTGCGC
NCPPB3608       CTGGTCGCCACGAGCTTGATAGAAGCGGGGGTGGACGTGGACTTCCCAACGGTGCTGCGC
X2003-3218      CTGGTCGCCACGAGCTTGATAGAAGCGGGGGTGGACGTGGACTTCCCAACGGTGCTGCGC
JF90-2          CTGGTCGCCACGAGCTTGATAGAAGCGGGGGTGGACGTGGACTTCCCAACGGTGCTGCGC
JM35            CTGGTCGCCACGAGCTTGATAGAAGCGGGGGTGGACGTGGACTTCCCAACGGTGCTGCGC
                ************************************************************

LG97            GCTAAAGCCGGTCTGGATTCCATCGCCCAGGCTGCCGGCCGCTGCAACCGCGAAGGCTTG
NCPPB3612       GCTGAAGCCGGTCTGGATTCCATCGCCCAGGCTGCCGGCCGCTGCAACCGCGAAGGCTTG
C40             GCTGAAGCCGGTCTGGATTCCATCGCCCAGGCTGCCGGCCGCTGCAACCGCGAAGGCTTG
CFBP2852        GCTGAAGCCGGTCTGGATTCCATCGCCCAGGCTGCCGGCCGCTGCAACCGCGAAGGCTTG
CFBP2911        GCTGAAGCCGGTCTGGATTCCATCGCCCAGGCTGCCGGCCGCTGCAACCGCGAAGGCTTG
FDC217          GCTGAAGCCGGTCTGGATTCCATCGCCCAGGCTGCCGGCCGCTGCAACCGCGAAGGCTTG
FDC1083         GCTGAAGCCGGTCTGGATTCCATCGCCCAGGCTGCCGGCCGCTGCAACCGCGAAGGCTTG
JF90-8          GCTGAAGCCGGTCTGGATTCCATCGCCCAGGCTGCCGGCCGCTGCAACCGCGAAGGCTTG
JJ10-1          GCTGAAGCCGGTCTGGATTCCATCGCCCAGGCTGCCGGCCGCTGCAACCGCGAAGGCTTG
JJ238-10        GCTGAAGCCGGTCTGGATTCCATCGCCCAGGCTGCCGGCCGCTGCAACCGCGAAGGCTTG
JJ238-24        GCTGAAGCCGGTCTGGATTCCATCGCCCAGGCTGCCGGCCGCTGCAACCGCGAAGGCTTG
JK2-10          GCTGAAGCCGGTCTGGATTCCATCGCCCAGGCTGCCGGCCGCTGCAACCGCGAAGGCTTG
JK4-1           GCTGAAGCCGGTCTGGATTCCATCGCCCAGGCTGCCGGCCGCTGCAACCGCGAAGGCTTG
JK143-11        GCTGAAGCCGGTCTGGATTCCATCGCCCAGGCTGCCGGCCGCTGCAACCGCGAAGGCTTG
JS581           GCTGAAGCCGGTCTGGATTCCATCGCCCAGGCTGCCGGCCGCTGCAACCGCGAAGGCTTG
JS584           GCTGAAGCCGGTCTGGATTCCATCGCCCAGGCTGCCGGCCGCTGCAACCGCGAAGGCTTG
JW160-1         GCTGAAGCCGGTCTGGATTCCATCGCCCAGGCTGCCGGCCGCTGCAACCGCGAAGGCTTG
LB100-1         GCTGAAGCCGGTCTGGATTCCATCGCCCAGGCTGCCGGCCGCTGCAACCGCGAAGGCTTG
LC80            GCTGAAGCCGGTCTGGATTCCATCGCCCAGGCTGCCGGCCGCTGCAACCGCGAAGGCTTG
LD71a           GCTGAAGCCGGTCTGGATTCCATCGCCCAGGCTGCCGGCCGCTGCAACCGCGAAGGCTTG
LE20-1          GCTGAAGCCGGTCTGGATTCCATCGCCCAGGCTGCCGGCCGCTGCAACCGCGAAGGCTTG
LG115           GCTGAAGCCGGTCTGGATTCCATCGCCCAGGCTGCCGGCCGCTGCAACCGCGAAGGCTTG
LG117           GCTGAAGCCGGTCTGGATTCCATCGCCCAGGCTGCCGGCCGCTGCAACCGCGAAGGCTTG
LH37-1          GCTGAAGCCGGTCTGGATTCCATCGCCCAGGCTGCCGGCCGCTGCAACCGCGAAGGCTTG
LMG9322         GCTGAAGCCGGTCTGGATTCCATCGCCCAGGCTGCCGGCCGCTGCAACCGCGAAGGCTTG
NCPPB3562       GCTGAAGCCGGTCTGGATTCCATCGCCCAGGCTGCCGGCCGCTGCAACCGCGAAGGCTTG
NCPPB3607       GCTGAAGCCGGTCTGGATTCCATCGCCCAGGCTGCCGGCCGCTGCAACCGCGAAGGCTTG
NCPPB3608       GCTGAAGCCGGTCTGGATTCCATCGCCCAGGCTGCCGGCCGCTGCAACCGCGAAGGCTTG
X2003-3218      GCTGAAGCCGGTCTGGATTCCATCGCCCAGGCTGCCGGCCGCTGCAACCGCGAAGGCTTG
JF90-2          GCTGAAGCCGGTCTGGATTCCATCGCCCAGGCTGCCGGCCGCTGCAACCGCGAAGGCTTG
JM35            GCTGAAGCCGGTCTGGATTCCATCGCCCAGGCTGCCGGCCGCTGCAACCGCGAAGGCTTG
                *** ********************************************************
```

```
LG97            CGGCCGGTAGAGGTCAGCCAAGTGCAGGTATTCGCGCCGGCCAATCCCGACTGGGCACCG
NCPPB3612       CGGCCGGTAGAGGTCAGCCAAGTGCAGGTATTCGCGCCGGCCAATCCCGACTGGGCACCG
C40             CGGCCGGTAGAGGTCAGCCAAGTGCAGGTATTCGCGCCGGCCAATCCCGACTGGGCACCG
CFBP2852        CGGCCGGTAGAGGTCAGCCAAGTGCAGGTATTCGCGCCGGCCAATCCCGACTGGGCACCG
CFBP2911        CGGCCGGTAGAGGTCAGCCAAGTGCAGGTATTCGCGCCGGCCAATCCCGACTGGGCACCG
FDC217          CGGCCGGTAGAGGTCAGCCAAGTGCAGGTATTCGCGCCGGCCAATCCCGACTGGGCACCG
FDC1083         CGGCCGGTAGAGGTCAGCCAAGTGCAGGTATTCGCGCCGGCCAATCCCGACTGGGCACCG
JF90-8          CGGCCGGTAGAGGTCAGCCAAGTGCAGGTATTCGCGCCGGCCAATCCCGACTGGGCACCG
JJ10-1          CGGCCGGTAGAGGTCAGCCAAGTGCAGGTATTCGCGCCGGCCAATCCCGACTGGGCACCG
JJ238-10        CGGCCGGTAGAGGTCAGCCAAGTGCAGGTATTCGCGCCGGCCAATCCCGACTGGGCACCG
JJ238-24        CGGCCGGTAGAGGTCAGCCAAGTGCAGGTATTCGCGCCGGCCAATCCCGACTGGGCACCG
JK2-10          CGGCCGGTAGAGGTCAGCCAAGTGCAGGTATTCGCGCCGGCCAATCCCGACTGGGCACCG
JK4-1           CGGCCGGTAGAGGTCAGCCAAGTGCAGGTATTCGCGCCGGCCAATCCCGACTGGGCACCG
JK143-11        CGGCCGGTAGAGGTCAGCCAAGTGCAGGTATTCGCGCCGGCCAATCCCGACTGGGCACCG
JS581           CGGCCGGTAGAGGTCAGCCAAGTGCAGGTATTCGCGCCGGCCAATCCCGACTGGGCACCG
JS584           CGGCCGGTAGAGGTCAGCCAAGTGCAGGTATTCGCGCCGGCCAATCCCGACTGGGCACCG
JW160-1         CGGCCGGTAGAGGTCAGCCAAGTGCAGGTATTCGCGCCGGCCAATCCCGACTGGGCACCG
LB100-1         CGGCCGGTAGAGGTCAGCCAAGTGCAGGTATTCGCGCCGGCCAATCCCGACTGGGCACCG
LC80            CGGCCGGTAGAGGTCAGCCAAGTGCAGGTATTCGCGCCGGCCAATCCCGACTGGGCACCG
LD71a           CGGCCGGTAGAGGTCAGCCAAGTGCAGGTATTCGCGCCGGCCAATCCCGACTGGGCACCG
LE20-1          CGGCCGGTAGAGGTCAGCCAAGTGCAGGTATTCGCGCCGGCCAATCCCGACTGGGCACCG
LG115           CGGCCGGTAGAGGTCAGCCAAGTGCAGGTATTCGCGCCGGCCAATCCCGACTGGGCACCG
LG117           CGGCCGGTAGAGGTCAGCCAAGTGCAGGTATTCGCGCCGGCCAATCCCGACTGGGCACCG
LH37-1          CGGCCGGTAGAGGTCAGCCAAGTGCAGGTATTCGCGCCGGCCAATCCCGACTGGGCACCG
LMG9322         CGGCCGGTAGAGGTCAGCCAAGTGCAGGTATTCGCGCCGGCCAATCCCGACTGGGCACCG
NCPPB3562       CGGCCGGTAGAGGTCAGCCAAGTGCAGGTATTCGCGCCGGCCAATCCCGACTGGGCACCG
NCPPB3607       CGGCCGGTAGAGGTCAGCCAAGTGCAGGTATTCGCGCCGGCCAATCCCGACTGGGCACCG
NCPPB3608       CGGCCGGTAGAGGTCAGCCAAGTGCAGGTATTCGCGCCGGCCAATCCCGACTGGGCACCG
X2003-3218      CGGCCGGTAGAGGTCAGCCAAGTGCAGGTATTCGCGCCGGCCAATCCCGACTGGGCACCG
JF90-2          CGGCCGGTAGAGGTCAGCCAAGTGCAGGTATTCGCGCCGGCCAATCCCGACTGGGCACCG
JM35            CGGCCGGTAGAGGTCAGCCAAGTGCAGGTATTCGCGCCGGCCAATCCCGACTGGGCACCG
                ************************************************************

LG97            CCAAAGGAACTAGACCTGTTTGCAGAGGTCTTCCGTGGCATCGAGCGTACCCACCGCAAC
NCPPB3612       CCAAAGGAACTAGACCTGTTTGCAGAGGTCTTCCGTGGCATCGAGCGTACCCACCGCAAC
C40             CCAAAGGAACTAGACCTGTTTGCAGAGGTCTTCCGTGGCATCGAGCGTACCCACCGCAAC
CFBP2852        CCAAAGGAACTAGACCTGTTTGCAGAGGTCTTCCGTGGCATCGAGCGTACCCACCGCAAC
CFBP2911        CCAAAGGAACTAGACCTGTTTGCAGAGGTCTTCCGTGGCATCGAGCGTACCCACCGCAAC
FDC217          CCAAAGGAACTAGACCTGTTTGCAGAGGTCTTCCGTGGCATCGAGCGTACCCACCGCAAC
FDC1083         CCAAAGGAACTAGACCTGTTTGCAGAGGTCTTCCGTGGCATCGAGCGTACCCACCGCAAC
JF90-8          CCAAAGGAACTAGACCTGTTTGCAGAGGTCTTCCGTGGCATCGAGCGTACCCACCGCAAC
JJ10-1          CCAAAGGAACTAGACCTGTTTGCAGAGGTCTTCCGTGGCATCGAGCGTACCCACCGCAAC
JJ238-10        CCAAAGGAACTAGACCTGTTTGCAGAGGTCTTCCGTGGCATCGAGCGTACCCACCGCAAC
JJ238-24        CCAAAGGAACTAGACCTGTTTGCAGAGGTCTTCCGTGGCATCGAGCGTACCCACCGCAAC
JK2-10          CCAAAGGAACTAGACCTGTTTGCAGAGGTCTTCCGTGGCATCGAGCGTACCCACCGCAAC
JK4-1           CCAAAGGAACTAGACCTGTTTGCAGAGGTCTTCCGTGGCATCGAGCGTACCCACCGCAAC
JK143-11        CCAAAGGAACTAGACCTGTTTGCAGAGGTCTTCCGTGGCATCGAGCGTACCCACCGCAAC
JS581           CCAAAGGAACTAGACCTGTTTGCAGAGGTCTTCCGTGGCATCGAGCGTACCCACCGCAAC
JS584           CCAAAGGAACTAGACCTGTTTGCAGAGGTCTTCCGTGGCATCGAGCGTACCCACCGCAAC
JW160-1         CCAAAGGAACTAGACCTGTTTGCAGAGGTCTTCCGTGGCATCGAGCGTACCCACCGCAAC
LB100-1         CCAAAGGAACTAGACCTGTTTGCAGAGGTCTTCCGTGGCATCGAGCGTACCCACCGCAAC
LC80            CCAAAGGAACTAGACCTGTTTGCAGAGGTCTTCCGTGGCATCGAGCGTACCCACCGCAAC
LD71a           CCAAAGGAACTAGACCTGTTTGCAGAGGTCTTCCGTGGCATCGAGCGTACCCACCGCAAC
LE20-1          CCAAAGGAACTAGACCTGTTTGCAGAGGTCTTCCGTGGCATCGAGCGTACCCACCGCAAC
LG115           CCAAAGGAACTAGACCTGTTTGCAGAGGTCTTCCGTGGCATCGAGCGTACCCACCGCAAC
LG117           CCAAAGGAACTAGACCTGTTTGCAGAGGTCTTCCGTGGCATCGAGCGTACCCACCGCAAC
LH37-1          CCAAAGGAACTAGACCTGTTTGCAGAGGTCTTCCGTGGCATCGAGCGTACCCACCGCAAC
LMG9322         CCAAAGGAACTAGACCTGTTTGCAGAGGTCTTCCGTGGCATCGAGCGTACCCACCGCAAC
NCPPB3562       CCAAAGGAACTAGACCTGTTTGCAGAGGTCTTCCGTGGCATCGAGCGTACCCACCGCAAC
NCPPB3607       CCAAAGGAACTAGACCTGTTTGCAGAGGTCTTCCGTGGCATCGAGCGTACCCACCGCAAC
NCPPB3608       CCAAAGGAACTAGACCTGTTTGCAGAGGTCTTCCGTGGCATCGAGCGTACCCACCGCAAC
X2003-3218      CCAAAGGAACTAGACCTGTTTGCAGAGGTCTTCCGTGGCATCGAGCGTACCCACCGCAAC
JF90-2          CCAAAGGAACTAGACCTGTTTGCAGAGGTCTTCCGTGGCATCGAGCGTACCCACCGCAAC
JM35            CCAAAGGAACTAGACCTGTTTGCAGAGGTCTTCCGTGGCATCGAGCGTACCCACCGCAAC
                ************************************************************
```

```
LG97            GACTTGCTGGCGATGGCAGCAGTCGCCGACTACTTCGCGGCGCTCTATCGTCGCCTGGGC
NCPPB3612       GACTTGCTGGCGATGGCAGCAGTCGCCGACTACTTCGCGGCGCTCTATCGTCGCCTGGGC
C40             GACTTGCTGGCGATGGCAGCAGTCGCCGACTACTTCGCGGCGCTCTATCGTCGCCTGGGC
CFBP2852        GACTTGCTGGCGATGGCAGCAGTCGCCGACTACTTCGCGGCGCTCTATCGTCGCCTGGGC
CFBP2911        GACTTGCTGGCGATGGCAGCAGTCGCCGACTACTTCGCGGCGCTCTATCGTCGCCTGGGC
FDC217          GACTTGCTGGCGATGGCAGCAGTCGCCGACTACTTCGCGGCGCTCTATCGTCGCCTGGGC
FDC1083         GACTTGCTGGCGATGGCAGCAGTCGCCGACTACTTCGCGGCGCTCTATCGTCGCCTGGGC
JF90-8          GACTTGCTGGCGATGGCAGCAGTCGCCGACTACTTCGCGGCGCTCTATCGTCGCCTGGGC
JJ10-1          GACTTGCTGGCGATGGCAGCAGTCGCCGACTACTTCGCGGCGCTCTATCGTCGCCTGGGC
JJ238-10        GACTTGCTGGCGATGGCAGCAGTCGCCGACTACTTCGCGGCGCTCTATCGTCGCCTGGGC
JJ238-24        GACTTGCTGGCGATGGCAGCAGTCGCCGACTACTTCGCGGCGCTCTATCGTCGCCTGGGC
JK2-10          GACTTGCTGGCGATGGCAGCAGTCGCCGACTACTTCGCGGCGCTCTATCGTCGCCTGGGC
JK4-1           GACTTGCTGGCGATGGCAGCAGTCGCCGACTACTTCGCGGCGCTCTATCGTCGCCTGGGC
JK143-11        GACTTGCTGGCGATGGCAGCAGTCGCCGACTACTTCGCGGCGCTCTATCGTCGCCTGGGC
JS581           GACTTGCTGGCGATGGCAGCAGTCGCCGACTACTTCGCGGCGCTCTATCGTCGCCTGGGC
JS584           GACTTGCTGGCGATGGCAGCAGTCGCCGACTACTTCGCGGCGCTCTATCGTCGCCTGGGC
JW160-1         GACTTGCTGGCGATGGCAGCAGTCGCCGACTACTTCGCGGCGCTCTATCGTCGCCTGGGC
LB100-1         GACTTGCTGGCGATGGCAGCAGTCGCCGACTACTTCGCGGCGCTCTATCGTCGCCTGGGC
LC80            GACTTGCTGGCGATGGCAGCAGTCGCCGACTACTTCGCGGCGCTCTATCGTCGCCTGGGC
LD71a           GACTTGCTGGCGATGGCAGCAGTCGCCGACTACTTCGCGGCGCTCTATCGTCGCCTGGGC
LE20-1          GACTTGCTGGCGATGGCAGCAGTCGCCGACTACTTCGCGGCGCTCTATCGTCGCCTGGGC
LG115           GACTTGCTGGCGATGGCAGCAGTCGCCGACTACTTCGCGGCGCTCTATCGTCGCCTGGGC
LG117           GACTTGCTGGCGATGGCAGCAGTCGCCGACTACTTCGCGGCGCTCTATCGTCGCCTGGGC
LH37-1          GACTTGCTGGCGATGGCAGCAGTCGCCGACTACTTCGCGGCGCTCTATCGTCGCCTGGGC
LMG9322         GACTTGCTGGCGATGGCAGCAGTCGCCGACTACTTCGCGGCGCTCTATCGTCGCCTGGGC
NCPPB3562       GACTTGCTGGCGATGGCAGCAGTCGCCGACTACTTCGCGGCGCTCTATCGTCGCCTGGGC
NCPPB3607       GACTTGCTGGCGATGGCAGCAGTCGCCGACTACTTCGCGGCGCTCTATCGTCGCCTGGGC
NCPPB3608       GACTTGCTGGCGATGGCAGCAGTCGCCGACTACTTCGCGGCGCTCTATCGTCGCCTGGGC
X2003-3218      GACTTGCTGGCGATGGCAGCAGTCGCCGACTACTTCGCGGCGCTCTATCGTCGCCTGGGC
JF90-2          GACTTGCTGGCGATGGCAGCAGTCGCCGACTACTTCGCGGCGCTCTATCGTCGCCTGGGC
JM35            GACTTGCTGGCGATGGCAGCAGTCGCCGACTACTTCGCGGCGCTCTATCGTCGCCTGGGC
                ************************************************************

LG97            GATGGACGGCTCGACCGCGAGGACGTGCTCGGCCTGCTGCGCGATGCCGACATCAAGGGT
NCPPB3612       GATGGACGGCTCGACCGCGAGGACGTGCTCGGCCTGCTGCGCGATGCCGACATCAAGGGT
C40             GATGGACGGCTCGACCGCGAGGACGTGCTCGGCCTGCTGCGCGATGCCGACATCAAGGGT
CFBP2852        GATGGACGGCTCGACCGCGAGGACGTGCTCGGCCTGCTGCGCGATGCCGACATCAAGGGT
CFBP2911        GATGGACGGCTCGACCGCGAGGACGTGCTCGGCCTGCTGCGCGATGCCGACATCAAGGGT
FDC217          GATGGACGGCTCGACCGCGAGGACGTGCTCGGCCTGCTGCGCGATGCCGACATCAAGGGT
FDC1083         GATGGACGGCTCGACCGCGAGGACGTGCTCGGCCTGCTGCGCGATGCCGACATCAAGGGT
JF90-8          GATGGACGGCTCGACCGCGAGGACGTGCTCGGCCTGCTGCGCGATGCCGACATCAAGGGT
JJ10-1          GATGGACGGCTCGACCGCGAGGACGTGCTCGGCCTGCTGCGCGATGCCGACATCAAGGGT
JJ238-10        GATGGACGGCTCGACCGCGAGGACGTGCTCGGCCTGCTGCGCGATGCCGACATCAAGGGT
JJ238-24        GATGGACGGCTCGACCGCGAGGACGTGCTCGGCCTGCTGCGCGATGCCGACATCAAGGGT
JK2-10          GATGGACGGCTCGACCGCGAGGACGTGCTCGGCCTGCTGCGCGATGCCGACATCAAGGGT
JK4-1           GATGGACGGCTCGACCGCGAGGACGTGCTCGGCCTGCTGCGCGATGCCGACATCAAGGGT
JK143-11        GATGGACGGCTCGACCGCGAGGACGTGCTCGGCCTGCTGCGCGATGCCGACATCAAGGGT
JS581           GATGGACGGCTCGACCGCGAGGACGTGCTCGGCCTGCTGCGCGATGCCGACATCAAGGGT
JS584           GATGGACGGCTCGACCGCGAGGACGTGCTCGGCCTGCTGCGCGATGCCGACATCAAGGGT
JW160-1         GATGGACGGCTCGACCGCGAGGACGTGCTCGGCCTGCTGCGCGATGCCGACATCAAGGGT
LB100-1         GATGGACGGCTCGACCGCGAGGACGTGCTCGGCCTGCTGCGCGATGCCGACATCAAGGGT
LC80            GATGGACGGCTCGACCGCGAGGACGTGCTCGGCCTGCTGCGCGATGCCGACATCAAGGGT
LD71a           GATGGACGGCTCGACCGCGAGGACGTGCTCGGCCTGCTGCGCGATGCCGACATCAAGGGT
LE20-1          GATGGACGGCTCGACCGCGAGGACGTGCTCGGCCTGCTGCGCGATGCCGACATCAAGGGT
LG115           GATGGACGGCTCGACCGCGAGGACGTGCTCGGCCTGCTGCGCGATGCCGACATCAAGGGT
LG117           GATGGACGGCTCGACCGCGAGGACGTGCTCGGCCTGCTGCGCGATGCCGACATCAAGGGT
LH37-1          GATGGACGGCTCGACCGCGAGGACGTGCTCGGCCTGCTGCGCGATGCCGACATCAAGGGT
LMG9322         GATGGACGGCTCGACCGCGAGGACGTGCTCGGCCTGCTGCGCGATGCCGACATCAAGGGT
NCPPB3562       GATGGACGGCTCGACCGCGAGGACGTGCTCGGCCTGCTGCGCGATGCCGACATCAAGGGT
NCPPB3607       GATGGACGGCTCGACCGCGAGGACGTGCTCGGCCTGCTGCGCGATGCCGACATCAAGGGT
NCPPB3608       GATGGACGGCTCGACCGCGAGGACGTGCTCGGCCTGCTGCGCGATGCCGACATCAAGGGT
X2003-3218      GATGGACGGCTCGACCGCGAGGACGTGCTCGGCCTGCTGCGCGATGCCGACATCAAGGGT
JF90-2          GATGGACGGCTCGACCGCGAGGACGTGCTCGGCCTGCTGCGCGATGCCGACATCAAGGGT
JM35            GATGGACGGCTCGACCGCGAGGACGTGCTCGGCCTGCTGCGCGATGCCGACATCAAGGGT
                ************************************************************
```

```
LG97            TTGCCGCTGGACACCCTGGCGCGGAAATTCAAGATGATCCAGACCACCCTGCGGCCGCTG
NCPPB3612       TTGCCGCTGGACACCCTGGCGCGGAAATTCAAGATGATCCAGACCACCCTGCGGCCGCTG
C40             TTGCCGCTGGACACCCTGGCGCGGAAATTCAAGATGATCCAGACCACCCTGCGGCCGCTG
CFBP2852        TTGCCGCTGGACACCCTGGCGCGGAAATTCAAGATGATCCAGACCACCCTGCGGCCGCTG
CFBP2911        TTGCCGCTGGACACCCTGGCGCGGAAATTCAAGATGATCCAGACCACCCTGCGGCCGCTG
FDC217          TTGCCGCTGGACACCCTGGCGCGGAAATTCAAGATGATCCAGACCACCCTGCGGCCGCTG
FDC1083         TTGCCGCTGGACACCCTGGCGCGGAAATTCAAGATGATCCAGACCACCCTGCGGCCGCTG
JF90-8          TTGCCGCTGGACACCCTGGCGCGGAAATTCAAGATGATCCAGACCACCCTGCGGCCGCTG
JJ10-1          TTGCCGCTGGACACCCTGGCGCGGAAATTCAAGATGATCCAGACCACCCTGCGGCCGCTG
JJ238-10        TTGCCGCTGGACACCCTGGCGCGGAAATTCAAGATGATCCAGACCACCCTGCGGCCGCTG
JJ238-24        TTGCCGCTGGACACCCTGGCGCGGAAATTCAAGATGATCCAGACCACCCTGCGGCCGCTG
JK2-10          TTGCCGCTGGACACCCTGGCGCGGAAATTCAAGATGATCCAGACCACCCTGCGGCCGCTG
JK4-1           TTGCCGCTGGACACCCTGGCGCGGAAATTCAAGATGATCCAGACCACCCTGCGGCCGCTG
JK143-11        TTGCCGCTGGACACCCTGGCGCGGAAATTCAAGATGATCCAGACCACCCTGCGGCCGCTG
JS581           TTGCCGCTGGACACCCTGGCGCGGAAATTCAAGATGATCCAGACCACCCTGCGGCCGCTG
JS584           TTGCCGCTGGACACCCTGGCGCGGAAATTCAAGATGATCCAGACCACCCTGCGGCCGCTG
JW160-1         TTGCCGCTGGACACCCTGGCGCGGAAATTCAAGATGATCCAGACCACCCTGCGGCCGCTG
LB100-1         TTGCCGCTGGACACCCTGGCGCGGAAATTCAAGATGATCCAGACCACCCTGCGGCCGCTG
LC80            TTGCCGCTGGACACCCTGGCGCGGAAATTCAAGATGATCCAGACCACCCTGCGGCCGCTG
LD71a           TTGCCGCTGGACACCCTGGCGCGGAAATTCAAGATGATCCAGACCACCCTGCGGCCGCTG
LE20-1          TTGCCGCTGGACACCCTGGCGCGGAAATTCAAGATGATCCAGACCACCCTGCGGCCGCTG
LG115           TTGCCGCTGGACACCCTGGCGCGGAAATTCAAGATGATCCAGACCACCCTGCGGCCGCTG
LG117           TTGCCGCTGGACACCCTGGCGCGGAAATTCAAGATGATCCAGACCACCCTGCGGCCGCTG
LH37-1          TTGCCGCTGGACACCCTGGCGCGGAAATTCAAGATGATCCAGACCACCCTGCGGCCGCTG
LMG9322         TTGCCGCTGGACACCCTGGCGCGGAAATTCAAGATGATCCAGACCACCCTGCGGCCGCTG
NCPPB3562       TTGCCGCTGGACACCCTGGCGCGGAAATTCAAGATGATCCAGACCACCCTGCGGCCGCTG
NCPPB3607       TTGCCGCTGGACACCCTGGCGCGGAAATTCAAGATGATCCAGACCACCCTGCGGCCGCTG
NCPPB3608       TTGCCGCTGGACACCCTGGCGCGGAAATTCAAGATGATCCAGACCACCCTGCGGCCGCTG
X2003-3218      TTGCCGCTGGACACCCTGGCGCGGAAATTCAAGATGATCCAGACCACCCTGCGGCCGCTG
JF90-2          TTGCCGCTGGACACCCTGGCGCGGAAATTCAAGATGATCCAGACCACCCTGCGGCCGCTG
JM35            TTGCCGCTGGACACCCTGGCGCGGAAATTCAAGATGATCCAGACCACCCTGCGGCCGCTG
                ************************************************************

LG97            ATCGTACCGTACGCCCCCTACGCCGAAGGCTGCGACAAAGAGGTGCCCGAGGTGGCCGAC
NCPPB3612       ATCGTACCGTACGCCCCCTACGCCGAAGGCTGCGACAAAGAGGTGCCCGAGGTGGCCGAC
C40             ATCGTACCGTACGCCCCCTACGCCGAAGGCTGCGACAAAGAGGTGCCCGAGGTGGCCGAC
CFBP2852        ATCGTACCGTACGCCCCCTACGCCGAAGGCTGCGACAAAGAGGTGCCCGAGGTGGCCGAC
CFBP2911        ATCGTACCGTACGCCCCCTACGCCGAAGGCTGCGACAAAGAGGTGCCCGAGGTGGCCGAC
FDC217          ATCGTACCGTACGCCCCCTACGCCGAAGGCTGCGACAAAGAGGTGCCCGAGGTGGCCGAC
FDC1083         ATCGTACCGTACGCCCCCTACGCCGAAGGCTGCGACAAAGAGGTGCCCGAGGTGGCCGAC
JF90-8          ATCGTACCGTACGCCCCCTACGCCGAAGGCTGCGACAAAGAGGTGCCCGAGGTGGCCGAC
JJ10-1          ATCGTACCGTACGCCCCCTACGCCGAAGGCTGCGACAAAGAGGTGCCCGAGGTGGCCGAC
JJ238-10        ATCGTACCGTACGCCCCCTACGCCGAAGGCTGCGACAAAGAGGTGCCCGAGGTGGCCGAC
JJ238-24        ATCGTACCGTACGCCCCCTACGCCGAAGGCTGCGACAAAGAGGTGCCCGAGGTGGCCGAC
JK2-10          ATCGTACCGTACGCCCCCTACGCCGAAGGCTGCGACAAAGAGGTGCCCGAGGTGGCCGAC
JK4-1           ATCGTACCGTACGCCCCCTACGCCGAAGGCTGCGACAAAGAGGTGCCCGAGGTGGCCGAC
JK143-11        ATCGTACCGTACGCCCCCTACGCCGAAGGCTGCGACAAAGAGGTGCCCGAGGTGGCCGAC
JS581           ATCGTACCGTACGCCCCCTACGCCGAAGGCTGCGACAAAGAGGTGCCCGAGGTGGCCGAC
JS584           ATCGTACCGTACGCCCCCTACGCCGAAGGCTGCGACAAAGAGGTGCCCGAGGTGGCCGAC
JW160-1         ATCGTACCGTACGCCCCCTACGCCGAAGGCTGCGACAAAGAGGTGCCCGAGGTGGCCGAC
LB100-1         ATCGTACCGTACGCCCCCTACGCCGAAGGCTGCGACAAAGAGGTGCCCGAGGTGGCCGAC
LC80            ATCGTACCGTACGCCCCCTACGCCGAAGGCTGCGACAAAGAGGTGCCCGAGGTGGCCGAC
LD71a           ATCGTACCGTACGCCCCCTACGCCGAAGGCTGCGACAAAGAGGTGCCCGAGGTGGCCGAC
LE20-1          ATCGTACCGTACGCCCCCTACGCCGAAGGCTGCGACAAAGAGGTGCCCGAGGTGGCCGAC
LG115           ATCGTACCGTACGCCCCCTACGCCGAAGGCTGCGACAAAGAGGTGCCCGAGGTGGCCGAC
LG117           ATCGTACCGTACGCCCCCTACGCCGAAGGCTGCGACAAAGAGGTGCCCGAGGTGGCCGAC
LH37-1          ATCGTACCGTACGCCCCCTACGCCGAAGGCTGCGACAAAGAGGTGCCCGAGGTGGCCGAC
LMG9322         ATCGTACCGTACGCCCCCTACGCCGAAGGCTGCGACAAAGAGGTGCCCGAGGTGGCCGAC
NCPPB3562       ATCGTACCGTACGCCCCCTACGCCGAAGGCTGCGACAAAGAGGTGCCCGAGGTGGCCGAC
NCPPB3607       ATCGTACCGTACGCCCCCTACGCCGAAGGCTGCGACAAAGAGGTGCCCGAGGTGGCCGAC
NCPPB3608       ATCGTACCGTACGCCCCCTACGCCGAAGGCTGCGACAAAGAGGTGCCCGAGGTGGCCGAC
X2003-3218      ATCGTACCGTACGCCCCCTACGCCGAAGGCTGCGACAAAGAGGTGCCCGAGGTGGCCGAC
JF90-2          ATCGTACCGTACGCCCCCTACGCC--------------------GCCCGAGGTGGCCGAC
JM35            ATCGTACCGTACGCCCCCTACGCC--------------------GCCCGAGGTGGCCGAC
                ************************                    ****************
```

```
LG97            ATCCTGCGCCAGCTCGAACATGCAGAACACGTGGGCGTAGCCGGCGCTGCCCGACGCCTG
NCPPB3612       ATCCTGCGCCAGCTCGAACATGCAGAACACGTGGGCGTAGCCGGCGCTGCCCGACGCCTG
C40             ATCCTGCGCCAGCTCGAACATGCAGAACACGTGGGCGTAGCCGGCGCTGCCCGACGCCTG
CFBP2852        ATCCTGCGCCAGCTCGAACATGCAGAACACGTGGGCGTAGCCGGCGCTGCCCGACGCCTG
CFBP2911        ATCCTGCGCCAGCTCGAACATGCAGAACACGTGGGCGTAGCCGGCGCTGCCCGACGCCTG
FDC217          ATCCTGCGCCAGCTCGAACATGCAGAACACGTGGGCGTAGCCGGCGCTGCCCGACGCCTG
FDC1083         ATCCTGCGCCAGCTCGAACATGCAGAACACGTGGGCGTAGCCGGCGCTGCCCGACGCCTG
JF90-8          ATCCTGCGCCAGCTCGAACATGCAGAACACGTGGGCGTAGCCGGCGCTGCCCGACGCCTG
JJ10-1          ATCCTGCGCCAGCTCGAACATGCAGAACACGTGGGCGTAGCCGGCGCTGCCCGACGCCTG
JJ238-10        ATCCTGCGCCAGCTCGAACATGCAGAACACGTGGGCGTAGCCGGCGCTGCCCGACGCCTG
JJ238-24        ATCCTGCGCCAGCTCGAACATGCAGAACACGTGGGCGTAGCCGGCGCTGCCCGACGCCTG
JK2-10          ATCCTGCGCCAGCTCGAACATGCAGAACACGTGGGCGTAGCCGGCGCTGCCCGACGCCTG
JK4-1           ATCCTGCGCCAGCTCGAACATGCAGAACACGTGGGCGTAGCCGGCGCTGCCCGACGCCTG
JK143-11        ATCCTGCGCCAGCTCGAACATGCAGAACACGTGGGCGTAGCCGGCGCTGCCCGACGCCTG
JS581           ATCCTGCGCCAGCTCGAACATGCAGAACACGTGGGCGTAGCCGGCGCTGCCCGACGCCTG
JS584           ATCCTGCGCCAGCTCGAACATGCAGAACACGTGGGCGTAGCCGGCGCTGCCCGACGCCTG
JW160-1         ATCCTGCGCCAGCTCGAACATGCAGAACACGTGGGCGTAGCCGGCGCTGCCCGACGCCTG
LB100-1         ATCCTGCGCCAGCTCGAACATGCAGAACACGTGGGCGTAGCCGGCGCTGCCCGACGCCTG
LC80            ATCCTGCGCCAGCTCGAACATGCAGAACACGTGGGCGTAGCCGGCGCTGCCCGACGCCTG
LD71a           ATCCTGCGCCAGCTCGAACATGCAGAACACGTGGGCGTAGCCGGCGCTGCCCGACGCCTG
LE20-1          ATCCTGCGCCAGCTCGAACATGCAGAACACGTGGGCGTAGCCGGCGCTGCCCGACGCCTG
LG115           ATCCTGCGCCAGCTCGAACATGCAGAACACGTGGGCGTAGCCGGCGCTGCCCGACGCCTG
LG117           ATCCTGCGCCAGCTCGAACATGCAGAACACGTGGGCGTAGCCGGCGCTGCCCGACGCCTG
LH37-1          ATCCTGCGCCAGCTCGAACATGCAGAACACGTGGGCGTAGCCGGCGCTGCCCGACGCCTG
LMG9322         ATCCTGCGCCAGCTCGAACATGCAGAACACGTGGGCGTAGCCGGCGCTGCCCGACGCCTG
NCPPB3562       ATCCTGCGCCAGCTCGAACATGCAGAACACGTGGGCGTAGCCGGCGCTGCCCGACGCCTG
NCPPB3607       ATCCTGCGCCAGCTCGAACATGCAGAACACGTGGGCGTAGCCGGCGCTGCCCGACGCCTG
NCPPB3608       ATCCTGCGCCAGCTCGAACATGCAGAACACGTGGGCGTAGCCGGCGCTGCCCGACGCCTG
X2003-3218      ATCCTGCGCCAGCTCGAACATGCAGAACACGTGGGCGTAGCCGGCGCTGCCCGACGCCTG
JF90-2          ATCCTGCGCCAGCTCGAACATGCAGAACACGTGGGCGTAGCCGGCGCTGCCCGACGCCTG
JM35            ATCCTGCGCCAGCTCGAACATGCAGAACACGTGGGCGTAGCCGGCGCTGCCCGACGCCTG
                ************************************************************

LG97            CAGCCGTGGCTGGTGCAGGTGCCCGAGCAGGCCTACCGCGCGCTGTGGCAGGCCAATGCC
NCPPB3612       CAGCCGTGGCTGGTGCAGGTGCCCGAGCAGGCCTACCGCGCGCTGTGGCAGGCCAATGCC
C40             CAGCCGTGGCTGGTGCAGGTGCCCGAGCAGGCCTACCGCGCGCTGTGGCAGGCCAATGCC
CFBP2852        CAGCCGTGGCTGGTGCAGGTGCCCGAGCAGGCCTACCGCGCGCTGTGGCAGGCCAATGCC
CFBP2911        CAGCCGTGGCTGGTGCAGGTGCCCGAGCAGGCCTACCGCGCGCTGTGGCAGGCCAATGCC
FDC217          CAGCCGTGGCTGGTGCAGGTGCCCGAGCAGGCCTACCGCGCGCTGTGGCAGGCCAATGCC
FDC1083         CAGCCGTGGCTGGTGCAGGTGCCCGAGCAGGCCTACCGCGCGCTGTGGCAGGCCAATGCC
JF90-8          CAGCCGTGGCTGGTGCAGGTGCCCGAGCAGGCCTACCGCGCGCTGTGGCAGGCCAATGCC
JJ10-1          CAGCCGTGGCTGGTGCAGGTGCCCGAGCAGGCCTACCGCGCGCTGTGGCAGGCCAATGCC
JJ238-10        CAGCCGTGGCTGGTGCAGGTGCCCGAGCAGGCCTACCGCGCGCTGTGGCAGGCCAATGCC
JJ238-24        CAGCCGTGGCTGGTGCAGGTGCCCGAGCAGGCCTACCGCGCGCTGTGGCAGGCCAATGCC
JK2-10          CAGCCGTGGCTGGTGCAGGTGCCCGAGCAGGCCTACCGCGCGCTGTGGCAGGCCAATGCC
JK4-1           CAGCCGTGGCTGGTGCAGGTGCCCGAGCAGGCCTACCGCGCGCTGTGGCAGGCCAATGCC
JK143-11        CAGCCGTGGCTGGTGCAGGTGCCCGAGCAGGCCTACCGCGCGCTGTGGCAGGCCAATGCC
JS581           CAGCCGTGGCTGGTGCAGGTGCCCGAGCAGGCCTACCGCGCGCTGTGGCAGGCCAATGCC
JS584           CAGCCGTGGCTGGTGCAGGTGCCCGAGCAGGCCTACCGCGCGCTGTGGCAGGCCAATGCC
JW160-1         CAGCCGTGGCTGGTGCAGGTGCCCGAGCAGGCCTACCGCGCGCTGTGGCAGGCCAATGCC
LB100-1         CAGCCGTGGCTGGTGCAGGTGCCCGAGCAGGCCTACCGCGCGCTGTGGCAGGCCAATGCC
LC80            CAGCCGTGGCTGGTGCAGGTGCCCGAGCAGGCCTACCGCGCGCTGTGGCAGGCCAATGCC
LD71a           CAGCCGTGGCTGGTGCAGGTGCCCGAGCAGGCCTACCGCGCGCTGTGGCAGGCCAATGCC
LE20-1          CAGCCGTGGCTGGTGCAGGTGCCCGAGCAGGCCTACCGCGCGCTGTGGCAGGCCAATGCC
LG115           CAGCCGTGGCTGGTGCAGGTGCCCGAGCAGGCCTACCGCGCGCTGTGGCAGGCCAATGCC
LG117           CAGCCGTGGCTGGTGCAGGTGCCCGAGCAGGCCTACCGCGCGCTGTGGCAGGCCAATGCC
LH37-1          CAGCCGTGGCTGGTGCAGGTGCCCGAGCAGGCCTACCGCGCGCTGTGGCAGGCCAATGCC
LMG9322         CAGCCGTGGCTGGTGCAGGTGCCCGAGCAGGCCTACCGCGCGCTGTGGCAGGCCAATGCC
NCPPB3562       CAGCCGTGGCTGGTGCAGGTGCCCGAGCAGGCCTACCGCGCGCTGTGGCAGGCCAATGCC
NCPPB3607       CAGCCGTGGCTGGTGCAGGTGCCCGAGCAGGCCTACCGCGCGCTGTGGCAGGCCAATGCC
NCPPB3608       CAGCCGTGGCTGGTGCAGGTGCCCGAGCAGGCCTACCGCGCGCTGTGGCAGGCCAATGCC
X2003-3218      CAGCCGTGGCTGGTGCAGGTGCCCGAGCAGGCCTACCGCGCGCTGTGGCAGGCCAATGCC
JF90-2          CAGCCGTGGCTGGTGCAGGTGCCCGAGCAGGCCTACCGCGCGCTGTGGCAGGCCAATGCC
JM35            CAGCCGTGGCTGGTGCAGGTGCCCGAGCAGGCCTACCGCGCGCTGTGGCAGGCCAATGCC
                ************************************************************
```

```
LG97            ATCGCCCCGGTGGCACAGGCGCGCTATGGCGAACAGTTCGTGAGGCTGGTCAATCCAAGA
NCPPB3612       ATCGCCCCGGTGGCACAGGCGCGCTATGGCGAACAGTTCGTGAGGCTGGTCAATCCAAGA
C40             ATCGCCCCGGTGGCACAGGCGCGCTATGGCGAACAGTTCGTGAGGCTGGTCAATCCAAGA
CFBP2852        ATCGCCCCGGTGGCACAGGCGCGCTATGGCGAACAGTTCGTGAGGCTGGTCAATCCAAGA
CFBP2911        ATCGCCCCGGTGGCACAGGCGCGCTATGGCGAACAGTTCGTGAGGCTGGTCAATCCAAGA
FDC217          ATCGCCCCGGTGGCACAGGCGCGCTATGGCGAACAGTTCGTGAGGCTGGTCAATCCAAGA
FDC1083         ATCGCCCCGGTGGCACAGGCGCGCTATGGCGAACAGTTCGTGAGGCTGGTCAATCCAAGA
JF90-8          ATCGCCCCGGTGGCACAGGCGCGCTATGGCGAACAGTTCGTGAGGCTGGTCAATCCAAGA
JJ10-1          ATCGCCCCGGTGGCACAGGCGCGCTATGGCGAACAGTTCGTGAGGCTGGTCAATCCAAGA
JJ238-10        ATCGCCCCGGTGGCACAGGCGCGCTATGGCGAACAGTTCGTGAGGCTGGTCAATCCAAGA
JJ238-24        ATCGCCCCGGTGGCACAGGCGCGCTATGGCGAACAGTTCGTGAGGCTGGTCAATCCAAGA
JK2-10          ATCGCCCCGGTGGCACAGGCGCGCTATGGCGAACAGTTCGTGAGGCTGGTCAATCCAAGA
JK4-1           ATCGCCCCGGTGGCACAGGCGCGCTATGGCGAACAGTTCGTGAGGCTGGTCAATCCAAGA
JK143-11        ATCGCCCCGGTGGCACAGGCGCGCTATGGCGAACAGTTCGTGAGGCTGGTCAATCCAAGA
JS581           ATCGCCCCGGTGGCACAGGCGCGCTATGGCGAACAGTTCGTGAGGCTGGTCAATCCAAGA
JS584           ATCGCCCCGGTGGCACAGGCGCGCTATGGCGAACAGTTCGTGAGGCTGGTCAATCCAAGA
JW160-1         ATCGCCCCGGTGGCACAGGCGCGCTATGGCGAACAGTTCGTGAGGCTGGTCAATCCAAGA
LB100-1         ATCGCCCCGGTGGCACAGGCGCGCTATGGCGAACAGTTCGTGAGGCTGGTCAATCCAAGA
LC80            ATCGCCCCGGTGGCACAGGCGCGCTATGGCGAACAGTTCGTGAGGCTGGTCAATCCAAGA
LD71a           ATCGCCCCGGTGGCACAGGCGCGCTATGGCGAACAGTTCGTGAGGCTGGTCAATCCAAGA
LE20-1          ATCGCCCCGGTGGCACAGGCGCGCTATGGCGAACAGTTCGTGAGGCTGGTCAATCCAAGA
LG115           ATCGCCCCGGTGGCACAGGCGCGCTATGGCGAACAGTTCGTGAGGCTGGTCAATCCAAGA
LG117           ATCGCCCCGGTGGCACAGGCGCGCTATGGCGAACAGTTCGTGAGGCTGGTCAATCCAAGA
LH37-1          ATCGCCCCGGTGGCACAGGCGCGCTATGGCGAACAGTTCGTGAGGCTGGTCAATCCAAGA
LMG9322         ATCGCCCCGGTGGCACAGGCGCGCTATGGCGAACAGTTCGTGAGGCTGGTCAATCCAAGA
NCPPB3562       ATCGCCCCGGTGGCACAGGCGCGCTATGGCGAACAGTTCGTGAGGCTGGTCAATCCAAGA
NCPPB3607       ATCGCCCCGGTGGCACAGGCGCGCTATGGCGAACAGTTCGTGAGGCTGGTCAATCCAAGA
NCPPB3608       ATCGCCCCGGTGGCACAGGCGCGCTATGGCGAACAGTTCGTGAGGCTGGTCAATCCAAGA
X2003-3218      ATCGCCCCGGTGGCACAGGCGCGCTATGGCGAACAGTTCGTGAGGCTGGTCAATCCAAGA
JF90-2          ATCGCCCCGGTGGCACAGGCGCGCTATGGCGAACAGTTCGTGAGGCTGGTCAATCCAAGA
JM35            ATCGCCCCGGTGGCACAGGCGCGCTATGGCGAACAGTTCGTGAGGCTGGTCAATCCAAGA
                ************************************************************

LG97            CTGTACGACCCGCGATTCGGGCTGCGCTGGGACGACAGCCCGCAGTTCCTGGCAGCCGAG
NCPPB3612       CTGTACGACCCGCGATTCGGGCTGCGCTGGGACGACAGCCCGCAGTTCCTGGCAGCCGAG
C40             CTGTACGACCCGCGATTCGGGCTGCGCTGGGACGACAGCCCGCAGTTCCTGGCAGCCGAG
CFBP2852        CTGTACGACCCGCGATTCGGGCTGCGCTGGGACGACAGCCCGCAGTTCCTGGCAGCCGAG
CFBP2911        CTGTACGACCCGCGATTCGGGCTGCGCTGGGACGACAGCCCGCAGTTCCTGGCAGCCGAG
FDC217          CTGTACGACCCGCGATTCGGGCTGCGCTGGGACGACAGCCCGCAGTTCCTGGCAGCCGAG
FDC1083         CTGTACGACCCGCGATTCGGGCTGCGCTGGGACGACAGCCCGCAGTTCCTGGCAGCCGAG
JF90-8          CTGTACGACCCGCGATTCGGGCTGCGCTGGGACGACAGCCCGCAGTTCCTGGCAGCCGAG
JJ10-1          CTGTACGACCCGCGATTCGGGCTGCGCTGGGACGACAGCCCGCAGTTCCTGGCAGCCGAG
JJ238-10        CTGTACGACCCGCGATTCGGGCTGCGCTGGGACGACAGCCCGCAGTTCCTGGCAGCCGAG
JJ238-24        CTGTACGACCCGCGATTCGGGCTGCGCTGGGACGACAGCCCGCAGTTCCTGGCAGCCGAG
JK2-10          CTGTACGACCCGCGATTCGGGCTGCGCTGGGACGACAGCCCGCAGTTCCTGGCAGCCGAG
JK4-1           CTGTACGACCCGCGATTCGGGCTGCGCTGGGACGACAGCCCGCAGTTCCTGGCAGCCGAG
JK143-11        CTGTACGACCCGCGATTCGGGCTGCGCTGGGACGACAGCCCGCAGTTCCTGGCAGCCGAG
JS581           CTGTACGACCCGCGATTCGGGCTGCGCTGGGACGACAGCCCGCAGTTCCTGGCAGCCGAG
JS584           CTGTACGACCCGCGATTCGGGCTGCGCTGGGACGACAGCCCGCAGTTCCTGGCAGCCGAG
JW160-1         CTGTACGACCCGCGATTCGGGCTGCGCTGGGACGACAGCCCGCAGTTCCTGGCAGCCGAG
LB100-1         CTGTACGACCCGCGATTCGGGCTGCGCTGGGACGACAGCCCGCAGTTCCTGGCAGCCGAG
LC80            CTGTACGACCCGCGATTCGGGCTGCGCTGGGACGACAGCCCGCAGTTCCTGGCAGCCGAG
LD71a           CTGTACGACCCGCGATTCGGGCTGCGCTGGGACGACAGCCCGCAGTTCCTGGCAGCCGAG
LE20-1          CTGTACGACCCGCGATTCGGGCTGCGCTGGGACGACAGCCCGCAGTTCCTGGCAGCCGAG
LG115           CTGTACGACCCGCGATTCGGGCTGCGCTGGGACGACAGCCCGCAGTTCCTGGCAGCCGAG
LG117           CTGTACGACCCGCGATTCGGGCTGCGCTGGGACGACAGCCCGCAGTTCCTGGCAGCCGAG
LH37-1          CTGTACGACCCGCGATTCGGGCTGCGCTGGGACGACAGCCCGCAGTTCCTGGCAGCCGAG
LMG9322         CTGTACGACCCGCGATTCGGGCTGCGCTGGGACGACAGCCCGCAGTTCCTGGCAGCCGAG
NCPPB3562       CTGTACGACCCGCGATTCGGGCTGCGCTGGGACGACAGCCCGCAGTTCCTGGCAGCCGAG
NCPPB3607       CTGTACGACCCGCGATTCGGGCTGCGCTGGGACGACAGCCCGCAGTTCCTGGCAGCCGAG
NCPPB3608       CTGTACGACCCGCGATTCGGGCTGCGCTGGGACGACAGCCCGCAGTTCCTGGCAGCCGAG
X2003-3218      CTGTACGACCCGCGATTCGGGCTGCGCTGGGACGACAGCCCGCAGTTCCTGGCAGCCGAG
JF90-2          CTGTACGACCCGCGATTCGGGCTGCGCTGGGACGACAGCCCGCAGTTCCTGGCAGCCGAG
JM35            CTGTACGACCCGCGATTCGGGCTGCGCTGGGACGACAGCCCGCAGTTCCTGGCAGCCGAG
                ************************************************************
```

```
LG97            GCTCTGGTCCAGTGA
NCPPB3612       GCTCTGGTCCAGTGA
C40             GCTCTGGTCCAGTGA
CFBP2852        GCTCTGGTCCAGTGA
CFBP2911        GCTCTGGTCCAGTGA
FDC217          GCTCTGGTCCAGTGA
FDC1083         GCTCTGGTCCAGTGA
JF90-8          GCTCTGGTCCAGTGA
JJ10-1          GCTCTGGTCCAGTGA
JJ238-10        GCTCTGGTCCAGTGA
JJ238-24        GCTCTGGTCCAGTGA
JK2-10          GCTCTGGTCCAGTGA
JK4-1           GCTCTGGTCCAGTGA
JK143-11        GCTCTGGTCCAGTGA
JS581           GCTCTGGTCCAGTGA
JS584           GCTCTGGTCCAGTGA
JW160-1         GCTCTGGTCCAGTGA
LB100-1         GCTCTGGTCCAGTGA
LC80            GCTCTGGTCCAGTGA
LD71a           GCTCTGGTCCAGTGA
LE20-1          GCTCTGGTCCAGTGA
LG115           GCTCTGGTCCAGTGA
LG117           GCTCTGGTCCAGTGA
LH37-1          GCTCTGGTCCAGTGA
LMG9322         GCTCTGGTCCAGTGA
NCPPB3562       GCTCTGGTCCAGTGA
NCPPB3607       GCTCTGGTCCAGTGA
NCPPB3608       GCTCTGGTCCAGTGA
X2003-3218      GCTCTGGTCCAGTGA
JF90-2          GCTCTGGTCCAGTGA
JM35            GCTCTGGTCCAGTGA
                ***************
```

Cas5 (Cascade/processing of the pre-crRNA transcripts)

```
C40             ATGAGTTACGGAGTGAAGCTCCACGTCTGGGGCGAACGAGCGCTGTTCACGCGGCCGGAA
CFBP2852        ATGAGTTACGGAGTGAAGCTCCACGTCTGGGGCGAACGAGCGCTGTTCACGCGGCCGGAA
CFBP2911        ATGAGTTACGGAGTGAAGCTCCACGTCTGGGGCGAACGAGCGCTGTTCACGCGGCCGGAA
FDC217          ATGAGTTACGGAGTGAAGCTCCACGTCTGGGGCGAACGAGCGCTGTTCACGCGGCCGGAA
FDC1083         ATGAGTTACGGAGTGAAGCTCCACGTCTGGGGCGAACGAGCGCTGTTCACGCGGCCGGAA
JF90-2          ATGAGTTACGGAGTGAAGCTCCACGTCTGGGGCGAACGAGCGCTGTTCACGCGGCCGGAA
JF90-8          ATGAGTTACGGAGTGAAGCTCCACGTCTGGGGCGAACGAGCGCTGTTCACGCGGCCGGAA
JJ10-1          ATGAGTTACGGAGTGAAGCTCCACGTCTGGGGCGAACGAGCGCTGTTCACGCGGCCGGAA
JJ238-10        ATGAGTTACGGAGTGAAGCTCCACGTCTGGGGCGAACGAGCGCTGTTCACGCGGCCGGAA
JJ238-24        ATGAGTTACGGAGTGAAGCTCCACGTCTGGGGCGAACGAGCGCTGTTCACGCGGCCGGAA
JK2-10          ATGAGTTACGGAGTGAAGCTCCACGTCTGGGGCGAACGAGCGCTGTTCACGCGGCCGGAA
JK4-1           ATGAGTTACGGAGTGAAGCTCCACGTCTGGGGCGAACGAGCGCTGTTCACGCGGCCGGAA
JK48            ATGAGTTACGGAGTGAAGCTCCACGTCTGGGGCGAACGAGCGCTGTTCACGCGGCCGGAA
JK143-9         ATGAGTTACGGAGTGAAGCTCCACGTCTGGGGCGAACGAGCGCTGTTCACGCGGCCGGAA
JK143-11        ATGAGTTACGGAGTGAAGCTCCACGTCTGGGGCGAACGAGCGCTGTTCACGCGGCCGGAA
JM35            ATGAGTTACGGAGTGAAGCTCCACGTCTGGGGCGAACGAGCGCTGTTCACGCGGCCGGAA
JS581           ATGAGTTACGGAGTGAAGCTCCACGTCTGGGGCGAACGAGCGCTGTTCACGCGGCCGGAA
JS584           ATGAGTTACGGAGTGAAGCTCCACGTCTGGGGCGAACGAGCGCTGTTCACGCGGCCGGAA
JW160-1         ATGAGTTACGGAGTGAAGCTCCACGTCTGGGGCGAACGAGCGCTGTTCACGCGGCCGGAA
LB100-1         ATGAGTTACGGAGTGAAGCTCCACGTCTGGGGCGAACGAGCGCTGTTCACGCGGCCGGAA
LC80            ATGAGTTACGGAGTGAAGCTCCACGTCTGGGGCGAACGAGCGCTGTTCACGCGGCCGGAA
LD7-1           ATGAGTTACGGAGTGAAGCTCCACGTCTGGGGCGAACGAGCGCTGTTCACGCGGCCGGAA
LD71a           ATGAGTTACGGAGTGAAGCTCCACGTCTGGGGCGAACGAGCGCTGTTCACGCGGCCGGAA
LE3-1           ATGAGTTACGGAGTGAAGCTCCACGTCTGGGGCGAACGAGCGCTGTTCACGCGGCCGGAA
LE20-1          ATGAGTTACGGAGTGAAGCTCCACGTCTGGGGCGAACGAGCGCTGTTCACGCGGCCGGAA
LG102           ATGAGTTACGGAGTGAAGCTCCACGTCTGGGGCGAACGAGCGCTGTTCACGCGGCCGGAA
LG117           ATGAGTTACGGAGTGAAGCTCCACGTCTGGGGCGAACGAGCGCTGTTCACGCGGCCGGAA
LH37-1          ATGAGTTACGGAGTGAAGCTCCACGTCTGGGGCGAACGAGCGCTGTTCACGCGGCCGGAA
LMG9322         ATGAGTTACGGAGTGAAGCTCCACGTCTGGGGCGAACGAGCGCTGTTCACGCGGCCGGAA
NCPPB3562       ATGAGTTACGGAGTGAAGCTCCACGTCTGGGGCGAACGAGCGCTGTTCACGCGGCCGGAA
NCPPB3607       ATGAGTTACGGAGTGAAGCTCCACGTCTGGGGCGAACGAGCGCTGTTCACGCGGCCGGAA
NCPPB3612       ATGAGTTACGGAGTGAAGCTCCACGTCTGGGGCGAACGAGCGCTGTTCACGCGGCCGGAA
LB302           ATGAGTTACGGAGTGAAGCTCCACGTCTGGGGCGAACGAGCGCTGTTCACGCGGCCGGAA
LG115           ATGAGTTACGGAGTGAAGCTCCACGTCTGGGGCGAACGAGCGCTGTTCACGCGGCCGGAA
NCPPB3608       ATGAGTTACGGAGTGAAGCTCCACGTCTGGGGCGAACGAGCGCTGTTCACGCGGCCGGAA
X2003-3218      ATGAGTTACGGAGTGAAGCTCCACGTCTGGGGCGAACGAGCGCTGTTCACGCGGCCGGAA
                ************************************************************

C40             ATGAAGGTGGAACGCGTCTCATACGACATCATCACGCCATCGGCGGCGCGCGGCATCCTG
CFBP2852        ATGAAGGTGGAACGCGTCTCATACGACATCATCACGCCATCGGCGGCGCGCGGCATCCTG
CFBP2911        ATGAAGGTGGAACGCGTCTCATACGACATCATCACGCCATCGGCGGCGCGCGGCATCCTG
FDC217          ATGAAGGTGGAACGCGTCTCATACGACATCATCACGCCATCGGCGGCGCGCGGCATCCTG
FDC1083         ATGAAGGTGGAACGCGTCTCATACGACATCATCACGCCATCGGCGGCGCGCGGCATCCTG
JF90-2          ATGAAGGTGGAACGCGTCTCATACGACATCATCACGCCATCGGCGGCGCGCGGCATCCTG
JF90-8          ATGAAGGTGGAACGCGTCTCATACGACATCATCACGCCATCGGCGGCGCGCGGCATCCTG
JJ10-1          ATGAAGGTGGAACGCGTCTCATACGACATCATCACGCCATCGGCGGCGCGCGGCATCCTG
JJ238-10        ATGAAGGTGGAACGCGTCTCATACGACATCATCACGCCATCGGCGGCGCGCGGCATCCTG
JJ238-24        ATGAAGGTGGAACGCGTCTCATACGACATCATCACGCCATCGGCGGCGCGCGGCATCCTG
JK2-10          ATGAAGGTGGAACGCGTCTCATACGACATCATCACGCCATCGGCGGCGCGCGGCATCCTG
JK4-1           ATGAAGGTGGAACGCGTCTCATACGACATCATCACGCCATCGGCGGCGCGCGGCATCCTG
JK48            ATGAAGGTGGAACGCGTCTCATACGACATCATCACGCCATCGGCGGCGCGCGGCATCCTG
JK143-9         ATGAAGGTGGAACGCGTCTCATACGACATCATCACGCCATCGGCGGCGCGCGGCATCCTG
JK143-11        ATGAAGGTGGAACGCGTCTCATACGACATCATCACGCCATCGGCGGCGCGCGGCATCCTG
JM35            ATGAAGGTGGAACGCGTCTCATACGACATCATCACGCCATCGGCGGCGCGCGGCATCCTG
JS581           ATGAAGGTGGAACGCGTCTCATACGACATCATCACGCCATCGGCGGCGCGCGGCATCCTG
JS584           ATGAAGGTGGAACGCGTCTCATACGACATCATCACGCCATCGGCGGCGCGCGGCATCCTG
JW160-1         ATGAAGGTGGAACGCGTCTCATACGACATCATCACGCCATCGGCGGCGCGCGGCATCCTG
LB100-1         ATGAAGGTGGAACGCGTCTCATACGACATCATCACGCCATCGGCGGCGCGCGGCATCCTG
LC80            ATGAAGGTGGAACGCGTCTCATACGACATCATCACGCCATCGGCGGCGCGCGGCATCCTG
LD7-1           ATGAAGGTGGAACGCGTCTCATACGACATCATCACGCCATCGGCGGCGCGCGGCATCCTG
LD71a           ATGAAGGTGGAACGCGTCTCATACGACATCATCACGCCATCGGCGGCGCGCGGCATCCTG
LE3-1           ATGAAGGTGGAACGCGTCTCATACGACATCATCACGCCATCGGCGGCGCGCGGCATCCTG
LE20-1          ATGAAGGTGGAACGCGTCTCATACGACATCATCACGCCATCGGCGGCGCGCGGCATCCTG
LG102           ATGAAGGTGGAACGCGTCTCATACGACATCATCACGCCATCGGCGGCGCGCGGCATCCTG
LG117           ATGAAGGTGGAACGCGTCTCATACGACATCATCACGCCATCGGCGGCGCGCGGCATCCTG
LH37-1          ATGAAGGTGGAACGCGTCTCATACGACATCATCACGCCATCGGCGGCGCGCGGCATCCTG
LMG9322         ATGAAGGTGGAACGCGTCTCATACGACATCATCACGCCATCGGCGGCGCGCGGCATCCTG
NCPPB3562       ATGAAGGTGGAACGCGTCTCATACGACATCATCACGCCATCGGCGGCGCGCGGCATCCTG
NCPPB3607       ATGAAGGTGGAACGCGTCTCATACGACATCATCACGCCATCGGCGGCGCGCGGCATCCTG
NCPPB3612       ATGAAGGTGGAACGCGTCTCATACGACATCATCACGCCATCGGCGGCGCGCGGCATCCTG
LB302           ATGAAGGTGGAACGCGTCTCATACGACATCATCACGCCATCGGCGGCGCGCGGCATCCTG
LG115           ATGAAGGTGGAACGCGTCTCATACGACATCATCACGCCATCGGCGGCGCGCGGCATCCTG
NCPPB3608       ATGAAGGTGGAACGCGTCTCATACGACATCATCACGCCATCGGCGGCGCGCGGCATCCTG
X2003-3218      ATGAAGGTGGAACGCGTCTCATACGACATCATCACGCCATCGGCGGCGCGCGGCATCCTG
                ************************************************************
```

```
C40             GAGGCGATCCACTGGAAACCGGCGATCCGCTGGATGGTGGACAGCATCCAGGTGCTCAAG
CFBP2852        GAGGCGATCCACTGGAAACCGGCGATCCGCTGGATGGTGGACAGCATCCAGGTGCTCAAG
CFBP2911        GAGGCGATCCACTGGAAACCGGCGATCCGCTGGATGGTGGACAGCATCCAGGTGCTCAAG
FDC217          GAGGCGATCCACTGGAAACCGGCGATCCGCTGGATGGTGGACAGCATCCAGGTGCTCAAG
FDC1083         GAGGCGATCCACTGGAAACCGGCGATCCGCTGGATGGTGGACAGCATCCAGGTGCTCAAG
JF90-2          GAGGCGATCCACTGGAAACCGGCGATCCGCTGGATGGTGGACAGCATCCAGGTGCTCAAG
JF90-8          GAGGCGATCCACTGGAAACCGGCGATCCGCTGGATGGTGGACAGCATCCAGGTGCTCAAG
JJ10-1          GAGGCGATCCACTGGAAACCGGCGATCCGCTGGATGGTGGACAGCATCCAGGTGCTCAAG
JJ238-10        GAGGCGATCCACTGGAAACCGGCGATCCGCTGGATGGTGGACAGCATCCAGGTGCTCAAG
JJ238-24        GAGGCGATCCACTGGAAACCGGCGATCCGCTGGATGGTGGACAGCATCCAGGTGCTCAAG
JK2-10          GAGGCGATCCACTGGAAACCGGCGATCCGCTGGATGGTGGACAGCATCCAGGTGCTCAAG
JK4-1           GAGGCGATCCACTGGAAACCGGCGATCCGCTGGATGGTGGACAGCATCCAGGTGCTCAAG
JK48            GAGGCGATCCACTGGAAACCGGCGATCCGCTGGATGGTGGACAGCATCCAGGTGCTCAAG
JK143-9         GAGGCGATCCACTGGAAACCGGCGATCCGCTGGATGGTGGACAGCATCCAGGTGCTCAAG
JK143-11        GAGGCGATCCACTGGAAACCGGCGATCCGCTGGATGGTGGACAGCATCCAGGTGCTCAAG
JM35            GAGGCGATCCACTGGAAACCGGCGATCCGCTGGATGGTGGACAGCATCCAGGTGCTCAAG
JS581           GAGGCGATCCACTGGAAACCGGCGATCCGCTGGATGGTGGACAGCATCCAGGTGCTCAAG
JS584           GAGGCGATCCACTGGAAACCGGCGATCCGCTGGATGGTGGACAGCATCCAGGTGCTCAAG
JW160-1         GAGGCGATCCACTGGAAACCGGCGATCCGCTGGATGGTGGACAGCATCCAGGTGCTCAAG
LB100-1         GAGGCGATCCACTGGAAACCGGCGATCCGCTGGATGGTGGACAGCATCCAGGTGCTCAAG
LC80            GAGGCGATCCACTGGAAACCGGCGATCCGCTGGATGGTGGACAGCATCCAGGTGCTCAAG
LD7-1           GAGGCGATCCACTGGAAACCGGCGATCCGCTGGATGGTGGACAGCATCCAGGTGCTCAAG
LD71a           GAGGCGATCCACTGGAAACCGGCGATCCGCTGGATGGTGGACAGCATCCAGGTGCTCAAG
LE3-1           GAGGCGATCCACTGGAAACCGGCGATCCGCTGGATGGTGGACAGCATCCAGGTGCTCAAG
LE20-1          GAGGCGATCCACTGGAAACCGGCGATCCGCTGGATGGTGGACAGCATCCAGGTGCTCAAG
LG102           GAGGCGATCCACTGGAAACCGGCGATCCGCTGGATGGTGGACAGCATCCAGGTGCTCAAG
LG117           GAGGCGATCCACTGGAAACCGGCGATCCGCTGGATGGTGGACAGCATCCAGGTGCTCAAG
LH37-1          GAGGCGATCCACTGGAAACCGGCGATCCGCTGGATGGTGGACAGCATCCAGGTGCTCAAG
LMG9322         GAGGCGATCCACTGGAAACCGGCGATCCGCTGGATGGTGGACAGCATCCAGGTGCTCAAG
NCPPB3562       GAGGCGATCCACTGGAAACCGGCGATCCGCTGGATGGTGGACAGCATCCAGGTGCTCAAG
NCPPB3607       GAGGCGATCCACTGGAAACCGGCGATCCGCTGGATGGTGGACAGCATCCAGGTGCTCAAG
NCPPB3612       GAGGCGATCCACTGGAAACCGGCGATCCGCTGGATGGTGGACAGCATCCAGGTGCTCAAG
LB302           GAGGCGATCCACTGGAAACCGGCGATCCGCTGGATGGTGGACAGCATCCAGGTGCTCAAG
LG115           GAGGCGATCCACTGGAAACCGGCGATCCGCTGGATGGTGGACAGCATCCAGGTGCTCAAG
NCPPB3608       GAGGCGATCCACTGGAAACCGGCGATCCGCTGGATGGTGGACAGCATCCAGGTGCTCAAG
X2003-3218      GAGGCGATCCACTGGAAACCGGCGATCCGCTGGATGGTGGACAGCATCCAGGTGCTCAAG
                ************************************************************

C40             CCGATTCGCTTCGAGTCGATCCGCCGCAACGAAGTGGGCGGCAAGCTGTCTGCCGCCAGC
CFBP2852        CCGATTCGCTTCGAGTCGATCCGCCGCAACGAAGTGGGCGGCAAGCTGTCTGCCGCCAGC
CFBP2911        CCGATTCGCTTCGAGTCGATCCGCCGCAACGAAGTGGGCGGCAAGCTGTCTGCCGCCAGC
FDC217          CCGATTCGCTTCGAGTCGATCCGCCGCAACGAAGTGGGCGGCAAGCTGTCTGCCGCCAGC
FDC1083         CCGATTCGCTTCGAGTCGATCCGCCGCAACGAAGTGGGCGGCAAGCTGTCTGCCGCCAGC
JF90-2          CCGATTCGCTTCGAGTCGATCCGCCGCAACGAAGTGGGCGGCAAGCTGTCTGCCGCCAGC
JF90-8          CCGATTCGCTTCGAGTCGATCCGCCGCAACGAAGTGGGCGGCAAGCTGTCTGCCGCCAGC
JJ10-1          CCGATTCGCTTCGAGTCGATCCGCCGCAACGAAGTGGGCGGCAAGCTGTCTGCCGCCAGC
JJ238-10        CCGATTCGCTTCGAGTCGATCCGCCGCAACGAAGTGGGCGGCAAGCTGTCTGCCGCCAGC
JJ238-24        CCGATTCGCTTCGAGTCGATCCGCCGCAACGAAGTGGGCGGCAAGCTGTCTGCCGCCAGC
JK2-10          CCGATTCGCTTCGAGTCGATCCGCCGCAACGAAGTGGGCGGCAAGCTGTCTGCCGCCAGC
JK4-1           CCGATTCGCTTCGAGTCGATCCGCCGCAACGAAGTGGGCGGCAAGCTGTCTGCCGCCAGC
JK48            CCGATTCGCTTCGAGTCGATCCGCCGCAACGAAGTGGGCGGCAAGCTGTCTGCCGCCAGC
JK143-9         CCGATTCGCTTCGAGTCGATCCGCCGCAACGAAGTGGGCGGCAAGCTGTCTGCCGCCAGC
JK143-11        CCGATTCGCTTCGAGTCGATCCGCCGCAACGAAGTGGGCGGCAAGCTGTCTGCCGCCAGC
JM35            CCGATTCGCTTCGAGTCGATCCGCCGCAACGAAGTGGGCGGCAAGCTGTCTGCCGCCAGC
JS581           CCGATTCGCTTCGAGTCGATCCGCCGCAACGAAGTGGGCGGCAAGCTGTCTGCCGCCAGC
JS584           CCGATTCGCTTCGAGTCGATCCGCCGCAACGAAGTGGGCGGCAAGCTGTCTGCCGCCAGC
JW160-1         CCGATTCGCTTCGAGTCGATCCGCCGCAACGAAGTGGGCGGCAAGCTGTCTGCCGCCAGC
LB100-1         CCGATTCGCTTCGAGTCGATCCGCCGCAACGAAGTGGGCGGCAAGCTGTCTGCCGCCAGC
LC80            CCGATTCGCTTCGAGTCGATCCGCCGCAACGAAGTGGGCGGCAAGCTGTCTGCCGCCAGC
LD7-1           CCGATTCGCTTCGAGTCGATCCGCCGCAACGAAGTGGGCGGCAAGCTGTCTGCCGCCAGC
LD71a           CCGATTCGCTTCGAGTCGATCCGCCGCAACGAAGTGGGCGGCAAGCTGTCTGCCGCCAGC
LE3-1           CCGATTCGCTTCGAGTCGATCCGCCGCAACGAAGTGGGCGGCAAGCTGTCTGCCGCCAGC
LE20-1          CCGATTCGCTTCGAGTCGATCCGCCGCAACGAAGTGGGCGGCAAGCTGTCTGCCGCCAGC
LG102           CCGATTCGCTTCGAGTCGATCCGCCGCAACGAAGTGGGCGGCAAGCTGTCTGCCGCCAGC
LG117           CCGATTCGCTTCGAGTCGATCCGCCGCAACGAAGTGGGCGGCAAGCTGTCTGCCGCCAGC
LH37-1          CCGATTCGCTTCGAGTCGATCCGCCGCAACGAAGTGGGCGGCAAGCTGTCTGCCGCCAGC
LMG9322         CCGATTCGCTTCGAGTCGATCCGCCGCAACGAAGTGGGCGGCAAGCTGTCTGCCGCCAGC
NCPPB3562       CCGATTCGCTTCGAGTCGATCCGCCGCAACGAAGTGGGCGGCAAGCTGTCTGCCGCCAGC
NCPPB3607       CCGATTCGCTTCGAGTCGATCCGCCGCAACGAAGTGGGCGGCAAGCTGTCTGCCGCCAGC
NCPPB3612       CCGATTCGCTTCGAGTCGATCCGCCGCAACGAAGTGGGCGGCAAGCTGTCTGCCGCCAGC
LB302           CCGATTCGCTTCGAGTCGATCCGCCGCAACGAAGTGGGCGGCAAGCTGTCTGCCGCCAGC
LG115           CCGATTCGCTTCGAGTCGATCCGCCGCAACGAAGTGGGCGGCAAGCTGTCTGCCGCCAGC
NCPPB3608       CCGATTCGCTTCGAGTCGATCCGCCGCAACGAAGTGGGCGGCAAGCTGTCTGCCGCCAGC
X2003-3218      CCGATTCGCTTCGAGTCGATCCGCCGCAACGAAGTGGGCGGCAAGCTGTCTGCCGCCAGC
                ************************************************************
```

```
C40               GTGAGCAAGGCGATCAAGGCCGGGCGCACCGATACGCTGGTGAGCTACGTCGAGGAGGAC
CFBP2852          GTGAGCAAGGCGATCAAGGCCGGGCGCACCGATACGCTGGTGAGCTACGTCGAGGAGGAC
CFBP2911          GTGAGCAAGGCGATCAAGGCCGGGCGCACCGATACGCTGGTGAGCTACGTCGAGGAGGAC
FDC217            GTGAGCAAGGCGATCAAGGCCGGGCGCACCGATACGCTGGTGAGCTACGTCGAGGAGGAC
FDC1083           GTGAGCAAGGCGATCAAGGCCGGGCGCACCGATACGCTGGTGAGCTACGTCGAGGAGGAC
JF90-2            GTGAGCAAGGCGATCAAGGCCGGGCGCACCGATACGCTGGTGAGCTACGTCGAGGAGGAC
JF90-8            GTGAGCAAGGCGATCAAGGCCGGGCGCACCGATACGCTGGTGAGCTACGTCGAGGAGGAC
JJ10-1            GTGAGCAAGGCGATCAAGGCCGGGCGCACCGATACGCTGGTGAGCTACGTCGAGGAGGAC
JJ238-10          GTGAGCAAGGCGATCAAGGCCGGGCGCACCGATACGCTGGTGAGCTACGTCGAGGAGGAC
JJ238-24          GTGAGCAAGGCGATCAAGGCCGGGCGCACCGATACGCTGGTGAGCTACGTCGAGGAGGAC
JK2-10            GTGAGCAAGGCGATCAAGGCCGGGCGCACCGATACGCTGGTGAGCTACGTCGAGGAGGAC
JK4-1             GTGAGCAAGGCGATCAAGGCCGGGCGCACCGATACGCTGGTGAGCTACGTCGAGGAGGAC
JK48              GTGAGCAAGGCGATCAAGGCCGGGCGCACCGATACGCTGGTGAGCTACGTCGAGGAGGAC
JK143-9           GTGAGCAAGGCGATCAAGGCCGGGCGCACCGATACGCTGGTGAGCTACGTCGAGGAGGAC
JK143-11          GTGAGCAAGGCGATCAAGGCCGGGCGCACCGATACGCTGGTGAGCTACGTCGAGGAGGAC
JM35              GTGAGCAAGGCGATCAAGGCCGGGCGCACCGATACGCTGGTGAGCTACGTCGAGGAGGAC
JS581             GTGAGCAAGGCGATCAAGGCCGGGCGCACCGATACGCTGGTGAGCTACGTCGAGGAGGAC
JS584             GTGAGCAAGGCGATCAAGGCCGGGCGCACCGATACGCTGGTGAGCTACGTCGAGGAGGAC
JW160-1           GTGAGCAAGGCGATCAAGGCCGGGCGCACCGATACGCTGGTGAGCTACGTCGAGGAGGAC
LB100-1           GTGAGCAAGGCGATCAAGGCCGGGCGCACCGATACGCTGGTGAGCTACGTCGAGGAGGAC
LC80              GTGAGCAAGGCGATCAAGGCCGGGCGCACCGATACGCTGGTGAGCTACGTCGAGGAGGAC
LD7-1             GTGAGCAAGGCGATCAAGGCCGGGCGCACCGATACGCTGGTGAGCTACGTCGAGGAGGAC
LD71a             GTGAGCAAGGCGATCAAGGCCGGGCGCACCGATACGCTGGTGAGCTACGTCGAGGAGGAC
LE3-1             GTGAGCAAGGCGATCAAGGCCGGGCGCACCGATACGCTGGTGAGCTACGTCGAGGAGGAC
LE20-1            GTGAGCAAGGCGATCAAGGCCGGGCGCACCGATACGCTGGTGAGCTACGTCGAGGAGGAC
LG102             GTGAGCAAGGCGATCAAGGCCGGGCGCACCGATACGCTGGTGAGCTACGTCGAGGAGGAC
LG117             GTGAGCAAGGCGATCAAGGCCGGGCGCACCGATACGCTGGTGAGCTACGTCGAGGAGGAC
LH37-1            GTGAGCAAGGCGATCAAGGCCGGGCGCACCGATACGCTGGTGAGCTACGTCGAGGAGGAC
LMG9322           GTGAGCAAGGCGATCAAGGCCGGGCGCACCGATACGCTGGTGAGCTACGTCGAGGAGGAC
NCPPB3562         GTGAGCAAGGCGATCAAGGCCGGGCGCACCGATACGCTGGTGAGCTACGTCGAGGAGGAC
NCPPB3607         GTGAGCAAGGCGATCAAGGCCGGGCGCACCGATACGCTGGTGAGCTACGTCGAGGAGGAC
NCPPB3612         GTGAGCAAGGCGATCAAGGCCGGGCGCACCGATACGCTGGTGAGCTACGTCGAGGAGGAC
LB302             GTGAGCAAGGCGATCAAGGCCGGGCGCACCGATACGCTGGTGAGCTACGTCGAGGAGGAC
LG115             GTGAGCAAGGCGATCAAGGCCGGGCGCACCGATACGCTGGTGAGCTACGTCGAGGAGGAC
NCPPB3608         GTGAGCAAGGCGATCAAGGCCGGGCGCACCGATACGCTGGTGAGCTACGTCGAGGAGGAC
X2003-3218        GTGAGCAAGGCGATCAAGGCCGGGCGCACCGATACGCTGGTGAGCTACGTCGAGGAGGAC
                  ************************************************************

C40               CGCCAACAACGCGCCGCCACGCTCCTGCGCGACGTGGGTTACGTGATCGCTGCACACTTC
CFBP2852          CGCCAACAACGCGCCGCCACGCTCCTGCGCGACGTGGGTTACGTGATCGCTGCACACTTC
CFBP2911          CGCCAACAACGCGCCGCCACGCTCCTGCGCGACGTGGGTTACGTGATCGCTGCACACTTC
FDC217            CGCCAACAACGCGCCGCCACGCTCCTGCGCGACGTGGGTTACGTGATCGCTGCACACTTC
FDC1083           CGCCAACAACGCGCCGCCACGCTCCTGCGCGACGTGGGTTACGTGATCGCTGCACACTTC
JF90-2            CGCCAACAACGCGCCGCCACGCTCCTGCGCGACGTGGGTTACGTGATCGCTGCACACTTC
JF90-8            CGCCAACAACGCGCCGCCACGCTCCTGCGCGACGTGGGTTACGTGATCGCTGCACACTTC
JJ10-1            CGCCAACAACGCGCCGCCACGCTCCTGCGCGACGTGGGTTACGTGATCGCTGCACACTTC
JJ238-10          CGCCAACAACGCGCCGCCACGCTCCTGCGCGACGTGGGTTACGTGATCGCTGCACACTTC
JJ238-24          CGCCAACAACGCGCCGCCACGCTCCTGCGCGACGTGGGTTACGTGATCGCTGCACACTTC
JK2-10            CGCCAACAACGCGCCGCCACGCTCCTGCGCGACGTGGGTTACGTGATCGCTGCACACTTC
JK4-1             CGCCAACAACGCGCCGCCACGCTCCTGCGCGACGTGGGTTACGTGATCGCTGCACACTTC
JK48              CGCCAACAACGCGCCGCCACGCTCCTGCGCGACGTGGGTTACGTGATCGCTGCACACTTC
JK143-9           CGCCAACAACGCGCCGCCACGCTCCTGCGCGACGTGGGTTACGTGATCGCTGCACACTTC
JK143-11          CGCCAACAACGCGCCGCCACGCTCCTGCGCGACGTGGGTTACGTGATCGCTGCACACTTC
JM35              CGCCAACAACGCGCCGCCACGCTCCTGCGCGACGTGGGTTACGTGATCGCTGCACACTTC
JS581             CGCCAACAACGCGCCGCCACGCTCCTGCGCGACGTGGGTTACGTGATCGCTGCACACTTC
JS584             CGCCAACAACGCGCCGCCACGCTCCTGCGCGACGTGGGTTACGTGATCGCTGCACACTTC
JW160-1           CGCCAACAACGCGCCGCCACGCTCCTGCGCGACGTGGGTTACGTGATCGCTGCACACTTC
LB100-1           CGCCAACAACGCGCCGCCACGCTCCTGCGCGACGTGGGTTACGTGATCGCTGCACACTTC
LC80              CGCCAACAACGCGCCGCCACGCTCCTGCGCGACGTGGGTTACGTGATCGCTGCACACTTC
LD7-1             CGCCAACAACGCGCCGCCACGCTCCTGCGCGACGTGGGTTACGTGATCGCTGCACACTTC
LD71a             CGCCAACAACGCGCCGCCACGCTCCTGCGCGACGTGGGTTACGTGATCGCTGCACACTTC
LE3-1             CGCCAACAACGCGCCGCCACGCTCCTGCGCGACGTGGGTTACGTGATCGCTGCACACTTC
LE20-1            CGCCAACAACGCGCCGCCACGCTCCTGCGCGACGTGGGTTACGTGATCGCTGCACACTTC
LG102             CGCCAACAACGCGCCGCCACGCTCCTGCGCGACGTGGGTTACGTGATCGCTGCACACTTC
LG117             CGCCAACAACGCGCCGCCACGCTCCTGCGCGACGTGGGTTACGTGATCGCTGCACACTTC
LH37-1            CGCCAACAACGCGCCGCCACGCTCCTGCGCGACGTGGGTTACGTGATCGCTGCACACTTC
LMG9322           CGCCAACAACGCGCCGCCACGCTCCTGCGCGACGTGGGTTACGTGATCGCTGCACACTTC
NCPPB3562         CGCCAACAACGCGCCGCCACGCTCCTGCGCGACGTGGGTTACGTGATCGCTGCACACTTC
NCPPB3607         CGCCAACAACGCGCCGCCACGCTCCTGCGCGACGTGGGTTACGTGATCGCTGCACACTTC
NCPPB3612         CGCCAACAACGCGCCGCCACGCTCCTGCGCGACGTGGGTTACGTGATCGCTGCACACTTC
LB302             CGCCAACAACGCGCCGCCACGCTCCTGCGCGACGTGGGTTACGTGATCGCTGCACACTTC
LG115             CGCCAACAACGCGCCGCCACGCTCCTGCGCGACGTGGGTTACGTGATCGCTGCACACTTC
NCPPB3608         CGCCAACAACGCGCCGCCACGCTCCTGCGCGACGTGGGTTACGTGATCGCTGCACACTTC
X2003-3218        CGCCAACAACGCGCCGCCACGCTCCTGCGCGACGTGGGTTACGTGATCGCTGCACACTTC
                  ************************************************************
```

```
C40             GAATTGACCGACAAGGCGGGCGCCGACGACAACGTCGGCAAGCACCTGGACATCTTCAAC
CFBP2852        GAATTGACCGACAAGGCGGGCGCCGACGACAACGTCGGCAAGCACCTGGACATCTTCAAC
CFBP2911        GAATTGACCGACAAGGCGGGCGCCGACGACAACGTCGGCAAGCACCTGGACATCTTCAAC
FDC217          GAATTGACCGACAAGGCGGGCGCCGACGACAACGTCGGCAAGCACCTGGACATCTTCAAC
FDC1083         GAATTGACCGACAAGGCGGGCGCCGACGACAACGTCGGCAAGCACCTGGACATCTTCAAC
JF90-2          GAATTGACCGACAAGGCGGGCGCCGACGACAACGTCGGCAAGCACCTGGACATCTTCAAC
JF90-8          GAATTGACCGACAAGGCGGGCGCCGACGACAACGTCGGCAAGCACCTGGACATCTTCAAC
JJ10-1          GAATTGACCGACAAGGCGGGCGCCGACGACAACGTCGGCAAGCACCTGGACATCTTCAAC
JJ238-10        GAATTGACCGACAAGGCGGGCGCCGACGACAACGTCGGCAAGCACCTGGACATCTTCAAC
JJ238-24        GAATTGACCGACAAGGCGGGCGCCGACGACAACGTCGGCAAGCACCTGGACATCTTCAAC
JK2-10          GAATTGACCGACAAGGCGGGCGCCGACGACAACGTCGGCAAGCACCTGGACATCTTCAAC
JK4-1           GAATTGACCGACAAGGCGGGCGCCGACGACAACGTCGGCAAGCACCTGGACATCTTCAAC
JK48            GAATTGACCGACAAGGCGGGCGCCGACGACAACGTCGGCAAGCACCTGGACATCTTCAAC
JK143-9         GAATTGACCGACAAGGCGGGCGCCGACGACAACGTCGGCAAGCACCTGGACATCTTCAAC
JK143-11        GAATTGACCGACAAGGCGGGCGCCGACGACAACGTCGGCAAGCACCTGGACATCTTCAAC
JM35            GAATTGACCGACAAGGCGGGCGCCGACGACAACGTCGGCAAGCACCTGGACATCTTCAAC
JS581           GAATTGACCGACAAGGCGGGCGCCGACGACAACGTCGGCAAGCACCTGGACATCTTCAAC
JS584           GAATTGACCGACAAGGCGGGCGCCGACGACAACGTCGGCAAGCACCTGGACATCTTCAAC
JW160-1         GAATTGACCGACAAGGCGGGCGCCGACGACAACGTCGGCAAGCACCTGGACATCTTCAAC
LB100-1         GAATTGACCGACAAGGCGGGCGCCGACGACAACGTCGGCAAGCACCTGGACATCTTCAAC
LC80            GAATTGACCGACAAGGCGGGCGCCGACGACAACGTCGGCAAGCACCTGGACATCTTCAAC
LD7-1           GAATTGACCGACAAGGCGGGCGCCGACGACAACGTCGGCAAGCACCTGGACATCTTCAAC
LD71a           GAATTGACCGACAAGGCGGGCGCCGACGACAACGTCGGCAAGCACCTGGACATCTTCAAC
LE3-1           GAATTGACCGACAAGGCGGGCGCCGACGACAACGTCGGCAAGCACCTGGACATCTTCAAC
LE20-1          GAATTGACCGACAAGGCGGGCGCCGACGACAACGTCGGCAAGCACCTGGACATCTTCAAC
LG102           GAATTGACCGACAAGGCGGGCGCCGACGACAACGTCGGCAAGCACCTGGACATCTTCAAC
LG117           GAATTGACCGACAAGGCGGGCGCCGACGACAACGTCGGCAAGCACCTGGACATCTTCAAC
LH37-1          GAATTGACCGACAAGGCGGGCGCCGACGACAACGTCGGCAAGCACCTGGACATCTTCAAC
LMG9322         GAATTGACCGACAAGGCGGGCGCCGACGACAACGTCGGCAAGCACCTGGACATCTTCAAC
NCPPB3562       GAATTGACCGACAAGGCGGGCGCCGACGACAACGTCGGCAAGCACCTGGACATCTTCAAC
NCPPB3607       GAATTGACCGACAAGGCGGGCGCCGACGACAACGTCGGCAAGCACCTGGACATCTTCAAC
NCPPB3612       GAATTGACCGACAAGGCGGGCGCCGACGACAACGTCGGCAAGCACCTGGACATCTTCAAC
LB302           GAATT----GACAAGGCGGGCGCCGACGACAACGTCGGCAAGCACCTGGACATCTTCAAC
LG115           GAATT----GACAAGGCGGGCGCCGACGACAACGTCGGCAAGCACCTGGACATCTTCAAC
NCPPB3608       GAATT----GACAAGGCGGGCGCCGACGACAACGTCGGCAAGCACCTGGACATCTTCAAC
X2003-3218      GAATT----GACAAGGCGGGCGCCGACGACAACGTCGGCAAGCACCTGGACATCTTCAAC
                *****    ***************************************************

C40             CGCCGTGCGCGGCGCGGGCAGTGCTTCCAGGCCCCGTGCCTGGGCACGCGCGAATTCCCG
CFBP2852        CGCCGTGCGCGGCGCGGGCAGTGCTTCCAGGCCCCGTGCCTGGGCACGCGCGAATTCCCG
CFBP2911        CGCCGTGCGCGGCGCGGGCAGTGCTTCCAGGCCCCGTGCCTGGGCACGCGCGAATTCCCG
FDC217          CGCCGTGCGCGGCGCGGGCAGTGCTTCCAGGCCCCGTGCCTGGGCACGCGCGAATTCCCG
FDC1083         CGCCGTGCGCGGCGCGGGCAGTGCTTCCAGGCCCCGTGCCTGGGCACGCGCGAATTCCCG
JF90-2          CGCCGTGCGCGGCGCGGGCAGTGCTTCCAGGCCCCGTGCCTGGGCACGCGCGAATTCCCG
JF90-8          CGCCGTGCGCGGCGCGGGCAGTGCTTCCAGGCCCCGTGCCTGGGCACGCGCGAATTCCCG
JJ10-1          CGCCGTGCGCGGCGCGGGCAGTGCTTCCAGGCCCCGTGCCTGGGCACGCGCGAATTCCCG
JJ238-10        CGCCGTGCGCGGCGCGGGCAGTGCTTCCAGGCCCCGTGCCTGGGCACGCGCGAATTCCCG
JJ238-24        CGCCGTGCGCGGCGCGGGCAGTGCTTCCAGGCCCCGTGCCTGGGCACGCGCGAATTCCCG
JK2-10          CGCCGTGCGCGGCGCGGGCAGTGCTTCCAGGCCCCGTGCCTGGGCACGCGCGAATTCCCG
JK4-1           CGCCGTGCGCGGCGCGGGCAGTGCTTCCAGGCCCCGTGCCTGGGCACGCGCGAATTCCCG
JK48            CGCCGTGCGCGGCGCGGGCAGTGCTTCCAGGCCCCGTGCCTGGGCACGCGCGAATTCCCG
JK143-9         CGCCGTGCGCGGCGCGGGCAGTGCTTCCAGGCCCCGTGCCTGGGCACGCGCGAATTCCCG
JK143-11        CGCCGTGCGCGGCGCGGGCAGTGCTTCCAGGCCCCGTGCCTGGGCACGCGCGAATTCCCG
JM35            CGCCGTGCGCGGCGCGGGCAGTGCTTCCAGGCCCCGTGCCTGGGCACGCGCGAATTCCCG
JS581           CGCCGTGCGCGGCGCGGGCAGTGCTTCCAGGCCCCGTGCCTGGGCACGCGCGAATTCCCG
JS584           CGCCGTGCGCGGCGCGGGCAGTGCTTCCAGGCCCCGTGCCTGGGCACGCGCGAATTCCCG
JW160-1         CGCCGTGCGCGGCGCGGGCAGTGCTTCCAGGCCCCGTGCCTGGGCACGCGCGAATTCCCG
LB100-1         CGCCGTGCGCGGCGCGGGCAGTGCTTCCAGGCCCCGTGCCTGGGCACGCGCGAATTCCCG
LC80            CGCCGTGCGCGGCGCGGGCAGTGCTTCCAGGCCCCGTGCCTGGGCACGCGCGAATTCCCG
LD7-1           CGCCGTGCGCGGCGCGGGCAGTGCTTCCAGGCCCCGTGCCTGGGCACGCGCGAATTCCCG
LD71a           CGCCGTGCGCGGCGCGGGCAGTGCTTCCAGGCCCCGTGCCTGGGCACGCGCGAATTCCCG
LE3-1           CGCCGTGCGCGGCGCGGGCAGTGCTTCCAGGCCCCGTGCCTGGGCACGCGCGAATTCCCG
LE20-1          CGCCGTGCGCGGCGCGGGCAGTGCTTCCAGGCCCCGTGCCTGGGCACGCGCGAATTCCCG
LG102           CGCCGTGCGCGGCGCGGGCAGTGCTTCCAGGCCCCGTGCCTGGGCACGCGCGAATTCCCG
LG117           CGCCGTGCGCGGCGCGGGCAGTGCTTCCAGGCCCCGTGCCTGGGCACGCGCGAATTCCCG
LH37-1          CGCCGTGCGCGGCGCGGGCAGTGCTTCCAGGCCCCGTGCCTGGGCACGCGCGAATTCCCG
LMG9322         CGCCGTGCGCGGCGCGGGCAGTGCTTCCAGGCCCCGTGCCTGGGCACGCGCGAATTCCCG
NCPPB3562       CGCCGTGCGCGGCGCGGGCAGTGCTTCCAGGCCCCGTGCCTGGGCACGCGCGAATTCCCG
NCPPB3607       CGCCGTGCGCGGCGCGGGCAGTGCTTCCAGGCCCCGTGCCTGGGCACGCGCGAATTCCCG
NCPPB3612       CGCCGTGCGCGGCGCGGGCAGTGCTTCCAGGCCCCGTGCCTGGGCACGCGCGAATTCCCG
LB302           CGCCGTGCGCGGCGCGGGCAGTGCTTCCAGGCCCCGTGCCTGGGCACGCGCGAATTCCCG
LG115           CGCCGTGCGCGGCGCGGGCAGTGCTTCCAGGCCCCGTGCCTGGGCACGCGCGAATTCCCG
NCPPB3608       CGCCGTGCGCGGCGCGGGCAGTGCTTCCAGGCCCCGTGCCTGGGCACGCGCGAATTCCCG
X2003-3218      CGCCGTGCGCGGCGCGGGCAGTGCTTCCAGGCCCCGTGCCTGGGCACGCGCGAATTCCCG
                ************************************************************
```

```
C40             GCCAGCTTCGCGCTGATCGAAGACGACGCGGCGATGCCGGCTGCCGATGCAACGCTGGCC
CFBP2852        GCCAGCTTCGCGCTGATCGAAGACGACGCGGCGATGCCGGCTGCCGATGCAACGCTGGCC
CFBP2911        GCCAGCTTCGCGCTGATCGAAGACGACGCGGCGATGCCGGCTGCCGATGCAACGCTGGCC
FDC217          GCCAGCTTCGCGCTGATCGAAGACGACGCGGCGATGCCGGCTGCCGATGCAACGCTGGCC
FDC1083         GCCAGCTTCGCGCTGATCGAAGACGACGCGGCGATGCCGGCTGCCGATGCAACGCTGGCC
JF90-2          GCCAGCTTCGCGCTGATCGAAGACGACGCGGCGATGCCGGCTGCCGATGCAACGCTGGCC
JF90-8          GCCAGCTTCGCGCTGATCGAAGACGACGCGGCGATGCCGGCTGCCGATGCAACGCTGGCC
JJ10-1          GCCAGCTTCGCGCTGATCGAAGACGACGCGGCGATGCCGGCTGCCGATGCAACGCTGGCC
JJ238-10        GCCAGCTTCGCGCTGATCGAAGACGACGCGGCGATGCCGGCTGCCGATGCAACGCTGGCC
JJ238-24        GCCAGCTTCGCGCTGATCGAAGACGACGCGGCGATGCCGGCTGCCGATGCAACGCTGGCC
JK2-10          GCCAGCTTCGCGCTGATCGAAGACGACGCGGCGATGCCGGCTGCCGATGCAACGCTGGCC
JK4-1           GCCAGCTTCGCGCTGATCGAAGACGACGCGGCGATGCCGGCTGCCGATGCAACGCTGGCC
JK48            GCCAGCTTCGCGCTGATCGAAGACGACGCGGCGATGCCGGCTGCCGATGCAACGCTGGCC
JK143-9         GCCAGCTTCGCGCTGATCGAAGACGACGCGGCGATGCCGGCTGCCGATGCAACGCTGGCC
JK143-11        GCCAGCTTCGCGCTGATCGAAGACGACGCGGCGATGCCGGCTGCCGATGCAACGCTGGCC
JM35            GCCAGCTTCGCGCTGATCGAAGACGACGCGGCGATGCCGGCTGCCGATGCAACGCTGGCC
JS581           GCCAGCTTCGCGCTGATCGAAGACGACGCGGCGATGCCGGCTGCCGATGCAACGCTGGCC
JS584           GCCAGCTTCGCGCTGATCGAAGACGACGCGGCGATGCCGGCTGCCGATGCAACGCTGGCC
JW160-1         GCCAGCTTCGCGCTGATCGAAGACGACGCGGCGATGCCGGCTGCCGATGCAACGCTGGCC
LB100-1         GCCAGCTTCGCGCTGATCGAAGACGACGCGGCGATGCCGGCTGCCGATGCAACGCTGGCC
LC80            GCCAGCTTCGCGCTGATCGAAGACGACGCGGCGATGCCGGCTGCCGATGCAACGCTGGCC
LD7-1           GCCAGCTTCGCGCTGATCGAAGACGACGCGGCGATGCCGGCTGCCGATGCAACGCTGGCC
LD71a           GCCAGCTTCGCGCTGATCGAAGACGACGCGGCGATGCCGGCTGCCGATGCAACGCTGGCC
LE3-1           GCCAGCTTCGCGCTGATCGAAGACGACGCGGCGATGCCGGCTGCCGATGCAACGCTGGCC
LE20-1          GCCAGCTTCGCGCTGATCGAAGACGACGCGGCGATGCCGGCTGCCGATGCAACGCTGGCC
LG102           GCCAGCTTCGCGCTGATCGAAGACGACGCGGCGATGCCGGCTGCCGATGCAACGCTGGCC
LG117           GCCAGCTTCGCGCTGATCGAAGACGACGCGGCGATGCCGGCTGCCGATGCAACGCTGGCC
LH37-1          GCCAGCTTCGCGCTGATCGAAGACGACGCGGCGATGCCGGCTGCCGATGCAACGCTGGCC
LMG9322         GCCAGCTTCGCGCTGATCGAAGACGACGCGGCGATGCCGGCTGCCGATGCAACGCTGGCC
NCPPB3562       GCCAGCTTCGCGCTGATCGAAGACGACGCGGCGATGCCGGCTGCCGATGCAACGCTGGCC
NCPPB3607       GCCAGCTTCGCGCTGATCGAAGACGACGCGGCGATGCCGGCTGCCGATGCAACGCTGGCC
NCPPB3612       GCCAGCTTCGCGCTGATCGAAGACGACGCGGCGATGCCGGCTGCCGATGCAACGCTGGCC
LB302           GCCAGCTTCGCGCTGATCGAAGACGACGCGGCGATGCCGGCTGCCGATGCAACGCTGGCC
LG115           GCCAGCTTCGCGCTGATCGAAGACGACGCGGCGATGCCGGCTGCCGATGCAACGCTGGCC
NCPPB3608       GCCAGCTTCGCGCTGATCGAAGACGACGCGGCGATGCCGGCTGCCGATGCAACGCTGGCC
X2003-3218      GCCAGCTTCGCGCTGATCGAAGACGACGCGGCGATGCCGGCTGCCGATGCAACGCTGGCC
                ************************************************************

C40             GGCGAGCGCGACCTGGGCTGGATGCTGCACGACATCGACTTCGCCGACGGCATGACCCCG
CFBP2852        GGCGAGCGCGACCTGGGCTGGATGCTGCACGACATCGACTTCGCCGACGGCATGACCCCG
CFBP2911        GGCGAGCGCGACCTGGGCTGGATGCTGCACGACATCGACTTCGCCGACGGCATGACCCCG
FDC217          GGCGAGCGCGACCTGGGCTGGATGCTGCACGACATCGACTTCGCCGACGGCATGACCCCG
FDC1083         GGCGAGCGCGACCTGGGCTGGATGCTGCACGACATCGACTTCGCCGACGGCATGACCCCG
JF90-2          GGCGAGCGCGACCTGGGCTGGATGCTGCACGACATCGACTTCGCCGACGGCATGACCCCG
JF90-8          GGCGAGCGCGACCTGGGCTGGATGCTGCACGACATCGACTTCGCCGACGGCATGACCCCG
JJ10-1          GGCGAGCGCGACCTGGGCTGGATGCTGCACGACATCGACTTCGCCGACGGCATGACCCCG
JJ238-10        GGCGAGCGCGACCTGGGCTGGATGCTGCACGACATCGACTTCGCCGACGGCATGACCCCG
JJ238-24        GGCGAGCGCGACCTGGGCTGGATGCTGCACGACATCGACTTCGCCGACGGCATGACCCCG
JK2-10          GGCGAGCGCGACCTGGGCTGGATGCTGCACGACATCGACTTCGCCGACGGCATGACCCCG
JK4-1           GGCGAGCGCGACCTGGGCTGGATGCTGCACGACATCGACTTCGCCGACGGCATGACCCCG
JK48            GGCGAGCGCGACCTGGGCTGGATGCTGCACGACATCGACTTCGCCGACGGCATGACCCCG
JK143-9         GGCGAGCGCGACCTGGGCTGGATGCTGCACGACATCGACTTCGCCGACGGCATGACCCCG
JK143-11        GGCGAGCGCGACCTGGGCTGGATGCTGCACGACATCGACTTCGCCGACGGCATGACCCCG
JM35            GGCGAGCGCGACCTGGGCTGGATGCTGCACGACATCGACTTCGCCGACGGCATGACCCCG
JS581           GGCGAGCGCGACCTGGGCTGGATGCTGCACGACATCGACTTCGCCGACGGCATGACCCCG
JS584           GGCGAGCGCGACCTGGGCTGGATGCTGCACGACATCGACTTCGCCGACGGCATGACCCCG
JW160-1         GGCGAGCGCGACCTGGGCTGGATGCTGCACGACATCGACTTCGCCGACGGCATGACCCCG
LB100-1         GGCGAGCGCGACCTGGGCTGGATGCTGCACGACATCGACTTCGCCGACGGCATGACCCCG
LC80            GGCGAGCGCGACCTGGGCTGGATGCTGCACGACATCGACTTCGCCGACGGCATGACCCCG
LD7-1           GGCGAGCGCGACCTGGGCTGGATGCTGCACGACATCGACTTCGCCGACGGCATGACCCCG
LD71a           GGCGAGCGCGACCTGGGCTGGATGCTGCACGACATCGACTTCGCCGACGGCATGACCCCG
LE3-1           GGCGAGCGCGACCTGGGCTGGATGCTGCACGACATCGACTTCGCCGACGGCATGACCCCG
LE20-1          GGCGAGCGCGACCTGGGCTGGATGCTGCACGACATCGACTTCGCCGACGGCATGACCCCG
LG102           GGCGAGCGCGACCTGGGCTGGATGCTGCACGACATCGACTTCGCCGACGGCATGACCCCG
LG117           GGCGAGCGCGACCTGGGCTGGATGCTGCACGACATCGACTTCGCCGACGGCATGACCCCG
LH37-1          GGCGAGCGCGACCTGGGCTGGATGCTGCACGACATCGACTTCGCCGACGGCATGACCCCG
LMG9322         GGCGAGCGCGACCTGGGCTGGATGCTGCACGACATCGACTTCGCCGACGGCATGACCCCG
NCPPB3562       GGCGAGCGCGACCTGGGCTGGATGCTGCACGACATCGACTTCGCCGACGGCATGACCCCG
NCPPB3607       GGCGAGCGCGACCTGGGCTGGATGCTGCACGACATCGACTTCGCCGACGGCATGACCCCG
NCPPB3612       GGCGAGCGCGACCTGGGCTGGATGCTGCACGACATCGACTTCGCCGACGGCATGACCCCG
LB302           GGCGAGCGCGACCTGGGCTGGATGCTGCACGACATCGACTTCGCCGACGGCATGACCCCG
LG115           GGCGAGCGCGACCTGGGCTGGATGCTGCACGACATCGACTTCGCCGACGGCATGACCCCG
NCPPB3608       GGCGAGCGCGACCTGGGCTGGATGCTGCACGACATCGACTTCGCCGACGGCATGACCCCG
X2003-3218      GGCGAGCGCGACCTGGGCTGGATGCTGCACGACATCGACTTCGCCGACGGCATGACCCCG
                ************************************************************
```

```
C40             CGCTTCTTCCGCGCACGCATGGTCGATGGCCAGGTCGAGGTGCCAGCGCCGGAAAACGGT
CFBP2852        CGCTTCTTCCGCGCACGCATGGTCGATGGCCAGGTCGAGGTGCCAGCGCCGGAAAACGGT
CFBP2911        CGCTTCTTCCGCGCACGCATGGTCGATGGCCAGGTCGAGGTGCCAGCGCCGGAAAACGGT
FDC217          CGCTTCTTCCGCGCACGCATGGTCGATGGCCAGGTCGAGGTGCCAGCGCCGGAAAACGGT
FDC1083         CGCTTCTTCCGCGCACGCATGGTCGATGGCCAGGTCGAGGTGCCAGCGCCGGAAAACGGT
JF90-2          CGCTTCTTCCGCGCACGCATGGTCGATGGCCAGGTCGAGGTGCCAGCGCCGGAAAACGGT
JF90-8          CGCTTCTTCCGCGCACGCATGGTCGATGGCCAGGTCGAGGTGCCAGCGCCGGAAAACGGT
JJ10-1          CGCTTCTTCCGCGCACGCATGGTCGATGGCCAGGTCGAGGTGCCAGCGCCGGAAAACGGT
JJ238-10        CGCTTCTTCCGCGCACGCATGGTCGATGGCCAGGTCGAGGTGCCAGCGCCGGAAAACGGT
JJ238-24        CGCTTCTTCCGCGCACGCATGGTCGATGGCCAGGTCGAGGTGCCAGCGCCGGAAAACGGT
JK2-10          CGCTTCTTCCGCGCACGCATGGTCGATGGCCAGGTCGAGGTGCCAGCGCCGGAAAACGGT
JK4-1           CGCTTCTTCCGCGCACGCATGGTCGATGGCCAGGTCGAGGTGCCAGCGCCGGAAAACGGT
JK48            CGCTTCTTCCGCGCACGCATGGTCGATGGCCAGGTCGAGGTGCCAGCGCCGGAAAACGGT
JK143-9         CGCTTCTTCCGCGCACGCATGGTCGATGGCCAGGTCGAGGTGCCAGCGCCGGAAAACGGT
JK143-11        CGCTTCTTCCGCGCACGCATGGTCGATGGCCAGGTCGAGGTGCCAGCGCCGGAAAACGGT
JM35            CGCTTCTTCCGCGCACGCATGGTCGATGGCCAGGTCGAGGTGCCAGCGCCGGAAAACGGT
JS581           CGCTTCTTCCGCGCACGCATGGTCGATGGCCAGGTCGAGGTGCCAGCGCCGGAAAACGGT
JS584           CGCTTCTTCCGCGCACGCATGGTCGATGGCCAGGTCGAGGTGCCAGCGCCGGAAAACGGT
JW160-1         CGCTTCTTCCGCGCACGCATGGTCGATGGCCAGGTCGAGGTGCCAGCGCCGGAAAACGGT
LB100-1         CGCTTCTTCCGCGCACGCATGGTCGATGGCCAGGTCGAGGTGCCAGCGCCGGAAAACGGT
LC80            CGCTTCTTCCGCGCACGCATGGTCGATGGCCAGGTCGAGGTGCCAGCGCCGGAAAACGGT
LD7-1           CGCTTCTTCCGCGCACGCATGGTCGATGGCCAGGTCGAGGTGCCAGCGCCGGAAAACGGT
LD71a           CGCTTCTTCCGCGCACGCATGGTCGATGGCCAGGTCGAGGTGCCAGCGCCGGAAAACGGT
LE3-1           CGCTTCTTCCGCGCACGCATGGTCGATGGCCAGGTCGAGGTGCCAGCGCCGGAAAACGGT
LE20-1          CGCTTCTTCCGCGCACGCATGGTCGATGGCCAGGTCGAGGTGCCAGCGCCGGAAAACGGT
LG102           CGCTTCTTCCGCGCACGCATGGTCGATGGCCAGGTCGAGGTGCCAGCGCCGGAAAACGGT
LG117           CGCTTCTTCCGCGCACGCATGGTCGATGGCCAGGTCGAGGTGCCAGCGCCGGAAAACGGT
LH37-1          CGCTTCTTCCGCGCACGCATGGTCGATGGCCAGGTCGAGGTGCCAGCGCCGGAAAACGGT
LMG9322         CGCTTCTTCCGCGCACGCATGGTCGATGGCCAGGTCGAGGTGCCAGCGCCGGAAAACGGT
NCPPB3562       CGCTTCTTCCGCGCACGCATGGTCGATGGCCAGGTCGAGGTGCCAGCGCCGGAAAACGGT
NCPPB3607       CGCTTCTTCCGCGCACGCATGGTCGATGGCCAGGTCGAGGTGCCAGCGCCGGAAAACGGT
NCPPB3612       CGCTTCTTCCGCGCACGCATGGTCGATGGCCAGGTCGAGGTGCCAGCGCCGGAAAACGGT
LB302           CGCTTCTTCCGCGCACGCATGGTCGATGGCCAGGTCGAGGTGCCAGCGCCGGAAAACGGT
LG115           CGCTTCTTCCGCGCACGCATGGTCGATGGCCAGGTCGAGGTGCCAGCGCCGGAAAACGGT
NCPPB3608       CGCTTCTTCCGCGCACGCATGGTCGATGGCCAGGTCGAGGTGCCAGCGCCGGAAAACGGT
X2003-3218      CGCTTCTTCCGCGCACGCATGGTCGATGGCCAGGTCGAGGTGCCAGCGCCGGAAAACGGT
                ************************************************************

C40             GGGGTGCGCGCATGA
CFBP2852        GGGGTGCGCGCATGA
CFBP2911        GGGGTGCGCGCATGA
FDC217          GGGGTGCGCGCATGA
FDC1083         GGGGTGCGCGCATGA
JF90-2          GGGGTGCGCGCATGA
JF90-8          GGGGTGCGCGCATGA
JJ10-1          GGGGTGCGCGCATGA
JJ238-10        GGGGTGCGCGCATGA
JJ238-24        GGGGTGCGCGCATGA
JK2-10          GGGGTGCGCGCATGA
JK4-1           GGGGTGCGCGCATGA
JK48            GGGGTGCGCGCATGA
JK143-9         GGGGTGCGCGCATGA
JK143-11        GGGGTGCGCGCATGA
JM35            GGGGTGCGCGCATGA
JS581           GGGGTGCGCGCATGA
JS584           GGGGTGCGCGCATGA
JW160-1         GGGGTGCGCGCATGA
LB100-1         GGGGTGCGCGCATGA
LC80            GGGGTGCGCGCATGA
LD7-1           GGGGTGCGCGCATGA
LD71a           GGGGTGCGCGCATGA
LE3-1           GGGGTGCGCGCATGA
LE20-1          GGGGTGCGCGCATGA
LG102           GGGGTGCGCGCATGA
LG117           GGGGTGCGCGCATGA
LH37-1          GGGGTGCGCGCATGA
LMG9322         GGGGTGCGCGCATGA
NCPPB3562       GGGGTGCGCGCATGA
NCPPB3607       GGGGTGCGCGCATGA
NCPPB3612       GGGGTGCGCGCATGA
LB302           GGGGTGCGCGCATGA
LG115           GGGGTGCGCGCATGA
NCPPB3608       GGGGTGCGCGCATGA
X2003-3218      GGGGTGCGCGCATGA
                ***************
```

Cas8c-Csd1 (Cascade)

```
JJ238-24        ATGATCCTGTCCGCACTTGCCGACTACTACCACCGGCTGCTGGACGATCCCACATCGGGG
JK143-9         ATGATCCTGTCCGCACTTGCCGACTACTACCACCGGCTGCTGGACGATCCCACATCGGGG
JK143-11        ATGATCCTGTCCGCACTTGCCGACTACTACCACCGGCTGCTGGACGATCCCACATCGGGG
LD71a           ATGATCCTGTCCGCACTTGCCGACTACTACCACCGGCTGCTGGACGATCCCACATCGGGG
C40             ATGATCCTGTCCGCACTTGCCGACTACTACCACCGGCTGCTGGACGATCCCACATCGGGG
NCPPB3612       ATGATCCTGTCCGCACTTGCCGACTACTACCACCGGCTGCTGGACGATCCCACATCGGGG
CFBP2852        ATGATCCTGTCCGCACTTGCCGACTACTACCACCGGCTGCTGGACGATCCCACATCGGGG
FDC217          ATGATCCTGTCCGCACTTGCCGACTACTACCACCGGCTGCTGGACGATCCCACATCGGGG
FDC1083         ATGATCCTGTCCGCACTTGCCGACTACTACCACCGGCTGCTGGACGATCCCACATCGGGG
JJ10-1          ATGATCCTGTCCGCACTTGCCGACTACTACCACCGGCTGCTGGACGATCCCACATCGGGG
JJ238-10        ATGATCCTGTCCGCACTTGCCGACTACTACCACCGGCTGCTGGACGATCCCACATCGGGG
JK4-1           ATGATCCTGTCCGCACTTGCCGACTACTACCACCGGCTGCTGGACGATCCCACATCGGGG
JW160-1         ATGATCCTGTCCGCACTTGCCGACTACTACCACCGGCTGCTGGACGATCCCACATCGGGG
LB100-1         ATGATCCTGTCCGCACTTGCCGACTACTACCACCGGCTGCTGGACGATCCCACATCGGGG
LC80            ATGATCCTGTCCGCACTTGCCGACTACTACCACCGGCTGCTGGACGATCCCACATCGGGG
LG117           ATGATCCTGTCCGCACTTGCCGACTACTACCACCGGCTGCTGGACGATCCCACATCGGGG
LH37-1          ATGATCCTGTCCGCACTTGCCGACTACTACCACCGGCTGCTGGACGATCCCACATCGGGG
LMG9322         ATGATCCTGTCCGCACTTGCCGACTACTACCACCGGCTGCTGGACGATCCCACATCGGGG
NCPPB3562       ATGATCCTGTCCGCACTTGCCGACTACTACCACCGGCTGCTGGACGATCCCACATCGGGG
CFBP2911        ATGATCCTGTCCGCACTTGCCGACTACTACCACCGGCTGCTGGACGATCCCACATCGGGG
JF90-2          ATGATCCTGTCCGCACTTGCCGACTACTACCACCGGCTGCTGGACGATCCCACATCGGGG
JK2-10          ATGATCCTGTCCGCACTTGCCGACTACTACCACCGGCTGCTGGACGATCCCACATCGGGG
JK48            ATGATCCTGTCCGCACTTGCCGACTACTACCACCGGCTGCTGGACGATCCCACATCGGGG
JM35            ATGATCCTGTCCGCACTTGCCGACTACTACCACCGGCTGCTGGACGATCCCACATCGGGG
JS581           ATGATCCTGTCCGCACTTGCCGACTACTACCACCGGCTGCTGGACGATCCCACATCGGGG
JS582           ATGATCCTGTCCGCACTTGCCGACTACTACCACCGGCTGCTGGACGATCCCACATCGGGG
JS584           ATGATCCTGTCCGCACTTGCCGACTACTACCACCGGCTGCTGGACGATCCCACATCGGGG
LB302           ATGATCCTGTCCGCACTTGCCGACTACTACCACCGGCTGCTGGACGATCCCACATCGGGG
LE20-1          ATGATCCTGTCCGCACTTGCCGACTACTACCACCGGCTGCTGGACGATCCCACATCGGGG
LG102           ATGATCCTGTCCGCACTTGCCGACTACTACCACCGGCTGCTGGACGATCCCACATCGGGG
LG115           ATGATCCTGTCCGCACTTGCCGACTACTACCACCGGCTGCTGGACGATCCCACATCGGGG
NCPPB3607       ATGATCCTGTCCGCACTTGCCGACTACTACCACCGGCTGCTGGACGATCCCACATCGGGG
NCPPB3608       ATGATCCTGTCCGCACTTGCCGACTACTACCACCGGCTGCTGGACGATCCCACATCGGGG
X2003-3218      ATGATCCTGTCCGCACTTGCCGACTACTACCACCGGCTGCTGGACGATCCCACATCGGGG
                ************************************************************

JJ238-24        ATCGCCGCGCCCGGCTACAGCCAGGAGAAGATCGGCTACACCATCGTGCTGGACGGCGGT
JK143-9         ATCGCCGCGCCCGGCTACAGCCAGGAGAAGATCGGCTACACCATCGTGCTGGACGGCGGT
JK143-11        ATCGCCGCGCCCGGCTACAGCCAGGAGAAGATCGGCTACACCATCGTGCTGGACGGCGGT
LD71a           ATCGCCGCGCCCGGCTACAGCCAGGAGAAGATCGGCTACACCATCGTGCTGGACGGCGGT
C40             ATCGCCGCGCCCGGCTACAGCCAGGAGAAGATCGGCTACACCATCGTGCTGGACGGCGGT
NCPPB3612       ATCGCCGCGCCCGGCTACAGCCAGGAGAAGATCGGCTACACCATCGTGCTGGACGGCGGT
CFBP2852        ATCGCCGCGCCCGGCTACAGCCAGGAGAAGATCGGCTACACCATCGTGCTGGACGGCGGT
FDC217          ATCGCCGCGCCCGGCTACAGCCAGGAGAAGATCGGCTACACCATCGTGCTGGACGGCGGT
FDC1083         ATCGCCGCGCCCGGCTACAGCCAGGAGAAGATCGGCTACACCATCGTGCTGGACGGCGGT
JJ10-1          ATCGCCGCGCCCGGCTACAGCCAGGAGAAGATCGGCTACACCATCGTGCTGGACGGCGGT
JJ238-10        ATCGCCGCGCCCGGCTACAGCCAGGAGAAGATCGGCTACACCATCGTGCTGGACGGCGGT
JK4-1           ATCGCCGCGCCCGGCTACAGCCAGGAGAAGATCGGCTACACCATCGTGCTGGACGGCGGT
JW160-1         ATCGCCGCGCCCGGCTACAGCCAGGAGAAGATCGGCTACACCATCGTGCTGGACGGCGGT
LB100-1         ATCGCCGCGCCCGGCTACAGCCAGGAGAAGATCGGCTACACCATCGTGCTGGACGGCGGT
LC80            ATCGCCGCGCCCGGCTACAGCCAGGAGAAGATCGGCTACACCATCGTGCTGGACGGCGGT
LG117           ATCGCCGCGCCCGGCTACAGCCAGGAGAAGATCGGCTACACCATCGTGCTGGACGGCGGT
LH37-1          ATCGCCGCGCCCGGCTACAGCCAGGAGAAGATCGGCTACACCATCGTGCTGGACGGCGGT
LMG9322         ATCGCCGCGCCCGGCTACAGCCAGGAGAAGATCGGCTACACCATCGTGCTGGACGGCGGT
NCPPB3562       ATCGCCGCGCCCGGCTACAGCCAGGAGAAGATCGGCTACACCATCGTGCTGGACGGCGGT
CFBP2911        ATCGCCGCGCCCGGCTACAGCCAGGAGAAGATCGGCTACACCATCGTGCTGGACGGCGGT
JF90-2          ATCGCCGCGCCCGGCTACAGCCAGGAGAAGATCGGCTACACCATCGTGCTGGACGGCGGT
JK2-10          ATCGCCGCGCCCGGCTACAGCCAGGAGAAGATCGGCTACACCATCGTGCTGGACGGCGGT
JK48            ATCGCCGCGCCCGGCTACAGCCAGGAGAAGATCGGCTACACCATCGTGCTGGACGGCGGT
JM35            ATCGCCGCGCCCGGCTACAGCCAGGAGAAGATCGGCTACACCATCGTGCTGGACGGCGGT
JS581           ATCGCCGCGCCCGGCTACAGCCAGGAGAAGATCGGCTACACCATCGTGCTGGACGGCGGT
JS582           ATCGCCGCGCCCGGCTACAGCCAGGAGAAGATCGGCTACACCATCGTGCTGGACGGCGGT
JS584           ATCGCCGCGCCCGGCTACAGCCAGGAGAAGATCGGCTACACCATCGTGCTGGACGGCGGT
LB302           ATCGCCGCGCCCGGCTACAGCCAGGAGAAGATCGGCTACACCATCGTGCTGGACGGCGGT
LE20-1          ATCGCCGCGCCCGGCTACAGCCAGGAGAAGATCGGCTACACCATCGTGCTGGACGGCGGT
LG102           ATCGCCGCGCCCGGCTACAGCCAGGAGAAGATCGGCTACACCATCGTGCTGGACGGCGGT
LG115           ATCGCCGCGCCCGGCTACAGCCAGGAGAAGATCGGCTACACCATCGTGCTGGACGGCGGT
NCPPB3607       ATCGCCGCGCCCGGCTACAGCCAGGAGAAGATCGGCTACACCATCGTGCTGGACGGCGGT
NCPPB3608       ATCGCCGCGCCCGGCTACAGCCAGGAGAAGATCGGCTACACCATCGTGCTGGACGGCGGT
X2003-3218      ATCGCCGCGCCCGGCTACAGCCAGGAGAAGATCGGCTACACCATCGTGCTGGACGGCGGT
                ************************************************************
```

```
JJ238-24        GGCCGCGTTGTGGCCGTGGAGGACGAACATGACTACGACAGCAAGAAGCGAGTCGCCAAG
JK143-9         GGCCGCGTTGTGGCCGTGGAGGACGAACATGACTACGACAGCAAGAAGCGAGTCGCCAAG
JK143-11        GGCCGCGTTGTGGCCGTGGAGGACGAACATGACTACGACAGCAAGAAGCGAGTCGCCAAG
LD71a           GGCCGCGTTGTGGCCGTGGAGGACGAACATGACTACGACAGCAAGAAGCGAGTCGCCAAG
C40             GGCCGCGTTGTGGCCGTGGAGGACGAACATGACTACGACAGCAAGAAGCGAGTCGCCAAG
NCPPB3612       GGCCGCGTTGTGGCCGTGGAGGACGAACATGACTACGACAGCAAAAAGCGAGTCGCCAAG
CFBP2852        GGCCGCGTTGTGGCCGTGGAGGACGAACATGACTACGACAGCAAGAAGCGAGTCGCCAAG
FDC217          GGCCGCGTTGTGGCCGTGGAGGACGAACATGACTACGACAGCAAGAAGCGAGTCGCCAAG
FDC1083         GGCCGCGTTGTGGCCGTGGAGGACGAACATGACTACGACAGCAAGAAGCGAGTCGCCAAG
JJ10-1          GGCCGCGTTGTGGCCGTGGAGGACGAACATGACTACGACAGCAAGAAGCGAGTCGCCAAG
JJ238-10        GGCCGCGTTGTGGCCGTGGAGGACGAACATGACTACGACAGCAAGAAGCGAGTCGCCAAG
JK4-1           GGCCGCGTTGTGGCCGTGGAGGACGAACATGACTACGACAGCAAGAAGCGAGTCGCCAAG
JW160-1         GGCCGCGTTGTGGCCGTGGAGGACGAACATGACTACGACAGCAAGAAGCGAGTCGCCAAG
LB100-1         GGCCGCGTTGTGGCCGTGGAGGACGAACATGACTACGACAGCAAGAAGCGAGTCGCCAAG
LC80            GGCCGCGTTGTGGCCGTGGAGGACGAACATGACTACGACAGCAAGAAGCGAGTCGCCAAG
LG117           GGCCGCGTTGTGGCCGTGGAGGACGAACATGACTACGACAGCAAGAAGCGAGTCGCCAAG
LH37-1          GGCCGCGTTGTGGCCGTGGAGGACGAACATGACTACGACAGCAAGAAGCGAGTCGCCAAG
LMG9322         GGCCGCGTTGTGGCCGTGGAGGACGAACATGACTACGACAGCAAGAAGCGAGTCGCCAAG
NCPPB3562       GGCCGCGTTGTGGCCGTGGAGGACGAACATGACTACGACAGCAAGAAGCGAGTCGCCAAG
CFBP2911        GGCCGCGTTGTGGCCGTGGAGGACGAACATGACTACGACAGCAAGAAGCGAGTCGCCAAG
JF90-2          GGCCGCGTTGTGGCCGTGGAGGACGAACATGACTACGACAGCAAGAAGCGAGTCGCCAAG
JK2-10          GGCCGCGTTGTGGCCGTGGAGGACGAACATGACTACGACAGCAAGAAGCGAGTCGCCAAG
JK48            GGCCGCGTTGTGGCCGTGGAGGACGAACATGACTACGACAGCAAGAAGCGAGTCGCCAAG
JM35            GGCCGCGTTGTGGCCGTGGAGGACGAACATGACTACGACAGCAAGAAGCGAGTCGCCAAG
JS581           GGCCGCGTTGTGGCCGTGGAGGACGAACATGACTACGACAGCAAGAAGCGAGTCGCCAAG
JS582           GGCCGCGTTGTGGCCGTGGAGGACGAACATGACTACGACAGCAAGAAGCGAGTCGCCAAG
JS584           GGCCGCGTTGTGGCCGTGGAGGACGAACATGACTACGACAGCAAGAAGCGAGTCGCCAAG
LB302           GGCCGCGTTGTGGCCGTGGAGGACGAACATGACTACGACAGCAAGAAGCGAGTCGCCAAG
LE20-1          GGCCGCGTTGTGGCCGTGGAGGACGAACATGACTACGACAGCAAGAAGCGAGTCGCCAAG
LG102           GGCCGCGTTGTGGCCGTGGAGGACGAACATGACTACGACAGCAAGAAGCGAGTCGCCAAG
LG115           GGCCGCGTTGTGGCCGTGGAGGACGAACATGACTACGACAGCAAGAAGCGAGTCGCCAAG
NCPPB3607       GGCCGCGTTGTGGCCGTGGAGGACGAACATGACTACGACAGCAAGAAGCGAGTCGCCAAG
NCPPB3608       GGCCGCGTTGTGGCCGTGGAGGACGAACATGACTACGACAGCAAGAAGCGAGTCGCCAAG
X2003-3218      GGCCGCGTTGTGGCCGTGGAGGACGAACATGACTACGACAGCAAGAAGCGAGTCGCCAAG
                ******************************************** ***************

JJ238-24        GCGCTGAGCGTGCCGCAGCCGGAGAAGCGCACCGTGGCGGTCAAGTCCAACTTTCTTTGG
JK143-9         GCGCTGAGCGTGCCGCAGCCGGAGAAGCGCACCGTGGCGGTCAAGTCCAACTTTCTTTGG
JK143-11        GCGCTGAGCGTGCCGCAGCCGGAGAAGCGCACCGTGGCGGTCAAGTCCAACTTTCTTTGG
LD71a           GCGCTGAGCGTGCCGCAGCCGGAGAAGCGCACCGTGGCGGTCAAGTCCAACTTTCTTTGG
C40             GCGCTGAGCGTGCCGCAGCCGGAGAAGCGCACCGTGGCGGTCAAGTCCAACTTTCTTTGG
NCPPB3612       GCGCTGAGCGTGCCGCAGCCGGAGAAGCGCACCGTGGCGGTCAAGTCCAACTTTCTTTGG
CFBP2852        GCGCTGAGCGTGCCGCAGCCGGAGAAGCGCACCGTGGCGGTCAAGTCCAACTTTCTTTGG
FDC217          GCGCTGAGCGTGCCGCAGCCGGAGAAGCGCACCGTGGCGGTCAAGTCCAACTTTCTTTGG
FDC1083         GCGCTGAGCGTGCCGCAGCCGGAGAAGCGCACCGTGGCGGTCAAGTCCAACTTTCTTTGG
JJ10-1          GCGCTGAGCGTGCCGCAGCCGGAGAAGCGCACCGTGGCGGTCAAGTCCAACTTTCTTTGG
JJ238-10        GCGCTGAGCGTGCCGCAGCCGGAGAAGCGCACCGTGGCGGTCAAGTCCAACTTTCTTTGG
JK4-1           GCGCTGAGCGTGCCGCAGCCGGAGAAGCGCACCGTGGCGGTCAAGTCCAACTTTCTTTGG
JW160-1         GCGCTGAGCGTGCCGCAGCCGGAGAAGCGCACCGTGGCGGTCAAGTCCAACTTTCTTTGG
LB100-1         GCGCTGAGCGTGCCGCAGCCGGAGAAGCGCACCGTGGCGGTCAAGTCCAACTTTCTTTGG
LC80            GCGCTGAGCGTGCCGCAGCCGGAGAAGCGCACCGTGGCGGTCAAGTCCAACTTTCTTTGG
LG117           GCGCTGAGCGTGCCGCAGCCGGAGAAGCGCACCGTGGCGGTCAAGTCCAACTTTCTTTGG
LH37-1          GCGCTGAGCGTGCCGCAGCCGGAGAAGCGCACCGTGGCGGTCAAGTCCAACTTTCTTTGG
LMG9322         GCGCTGAGCGTGCCGCAGCCGGAGAAGCGCACCGTGGCGGTCAAGTCCAACTTTCTTTGG
NCPPB3562       GCGCTGAGCGTGCCGCAGCCGGAGAAGCGCACCGTGGCGGTCAAGTCCAACTTTCTTTGG
CFBP2911        GCGCTGAGCGTGCCGCAGCCGGAGAAGCGCACCGTGGCGGTCAAGTCCAACTTTCTTTGG
JF90-2          GCGCTGAGCGTGCCGCAGCCGGAGAAGCGCACCGTGGCGGTCAAGTCCAACTTTCTTTGG
JK2-10          GCGCTGAGCGTGCCGCAGCCGGAGAAGCGCACCGTGGCGGTCAAGTCCAACTTTCTTTGG
JK48            GCGCTGAGCGTGCCGCAGCCGGAGAAGCGCACCGTGGCGGTCAAGTCCAACTTTCTTTGG
JM35            GCGCTGAGCGTGCCGCAGCCGGAGAAGCGCACCGTGGCGGTCAAGTCCAACTTTCTTTGG
JS581           GCGCTGAGCGTGCCGCAGCCGGAGAAGCGCACCGTGGCGGTCAAGTCCAACTTTCTTTGG
JS582           GCGCTGAGCGTGCCGCAGCCGGAGAAGCGCACCGTGGCGGTCAAGTCCAACTTTCTTTGG
JS584           GCGCTGAGCGTGCCGCAGCCGGAGAAGCGCACCGTGGCGGTCAAGTCCAACTTTCTTTGG
LB302           GCGCTGAGCGTGCCGCAGCCGGAGAAGCGCACCGTGGCGGTCAAGTCCAACTTTCTTTGG
LE20-1          GCGCTGAGCGTGCCGCAGCCGGAGAAGCGCACCGTGGCGGTCAAGTCCAACTTTCTTTGG
LG102           GCGCTGAGCGTGCCGCAGCCGGAGAAGCGCACCGTGGCGGTCAAGTCCAACTTTCTTTGG
LG115           GCGCTGAGCGTGCCGCAGCCGGAGAAGCGCACCGTGGCGGTCAAGTCCAACTTTCTTTGG
NCPPB3607       GCGCTGAGCGTGCCGCAGCCGGAGAAGCGCACCGTGGCGGTCAAGTCCAACTTTCTTTGG
NCPPB3608       GCGCTGAGCGTGCCGCAGCCGGAGAAGCGCACCGTGGCGGTCAAGTCCAACTTTCTTTGG
X2003-3218      GCGCTGAGCGTGCCGCAGCCGGAGAAGCGCACCGTGGCGGTCAAGTCCAACTTTCTTTGG
                ************************************************************
```

```
JJ238-24        GACAAGACCAGCTACGCGCTGGGCGTCAGTGCCAGCAGCAAGCGCAGCGCGCAGGAACAC
JK143-9         GACAAGACCAGCTACGCGCTGGGCGTCAGTGCCAGCAGCAAGCGCAGCGCGCAGGAACAC
JK143-11        GACAAGACCAGCTACGCGCTGGGCGTCAGTGCCAGCAGCAAGCGCAGCGCGCAGGAACAC
LD71a           GACAAGACCAGCTACGCGCTGGGCGTCAGTGCCAGCAGCAAGCGCAGCGCGCAGGAACAC
C40             GACAAGACCAGCTACGCGCTGGGCGTCAGTGCCAGCAGCAAGCGCAGCGCGCAGGAACAC
NCPPB3612       GACAAGACCAGCTACGCGCTGGGCGTCAGTGCCAGCAGCAAGCGCAGCGCGCAGGAACAC
CFBP2852        GACAAGACCAGCTACGCGCTGGGCGTCAGTGCCAGCAGCAAGCGCAGCGCGCAGGAACAC
FDC217          GACAAGACCAGCTACGCGCTGGGCGTCAGTGCCAGCAGCAAGCGCAGCGCGCAGGAACAC
FDC1083         GACAAGACCAGCTACGCGCTGGGCGTCAGTGCCAGCAGCAAGCGCAGCGCGCAGGAACAC
JJ10-1          GACAAGACCAGCTACGCGCTGGGCGTCAGTGCCAGCAGCAAGCGCAGCGCGCAGGAACAC
JJ238-10        GACAAGACCAGCTACGCGCTGGGCGTCAGTGCCAGCAGCAAGCGCAGCGCGCAGGAACAC
JK4-1           GACAAGACCAGCTACGCGCTGGGCGTCAGTGCCAGCAGCAAGCGCAGCGCGCAGGAACAC
JW160-1         GACAAGACCAGCTACGCGCTGGGCGTCAGTGCCAGCAGCAAGCGCAGCGCGCAGGAACAC
LB100-1         GACAAGACCAGCTACGCGCTGGGCGTCAGTGCCAGCAGCAAGCGCAGCGCGCAGGAACAC
LC80            GACAAGACCAGCTACGCGCTGGGCGTCAGTGCCAGCAGCAAGCGCAGCGCGCAGGAACAC
LG117           GACAAGACCAGCTACGCGCTGGGCGTCAGTGCCAGCAGCAAGCGCAGCGCGCAGGAACAC
LH37-1          GACAAGACCAGCTACGCGCTGGGCGTCAGTGCCAGCAGCAAGCGCAGCGCGCAGGAACAC
LMG9322         GACAAGACCAGCTACGCGCTGGGCGTCAGTGCCAGCAGCAAGCGCAGCGCGCAGGAACAC
NCPPB3562       GACAAGACCAGCTACGCGCTGGGCGTCAGTGCCAGCAGCAAGCGCAGCGCGCAGGAACAC
CFBP2911        GACAAGACCAGCTACGCGCTGGGCGTCAGTGCCAGCAGCAAGCGCAGCGCGCAGGAACAC
JF90-2          GACAAGACCAGCTACGCGCTGGGCGTCAGTGCCAGCAGCAAGCGCAGCGCGCAGGAACAC
JK2-10          GACAAGACCAGCTACGCGCTGGGCGTCAGTGCCAGCAGCAAGCGCAGCGCGCAGGAACAC
JK48            GACAAGACCAGCTACGCGCTGGGCGTCAGTGCCAGCAGCAAGCGCAGCGCGCAGGAACAC
JM35            GACAAGACCAGCTACGCGCTGGGCGTCAGTGCCAGCAGCAAGCGCAGCGCGCAGGAACAC
JS581           GACAAGACCAGCTACGCGCTGGGCGTCAGTGCCAGCAGCAAGCGCAGCGCGCAGGAACAC
JS582           GACAAGACCAGCTACGCGCTGGGCGTCAGTGCCAGCAGCAAGCGCAGCGCGCAGGAACAC
JS584           GACAAGACCAGCTACGCGCTGGGCGTCAGTGCCAGCAGCAAGCGCAGCGCGCAGGAACAC
LB302           GACAAGACCAGCTACGCGCTGGGCGTCAGTGCCAGCAGCAAGCGCAGCGCGCAGGAACAC
LE20-1          GACAAGACCAGCTACGCGCTGGGCGTCAGTGCCAGCAGCAAGCGCAGCGCGCAGGAACAC
LG102           GACAAGACCAGCTACGCGCTGGGCGTCAGTGCCAGCAGCAAGCGCAGCGCGCAGGAACAC
LG115           GACAAGACCAGCTACGCGCTGGGCGTCAGTGCCAGCAGCAAGCGCAGCGCGCAGGAACAC
NCPPB3607       GACAAGACCAGCTACGCGCTGGGCGTCAGTGCCAGCAGCAAGCGCAGCGCGCAGGAACAC
NCPPB3608       GACAAGACCAGCTACGCGCTGGGCGTCAGTGCCAGCAGCAAGCGCAGCGCGCAGGAACAC
X2003-3218      GACAAGACCAGCTACGCGCTGGGCGTCAGTGCCAGCAGCAAGCGCAGCGCGCAGGAACAC
                ************************************************************

JJ238-24        GCTGCATTCAAGACCTTGCACCAGCAGGCGCTCGGCGGCAGCGAAGACCCCGGGCTGCGT
JK143-9         GCTGCATTCAAGACCTTGCACCAGCAGGCGCTCGGCGGCAGCGAAGACCCCGGGCTGCGT
JK143-11        GCTGCATTCAAGACCTTGCACCAGCAGGCGCTCGGCGGCAGCGAAGACCCCGGGCTGCGT
LD71a           GCTGCATTCAAGACCTTGCACCAGCAGGCGCTCGGCGGCAGCGAAGACCCCGGGCTGCGT
C40             GCTGCATTCAAGACCTTGCACCAGCAGGCGCTCGGCGGCAGCGAAGACCCCGGGCTGCGT
NCPPB3612       GCTGCATTCAAGACCTTGCACCAGCAGGCGCTCGGCGGCAGCGAAGACCCCGGGCTGCGT
CFBP2852        GCTGCATTCAAGACCTTGCACCAGCAGGCGCTCGGCGGCAGCGAAGACCCCGGGCTGCGT
FDC217          GCTGCATTCAAGACCTTGCACCAGCAGGCGCTCGGCGGCAGCGAAGACCCCGGGCTGCGT
FDC1083         GCTGCATTCAAGACCTTGCACCAGCAGGCGCTCGGCGGCAGCGAAGACCCCGGGCTGCGT
JJ10-1          GCTGCATTCAAGACCTTGCACCAGCAGGCGCTCGGCGGCAGCGAAGACCCCGGGCTGCGT
JJ238-10        GCTGCATTCAAGACCTTGCACCAGCAGGCGCTCGGCGGCAGCGAAGACCCCGGGCTGCGT
JK4-1           GCTGCATTCAAGACCTTGCACCAGCAGGCGCTCGGCGGCAGCGAAGACCCCGGGCTGCGT
JW160-1         GCTGCATTCAAGACCTTGCACCAGCAGGCGCTCGGCGGCAGCGAAGACCCCGGGCTGCGT
LB100-1         GCTGCATTCAAGACCTTGCACCAGCAGGCGCTCGGCGGCAGCGAAGACCCCGGGCTGCGT
LC80            GCTGCATTCAAGACCTTGCACCAGCAGGCGCTCGGCGGCAGCGAAGACCCCGGGCTGCGT
LG117           GCTGCATTCAAGACCTTGCACCAGCAGGCGCTCGGCGGCAGCGAAGACCCCGGGCTGCGT
LH37-1          GCTGCATTCAAGACCTTGCACCAGCAGGCGCTCGGCGGCAGCGAAGACCCCGGGCTGCGT
LMG9322         GCTGCATTCAAGACCTTGCACCAGCAGGCGCTCGGCGGCAGCGAAGACCCCGGGCTGCGT
NCPPB3562       GCTGCATTCAAGACCTTGCACCAGCAGGCGCTCGGCGGCAGCGAAGACCCCGGGCTGCGT
CFBP2911        GCTGCATTCAAGACCTTGCACCAGCAGGCGCTCGGCGGCAGCGAAGACCCCGGGCTGCGT
JF90-2          GCTGCATTCAAGACCTTGCACCAGCAGGCGCTCGGCGGCAGCGAAGACCCCGGGCTGCGT
JK2-10          GCTGCATTCAAGACCTTGCACCAGCAGGCGCTCGGCGGCAGCGAAGACCCCGGGCTGCGT
JK48            GCTGCATTCAAGACCTTGCACCAGCAGGCGCTCGGCGGCAGCGAAGACCCCGGGCTGCGT
JM35            GCTGCATTCAAGACCTTGCACCAGCAGGCGCTCGGCGGCAGCGAAGACCCCGGGCTGCGT
JS581           GCTGCATTCAAGACCTTGCACCAGCAGGCGCTCGGCGGCAGCGAAGACCCCGGGCTGCGT
JS582           GCTGCATTCAAGACCTTGCACCAGCAGGCGCTCGGCGGCAGCGAAGACCCCGGGCTGCGT
JS584           GCTGCATTCAAGACCTTGCACCAGCAGGCGCTCGGCGGCAGCGAAGACCCCGGGCTGCGT
LB302           GCTGCATTCAAGACCTTGCACCAGCAGGCGCTCGGCGGCAGCGAAGACCCCGGGCTGCGT
LE20-1          GCTGCATTCAAGACCTTGCACCAGCAGGCGCTCGGCGGCAGCGAAGACCCCGGGCTGCGT
LG102           GCTGCATTCAAGACCTTGCACCAGCAGGCGCTCGGCGGCAGCGAAGACCCCGGGCTGCGT
LG115           GCTGCATTCAAGACCTTGCACCAGCAGGCGCTCGGCGGCAGCGAAGACCCCGGGCTGCGT
NCPPB3607       GCTGCATTCAAGACCTTGCACCAGCAGGCGCTCGGCGGCAGCGAAGACCCCGGGCTGCGT
NCPPB3608       GCTGCATTCAAGACCTTGCACCAGCAGGCGCTCGGCGGCAGCGAAGACCCCGGGCTGCGT
X2003-3218      GCTGCATTCAAGACCTTGCACCAGCAGGCGCTCGGCGGCAGCGAAGACCCCGGGCTGCGT
                ************************************************************
```

```
JJ238-24        GCGCTATTGACGTTCCTCGACACCTGGTCGCCCGCGCAATTCGCCGATCATCCACAGTTT
JK143-9         GCGCTATTGACGTTCCTCGACACCTGGTCGCCCGCGCAATTCGCCGATCATCCACAGTTT
JK143-11        GCGCTATTGACGTTCCTCGACACCTGGTCGCCCGCGCAATTCGCCGATCATCCACAGTTT
LD71a           GCGCTATTGACGTTCCTCGACACCTGGTCGCCCGCGCAATTCGCCGATCATCCACAGTTT
C40             GCGCTATTGACGTTCCTCGACGCCTGGTCGCCCGCGCAATTCGCCGATCATCCACAGTTT
NCPPB3612       GCGCTATTGACGTTCCTCGACACCTGGTCGCCCGCGCAATTCGCCGATCATCCACAGTTT
CFBP2852        GCGCTATTGACGTTCCTCGACACCTGGTCGCCCGCGCAATTCGCCGATCATCCACAGTTT
FDC217          GCGCTATTGACGTTCCTCGACACCTGGTCGCCCGCGCAATTCGCCGATCATCCACAGTTT
FDC1083         GCGCTATTGACGTTCCTCGACACCTGGTCGCCCGCGCAATTCGCCGATCATCCACAGTTT
JJ10-1          GCGCTATTGACGTTCCTCGACACCTGGTCGCCCGCGCAATTCGCCGATCATCCACAGTTT
JJ238-10        GCGCTATTGACGTTCCTCGACACCTGGTCGCCCGCGCAATTCGCCGATCATCCACAGTTT
JK4-1           GCGCTATTGACGTTCCTCGACACCTGGTCGCCCGCGCAATTCGCCGATCATCCACAGTTT
JW160-1         GCGCTATTGACGTTCCTCGACACCTGGTCGCCCGCGCAATTCGCCGATCATCCACAGTTT
LB100-1         GCGCTATTGACGTTCCTCGACACCTGGTCGCCCGCGCAATTCGCCGATCATCCACAGTTT
LC80            GCGCTATTGACGTTCCTCGACACCTGGTCGCCCGCGCAATTCGCCGATCATCCACAGTTT
LG117           GCGCTATTGACGTTCCTCGACACCTGGTCGCCCGCGCAATTCGCCGATCATCCACAGTTT
LH37-1          GCGCTATTGACGTTCCTCGACACCTGGTCGCCCGCGCAATTCGCCGATCATCCACAGTTT
LMG9322         GCGCTATTGACGTTCCTCGACACCTGGTCGCCCGCGCAATTCGCCGATCATCCACAGTTT
NCPPB3562       GCGCTATTGACGTTCCTCGACACCTGGTCGCCCGCGCAATTCGCCGATCATCCACAGTTT
CFBP2911        GCGCTATTGACGTTCCTCGACACCTGGTCGCCCGCGCAATTCGCCGATCATCCACAGTTT
JF90-2          GCGCTATTGACGTTCCTCGACACCTGGTCGCCCGCGCAATTCGCCGATCATCCACAGTTT
JK2-10          GCGCTATTGACGTTCCTCGACACCTGGTCGCCCGCGCAATTCGCCGATCATCCACAGTTT
JK48            GCGCTATTGACGTTCCTCGACACCTGGTCGCCCGCGCAATTCGCCGATCATCCACAGTTT
JM35            GCGCTATTGACGTTCCTCGACACCTGGTCGCCCGCGCAATTCGCCGATCATCCACAGTTT
JS581           GCGCTATTGACGTTCCTCGACACCTGGTCGCCCGCGCAATTCGCCGATCATCCACAGTTT
JS582           GCGCTATTGACGTTCCTCGACACCTGGTCGCCCGCGCAATTCGCCGATCATCCACAGTTT
JS584           GCGCTATTGACGTTCCTCGACACCTGGTCGCCCGCGCAATTCGCCGATCATCCACAGTTT
LB302           GCGCTATTGACGTTCCTCGACACCTGGTCGCCCGCGCAATTCGCCGATCATCCACAGTTT
LE20-1          GCGCTATTGACGTTCCTCGACACCTGGTCGCCCGCGCAATTCGCCGATCATCCACAGTTT
LG102           GCGCTATTGACGTTCCTCGACACCTGGTCGCCCGCGCAATTCGCCGATCATCCACAGTTT
LG115           GCGCTATTGACGTTCCTCGACACCTGGTCGCCCGCGCAATTCGCCGATCATCCACAGTTT
NCPPB3607       GCGCTATTGACGTTCCTCGACACCTGGTCGCCCGCGCAATTCGCCGATCATCCACAGTTT
NCPPB3608       GCGCTATTGACGTTCCTCGACACCTGGTCGCCCGCGCAATTCGCCGATCATCCACAGTTT
X2003-3218      GCGCTATTGACGTTCCTCGACACCTGGTCGCCCGCGCAATTCGCCGATCATCCACAGTTT
                ********************* **************************************

JJ238-24        TCCCGTCACGGCGAGGCGCTATTGGATACCAACCTGGTGTTCCGCCTGGAGGGCGACACC
JK143-9         TCCCGTCACGGCGAGGCGCTATTGGATACCAACCTGGTGTTCCGCCTGGAGGGCGACACC
JK143-11        TCCCGTCACGGCGAGGCGCTATTGGATACCAACCTGGTGTTCCGCCTGGAGGGCGACACC
LD71a           TCCCGTCACGGCGAGGCGCTATTGGATACCAACCTGGTGTTCCGCCTGGAGGGCGACACC
C40             TCCCGTCACGGCGAGGCGCTATTGGATACCAACCTGGTGTTCCGCCTGGAGGGCGACACC
NCPPB3612       TCCCGTCACGGCGAGGCGCTATTGGATACCAACCTGGTGTTCCGCCTGGAGGGCGACACC
CFBP2852        TCCCGTCACGGCGAGGCGCTATTGGATACCAACCTGGTGTTCCGCCTGGAGGGCGACACC
FDC217          TCCCGTCACGGCGAGGCGCTATTGGATACCAACCTGGTGTTCCGCCTGGAGGGCGACACC
FDC1083         TCCCGTCACGGCGAGGCGCTATTGGATACCAACCTGGTGTTCCGCCTGGAGGGCGACACC
JJ10-1          TCCCGTCACGGCGAGGCGCTATTGGATACCAACCTGGTGTTCCGCCTGGAGGGCGACACC
JJ238-10        TCCCGTCACGGCGAGGCGCTATTGGATACCAACCTGGTGTTCCGCCTGGAGGGCGACACC
JK4-1           TCCCGTCACGGCGAGGCGCTATTGGATACCAACCTGGTGTTCCGCCTGGAGGGCGACACC
JW160-1         TCCCGTCACGGCGAGGCGCTATTGGATACCAACCTGGTGTTCCGCCTGGAGGGCGACACC
LB100-1         TCCCGTCACGGCGAGGCGCTATTGGATACCAACCTGGTGTTCCGCCTGGAGGGCGACACC
LC80            TCCCGTCACGGCGAGGCGCTATTGGATACCAACCTGGTGTTCCGCCTGGAGGGCGACACC
LG117           TCCCGTCACGGCGAGGCGCTATTGGATACCAACCTGGTGTTCCGCCTGGAGGGCGACACC
LH37-1          TCCCGTCACGGCGAGGCGCTATTGGATACCAACCTGGTGTTCCGCCTGGAGGGCGACACC
LMG9322         TCCCGTCACGGCGAGGCGCTATTGGATACCAACCTGGTGTTCCGCCTGGAGGGCGACACC
NCPPB3562       TCCCGTCACGGCGAGGCGCTATTGGATACCAACCTGGTGTTCCGCCTGGAGGGCGACACC
CFBP2911        TCCCGTCACGGCGAGGCGCTATTGGATACCAACCTGGTGTTCCGCCTGGAGGGCGACACC
JF90-2          TCCCGTCACGGCGAGGCGCTATTGGATACCAACCTGGTGTTCCGCCTGGAGGGCGACACC
JK2-10          TCCCGTCACGGCGAGGCGCTATTGGATACCAACCTGGTGTTCCGCCTGGAGGGCGACACC
JK48            TCCCGTCACGGCGAGGCGCTATTGGATACCAACCTGGTGTTCCGCCTGGAGGGCGACACC
JM35            TCCCGTCACGGCGAGGCGCTATTGGATACCAACCTGGTGTTCCGCCTGGAGGGCGACACC
JS581           TCCCGTCACGGCGAGGCGCTATTGGATACCAACCTGGTGTTCCGCCTGGAGGGCGACACC
JS582           TCCCGTCACGGCGAGGCGCTATTGGATACCAACCTGGTGTTCCGCCTGGAGGGCGACACC
JS584           TCCCGTCACGGCGAGGCGCTATTGGATACCAACCTGGTGTTCCGCCTGGAGGGCGACACC
LB302           TCCCGTCACGGCGAGGCGCTATTGGATACCAACCTGGTGTTCCGCCTGGAGGGCGACACC
LE20-1          TCCCGTCACGGCGAGGCGCTATTGGATACCAACCTGGTGTTCCGCCTGGAGGGCGACACC
LG102           TCCCGTCACGGCGAGGCGCTATTGGATACCAACCTGGTGTTCCGCCTGGAGGGCGACACC
LG115           TCCCGTCACGGCGAGGCGCTATTGGATACCAACCTGGTGTTCCGCCTGGAGGGCGACACC
NCPPB3607       TCCCGTCACGGCGAGGCGCTATTGGATACCAACCTGGTGTTCCGCCTGGAGGGCGACACC
NCPPB3608       TCCCGTCACGGCGAGGCGCTATTGGATACCAACCTGGTGTTCCGCCTGGAGGGCGACACC
X2003-3218      TCCCGTCACGGCGAGGCGCTATTGGATACCAACCTGGTGTTCCGCCTGGAGGGCGACACC
                ************************************************************
```

```
JJ238-24        GGCTATCTGCATCAGCGCGCCACGGCGCGCGGGGCATGGGAGCGACTGCA--AGGCCAAA
JK143-9         GGCTATCTGCATCAGCGCGCCACGGCGCGCGGGGCATGGGAGCGACTGCA--AGGCCAAA
JK143-11        GGCTATCTGCATCAGCGCGCCACGGCGCGCGGGGCATGGGAGCGACTGCA--AGGCCAAA
LD71a           GGCTATCTGCATCAGCGCGCCACGGCGCGCGGGGCATGGGAGCGACTGCA--AGGCCAAA
C40             GGCTATCTGCATCAGCGCGCCACGGCGCGCGCGGCATGGGAGCGACTGCAAGAGGCCAAA
NCPPB3612       GGCTATCTGCATCAGCGCGCCACGGCGCGCGCGGCATGGGAGCGACTGCAAGAGGCCAAA
CFBP2852        GGCTATCTGCATCAGCGCGCCACGGCGCGCGCGGCATGGGAGCGACTGCAAGAGGCCAAA
FDC217          GGCTATCTGCATCAGCGCGCCACGGCGCGCGCGGCATGGGAGCGACTGCAAGAGGCCAAA
FDC1083         GGCTATCTGCATCAGCGCGCCACGGCGCGCGCGGCATGGGAGCGACTGCAAGAGGCCAAA
JJ10-1          GGCTATCTGCATCAGCGCGCCACGGCGCGCGCGGCATGGGAGCGACTGCAAGAGGCCAAA
JJ238-10        GGCTATCTGCATCAGCGCGCCACGGCGCGCGCGGCATGGGAGCGACTGCAAGAGGCCAAA
JK4-1           GGCTATCTGCATCAGCGCGCCACGGCGCGCGCGGCATGGGAGCGACTGCAAGAGGCCAAA
JW160-1         GGCTATCTGCATCAGCGCGCCACGGCGCGCGCGGCATGGGAGCGACTGCAAGAGGCCAAA
LB100-1         GGCTATCTGCATCAGCGCGCCACGGCGCGCGCGGCATGGGAGCGACTGCAAGAGGCCAAA
LC80            GGCTATCTGCATCAGCGCGCCACGGCGCGCGCGGCATGGGAGCGACTGCAAGAGGCCAAA
LG117           GGCTATCTGCATCAGCGCGCCACGGCGCGCGCGGCATGGGAGCGACTGCAAGAGGCCAAA
LH37-1          GGCTATCTGCATCAGCGCGCCACGGCGCGCGCGGCATGGGAGCGACTGCAAGAGGCCAAA
LMG9322         GGCTATCTGCATCAGCGCGCCACGGCGCGCGCGGCATGGGAGCGACTGCAAGAGGCCAAA
NCPPB3562       GGCTATCTGCATCAGCGCGCCACGGCGCGCGCGGCATGGGAGCGACTGCAAGAGGCCAAA
CFBP2911        GGCTATCTGCATCAGCGCGCCACGGCGCGCGCGGCATGGGAGCGACTGCA--AGGCCAAA
JF90-2          GGCTATCTGCATCAGCGCGCCACGGCGCGCGCGGCATGGGAGCGACTGCA--AGGCCAAA
JK2-10          GGCTATCTGCATCAGCGCGCCACGGCGCGCGCGGCATGGGAGCGACTGCA--AGGCCAAA
JK48            GGCTATCTGCATCAGCGCGCCACGGCGCGCGCGGCATGGGAGCGACTGCA--AGGCCAAA
JM35            GGCTATCTGCATCAGCGCGCCACGGCGCGCGCGGCATGGGAGCGACTGCA--AGGCCAAA
JS581           GGCTATCTGCATCAGCGCGCCACGGCGCGCGCGGCATGGGAGCGACTGCA--AGGCCAAA
JS582           GGCTATCTGCATCAGCGCGCCACGGCGCGCGCGGCATGGGAGCGACTGCA--AGGCCAAA
JS584           GGCTATCTGCATCAGCGCGCCACGGCGCGCGCGGCATGGGAGCGACTGCA--AGGCCAAA
LB302           GGCTATCTGCATCAGCGCGCCACGGCGCGCGCGGCATGGGAGCGACTGCA--AGGCCAAA
LE20-1          GGCTATCTGCATCAGCGCGCCACGGCGCGCGCGGCATGGGAGCGACTGCA--AGGCCAAA
LG102           GGCTATCTGCATCAGCGCGCCACGGCGCGCGCGGCATGGGAGCGACTGCA--AGGCCAAA
LG115           GGCTATCTGCATCAGCGCGCCACGGCGCGCGCGGCATGGGAGCGACTGCA--AGGCCAAA
NCPPB3607       GGCTATCTGCATCAGCGCGCCACGGCGCGCGCGGCATGGGAGCGACTGCA--AGGCCAAA
NCPPB3608       GGCTATCTGCATCAGCGCGCCACGGCGCGCGCGGCATGGGAGCGACTGCA--AGGCCAAA
X2003-3218      GGCTATCTGCATCAGCGCGCCACGGCGCGCGCGGCATGGGAGCGACTGCA--AGGCCAAA
                ******************************* ******************  ********

JJ238-24        GCGCCGACGGCGCATCCGGCATGTGCCTGGTCAGTGGCTTACGCGCGCCGTTGGCGCGGC
JK143-9         GCGCCGACGGCGCATCCGGCATGTGCCTGGTCAGTGGCTTACGCGCGCCGTTGGCGCGGC
JK143-11        GCGCCGACGGCGCATCCGGCATGTGCCTGGTCAGTGGCTTACGCGCGCCGTTGGCGCGGC
LD71a           GCGCCGACGGCGCATCCGGCATGTGCCTGGTCAGTGGCTTACGCGCGCCGTTGGCGCGGC
C40             GCGCCGACGGCGCATCCGGCATGTGCCTGGTCAGTGGCTTACGCGCGCCGTTGGCGCGGC
NCPPB3612       GCGCCGACGGCGCATCCGGCATGTGCCTGGTCAGTGGCTTACGCGCGCCGTTGGCGCGGC
CFBP2852        GCGCCGACGGCGCATCCGGCATGTGCCTGGTCAGTGGCTTACGCGCGCCGTTGGCGCGGC
FDC217          GCGCCGACGGCGCATCCGGCATGTGCCTGGTCAGTGGCTTACGCGCGCCGTTGGCGCGGC
FDC1083         GCGCCGACGGCGCATCCGGCATGTGCCTGGTCAGTGGCTTACGCGCGCCGTTGGCGCGGC
JJ10-1          GCGCCGACGGCGCATCCGGCATGTGCCTGGTCAGTGGCTTACGCGCGCCGTTGGCGCGGC
JJ238-10        GCGCCGACGGCGCATCCGGCATGTGCCTGGTCAGTGGCTTACGCGCGCCGTTGGCGCGGC
JK4-1           GCGCCGACGGCGCATCCGGCATGTGCCTGGTCAGTGGCTTACGCGCGCCGTTGGCGCGGC
JW160-1         GCGCCGACGGCGCATCCGGCATGTGCCTGGTCAGTGGCTTACGCGCGCCGTTGGCGCGGC
LB100-1         GCGCCGACGGCGCATCCGGCATGTGCCTGGTCAGTGGCTTACGCGCGCCGTTGGCGCGGC
LC80            GCGCCGACGGCGCATCCGGCATGTGCCTGGTCAGTGGCTTACGCGCGCCGTTGGCGCGGC
LG117           GCGCCGACGGCGCATCCGGCATGTGCCTGGTCAGTGGCTTACGCGCGCCGTTGGCGCGGC
LH37-1          GCGCCGACGGCGCATCCGGCATGTGCCTGGTCAGTGGCTTACGCGCGCCGTTGGCGCGGC
LMG9322         GCGCCGACGGCGCATCCGGCATGTGCCTGGTCAGTGGCTTACGCGCGCCGTTGGCGCGGC
NCPPB3562       GCGCCGACGGCGCATCCGGCATGTGCCTGGTCAGTGGCTTACGCGCGCCGTTGGCGCGGC
CFBP2911        GCGCCGACGGCGCATCCGGCATGTGCCTGGTCAGTGGCTTACGCGCGCCGTTGGCGCGGC
JF90-2          GCGCCGACGGCGCATCCGGCATGTGCCTGGTCAGTGGCTTACGCGCGCCGTTGGCGCGGC
JK2-10          GCGCCGACGGCGCATCCGGCATGTGCCTGGTCAGTGGCTTACGCGCGCCGTTGGCGCGGC
JK48            GCGCCGACGGCGCATCCGGCATGTGCCTGGTCAGTGGCTTACGCGCGCCGTTGGCGCGGC
JM35            GCGCCGACGGCGCATCCGGCATGTGCCTGGTCAGTGGCTTACGCGCGCCGTTGGCGCGGC
JS581           GCGCCGACGGCGCATCCGGCATGTGCCTGGTCAGTGGCTTACGCGCGCCGTTGGCGCGGC
JS582           GCGCCGACGGCGCATCCGGCATGTGCCTGGTCAGTGGCTTACGCGCGCCGTTGGCGCGGC
JS584           GCGCCGACGGCGCATCCGGCATGTGCCTGGTCAGTGGCTTACGCGCGCCGTTGGCGCGGC
LB302           GCGCCGACGGCGCATCCGGCATGTGCCTGGTCAGTGGCTTACGCGCGCCGTTGGCGCGGC
LE20-1          GCGCCGACGGCGCATCCGGCATGTGCCTGGTCAGTGGCTTACGCGCGCCGTTGGCGCGGC
LG102           GCGCCGACGGCGCATCCGGCATGTGCCTGGTCAGTGGCTTACGCGCGCCGTTGGCGCGGC
LG115           GCGCCGACGGCGCATCCGGCATGTGCCTGGTCAGTGGCTTACGCGCGCCGTTGGCGCGGC
NCPPB3607       GCGCCGACGGCGCATCCGGCATGTGCCTGGTCAGTGGCTTACGCGCGCCGTTGGCGCGGC
NCPPB3608       GCGCCGACGGCGCATCCGGCATGTGCCTGGTCAGTGGCTTACGCGCGCCGTTGGCGCGGC
X2003-3218      GCGCCGACGGCGCATCCGGCATGTGCCTGGTCAGTGGCTTACGCGCGCCGTTGGCGCGGC
                ************************************************************
```

```
JJ238-24        TGCACCCGCCGATCAAAGGATTTGAAAGCACGGGATCTTCCATCGTTTCTTTTAACGCGG
JK143-9         TGCACCCGCCGATCAAAGGATTTGAAAGCACGGGATCTTCCATCGTTTCTTTTAACGCGG
JK143-11        TGCACCCGCCGATCAAAGGATTTGAAAGCACGGGATCTTCCATCGTTTCTTTTAACGCGG
LD71a           TGCACCCGCCGATCAAAGGATTTGAAAGCACGGGATCTTCCATCGTTTCTTTTAACGCGG
C40             TGCACCCGCCGATCAAAGGATTTGAAAGCACGGGATCTTCCATCGTTTCTTTTAACGCGG
NCPPB3612       TGCACCCGCCGATCAAAGGATTTGAAAGCACGGGATCTTCCATCGTTTCTTTTAACGCGG
CFBP2852        TGCACCCGCCGATCAAAGGATTTGAAAGCACGGGATCTTCCATCGTTTCTTTTAACGCGG
FDC217          TGCACCCGCCGATCAAAGGATTTGAAAGCACGGGATCTTCCATCGTTTCTTTTAACGCGG
FDC1083         TGCACCCGCCGATCAAAGGATTTGAAAGCACGGGATCTTCCATCGTTTCTTTTAACGCGG
JJ10-1          TGCACCCGCCGATCAAAGGATTTGAAAGCACGGGATCTTCCATCGTTTCTTTTAACGCGG
JJ238-10        TGCACCCGCCGATCAAAGGATTTGAAAGCACGGGATCTTCCATCGTTTCTTTTAACGCGG
JK4-1           TGCACCCGCCGATCAAAGGATTTGAAAGCACGGGATCTTCCATCGTTTCTTTTAACGCGG
JW160-1         TGCACCCGCCGATCAAAGGATTTGAAAGCACGGGATCTTCCATCGTTTCTTTTAACGCGG
LB100-1         TGCACCCGCCGATCAAAGGATTTGAAAGCACGGGATCTTCCATCGTTTCTTTTAACGCGG
LC80            TGCACCCGCCGATCAAAGGATTTGAAAGCACGGGATCTTCCATCGTTTCTTTTAACGCGG
LG117           TGCACCCGCCGATCAAAGGATTTGAAAGCACGGGATCTTCCATCGTTTCTTTTAACGCGG
LH37-1          TGCACCCGCCGATCAAAGGATTTGAAAGCACGGGATCTTCCATCGTTTCTTTTAACGCGG
LMG9322         TGCACCCGCCGATCAAAGGATTTGAAAGCACGGGATCTTCCATCGTTTCTTTTAACGCGG
NCPPB3562       TGCACCCGCCGATCAAAGGATTTGAAAGCACGGGATCTTCCATCGTTTCTTTTAACGCGG
CFBP2911        TGCACCCGCCGATCAAAGGATTTGAAAGCACGGGATCTTCCATCGTTTCTTTTAACGCGG
JF90-2          TGCACCCGCCGATCAAAGGATTTGAAAGCACGGGATCTTCCATCGTTTCTTTTAACGCGG
JK2-10          TGCACCCGCCGATCAAAGGATTTGAAAGCACGGGATCTTCCATCGTTTCTTTTAACGCGG
JK48            TGCACCCGCCGATCAAAGGATTTGAAAGCACGGGATCTTCCATCGTTTCTTTTAACGCGG
JM35            TGCACCCGCCGATCAAAGGATTTGAAAGCACGGGATCTTCCATCGTTTCTTTTAACGCGG
JS581           TGCACCCGCCGATCAAAGGATTTGAAAGCACGGGATCTTCCATCGTTTCTTTTAACGCGG
JS582           TGCACCCGCCGATCAAAGGATTTGAAAGCACGGGATCTTCCATCGTTTCTTTTAACGCGG
JS584           TGCACCCGCCGATCAAAGGATTTGAAAGCACGGGATCTTCCATCGTTTCTTTTAACGCGG
LB302           TGCACCCGCCGATCAAAGGATTTGAAAGCACGGGATCTTCCATCGTTTCTTTTAACGCGG
LE20-1          TGCACCCGCCGATCAAAGGATTTGAAAGCACGGGATCTTCCATCGTTTCTTTTAACGCGG
LG102           TGCACCCGCCGATCAAAGGATTTGAAAGCACGGGATCTTCCATCGTTTCTTTTAACGCGG
LG115           TGCACCCGCCGATCAAAGGATTTGAAAGCACGGGATCTTCCATCGTTTCTTTTAACGCGG
NCPPB3607       TGCACCCGCCGATCAAAGGATTTGAAAGCACGGGATCTTCCATCGTTTCTTTTAACGCGG
NCPPB3608       TGCACCCGCCGATCAAAGGATTTGAAAGCACGGGATCTTCCATCGTTTCTTTTAACGCGG
X2003-3218      TGCACCCGCCGATCAAAGGATTTGAAAGCACGGGATCTTCCATCGTTTCTTTTAACGCGG
                ************************************************************

JJ238-24        ATGCTTATATTTCATACAGAGGTTCGCTCCTAAAAGTTCAGTCGAAACACAAGGAAAATG
JK143-9         ATGCTTATATTTCATACAGAGGTTCGCTCCTAAAAGTTCAGTCGAAACACAAGGAAAATG
JK143-11        ATGCTTATATTTCATACAGAGGTTCGCTCCTAAAAGTTCAGTCGAAACACAAGGAAAATG
LD71a           ATGCTTATATTTCATACAGAGGTTCGCTCCTAAAAGTTCAGTCGAAACACAAGGAAAATG
C40             ATGCTTATATTTCATACAGAGGTTCGCTCCTAAAAGTTCAGTCGAAACACAAGGAAAATG
NCPPB3612       ATGCTTATATTTCATACAGAGGTTCGCTCCTAAAAGTTCAGTCGAAACACAAGGAAAATG
CFBP2852        ATGCTTATATTTCATACAGAGGTTCGCTCCTAAAAGTTCAGTCGAAACACAAGGAAAATG
FDC217          ATGCTTATATTTCATACAGAGGTTCGCTCCTAAAAGTTCAGTCGAAACACAAGGAAAATG
FDC1083         ATGCTTATATTTCATACAGAGGTTCGCTCCTAAAAGTTCAGTCGAAACACAAGGAAAATG
JJ10-1          ATGCTTATATTTCATACAGAGGTTCGCTCCTAAAAGTTCAGTCGAAACACAAGGAAAATG
JJ238-10        ATGCTTATATTTCATACAGAGGTTCGCTCCTAAAAGTTCAGTCGAAACACAAGGAAAATG
JK4-1           ATGCTTATATTTCATACAGAGGTTCGCTCCTAAAAGTTCAGTCGAAACACAAGGAAAATG
JW160-1         ATGCTTATATTTCATACAGAGGTTCGCTCCTAAAAGTTCAGTCGAAACACAAGGAAAATG
LB100-1         ATGCTTATATTTCATACAGAGGTTCGCTCCTAAAAGTTCAGTCGAAACACAAGGAAAATG
LC80            ATGCTTATATTTCATACAGAGGTTCGCTCCTAAAAGTTCAGTCGAAACACAAGGAAAATG
LG117           ATGCTTATATTTCATACAGAGGTTCGCTCCTAAAAGTTCAGTCGAAACACAAGGAAAATG
LH37-1          ATGCTTATATTTCATACAGAGGTTCGCTCCTAAAAGTTCAGTCGAAACACAAGGAAAATG
LMG9322         ATGCTTATATTTCATACAGAGGTTCGCTCCTAAAAGTTCAGTCGAAACACAAGGAAAATG
NCPPB3562       ATGCTTATATTTCATACAGAGGTTCGCTCCTAAAAGTTCAGTCGAAACACAAGGAAAATG
CFBP2911        ATGCTTATATTTCATACAGAGGTTCGCTCCTAAAAGTTCAGTCGAAACACAAGGAAAATG
JF90-2          ATGCTTATATTTCATACAGAGGTTCGCTCCTAAAAGTTCAGTCGAAACACAAGGAAAATG
JK2-10          ATGCTTATATTTCATACAGAGGTTCGCTCCTAAAAGTTCAGTCGAAACACAAGGAAAATG
JK48            ATGCTTATATTTCATACAGAGGTTCGCTCCTAAAAGTTCAGTCGAAACACAAGGAAAATG
JM35            ATGCTTATATTTCATACAGAGGTTCGCTCCTAAAAGTTCAGTCGAAACACAAGGAAAATG
JS581           ATGCTTATATTTCATACAGAGGTTCGCTCCTAAAAGTTCAGTCGAAACACAAGGAAAATG
JS582           ATGCTTATATTTCATACAGAGGTTCGCTCCTAAAAGTTCAGTCGAAACACAAGGAAAATG
JS584           ATGCTTATATTTCATACAGAGGTTCGCTCCTAAAAGTTCAGTCGAAACACAAGGAAAATG
LB302           ATGCTTATATTTCATACAGAGGTTCGCTCCTAAAAGTTCAGTCGAAACACAAGGAAAATG
LE20-1          ATGCTTATATTTCATACAGAGGTTCGCTCCTAAAAGTTCAGTCGAAACACAAGGAAAATG
LG102           ATGCTTATATTTCATACAGAGGTTCGCTCCTAAAAGTTCAGTCGAAACACAAGGAAAATG
LG115           ATGCTTATATTTCATACAGAGGTTCGCTCCTAAAAGTTCAGTCGAAACACAAGGAAAATG
NCPPB3607       ATGCTTATATTTCATACAGAGGTTCGCTCCTAAAAGTTCAGTCGAAACACAAGGAAAATG
NCPPB3608       ATGCTTATATTTCATACAGAGGTTCGCTCCTAAAAGTTCAGTCGAAACACAAGGAAAATG
X2003-3218      ATGCTTATATTTCATACAGAGGTTCGCTCCTAAAAGTTCAGTCGAAACACAAGGAAAATG
                ************************************************************
```

```
JJ238-24        ATAGCGGTGCCAATGCGCCGATTTCTAAATCTGCGGCTTTCGCCTACACCACCGCGCTCA
JK143-9         ATAGCGGTGCCAATGCGCCGATTTCTAAATCTGCGGCTTTCGCCTACACCACCGCGCTCA
JK143-11        ATAGCGGTGCCAATGCGCCGATTTCTAAATCTGCGGCTTTCGCCTACACCACCGCGCTCA
LD71a           ATAGCGGTGCCAATGCGCCGATTTCTAAATCTGCGGCTTTCGCCTACACCACCGCGCTCA
C40             ATAGCGGTGCCAATGCGCCGATTTCTAAATCTGCGGCTTTCGCCTACACCACCGCGCTCA
NCPPB3612       ATAGCGGTGCCAATGCGCCGATTTCTAAATCTGCGGCTTTCGCCTACACCACCGCGCTCA
CFBP2852        ATAGCGGTGCCAATGCGCCGATTTCTAAATCTGCGGCTTTCGCCTACACCACCGCGCTCA
FDC217          ATAGCGGTGCCAATGCGCCGATTTCTAAATCTGCGGCTTTCGCCTACACCACCGCGCTCA
FDC1083         ATAGCGGTGCCAATGCGCCGATTTCTAAATCTGCGGCTTTCGCCTACACCACCGCGCTCA
JJ10-1          ATAGCGGTGCCAATGCGCCGATTTCTAAATCTGCGGCTTTCGCCTACACCACCGCGCTCA
JJ238-10        ATAGCGGTGCCAATGCGCCGATTTCTAAATCTGCGGCTTTCGCCTACACCACCGCGCTCA
JK4-1           ATAGCGGTGCCAATGCGCCGATTTCTAAATCTGCGGCTTTCGCCTACACCACCGCGCTCA
JW160-1         ATAGCGGTGCCAATGCGCCGATTTCTAAATCTGCGGCTTTCGCCTACACCACCGCGCTCA
LB100-1         ATAGCGGTGCCAATGCGCCGATTTCTAAATCTGCGGCTTTCGCCTACACCACCGCGCTCA
LC80            ATAGCGGTGCCAATGCGCCGATTTCTAAATCTGCGGCTTTCGCCTACACCACCGCGCTCA
LG117           ATAGCGGTGCCAATGCGCCGATTTCTAAATCTGCGGCTTTCGCCTACACCACCGCGCTCA
LH37-1          ATAGCGGTGCCAATGCGCCGATTTCTAAATCTGCGGCTTTCGCCTACACCACCGCGCTCA
LMG9322         ATAGCGGTGCCAATGCGCCGATTTCTAAATCTGCGGCTTTCGCCTACACCACCGCGCTCA
NCPPB3562       ATAGCGGTGCCAATGCGCCGATTTCTAAATCTGCGGCTTTCGCCTACACCACCGCGCTCA
CFBP2911        ATAGCGGTGCCAATGCGCCGATTTCTAAATCTGCGGCTTTCGCCTACACCACCGCGCTCA
JF90-2          ATAGCGGTGCCAATGCGCCGATTTCTAAATCTGCGGCTTTCGCCTACACCACCGCGCTCA
JK2-10          ATAGCGGTGCCAATGCGCCGATTTCTAAATCTGCGGCTTTCGCCTACACCACCGCGCTCA
JK48            ATAGCGGTGCCAATGCGCCGATTTCTAAATCTGCGGCTTTCGCCTACACCACCGCGCTCA
JM35            ATAGCGGTGCCAATGCGCCGATTTCTAAATCTGCGGCTTTCGCCTACACCACCGCGCTCA
JS581           ATAGCGGTGCCAATGCGCCGATTTCTAAATCTGCGGCTTTCGCCTACACCACCGCGCTCA
JS582           ATAGCGGTGCCAATGCGCCGATTTCTAAATCTGCGGCTTTCGCCTACACCACCGCGCTCA
JS584           ATAGCGGTGCCAATGCGCCGATTTCTAAATCTGCGGCTTTCGCCTACACCACCGCGCTCA
LB302           ATAGCGGTGCCAATGCGCCGATTTCTAAATCTGCGGCTTTCGCCTACACCACCGCGCTCA
LE20-1          ATAGCGGTGCCAATGCGCCGATTTCTAAATCTGCGGCTTTCGCCTACACCACCGCGCTCA
LG102           ATAGCGGTGCCAATGCGCCGATTTCTAAATCTGCGGCTTTCGCCTACACCACCGCGCTCA
LG115           ATAGCGGTGCCAATGCGCCGATTTCTAAATCTGCGGCTTTCGCCTACACCACCGCGCTCA
NCPPB3607       ATAGCGGTGCCAATGCGCCGATTTCTAAATCTGCGGCTTTCGCCTACACCACCGCGCTCA
NCPPB3608       ATAGCGGTGCCAATGCGCCGATTTCTAAATCTGCGGCTTTCGCCTACACCACCGCGCTCA
X2003-3218      ATAGCGGTGCCAATGCGCCGATTTCTAAATCTGCGGCTTTCGCCTACACCACCGCGCTCA
                ************************************************************

JJ238-24        ACCATCTGCTGCGCCGCGACCCACGCAATCGCCAACGTGTGCAGATCGGCGACACCACGG
JK143-9         ACCATCTGCTGCGCCGCGACCCACGCAATCGCCAACGTGTGCAGATCGGCGACACCACGG
JK143-11        ACCATCTGCTGCGCCGCGACCCACGCAATCGCCAACGTGTGCAGATCGGCGACACCACGG
LD71a           ACCATCTGCTGCGCCGCGACCCACGCAATCGCCAACGTGTGCAGATCGGCGACACCACGG
C40             ACCATCTGCTGCGCCGCGACCCACGCAATCGCCAACGTGTGCAGATCGGCGACACCACGG
NCPPB3612       ACCATCTGCTGCGCCGCGACCCACGCAATCGCCAACGTGTGCAGATCGGCGACACCACGG
CFBP2852        ACCATCTGCTGCGCCGCGACCCACGCAATCGCCAACGTGTGCAGATCGGCGACACCACGG
FDC217          ACCATCTGCTGCGCCGCGACCCACGCAATCGCCAACGTGTGCAGATCGGCGACACCACGG
FDC1083         ACCATCTGCTGCGCCGCGACCCACGCAATCGCCAACGTGTGCAGATCGGCGACACCACGG
JJ10-1          ACCATCTGCTGCGCCGCGACCCACGCAATCGCCAACGTGTGCAGATCGGCGACACCACGG
JJ238-10        ACCATCTGCTGCGCCGCGACCCACGCAATCGCCAACGTGTGCAGATCGGCGACACCACGG
JK4-1           ACCATCTGCTGCGCCGCGACCCACGCAATCGCCAACGTGTGCAGATCGGCGACACCACGG
JW160-1         ACCATCTGCTGCGCCGCGACCCACGCAATCGCCAACGTGTGCAGATCGGCGACACCACGG
LB100-1         ACCATCTGCTGCGCCGCGACCCACGCAATCGCCAACGTGTGCAGATCGGCGACACCACGG
LC80            ACCATCTGCTGCGCCGCGACCCACGCAATCGCCAACGTGTGCAGATCGGCGACACCACGG
LG117           ACCATCTGCTGCGCCGCGACCCACGCAATCGCCAACGTGTGCAGATCGGCGACACCACGG
LH37-1          ACCATCTGCTGCGCCGCGACCCACGCAATCGCCAACGTGTGCAGATCGGCGACACCACGG
LMG9322         ACCATCTGCTGCGCCGCGACCCACGCAATCGCCAACGTGTGCAGATCGGCGACACCACGG
NCPPB3562       ACCATCTGCTGCGCCGCGACCCACGCAATCGCCAACGTGTGCAGATCGGCGACACCACGG
CFBP2911        ACCATCTGCTGCGCCGCGACCCACGCAATCGCCAACGTGTGCAGATCGGCGACACCACGG
JF90-2          ACCATCTGCTGCGCCGCGACCCACGCAATCGCCAACGTGTGCAGATCGGCGACACCACGG
JK2-10          ACCATCTGCTGCGCCGCGACCCACGCAATCGCCAACGTGTGCAGATCGGCGACACCACGG
JK48            ACCATCTGCTGCGCCGCGACCCACGCAATCGCCAACGTGTGCAGATCGGCGACACCACGG
JM35            ACCATCTGCTGCGCCGCGACCCACGCAATCGCCAACGTGTGCAGATCGGCGACACCACGG
JS581           ACCATCTGCTGCGCCGCGACCCACGCAATCGCCAACGTGTGCAGATCGGCGACACCACGG
JS582           ACCATCTGCTGCGCCGCGACCCACGCAATCGCCAACGTGTGCAGATCGGCGACACCACGG
JS584           ACCATCTGCTGCGCCGCGACCCACGCAATCGCCAACGTGTGCAGATCGGCGACACCACGG
LB302           ACCATCTGCTGCGCCGCGACCCACGCAATCGCCAACGTGTGCAGATCGGCGACACCACGG
LE20-1          ACCATCTGCTGCGCCGCGACCCACGCAATCGCCAACGTGTGCAGATCGGCGACACCACGG
LG102           ACCATCTGCTGCGCCGCGACCCACGCAATCGCCAACGTGTGCAGATCGGCGACACCACGG
LG115           ACCATCTGCTGCGCCGCGACCCACGCAATCGCCAACGTGTGCAGATCGGCGACACCACGG
NCPPB3607       ACCATCTGCTGCGCCGCGACCCACGCAATCGCCAACGTGTGCAGATCGGCGACACCACGG
NCPPB3608       ACCATCTGCTGCGCCGCGACCCACGCAATCGCCAACGTGTGCAGATCGGCGACACCACGG
X2003-3218      ACCATCTGCTGCGCCGCGACCCACGCAATCGCCAACGTGTGCAGATCGGCGACACCACGG
                ************************************************************
```

```
JJ238-24        TGGTGTTCTGGGCGCAGGCCAGGACGACCGCGCACGCTGAGGAGGCCGAAGATCTGATCG
JK143-9         TGGTGTTCTGGGCGCAGGCCAGGACGACCGCGCACGCTGAGGAGGCCGAAGATCTGATCG
JK143-11        TGGTGTTCTGGGCGCAGGCCAGGACGACCGCGCACGCTGAGGAGGCCGAAGATCTGATCG
LD71a           TGGTGTTCTGGGCGCAGGCCAGGACGACCGCGCACGCTGAGGAGGCCGAAGATCTGATCG
C40             TGGTGTTCTGGGCGCAGGCCAGGACGACCGCGCACGCTGAGGAGGCCGAAGATCTGATCG
NCPPB3612       TGGTGTTCTGGGCGCAGGCCAGGACGACCGCGCACGCTGAGGAGGCCGAAGATCTGATCG
CFBP2852        TGGTGTTCTGGGCGCAGGCCAGGACGACCGCGCACGCTGAGGAGGCCGAAGATCTGATCG
FDC217          TGGTGTTCTGGGCGCAGGCCAGGACGACCGCGCACGCTGAGGAGGCCGAAGATCTGATCG
FDC1083         TGGTGTTCTGGGCGCAGGCCAGGACGACCGCGCACGCTGAGGAGGCCGAAGATCTGATCG
JJ10-1          TGGTGTTCTGGGCGCAGGCCAGGACGACCGCGCACGCTGAGGAGGCCGAAGATCTGATCG
JJ238-10        TGGTGTTCTGGGCGCAGGCCAGGACGACCGCGCACGCTGAGGAGGCCGAAGATCTGATCG
JK4-1           TGGTGTTCTGGGCGCAGGCCAGGACGACCGCGCACGCTGAGGAGGCCGAAGATCTGATCG
JW160-1         TGGTGTTCTGGGCGCAGGCCAGGACGACCGCGCACGCTGAGGAGGCCGAAGATCTGATCG
LB100-1         TGGTGTTCTGGGCGCAGGCCAGGACGACCGCGCACGCTGAGGAGGCCGAAGATCTGATCG
LC80            TGGTGTTCTGGGCGCAGGCCAGGACGACCGCGCACGCTGAGGAGGCCGAAGATCTGATCG
LG117           TGGTGTTCTGGGCGCAGGCCAGGACGACCGCGCACGCTGAGGAGGCCGAAGATCTGATCG
LH37-1          TGGTGTTCTGGGCGCAGGCCAGGACGACCGCGCACGCTGAGGAGGCCGAAGATCTGATCG
LMG9322         TGGTGTTCTGGGCGCAGGCCAGGACGACCGCGCACGCTGAGGAGGCCGAAGATCTGATCG
NCPPB3562       TGGTGTTCTGGGCGCAGGCCAGGACGACCGCGCACGCTGAGGAGGCCGAAGATCTGATCG
CFBP2911        TGGTGTTCTGGGCGCAGGCCAGGACGACCGCGCACGCTGAGGAGGCCGAAGATCTGATCG
JF90-2          TGGTGTTCTGGGCGCAGGCCAGGACGACCGCGCACGCTGAGGAGGCCGAAGATCTGATCG
JK2-10          TGGTGTTCTGGGCGCAGGCCAGGACGACCGCGCACGCTGAGGAGGCCGAAGATCTGATCG
JK48            TGGTGTTCTGGGCGCAGGCCAGGACGACCGCGCACGCTGAGGAGGCCGAAGATCTGATCG
JM35            TGGTGTTCTGGGCGCAGGCCAGGACGACCGCGCACGCTGAGGAGGCCGAAGATCTGATCG
JS581           TGGTGTTCTGGGCGCAGGCCAGGACGACCGCGCACGCTGAGGAGGCCGAAGATCTGATCG
JS582           TGGTGTTCTGGGCGCAGGCCAGGACGACCGCGCACGCTGAGGAGGCCGAAGATCTGATCG
JS584           TGGTGTTCTGGGCGCAGGCCAGGACGACCGCGCACGCTGAGGAGGCCGAAGATCTGATCG
LB302           TGGTGTTCTGGGCGCAGGCCAGGACGACCGCGCACGCTGAGGAGGCCGAAGATCTGATCG
LE20-1          TGGTGTTCTGGGCGCAGGCCAGGACGACCGCGCACGCTGAGGAGGCCGAAGATCTGATCG
LG102           TGGTGTTCTGGGCGCAGGCCAGGACGACCGCGCACGCTGAGGAGGCCGAAGATCTGATCG
LG115           TGGTGTTCTGGGCGCAGGCCAGGACGACCGCGCACGCTGAGGAGGCCGAAGATCTGATCG
NCPPB3607       TGGTGTTCTGGGCGCAGGCCAGGACGACCGCGCACGCTGAGGAGGCCGAAGATCTGATCG
NCPPB3608       TGGTGTTCTGGGCGCAGGCCAGGACGACCGCGCACGCTGAGGAGGCCGAAGATCTGATCG
X2003-3218      TGGTGTTCTGGGCGCAGGCCAGGACGACCGCGCACGCTGAGGAGGCCGAAGATCTGATCG
                ************************************************************

JJ238-24        CCGATTTCCTGCGCGGCGGTGAAACAGAAGGCCCTGGCATCGCCGACGGCCAGGCCACCC
JK143-9         CCGATTTCCTGCGCGGCGGTGAAACAGAAGGCCCTGGCATCGCCGACGGCCAGGCCACCC
JK143-11        CCGATTTCCTGCGCGGCGGTGAAACAGAAGGCCCTGGCATCGCCGACGGCCAGGCCACCC
LD71a           CCGATTTCCTGCGCGGCGGTGAAACAGAAGGCCCTGGCATCGCCGACGGCCAGGCCACCC
C40             CCGATTTCCTGCGCGGCGGTGAAACAGAAGGCCCTGGCATCGCCGACGGCCAGGCCACCC
NCPPB3612       CCGATTTCCTGCGCGGCGGTGAAACAGAAGGCCCTGGCATCGCCGACGGCCAGGCCACCC
CFBP2852        CCGATTTCCTGCGCGGCGGTGAAACAGAAGGCCCTGGCATCGCCGACGGCCAGGCCACCC
FDC217          CCGATTTCCTGCGCGGCGGTGAAACAGAAGGCCCTGGCATCGCCGACGGCCAGGCCACCC
FDC1083         CCGATTTCCTGCGCGGCGGTGAAACAGAAGGCCCTGGCATCGCCGACGGCCAGGCCACCC
JJ10-1          CCGATTTCCTGCGCGGCGGTGAAACAGAAGGCCCTGGCATCGCCGACGGCCAGGCCACCC
JJ238-10        CCGATTTCCTGCGCGGCGGTGAAACAGAAGGCCCTGGCATCGCCGACGGCCAGGCCACCC
JK4-1           CCGATTTCCTGCGCGGCGGTGAAACAGAAGGCCCTGGCATCGCCGACGGCCAGGCCACCC
JW160-1         CCGATTTCCTGCGCGGCGGTGAAACAGAAGGCCCTGGCATCGCCGACGGCCAGGCCACCC
LB100-1         CCGATTTCCTGCGCGGCGGTGAAACAGAAGGCCCTGGCATCGCCGACGGCCAGGCCACCC
LC80            CCGATTTCCTGCGCGGCGGTGAAACAGAAGGCCCTGGCATCGCCGACGGCCAGGCCACCC
LG117           CCGATTTCCTGCGCGGCGGTGAAACAGAAGGCCCTGGCATCGCCGACGGCCAGGCCACCC
LH37-1          CCGATTTCCTGCGCGGCGGTGAAACAGAAGGCCCTGGCATCGCCGACGGCCAGGCCACCC
LMG9322         CCGATTTCCTGCGCGGCGGTGAAACAGAAGGCCCTGGCATCGCCGACGGCCAGGCCACCC
NCPPB3562       CCGATTTCCTGCGCGGCGGTGAAACAGAAGGCCCTGGCATCGCCGACGGCCAGGCCACCC
CFBP2911        CCGATTTCCTGCGCGGCGGTGAAACAGAAGGCCCTGGCATCGCCGACGGCCAGGCCACCC
JF90-2          CCGATTTCCTGCGCGGCGGTGAAACAGAAGGCCCTGGCATCGCCGACGGCCAGGCCACCC
JK2-10          CCGATTTCCTGCGCGGCGGTGAAACAGAAGGCCCTGGCATCGCCGACGGCCAGGCCACCC
JK48            CCGATTTCCTGCGCGGCGGTGAAACAGAAGGCCCTGGCATCGCCGACGGCCAGGCCACCC
JM35            CCGATTTCCTGCGCGGCGGTGAAACAGAAGGCCCTGGCATCGCCGACGGCCAGGCCACCC
JS581           CCGATTTCCTGCGCGGCGGTGAAACAGAAGGCCCTGGCATCGCCGACGGCCAGGCCACCC
JS582           CCGATTTCCTGCGCGGCGGTGAAACAGAAGGCCCTGGCATCGCCGACGGCCAGGCCACCC
JS584           CCGATTTCCTGCGCGGCGGTGAAACAGAAGGCCCTGGCATCGCCGACGGCCAGGCCACCC
LB302           CCGATTTCCTGCGCGGCGGTGAAACAGAAGGCCCTGGCATCGCCGACGGCCAGGCCACCC
LE20-1          CCGATTTCCTGCGCGGCGGTGAAACAGAAGGCCCTGGCATCGCCGACGGCCAGGCCACCC
LG102           CCGATTTCCTGCGCGGCGGTGAAACAGAAGGCCCTGGCATCGCCGACGGCCAGGCCACCC
LG115           CCGATTTCCTGCGCGGCGGTGAAACAGAAGGCCCTGGCATCGCCGACGGCCAGGCCACCC
NCPPB3607       CCGATTTCCTGCGCGGCGGTGAAACAGAAGGCCCTGGCATCGCCGACGGCCAGGCCACCC
NCPPB3608       CCGATTTCCTGCGCGGCGGTGAAACAGAAGGCCCTGGCATCGCCGACGGCCAGGCCACCC
X2003-3218      CCGATTTCCTGCGCGGCGGTGAAACAGAAGGCCCTGGCATCGCCGACGGCCAGGCCACCC
                ************************************************************
```

```
JJ238-24        AGCGCCTGCACCTGGCACTGGAGCAGGTGCGACAGGCACGTCCACTACGCGAAATGGACG
JK143-9         AGCGCCTGCACCTGGCACTGGAGCAGGTGCGACAGGCACGTCCACTACGCGAAATGGACG
JK143-11        AGCGCCTGCACCTGGCACTGGAGCAGGTGCGACAGGCACGTCCACTACGCGAAATGGACG
LD71a           AGCGCCTGCACCTGGCACTGGAGCAGGTGCGACAGGCACGTCCACTACGCGAAATGGACG
C40             AGCGCCTGCACCTGGCACTGGAGCAGGTGCGACAGGCACGTCCACTACGCGAAATGGACG
NCPPB3612       AGCGCCTGCACCTGGCACTGGAGCAGGTGCGACAGGCACGTCCACTACGCGAAATGGACG
CFBP2852        AGCGCCTGCACCTGGCACTGGAGCAGGTGCGACAGGCACGTCCACTACGCGAAATGGACG
FDC217          AGCGCCTGCACCTGGCACTGGAGCAGGTGCGACAGGCACGTCCACTACGCGAAATGGACG
FDC1083         AGCGCCTGCACCTGGCACTGGAGCAGGTGCGACAGGCACGTCCACTACGCGAAATGGACG
JJ10-1          AGCGCCTGCACCTGGCACTGGAGCAGGTGCGACAGGCACGTCCACTACGCGAAATGGACG
JJ238-10        AGCGCCTGCACCTGGCACTGGAGCAGGTGCGACAGGCACGTCCACTACGCGAAATGGACG
JK4-1           AGCGCCTGCACCTGGCACTGGAGCAGGTGCGACAGGCACGTCCACTACGCGAAATGGACG
JW160-1         AGCGCCTGCACCTGGCACTGGAGCAGGTGCGACAGGCACGTCCACTACGCGAAATGGACG
LB100-1         AGCGCCTGCACCTGGCACTGGAGCAGGTGCGACAGGCACGTCCACTACGCGAAATGGACG
LC80            AGCGCCTGCACCTGGCACTGGAGCAGGTGCGACAGGCACGTCCACTACGCGAAATGGACG
LG117           AGCGCCTGCACCTGGCACTGGAGCAGGTGCGACAGGCACGTCCACTACGCGAAATGGACG
LH37-1          AGCGCCTGCACCTGGCACTGGAGCAGGTGCGACAGGCACGTCCACTACGCGAAATGGACG
LMG9322         AGCGCCTGCACCTGGCACTGGAGCAGGTGCGACAGGCACGTCCACTACGCGAAATGGACG
NCPPB3562       AGCGCCTGCACCTGGCACTGGAGCAGGTGCGACAGGCACGTCCACTACGCGAAATGGACG
CFBP2911        AGCGCCTGCACCTGGCACTGGAGCAGGTGCGACAGGCACGTCCACTACGCGAAATGGACG
JF90-2          AGCGCCTGCACCTGGCACTGGAGCAGGTGCGACAGGCACGTCCACTACGCGAAATGGACG
JK2-10          AGCGCCTGCACCTGGCACTGGAGCAGGTGCGACAGGCACGTCCACTACGCGAAATGGACG
JK48            AGCGCCTGCACCTGGCACTGGAGCAGGTGCGACAGGCACGTCCACTACGCGAAATGGACG
JM35            AGCGCCTGCACCTGGCACTGGAGCAGGTGCGACAGGCACGTCCACTACGCGAAATGGACG
JS581           AGCGCCTGCACCTGGCACTGGAGCAGGTGCGACAGGCACGTCCACTACGCGAAATGGACG
JS582           AGCGCCTGCACCTGGCACTGGAGCAGGTGCGACAGGCACGTCCACTACGCGAAATGGACG
JS584           AGCGCCTGCACCTGGCACTGGAGCAGGTGCGACAGGCACGTCCACTACGCGAAATGGACG
LB302           AGCGCCTGCACCTGGCACTGGAGCAGGTGCGACAGGCACGTCCACTACGCGAAATGGACG
LE20-1          AGCGCCTGCACCTGGCACTGGAGCAGGTGCGACAGGCACGTCCACTACGCGAAATGGACG
LG102           AGCGCCTGCACCTGGCACTGGAGCAGGTGCGACAGGCACGTCCACTACGCGAAATGGACG
LG115           AGCGCCTGCACCTGGCACTGGAGCAGGTGCGACAGGCACGTCCACTACGCGAAATGGACG
NCPPB3607       AGCGCCTGCACCTGGCACTGGAGCAGGTGCGACAGGCACGTCCACTACGCGAAATGGACG
NCPPB3608       AGCGCCTGCACCTGGCACTGGAGCAGGTGCGACAGGCACGTCCACTACGCGAAATGGACG
X2003-3218      AGCGCCTGCACCTGGCACTGGAGCAGGTGCGACAGGCACGTCCACTACGCGAAATGGACG
                ************************************************************

JJ238-24        ACGCACTGGACGACGACGCCCGCATCTTCGTACTGGGCCTGGCACCCAACGCCTCGCGCC
JK143-9         ACGCACTGGACGACGACGCCCGCATCTTCGTACTGGGCCTGGCACCCAACGCCTCGCGCC
JK143-11        ACGCACTGGACGACGACGCCCGCATCTTCGTACTGGGCCTGGCACCCAACGCCTCGCGCC
LD71a           ACGCACTGGACGACGACGCCCGCATCTTCGTACTGGGCCTGGCACCCAACGCCTCGCGCC
C40             ACGCACTGGACGACGACGCCCGCATCTTCGTACTGGGCCTGGCACCCAACGCCTCGCGCC
NCPPB3612       ACGCACTGGACGACGACGCCCGCATCTTCGTACTGGGCCTGGCACCCAACGCCTCGCGCC
CFBP2852        ACGCACTGGACGACGACGCCCGCATCTTCGTACTGGGCCTGGCACCCAACGCCTCGCGCC
FDC217          ACGCACTGGACGACGACGCCCGCATCTTCGTACTGGGCCTGGCACCCAACGCCTCGCGCC
FDC1083         ACGCACTGGACGACGACGCCCGCATCTTCGTACTGGGCCTGGCACCCAACGCCTCGCGCC
JJ10-1          ACGCACTGGACGACGACGCCCGCATCTTCGTACTGGGCCTGGCACCCAACGCCTCGCGCC
JJ238-10        ACGCACTGGACGACGACGCCCGCATCTTCGTACTGGGCCTGGCACCCAACGCCTCGCGCC
JK4-1           ACGCACTGGACGACGACGCCCGCATCTTCGTACTGGGCCTGGCACCCAACGCCTCGCGCC
JW160-1         ACGCACTGGACGACGACGCCCGCATCTTCGTACTGGGCCTGGCACCCAACGCCTCGCGCC
LB100-1         ACGCACTGGACGACGACGCCCGCATCTTCGTACTGGGCCTGGCACCCAACGCCTCGCGCC
LC80            ACGCACTGGACGACGACGCCCGCATCTTCGTACTGGGCCTGGCACCCAACGCCTCGCGCC
LG117           ACGCACTGGACGACGACGCCCGCATCTTCGTACTGGGCCTGGCACCCAACGCCTCGCGCC
LH37-1          ACGCACTGGACGACGACGCCCGCATCTTCGTACTGGGCCTGGCACCCAACGCCTCGCGCC
LMG9322         ACGCACTGGACGACGACGCCCGCATCTTCGTACTGGGCCTGGCACCCAACGCCTCGCGCC
NCPPB3562       ACGCACTGGACGACGACGCCCGCATCTTCGTACTGGGCCTGGCACCCAACGCCTCGCGCC
CFBP2911        ACGCACTGGACGACGACGCCCGCATCTTCGTACTGGGCCTGGCACCCAACGCCTCGCGCC
JF90-2          ACGCACTGGACGACGACGCCCGCATCTTCGTACTGGGCCTGGCACCCAACGCCTCGCGCC
JK2-10          ACGCACTGGACGACGACGCCCGCATCTTCGTACTGGGCCTGGCACCCAACGCCTCGCGCC
JK48            ACGCACTGGACGACGACGCCCGCATCTTCGTACTGGGCCTGGCACCCAACGCCTCGCGCC
JM35            ACGCACTGGACGACGACGCCCGCATCTTCGTACTGGGCCTGGCACCCAACGCCTCGCGCC
JS581           ACGCACTGGACGACGACGCCCGCATCTTCGTACTGGGCCTGGCACCCAACGCCTCGCGCC
JS582           ACGCACTGGACGACGACGCCCGCATCTTCGTACTGGGCCTGGCACCCAACGCCTCGCGCC
JS584           ACGCACTGGACGACGACGCCCGCATCTTCGTACTGGGCCTGGCACCCAACGCCTCGCGCC
LB302           ACGCACTGGACGACGACGCCCGCATCTTCGTACTGGGCCTGGCACCCAACGCCTCGCGCC
LE20-1          ACGCACTGGACGACGACGCCCGCATCTTCGTACTGGGCCTGGCACCCAACGCCTCGCGCC
LG102           ACGCACTGGACGACGACGCCCGCATCTTCGTACTGGGCCTGGCACCCAACGCCTCGCGCC
LG115           ACGCACTGGACGACGACGCCCGCATCTTCGTACTGGGCCTGGCACCCAACGCCTCGCGCC
NCPPB3607       ACGCACTGGACGACGACGCCCGCATCTTCGTACTGGGCCTGGCACCCAACGCCTCGCGCC
NCPPB3608       ACGCACTGGACGACGACGCCCGCATCTTCGTACTGGGCCTGGCACCCAACGCCTCGCGCC
X2003-3218      ACGCACTGGACGACGACGCCCGCATCTTCGTACTGGGCCTGGCACCCAACGCCTCGCGCC
                ************************************************************
```

```
JJ238-24        TGTCGATCCGCTTCTGGGAAACGCAGACGCTGGCCGGCTTTGCCGCACGGCTGGCTGCCC
JK143-9         TGTCGATCCGCTTCTGGGAAACGCAGACGCTGGCCGGCTTTGCCGCACGGCTGGCTGCCC
JK143-11        TGTCGATCCGCTTCTGGGAAACGCAGACGCTGGCCGGCTTTGCCGCACGGCTGGCTGCCC
LD71a           TGTCGATCCGCTTCTGGGAAACGCAGACGCTGGCCGGCTTTGCCGCACGGCTGGCTGCCC
C40             TGTCGATCCGCTTCTGGGAAACGCAGACGCTGGCCGGCTTTGCCGCACGGCTGGCTGCCC
NCPPB3612       TGTCGATCCGCTTCTGGGAAACGCAGACGCTGGCCGGCTTTGCCGCACGGCTGGCTGCCC
CFBP2852        TGTCGATCCGCTTCTGGGAAACGCAGACGCTGGCCGGCTTTGCCGCACGGCTGGCTGCCC
FDC217          TGTCGATCCGCTTCTGGGAAACGCAGACGCTGGCCGGCTTTGCCGCACGGCTGGCTGCCC
FDC1083         TGTCGATCCGCTTCTGGGAAACGCAGACGCTGGCCGGCTTTGCCGCACGGCTGGCTGCCC
JJ10-1          TGTCGATCCGCTTCTGGGAAACGCAGACGCTGGCCGGCTTTGCCGCACGGCTGGCTGCCC
JJ238-10        TGTCGATCCGCTTCTGGGAAACGCAGACGCTGGCCGGCTTTGCCGCACGGCTGGCTGCCC
JK4-1           TGTCGATCCGCTTCTGGGAAACGCAGACGCTGGCCGGCTTTGCCGCACGGCTGGCTGCCC
JW160-1         TGTCGATCCGCTTCTGGGAAACGCAGACGCTGGCCGGCTTTGCCGCACGGCTGGCTGCCC
LB100-1         TGTCGATCCGCTTCTGGGAAACGCAGACGCTGGCCGGCTTTGCCGCACGGCTGGCTGCCC
LC80            TGTCGATCCGCTTCTGGGAAACGCAGACGCTGGCCGGCTTTGCCGCACGGCTGGCTGCCC
LG117           TGTCGATCCGCTTCTGGGAAACGCAGACGCTGGCCGGCTTTGCCGCACGGCTGGCTGCCC
LH37-1          TGTCGATCCGCTTCTGGGAAACGCAGACGCTGGCCGGCTTTGCCGCACGGCTGGCTGCCC
LMG9322         TGTCGATCCGCTTCTGGGAAACGCAGACGCTGGCCGGCTTTGCCGCACGGCTGGCTGCCC
NCPPB3562       TGTCGATCCGCTTCTGGGAAACGCAGACGCTGGCCGGCTTTGCCGCACGGCTGGCTGCCC
CFBP2911        TGTCGATCCGCTTCTGGGAAACGCAGACGCTGGCCGGCTTTGCCGCACGGCTGGCTGCCC
JF90-2          TGTCGATCCGCTTCTGGGAAACGCAGACGCTGGCCGGCTTTGCCGCACGGCTGGCTGCCC
JK2-10          TGTCGATCCGCTTCTGGGAAACGCAGACGCTGGCCGGCTTTGCCGCACGGCTGGCTGCCC
JK48            TGTCGATCCGCTTCTGGGAAACGCAGACGCTGGCCGGCTTTGCCGCACGGCTGGCTGCCC
JM35            TGTCGATCCGCTTCTGGGAAACGCAGACGCTGGCCGGCTTTGCCGCACGGCTGGCTGCCC
JS581           TGTCGATCCGCTTCTGGGAAACGCAGACGCTGGCCGGCTTTGCCGCACGGCTGGCTGCCC
JS582           TGTCGATCCGCTTCTGGGAAACGCAGACGCTGGCCGGCTTTGCCGCACGGCTGGCTGCCC
JS584           TGTCGATCCGCTTCTGGGAAACGCAGACGCTGGCCGGCTTTGCCGCACGGCTGGCTGCCC
LB302           TGTCGATCCGCTTCTGGGAAACGCAGACGCTGGCCGGCTTTGCCGCACGGCTGGCTGCCC
LE20-1          TGTCGATCCGCTTCTGGGAAACGCAGACGCTGGCCGGCTTTGCCGCACGGCTGGCTGCCC
LG102           TGTCGATCCGCTTCTGGGAAACGCAGACGCTGGCCGGCTTTGCCGCACGGCTGGCTGCCC
LG115           TGTCGATCCGCTTCTGGGAAACGCAGACGCTGGCCGGCTTTGCCGCACGGCTGGCTGCCC
NCPPB3607       TGTCGATCCGCTTCTGGGAAACGCAGACGCTGGCCGGCTTTGCCGCACGGCTGGCTGCCC
NCPPB3608       TGTCGATCCGCTTCTGGGAAACGCAGACGCTGGCCGGCTTTGCCGCACGGCTGGCTGCCC
X2003-3218      TGTCGATCCGCTTCTGGGAAACGCAGACGCTGGCCGGCTTTGCCGCACGGCTGGCTGCCC
                ************************************************************

JJ238-24        ACTATCAGGACCTCAAGCTCGAGCCGCCAGCCTGGAAACGCGCGCCTACCCCGCAGTTTC
JK143-9         ACTATCAGGACCTCAAGCTCGAGCCGCCAGCCTGGAAACGCGCGCCTACCCCGCAGTTTC
JK143-11        ACTATCAGGACCTCAAGCTCGAGCCGCCAGCCTGGAAACGCGCGCCTACCCCGCAGTTTC
LD71a           ACTATCAGGACCTCAAGCTCGAGCCGCCAGCCTGGAAACGCGCGCCTACCCCGCAGTTTC
C40             ACTATCAGGACCTCAAGCTCGAGCCGCCAGCCTGGAAACGCGCGCCTACCCCGCAGTTTC
NCPPB3612       ACTATCAGGACCTCAAGCTCGAGCCGCCAGCCTGGAAACGCGCGCCTACCCCGCAGTTTC
CFBP2852        ACTATCAGGACCTCAAGCTCGAGCCGCCAGCCTGGAAACGCGCGCCTACCCCGCAGTTTC
FDC217          ACTATCAGGACCTCAAGCTCGAGCCGCCAGCCTGGAAACGCGCGCCTACCCCGCAGTTTC
FDC1083         ACTATCAGGACCTCAAGCTCGAGCCGCCAGCCTGGAAACGCGCGCCTACCCCGCAGTTTC
JJ10-1          ACTATCAGGACCTCAAGCTCGAGCCGCCAGCCTGGAAACGCGCGCCTACCCCGCAGTTTC
JJ238-10        ACTATCAGGACCTCAAGCTCGAGCCGCCAGCCTGGAAACGCGCGCCTACCCCGCAGTTTC
JK4-1           ACTATCAGGACCTCAAGCTCGAGCCGCCAGCCTGGAAACGCGCGCCTACCCCGCAGTTTC
JW160-1         ACTATCAGGACCTCAAGCTCGAGCCGCCAGCCTGGAAACGCGCGCCTACCCCGCAGTTTC
LB100-1         ACTATCAGGACCTCAAGCTCGAGCCGCCAGCCTGGAAACGCGCGCCTACCCCGCAGTTTC
LC80            ACTATCAGGACCTCAAGCTCGAGCCGCCAGCCTGGAAACGCGCGCCTACCCCGCAGTTTC
LG117           ACTATCAGGACCTCAAGCTCGAGCCGCCAGCCTGGAAACGCGCGCCTACCCCGCAGTTTC
LH37-1          ACTATCAGGACCTCAAGCTCGAGCCGCCAGCCTGGAAACGCGCGCCTACCCCGCAGTTTC
LMG9322         ACTATCAGGACCTCAAGCTCGAGCCGCCAGCCTGGAAACGCGCGCCTACCCCGCAGTTTC
NCPPB3562       ACTATCAGGACCTCAAGCTCGAGCCGCCAGCCTGGAAACGCGCGCCTACCCCGCAGTTTC
CFBP2911        ACTATCAGGACCTCAAGCTCGAGCCGCCAGCCTGGAAACGCGCGCCTACCCCGCAGTTTC
JF90-2          ACTATCAGGACCTCAAGCTCGAGCCGCCAGCCTGGAAACGCGCGCCTACCCCGCAGTTTC
JK2-10          ACTATCAGGACCTCAAGCTCGAGCCGCCAGCCTGGAAACGCGCGCCTACCCCGCAGTTTC
JK48            ACTATCAGGACCTCAAGCTCGAGCCGCCAGCCTGGAAACGCGCGCCTACCCCGCAGTTTC
JM35            ACTATCAGGACCTCAAGCTCGAGCCGCCAGCCTGGAAACGCGCGCCTACCCCGCAGTTTC
JS581           ACTATCAGGACCTCAAGCTCGAGCCGCCAGCCTGGAAACGCGCGCCTACCCCGCAGTTTC
JS582           ACTATCAGGACCTCAAGCTCGAGCCGCCAGCCTGGAAACGCGCGCCTACCCCGCAGTTTC
JS584           ACTATCAGGACCTCAAGCTCGAGCCGCCAGCCTGGAAACGCGCGCCTACCCCGCAGTTTC
LB302           ACTATCAGGACCTCAAGCTCGAGCCGCCAGCCTGGAAACGCGCGCCTACCCCGCAGTTTC
LE20-1          ACTATCAGGACCTCAAGCTCGAGCCGCCAGCCTGGAAACGCGCGCCTACCCCGCAGTTTC
LG102           ACTATCAGGACCTCAAGCTCGAGCCGCCAGCCTGGAAACGCGCGCCTACCCCGCAGTTTC
LG115           ACTATCAGGACCTCAAGCTCGAGCCGCCAGCCTGGAAACGCGCGCCTACCCCGCAGTTTC
NCPPB3607       ACTATCAGGACCTCAAGCTCGAGCCGCCAGCCTGGAAACGCGCGCCTACCCCGCAGTTTC
NCPPB3608       ACTATCAGGACCTCAAGCTCGAGCCGCCAGCCTGGAAACGCGCGCCTACCCCGCAGTTTC
X2003-3218      ACTATCAGGACCTCAAGCTCGAGCCGCCAGCCTGGAAACGCGCGCCTACCCCGCAGTTTC
                ************************************************************
```

```
JJ238-24       TCGCCCTGCAGACAGCGCCGGTCTACGGCGAGCACGGCAAGCCCAAGGCCGAGGACGTCT
JK143-9        TCGCCCTGCAGACAGCGCCGGTCTACGGCGAGCACGGCAAGCCCAAGGCCGAGGACGTCT
JK143-11       TCGCCCTGCAGACAGCGCCGGTCTACGGCGAGCACGGCAAGCCCAAGGCCGAGGACGTCT
LD71a          TCGCCCTGCAGACAGCGCCGGTCTACGGCGAGCACGGCAAGCCCAAGGCCGAGGACGTCT
C40            TCGCCCTGCAGACAGCGCCGGTCTACGGCGAGCACGGCAAGCCCAAGGCCGAGGACGTCT
NCPPB3612      TCGCCCTGCAGACAGCGCCGGTCTACGGCGAGCACGGCAAGCCCAAGGCCGAGGACGTCT
CFBP2852       TCGCCCTGCAGACAGCGCCGGTCTACGGCGAGCACGGCAAGCCCAAGGCCGAGGACGTCT
FDC217         TCGCCCTGCAGACAGCGCCGGTCTACGGCGAGCACGGCAAGCCCAAGGCCGAGGACGTCT
FDC1083        TCGCCCTGCAGACAGCGCCGGTCTACGGCGAGCACGGCAAGCCCAAGGCCGAGGACGTCT
JJ10-1         TCGCCCTGCAGACAGCGCCGGTCTACGGCGAGCACGGCAAGCCCAAGGCCGAGGACGTCT
JJ238-10       TCGCCCTGCAGACAGCGCCGGTCTACGGCGAGCACGGCAAGCCCAAGGCCGAGGACGTCT
JK4-1          TCGCCCTGCAGACAGCGCCGGTCTACGGCGAGCACGGCAAGCCCAAGGCCGAGGACGTCT
JW160-1        TCGCCCTGCAGACAGCGCCGGTCTACGGCGAGCACGGCAAGCCCAAGGCCGAGGACGTCT
LB100-1        TCGCCCTGCAGACAGCGCCGGTCTACGGCGAGCACGGCAAGCCCAAGGCCGAGGACGTCT
LC80           TCGCCCTGCAGACAGCGCCGGTCTACGGCGAGCACGGCAAGCCCAAGGCCGAGGACGTCT
LG117          TCGCCCTGCAGACAGCGCCGGTCTACGGCGAGCACGGCAAGCCCAAGGCCGAGGACGTCT
LH37-1         TCGCCCTGCAGACAGCGCCGGTCTACGGCGAGCACGGCAAGCCCAAGGCCGAGGACGTCT
LMG9322        TCGCCCTGCAGACAGCGCCGGTCTACGGCGAGCACGGCAAGCCCAAGGCCGAGGACGTCT
NCPPB3562      TCGCCCTGCAGACAGCGCCGGTCTACGGCGAGCACGGCAAGCCCAAGGCCGAGGACGTCT
CFBP2911       TCGCCCTGCAGACAGCGCCGGTCTACGGCGAGCACGGCAAGCCCAAGGCCGAGGACGTCT
JF90-2         TCGCCCTGCAGACAGCGCCGGTCTACGGCGAGCACGGCAAGCCCAAGGCCGAGGACGTCT
JK2-10         TCGCCCTGCAGACAGCGCCGGTCTACGGCGAGCACGGCAAGCCCAAGGCCGAGGACGTCT
JK48           TCGCCCTGCAGACAGCGCCGGTCTACGGCGAGCACGGCAAGCCCAAGGCCGAGGACGTCT
JM35           TCGCCCTGCAGACAGCGCCGGTCTACGGCGAGCACGGCAAGCCCAAGGCCGAGGACGTCT
JS581          TCGCCCTGCAGACAGCGCCGGTCTACGGCGAGCACGGCAAGCCCAAGGCCGAGGACGTCT
JS582          TCGCCCTGCAGACAGCGCCGGTCTACGGCGAGCACGGCAAGCCCAAGGCCGAGGACGTCT
JS584          TCGCCCTGCAGACAGCGCCGGTCTACGGCGAGCACGGCAAGCCCAAGGCCGAGGACGTCT
LB302          TCGCCCTGCAGACAGCGCCGGTCTACGGCGAGCACGGCAAGCCCAAGGCCGAGGACGTCT
LE20-1         TCGCCCTGCAGACAGCGCCGGTCTACGGCGAGCACGGCAAGCCCAAGGCCGAGGACGTCT
LG102          TCGCCCTGCAGACAGCGCCGGTCTACGGCGAGCACGGCAAGCCCAAGGCCGAGGACGTCT
LG115          TCGCCCTGCAGACAGCGCCGGTCTACGGCGAGCACGGCAAGCCCAAGGCCGAGGACGTCT
NCPPB3607      TCGCCCTGCAGACAGCGCCGGTCTACGGCGAGCACGGCAAGCCCAAGGCCGAGGACGTCT
NCPPB3608      TCGCCCTGCAGACAGCGCCGGTCTACGGCGAGCACGGCAAGCCCAAGGCCGAGGACGTCT
X2003-3218     TCGCCCTGCAGACAGCGCCGGTCTACGGCGAGCACGGCAAGCCCAAGGCCGAGGACGTCT
               ************************************************************

JJ238-24       CGCCGCTTCTGGCAGGGGAGCTGACCCGCGCCATCCTCGCCGGCACCCGCTATCCGCGCA
JK143-9        CGCCGCTTCTGGCAGGGGAGCTGACCCGCGCCATCCTCGCCGGCACCCGCTATCCGCGCA
JK143-11       CGCCGCTTCTGGCAGGGGAGCTGACCCGCGCCATCCTCGCCGGCACCCGCTATCCGCGCA
LD71a          CGCCGCTTCTGGCAGGGGAGCTGACCCGCGCCATCCTCGCCGGCACCCGCTATCCGCGCA
C40            CGCCGCTTCTGGCAGGGGAGCTGACCCGCGCCATCCTCGCCGGCACCCGCTATCCGCGCA
NCPPB3612      CGCCGCTTCTGGCAGGGGAGCTGACCCGCGCCATCCTCGCCGGCACCCGCTATCCGCGCA
CFBP2852       CGCCGCTTCTGGCAGGGGAGCTGACCCGCGCCATCCTCGCCGGCACCCGCTATCCGCGCA
FDC217         CGCCGCTTCTGGCAGGGGAGCTGACCCGCGCCATCCTCGCCGGCACCCGCTATCCGCGCA
FDC1083        CGCCGCTTCTGGCAGGGGAGCTGACCCGCGCCATCCTCGCCGGCACCCGCTATCCGCGCA
JJ10-1         CGCCGCTTCTGGCAGGGGAGCTGACCCGCGCCATCCTCGCCGGCACCCGCTATCCGCGCA
JJ238-10       CGCCGCTTCTGGCAGGGGAGCTGACCCGCGCCATCCTCGCCGGCACCCGCTATCCGCGCA
JK4-1          CGCCGCTTCTGGCAGGGGAGCTGACCCGCGCCATCCTCGCCGGCACCCGCTATCCGCGCA
JW160-1        CGCCGCTTCTGGCAGGGGAGCTGACCCGCGCCATCCTCGCCGGCACCCGCTATCCGCGCA
LB100-1        CGCCGCTTCTGGCAGGGGAGCTGACCCGCGCCATCCTCGCCGGCACCCGCTATCCGCGCA
LC80           CGCCGCTTCTGGCAGGGGAGCTGACCCGCGCCATCCTCGCCGGCACCCGCTATCCGCGCA
LG117          CGCCGCTTCTGGCAGGGGAGCTGACCCGCGCCATCCTCGCCGGCACCCGCTATCCGCGCA
LH37-1         CGCCGCTTCTGGCAGGGGAGCTGACCCGCGCCATCCTCGCCGGCACCCGCTATCCGCGCA
LMG9322        CGCCGCTTCTGGCAGGGGAGCTGACCCGCGCCATCCTCGCCGGCACCCGCTATCCGCGCA
NCPPB3562      CGCCGCTTCTGGCAGGGGAGCTGACCCGCGCCATCCTCGCCGGCACCCGCTATCCGCGCA
CFBP2911       CGCCGCTTCTGGCAGGGGAGCTGACCCGCGCCATCCTCGCCGGCACCCGCTATCCGCGCA
JF90-2         CGCCGCTTCTGGCAGGGGAGCTGACCCGCGCCATCCTCGCCGGCACCCGCTATCCGCGCA
JK2-10         CGCCGCTTCTGGCAGGGGAGCTGACCCGCGCCATCCTCGCCGGCACCCGCTATCCGCGCA
JK48           CGCCGCTTCTGGCAGGGGAGCTGACCCGCGCCATCCTCGCCGGCACCCGCTATCCGCGCA
JM35           CGCCGCTTCTGGCAGGGGAGCTGACCCGCGCCATCCTCGCCGGCACCCGCTATCCGCGCA
JS581          CGCCGCTTCTGGCAGGGGAGCTGACCCGCGCCATCCTCGCCGGCACCCGCTATCCGCGCA
JS582          CGCCGCTTCTGGCAGGGGAGCTGACCCGCGCCATCCTCGCCGGCACCCGCTATCCGCGCA
JS584          CGCCGCTTCTGGCAGGGGAGCTGACCCGCGCCATCCTCGCCGGCACCCGCTATCCGCGCA
LB302          CGCCGCTTCTGGCAGGGGAGCTGACCCGCGCCATCCTCGCCGGCACCCGCTATCCGCGCA
LE20-1         CGCCGCTTCTGGCAGGGGAGCTGACCCGCGCCATCCTCGCCGGCACCCGCTATCCGCGCA
LG102          CGCCGCTTCTGGCAGGGGAGCTGACCCGCGCCATCCTCGCCGGCACCCGCTATCCGCGCA
LG115          CGCCGCTTCTGGCAGGGGAGCTGACCCGCGCCATCCTCGCCGGCACCCGCTATCCGCGCA
NCPPB3607      CGCCGCTTCTGGCAGGGGAGCTGACCCGCGCCATCCTCGCCGGCACCCGCTATCCGCGCA
NCPPB3608      CGCCGCTTCTGGCAGGGGAGCTGACCCGCGCCATCCTCGCCGGCACCCGCTATCCGCGCA
X2003-3218     CGCCGCTTCTGGCAGGGGAGCTGACCCGCGCCATCCTCGCCGGCACCCGCTATCCGCGCA
               ************************************************************
```

```
JJ238-24        GCCTGCTCGGCGGCATCGTCATGCGCTTCCGCGCCGATGGGCAGGTCAACCCGCTGCGCG
JK143-9         GCCTGCTCGGCGGCATCGTCATGCGCTTCCGCGCCGATGGGCAGGTCAACCCGCTGCGCG
JK143-11        GCCTGCTCGGCGGCATCGTCATGCGCTTCCGCGCCGATGGGCAGGTCAACCCGCTGCGCG
LD71a           GCCTGCTCGGCGGCATCGTCATGCGCTTCCGCGCCGATGGGCAGGTCAACCCGCTGCGCG
C40             GCCTGCTCGGCGGCATCGTCATGCGCTTCCGCGCCGATGGGCAGGTCAACCCGCTGCGCG
NCPPB3612       GCCTGCTCGGCGGCATCGTCATGCGCTTCCGCGCCGATGGGCAGGTCAACCCGCTGCGCG
CFBP2852        GCCTGCTCGGCGGCATCGTCATGCGCTTCCGCGCCGATGGGCAGGTCAACCCGCTGCGCG
FDC217          GCCTGCTCGGCGGCATCGTCATGCGCTTCCGCGCCGATGGGCAGGTCAACCCGCTGCGCG
FDC1083         GCCTGCTCGGCGGCATCGTCATGCGCTTCCGCGCCGATGGGCAGGTCAACCCGCTGCGCG
JJ10-1          GCCTGCTCGGCGGCATCGTCATGCGCTTCCGCGCCGATGGGCAGGTCAACCCGCTGCGCG
JJ238-10        GCCTGCTCGGCGGCATCGTCATGCGCTTCCGCGCCGATGGGCAGGTCAACCCGCTGCGCG
JK4-1           GCCTGCTCGGCGGCATCGTCATGCGCTTCCGCGCCGATGGGCAGGTCAACCCGCTGCGCG
JW160-1         GCCTGCTCGGCGGCATCGTCATGCGCTTCCGCGCCGATGGGCAGGTCAACCCGCTGCGCG
LB100-1         GCCTGCTCGGCGGCATCGTCATGCGCTTCCGCGCCGATGGGCAGGTCAACCCGCTGCGCG
LC80            GCCTGCTCGGCGGCATCGTCATGCGCTTCCGCGCCGATGGGCAGGTCAACCCGCTGCGCG
LG117           GCCTGCTCGGCGGCATCGTCATGCGCTTCCGCGCCGATGGGCAGGTCAACCCGCTGCGCG
LH37-1          GCCTGCTCGGCGGCATCGTCATGCGCTTCCGCGCCGATGGGCAGGTCAACCCGCTGCGCG
LMG9322         GCCTGCTCGGCGGCATCGTCATGCGCTTCCGCGCCGATGGGCAGGTCAACCCGCTGCGCG
NCPPB3562       GCCTGCTCGGCGGCATCGTCATGCGCTTCCGCGCCGATGGGCAGGTCAACCCGCTGCGCG
CFBP2911        GCCTGCTCGGCGGCATCGTCATGCGCTTCCGCGCCGATGGGCAGGTCAACCCGCTGCGCG
JF90-2          GCCTGCTCGGCGGCATCGTCATGCGCTTCCGCGCCGATGGGCAGGTCAACCCGCTGCGCG
JK2-10          GCCTGCTCGGCGGCATCGTCATGCGCTTCCGCGCCGATGGGCAGGTCAACCCGCTGCGCG
JK48            GCCTGCTCGGCGGCATCGTCATGCGCTTCCGCGCCGATGGGCAGGTCAACCCGCTGCGCG
JM35            GCCTGCTCGGCGGCATCGTCATGCGCTTCCGCGCCGATGGGCAGGTCAACCCGCTGCGCG
JS581           GCCTGCTCGGCGGCATCGTCATGCGCTTCCGCGCCGATGGGCAGGTCAACCCGCTGCGCG
JS582           GCCTGCTCGGCGGCATCGTCATGCGCTTCCGCGCCGATGGGCAGGTCAACCCGCTGCGCG
JS584           GCCTGCTCGGCGGCATCGTCATGCGCTTCCGCGCCGATGGGCAGGTCAACCCGCTGCGCG
LB302           GCCTGCTCGGCGGCATCGTCATGCGCTTCCGCGCCGATGGGCAGGTCAACCCGCTGCGCG
LE20-1          GCCTGCTCGGCGGCATCGTCATGCGCTTCCGCGCCGATGGGCAGGTCAACCCGCTGCGCG
LG102           GCCTGCTCGGCGGCATCGTCATGCGCTTCCGCGCCGATGGGCAGGTCAACCCGCTGCGCG
LG115           GCCTGCTCGGCGGCATCGTCATGCGCTTCCGCGCCGATGGGCAGGTCAACCCGCTGCGCG
NCPPB3607       GCCTGCTCGGCGGCATCGTCATGCGCTTCCGCGCCGATGGGCAGGTCAACCCGCTGCGCG
NCPPB3608       GCCTGCTCGGCGGCATCGTCATGCGCTTCCGCGCCGATGGGCAGGTCAACCCGCTGCGCG
X2003-3218      GCCTGCTCGGCGGCATCGTCATGCGCTTCCGCGCCGATGGGCAGGTCAACCCGCTGCGCG
                ************************************************************

JJ238-24        TGGCCTTGTGCCGGGCAGTACTGGCACGCGAGGCACGTCTGGACACGCAACAAGGACTGT
JK143-9         TGGCCTTGTGCCGGGCAGTACTGGCACGCGAGGCACGTCTGGACACGCAACAAGGACTGT
JK143-11        TGGCCTTGTGCCGGGCAGTACTGGCACGCGAGGCACGTCTGGACACGCAACAAGGACTGT
LD71a           TGGCCTTGTGCCGGGCAGTACTGGCACGCGAGGCACGTCTGGACACGCAACAAGGACTGT
C40             TGGCCTTGTGCCGGGCAGTACTGGCACGCGAGGCACGTCTGGACACGCAACAAGGACTGT
NCPPB3612       TGGCCTTGTGCCGGGCAGTACTGGCACGCGAGGCACGTCTGGACACGCAACAAGGACTGT
CFBP2852        TGGCCTTGTGCCGGGCAGTACTGGCACGCGAGGCACGTCTGGACACGCAACAAGGACTGT
FDC217          TGGCCTTGTGCCGGGCAGTACTGGCACGCGAGGCACGTCTGGACACGCAACAAGGACTGT
FDC1083         TGGCCTTGTGCCGGGCAGTACTGGCACGCGAGGCACGTCTGGACACGCAACAAGGACTGT
JJ10-1          TGGCCTTGTGCCGGGCAGTACTGGCACGCGAGGCACGTCTGGACACGCAACAAGGACTGT
JJ238-10        TGGCCTTGTGCCGGGCAGTACTGGCACGCGAGGCACGTCTGGACACGCAACAAGGACTGT
JK4-1           TGGCCTTGTGCCGGGCAGTACTGGCACGCGAGGCACGTCTGGACACGCAACAAGGACTGT
JW160-1         TGGCCTTGTGCCGGGCAGTACTGGCACGCGAGGCACGTCTGGACACGCAACAAGGACTGT
LB100-1         TGGCCTTGTGCCGGGCAGTACTGGCACGCGAGGCACGTCTGGACACGCAACAAGGACTGT
LC80            TGGCCTTGTGCCGGGCAGTACTGGCACGCGAGGCACGTCTGGACACGCAACAAGGACTGT
LG117           TGGCCTTGTGCCGGGCAGTACTGGCACGCGAGGCACGTCTGGACACGCAACAAGGACTGT
LH37-1          TGGCCTTGTGCCGGGCAGTACTGGCACGCGAGGCACGTCTGGACACGCAACAAGGACTGT
LMG9322         TGGCCTTGTGCCGGGCAGTACTGGCACGCGAGGCACGTCTGGACACGCAACAAGGACTGT
NCPPB3562       TGGCCTTGTGCCGGGCAGTACTGGCACGCGAGGCACGTCTGGACACGCAACAAGGACTGT
CFBP2911        TGGCCTTGTGCCGGGCAGTACTGGCACGCGAGGCACGTCTGGACACGCAACAAGGACTGT
JF90-2          TGGCCTTGTGCCGGGCAGTACTGGCACGCGAGGCACGTCTGGACACGCAACAAGGACTGT
JK2-10          TGGCCTTGTGCCGGGCAGTACTGGCACGCGAGGCACGTCTGGACACGCAACAAGGACTGT
JK48            TGGCCTTGTGCCGGGCAGTACTGGCACGCGAGGCACGTCTGGACACGCAACAAGGACTGT
JM35            TGGCCTTGTGCCGGGCAGTACTGGCACGCGAGGCACGTCTGGACACGCAACAAGGACTGT
JS581           TGGCCTTGTGCCGGGCAGTACTGGCACGCGAGGCACGTCTGGACACGCAACAAGGACTGT
JS582           TGGCCTTGTGCCGGGCAGTACTGGCACGCGAGGCACGTCTGGACACGCAACAAGGACTGT
JS584           TGGCCTTGTGCCGGGCAGTACTGGCACGCGAGGCACGTCTGGACACGCAACAAGGACTGT
LB302           TGGCCTTGTGCCGGGCAGTACTGGCACGCGAGGCACGTCTGGACACGCAACAAGGACTGT
LE20-1          TGGCCTTGTGCCGGGCAGTACTGGCACGCGAGGCACGTCTGGACACGCAACAAGGACTGT
LG102           TGGCCTTGTGCCGGGCAGTACTGGCACGCGAGGCACGTCTGGACACGCAACAAGGACTGT
LG115           TGGCCTTGTGCCGGGCAGTACTGGCACGCGAGGCACGTCTGGACACGCAACAAGGACTGT
NCPPB3607       TGGCCTTGTGCCGGGCAGTACTGGCACGCGAGGCACGTCTGGACACGCAACAAGGACTGT
NCPPB3608       TGGCCTTGTGCCGGGCAGTACTGGCACGCGAGGCACGTCTGGACACGCAACAAGGACTGT
X2003-3218      TGGCCTTGTGCCGGGCAGTACTGGCACGCGAGGCACGTCTGGACACGCAACAAGGACTGT
                ************************************************************
```

```
JJ238-24        CATCCACCAAGGGAGAGCCCCCCGTGAGTCTCGATACCGCCAATACCGACCCCGGCTACT
JK143-9         CATCCACCAAGGGAGAGCCCCCCGTGAGTCTCGATACCGCCAATACCGACCCCGGCTACT
JK143-11        CATCCACCAAGGGAGAGCCCCCCGTGAGTCTCGATACCGCCAATACCGACCCCGGCTACT
LD71a           CATCCACCAAGGGAGAGCCCCCCGTGAGTCTCGATACCGCCAATACCGACCCCGGCTACT
C40             CATCCACCAAGGGAGAGCCCCCCGTGAGTCTCGATACCGCCAATACCGACCCCGGCTACT
NCPPB3612       CATCCACCAAGGGAGAGCCCCCCGTGAGTCTCGATACCGCCAATACCGACCCCGGCTACT
CFBP2852        CATCCACCAAGGGAGAGCCCCCCGTGAGTCTCGATACCGCCAATACCGACCCCGGCTACT
FDC217          CATCCACCAAGGGAGAGCCCCCCGTGAGTCTCGATACCGCCAATACCGACCCCGGCTACT
FDC1083         CATCCACCAAGGGAGAGCCCCCCGTGAGTCTCGATACCGCCAATACCGACCCCGGCTACT
JJ10-1          CATCCACCAAGGGAGAGCCCCCCGTGAGTCTCGATACCGCCAATACCGACCCCGGCTACT
JJ238-10        CATCCACCAAGGGAGAGCCCCCCGTGAGTCTCGATACCGCCAATACCGACCCCGGCTACT
JK4-1           CATCCACCAAGGGAGAGCCCCCCGTGAGTCTCGATACCGCCAATACCGACCCCGGCTACT
JW160-1         CATCCACCAAGGGAGAGCCCCCCGTGAGTCTCGATACCGCCAATACCGACCCCGGCTACT
LB100-1         CATCCACCAAGGGAGAGCCCCCCGTGAGTCTCGATACCGCCAATACCGACCCCGGCTACT
LC80            CATCCACCAAGGGAGAGCCCCCCGTGAGTCTCGATACCGCCAATACCGACCCCGGCTACT
LG117           CATCCACCAAGGGAGAGCCCCCCGTGAGTCTCGATACCGCCAATACCGACCCCGGCTACT
LH37-1          CATCCACCAAGGGAGAGCCCCCCGTGAGTCTCGATACCGCCAATACCGACCCCGGCTACT
LMG9322         CATCCACCAAGGGAGAGCCCCCCGTGAGTCTCGATACCGCCAATACCGACCCCGGCTACT
NCPPB3562       CATCCACCAAGGGAGAGCCCCCCGTGAGTCTCGATACCGCCAATACCGACCCCGGCTACT
CFBP2911        CATCCACCAAGGGAGAGCCCCCCGTGAGTCTCGATACCGCCAATACCGACCCCGGCTACT
JF90-2          CATCCACCAAGGGAGAGCCCCCCGTGAGTCTCGATACCGCCAATACCGACCCCGGCTACT
JK2-10          CATCCACCAAGGGAGAGCCCCCCGTGAGTCTCGATACCGCCAATACCGACCCCGGCTACT
JK48            CATCCACCAAGGGAGAGCCCCCCGTGAGTCTCGATACCGCCAATACCGACCCCGGCTACT
JM35            CATCCACCAAGGGAGAGCCCCCCGTGAGTCTCGATACCGCCAATACCGACCCCGGCTACT
JS581           CATCCACCAAGGGAGAGCCCCCCGTGAGTCTCGATACCGCCAATACCGACCCCGGCTACT
JS582           CATCCACCAAGGGAGAGCCCCCCGTGAGTCTCGATACCGCCAATACCGACCCCGGCTACT
JS584           CATCCACCAAGGGAGAGCCCCCCGTGAGTCTCGATACCGCCAATACCGACCCCGGCTACT
LB302           CATCCACCAAGGGAGAGCCCCCCGTGAGTCTCGATACCGCCAATACCGACCCCGGCTACT
LE20-1          CATCCACCAAGGGAGAGCCCCCCGTGAGTCTCGATACCGCCAATACCGACCCCGGCTACT
LG102           CATCCACCAAGGGAGAGCCCCCCGTGAGTCTCGATACCGCCAATACCGACCCCGGCTACT
LG115           CATCCACCAAGGGAGAGCCCCCCGTGAGTCTCGATACCGCCAATACCGACCCCGGCTACT
NCPPB3607       CATCCACCAAGGGAGAGCCCCCCGTGAGTCTCGATACCGCCAATACCGACCCCGGCTACT
NCPPB3608       CATCCACCAAGGGAGAGCCCCCCGTGAGTCTCGATACCGCCAATACCGACCCCGGCTACT
X2003-3218      CATCCACCAAGGGAGAGCCCCCCGTGAGTCTCGATACCGCCAATACCGACCCCGGCTACT
                ************************************************************

JJ238-24        TGCTGGGCCGGCTGTTCTCATCGCTGGAAAACCTGCAACGCACCGCACTGGGCGGGCAGG
JK143-9         TGCTGGGCCGGCTGTTCTCATCGCTGGAAAACCTGCAACGCACCGCACTGGGCGGGCAGG
JK143-11        TGCTGGGCCGGCTGTTCTCATCGCTGGAAAACCTGCAACGCACCGCACTGGGCGGGCAGG
LD71a           TGCTGGGCCGGCTGTTCTCATCGCTGGAAAACCTGCAACGCACCGCACTGGGCGGGCAGG
C40             TGCTGGGCCGGCTGTTCTCATCGCTGGAAAACCTGCAACGCACCGCACTGGGCGGGCAGG
NCPPB3612       TGCTGGGCCGGCTGTTCTCATCGCTGGAAAACCTGCAACGCACCGCACTGGGCGGGCAGG
CFBP2852        TGCTGGGCCGGCTGTTCTCATCGCTGGAAAACCTGCAACGCACCGCACTGGGCGGGCAGG
FDC217          TGCTGGGCCGGCTGTTCTCATCGCTGGAAAACCTGCAACGCACCGCACTGGGCGGGCAGG
FDC1083         TGCTGGGCCGGCTGTTCTCATCGCTGGAAAACCTGCAACGCACCGCACTGGGCGGGCAGG
JJ10-1          TGCTGGGCCGGCTGTTCTCATCGCTGGAAAACCTGCAACGCACCGCACTGGGCGGGCAGG
JJ238-10        TGCTGGGCCGGCTGTTCTCATCGCTGGAAAACCTGCAACGCACCGCACTGGGCGGGCAGG
JK4-1           TGCTGGGCCGGCTGTTCTCATCGCTGGAAAACCTGCAACGCACCGCACTGGGCGGGCAGG
JW160-1         TGCTGGGCCGGCTGTTCTCATCGCTGGAAAACCTGCAACGCACCGCACTGGGCGGGCAGG
LB100-1         TGCTGGGCCGGCTGTTCTCATCGCTGGAAAACCTGCAACGCACCGCACTGGGCGGGCAGG
LC80            TGCTGGGCCGGCTGTTCTCATCGCTGGAAAACCTGCAACGCACCGCACTGGGCGGGCAGG
LG117           TGCTGGGCCGGCTGTTCTCATCGCTGGAAAACCTGCAACGCACCGCACTGGGCGGGCAGG
LH37-1          TGCTGGGCCGGCTGTTCTCATCGCTGGAAAACCTGCAACGCACCGCACTGGGCGGGCAGG
LMG9322         TGCTGGGCCGGCTGTTCTCATCGCTGGAAAACCTGCAACGCACCGCACTGGGCGGGCAGG
NCPPB3562       TGCTGGGCCGGCTGTTCTCATCGCTGGAAAACCTGCAACGCACCGCACTGGGCGGGCAGG
CFBP2911        TGCTGGGCCGGCTGTTCTCATCGCTGGAAAACCTGCAACGCACCGCACTGGGCGGGCAGG
JF90-2          TGCTGGGCCGGCTGTTCTCATCGCTGGAAAACCTGCAACGCACCGCACTGGGCGGGCAGG
JK2-10          TGCTGGGCCGGCTGTTCTCATCGCTGGAAAACCTGCAACGCACCGCACTGGGCGGGCAGG
JK48            TGCTGGGCCGGCTGTTCTCATCGCTGGAAAACCTGCAACGCACCGCACTGGGCGGGCAGG
JM35            TGCTGGGCCGGCTGTTCTCATCGCTGGAAAACCTGCAACGCACCGCACTGGGCGGGCAGG
JS581           TGCTGGGCCGGCTGTTCTCATCGCTGGAAAACCTGCAACGCACCGCACTGGGCGGGCAGG
JS582           TGCTGGGCCGGCTGTTCTCATCGCTGGAAAACCTGCAACGCACCGCACTGGGCGGGCAGG
JS584           TGCTGGGCCGGCTGTTCTCATCGCTGGAAAACCTGCAACGCACCGCACTGGGCGGGCAGG
LB302           TGCTGGGCCGGCTGTTCTCATCGCTGGAAAACCTGCAACGCACCGCACTGGGCGGGCAGG
LE20-1          TGCTGGGCCGGCTGTTCTCATCGCTGGAAAACCTGCAACGCACCGCACTGGGCGGGCAGG
LG102           TGCTGGGCCGGCTGTTCTCATCGCTGGAAAACCTGCAACGCACCGCACTGGGCGGGCAGG
LG115           TGCTGGGCCGGCTGTTCTCATCGCTGGAAAACCTGCAACGCACCGCACTGGGCGGGCAGG
NCPPB3607       TGCTGGGCCGGCTGTTCTCATCGCTGGAAAACCTGCAACGCACCGCACTGGGCGGGCAGG
NCPPB3608       TGCTGGGCCGGCTGTTCTCATCGCTGGAAAACCTGCAACGCACCGCACTGGGCGGGCAGG
X2003-3218      TGCTGGGCCGGCTGTTCTCATCGCTGGAAAACCTGCAACGCACCGCACTGGGCGGGCAGG
                ************************************************************
```

```
JJ238-24        TCAACGCCACCATCCGCGACCGTTACTACGGCGCCGCCTCTGCAACCCCGGCGAGCATTT
JK143-9         TCAACGCCACCATCCGCGACCGTTACTACGGCGCCGCCTCTGCAACCCCGGCGAGCATTT
JK143-11        TCAACGCCACCATCCGCGACCGTTACTACGGCGCCGCCTCTGCAACCCCGGCGAGCATTT
LD71a           TCAACGCCACCATCCGCGACCGTTACTACGGCGCCGCCTCTGCAACCCCGGCGAGCATTT
C40             TCAACGCCACCATCCGCGACCGTTACTACGGCGCCGCCTCTGCAACCCCGGCGAGCATTT
NCPPB3612       TCAACGCCACCATCCGCGACCGTTACTACGGCGCCGCCTCTGCAACCCCGGCGAGCATTT
CFBP2852        TCAACGCCACCATCCGCGACCGTTACTACGGCGCCGCCTCTGCAACCCCGGCGAGCATTT
FDC217          TCAACGCCACCATCCGCGACCGTTACTACGGCGCCGCCTCTGCAACCCCGGCGAGCATTT
FDC1083         TCAACGCCACCATCCGCGACCGTTACTACGGCGCCGCCTCTGCAACCCCGGCGAGCATTT
JJ10-1          TCAACGCCACCATCCGCGACCGTTACTACGGCGCCGCCTCTGCAACCCCGGCGAGCATTT
JJ238-10        TCAACGCCACCATCCGCGACCGTTACTACGGCGCCGCCTCTGCAACCCCGGCGAGCATTT
JK4-1           TCAACGCCACCATCCGCGACCGTTACTACGGCGCCGCCTCTGCAACCCCGGCGAGCATTT
JW160-1         TCAACGCCACCATCCGCGACCGTTACTACGGCGCCGCCTCTGCAACCCCGGCGAGCATTT
LB100-1         TCAACGCCACCATCCGCGACCGTTACTACGGCGCCGCCTCTGCAACCCCGGCGAGCATTT
LC80            TCAACGCCACCATCCGCGACCGTTACTACGGCGCCGCCTCTGCAACCCCGGCGAGCATTT
LG117           TCAACGCCACCATCCGCGACCGTTACTACGGCGCCGCCTCTGCAACCCCGGCGAGCATTT
LH37-1          TCAACGCCACCATCCGCGACCGTTACTACGGCGCCGCCTCTGCAACCCCGGCGAGCATTT
LMG9322         TCAACGCCACCATCCGCGACCGTTACTACGGCGCCGCCTCTGCAACCCCGGCGAGCATTT
NCPPB3562       TCAACGCCACCATCCGCGACCGTTACTACGGCGCCGCCTCTGCAACCCCGGCGAGCATTT
CFBP2911        TCAACGCCACCATCCGCGACCGTTACTACGGCGCCGCCTCTGCAACCCCGGCGAGCATTT
JF90-2          TCAACGCCACCATCCGCGACCGTTACTACGGCGCCGCCTCTGCAACCCCGGCGAGCATTT
JK2-10          TCAACGCCACCATCCGCGACCGTTACTACGGCGCCGCCTCTGCAACCCCGGCGAGCATTT
JK48            TCAACGCCACCATCCGCGACCGTTACTACGGCGCCGCCTCTGCAACCCCGGCGAGCATTT
JM35            TCAACGCCACCATCCGCGACCGTTACTACGGCGCCGCCTCTGCAACCCCGGCGAGCATTT
JS581           TCAACGCCACCATCCGCGACCGTTACTACGGCGCCGCCTCTGCAACCCCGGCGAGCATTT
JS582           TCAACGCCACCATCCGCGACCGTTACTACGGCGCCGCCTCTGCAACCCCGGCGAGCATTT
JS584           TCAACGCCACCATCCGCGACCGTTACTACGGCGCCGCCTCTGCAACCCCGGCGAGCATTT
LB302           TCAACGCCACCATCCGCGACCGTTACTACGGCGCCGCCTCTGCAACCCCGGCGAGCATTT
LE20-1          TCAACGCCACCATCCGCGACCGTTACTACGGCGCCGCCTCTGCAACCCCGGCGAGCATTT
LG102           TCAACGCCACCATCCGCGACCGTTACTACGGCGCCGCCTCTGCAACCCCGGCGAGCATTT
LG115           TCAACGCCACCATCCGCGACCGTTACTACGGCGCCGCCTCTGCAACCCCGGCGAGCATTT
NCPPB3607       TCAACGCCACCATCCGCGACCGTTACTACGGCGCCGCCTCTGCAACCCCGGCGAGCATTT
NCPPB3608       TCAACGCCACCATCCGCGACCGTTACTACGGCGCCGCCTCTGCAACCCCGGCGAGCATTT
X2003-3218      TCAACGCCACCATCCGCGACCGTTACTACGGCGCCGCCTCTGCAACCCCGGCGAGCATTT
                ************************************************************

JJ238-24        TCCCGGTGCTGTTGCGCAATGCGCAGAATCACTTCGGCAAGCTGCGCAAAGACAAGGCGG
JK143-9         TCCCGGTGCTGTTGCGCAATGCGCAGAATCACTTCGGCAAGCTGCGCAAAGACAAGGCGG
JK143-11        TCCCGGTGCTGTTGCGCAATGCGCAGAATCACTTCGGCAAGCTGCGCAAAGACAAGGCGG
LD71a           TCCCGGTGCTGTTGCGCAATGCGCAGAATCACTTCGGCAAGCTGCGCAAAGACAAGGCGG
C40             TCCCGGTGCTGTTGCGCAATGCGCAGAATCACTTCGGCAAGCTGCGCAAAGACAAGGCGG
NCPPB3612       TCCCGGTGCTGTTGCGCAATGCGCAGAATCACTTCGGCAAGCTGCGCAAAGACAAGGCGG
CFBP2852        TCCCGGTGCTGTTGCGCAATGCGCAGAATCACTTCGGCAAGCTGCGCAAAGACAAGGCGG
FDC217          TCCCGGTGCTGTTGCGCAATGCGCAGAATCACTTCGGCAAGCTGCGCAAAGACAAGGCGG
FDC1083         TCCCGGTGCTGTTGCGCAATGCGCAGAATCACTTCGGCAAGCTGCGCAAAGACAAGGCGG
JJ10-1          TCCCGGTGCTGTTGCGCAATGCGCAGAATCACTTCGGCAAGCTGCGCAAAGACAAGGCGG
JJ238-10        TCCCGGTGCTGTTGCGCAATGCGCAGAATCACTTCGGCAAGCTGCGCAAAGACAAGGCGG
JK4-1           TCCCGGTGCTGTTGCGCAATGCGCAGAATCACTTCGGCAAGCTGCGCAAAGACAAGGCGG
JW160-1         TCCCGGTGCTGTTGCGCAATGCGCAGAATCACTTCGGCAAGCTGCGCAAAGACAAGGCGG
LB100-1         TCCCGGTGCTGTTGCGCAATGCGCAGAATCACTTCGGCAAGCTGCGCAAAGACAAGGCGG
LC80            TCCCGGTGCTGTTGCGCAATGCGCAGAATCACTTCGGCAAGCTGCGCAAAGACAAGGCGG
LG117           TCCCGGTGCTGTTGCGCAATGCGCAGAATCACTTCGGCAAGCTGCGCAAAGACAAGGCGG
LH37-1          TCCCGGTGCTGTTGCGCAATGCGCAGAATCACTTCGGCAAGCTGCGCAAAGACAAGGCGG
LMG9322         TCCCGGTGCTGTTGCGCAATGCGCAGAATCACTTCGGCAAGCTGCGCAAAGACAAGGCGG
NCPPB3562       TCCCGGTGCTGTTGCGCAATGCGCAGAATCACTTCGGCAAGCTGCGCAAAGACAAGGCGG
CFBP2911        TCCCGGTGCTGTTGCGCAATGCGCAGAATCACTTCGGCAAGCTGCGCAAAGACAAGGCGG
JF90-2          TCCCGGTGCTGTTGCGCAATGCGCAGAATCACTTCGGCAAGCTGCGCAAAGACAAGGCGG
JK2-10          TCCCGGTGCTGTTGCGCAATGCGCAGAATCACTTCGGCAAGCTGCGCAAAGACAAGGCGG
JK48            TCCCGGTGCTGTTGCGCAATGCGCAGAATCACTTCGGCAAGCTGCGCAAAGACAAGGCGG
JM35            TCCCGGTGCTGTTGCGCAATGCGCAGAATCACTTCGGCAAGCTGCGCAAAGACAAGGCGG
JS581           TCCCGGTGCTGTTGCGCAATGCGCAGAATCACTTCGGCAAGCTGCGCAAAGACAAGGCGG
JS582           TCCCGGTGCTGTTGCGCAATGCGCAGAATCACTTCGGCAAGCTGCGCAAAGACAAGGCGG
JS584           TCCCGGTGCTGTTGCGCAATGCGCAGAATCACTTCGGCAAGCTGCGCAAAGACAAGGCGG
LB302           TCCCGGTGCTGTTGCGCAATGCGCAGAATCACTTCGGCAAGCTGCGCAAAGACAAGGCGG
LE20-1          TCCCGGTGCTGTTGCGCAATGCGCAGAATCACTTCGGCAAGCTGCGCAAAGACAAGGCGG
LG102           TCCCGGTGCTGTTGCGCAATGCGCAGAATCACTTCGGCAAGCTGCGCAAAGACAAGGCGG
LG115           TCCCGGTGCTGTTGCGCAATGCGCAGAATCACTTCGGCAAGCTGCGCAAAGACAAGGCGG
NCPPB3607       TCCCGGTGCTGTTGCGCAATGCGCAGAATCACTTCGGCAAGCTGCGCAAAGACAAGGCGG
NCPPB3608       TCCCGGTGCTGTTGCGCAATGCGCAGAATCACTTCGGCAAGCTGCGCAAAGACAAGGCGG
X2003-3218      TCCCGGTGCTGTTGCGCAATGCGCAGAATCACTTCGGCAAGCTGCGCAAAGACAAGGCGG
                ************************************************************
```

```
JJ238-24        GCCTGGCGGTGAACCTGGAAAAAGAAGTCGGCCAGATCATCGACGCACTTCCCGCCAGCT
JK143-9         GCCTGGCGGTGAACCTGGAAAAAGAAGTCGGCCAGATCATCGACGCACTTCCCGCCAGCT
JK143-11        GCCTGGCGGTGAACCTGGAAAAAGAAGTCGGCCAGATCATCGACGCACTTCCCGCCAGCT
LD71a           GCCTGGCGGTGAACCTGGAAAAAGAAGTCGGCCAGATCATCGACGCACTTCCCGCCAGCT
C40             GCCTGGCGGTGAACCTGGAAAAAGAAGTCGGCCAGATCATCGACGCACTTCCCGCCAGCT
NCPPB3612       GCCTGGCGGTGAACCTGGAAAAAGAAGTCGGCCAGATCATCGACGCACTTCCCGCCAGCT
CFBP2852        GCCTGGCGGTGAACCTGGAAAAAGAAGTCGGCCAGATCATCGACGCACTTCCCGCCAGCT
FDC217          GCCTGGCGGTGAACCTGGAAAAAGAAGTCGGCCAGATCATCGACGCACTTCCCGCCAGCT
FDC1083         GCCTGGCGGTGAACCTGGAAAAAGAAGTCGGCCAGATCATCGACGCACTTCCCGCCAGCT
JJ10-1          GCCTGGCGGTGAACCTGGAAAAAGAAGTCGGCCAGATCATCGACGCACTTCCCGCCAGCT
JJ238-10        GCCTGGCGGTGAACCTGGAAAAAGAAGTCGGCCAGATCATCGACGCACTTCCCGCCAGCT
JK4-1           GCCTGGCGGTGAACCTGGAAAAAGAAGTCGGCCAGATCATCGACGCACTTCCCGCCAGCT
JW160-1         GCCTGGCGGTGAACCTGGAAAAAGAAGTCGGCCAGATCATCGACGCACTTCCCGCCAGCT
LB100-1         GCCTGGCGGTGAACCTGGAAAAAGAAGTCGGCCAGATCATCGACGCACTTCCCGCCAGCT
LC80            GCCTGGCGGTGAACCTGGAAAAAGAAGTCGGCCAGATCATCGACGCACTTCCCGCCAGCT
LG117           GCCTGGCGGTGAACCTGGAAAAAGAAGTCGGCCAGATCATCGACGCACTTCCCGCCAGCT
LH37-1          GCCTGGCGGTGAACCTGGAAAAAGAAGTCGGCCAGATCATCGACGCACTTCCCGCCAGCT
LMG9322         GCCTGGCGGTGAACCTGGAAAAAGAAGTCGGCCAGATCATCGACGCACTTCCCGCCAGCT
NCPPB3562       GCCTGGCGGTGAACCTGGAAAAAGAAGTCGGCCAGATCATCGACGCACTTCCCGCCAGCT
CFBP2911        GCCTGGCGGTGAACCTGGAAAAAGAAGTCGGCCAGATCATCGACGCACTTCCCGCCAGCT
JF90-2          GCCTGGCGGTGAACCTGGAAAAAGAAGTCGGCCAGATCATCGACGCACTTCCCGCCAGCT
JK2-10          GCCTGGCGGTGAACCTGGAAAAAGAAGTCGGCCAGATCATCGACGCACTTCCCGCCAGCT
JK48            GCCTGGCGGTGAACCTGGAAAAAGAAGTCGGCCAGATCATCGACGCACTTCCCGCCAGCT
JM35            GCCTGGCGGTGAACCTGGAAAAAGAAGTCGGCCAGATCATCGACGCACTTCCCGCCAGCT
JS581           GCCTGGCGGTGAACCTGGAAAAAGAAGTCGGCCAGATCATCGACGCACTTCCCGCCAGCT
JS582           GCCTGGCGGTGAACCTGGAAAAAGAAGTCGGCCAGATCATCGACGCACTTCCCGCCAGCT
JS584           GCCTGGCGGTGAACCTGGAAAAAGAAGTCGGCCAGATCATCGACGCACTTCCCGCCAGCT
LB302           GCCTGGCGGTGAACCTGGAAAAAGAAGTCGGCCAGATCATCGACGCACTTCCCGCCAGCT
LE20-1          GCCTGGCGGTGAACCTGGAAAAAGAAGTCGGCCAGATCATCGACGCACTTCCCGCCAGCT
LG102           GCCTGGCGGTGAACCTGGAAAAAGAAGTCGGCCAGATCATCGACGCACTTCCCGCCAGCT
LG115           GCCTGGCGGTGAACCTGGAAAAAGAAGTCGGCCAGATCATCGACGCACTTCCCGCCAGCT
NCPPB3607       GCCTGGCGGTGAACCTGGAAAAAGAAGTCGGCCAGATCATCGACGCACTTCCCGCCAGCT
NCPPB3608       GCCTGGCGGTGAACCTGGAAAAAGAAGTCGGCCAGATCATCGACGCACTTCCCGCCAGCT
X2003-3218      GCCTGGCGGTGAACCTGGAAAAAGAAGTCGGCCAGATCATCGACGCACTTCCCGCCAGCT
                ************************************************************

JJ238-24        TCCCGCGCAGCCTGCCCATCCACGAGCAAGGCCGTTTCGCCATCGGTTACTACCACCAGA
JK143-9         TCCCGCGCAGCCTGCCCATCCACGAGCAAGGCCGTTTCGCCATCGGTTACTACCACCAGA
JK143-11        TCCCGCGCAGCCTGCCCATCCACGAGCAAGGCCGTTTCGCCATCGGTTACTACCACCAGA
LD71a           TCCCGCGCAGCCTGCCCATCCACGAGCAAGGCCGTTTCGCCATCGGTTACTACCACCAGA
C40             TCCCGCGCAGCCTGCCCATCCACGAGCAAGGCCGTTTCGCCATCGGTTACTACCACCAGA
NCPPB3612       TCCCGCGCAGCCTGCCCATCCACGAGCAAGGCCGTTTCGCCATCGGTTACTACCACCAGA
CFBP2852        TCCCGCGCAGCCTGCCCATCCACGAGCAAGGCCGTTTCGCCATCGGTTACTACCACCAGA
FDC217          TCCCGCGCAGCCTGCCCATCCACGAGCAAGGCCGTTTCGCCATCGGTTACTACCACCAGA
FDC1083         TCCCGCGCAGCCTGCCCATCCACGAGCAAGGCCGTTTCGCCATCGGTTACTACCACCAGA
JJ10-1          TCCCGCGCAGCCTGCCCATCCACGAGCAAGGCCGTTTCGCCATCGGTTACTACCACCAGA
JJ238-10        TCCCGCGCAGCCTGCCCATCCACGAGCAAGGCCGTTTCGCCATCGGTTACTACCACCAGA
JK4-1           TCCCGCGCAGCCTGCCCATCCACGAGCAAGGCCGTTTCGCCATCGGTTACTACCACCAGA
JW160-1         TCCCGCGCAGCCTGCCCATCCACGAGCAAGGCCGTTTCGCCATCGGTTACTACCACCAGA
LB100-1         TCCCGCGCAGCCTGCCCATCCACGAGCAAGGCCGTTTCGCCATCGGTTACTACCACCAGA
LC80            TCCCGCGCAGCCTGCCCATCCACGAGCAAGGCCGTTTCGCCATCGGTTACTACCACCAGA
LG117           TCCCGCGCAGCCTGCCCATCCACGAGCAAGGCCGTTTCGCCATCGGTTACTACCACCAGA
LH37-1          TCCCGCGCAGCCTGCCCATCCACGAGCAAGGCCGTTTCGCCATCGGTTACTACCACCAGA
LMG9322         TCCCGCGCAGCCTGCCCATCCACGAGCAAGGCCGTTTCGCCATCGGTTACTACCACCAGA
NCPPB3562       TCCCGCGCAGCCTGCCCATCCACGAGCAAGGCCGTTTCGCCATCGGTTACTACCACCAGA
CFBP2911        TCCCGCGCAGCCTGCCCATCCACGAGCAAGGCCGTTTCGCCATCGGTTACTACCACCAGA
JF90-2          TCCCGCGCAGCCTGCCCATCCACGAGCAAGGCCGTTTCGCCATCGGTTACTACCACCAGA
JK2-10          TCCCGCGCAGCCTGCCCATCCACGAGCAAGGCCGTTTCGCCATCGGTTACTACCACCAGA
JK48            TCCCGCGCAGCCTGCCCATCCACGAGCAAGGCCGTTTCGCCATCGGTTACTACCACCAGA
JM35            TCCCGCGCAGCCTGCCCATCCACGAGCAAGGCCGTTTCGCCATCGGTTACTACCACCAGA
JS581           TCCCGCGCAGCCTGCCCATCCACGAGCAAGGCCGTTTCGCCATCGGTTACTACCACCAGA
JS582           TCCCGCGCAGCCTGCCCATCCACGAGCAAGGCCGTTTCGCCATCGGTTACTACCACCAGA
JS584           TCCCGCGCAGCCTGCCCATCCACGAGCAAGGCCGTTTCGCCATCGGTTACTACCACCAGA
LB302           TCCCGCGCAGCCTGCCCATCCACGAGCAAGGCCGTTTCGCCATCGGTTACTACCACCAGA
LE20-1          TCCCGCGCAGCCTGCCCATCCACGAGCAAGGCCGTTTCGCCATCGGTTACTACCACCAGA
LG102           TCCCGCGCAGCCTGCCCATCCACGAGCAAGGCCGTTTCGCCATCGGTTACTACCACCAGA
LG115           TCCCGCGCAGCCTGCCCATCCACGAGCAAGGCCGTTTCGCCATCGGTTACTACCACCAGA
NCPPB3607       TCCCGCGCAGCCTGCCCATCCACGAGCAAGGCCGTTTCGCCATCGGTTACTACCACCAGA
NCPPB3608       TCCCGCGCAGCCTGCCCATCCACGAGCAAGGCCGTTTCGCCATCGGTTACTACCACCAGA
X2003-3218      TCCCGCGCAGCCTGCCCATCCACGAGCAAGGCCGTTTCGCCATCGGTTACTACCACCAGA
                ************************************************************
```

```
JJ238-24        CCCAGGCGCGCTTCGCCCGCAACAACGGCCAGGACGCCCCCGACACCGCTTCCGAAGGAG
JK143-9         CCCAGGCGCGCTTCGCCCGCAACAACGGCCAGGACGCCCCCGACACCGCTTCCGAAGGAG
JK143-11        CCCAGGCGCGCTTCGCCCGCAACAACGGCCAGGACGCCCCCGACACCGCTTCCGAAGGAG
LD71a           CCCAGGCGCGCTTCGCCCGCAACAACGGCCAGGACGCCCCCGACACCGCTTCCGAAGGAG
C40             CCCAGGCGCGCTTCGCCCGCAACAACGGCCAGGACGCCCCCGACACCGCTTCCGAAGGAG
NCPPB3612       CCCAGGCGCGCTTCGCCCGCAACAACGGCCAGGACGCCCCCGACACCGCTTCCGAAGGAG
CFBP2852        CCCAGGCGCGCTTCGCCCGCAACAACGGCCAGGACGCCCCCGACACCGCTTCCGAAGGAG
FDC217          CCCAGGCGCGCTTCGCCCGCAACAACGGCCAGGACGCCCCCGACACCGCTTCCGAAGGAG
FDC1083         CCCAGGCGCGCTTCGCCCGCAACAACGGCCAGGACGCCCCCGACACCGCTTCCGAAGGAG
JJ10-1          CCCAGGCGCGCTTCGCCCGCAACAACGGCCAGGACGCCCCCGACACCGCTTCCGAAGGAG
JJ238-10        CCCAGGCGCGCTTCGCCCGCAACAACGGCCAGGACGCCCCCGACACCGCTTCCGAAGGAG
JK4-1           CCCAGGCGCGCTTCGCCCGCAACAACGGCCAGGACGCCCCCGACACCGCTTCCGAAGGAG
JW160-1         CCCAGGCGCGCTTCGCCCGCAACAACGGCCAGGACGCCCCCGACACCGCTTCCGAAGGAG
LB100-1         CCCAGGCGCGCTTCGCCCGCAACAACGGCCAGGACGCCCCCGACACCGCTTCCGAAGGAG
LC80            CCCAGGCGCGCTTCGCCCGCAACAACGGCCAGGACGCCCCCGACACCGCTTCCGAAGGAG
LG117           CCCAGGCGCGCTTCGCCCGCAACAACGGCCAGGACGCCCCCGACACCGCTTCCGAAGGAG
LH37-1          CCCAGGCGCGCTTCGCCCGCAACAACGGCCAGGACGCCCCCGACACCGCTTCCGAAGGAG
LMG9322         CCCAGGCGCGCTTCGCCCGCAACAACGGCCAGGACGCCCCCGACACCGCTTCCGAAGGAG
NCPPB3562       CCCAGGCGCGCTTCGCCCGCAACAACGGCCAGGACGCCCCCGACACCGCTTCCGAAGGAG
CFBP2911        CCCAGGCGCGCTTCGCCCGCAACAACGGCCAGGACGCCCCCGACACCGCTTCCGAAGGAG
JF90-2          CCCAGGCGCGCTTCGCCCGCAACAACGGCCAGGACGCCCCCGACACCGCTTCCGAAGGAG
JK2-10          CCCAGGCGCGCTTCGCCCGCAACAACGGCCAGGACGCCCCCGACACCGCTTCCGAAGGAG
JK48            CCCAGGCGCGCTTCGCCCGCAACAACGGCCAGGACGCCCCCGACACCGCTTCCGAAGGAG
JM35            CCCAGGCGCGCTTCGCCCGCAACAACGGCCAGGACGCCCCCGACACCGCTTCCGAAGGAG
JS581           CCCAGGCGCGCTTCGCCCGCAACAACGGCCAGGACGCCCCCGACACCGCTTCCGAAGGAG
JS582           CCCAGGCGCGCTTCGCCCGCAACAACGGCCAGGACGCCCCCGACACCGCTTCCGAAGGAG
JS584           CCCAGGCGCGCTTCGCCCGCAACAACGGCCAGGACGCCCCCGACACCGCTTCCGAAGGAG
LB302           CCCAGGCGCGCTTCGCCCGCAACAACGGCCAGGACGCCCCCGACACCGCTTCCGAAGGAG
LE20-1          CCCAGGCGCGCTTCGCCCGCAACAACGGCCAGGACGCCCCCGACACCGCTTCCGAAGGAG
LG102           CCCAGGCGCGCTTCGCCCGCAACAACGGCCAGGACGCCCCCGACACCGCTTCCGAAGGAG
LG115           CCCAGGCGCGCTTCGCCCGCAACAACGGCCAGGACGCCCCCGACACCGCTTCCGAAGGAG
NCPPB3607       CCCAGGCGCGCTTCGCCCGCAACAACGGCCAGGACGCCCCCGACACCGCTTCCGAAGGAG
NCPPB3608       CCCAGGCGCGCTTCGCCCGCAACAACGGCCAGGACGCCCCCGACACCGCTTCCGAAGGAG
X2003-3218      CCCAGGCGCGCTTCGCCCGCAACAACGGCCAGGACGCCCCCGACACCGCTTCCGAAGGAG
                ************************************************************

JJ238-24        AACCCACATGA
JK143-9         AACCCACATGA
JK143-11        AACCCACATGA
LD71a           AACCCACATGA
C40             AACCCACATGA
NCPPB3612       AACCCACATGA
CFBP2852        AACCCACATGA
FDC217          AACCCACATGA
FDC1083         AACCCACATGA
JJ10-1          AACCCACATGA
JJ238-10        AACCCACATGA
JK4-1           AACCCACATGA
JW160-1         AACCCACATGA
LB100-1         AACCCACATGA
LC80            AACCCACATGA
LG117           AACCCACATGA
LH37-1          AACCCACATGA
LMG9322         AACCCACATGA
NCPPB3562       AACCCACATGA
CFBP2911        AACCCACATGA
JF90-2          AACCCACATGA
JK2-10          AACCCACATGA
JK48            AACCCACATGA
JM35            AACCCACATGA
JS581           AACCCACATGA
JS582           AACCCACATGA
JS584           AACCCACATGA
LB302           AACCCACATGA
LE20-1          AACCCACATGA
LG102           AACCCACATGA
LG115           AACCCACATGA
NCPPB3607       AACCCACATGA
NCPPB3608       AACCCACATGA
X2003-3218      AACCCACATGA
                ***********
```

Cas7-Csd2 (Cascade)

```
C40             ATGAGCGCCATCGCCCACCGCTATGAATTCGTCTATCTATTCGACGTGGCCAACGGTAAC
CFBP2852        ATGAGCGCCATCGCCCACCGCTATGAATTCGTCTATCTATTCGACGTGGCCAACGGTAAC
CFBP2911        ATGAGCGCCATCGCCCACCGCTATGAATTCGTCTATCTATTCGACGTGGCCAACGGTAAC
FDC217          ATGAGCGCCATCGCCCACCGCTATGAATTCGTCTATCTATTCGACGTGGCCAACGGTAAC
FDC1083         ATGAGCGCCATCGCCCACCGCTATGAATTCGTCTATCTATTCGACGTGGCCAACGGTAAC
JF90-2          ATGAGCGCCATCGCCCACCGCTATGAATTCGTCTATCTATTCGACGTGGCCAACGGTAAC
JF90-8          ATGAGCGCCATCGCCCACCGCTATGAATTCGTCTATCTATTCGACGTGGCCAACGGTAAC
JJ10-1          ATGAGCGCCATCGCCCACCGCTATGAATTCGTCTATCTATTCGACGTGGCCAACGGTAAC
JJ238-10        ATGAGCGCCATCGCCCACCGCTATGAATTCGTCTATCTATTCGACGTGGCCAACGGTAAC
JJ238-24        ATGAGCGCCATCGCCCACCGCTATGAATTCGTCTATCTATTCGACGTGGCCAACGGTAAC
JK2-10          ATGAGCGCCATCGCCCACCGCTATGAATTCGTCTATCTATTCGACGTGGCCAACGGTAAC
JK4-1           ATGAGCGCCATCGCCCACCGCTATGAATTCGTCTATCTATTCGACGTGGCCAACGGTAAC
JK48            ATGAGCGCCATCGCCCACCGCTATGAATTCGTCTATCTATTCGACGTGGCCAACGGTAAC
JK143-9         ATGAGCGCCATCGCCCACCGCTATGAATTCGTCTATCTATTCGACGTGGCCAACGGTAAC
JK143-11        ATGAGCGCCATCGCCCACCGCTATGAATTCGTCTATCTATTCGACGTGGCCAACGGTAAC
JM35            ATGAGCGCCATCGCCCACCGCTATGAATTCGTCTATCTATTCGACGTGGCCAACGGTAAC
JS581           ATGAGCGCCATCGCCCACCGCTATGAATTCGTCTATCTATTCGACGTGGCCAACGGTAAC
JS582           ATGAGCGCCATCGCCCACCGCTATGAATTCGTCTATCTATTCGACGTGGCCAACGGTAAC
JS584           ATGAGCGCCATCGCCCACCGCTATGAATTCGTCTATCTATTCGACGTGGCCAACGGTAAC
JW160-1         ATGAGCGCCATCGCCCACCGCTATGAATTCGTCTATCTATTCGACGTGGCCAACGGTAAC
LB100-1         ATGAGCGCCATCGCCCACCGCTATGAATTCGTCTATCTATTCGACGTGGCCAACGGTAAC
LB302           ATGAGCGCCATCGCCCACCGCTATGAATTCGTCTATCTATTCGACGTGGCCAACGGTAAC
LC80            ATGAGCGCCATCGCCCACCGCTATGAATTCGTCTATCTATTCGACGTGGCCAACGGTAAC
LD7-1           ATGAGCGCCATCGCCCACCGCTATGAATTCGTCTATCTATTCGACGTGGCCAACGGTAAC
LD71a           ATGAGCGCCATCGCCCACCGCTATGAATTCGTCTATCTATTCGACGTGGCCAACGGTAAC
LE3-1           ATGAGCGCCATCGCCCACCGCTATGAATTCGTCTATCTATTCGACGTGGCCAACGGTAAC
LE20-1          ATGAGCGCCATCGCCCACCGCTATGAATTCGTCTATCTATTCGACGTGGCCAACGGTAAC
LE116-1         ATGAGCGCCATCGCCCACCGCTATGAATTCGTCTATCTATTCGACGTGGCCAACGGTAAC
LG97            ATGAGCGCCATCGCCCACCGCTATGAATTCGTCTATCTATTCGACGTGGCCAACGGTAAC
LG102           ATGAGCGCCATCGCCCACCGCTATGAATTCGTCTATCTATTCGACGTGGCCAACGGTAAC
LG115           ATGAGCGCCATCGCCCACCGCTATGAATTCGTCTATCTATTCGACGTGGCCAACGGTAAC
LG117           ATGAGCGCCATCGCCCACCGCTATGAATTCGTCTATCTATTCGACGTGGCCAACGGTAAC
LH37-1          ATGAGCGCCATCGCCCACCGCTATGAATTCGTCTATCTATTCGACGTGGCCAACGGTAAC
LMG9322         ATGAGCGCCATCGCCCACCGCTATGAATTCGTCTATCTATTCGACGTGGCCAACGGTAAC
NCPPB3562       ATGAGCGCCATCGCCCACCGCTATGAATTCGTCTATCTATTCGACGTGGCCAACGGTAAC
NCPPB3607       ATGAGCGCCATCGCCCACCGCTATGAATTCGTCTATCTATTCGACGTGGCCAACGGTAAC
NCPPB3608       ATGAGCGCCATCGCCCACCGCTATGAATTCGTCTATCTATTCGACGTGGCCAACGGTAAC
NCPPB3610       ATGAGCGCCATCGCCCACCGCTATGAATTCGTCTATCTATTCGACGTGGCCAACGGTAAC
NCPPB3612       ATGAGCGCCATCGCCCACCGCTATGAATTCGTCTATCTATTCGACGTGGCCAACGGTAAC
NCPPB3615       ATGAGCGCCATCGCCCACCGCTATGAATTCGTCTATCTATTCGACGTGGCCAACGGTAAC
X2003-3218      ATGAGCGCCATCGCCCACCGCTATGAATTCGTCTATCTATTCGACGTGGCCAACGGTAAC
                ************************************************************

C40             CCTAACGGTGACCCCGACGCCGGCAACCTGCCGCGCCTGGACCCGGAGACCAATCGCGGC
CFBP2852        CCTAACGGTGACCCCGACGCCGGCAACCTGCCGCGCCTGGACCCGGAGACCAATCGCGGC
CFBP2911        CCTAACGGTGACCCCGACGCCGGCAACCTGCCGCGCCTGGACCCGGAGACCAATCGCGGC
FDC217          CCTAACGGTGACCCCGACGCCGGCAACCTGCCGCGCCTGGACCCGGAGACCAATCGCGGC
FDC1083         CCTAACGGTGACCCCGACGCCGGCAACCTGCCGCGCCTGGACCCGGAGACCAATCGCGGC
JF90-2          CCTAACGGTGACCCCGACGCCGGCAACCTGCCGCGCCTGGACCCGGAGACCAATCGCGGC
JF90-8          CCTAACGGTGACCCCGACGCCGGCAACCTGCCGCGCCTGGACCCGGAGACCAATCGCGGC
JJ10-1          CCTAACGGTGACCCCGACGCCGGCAACCTGCCGCGCCTGGACCCGGAGACCAATCGCGGC
JJ238-10        CCTAACGGTGACCCCGACGCCGGCAACCTGCCGCGCCTGGACCCGGAGACCAATCGCGGC
JJ238-24        CCTAACGGTGACCCCGACGCCGGCAACCTGCCGCGCCTGGACCCGGAGACCAATCGCGGC
JK2-10          CCTAACGGTGACCCCGACGCCGGCAACCTGCCGCGCCTGGACCCGGAGACCAATCGCGGC
JK4-1           CCTAACGGTGACCCCGACGCCGGCAACCTGCCGCGCCTGGACCCGGAGACCAATCGCGGC
JK48            CCTAACGGTGACCCCGACGCCGGCAACCTGCCGCGCCTGGACCCGGAGACCAATCGCGGC
JK143-9         CCTAACGGTGACCCCGACGCCGGCAACCTGCCGCGCCTGGACCCGGAGACCAATCGCGGC
JK143-11        CCTAACGGTGACCCCGACGCCGGCAACCTGCCGCGCCTGGACCCGGAGACCAATCGCGGC
JM35            CCTAACGGTGACCCCGACGCCGGCAACCTGCCGCGCCTGGACCCGGAGACCAATCGCGGC
JS581           CCTAACGGTGACCCCGACGCCGGCAACCTGCCGCGCCTGGACCCGGAGACCAATCGCGGC
JS582           CCTAACGGTGACCCCGACGCCGGCAACCTGCCGCGCCTGGACCCGGAGACCAATCGCGGC
JS584           CCTAACGGTGACCCCGACGCCGGCAACCTGCCGCGCCTGGACCCGGAGACCAATCGCGGC
JW160-1         CCTAACGGTGACCCCGACGCCGGCAACCTGCCGCGCCTGGACCCGGAGACCAATCGCGGC
LB100-1         CCTAACGGTGACCCCGACGCCGGCAACCTGCCGCGCCTGGACCCGGAGACCAATCGCGGC
LB302           CCTAACGGTGACCCCGACGCCGGCAACCTGCCGCGCCTGGACCCGGAGACCAATCGCGGC
LC80            CCTAACGGTGACCCCGACGCCGGCAACCTGCCGCGCCTGGACCCGGAGACCAATCGCGGC
LD7-1           CCTAACGGTGACCCCGACGCCGGCAACCTGCCGCGCCTGGACCCGGAGACCAATCGCGGC
LD71a           CCTAACGGTGACCCCGACGCCGGCAACCTGCCGCGCCTGGACCCGGAGACCAATCGCGGC
LE3-1           CCTAACGGTGACCCCGACGCCGGCAACCTGCCGCGCCTGGACCCGGAGACCAATCGCGGC
LE20-1          CCTAACGGTGACCCCGACGCCGGCAACCTGCCGCGCCTGGACCCGGAGACCAATCGCGGC
LE116-1         CCTAACGGTGACCCCGACGCCGGCAACCTGCCGCGCCTGGACCCGGAGACCAATCGCGGC
LG97            CCTAACGGTGACCCCGACGCCGGCAACCTGCCGCGCCTGGACCCGGAGACCAATCGCGGC
LG102           CCTAACGGTGACCCCGACGCCGGCAACCTGCCGCGCCTGGACCCGGAGACCAATCGCGGC
LG115           CCTAACGGTGACCCCGACGCCGGCAACCTGCCGCGCCTGGACCCGGAGACCAATCGCGGC
LG117           CCTAACGGTGACCCCGACGCCGGCAACCTGCCGCGCCTGGACCCGGAGACCAATCGCGGC
LH37-1          CCTAACGGTGACCCCGACGCCGGCAACCTGCCGCGCCTGGACCCGGAGACCAATCGCGGC
LMG9322         CCTAACGGTGACCCCGACGCCGGCAACCTGCCGCGCCTGGACCCGGAGACCAATCGCGGC
NCPPB3562       CCTAACGGTGACCCCGACGCCGGCAACCTGCCGCGCCTGGACCCGGAGACCAATCGCGGC
NCPPB3607       CCTAACGGTGACCCCGACGCCGGCAACCTGCCGCGCCTGGACCCGGAGACCAATCGCGGC
NCPPB3608       CCTAACGGTGACCCCGACGCCGGCAACCTGCCGCGCCTGGACCCGGAGACCAATCGCGGC
NCPPB3610       CCTAACGGTGACCCCGACGCCGGCAACCTGCCGCGCCTGGACCCGGAGACCAATCGCGGC
NCPPB3612       CCTAACGGTGACCCCGACGCCGGCAACCTGCCGCGCCTGGACCCGGAGACCAATCGCGGC
NCPPB3615       CCTAACGGTGACCCCGACGCCGGCAACCTGCCGCGCCTGGACCCGGAGACCAATCGCGGC
X2003-3218      CCTAACGGTGACCCCGACGCCGGCAACCTGCCGCGCCTGGACCCGGAGACCAATCGCGGC
                ************************************************************
```

```
C40             CTGGTGACCGACGTGGCGCTCAAGCGCAAGATCCGCAACTACGTGGCGCTGGAGAAAGAC
CFBP2852        CTGGTGACCGACGTGGCGCTCAAGCGCAAGATCCGCAACTACGTGGCGCTGGAGAAAGAC
CFBP2911        CTGGTGACCGACGTGGCGCTCAAGCGCAAGATCCGCAACTACGTGGCGCTGGAGAAAGAC
FDC217          CTGGTGACCGACGTGGCGCTCAAGCGCAAGATCCGCAACTACGTGGCGCTGGAGAAAGAC
FDC1083         CTGGTGACCGACGTGGCGCTCAAGCGCAAGATCCGCAACTACGTGGCGCTGGAGAAAGAC
JF90-2          CTGGTGACCGACGTGGCGCTCAAGCGCAAGATCCGCAACTACGTGGCGCTGGAGAAAGAC
JF90-8          CTGGTGACCGACGTGGCGCTCAAGCGCAAGATCCGCAACTACGTGGCGCTGGAGAAAGAC
JJ10-1          CTGGTGACCGACGTGGCGCTCAAGCGCAAGATCCGCAACTACGTGGCGCTGGAGAAAGAC
JJ238-10        CTGGTGACCGACGTGGCGCTCAAGCGCAAGATCCGCAACTACGTGGCGCTGGAGAAAGAC
JJ238-24        CTGGTGACCGACGTGGCGCTCAAGCGCAAGATCCGCAACTACGTGGCGCTGGAGAAAGAC
JK2-10          CTGGTGACCGACGTGGCGCTCAAGCGCAAGATCCGCAACTACGTGGCGCTGGAGAAAGAC
JK4-1           CTGGTGACCGACGTGGCGCTCAAGCGCAAGATCCGCAACTACGTGGCGCTGGAGAAAGAC
JK48            CTGGTGACCGACGTGGCGCTCAAGCGCAAGATCCGCAACTACGTGGCGCTGGAGAAAGAC
JK143-9         CTGGTGACCGACGTGGCGCTCAAGCGCAAGATCCGCAACTACGTGGCGCTGGAGAAAGAC
JK143-11        CTGGTGACCGACGTGGCGCTCAAGCGCAAGATCCGCAACTACGTGGCGCTGGAGAAAGAC
JM35            CTGGTGACCGACGTGGCGCTCAAGCGCAAGATCCGCAACTACGTGGCGCTGGAGAAAGAC
JS581           CTGGTGACCGACGTGGCGCTCAAGCGCAAGATCCGCAACTACGTGGCGCTGGAGAAAGAC
JS582           CTGGTGACCGACGTGGCGCTCAAGCGCAAGATCCGCAACTACGTGGCGCTGGAGAAAGAC
JS584           CTGGTGACCGACGTGGCGCTCAAGCGCAAGATCCGCAACTACGTGGCGCTGGAGAAAGAC
JW160-1         CTGGTGACCGACGTGGCGCTCAAGCGCAAGATCCGCAACTACGTGGCGCTGGAGAAAGAC
LB100-1         CTGGTGACCGACGTGGCGCTCAAGCGCAAGATCCGCAACTACGTGGCGCTGGAGAAAGAC
LB302           CTGGTGACCGACGTGGCGCTCAAGCGCAAGATCCGCAACTACGTGGCGCTGGAGAAAGAC
LC80            CTGGTGACCGACGTGGCGCTCAAGCGCAAGATCCGCAACTACGTGGCGCTGGAGAAAGAC
LD7-1           CTGGTGACCGACGTGGCGCTCAAGCGCAAGATCCGCAACTACGTGGCGCTGGAGAAAGAC
LD71a           CTGGTGACCGACGTGGCGCTCAAGCGCAAGATCCGCAACTACGTGGCGCTGGAGAAAGAC
LE3-1           CTGGTGACCGACGTGGCGCTCAAGCGCAAGATCCGCAACTACGTGGCGCTGGAGAAAGAC
LE20-1          CTGGTGACCGACGTGGCGCTCAAGCGCAAGATCCGCAACTACGTGGCGCTGGAGAAAGAC
LE116-1         CTGGTGACCGACGTGGCGCTCAAGCGCAAGATCCGCAACTACGTGGCGCTGGAGAAAGAC
LG97            CTGGTGACCGACGTGGCGCTCAAGCGCAAGATCCGCAACTACGTGGCGCTGGAGAAAGAC
LG102           CTGGTGACCGACGTGGCGCTCAAGCGCAAGATCCGCAACTACGTGGCGCTGGAGAAAGAC
LG115           CTGGTGACCGACGTGGCGCTCAAGCGCAAGATCCGCAACTACGTGGCGCTGGAGAAAGAC
LG117           CTGGTGACCGACGTGGCGCTCAAGCGCAAGATCCGCAACTACGTGGCGCTGGAGAAAGAC
LH37-1          CTGGTGACCGACGTGGCGCTCAAGCGCAAGATCCGCAACTACGTGGCGCTGGAGAAAGAC
LMG9322         CTGGTGACCGACGTGGCGCTCAAGCGCAAGATCCGCAACTACGTGGCGCTGGAGAAAGAC
NCPPB3562       CTGGTGACCGACGTGGCGCTCAAGCGCAAGATCCGCAACTACGTGGCGCTGGAGAAAGAC
NCPPB3607       CTGGTGACCGACGTGGCGCTCAAGCGCAAGATCCGCAACTACGTGGCGCTGGAGAAAGAC
NCPPB3608       CTGGTGACCGACGTGGCGCTCAAGCGCAAGATCCGCAACTACGTGGCGCTGGAGAAAGAC
NCPPB3610       CTGGTGACCGACGTGGCGCTCAAGCGCAAGATCCGCAACTACGTGGCGCTGGAGAAAGAC
NCPPB3612       CTGGTGACCGACGTGGCGCTCAAGCGCAAGATCCGCAACTACGTGGCGCTGGAGAAAGAC
NCPPB3615       CTGGTGACCGACGTGGCGCTCAAGCGCAAGATCCGCAACTACGTGGCGCTGGAGAAAGAC
X2003-3218      CTGGTGACCGACGTGGCGCTCAAGCGCAAGATCCGCAACTACGTGGCGCTGGAGAAAGAC
                ************************************************************

C40             AACGCGCCGGGTTACACGATCTACATGCAGGAGAAATCGGTACTGAACAACCAGCACAAG
CFBP2852        AACGCGCCGGGTTACACGATCTACATGCAGGAGAAATCGGTACTGAACAACCAGCACAAG
CFBP2911        AACGCGCCGGGTTACACGATCTACATGCAGGAGAAATCGGTACTGAACAACCAGCACAAG
FDC217          AACGCGCCGGGTTACACGATCTACATGCAGGAGAAATCGGTACTGAACAACCAGCACAAG
FDC1083         AACGCGCCGGGTTACACGATCTACATGCAGGAGAAATCGGTACTGAACAACCAGCACAAG
JF90-2          AACGCGCCGGGTTACACGATCTACATGCAGGAGAAATCGGTACTGAACAACCAGCACAAG
JF90-8          AACGCGCCGGGTTACACGATCTACATGCAGGAGAAATCGGTACTGAACAACCAGCACAAG
JJ10-1          AACGCGCCGGGTTACACGATCTACATGCAGGAGAAATCGGTACTGAACAACCAGCACAAG
JJ238-10        AACGCGCCGGGTTACACGATCTACATGCAGGAGAAATCGGTACTGAACAACCAGCACAAG
JJ238-24        AACGCGCCGGGTTACACGATCTACATGCAGGAGAAATCGGTACTGAACAACCAGCACAAG
JK2-10          AACGCGCCGGGTTACACGATCTACATGCAGGAGAAATCGGTACTGAACAACCAGCACAAG
JK4-1           AACGCGCCGGGTTACACGATCTACATGCAGGAGAAATCGGTACTGAACAACCAGCACAAG
JK48            AACGCGCCGGGTTACACGATCTACATGCAGGAGAAATCGGTACTGAACAACCAGCACAAG
JK143-9         AACGCGCCGGGTTACACGATCTACATGCAGGAGAAATCGGTACTGAACAACCAGCACAAG
JK143-11        AACGCGCCGGGTTACACGATCTACATGCAGGAGAAATCGGTACTGAACAACCAGCACAAG
JM35            AACGCGCCGGGTTACACGATCTACATGCAGGAGAAATCGGTACTGAACAACCAGCACAAG
JS581           AACGCGCCGGGTTACACGATCTACATGCAGGAGAAATCGGTACTGAACAACCAGCACAAG
JS582           AACGCGCCGGGTTACACGATCTACATGCAGGAGAAATCGGTACTGAACAACCAGCACAAG
JS584           AACGCGCCGGGTTACACGATCTACATGCAGGAGAAATCGGTACTGAACAACCAGCACAAG
JW160-1         AACGCGCCGGGTTACACGATCTACATGCAGGAGAAATCGGTACTGAACAACCAGCACAAG
LB100-1         AACGCGCCGGGTTACACGATCTACATGCAGGAGAAATCGGTACTGAACAACCAGCACAAG
LB302           AACGCGCCGGGTTACACGATCTACATGCAGGAGAAATCGGTACTGAACAACCAGCACAAG
LC80            AACGCGCCGGGTTACACGATCTACATGCAGGAGAAATCGGTACTGAACAACCAGCACAAG
LD7-1           AACGCGCCGGGTTACACGATCTACATGCAGGAGAAATCGGTACTGAACAACCAGCACAAG
LD71a           AACGCGCCGGGTTACACGATCTACATGCAGGAGAAATCGGTACTGAACAACCAGCACAAG
LE3-1           AACGCGCCGGGTTACACGATCTACATGCAGGAGAAATCGGTACTGAACAACCAGCACAAG
LE20-1          AACGCGCCGGGTTACACGATCTACATGCAGGAGAAATCGGTACTGAACAACCAGCACAAG
LE116-1         AACGCGCCGGGTTACACGATCTACATGCAGGAGAAATCGGTACTGAACAACCAGCACAAG
LG97            AACGCGCCGGGTTACACGATCTACATGCAGGAGAAATCGGTACTGAACAACCAGCACAAG
LG102           AACGCGCCGGGTTACACGATCTACATGCAGGAGAAATCGGTACTGAACAACCAGCACAAG
LG115           AACGCGCCGGGTTACACGATCTACATGCAGGAGAAATCGGTACTGAACAACCAGCACAAG
LG117           AACGCGCCGGGTTACACGATCTACATGCAGGAGAAATCGGTACTGAACAACCAGCACAAG
LH37-1          AACGCGCCGGGTTACACGATCTACATGCAGGAGAAATCGGTACTGAACAACCAGCACAAG
LMG9322         AACGCGCCGGGTTACACGATCTACATGCAGGAGAAATCGGTACTGAACAACCAGCACAAG
NCPPB3562       AACGCGCCGGGTTACACGATCTACATGCAGGAGAAATCGGTACTGAACAACCAGCACAAG
NCPPB3607       AACGCGCCGGGTTACACGATCTACATGCAGGAGAAATCGGTACTGAACAACCAGCACAAG
NCPPB3608       AACGCGCCGGGTTACACGATCTACATGCAGGAGAAATCGGTACTGAACAACCAGCACAAG
NCPPB3610       AACGCGCCGGGTTACACGATCTACATGCAGGAGAAATCGGTACTGAACAACCAGCACAAG
NCPPB3612       AACGCGCCGGGTTACACGATCTACATGCAGGAGAAATCGGTACTGAACAACCAGCACAAG
NCPPB3615       AACGCGCCGGGTTACACGATCTACATGCAGGAGAAATCGGTACTGAACAACCAGCACAAG
X2003-3218      AACGCGCCGGGTTACACGATCTACATGCAGGAGAAATCGGTACTGAACAACCAGCACAAG
                ************************************************************
```

```
C40               CAGGCCTACACCGCACTTGGCATCGAGCATGAAGCCAAGAAACTGCCCAAGGAGGGCGAC
CFBP2852          CAGGCCTACACCGCACTTGGCATCGAGCATGAAGCCAAGAAACTGCCCAAGGAGGGCGAC
CFBP2911          CAGGCCTACACCGCACTTGGCATCGAGCATGAAGCCAAGAAACTGCCCAAGGAGGGCGAC
FDC217            CAGGCCTACACCGCACTTGGCATCGAGCATGAAGCCAAGAAACTGCCCAAGGAGGGCGAC
FDC1083           CAGGCCTACACCGCACTTGGCATCGAGCATGAAGCCAAGAAACTGCCCAAGGAGGGCGAC
JF90-2            CAGGCCTACACCGCACTTGGCATCGAGCATGAAGCCAAGAAACTGCCCAAGGAGGGCGAC
JF90-8            CAGGCCTACACCGCACTTGGCATCGAGCATGAAGCCAAGAAACTGCCCAAGGAGGGCGAC
JJ10-1            CAGGCCTACACCGCACTTGGCATCGAGCATGAAGCCAAGAAACTGCCCAAGGAGGGCGAC
JJ238-10          CAGGCCTACACCGCACTTGGCATCGAGCATGAAGCCAAGAAACTGCCCAAGGAGGGCGAC
JJ238-24          CAGGCCTACACCGCACTTGGCATCGAGCATGAAGCCAAGAAACTGCCCAAGGAGGGCGAC
JK2-10            CAGGCCTACACCGCACTTGGCATCGAGCATGAAGCCAAGAAACTGCCCAAGGAGGGCGAC
JK4-1             CAGGCCTACACCGCACTTGGCATCGAGCATGAAGCCAAGAAACTGCCCAAGGAGGGCGAC
JK48              CAGGCCTACACCGCACTTGGCATCGAGCATGAAGCCAAGAAACTGCCCAAGGAGGGCGAC
JK143-9           CAGGCCTACACCGCACTTGGCATCGAGCATGAAGCCAAGAAACTGCCCAAGGAGGGCGAC
JK143-11          CAGGCCTACACCGCACTTGGCATCGAGCATGAAGCCAAGAAACTGCCCAAGGAGGGCGAC
JM35              CAGGCCTACACCGCACTTGGCATCGAGCATGAAGCCAAGAAACTGCCCAAGGAGGGCGAC
JS581             CAGGCCTACACCGCACTTGGCATCGAGCATGAAGCCAAGAAACTGCCCAAGGAGGGCGAC
JS582             CAGGCCTACACCGCACTTGGCATCGAGCATGAAGCCAAGAAACTGCCCAAGGAGGGCGAC
JS584             CAGGCCTACACCGCACTTGGCATCGAGCATGAAGCCAAGAAACTGCCCAAGGAGGGCGAC
JW160-1           CAGGCCTACACCGCACTTGGCATCGAGCATGAAGCCAAGAAACTGCCCAAGGAGGGCGAC
LB100-1           CAGGCCTACACCGCACTTGGCATCGAGCATGAAGCCAAGAAACTGCCCAAGGAGGGCGAC
LB302             CAGGCCTACACCGCACTTGGCATCGAGCATGAAGCCAAGAAACTGCCCAAGGAGGGCGAC
LC80              CAGGCCTACACCGCACTTGGCATCGAGCATGAAGCCAAGAAACTGCCCAAGGAGGGCGAC
LD7-1             CAGGCCTACACCGCACTTGGCATCGAGCATGAAGCCAAGAAACTGCCCAAGGAGGGCGAC
LD71a             CAGGCCTACACCGCACTTGGCATCGAGCATGAAGCCAAGAAACTGCCCAAGGAGGGCGAC
LE3-1             CAGGCCTACACCGCACTTGGCATCGAGCATGAAGCCAAGAAACTGCCCAAGGAGGGCGAC
LE20-1            CAGGCCTACACCGCACTTGGCATCGAGCATGAAGCCAAGAAACTGCCCAAGGAGGGCGAC
LE116-1           CAGGCCTACACCGCACTTGGCATCGAGCATGAAGCCAAGAAACTGCCCAAGGAGGGCGAC
LG97              CAGGCCTACACCGCACTTGGCATCGAGCATGAAGCCAAGAAACTGCCCAAGGAGGGCGAC
LG102             CAGGCCTACACCGCACTTGGCATCGAGCATGAAGCCAAGAAACTGCCCAAGGAGGGCGAC
LG115             CAGGCCTACACCGCACTTGGCATCGAGCATGAAGCCAAGAAACTGCCCAAGGAGGGCGAC
LG117             CAGGCCTACACCGCACTTGGCATCGAGCATGAAGCCAAGAAACTGCCCAAGGAGGGCGAC
LH37-1            CAGGCCTACACCGCACTTGGCATCGAGCATGAAGCCAAGAAACTGCCCAAGGAGGGCGAC
LMG9322           CAGGCCTACACCGCACTTGGCATCGAGCATGAAGCCAAGAAACTGCCCAAGGAGGGCGAC
NCPPB3562         CAGGCCTACACCGCACTTGGCATCGAGCATGAAGCCAAGAAACTGCCCAAGGAGGGCGAC
NCPPB3607         CAGGCCTACACCGCACTTGGCATCGAGCATGAAGCCAAGAAACTGCCCAAGGAGGGCGAC
NCPPB3608         CAGGCCTACACCGCACTTGGCATCGAGCATGAAGCCAAGAAACTGCCCAAGGAGGGCGAC
NCPPB3610         CAGGCCTACACCGCACTTGGCATCGAGCATGAAGCCAAGAAACTGCCCAAGGAGGGCGAC
NCPPB3612         CAGGCCTACACCGCACTTGGCATCGAGCATGAAGCCAAGAAACTGCCCAAGGAGGGCGAC
NCPPB3615         CAGGCCTACACCGCACTTGGCATCGAGCATGAAGCCAAGAAACTGCCCAAGGAGGGCGAC
X2003-3218        CAGGCCTACACCGCACTTGGCATCGAGCATGAAGCCAAGAAACTGCCCAAGGAGGGCGAC
                  ************************************************************

C40               AAAGCGCGCCAATTGACCGCGTGGATGTGTGAAAACTTCTTCGACGTGCGCACCTTCGGT
CFBP2852          AAAGCGCGCCAATTGACCGCGTGGATGTGTGAAAACTTCTTCGACGTGCGCACCTTCGGT
CFBP2911          AAAGCGCGCCAATTGACCGCGTGGATGTGTGAAAACTTCTTCGACGTGCGCACCTTCGGT
FDC217            AAAGCGCGCCAATTGACCGCGTGGATGTGTGAAAACTTCTTCGACGTGCGCACCTTCGGT
FDC1083           AAAGCGCGCCAATTGACCGCGTGGATGTGTGAAAACTTCTTCGACGTGCGCACCTTCGGT
JF90-2            AAAGCGCGCCAATTGACCGCGTGGATGTGTGAAAACTTCTTCGACGTGCGCACCTTCGGT
JF90-8            AAAGCGCGCCAATTGACCGCGTGGATGTGTGAAAACTTCTTCGACGTGCGCACCTTCGGT
JJ10-1            AAAGCGCGCCAATTGACCGCGTGGATGTGTGAAAACTTCTTCGACGTGCGCACCTTCGGT
JJ238-10          AAAGCGCGCCAATTGACCGCGTGGATGTGTGAAAACTTCTTCGACGTGCGCACCTTCGGT
JJ238-24          AAAGCGCGCCAATTGACCGCGTGGATGTGTGAAAACTTCTTCGACGTGCGCACCTTCGGT
JK2-10            AAAGCGCGCCAATTGACCGCGTGGATGTGTGAAAACTTCTTCGACGTGCGCACCTTCGGT
JK4-1             AAAGCGCGCCAATTGACCGCGTGGATGTGTGAAAACTTCTTCGACGTGCGCACCTTCGGT
JK48              AAAGCGCGCCAATTGACCGCGTGGATGTGTGAAAACTTCTTCGACGTGCGCACCTTCGGT
JK143-9           AAAGCGCGCCAATTGACCGCGTGGATGTGTGAAAACTTCTTCGACGTGCGCACCTTCGGT
JK143-11          AAAGCGCGCCAATTGACCGCGTGGATGTGTGAAAACTTCTTCGACGTGCGCACCTTCGGT
JM35              AAAGCGCGCCAATTGACCGCGTGGATGTGTGAAAACTTCTTCGACGTGCGCACCTTCGGT
JS581             AAAGCGCGCCAATTGACCGCGTGGATGTGTGAAAACTTCTTCGACGTGCGCACCTTCGGT
JS582             AAAGCGCGCCAATTGACCGCGTGGATGTGTGAAAACTTCTTCGACGTGCGCACCTTCGGT
JS584             AAAGCGCGCCAATTGACCGCGTGGATGTGTGAAAACTTCTTCGACGTGCGCACCTTCGGT
JW160-1           AAAGCGCGCCAATTGACCGCGTGGATGTGTGAAAACTTCTTCGACGTGCGCACCTTCGGT
LB100-1           AAAGCGCGCCAATTGACCGCGTGGATGTGTGAAAACTTCTTCGACGTGCGCACCTTCGGT
LB302             AAAGCGCGCCAATTGACCGCGTGGATGTGTGAAAACTTCTTCGACGTGCGCACCTTCGGT
LC80              AAAGCGCGCCAATTGACCGCGTGGATGTGTGAAAACTTCTTCGACGTGCGCACCTTCGGT
LD7-1             AAAGCGCGCCAATTGACCGCGTGGATGTGTGAAAACTTCTTCGACGTGCGCACCTTCGGT
LD71a             AAAGCGCGCCAATTGACCGCGTGGATGTGTGAAAACTTCTTCGACGTGCGCACCTTCGGT
LE3-1             AAAGCGCGCCAATTGACCGCGTGGATGTGTGAAAACTTCTTCGACGTGCGCACCTTCGGT
LE20-1            AAAGCGCGCCAATTGACCGCGTGGATGTGTGAAAACTTCTTCGACGTGCGCACCTTCGGT
LE116-1           AAAGCGCGCCAATTGACCGCGTGGATGTGTGAAAACTTCTTCGACGTGCGCACCTTCGGT
LG97              AAAGCGCGCCAATTGACCGCGTGGATGTGTGAAAACTTCTTCGACGTGCGCACCTTCGGT
LG102             AAAGCGCGCCAATTGACCGCGTGGATGTGTGAAAACTTCTTCGACGTGCGCACCTTCGGT
LG115             AAAGCGCGCCAATTGACCGCGTGGATGTGTGAAAACTTCTTCGACGTGCGCACCTTCGGT
LG117             AAAGCGCGCCAATTGACCGCGTGGATGTGTGAAAACTTCTTCGACGTGCGCACCTTCGGT
LH37-1            AAAGCGCGCCAATTGACCGCGTGGATGTGTGAAAACTTCTTCGACGTGCGCACCTTCGGT
LMG9322           AAAGCGCGCCAATTGACCGCGTGGATGTGTGAAAACTTCTTCGACGTGCGCACCTTCGGT
NCPPB3562         AAAGCGCGCCAATTGACCGCGTGGATGTGTGAAAACTTCTTCGACGTGCGCACCTTCGGT
NCPPB3607         AAAGCGCGCCAATTGACCGCGTGGATGTGTGAAAACTTCTTCGACGTGCGCACCTTCGGT
NCPPB3608         AAAGCGCGCCAATTGACCGCGTGGATGTGTGAAAACTTCTTCGACGTGCGCACCTTCGGT
NCPPB3610         AAAGCGCGCCAATTGACCGCGTGGATGTGTGAAAACTTCTTCGACGTGCGCACCTTCGGT
NCPPB3612         AAAGCGCGCCAATTGACCGCGTGGATGTGTGAAAACTTCTTCGACGTGCGCACCTTCGGT
NCPPB3615         AAAGCGCGCCAATTGACCGCGTGGATGTGTGAAAACTTCTTCGACGTGCGCACCTTCGGT
X2003-3218        AAAGCGCGCCAATTGACCGCGTGGATGTGTGAAAACTTCTTCGACGTGCGCACCTTCGGT
                  ************************************************************
```

```
C40             GCGGTGATGACCACCGAAGTCAATACCGGCCAGGTCCGCGGCCCGGTGCAACTGGCCTTC
CFBP2852        GCGGTGATGACCACCGAAGTCAATACCGGCCAGGTCCGCGGCCCGGTGCAACTGGCCTTC
CFBP2911        GCGGTGATGACCACCGAAGTCAATACCGGCCAGGTCCGCGGCCCGGTGCAACTGGCCTTC
FDC217          GCGGTGATGACCACCGAAGTCAATACCGGCCAGGTCCGCGGCCCGGTGCAACTGGCCTTC
FDC1083         GCGGTGATGACCACCGAAGTCAATACCGGCCAGGTCCGCGGCCCGGTGCAACTGGCCTTC
JF90-2          GCGGTGATGACCACCGAAGTCAATACCGGCCAGGTCCGCGGCCCGGTGCAACTGGCCTTC
JF90-8          GCGGTGATGACCACCGAAGTCAATACCGGCCAGGTCCGCGGCCCGGTGCAACTGGCCTTC
JJ10-1          GCGGTGATGACCACCGAAGTCAATACCGGCCAGGTCCGCGGCCCGGTGCAACTGGCCTTC
JJ238-10        GCGGTGATGACCACCGAAGTCAATACCGGCCAGGTCCGCGGCCCGGTGCAACTGGCCTTC
JJ238-24        GCGGTGATGACCACCGAAGTCAATACCGGCCAGGTCCGCGGCCCGGTGCAACTGGCCTTC
JK2-10          GCGGTGATGACCACCGAAGTCAATACCGGCCAGGTCCGCGGCCCGGTGCAACTGGCCTTC
JK4-1           GCGGTGATGACCACCGAAGTCAATACCGGCCAGGTCCGCGGCCCGGTGCAACTGGCCTTC
JK48            GCGGTGATGACCACCGAAGTCAATACCGGCCAGGTCCGCGGCCCGGTGCAACTGGCCTTC
JK143-9         GCGGTGATGACCACCGAAGTCAATACCGGCCAGGTCCGCGGCCCGGTGCAACTGGCCTTC
JK143-11        GCGGTGATGACCACCGAAGTCAATACCGGCCAGGTCCGCGGCCCGGTGCAACTGGCCTTC
JM35            GCGGTGATGACCACCGAAGTCAATACCGGCCAGGTCCGCGGCCCGGTGCAACTGGCCTTC
JS581           GCGGTGATGACCACCGAAGTCAATACCGGCCAGGTCCGCGGCCCGGTGCAACTGGCCTTC
JS582           GCGGTGATGACCACCGAAGTCAATACCGGCCAGGTCCGCGGCCCGGTGCAACTGGCCTTC
JS584           GCGGTGATGACCACCGAAGTCAATACCGGCCAGGTCCGCGGCCCGGTGCAACTGGCCTTC
JW160-1         GCGGTGATGACCACCGAAGTCAATACCGGCCAGGTCCGCGGCCCGGTGCAACTGGCCTTC
LB100-1         GCGGTGATGACCACCGAAGTCAATACCGGCCAGGTCCGCGGCCCGGTGCAACTGGCCTTC
LB302           GCGGTGATGACCACCGAAGTCAATACCGGCCAGGTCCGCGGCCCGGTGCAACTGGCCTTC
LC80            GCGGTGATGACCACCGAAGTCAATACCGGCCAGGTCCGCGGCCCGGTGCAACTGGCCTTC
LD7-1           GCGGTGATGACCACCGAAGTCAATACCGGCCAGGTCCGCGGCCCGGTGCAACTGGCCTTC
LD71a           GCGGTGATGACCACCGAAGTCAATACCGGCCAGGTCCGCGGCCCGGTGCAACTGGCCTTC
LE3-1           GCGGTGATGACCACCGAAGTCAATACCGGCCAGGTCCGCGGCCCGGTGCAACTGGCCTTC
LE20-1          GCGGTGATGACCACCGAAGTCAATACCGGCCAGGTCCGCGGCCCGGTGCAACTGGCCTTC
LE116-1         GCGGTGATGACCACCGAAGTCAATACCGGCCAGGTCCGCGGCCCGGTGCAACTGGCCTTC
LG97            GCGGTGATGACCACCGAAGTCAATACCGGCCAGGTCCGCGGCCCGGTGCAACTGGCCTTC
LG102           GCGGTGATGACCACCGAAGTCAATACCGGCCAGGTCCGCGGCCCGGTGCAACTGGCCTTC
LG115           GCGGTGATGACCACCGAAGTCAATACCGGCCAGGTCCGCGGCCCGGTGCAACTGGCCTTC
LG117           GCGGTGATGACCACCGAAGTCAATACCGGCCAGGTCCGCGGCCCGGTGCAACTGGCCTTC
LH37-1          GCGGTGATGACCACCGAAGTCAATACCGGCCAGGTCCGCGGCCCGGTGCAACTGGCCTTC
LMG9322         GCGGTGATGACCACCGAAGTCAATACCGGCCAGGTCCGCGGCCCGGTGCAACTGGCCTTC
NCPPB3562       GCGGTGATGACCACCGAAGTCAATACCGGCCAGGTCCGCGGCCCGGTGCAACTGGCCTTC
NCPPB3607       GCGGTGATGACCACCGAAGTCAATACCGGCCAGGTCCGCGGCCCGGTGCAACTGGCCTTC
NCPPB3608       GCGGTGATGACCACCGAAGTCAATACCGGCCAGGTCCGCGGCCCGGTGCAACTGGCCTTC
NCPPB3610       GCGGTGATGACCACCGAAGTCAATACCGGCCAGGTCCGCGGCCCGGTGCAACTGGCCTTC
NCPPB3612       GCGGTGATGACCACCGAAGTCAATACCGGCCAGGTCCGCGGCCCGGTGCAACTGGCCTTC
NCPPB3615       GCGGTGATGACCACCGAAGTCAATACCGGCCAGGTCCGCGGCCCGGTGCAACTGGCCTTC
X2003-3218      GCGGTGATGACCACCGAAGTCAATACCGGCCAGGTCCGCGGCCCGGTGCAACTGGCCTTC
                ************************************************************
```

```
C40             GCCACTTCGGTCGAACCGGTATTGCCACTGGAAGTATCGATCACCCGCGTGGCGGTGACC
CFBP2852        GCCACTTCGGTCGAACCGGTATTGCCACTGGAAGTATCGATCACCCGCGTGGCGGTGACC
CFBP2911        GCCACTTCGGTCGAACCGGTATTGCCACTGGAAGTATCGATCACCCGCGTGGCGGTGACC
FDC217          GCCACTTCGGTCGAACCGGTATTGCCACTGGAAGTATCGATCACCCGCGTGGCGGTGACC
FDC1083         GCCACTTCGGTCGAACCGGTATTGCCACTGGAAGTATCGATCACCCGCGTGGCGGTGACC
JF90-2          GCCACTTCGGTCGAACCGGTATTGCCACTGGAAGTATCGATCACCCGCGTGGCGGTGACC
JF90-8          GCCACTTCGGTCGAACCGGTATTGCCACTGGAAGTATCGATCACCCGCGTGGCGGTGACC
JJ10-1          GCCACTTCGGTCGAACCGGTATTGCCACTGGAAGTATCGATCACCCGCGTGGCGGTGACC
JJ238-10        GCCACTTCGGTCGAACCGGTATTGCCACTGGAAGTATCGATCACCCGCGTGGCGGTGACC
JJ238-24        GCCACTTCGGTCGAACCGGTATTGCCACTGGAAGTATCGATCACCCGCGTGGCGGTGACC
JK2-10          GCCACTTCGGTCGAACCGGTATTGCCACTGGAAGTATCGATCACCCGCGTGGCGGTGACC
JK4-1           GCCACTTCGGTCGAACCGGTATTGCCACTGGAAGTATCGATCACCCGCGTGGCGGTGACC
JK48            GCCACTTCGGTCGAACCGGTATTGCCACTGGAAGTATCGATCACCCGCGTGGCGGTGACC
JK143-9         GCCACTTCGGTCGAACCGGTATTGCCACTGGAAGTATCGATCACCCGCGTGGCGGTGACC
JK143-11        GCCACTTCGGTCGAACCGGTATTGCCACTGGAAGTATCGATCACCCGCGTGGCGGTGACC
JM35            GCCACTTCGGTCGAACCGGTATTGCCACTGGAAGTATCGATCACCCGCGTGGCGGTGACC
JS581           GCCACTTCGGTCGAACCGGTATTGCCACTGGAAGTATCGATCACCCGCGTGGCGGTGACC
JS582           GCCACTTCGGTCGAACCGGTATTGCCACTGGAAGTATCGATCACCCGCGTGGCGGTGACC
JS584           GCCACTTCGGTCGAACCGGTATTGCCACTGGAAGTATCGATCACCCGCGTGGCGGTGACC
JW160-1         GCCACTTCGGTCGAACCGGTATTGCCACTGGAAGTATCGATCACCCGCGTGGCGGTGACC
LB100-1         GCCACTTCGGTCGAACCGGTATTGCCACTGGAAGTATCGATCACCCGCGTGGCGGTGACC
LB302           GCCACTTCGGTCGAACCGGTATTGCCACTGGAAGTATCGATCACCCGCGTGGCGGTGACC
LC80            GCCACTTCGGTCGAACCGGTATTGCCACTGGAAGTATCGATCACCCGCGTGGCGGTGACC
LD7-1           GCCACTTCGGTCGAACCGGTATTGCCACTGGAAGTATCGATCACCCGCGTGGCGGTGACC
LD71a           GCCACTTCGGTCGAACCGGTATTGCCACTGGAAGTATCGATCACCCGCGTGGCGGTGACC
LE3-1           GCCACTTCGGTCGAACCGGTATTGCCACTGGAAGTATCGATCACCCGCGTGGCGGTGACC
LE20-1          GCCACTTCGGTCGAACCGGTATTGCCACTGGAAGTATCGATCACCCGCGTGGCGGTGACC
LE116-1         GCCACTTCGGTCGAACCGGTATTGCCACTGGAAGTATCGATCACCCGCGTGGCGGTGACC
LG97            GCCACTTCGGTCGAACCGGTATTGCCACTGGAAGTATCGATCACCCGCGTGGCGGTGACC
LG102           GCCACTTCGGTCGAACCGGTATTGCCACTGGAAGTATCGATCACCCGCGTGGCGGTGACC
LG115           GCCACTTCGGTCGAACCGGTATTGCCACTGGAAGTATCGATCACCCGCGTGGCGGTGACC
LG117           GCCACTTCGGTCGAACCGGTATTGCCACTGGAAGTATCGATCACCCGCGTGGCGGTGACC
LH37-1          GCCACTTCGGTCGAACCGGTATTGCCACTGGAAGTATCGATCACCCGCGTGGCGGTGACC
LMG9322         GCCACTTCGGTCGAACCGGTATTGCCACTGGAAGTATCGATCACCCGCGTGGCGGTGACC
NCPPB3562       GCCACTTCGGTCGAACCGGTATTGCCACTGGAAGTATCGATCACCCGCGTGGCGGTGACC
NCPPB3607       GCCACTTCGGTCGAACCGGTATTGCCACTGGAAGTATCGATCACCCGCGTGGCGGTGACC
NCPPB3608       GCCACTTCGGTCGAACCGGTATTGCCACTGGAAGTATCGATCACCCGCGTGGCGGTGACC
NCPPB3610       GCCACTTCGGTCGAACCGGTATTGCCACTGGAAGTATCGATCACCCGCGTGGCGGTGACC
NCPPB3612       GCCACTTCGGTCGAACCGGTATTGCCACTGGAAGTATCGATCACCCGCGTGGCGGTGACC
NCPPB3615       GCCACTTCGGTCGAACCGGTATTGCCACTGGAAGTATCGATCACCCGCGTGGCGGTGACC
X2003-3218      GCCACTTCGGTCGAACCGGTATTGCCACTGGAAGTATCGATCACCCGCGTGGCGGTGACC
                ************************************************************
```

```
C40             AACGAAAAAGACCTGGAGAAGGAGCGCACCATGGGCCGCAAGCACATTCTGCCCTACGGC
CFBP2852        AACGAAAAAGACCTGGAGAAGGAGCGCACCATGGGCCGCAAGCACATTCTGCCCTACGGC
CFBP2911        AACGAAAAAGACCTGGAGAAGGAGCGCACCATGGGCCGCAAGCACATTCTGCCCTACGGC
FDC217          AACGAAAAAGACCTGGAGAAGGAGCGCACCATGGGCCGCAAGCACATTCTGCCCTACGGC
FDC1083         AACGAAAAAGACCTGGAGAAGGAGCGCACCATGGGCCGCAAGCACATTCTGCCCTACGGC
JF90-2          AACGAAAAAGACCTGGAGAAGGAGCGCACCATGGGCCGCAAGCACATTCTGCCCTACGGC
JF90-8          AACGAAAAAGACCTGGAGAAGGAGCGCACCATGGGCCGCAAGCACATTCTGCCCTACGGC
JJ10-1          AACGAAAAAGACCTGGAGAAGGAGCGCACCATGGGCCGCAAGCACATTCTGCCCTACGGC
JJ238-10        AACGAAAAAGACCTGGAGAAGGAGCGCACCATGGGCCGCAAGCACATTCTGCCCTACGGC
JJ238-24        AACGAAAAAGACCTGGAGAAGGAGCGCACCATGGGCCGCAAGCACATTCTGCCCTACGGC
JK2-10          AACGAAAAAGACCTGGAGAAGGAGCGCACCATGGGCCGCAAGCACATTCTGCCCTACGGC
JK4-1           AACGAAAAAGACCTGGAGAAGGAGCGCACCATGGGCCGCAAGCACATTCTGCCCTACGGC
JK48            AACGAAAAAGACCTGGAGAAGGAGCGCACCATGGGCCGCAAGCACATTCTGCCCTACGGC
JK143-9         AACGAAAAAGACCTGGAGAAGGAGCGCACCATGGGCCGCAAGCACATTCTGCCCTACGGC
JK143-11        AACGAAAAAGACCTGGAGAAGGAGCGCACCATGGGCCGCAAGCACATTCTGCCCTACGGC
JM35            AACGAAAAAGACCTGGAGAAGGAGCGCACCATGGGCCGCAAGCACATTCTGCCCTACGGC
JS581           AACGAAAAAGACCTGGAGAAGGAGCGCACCATGGGCCGCAAGCACATTCTGCCCTACGGC
JS582           AACGAAAAAGACCTGGAGAAGGAGCGCACCATGGGCCGCAAGCACATTCTGCCCTACGGC
JS584           AACGAAAAAGACCTGGAGAAGGAGCGCACCATGGGCCGCAAGCACATTCTGCCCTACGGC
JW160-1         AACGAAAAAGACCTGGAGAAGGAGCGCACCATGGGCCGCAAGCACATTCTGCCCTACGGC
LB100-1         AACGAAAAAGACCTGGAGAAGGAGCGCACCATGGGCCGCAAGCACATTCTGCCCTACGGC
LB302           AACGAAAAAGACCTGGAGAAGGAGCGCACCATGGGCCGCAAGCACATTCTGCCCTACGGC
LC80            AACGAAAAAGACCTGGAGAAGGAGCGCACCATGGGCCGCAAGCACATTCTGCCCTACGGC
LD7-1           AACGAAAAAGACCTGGAGAAGGAGCGCACCATGGGCCGCAAGCACATTCTGCCCTACGGC
LD71a           AACGAAAAAGACCTGGAGAAGGAGCGCACCATGGGCCGCAAGCACATTCTGCCCTACGGC
LE3-1           AACGAAAAAGACCTGGAGAAGGAGCGCACCATGGGCCGCAAGCACATTCTGCCCTACGGC
LE20-1          AACGAAAAAGACCTGGAGAAGGAGCGCACCATGGGCCGCAAGCACATTCTGCCCTACGGC
LE116-1         AACGAAAAAGACCTGGAGAAGGAGCGCACCATGGGCCGCAAGCACATTCTGCCCTACGGC
LG97            AACGAAAAAGACCTGGAGAAGGAGCGCACCATGGGCCGCAAGCACATTCTGCCCTACGGC
LG102           AACGAAAAAGACCTGGAGAAGGAGCGCACCATGGGCCGCAAGCACATTCTGCCCTACGGC
LG115           AACGAAAAAGACCTGGAGAAGGAGCGCACCATGGGCCGCAAGCACATTCTGCCCTACGGC
LG117           AACGAAAAAGACCTGGAGAAGGAGCGCACCATGGGCCGCAAGCACATTCTGCCCTACGGC
LH37-1          AACGAAAAAGACCTGGAGAAGGAGCGCACCATGGGCCGCAAGCACATTCTGCCCTACGGC
LMG9322         AACGAAAAAGACCTGGAGAAGGAGCGCACCATGGGCCGCAAGCACATTCTGCCCTACGGC
NCPPB3562       AACGAAAAAGACCTGGAGAAGGAGCGCACCATGGGCCGCAAGCACATTCTGCCCTACGGC
NCPPB3607       AACGAAAAAGACCTGGAGAAGGAGCGCACCATGGGCCGCAAGCACATTCTGCCCTACGGC
NCPPB3608       AACGAAAAAGACCTGGAGAAGGAGCGCACCATGGGCCGCAAGCACATTCTGCCCTACGGC
NCPPB3610       AACGAAAAAGACCTGGAGAAGGAGCGCACCATGGGCCGCAAGCACATTCTGCCCTACGGC
NCPPB3612       AACGAAAAAGACCTGGAGAAGGAGCGCACCATGGGCCGCAAGCACATTCTGCCCTACGGC
NCPPB3615       AACGAAAAAGACCTGGAGAAGGAGCGCACCATGGGCCGCAAGCACATTCTGCCCTACGGC
X2003-3218      AACGAAAAAGACCTGGAGAAGGAGCGCACCATGGGCCGCAAGCACATTCTGCCCTACGGC
                ************************************************************

C40             CTGTATCGCGCGCATGGCTTTGTGTCGGCAAAGCTGGCCGAGCGCACCGGGTTCTCCGAG
CFBP2852        CTGTATCGCGCGCATGGCTTTGTGTCGGCAAAGCTGGCCGAGCGCACCGGGTTCTCCGAG
CFBP2911        CTGTATCGCGCGCATGGCTTTGTGTCGGCAAAGCTGGCCGAGCGCACCGGGTTCTCCGAG
FDC217          CTGTATCGCGCGCATGGCTTTGTGTCGGCAAAGCTGGCCGAGCGCACCGGGTTCTCCGAG
FDC1083         CTGTATCGCGCGCATGGCTTTGTGTCGGCAAAGCTGGCCGAGCGCACCGGGTTCTCCGAG
JF90-2          CTGTATCGCGCGCATGGCTTTGTGTCGGCAAAGCTGGCCGAGCGCACCGGGTTCTCCGAG
JF90-8          CTGTATCGCGCGCATGGCTTTGTGTCGGCAAAGCTGGCCGAGCGCACCGGGTTCTCCGAG
JJ10-1          CTGTATCGCGCGCATGGCTTTGTGTCGGCAAAGCTGGCCGAGCGCACCGGGTTCTCCGAG
JJ238-10        CTGTATCGCGCGCATGGCTTTGTGTCGGCAAAGCTGGCCGAGCGCACCGGGTTCTCCGAG
JJ238-24        CTGTATCGCGCGCATGGCTTTGTGTCGGCAAAGCTGGCCGAGCGCACCGGGTTCTCCGAG
JK2-10          CTGTATCGCGCGCATGGCTTTGTGTCGGCAAAGCTGGCCGAGCGCACCGGGTTCTCCGAG
JK4-1           CTGTATCGCGCGCATGGCTTTGTGTCGGCAAAGCTGGCCGAGCGCACCGGGTTCTCCGAG
JK48            CTGTATCGCGCGCATGGCTTTGTGTCGGCAAAGCTGGCCGAGCGCACCGGGTTCTCCGAG
JK143-9         CTGTATCGCGCGCATGGCTTTGTGTCGGCAAAGCTGGCCGAGCGCACCGGGTTCTCCGAG
JK143-11        CTGTATCGCGCGCATGGCTTTGTGTCGGCAAAGCTGGCCGAGCGCACCGGGTTCTCCGAG
JM35            CTGTATCGCGCGCATGGCTTTGTGTCGGCAAAGCTGGCCGAGCGCACCGGGTTCTCCGAG
JS581           CTGTATCGCGCGCATGGCTTTGTGTCGGCAAAGCTGGCCGAGCGCACCGGGTTCTCCGAG
JS582           CTGTATCGCGCGCATGGCTTTGTGTCGGCAAAGCTGGCCGAGCGCACCGGGTTCTCCGAG
JS584           CTGTATCGCGCGCATGGCTTTGTGTCGGCAAAGCTGGCCGAGCGCACCGGGTTCTCCGAG
JW160-1         CTGTATCGCGCGCATGGCTTTGTGTCGGCAAAGCTGGCCGAGCGCACCGGGTTCTCCGAG
LB100-1         CTGTATCGCGCGCATGGCTTTGTGTCGGCAAAGCTGGCCGAGCGCACCGGGTTCTCCGAG
LB302           CTGTATCGCGCGCATGGCTTTGTGTCGGCAAAGCTGGCCGAGCGCACCGGGTTCTCCGAG
LC80            CTGTATCGCGCGCATGGCTTTGTGTCGGCAAAGCTGGCCGAGCGCACCGGGTTCTCCGAG
LD7-1           CTGTATCGCGCGCATGGCTTTGTGTCGGCAAAGCTGGCCGAGCGCACCGGGTTCTCCGAG
LD71a           CTGTATCGCGCGCATGGCTTTGTGTCGGCAAAGCTGGCCGAGCGCACCGGGTTCTCCGAG
LE3-1           CTGTATCGCGCGCATGGCTTTGTGTCGGCAAAGCTGGCCGAGCGCACCGGGTTCTCCGAG
LE20-1          CTGTATCGCGCGCATGGCTTTGTGTCGGCAAAGCTGGCCGAGCGCACCGGGTTCTCCGAG
LE116-1         CTGTATCGCGCGCATGGCTTTGTGTCGGCAAAGCTGGCCGAGCGCACCGGGTTCTCCGAG
LG97            CTGTATCGCGCGCATGGCTTTGTGTCGGCAAAGCTGGCCGAGCGCACCGGGTTCTCCGAG
LG102           CTGTATCGCGCGCATGGCTTTGTGTCGGCAAAGCTGGCCGAGCGCACCGGGTTCTCCGAG
LG115           CTGTATCGCGCGCATGGCTTTGTGTCGGCAAAGCTGGCCGAGCGCACCGGGTTCTCCGAG
LG117           CTGTATCGCGCGCATGGCTTTGTGTCGGCAAAGCTGGCCGAGCGCACCGGGTTCTCCGAG
LH37-1          CTGTATCGCGCGCATGGCTTTGTGTCGGCAAAGCTGGCCGAGCGCACCGGGTTCTCCGAG
LMG9322         CTGTATCGCGCGCATGGCTTTGTGTCGGCAAAGCTGGCCGAGCGCACCGGGTTCTCCGAG
NCPPB3562       CTGTATCGCGCGCATGGCTTTGTGTCGGCAAAGCTGGCCGAGCGCACCGGGTTCTCCGAG
NCPPB3607       CTGTATCGCGCGCATGGCTTTGTGTCGGCAAAGCTGGCCGAGCGCACCGGGTTCTCCGAG
NCPPB3608       CTGTATCGCGCGCATGGCTTTGTGTCGGCAAAGCTGGCCGAGCGCACCGGGTTCTCCGAG
NCPPB3610       CTGTATCGCGCGCATGGCTTTGTGTCGGCAAAGCTGGCCGAGCGCACCGGGTTCTCCGAG
NCPPB3612       CTGTATCGCGCGCATGGCTTTGTGTCGGCAAAGCTGGCCGAGCGCACCGGGTTCTCCGAG
NCPPB3615       CTGTATCGCGCGCATGGCTTTGTGTCGGCAAAGCTGGCCGAGCGCACCGGGTTCTCCGAG
X2003-3218      CTGTATCGCGCGCATGGCTTTGTGTCGGCAAAGCTGGCCGAGCGCACCGGGTTCTCCGAG
                ************************************************************
```

```
C40             GAAGACCTGCAACTGCTGTGGCGCGCATTGACCAATCTATTCGAGCACGACCGCTCCGCC
CFBP2852        GAAGACCTGCAACTGCTGTGGCGCGCATTGACCAATCTATTCGAGCACGACCGCTCCGCC
CFBP2911        GAAGACCTGCAACTGCTGTGGCGCGCATTGACCAATCTATTCGAGCACGACCGCTCCGCC
FDC217          GAAGACCTGCAACTGCTGTGGCGCGCATTGACCAATCTATTCGAGCACGACCGCTCCGCC
FDC1083         GAAGACCTGCAACTGCTGTGGCGCGCATTGACCAATCTATTCGAGCACGACCGCTCCGCC
JF90-2          GAAGACCTGCAACTGCTGTGGCGCGCATTGACCAATCTATTCGAGCACGACCGCTCCGCC
JF90-8          GAAGACCTGCAACTGCTGTGGCGCGCATTGACCAATCTATTCGAGCACGACCGCTCCGCC
JJ10-1          GAAGACCTGCAACTGCTGTGGCGCGCATTGACCAATCTATTCGAGCACGACCGCTCCGCC
JJ238-10        GAAGACCTGCAACTGCTGTGGCGCGCATTGACCAATCTATTCGAGCACGACCGCTCCGCC
JJ238-24        GAAGACCTGCAACTGCTGTGGCGCGCATTGACCAATCTATTCGAGCACGACCGCTCCGCC
JK2-10          GAAGACCTGCAACTGCTGTGGCGCGCATTGACCAATCTATTCGAGCACGACCGCTCCGCC
JK4-1           GAAGACCTGCAACTGCTGTGGCGCGCATTGACCAATCTATTCGAGCACGACCGCTCCGCC
JK48            GAAGACCTGCAACTGCTGTGGCGCGCATTGACCAATCTATTCGAGCACGACCGCTCCGCC
JK143-9         GAAGACCTGCAACTGCTGTGGCGCGCATTGACCAATCTATTCGAGCACGACCGCTCCGCC
JK143-11        GAAGACCTGCAACTGCTGTGGCGCGCATTGACCAATCTATTCGAGCACGACCGCTCCGCC
JM35            GAAGACCTGCAACTGCTGTGGCGCGCATTGACCAATCTATTCGAGCACGACCGCTCCGCC
JS581           GAAGACCTGCAACTGCTGTGGCGCGCATTGACCAATCTATTCGAGCACGACCGCTCCGCC
JS582           GAAGACCTGCAACTGCTGTGGCGCGCATTGACCAATCTATTCGAGCACGACCGCTCCGCC
JS584           GAAGACCTGCAACTGCTGTGGCGCGCATTGACCAATCTATTCGAGCACGACCGCTCCGCC
JW160-1         GAAGACCTGCAACTGCTGTGGCGCGCATTGACCAATCTATTCGAGCACGACCGCTCCGCC
LB100-1         GAAGACCTGCAACTGCTGTGGCGCGCATTGACCAATCTATTCGAGCACGACCGCTCCGCC
LB302           GAAGACCTGCAACTGCTGTGGCGCGCATTGACCAATCTATTCGAGCACGACCGCTCCGCC
LC80            GAAGACCTGCAACTGCTGTGGCGCGCATTGACCAATCTATTCGAGCACGACCGCTCCGCC
LD7-1           GAAGACCTGCAACTGCTGTGGCGCGCATTGACCAATCTATTCGAGCACGACCGCTCCGCC
LD71a           GAAGACCTGCAACTGCTGTGGCGCGCATTGACCAATCTATTCGAGCACGACCGCTCCGCC
LE3-1           GAAGACCTGCAACTGCTGTGGCGCGCATTGACCAATCTATTCGAGCACGACCGCTCCGCC
LE20-1          GAAGACCTGCAACTGCTGTGGCGCGCATTGACCAATCTATTCGAGCACGACCGCTCCGCC
LE116-1         GAAGACCTGCAACTGCTGTGGCGCGCATTGACCAATCTATTCGAGCACGACCGCTCCGCC
LG97            GAAGACCTGCAACTGCTGTGGCGCGCATTGACCAATCTATTCGAGCACGACCGCTCCGCC
LG102           GAAGACCTGCAACTGCTGTGGCGCGCATTGACCAATCTATTCGAGCACGACCGCTCCGCC
LG115           GAAGACCTGCAACTGCTGTGGCGCGCATTGACCAATCTATTCGAGCACGACCGCTCCGCC
LG117           GAAGACCTGCAACTGCTGTGGCGCGCATTGACCAATCTATTCGAGCACGACCGCTCCGCC
LH37-1          GAAGACCTGCAACTGCTGTGGCGCGCATTGACCAATCTATTCGAGCACGACCGCTCCGCC
LMG9322         GAAGACCTGCAACTGCTGTGGCGCGCATTGACCAATCTATTCGAGCACGACCGCTCCGCC
NCPPB3562       GAAGACCTGCAACTGCTGTGGCGCGCATTGACCAATCTATTCGAGCACGACCGCTCCGCC
NCPPB3607       GAAGACCTGCAACTGCTGTGGCGCGCATTGACCAATCTATTCGAGCACGACCGCTCCGCC
NCPPB3608       GAAGACCTGCAACTGCTGTGGCGCGCATTGACCAATCTATTCGAGCACGACCGCTCCGCC
NCPPB3610       GAAGACCTGCAACTGCTGTGGCGCGCATTGACCAATCTATTCGAGCACGACCGCTCCGCC
NCPPB3612       GAAGACCTGCAACTGCTGTGGCGCGCATTGACCAATCTATTCGAGCACGACCGCTCCGCC
NCPPB3615       GAAGACCTGCAACTGCTGTGGCGCGCATTGACCAATCTATTCGAGCACGACCGCTCCGCC
X2003-3218      GAAGACCTGCAACTGCTGTGGCGCGCATTGACCAATCTATTCGAGCACGACCGCTCCGCC
                ************************************************************

C40             GCGCGCGGCGAGATGGCCGCACGCAAACTGATCGTATTCGAACACGAACACCCGATGGGC
CFBP2852        GCGCGCGGCGAGATGGCCGCACGCAAACTGATCGTATTCGAACACGAACACCCGATGGGC
CFBP2911        GCGCGCGGCGAGATGGCCGCACGCAAACTGATCGTATTCGAACACGAACACCCGATGGGC
FDC217          GCGCGCGGCGAGATGGCCGCACGCAAACTGATCGTATTCGAACACGAACACCCGATGGGC
FDC1083         GCGCGCGGCGAGATGGCCGCACGCAAACTGATCGTATTCGAACACGAACACCCGATGGGC
JF90-2          GCGCGCGGCGAGATGGCCGCACGCAAACTGATCGTATTCGAACACGAACACCCGATGGGC
JF90-8          GCGCGCGGCGAGATGGCCGCACGCAAACTGATCGTATTCGAACACGAACACCCGATGGGC
JJ10-1          GCGCGCGGCGAGATGGCCGCACGCAAACTGATCGTATTCGAACACGAACACCCGATGGGC
JJ238-10        GCGCGCGGCGAGATGGCCGCACGCAAACTGATCGTATTCGAACACGAACACCCGATGGGC
JJ238-24        GCGCGCGGCGAGATGGCCGCACGCAAACTGATCGTATTCGAACACGAACACCCGATGGGC
JK2-10          GCGCGCGGCGAGATGGCCGCACGCAAACTGATCGTATTCGAACACGAACACCCGATGGGC
JK4-1           GCGCGCGGCGAGATGGCCGCACGCAAACTGATCGTATTCGAACACGAACACCCGATGGGC
JK48            GCGCGCGGCGAGATGGCCGCACGCAAACTGATCGTATTCGAACACGAACACCCGATGGGC
JK143-9         GCGCGCGGCGAGATGGCCGCACGCAAACTGATCGTATTCGAACACGAACACCCGATGGGC
JK143-11        GCGCGCGGCGAGATGGCCGCACGCAAACTGATCGTATTCGAACACGAACACCCGATGGGC
JM35            GCGCGCGGCGAGATGGCCGCACGCAAACTGATCGTATTCGAACACGAACACCCGATGGGC
JS581           GCGCGCGGCGAGATGGCCGCACGCAAACTGATCGTATTCGAACACGAACACCCGATGGGC
JS582           GCGCGCGGCGAGATGGCCGCACGCAAACTGATCGTATTCGAACACGAACACCCGATGGGC
JS584           GCGCGCGGCGAGATGGCCGCACGCAAACTGATCGTATTCGAACACGAACACCCGATGGGC
JW160-1         GCGCGCGGCGAGATGGCCGCACGCAAACTGATCGTATTCGAACACGAACACCCGATGGGC
LB100-1         GCGCGCGGCGAGATGGCCGCACGCAAACTGATCGTATTCGAACACGAACACCCGATGGGC
LB302           GCGCGCGGCGAGATGGCCGCACGCAAACTGATCGTATTCGAACACGAACACCCGATGGGC
LC80            GCGCGCGGCGAGATGGCCGCACGCAAACTGATCGTATTCGAACACGAACACCCGATGGGC
LD7-1           GCGCGCGGCGAGATGGCCGCACGCAAACTGATCGTATTCGAACACGAACACCCGATGGGC
LD71a           GCGCGCGGCGAGATGGCCGCACGCAAACTGATCGTATTCGAACACGAACACCCGATGGGC
LE3-1           GCGCGCGGCGAGATGGCCGCACGCAAACTGATCGTATTCGAACACGAACACCCGATGGGC
LE20-1          GCGCGCGGCGAGATGGCCGCACGCAAACTGATCGTATTCGAACACGAACACCCGATGGGC
LE116-1         GCGCGCGGCGAGATGGCCGCACGCAAACTGATCGTATTCGAACACGAACACCCGATGGGC
LG97            GCGCGCGGCGAGATGGCCGCACGCAAACTGATCGTATTCGAACACGAACACCCGATGGGC
LG102           GCGCGCGGCGAGATGGCCGCACGCAAACTGATCGTATTCGAACACGAACACCCGATGGGC
LG115           GCGCGCGGCGAGATGGCCGCACGCAAACTGATCGTATTCGAACACGAACACCCGATGGGC
LG117           GCGCGCGGCGAGATGGCCGCACGCAAACTGATCGTATTCGAACACGAACACCCGATGGGC
LH37-1          GCGCGCGGCGAGATGGCCGCACGCAAACTGATCGTATTCGAACACGAACACCCGATGGGC
LMG9322         GCGCGCGGCGAGATGGCCGCACGCAAACTGATCGTATTCGAACACGAACACCCGATGGGC
NCPPB3562       GCGCGCGGCGAGATGGCCGCACGCAAACTGATCGTATTCGAACACGAACACCCGATGGGC
NCPPB3607       GCGCGCGGCGAGATGGCCGCACGCAAACTGATCGTATTCGAACACGAACACCCGATGGGC
NCPPB3608       GCGCGCGGCGAGATGGCCGCACGCAAACTGATCGTATTCGAACACGAACACCCGATGGGC
NCPPB3610       GCGCGCGGCGAGATGGCCGCACGCAAACTGATCGTATTCGAACACGAACACCCGATGGGC
NCPPB3612       GCGCGCGGCGAGATGGCCGCACGCAAACTGATCGTATTCGAACACGAACACCCGATGGGC
NCPPB3615       GCGCGCGGCGAGATGGCCGCACGCAAACTGATCGTATTCGAACACGAACACCCGATGGGC
X2003-3218      GCGCGCGGCGAGATGGCCGCACGCAAACTGATCGTATTCGAACACGAACACCCGATGGGC
                ************************************************************
```

```
C40             AACGCACCGGCGCATGTGCTCTTCGACAAGGTCAAGGTCGAGCGTATTGACCAGGCGGAC
CFBP2852        AACGCACCGGCGCATGTGCTCTTCGACAAGGTCAAGGTCGAGCGTATTGACCAGGCGGAC
CFBP2911        AACGCACCGGCGCATGTGCTCTTCGACAAGGTCAAGGTCGAGCGTATTGACCAGGCGGAC
FDC217          AACGCACCGGCGCATGTGCTCTTCGACAAGGTCAAGGTCGAGCGTATTGACCAGGCGGAC
FDC1083         AACGCACCGGCGCATGTGCTCTTCGACAAGGTCAAGGTCGAGCGTATTGACCAGGCGGAC
JF90-2          AACGCACCGGCGCATGTGCTCTTCGACAAGGTCAAGGTCGAGCGTATTGACCAGGCGGAC
JF90-8          AACGCACCGGCGCATGTGCTCTTCGACAAGGTCAAGGTCGAGCGTATTGACCAGGCGGAC
JJ10-1          AACGCACCGGCGCATGTGCTCTTCGACAAGGTCAAGGTCGAGCGTATTGACCAGGCGGAC
JJ238-10        AACGCACCGGCGCATGTGCTCTTCGACAAGGTCAAGGTCGAGCGTATTGACCAGGCGGAC
JJ238-24        AACGCACCGGCGCATGTGCTCTTCGACAAGGTCAAGGTCGAGCGTATTGACCAGGCGGAC
JK2-10          AACGCACCGGCGCATGTGCTCTTCGACAAGGTCAAGGTCGAGCGTATTGACCAGGCGGAC
JK4-1           AACGCACCGGCGCATGTGCTCTTCGACAAGGTCAAGGTCGAGCGTATTGACCAGGCGGAC
JK48            AACGCACCGGCGCATGTGCTCTTCGACAAGGTCAAGGTCGAGCGTATTGACCAGGCGGAC
JK143-9         AACGCACCGGCGCATGTGCTCTTCGACAAGGTCAAGGTCGAGCGTATTGACCAGGCGGAC
JK143-11        AACGCACCGGCGCATGTGCTCTTCGACAAGGTCAAGGTCGAGCGTATTGACCAGGCGGAC
JM35            AACGCACCGGCGCATGTGCTCTTCGACAAGGTCAAGGTCGAGCGTATTGACCAGGCGGAC
JS581           AACGCACCGGCGCATGTGCTCTTCGACAAGGTCAAGGTCGAGCGTATTGACCAGGCGGAC
JS582           AACGCACCGGCGCATGTGCTCTTCGACAAGGTCAAGGTCGAGCGTATTGACCAGGCGGAC
JS584           AACGCACCGGCGCATGTGCTCTTCGACAAGGTCAAGGTCGAGCGTATTGACCAGGCGGAC
JW160-1         AACGCACCGGCGCATGTGCTCTTCGACAAGGTCAAGGTCGAGCGTATTGACCAGGCGGAC
LB100-1         AACGCACCGGCGCATGTGCTCTTCGACAAGGTCAAGGTCGAGCGTATTGACCAGGCGGAC
LB302           AACGCACCGGCGCATGTGCTCTTCGACAAGGTCAAGGTCGAGCGTATTGACCAGGCGGAC
LC80            AACGCACCGGCGCATGTGCTCTTCGACAAGGTCAAGGTCGAGCGTATTGACCAGGCGGAC
LD7-1           AACGCACCGGCGCATGTGCTCTTCGACAAGGTCAAGGTCGAGCGTATTGACCAGGCGGAC
LD71a           AACGCACCGGCGCATGTGCTCTTCGACAAGGTCAAGGTCGAGCGTATTGACCAGGCGGAC
LE3-1           AACGCACCGGCGCATGTGCTCTTCGACAAGGTCAAGGTCGAGCGTATTGACCAGGCGGAC
LE20-1          AACGCACCGGCGCATGTGCTCTTCGACAAGGTCAAGGTCGAGCGTATTGACCAGGCGGAC
LE116-1         AACGCACCGGCGCATGTGCTCTTCGACAAGGTCAAGGTCGAGCGTATTGACCAGGCGGAC
LG97            AACGCACCGGCGCATGTGCTCTTCGACAAGGTCAAGGTCGAGCGTATTGACCAGGCGGAC
LG102           AACGCACCGGCGCATGTGCTCTTCGACAAGGTCAAGGTCGAGCGTATTGACCAGGCGGAC
LG115           AACGCACCGGCGCATGTGCTCTTCGACAAGGTCAAGGTCGAGCGTATTGACCAGGCGGAC
LG117           AACGCACCGGCGCATGTGCTCTTCGACAAGGTCAAGGTCGAGCGTATTGACCAGGCGGAC
LH37-1          AACGCACCGGCGCATGTGCTCTTCGACAAGGTCAAGGTCGAGCGTATTGACCAGGCGGAC
LMG9322         AACGCACCGGCGCATGTGCTCTTCGACAAGGTCAAGGTCGAGCGTATTGACCAGGCGGAC
NCPPB3562       AACGCACCGGCGCATGTGCTCTTCGACAAGGTCAAGGTCGAGCGTATTGACCAGGCGGAC
NCPPB3607       AACGCACCGGCGCATGTGCTCTTCGACAAGGTCAAGGTCGAGCGTATTGACCAGGCGGAC
NCPPB3608       AACGCACCGGCGCATGTGCTCTTCGACAAGGTCAAGGTCGAGCGTATTGACCAGGCGGAC
NCPPB3610       AACGCACCGGCGCATGTGCTCTTCGACAAGGTCAAGGTCGAGCGTATTGACCAGGCGGAC
NCPPB3612       AACGCACCGGCGCATGTGCTCTTCGACAAGGTCAAGGTCGAGCGTATTGACCAGGCGGAC
NCPPB3615       AACGCACCGGCGCATGTGCTCTTCGACAAGGTCAAGGTCGAGCGTATTGACCAGGCGGAC
X2003-3218      AACGCACCGGCGCATGTGCTCTTCGACAAGGTCAAGGTCGAGCGTATTGACCAGGCGGAC
                ************************************************************

C40             CAGGGACCGGCACGCAGCTTTTCCGATTACCGGGTGGTGATCGACCACGCGCTTCCTGCC
CFBP2852        CAGGGACCGGCACGCAGCTTTTCCGATTACCGGGTGGTGATCGACCACGCGCTTCCTGCC
CFBP2911        CAGGGACCGGCACGCAGCTTTTCCGATTACCGGGTGGTGATCGACCACGCGCTTCCTGCC
FDC217          CAGGGACCGGCACGCAGCTTTTCCGATTACCGGGTGGTGATCGACCACGCGCTTCCTGCC
FDC1083         CAGGGACCGGCACGCAGCTTTTCCGATTACCGGGTGGTGATCGACCACGCGCTTCCTGCC
JF90-2          CAGGGACCGGCACGCAGCTTTTCCGATTACCGGGTGGTGATCGACCACGCGCTTCCTGCC
JF90-8          CAGGGACCGGCACGCAGCTTTTCCGATTACCGGGTGGTGATCGACCACGCGCTTCCTGCC
JJ10-1          CAGGGACCGGCACGCAGCTTTTCCGATTACCGGGTGGTGATCGACCACGCGCTTCCTGCC
JJ238-10        CAGGGACCGGCACGCAGCTTTTCCGATTACCGGGTGGTGATCGACCACGCGCTTCCTGCC
JJ238-24        CAGGGACCGGCACGCAGCTTTTCCGATTACCGGGTGGTGATCGACCACGCGCTTCCTGCC
JK2-10          CAGGGACCGGCACGCAGCTTTTCCGATTACCGGGTGGTGATCGACCACGCGCTTCCTGCC
JK4-1           CAGGGACCGGCACGCAGCTTTTCCGATTACCGGGTGGTGATCGACCACGCGCTTCCTGCC
JK48            CAGGGACCGGCACGCAGCTTTTCCGATTACCGGGTGGTGATCGACCACGCGCTTCCTGCC
JK143-9         CAGGGACCGGCACGCAGCTTTTCCGATTACCGGGTGGTGATCGACCACGCGCTTCCTGCC
JK143-11        CAGGGACCGGCACGCAGCTTTTCCGATTACCGGGTGGTGATCGACCACGCGCTTCCTGCC
JM35            CAGGGACCGGCACGCAGCTTTTCCGATTACCGGGTGGTGATCGACCACGCGCTTCCTGCC
JS581           CAGGGACCGGCACGCAGCTTTTCCGATTACCGGGTGGTGATCGACCACGCGCTTCCTGCC
JS582           CAGGGACCGGCACGCAGCTTTTCCGATTACCGGGTGGTGATCGACCACGCGCTTCCTGCC
JS584           CAGGGACCGGCACGCAGCTTTTCCGATTACCGGGTGGTGATCGACCACGCGCTTCCTGCC
JW160-1         CAGGGACCGGCACGCAGCTTTTCCGATTACCGGGTGGTGATCGACCACGCGCTTCCTGCC
LB100-1         CAGGGACCGGCACGCAGCTTTTCCGATTACCGGGTGGTGATCGACCACGCGCTTCCTGCC
LB302           CAGGGACCGGCACGCAGCTTTTCCGATTACCGGGTGGTGATCGACCACGCGCTTCCTGCC
LC80            CAGGGACCGGCACGCAGCTTTTCCGATTACCGGGTGGTGATCGACCACGCGCTTCCTGCC
LD7-1           CAGGGACCGGCACGCAGCTTTTCCGATTACCGGGTGGTGATCGACCACGCGCTTCCTGCC
LD71a           CAGGGACCGGCACGCAGCTTTTCCGATTACCGGGTGGTGATCGACCACGCGCTTCCTGCC
LE3-1           CAGGGACCGGCACGCAGCTTTTCCGATTACCGGGTGGTGATCGACCACGCGCTTCCTGCC
LE20-1          CAGGGACCGGCACGCAGCTTTTCCGATTACCGGGTGGTGATCGACCACGCGCTTCCTGCC
LE116-1         CAGGGACCGGCACGCAGCTTTTCCGATTACCGGGTGGTGATCGACCACGCGCTTCCTGCC
LG97            CAGGGACCGGCACGCAGCTTTTCCGATTACCGGGTGGTGATCGACCACGCGCTTCCTGCC
LG102           CAGGGACCGGCACGCAGCTTTTCCGATTACCGGGTGGTGATCGACCACGCGCTTCCTGCC
LG115           CAGGGACCGGCACGCAGCTTTTCCGATTACCGGGTGGTGATCGACCACGCGCTTCCTGCC
LG117           CAGGGACCGGCACGCAGCTTTTCCGATTACCGGGTGGTGATCGACCACGCGCTTCCTGCC
LH37-1          CAGGGACCGGCACGCAGCTTTTCCGATTACCGGGTGGTGATCGACCACGCGCTTCCTGCC
LMG9322         CAGGGACCGGCACGCAGCTTTTCCGATTACCGGGTGGTGATCGACCACGCGCTTCCTGCC
NCPPB3562       CAGGGACCGGCACGCAGCTTTTCCGATTACCGGGTGGTGATCGACCACGCGCTTCCTGCC
NCPPB3607       CAGGGACCGGCACGCAGCTTTTCCGATTACCGGGTGGTGATCGACCACGCGCTTCCTGCC
NCPPB3608       CAGGGACCGGCACGCAGCTTTTCCGATTACCGGGTGGTGATCGACCACGCGCTTCCTGCC
NCPPB3610       CAGGGACCGGCACGCAGCTTTTCCGATTACCGGGTGGTGATCGACCACGCGCTTCCTGCC
NCPPB3612       CAGGGACCGGCACGCAGCTTTTCCGATTACCGGGTGGTGATCGACCACGCGCTTCCTGCC
NCPPB3615       CAGGGACCGGCACGCAGCTTTTCCGATTACCGGGTGGTGATCGACCACGCGCTTCCTGCC
X2003-3218      CAGGGACCGGCACGCAGCTTTTCCGATTACCGGGTGGTGATCGACCACGCGCTTCCTGCC
                ************************************************************
```

```
C40             GGCGTAAGCGTGACCGAACTGTTCTGA
CFBP2852        GGCGTAAGCGTGACCGAACTGTTCTGA
CFBP2911        GGCGTAAGCGTGACCGAACTGTTCTGA
FDC217          GGCGTAAGCGTGACCGAACTGTTCTGA
FDC1083         GGCGTAAGCGTGACCGAACTGTTCTGA
JF90-2          GGCGTAAGCGTGACCGAACTGTTCTGA
JF90-8          GGCGTAAGCGTGACCGAACTGTTCTGA
JJ10-1          GGCGTAAGCGTGACCGAACTGTTCTGA
JJ238-10        GGCGTAAGCGTGACCGAACTGTTCTGA
JJ238-24        GGCGTAAGCGTGACCGAACTGTTCTGA
JK2-10          GGCGTAAGCGTGACCGAACTGTTCTGA
JK4-1           GGCGTAAGCGTGACCGAACTGTTCTGA
JK48            GGCGTAAGCGTGACCGAACTGTTCTGA
JK143-9         GGCGTAAGCGTGACCGAACTGTTCTGA
JK143-11        GGCGTAAGCGTGACCGAACTGTTCTGA
JM35            GGCGTAAGCGTGACCGAACTGTTCTGA
JS581           GGCGTAAGCGTGACCGAACTGTTCTGA
JS582           GGCGTAAGCGTGACCGAACTGTTCTGA
JS584           GGCGTAAGCGTGACCGAACTGTTCTGA
JW160-1         GGCGTAAGCGTGACCGAACTGTTCTGA
LB100-1         GGCGTAAGCGTGACCGAACTGTTCTGA
LB302           GGCGTAAGCGTGACCGAACTGTTCTGA
LC80            GGCGTAAGCGTGACCGAACTGTTCTGA
LD7-1           GGCGTAAGCGTGACCGAACTGTTCTGA
LD71a           GGCGTAAGCGTGACCGAACTGTTCTGA
LE3-1           GGCGTAAGCGTGACCGAACTGTTCTGA
LE20-1          GGCGTAAGCGTGACCGAACTGTTCTGA
LE116-1         GGCGTAAGCGTGACCGAACTGTTCTGA
LG97            GGCGTAAGCGTGACCGAACTGTTCTGA
LG102           GGCGTAAGCGTGACCGAACTGTTCTGA
LG115           GGCGTAAGCGTGACCGAACTGTTCTGA
LG117           GGCGTAAGCGTGACCGAACTGTTCTGA
LH37-1          GGCGTAAGCGTGACCGAACTGTTCTGA
LMG9322         GGCGTAAGCGTGACCGAACTGTTCTGA
NCPPB3562       GGCGTAAGCGTGACCGAACTGTTCTGA
NCPPB3607       GGCGTAAGCGTGACCGAACTGTTCTGA
NCPPB3608       GGCGTAAGCGTGACCGAACTGTTCTGA
NCPPB3610       GGCGTAAGCGTGACCGAACTGTTCTGA
NCPPB3612       GGCGTAAGCGTGACCGAACTGTTCTGA
NCPPB3615       GGCGTAAGCGTGACCGAACTGTTCTGA
X2003-3218      GGCGTAAGCGTGACCGAACTGTTCTGA
                ***************************
```

Cas4 (acquisition)

```
C40             ATGGACGACGACGACCTGATCCCGCTCTCTGCCCTACAGCACTACCTATACTGCCCGCGC
CFBP2852        ATGGACGACGACGACCTGATCCCGCTCTCTGCCCTACAGCACTACCTATACTGCCCGCGC
FDC217          ATGGACGACGACGACCTGATCCCGCTCTCTGCCCTACAGCACTACCTATACTGCCCGCGC
FDC1083         ATGGACGACGACGACCTGATCCCGCTCTCTGCCCTACAGCACTACCTATACTGCCCGCGC
JJ10-1          ATGGACGACGACGACCTGATCCCGCTCTCTGCCCTACAGCACTACCTATACTGCCCGCGC
JJ238-10        ATGGACGACGACGACCTGATCCCGCTCTCTGCCCTACAGCACTACCTATACTGCCCGCGC
JK4-1           ATGGACGACGACGACCTGATCCCGCTCTCTGCCCTACAGCACTACCTATACTGCCCGCGC
JW160-1         ATGGACGACGACGACCTGATCCCGCTCTCTGCCCTACAGCACTACCTATACTGCCCGCGC
LB100-1         ATGGACGACGACGACCTGATCCCGCTCTCTGCCCTACAGCACTACCTATACTGCCCGCGC
LC80            ATGGACGACGACGACCTGATCCCGCTCTCTGCCCTACAGCACTACCTATACTGCCCGCGC
LD7-1           ATGGACGACGACGACCTGATCCCGCTCTCTGCCCTACAGCACTACCTATACTGCCCGCGC
LE116-1         ATGGACGACGACGACCTGATCCCGCTCTCTGCCCTACAGCACTACCTATACTGCCCGCGC
LG117           ATGGACGACGACGACCTGATCCCGCTCTCTGCCCTACAGCACTACCTATACTGCCCGCGC
LH37-1          ATGGACGACGACGACCTGATCCCGCTCTCTGCCCTACAGCACTACCTATACTGCCCGCGC
LMG9322         ATGGACGACGACGACCTGATCCCGCTCTCTGCCCTACAGCACTACCTATACTGCCCGCGC
NCPPB3562       ATGGACGACGACGACCTGATCCCGCTCTCTGCCCTACAGCACTACCTATACTGCCCGCGC
NCPPB3610       ATGGACGACGACGACCTGATCCCGCTCTCTGCCCTACAGCACTACCTATACTGCCCGCGC
NCPPB3612       ATGGACGACGACGACCTGATCCCGCTCTCTGCCCTACAGCACTACCTATACTGCCCGCGC
CFBP2911        ATGGACGACGACGACCTGATCCCGCTCTCTGCCCTACAGCACTACCTATACTGCCCGCGC
JF90-2          ATGGACGACGACGACCTGATCCCGCTCTCTGCCCTACAGCACTACCTATACTGCCCGCGC
JF90-8          ATGGACGACGACGACCTGATCCCGCTCTCTGCCCTACAGCACTACCTATACTGCCCGCGC
JJ238-24        ATGGACGACGACGACCTGATCCCGCTCTCTGCCCTACAGCACTACCTATACTGCCCGCGC
JK2-10          ATGGACGACGACGACCTGATCCCGCTCTCTGCCCTACAGCACTACCTATACTGCCCGCGC
JK48            ATGGACGACGACGACCTGATCCCGCTCTCTGCCCTACAGCACTACCTATACTGCCCGCGC
JK143-9         ATGGACGACGACGACCTGATCCCGCTCTCTGCCCTACAGCACTACCTATACTGCCCGCGC
JK143-11        ATGGACGACGACGACCTGATCCCGCTCTCTGCCCTACAGCACTACCTATACTGCCCGCGC
JM35            ATGGACGACGACGACCTGATCCCGCTCTCTGCCCTACAGCACTACCTATACTGCCCGCGC
JS581           ATGGACGACGACGACCTGATCCCGCTCTCTGCCCTACAGCACTACCTATACTGCCCGCGC
JS582           ATGGACGACGACGACCTGATCCCGCTCTCTGCCCTACAGCACTACCTATACTGCCCGCGC
JS584           ATGGACGACGACGACCTGATCCCGCTCTCTGCCCTACAGCACTACCTATACTGCCCGCGC
LB302           ATGGACGACGACGACCTGATCCCGCTCTCTGCCCTACAGCACTACCTATACTGCCCGCGC
LD71a           ATGGACGACGACGACCTGATCCCGCTCTCTGCCCTACAGCACTACCTATACTGCCCGCGC
LE3-1           ATGGACGACGACGACCTGATCCCGCTCTCTGCCCTACAGCACTACCTATACTGCCCGCGC
LE20-1          ATGGACGACGACGACCTGATCCCGCTCTCTGCCCTACAGCACTACCTATACTGCCCGCGC
LG97            ATGGACGACGACGACCTGATCCCGCTCTCTGCCCTACAGCACTACCTATACTGCCCGCGC
LG102           ATGGACGACGACGACCTGATCCCGCTCTCTGCCCTACAGCACTACCTATACTGCCCGCGC
LG115           ATGGACGACGACGACCTGATCCCGCTCTCTGCCCTACAGCACTACCTATACTGCCCGCGC
NCPPB3607       ATGGACGACGACGACCTGATCCCGCTCTCTGCCCTACAGCACTACCTATACTGCCCGCGC
NCPPB3608       ATGGACGACGACGACCTGATCCCGCTCTCTGCCCTACAGCACTACCTATACTGCCCGCGC
NCPPB3615       ATGGACGACGACGACCTGATCCCGCTCTCTGCCCTACAGCACTACCTATACTGCCCGCGC
X2003-3218      ATGGACGACGACGACCTGATCCCGCTCTCTGCCCTACAGCACTACCTATACTGCCCGCGC
                ************************************************************

C40             CAATGCGCGCTGATCCATGTCGAACGGCAATGGGCAGAGAACAGGCACACCGCCGAAGGC
CFBP2852        CAATGCGCGCTGATCCATGTCGAACGGCAATGGGCAGAGAACAGGCACACCGCCGAAGGC
FDC217          CAATGCGCGCTGATCCATGTCGAACGGCAATGGGCAGAGAACAGGCACACCGCCGAAGGC
FDC1083         CAATGCGCGCTGATCCATGTCGAACGGCAATGGGCAGAGAACAGGCACACCGCCGAAGGC
JJ10-1          CAATGCGCGCTGATCCATGTCGAACGGCAATGGGCAGAGAACAGGCACACCGCCGAAGGC
JJ238-10        CAATGCGCGCTGATCCATGTCGAACGGCAATGGGCAGAGAACAGGCACACCGCCGAAGGC
JK4-1           CAATGCGCGCTGATCCATGTCGAACGGCAATGGGCAGAGAACAGGCACACCGCCGAAGGC
JW160-1         CAATGCGCGCTGATCCATGTCGAACGGCAATGGGCAGAGAACAGGCACACCGCCGAAGGC
LB100-1         CAATGCGCGCTGATCCATGTCGAACGGCAATGGGCAGAGAACAGGCACACCGCCGAAGGC
LC80            CAATGCGCGCTGATCCATGTCGAACGGCAATGGGCAGAGAACAGGCACACCGCCGAAGGC
LD7-1           CAATGCGCGCTGATCCATGTCGAACGGCAATGGGCAGAGAACAGGCACACCGCCGAAGGC
LE116-1         CAATGCGCGCTGATCCATGTCGAACGGCAATGGGCAGAGAACAGGCACACCGCCGAAGGC
LG117           CAATGCGCGCTGATCCATGTCGAACGGCAATGGGCAGAGAACAGGCACACCGCCGAAGGC
LH37-1          CAATGCGCGCTGATCCATGTCGAACGGCAATGGGCAGAGAACAGGCACACCGCCGAAGGC
LMG9322         CAATGCGCGCTGATCCATGTCGAACGGCAATGGGCAGAGAACAGGCACACCGCCGAAGGC
NCPPB3562       CAATGCGCGCTGATCCATGTCGAACGGCAATGGGCAGAGAACAGGCACACCGCCGAAGGC
NCPPB3610       CAATGCGCGCTGATCCATGTCGAACGGCAATGGGCAGAGAACAGGCACACCGCCGAAGGC
NCPPB3612       CAATGCGCGCTGATCCATGTCGAACGGCAATGGGCAGAGAACAGGCACACCGCCGAAGGC
CFBP2911        CAATGCGCGCTGATCCATGTCGAACGGCAATGGGCAGAGAACAGGCACACCGCCGAAGGC
JF90-2          CAATGCGCGCTGATCCATGTCGAACGGCAATGGGCAGAGAACAGGCACACCGCCGAAGGC
JF90-8          CAATGCGCGCTGATCCATGTCGAACGGCAATGGGCAGAGAACAGGCACACCGCCGAAGGC
JJ238-24        CAATGCGCGCTGATCCATGTCGAACGGCAATGGGCAGAGAACAGGCACACCGCCGAAGGC
JK2-10          CAATGCGCGCTGATCCATGTCGAACGGCAATGGGCAGAGAACAGGCACACCGCCGAAGGC
JK48            CAATGCGCGCTGATCCATGTCGAACGGCAATGGGCAGAGAACAGGCACACCGCCGAAGGC
JK143-9         CAATGCGCGCTGATCCATGTCGAACGGCAATGGGCAGAGAACAGGCACACCGCCGAAGGC
JK143-11        CAATGCGCGCTGATCCATGTCGAACGGCAATGGGCAGAGAACAGGCACACCGCCGAAGGC
JM35            CAATGCGCGCTGATCCATGTCGAACGGCAATGGGCAGAGAACAGGCACACCGCCGAAGGC
JS581           CAATGCGCGCTGATCCATGTCGAACGGCAATGGGCAGAGAACAGGCACACCGCCGAAGGC
JS582           CAATGCGCGCTGATCCATGTCGAACGGCAATGGGCAGAGAACAGGCACACCGCCGAAGGC
JS584           CAATGCGCGCTGATCCATGTCGAACGGCAATGGGCAGAGAACAGGCACACCGCCGAAGGC
LB302           CAATGCGCGCTGATCCATGTCGAACGGCAATGGGCAGAGAACAGGCACACCGCCGAAGGC
LD71a           CAATGCGCGCTGATCCATGTCGAACGGCAATGGGCAGAGAACAGGCACACCGCCGAAGGC
LE3-1           CAATGCGCGCTGATCCATGTCGAACGGCAATGGGCAGAGAACAGGCACACCGCCGAAGGC
LE20-1          CAATGCGCGCTGATCCATGTCGAACGGCAATGGGCAGAGAACAGGCACACCGCCGAAGGC
LG97            CAATGCGCGCTGATCCATGTCGAACGGCAATGGGCAGAGAACAGGCACACCGCCGAAGGC
LG102           CAATGCGCGCTGATCCATGTCGAACGGCAATGGGCAGAGAACAGGCACACCGCCGAAGGC
LG115           CAATGCGCGCTGATCCATGTCGAACGGCAATGGGCAGAGAACAGGCACACCGCCGAAGGC
NCPPB3607       CAATGCGCGCTGATCCATGTCGAACGGCAATGGGCAGAGAACAGGCACACCGCCGAAGGC
NCPPB3608       CAATGCGCGCTGATCCATGTCGAACGGCAATGGGCAGAGAACAGGCACACCGCCGAAGGC
NCPPB3615       CAATGCGCGCTGATCCATGTCGAACGGCAATGGGCAGAGAACAGGCACACCGCCGAAGGC
X2003-3218      CAATGCGCGCTGATCCATGTCGAACGGCAATGGGCAGAGAACAGGCACACCGCCGAAGGC
                ************************************************************
```

```
C40             CGGCTGCTGCACGAACGTGCCGATGTGGCACACACGCAGCGCAGGCGTGGCGTGCGCACC
CFBP2852        CGGCTGCTGCACGAACGTGCCGATGTGGCACACACGCAGCGCAGGCGTGGCGTGCGCACC
FDC217          CGGCTGCTGCACGAACGTGCCGATGTGGCACACACGCAGCGCAGGCGTGGCGTGCGCACC
FDC1083         CGGCTGCTGCACGAACGTGCCGATGTGGCACACACGCAGCGCAGGCGTGGCGTGCGCACC
JJ10-1          CGGCTGCTGCACGAACGTGCCGATGTGGCACACACGCAGCGCAGGCGTGGCGTGCGCACC
JJ238-10        CGGCTGCTGCACGAACGTGCCGATGTGGCACACACGCAGCGCAGGCGTGGCGTGCGCACC
JK4-1           CGGCTGCTGCACGAACGTGCCGATGTGGCACACACGCAGCGCAGGCGTGGCGTGCGCACC
JW160-1         CGGCTGCTGCACGAACGTGCCGATGTGGCACACACGCAGCGCAGGCGTGGCGTGCGCACC
LB100-1         CGGCTGCTGCACGAACGTGCCGATGTGGCACACACGCAGCGCAGGCGTGGCGTGCGCACC
LC80            CGGCTGCTGCACGAACGTGCCGATGTGGCACACACGCAGCGCAGGCGTGGCGTGCGCACC
LD7-1           CGGCTGCTGCACGAACGTGCCGATGTGGCACACACGCAGCGCAGGCGTGGCGTGCGCACC
LE116-1         CGGCTGCTGCACGAACGTGCCGATGTGGCACACACGCAGCGCAGGCGTGGCGTGCGCACC
LG117           CGGCTGCTGCACGAACGTGCCGATGTGGCACACACGCAGCGCAGGCGTGGCGTGCGCACC
LH37-1          CGGCTGCTGCACGAACGTGCCGATGTGGCACACACGCAGCGCAGGCGTGGCGTGCGCACC
LMG9322         CGGCTGCTGCACGAACGTGCCGATGTGGCACACACGCAGCGCAGGCGTGGCGTGCGCACC
NCPPB3562       CGGCTGCTGCACGAACGTGCCGATGTGGCACACACGCAGCGCAGGCGTGGCGTGCGCACC
NCPPB3610       CGGCTGCTGCACGAACGTGCCGATGTGGCACACACGCAGCGCAGGCGTGGCGTGCGCACC
NCPPB3612       CGGCTGCTGCACGAACGTGCCGATGTGGCACACACGCAGCGCAGGCGTGGCGTGCGCACC
CFBP2911        CGGCTGCTGCACGAACGTGCCGATGTGGCACACACGCAGCGCAGGCGTGGCGTGCGCACC
JF90-2          CGGCTGCTGCACGAACGTGCCGATGTGGCACACACGCAGCGCAGGCGTGGCGTGCGCACC
JF90-8          CGGCTGCTGCACGAACGTGCCGATGTGGCACACACGCAGCGCAGGCGTGGCGTGCGCACC
JJ238-24        CGGCTGCTGCACGAACGTGCCGATGTGGCACACACGCAGCGCAGGCGTGGCGTGCGCACC
JK2-10          CGGCTGCTGCACGAACGTGCCGATGTGGCACACACGCAGCGCAGGCGTGGCGTGCGCACC
JK48            CGGCTGCTGCACGAACGTGCCGATGTGGCACACACGCAGCGCAGGCGTGGCGTGCGCACC
JK143-9         CGGCTGCTGCACGAACGTGCCGATGTGGCACACACGCAGCGCAGGCGTGGCGTGCGCACC
JK143-11        CGGCTGCTGCACGAACGTGCCGATGTGGCACACACGCAGCGCAGGCGTGGCGTGCGCACC
JM35            CGGCTGCTGCACGAACGTGCCGATGTGGCACACACGCAGCGCAGGCGTGGCGTGCGCACC
JS581           CGGCTGCTGCACGAACGTGCCGATGTGGCACACACGCAGCGCAGGCGTGGCGTGCGCACC
JS582           CGGCTGCTGCACGAACGTGCCGATGTGGCACACACGCAGCGCAGGCGTGGCGTGCGCACC
JS584           CGGCTGCTGCACGAACGTGCCGATGTGGCACACACGCAGCGCAGGCGTGGCGTGCGCACC
LB302           CGGCTGCTGCACGAACGTGCCGATGTGGCACACACGCAGCGCAGGCGTGGCGTGCGCACC
LD71a           CGGCTGCTGCACGAACGTGCCGATGTGGCACACACGCAGCGCAGGCGTGGCGTGCGCACC
LE3-1           CGGCTGCTGCACGAACGTGCCGATGTGGCACACACGCAGCGCAGGCGTGGCGTGCGCACC
LE20-1          CGGCTGCTGCACGAACGTGCCGATGTGGCACACACGCAGCGCAGGCGTGGCGTGCGCACC
LG97            CGGCTGCTGCACGAACGTGCCGATGTGGCACACACGCAGCGCAGGCGTGGCGTGCGCACC
LG102           CGGCTGCTGCACGAACGTGCCGATGTGGCACACACGCAGCGCAGGCGTGGCGTGCGCACC
LG115           CGGCTGCTGCACGAACGTGCCGATGTGGCACACACGCAGCGCAGGCGTGGCGTGCGCACC
NCPPB3607       CGGCTGCTGCACGAACGTGCCGATGTGGCACACACGCAGCGCAGGCGTGGCGTGCGCACC
NCPPB3608       CGGCTGCTGCACGAACGTGCCGATGTGGCACACACGCAGCGCAGGCGTGGCGTGCGCACC
NCPPB3615       CGGCTGCTGCACGAACGTGCCGATGTGGCACACACGCAGCGCAGGCGTGGCGTGCGCACC
X2003-3218      CGGCTGCTGCACGAACGTGCCGATGTGGCACACACGCAGCGCAGGCGTGGCGTGCGCACC
                ************************************************************

C40             GTCACCGCGATGCCGCTGCTGGCGCTCGAACTGGGCATCACGGGCAAGGCCGATGTGGTC
CFBP2852        GTCACCGCGATGCCGCTGCTGGCGCTCGAACTGGGCATCACGGGCAAGGCCGATGTGGTC
FDC217          GTCACCGCGATGCCGCTGCTGGCGCTCGAACTGGGCATCACGGGCAAGGCCGATGTGGTC
FDC1083         GTCACCGCGATGCCGCTGCTGGCGCTCGAACTGGGCATCACGGGCAAGGCCGATGTGGTC
JJ10-1          GTCACCGCGATGCCGCTGCTGGCGCTCGAACTGGGCATCACGGGCAAGGCCGATGTGGTC
JJ238-10        GTCACCGCGATGCCGCTGCTGGCGCTCGAACTGGGCATCACGGGCAAGGCCGATGTGGTC
JK4-1           GTCACCGCGATGCCGCTGCTGGCGCTCGAACTGGGCATCACGGGCAAGGCCGATGTGGTC
JW160-1         GTCACCGCGATGCCGCTGCTGGCGCTCGAACTGGGCATCACGGGCAAGGCCGATGTGGTC
LB100-1         GTCACCGCGATGCCGCTGCTGGCGCTCGAACTGGGCATCACGGGCAAGGCCGATGTGGTC
LC80            GTCACCGCGATGCCGCTGCTGGCGCTCGAACTGGGCATCACGGGCAAGGCCGATGTGGTC
LD7-1           GTCACCGCGATGCCGCTGCTGGCGCTCGAACTGGGCATCACGGGCAAGGCCGATGTGGTC
LE116-1         GTCACCGCGATGCCGCTGCTGGCGCTCGAACTGGGCATCACGGGCAAGGCCGATGTGGTC
LG117           GTCACCGCGATGCCGCTGCTGGCGCTCGAACTGGGCATCACGGGCAAGGCCGATGTGGTC
LH37-1          GTCACCGCGATGCCGCTGCTGGCGCTCGAACTGGGCATCACGGGCAAGGCCGATGTGGTC
LMG9322         GTCACCGCGATGCCGCTGCTGGCGCTCGAACTGGGCATCACGGGCAAGGCCGATGTGGTC
NCPPB3562       GTCACCGCGATGCCGCTGCTGGCGCTCGAACTGGGCATCACGGGCAAGGCCGATGTGGTC
NCPPB3610       GTCACCGCGATGCCGCTGCTGGCGCTCGAACTGGGCATCACGGGCAAGGCCGATGTGGTC
NCPPB3612       GTCACCGCGATGCCGCTGCTGGCGCTCGAACTGGGCATCACGGGCAAGGCCGATGTGGTC
CFBP2911        GTCACCGCGATGCCGCTGCTGGCGCTCGAACTGGGCATCACGGGCAAGGCCGATGTGGTC
JF90-2          GTCACCGCGATGCCGCTGCTGGCGCTCGAACTGGGCATCACGGGCAAGGCCGATGTGGTC
JF90-8          GTCACCGCGATGCCGCTGCTGGCGCTCGAACTGGGCATCACGGGCAAGGCCGATGTGGTC
JJ238-24        GTCACCGCGATGCCGCTGCTGGCGCTCGAACTGGGCATCACGGGCAAGGCCGATGTGGTC
JK2-10          GTCACCGCGATGCCGCTGCTGGCGCTCGAACTGGGCATCACGGGCAAGGCCGATGTGGTC
JK48            GTCACCGCGATGCCGCTGCTGGCGCTCGAACTGGGCATCACGGGCAAGGCCGATGTGGTC
JK143-9         GTCACCGCGATGCCGCTGCTGGCGCTCGAACTGGGCATCACGGGCAAGGCCGATGTGGTC
JK143-11        GTCACCGCGATGCCGCTGCTGGCGCTCGAACTGGGCATCACGGGCAAGGCCGATGTGGTC
JM35            GTCACCGCGATGCCGCTGCTGGCGCTCGAACTGGGCATCACGGGCAAGGCCGATGTGGTC
JS581           GTCACCGCGATGCCGCTGCTGGCGCTCGAACTGGGCATCACGGGCAAGGCCGATGTGGTC
JS582           GTCACCGCGATGCCGCTGCTGGCGCTCGAACTGGGCATCACGGGCAAGGCCGATGTGGTC
JS584           GTCACCGCGATGCCGCTGCTGGCGCTCGAACTGGGCATCACGGGCAAGGCCGATGTGGTC
LB302           GTCACCGCGATGCCGCTGCTGGCGCTCGAACTGGGCATCACGGGCAAGGCCGATGTGGTC
LD71a           GTCACCGCGATGCCGCTGCTGGCGCTCGAACTGGGCATCACGGGCAAGGCCGATGTGGTC
LE3-1           GTCACCGCGATGCCGCTGCTGGCGCTCGAACTGGGCATCACGGGCAAGGCCGATGTGGTC
LE20-1          GTCACCGCGATGCCGCTGCTGGCGCTCGAACTGGGCATCACGGGCAAGGCCGATGTGGTC
LG97            GTCACCGCGATGCCGCTGCTGGCGCTCGAACTGGGCATCACGGGCAAGGCCGATGTGGTC
LG102           GTCACCGCGATGCCGCTGCTGGCGCTCGAACTGGGCATCACGGGCAAGGCCGATGTGGTC
LG115           GTCACCGCGATGCCGCTGCTGGCGCTCGAACTGGGCATCACGGGCAAGGCCGATGTGGTC
NCPPB3607       GTCACCGCGATGCCGCTGCTGGCGCTCGAACTGGGCATCACGGGCAAGGCCGATGTGGTC
NCPPB3608       GTCACCGCGATGCCGCTGCTGGCGCTCGAACTGGGCATCACGGGCAAGGCCGATGTGGTC
NCPPB3615       GTCACCGCGATGCCGCTGCTGGCGCTCGAACTGGGCATCACGGGCAAGGCCGATGTGGTC
X2003-3218      GTCACCGCGATGCCGCTGCTGGCGCTCGAACTGGGCATCACGGGCAAGGCCGATGTGGTC
                ************************************************************
```

```
C40             GAGTTCCATCACGATCCCGCAGGGGAATTCGCGTTCCCCGTGGAGTACAAACGGGGCCGA
CFBP2852        GAGTTCCATCACGATCCCGCAGGGGAATTCGCGTTCCCCGTGGAGTACAAACGGGGCCGA
FDC217          GAGTTCCATCACGATCCCGCAGGGGAATTCGCGTTCCCCGTGGAGTACAAACGGGGCCGA
FDC1083         GAGTTCCATCACGATCCCGCAGGGGAATTCGCGTTCCCCGTGGAGTACAAACGGGGCCGA
JJ10-1          GAGTTCCATCACGATCCCGCAGGGGAATTCGCGTTCCCCGTGGAGTACAAACGGGGCCGA
JJ238-10        GAGTTCCATCACGATCCCGCAGGGGAATTCGCGTTCCCCGTGGAGTACAAACGGGGCCGA
JK4-1           GAGTTCCATCACGATCCCGCAGGGGAATTCGCGTTCCCCGTGGAGTACAAACGGGGCCGA
JW160-1         GAGTTCCATCACGATCCCGCAGGGGAATTCGCGTTCCCCGTGGAGTACAAACGGGGCCGA
LB100-1         GAGTTCCATCACGATCCCGCAGGGGAATTCGCGTTCCCCGTGGAGTACAAACGGGGCCGA
LC80            GAGTTCCATCACGATCCCGCAGGGGAATTCGCGTTCCCCGTGGAGTACAAACGGGGCCGA
LD7-1           GAGTTCCATCACGATCCCGCAGGGGAATTCGCGTTCCCCGTGGAGTACAAACGGGGCCGA
LE116-1         GAGTTCCATCACGATCCCGCAGGGGAATTCGCGTTCCCCGTGGAGTACAAACGGGGCCGA
LG117           GAGTTCCATCACGATCCCGCAGGGGAATTCGCGTTCCCCGTGGAGTACAAACGGGGCCGA
LH37-1          GAGTTCCATCACGATCCCGCAGGGGAATTCGCGTTCCCCGTGGAGTACAAACGGGGCCGA
LMG9322         GAGTTCCATCACGATCCCGCAGGGGAATTCGCGTTCCCCGTGGAGTACAAACGGGGCCGA
NCPPB3562       GAGTTCCATCACGATCCCGCAGGGGAATTCGCGTTCCCCGTGGAGTACAAACGGGGCCGA
NCPPB3610       GAGTTCCATCACGATCCCGCAGGGGAATTCGCGTTCCCCGTGGAGTACAAACGGGGCCGA
NCPPB3612       GAGTTCCATCACGATCCCGCAGGGGAATTCGCGTTCCCCGTGGAGTACAAACGGGGCCGA
CFBP2911        GAGTTCCATCACGATCCCGCAGGGGAATTCGCGTTCCCCGTGGAGTACAAACGGGGCCGA
JF90-2          GAGTTCCATCACGATCCCGCAGGGGAATTCGCGTTCCCCGTGGAGTACAAACGGGGCCGA
JF90-8          GAGTTCCATCACGATCCCGCAGGGGAATTCGCGTTCCCCGTGGAGTACAAACGGGGCCGA
JJ238-24        GAGTTCCATCACGATCCCGCAGGGGAATTCGCGTTCCCCGTGGAGTACAAACGGGGCCGA
JK2-10          GAGTTCCATCACGATCCCGCAGGGGAATTCGCGTTCCCCGTGGAGTACAAACGGGGCCGA
JK48            GAGTTCCATCACGATCCCGCAGGGGAATTCGCGTTCCCCGTGGAGTACAAACGGGGCCGA
JK143-9         GAGTTCCATCACGATCCCGCAGGGGAATTCGCGTTCCCCGTGGAGTACAAACGGGGCCGA
JK143-11        GAGTTCCATCACGATCCCGCAGGGGAATTCGCGTTCCCCGTGGAGTACAAACGGGGCCGA
JM35            GAGTTCCATCACGATCCCGCAGGGGAATTCGCGTTCCCCGTGGAGTACAAACGGGGCCGA
JS581           GAGTTCCATCACGATCCCGCAGGGGAATTCGCGTTCCCCGTGGAGTACAAACGGGGCCGA
JS582           GAGTTCCATCACGATCCCGCAGGGGAATTCGCGTTCCCCGTGGAGTACAAACGGGGCCGA
JS584           GAGTTCCATCACGATCCCGCAGGGGAATTCGCGTTCCCCGTGGAGTACAAACGGGGCCGA
LB302           GAGTTCCATCACGATCCCGCAGGGGAATTCGCGTTCCCCGTGGAGTACAAACGGGGCCGA
LD71a           GAGTTCCATCACGATCCCGCAGGGGAATTCGCGTTCCCCGTGGAGTACAAACGGGGCCGA
LE3-1           GAGTTCCATCACGATCCCGCAGGGGAATTCGCGTTCCCCGTGGAGTACAAACGGGGCCGA
LE20-1          GAGTTCCATCACGATCCCGCAGGGGAATTCGCGTTCCCCGTGGAGTACAAACGGGGCCGA
LG97            GAGTTCCATCACGATCCCGCAGGGGAATTCGCGTTCCCCGTGGAGTACAAACGGGGCCGA
LG102           GAGTTCCATCACGATCCCGCAGGGGAATTCGCGTTCCCCGTGGAGTACAAACGGGGCCGA
LG115           GAGTTCCATCACGATCCCGCAGGGGAATTCGCGTTCCCCGTGGAGTACAAACGGGGCCGA
NCPPB3607       GAGTTCCATCACGATCCCGCAGGGGAATTCGCGTTCCCCGTGGAGTACAAACGGGGCCGA
NCPPB3608       GAGTTCCATCACGATCCCGCAGGGGAATTCGCGTTCCCCGTGGAGTACAAACGGGGCCGA
NCPPB3615       GAGTTCCATCACGATCCCGCAGGGGAATTCGCGTTCCCCGTGGAGTACAAACGGGGCCGA
X2003-3218      GAGTTCCATCACGATCCCGCAGGGGAATTCGCGTTCCCCGTGGAGTACAAACGGGGCCGA
                ************************************************************

C40             CCCAAGTCGCACCGGGCCGATGAGGTACAGCTGTGTGCCCAGGCACTGTGTCTGGAGAGC
CFBP2852        CCCAAGTCGCACCGGGCCGATGAGGTACAGCTGTGTGCCCAGGCACTGTGTCTGGAGAGC
FDC217          CCCAAGTCGCACCGGGCCGATGAGGTACAGCTGTGTGCCCAGGCACTGTGTCTGGAGAGC
FDC1083         CCCAAGTCGCACCGGGCCGATGAGGTACAGCTGTGTGCCCAGGCACTGTGTCTGGAGAGC
JJ10-1          CCCAAGTCGCACCGGGCCGATGAGGTACAGCTGTGTGCCCAGGCACTGTGTCTGGAGAGC
JJ238-10        CCCAAGTCGCACCGGGCCGATGAGGTACAGCTGTGTGCCCAGGCACTGTGTCTGGAGAGC
JK4-1           CCCAAGTCGCACCGGGCCGATGAGGTACAGCTGTGTGCCCAGGCACTGTGTCTGGAGAGC
JW160-1         CCCAAGTCGCACCGGGCCGATGAGGTACAGCTGTGTGCCCAGGCACTGTGTCTGGAGAGC
LB100-1         CCCAAGTCGCACCGGGCCGATGAGGTACAGCTGTGTGCCCAGGCACTGTGTCTGGAGAGC
LC80            CCCAAGTCGCACCGGGCCGATGAGGTACAGCTGTGTGCCCAGGCACTGTGTCTGGAGAGC
LD7-1           CCCAAGTCGCACCGGGCCGATGAGGTACAGCTGTGTGCCCAGGCACTGTGTCTGGAGAGC
LE116-1         CCCAAGTCGCACCGGGCCGATGAGGTACAGCTGTGTGCCCAGGCACTGTGTCTGGAGAGC
LG117           CCCAAGTCGCACCGGGCCGATGAGGTACAGCTGTGTGCCCAGGCACTGTGTCTGGAGAGC
LH37-1          CCCAAGTCGCACCGGGCCGATGAGGTACAGCTGTGTGCCCAGGCACTGTGTCTGGAGAGC
LMG9322         CCCAAGTCGCACCGGGCCGATGAGGTACAGCTGTGTGCCCAGGCACTGTGTCTGGAGAGC
NCPPB3562       CCCAAGTCGCACCGGGCCGATGAGGTACAGCTGTGTGCCCAGGCACTGTGTCTGGAGAGC
NCPPB3610       CCCAAGTCGCACCGGGCCGATGAGGTACAGCTGTGTGCCCAGGCACTGTGTCTGGAGAGC
NCPPB3612       CCCAAGTCGCACCGGGCCGATGAGGTACAGCTGTGTGCCCAGGCACTGTGTCTGGAGAGC
CFBP2911        CCCAAGTCGCACCGGGCCGATGAGGTACAGCTGTGTGCCCAGGCACTGTGTCTGGAGAGC
JF90-2          CCCAAGTCGCACCGGGCCGATGAGGTACAGCTGTGTGCCCAGGCACTGTGTCTGGAGAGC
JF90-8          CCCAAGTCGCACCGGGCCGATGAGGTACAGCTGTGTGCCCAGGCACTGTGTCTGGAGAGC
JJ238-24        CCCAAGTCGCACCGGGCCGATGAGGTACAGCTGTGTGCCCAGGCACTGTGTCTGGAGAGC
JK2-10          CCCAAGTCGCACCGGGCCGATGAGGTACAGCTGTGTGCCCAGGCACTGTGTCTGGAGAGC
JK48            CCCAAGTCGCACCGGGCCGATGAGGTACAGCTGTGTGCCCAGGCACTGTGTCTGGAGAGC
JK143-9         CCCAAGTCGCACCGGGCCGATGAGGTACAGCTGTGTGCCCAGGCACTGTGTCTGGAGAGC
JK143-11        CCCAAGTCGCACCGGGCCGATGAGGTACAGCTGTGTGCCCAGGCACTGTGTCTGGAGAGC
JM35            CCCAAGTCGCACCGGGCCGATGAGGTACAGCTGTGTGCCCAGGCACTGTGTCTGGAGAGC
JS581           CCCAAGTCGCACCGGGCCGATGAGGTACAGCTGTGTGCCCAGGCACTGTGTCTGGAGAGC
JS582           CCCAAGTCGCACCGGGCCGATGAGGTACAGCTGTGTGCCCAGGCACTGTGTCTGGAGAGC
JS584           CCCAAGTCGCACCGGGCCGATGAGGTACAGCTGTGTGCCCAGGCACTGTGTCTGGAGAGC
LB302           CCCAAGTCGCACCGGGCCGATGAGGTACAGCTGTGTGCCCAGGCACTGTGTCTGGAGAGC
LD71a           CCCAAGTCGCACCGGGCCGATGAGGTACAGCTGTGTGCCCAGGCACTGTGTCTGGAGAGC
LE3-1           CCCAAGTCGCACCGGGCCGATGAGGTACAGCTGTGTGCCCAGGCACTGTGTCTGGAGAGC
LE20-1          CCCAAGTCGCACCGGGCCGATGAGGTACAGCTGTGTGCCCAGGCACTGTGTCTGGAGAGC
LG97            CCCAAGTCGCACCGGGCCGATGAGGTACAGCTGTGTGCCCAGGCACTGTGTCTGGAGAGC
LG102           CCCAAGTCGCACCGGGCCGATGAGGTACAGCTGTGTGCCCAGGCACTGTGTCTGGAGAGC
LG115           CCCAAGTCGCACCGGGCCGATGAGGTACAGCTGTGTGCCCAGGCACTGTGTCTGGAGAGC
NCPPB3607       CCCAAGTCGCACCGGGCCGATGAGGTACAGCTGTGTGCCCAGGCACTGTGTCTGGAGAGC
NCPPB3608       CCCAAGTCGCACCGGGCCGATGAGGTACAGCTGTGTGCCCAGGCACTGTGTCTGGAGAGC
NCPPB3615       CCCAAGTCGCACCGGGCCGATGAGGTACAGCTGTGTGCCCAGGCACTGTGTCTGGAGAGC
X2003-3218      CCCAAGTCGCACCGGGCCGATGAGGTACAGCTGTGTGCCCAGGCACTGTGTCTGGAGAGC
                ************************************************************
```

```
C40                ATGCTCAAACGCCCAGTAGACGCCGGCGCGCTGTTCTACGGGCAGCCTCGCAGGCGCAAG
CFBP2852           ATGCTCAAACGCCCAGTAGACGCCGGCGCGCTGTTCTACGGGCAGCCTCGCAGGCGCAAG
FDC217             ATGCTCAAACGCCCAGTAGACGCCGGCGCGCTGTTCTACGGGCAGCCTCGCAGGCGCAAG
FDC1083            ATGCTCAAACGCCCAGTAGACGCCGGCGCGCTGTTCTACGGGCAGCCTCGCAGGCGCAAG
JJ10-1             ATGCTCAAACGCCCAGTAGACGCCGGCGCGCTGTTCTACGGGCAGCCTCGCAGGCGCAAG
JJ238-10           ATGCTCAAACGCCCAGTAGACGCCGGCGCGCTGTTCTACGGGCAGCCTCGCAGGCGCAAG
JK4-1              ATGCTCAAACGCCCAGTAGACGCCGGCGCGCTGTTCTACGGGCAGCCTCGCAGGCGCAAG
JW160-1            ATGCTCAAACGCCCAGTAGACGCCGGCGCGCTGTTCTACGGGCAGCCTCGCAGGCGCAAG
LB100-1            ATGCTCAAACGCCCAGTAGACGCCGGCGCGCTGTTCTACGGGCAGCCTCGCAGGCGCAAG
LC80               ATGCTCAAACGCCCAGTAGACGCCGGCGCGCTGTTCTACGGGCAGCCTCGCAGGCGCAAG
LD7-1              ATGCTCAAACGCCCAGTAGACGCCGGCGCGCTGTTCTACGGGCAGCCTCGCAGGCGCAAG
LE116-1            ATGCTCAAACGCCCAGTAGACGCCGGCGCGCTGTTCTACGGGCAGCCTCGCAGGCGCAAG
LG117              ATGCTCAAACGCCCAGTAGACGCCGGCGCGCTGTTCTACGGGCAGCCTCGCAGGCGCAAG
LH37-1             ATGCTCAAACGCCCAGTAGACGCCGGCGCGCTGTTCTACGGGCAGCCTCGCAGGCGCAAG
LMG9322            ATGCTCAAACGCCCAGTAGACGCCGGCGCGCTGTTCTACGGGCAGCCTCGCAGGCGCAAG
NCPPB3562          ATGCTCAAACGCCCAGTAGACGCCGGCGCGCTGTTCTACGGGCAGCCTCGCAGGCGCAAG
NCPPB3610          ATGCTCAAACGCCCAGTAGACGCCGGCGCGCTGTTCTACGGGCAGCCTCGCAGGCGCAAG
NCPPB3612          ATGCTCAAACGCCCAGTAGACGCCGGCGCGCTGTTCTACGGGCAGCCTCGCAGGCGCAAG
CFBP2911           ATGCTCAAACGCCCAGTAGACGCCGGCGCGCTGTTCTACGGGCAGACTCGCAGGCGCAAG
JF90-2             ATGCTCAAACGCCCAGTAGACGCCGGCGCGCTGTTCTACGGGCAGACTCGCAGGCGCAAG
JF90-8             ATGCTCAAACGCCCAGTAGACGCCGGCGCGCTGTTCTACGGGCAGACTCGCAGGCGCAAG
JJ238-24           ATGCTCAAACGCCCAGTAGACGCCGGCGCGCTGTTCTACGGGCAGACTCGCAGGCGCAAG
JK2-10             ATGCTCAAACGCCCAGTAGACGCCGGCGCGCTGTTCTACGGGCAGACTCGCAGGCGCAAG
JK48               ATGCTCAAACGCCCAGTAGACGCCGGCGCGCTGTTCTACGGGCAGACTCGCAGGCGCAAG
JK143-9            ATGCTCAAACGCCCAGTAGACGCCGGCGCGCTGTTCTACGGGCAGACTCGCAGGCGCAAG
JK143-11           ATGCTCAAACGCCCAGTAGACGCCGGCGCGCTGTTCTACGGGCAGACTCGCAGGCGCAAG
JM35               ATGCTCAAACGCCCAGTAGACGCCGGCGCGCTGTTCTACGGGCAGACTCGCAGGCGCAAG
JS581              ATGCTCAAACGCCCAGTAGACGCCGGCGCGCTGTTCTACGGGCAGACTCGCAGGCGCAAG
JS582              ATGCTCAAACGCCCAGTAGACGCCGGCGCGCTGTTCTACGGGCAGACTCGCAGGCGCAAG
JS584              ATGCTCAAACGCCCAGTAGACGCCGGCGCGCTGTTCTACGGGCAGACTCGCAGGCGCAAG
LB302              ATGCTCAAACGCCCAGTAGACGCCGGCGCGCTGTTCTACGGGCAGACTCGCAGGCGCAAG
LD71a              ATGCTCAAACGCCCAGTAGACGCCGGCGCGCTGTTCTACGGGCAGACTCGCAGGCGCAAG
LE3-1              ATGCTCAAACGCCCAGTAGACGCCGGCGCGCTGTTCTACGGGCAGACTCGCAGGCGCAAG
LE20-1             ATGCTCAAACGCCCAGTAGACGCCGGCGCGCTGTTCTACGGGCAGACTCGCAGGCGCAAG
LG97               ATGCTCAAACGCCCAGTAGACGCCGGCGCGCTGTTCTACGGGCAGACTCGCAGGCGCAAG
LG102              ATGCTCAAACGCCCAGTAGACGCCGGCGCGCTGTTCTACGGGCAGACTCGCAGGCGCAAG
LG115              ATGCTCAAACGCCCAGTAGACGCCGGCGCGCTGTTCTACGGGCAGACTCGCAGGCGCAAG
NCPPB3607          ATGCTCAAACGCCCAGTAGACGCCGGCGCGCTGTTCTACGGGCAGACTCGCAGGCGCAAG
NCPPB3608          ATGCTCAAACGCCCAGTAGACGCCGGCGCGCTGTTCTACGGGCAGACTCGCAGGCGCAAG
NCPPB3615          ATGCTCAAACGCCCAGTAGACGCCGGCGCGCTGTTCTACGGGCAGACTCGCAGGCGCAAG
X2003-3218         ATGCTCAAACGCCCAGTAGACGCCGGCGCGCTGTTCTACGGGCAGACTCGCAGGCGCAAG
                   ********************************************* **************

C40                GATGTGGTCTTCGATCCAGCGCTGCGCGAGCTCACGCAGCGCACCATCGCCGAAACCCGT
CFBP2852           GATGTGGTCTTCGATCCAGCGCTGCGCGAGCTCACGCAGCGCACCATCGCCGAAACCCGT
FDC217             GATGTGGTCTTCGATCCAGCGCTGCGCGAGCTCACGCAGCGCACCATCGCCGAAACCCGT
FDC1083            GATGTGGTCTTCGATCCAGCGCTGCGCGAGCTCACGCAGCGCACCATCGCCGAAACCCGT
JJ10-1             GATGTGGTCTTCGATCCAGCGCTGCGCGAGCTCACGCAGCGCACCATCGCCGAAACCCGT
JJ238-10           GATGTGGTCTTCGATCCAGCGCTGCGCGAGCTCACGCAGCGCACCATCGCCGAAACCCGT
JK4-1              GATGTGGTCTTCGATCCAGCGCTGCGCGAGCTCACGCAGCGCACCATCGCCGAAACCCGT
JW160-1            GATGTGGTCTTCGATCCAGCGCTGCGCGAGCTCACGCAGCGCACCATCGCCGAAACCCGT
LB100-1            GATGTGGTCTTCGATCCAGCGCTGCGCGAGCTCACGCAGCGCACCATCGCCGAAACCCGT
LC80               GATGTGGTCTTCGATCCAGCGCTGCGCGAGCTCACGCAGCGCACCATCGCCGAAACCCGT
LD7-1              GATGTGGTCTTCGATCCAGCGCTGCGCGAGCTCACGCAGCGCACCATCGCCGAAACCCGT
LE116-1            GATGTGGTCTTCGATCCAGCGCTGCGCGAGCTCACGCAGCGCACCATCGCCGAAACCCGT
LG117              GATGTGGTCTTCGATCCAGCGCTGCGCGAGCTCACGCAGCGCACCATCGCCGAAACCCGT
LH37-1             GATGTGGTCTTCGATCCAGCGCTGCGCGAGCTCACGCAGCGCACCATCGCCGAAACCCGT
LMG9322            GATGTGGTCTTCGATCCAGCGCTGCGCGAGCTCACGCAGCGCACCATCGCCGAAACCCGT
NCPPB3562          GATGTGGTCTTCGATCCAGCGCTGCGCGAGCTCACGCAGCGCACCATCGCCGAAACCCGT
NCPPB3610          GATGTGGTCTTCGATCCAGCGCTGCGCGAGCTCACGCAGCGCACCATCGCCGAAACCCGT
NCPPB3612          GATGTGGTCTTCGATCCAGCGCTGCGCGAGCTCACGCAGCGCACCATCGCCGAAACCCGT
CFBP2911           GATGTGGTCTTCGATCCAGCGCTGCGCGAGCTCACGCAGCGCACCATCGCCGAAACCCGT
JF90-2             GATGTGGTCTTCGATCCAGCGCTGCGCGAGCTCACGCAGCGCACCATCGCCGAAACCCGT
JF90-8             GATGTGGTCTTCGATCCAGCGCTGCGCGAGCTCACGCAGCGCACCATCGCCGAAACCCGT
JJ238-24           GATGTGGTCTTCGATCCAGCGCTGCGCGAGCTCACGCAGCGCACCATCGCCGAAACCCGT
JK2-10             GATGTGGTCTTCGATCCAGCGCTGCGCGAGCTCACGCAGCGCACCATCGCCGAAACCCGT
JK48               GATGTGGTCTTCGATCCAGCGCTGCGCGAGCTCACGCAGCGCACCATCGCCGAAACCCGT
JK143-9            GATGTGGTCTTCGATCCAGCGCTGCGCGAGCTCACGCAGCGCACCATCGCCGAAACCCGT
JK143-11           GATGTGGTCTTCGATCCAGCGCTGCGCGAGCTCACGCAGCGCACCATCGCCGAAACCCGT
JM35               GATGTGGTCTTCGATCCAGCGCTGCGCGAGCTCACGCAGCGCACCATCGCCGAAACCCGT
JS581              GATGTGGTCTTCGATCCAGCGCTGCGCGAGCTCACGCAGCGCACCATCGCCGAAACCCGT
JS582              GATGTGGTCTTCGATCCAGCGCTGCGCGAGCTCACGCAGCGCACCATCGCCGAAACCCGT
JS584              GATGTGGTCTTCGATCCAGCGCTGCGCGAGCTCACGCAGCGCACCATCGCCGAAACCCGT
LB302              GATGTGGTCTTCGATCCAGCGCTGCGCGAGCTCACGCAGCGCACCATCGCCGAAACCCGT
LD71a              GATGTGGTCTTCGATCCAGCGCTGCGCGAGCTCACGCAGCGCACCATCGCCGAAACCCGT
LE3-1              GATGTGGTCTTCGATCCAGCGCTGCGCGAGCTCACGCAGCGCACCATCGCCGAAACCCGT
LE20-1             GATGTGGTCTTCGATCCAGCGCTGCGCGAGCTCACGCAGCGCACCATCGCCGAAACCCGT
LG97               GATGTGGTCTTCGATCCAGCGCTGCGCGAGCTCACGCAGCGCACCATCGCCGAAACCCGT
LG102              GATGTGGTCTTCGATCCAGCGCTGCGCGAGCTCACGCAGCGCACCATCGCCGAAACCCGT
LG115              GATGTGGTCTTCGATCCAGCGCTGCGCGAGCTCACGCAGCGCACCATCGCCGAAACCCGT
NCPPB3607          GATGTGGTCTTCGATCCAGCGCTGCGCGAGCTCACGCAGCGCACCATCGCCGAAACCCGT
NCPPB3608          GATGTGGTCTTCGATCCAGCGCTGCGCGAGCTCACGCAGCGCACCATCGCCGAAACCCGT
NCPPB3615          GATGTGGTCTTCGATCCAGCGCTGCGCGAGCTCACGCAGCGCACCATCGCCGAAACCCGT
X2003-3218         GATGTGGTCTTCGATCCAGCGCTGCGCGAGCTCACGCAGCGCACCATCGCCGAAACCCGT
                   ************************************************************
```

```
C40             GCACTGCTGTCTCATGGGCTGACGCCCGGTGCGCGCTATGACAGCAAACGCTGCGATGCC
CFBP2852        GCACTGCTGTCTCATGGGCTGACGCCCGGTGCGCGCTATGACAGCAAACGCTGCGATGCC
FDC217          GCACTGCTGTCTCATGGGCTGACGCCCGGTGCGCGCTATGACAGCAAACGCTGCGATGCC
FDC1083         GCACTGCTGTCTCATGGGCTGACGCCCGGTGCGCGCTATGACAGCAAACGCTGCGATGCC
JJ10-1          GCACTGCTGTCTCATGGGCTGACGCCCGGTGCGCGCTATGACAGCAAACGCTGCGATGCC
JJ238-10        GCACTGCTGTCTCATGGGCTGACGCCCGGTGCGCGCTATGACAGCAAACGCTGCGATGCC
JK4-1           GCACTGCTGTCTCATGGGCTGACGCCCGGTGCGCGCTATGACAGCAAACGCTGCGATGCC
JW160-1         GCACTGCTGTCTCATGGGCTGACGCCCGGTGCGCGCTATGACAGCAAACGCTGCGATGCC
LB100-1         GCACTGCTGTCTCATGGGCTGACGCCCGGTGCGCGCTATGACAGCAAACGCTGCGATGCC
LC80            GCACTGCTGTCTCATGGGCTGACGCCCGGTGCGCGCTATGACAGCAAACGCTGCGATGCC
LD7-1           GCACTGCTGTCTCATGGGCTGACGCCCGGTGCGCGCTATGACAGCAAACGCTGCGATGCC
LE116-1         GCACTGCTGTCTCATGGGCTGACGCCCGGTGCGCGCTATGACAGCAAACGCTGCGATGCC
LG117           GCACTGCTGTCTCATGGGCTGACGCCCGGTGCGCGCTATGACAGCAAACGCTGCGATGCC
LH37-1          GCACTGCTGTCTCATGGGCTGACGCCCGGTGCGCGCTATGACAGCAAACGCTGCGATGCC
LMG9322         GCACTGCTGTCTCATGGGCTGACGCCCGGTGCGCGCTATGACAGCAAACGCTGCGATGCC
NCPPB3562       GCACTGCTGTCTCATGGGCTGACGCCCGGTGCGCGCTATGACAGCAAACGCTGCGATGCC
NCPPB3610       GCACTGCTGTCTCATGGGCTGACGCCCGGTGCGCGCTATGACAGCAAACGCTGCGATGCC
NCPPB3612       GCACTGCTGTCTCATGGGCTGACGCCCGGTGCGCGCTATGACAGCAAACGCTGCGATGCC
CFBP2911        GCACTGCTGTCTCATGGGCTGACGCCCGGTGCGCGCTATGACAGCAAACGCTGCGATGCC
JF90-2          GCACTGCTGTCTCATGGGCTGACGCCCGGTGCGCGCTATGACAGCAAACGCTGCGATGCC
JF90-8          GCACTGCTGTCTCATGGGCTGACGCCCGGTGCGCGCTATGACAGCAAACGCTGCGATGCC
JJ238-24        GCACTGCTGTCTCATGGGCTGACGCCCGGTGCGCGCTATGACAGCAAACGCTGCGATGCC
JK2-10          GCACTGCTGTCTCATGGGCTGACGCCCGGTGCGCGCTATGACAGCAAACGCTGCGATGCC
JK48            GCACTGCTGTCTCATGGGCTGACGCCCGGTGCGCGCTATGACAGCAAACGCTGCGATGCC
JK143-9         GCACTGCTGTCTCATGGGCTGACGCCCGGTGCGCGCTATGACAGCAAACGCTGCGATGCC
JK143-11        GCACTGCTGTCTCATGGGCTGACGCCCGGTGCGCGCTATGACAGCAAACGCTGCGATGCC
JM35            GCACTGCTGTCTCATGGGCTGACGCCCGGTGCGCGCTATGACAGCAAACGCTGCGATGCC
JS581           GCACTGCTGTCTCATGGGCTGACGCCCGGTGCGCGCTATGACAGCAAACGCTGCGATGCC
JS582           GCACTGCTGTCTCATGGGCTGACGCCCGGTGCGCGCTATGACAGCAAACGCTGCGATGCC
JS584           GCACTGCTGTCTCATGGGCTGACGCCCGGTGCGCGCTATGACAGCAAACGCTGCGATGCC
LB302           GCACTGCTGTCTCATGGGCTGACGCCCGGTGCGCGCTATGACAGCAAACGCTGCGATGCC
LD71a           GCACTGCTGTCTCATGGGCTGACGCCCGGTGCGCGCTATGACAGCAAACGCTGCGATGCC
LE3-1           GCACTGCTGTCTCATGGGCTGACGCCCGGTGCGCGCTATGACAGCAAACGCTGCGATGCC
LE20-1          GCACTGCTGTCTCATGGGCTGACGCCCGGTGCGCGCTATGACAGCAAACGCTGCGATGCC
LG97            GCACTGCTGTCTCATGGGCTGACGCCCGGTGCGCGCTATGACAGCAAACGCTGCGATGCC
LG102           GCACTGCTGTCTCATGGGCTGACGCCCGGTGCGCGCTATGACAGCAAACGCTGCGATGCC
LG115           GCACTGCTGTCTCATGGGCTGACGCCCGGTGCGCGCTATGACAGCAAACGCTGCGATGCC
NCPPB3607       GCACTGCTGTCTCATGGGCTGACGCCCGGTGCGCGCTATGACAGCAAACGCTGCGATGCC
NCPPB3608       GCACTGCTGTCTCATGGGCTGACGCCCGGTGCGCGCTATGACAGCAAACGCTGCGATGCC
NCPPB3615       GCACTGCTGTCTCATGGGCTGACGCCCGGTGCGCGCTATGACAGCAAACGCTGCGATGCC
X2003-3218      GCACTGCTGTCTCATGGGCTGACGCCCGGTGCGCGCTATGACAGCAAACGCTGCGATGCC
                ************************************************************

C40             TGTTCATTGATCGACCTGTGTCAGCCGCGCCTGCTCGGCCGCGGCAGCGTGGACACCTGG
CFBP2852        TGTTCATTGATCGACCTGTGTCAGCCGCGCCTGCTCGGCCGCGGCAGCGTGGACACCTGG
FDC217          TGTTCATTGATCGACCTGTGTCAGCCGCGCCTGCTCGGCCGCGGCAGCGTGGACACCTGG
FDC1083         TGTTCATTGATCGACCTGTGTCAGCCGCGCCTGCTCGGCCGCGGCAGCGTGGACACCTGG
JJ10-1          TGTTCATTGATCGACCTGTGTCAGCCGCGCCTGCTCGGCCGCGGCAGCGTGGACACCTGG
JJ238-10        TGTTCATTGATCGACCTGTGTCAGCCGCGCCTGCTCGGCCGCGGCAGCGTGGACACCTGG
JK4-1           TGTTCATTGATCGACCTGTGTCAGCCGCGCCTGCTCGGCCGCGGCAGCGTGGACACCTGG
JW160-1         TGTTCATTGATCGACCTGTGTCAGCCGCGCCTGCTCGGCCGCGGCAGCGTGGACACCTGG
LB100-1         TGTTCATTGATCGACCTGTGTCAGCCGCGCCTGCTCGGCCGCGGCAGCGTGGACACCTGG
LC80            TGTTCATTGATCGACCTGTGTCAGCCGCGCCTGCTCGGCCGCGGCAGCGTGGACACCTGG
LD7-1           TGTTCATTGATCGACCTGTGTCAGCCGCGCCTGCTCGGCCGCGGCAGCGTGGACACCTGG
LE116-1         TGTTCATTGATCGACCTGTGTCAGCCGCGCCTGCTCGGCCGCGGCAGCGTGGACACCTGG
LG117           TGTTCATTGATCGACCTGTGTCAGCCGCGCCTGCTCGGCCGCGGCAGCGTGGACACCTGG
LH37-1          TGTTCATTGATCGACCTGTGTCAGCCGCGCCTGCTCGGCCGCGGCAGCGTGGACACCTGG
LMG9322         TGTTCATTGATCGACCTGTGTCAGCCGCGCCTGCTCGGCCGCGGCAGCGTGGACACCTGG
NCPPB3562       TGTTCATTGATCGACCTGTGTCAGCCGCGCCTGCTCGGCCGCGGCAGCGTGGACACCTGG
NCPPB3610       TGTTCATTGATCGACCTGTGTCAGCCGCGCCTGCTCGGCCGCGGCAGCGTGGACACCTGG
NCPPB3612       TGTTCATTGATCGACCTGTGTCAGCCGCGCCTGCTCGGCCGCGGCAGCGTGGACACCTGG
CFBP2911        TGTTCATTGATCGACCTGTGTCAGCCGCGCCTGCTCGGCCGCGGCAGCGTGGACACCTGG
JF90-2          TGTTCATTGATCGACCTGTGTCAGCCGCGCCTGCTCGGCCGCGGCAGCGTGGACACCTGG
JF90-8          TGTTCATTGATCGACCTGTGTCAGCCGCGCCTGCTCGGCCGCGGCAGCGTGGACACCTGG
JJ238-24        TGTTCATTGATCGACCTGTGTCAGCCGCGCCTGCTCGGCCGCGGCAGCGTGGACACCTGG
JK2-10          TGTTCATTGATCGACCTGTGTCAGCCGCGCCTGCTCGGCCGCGGCAGCGTGGACACCTGG
JK48            TGTTCATTGATCGACCTGTGTCAGCCGCGCCTGCTCGGCCGCGGCAGCGTGGACACCTGG
JK143-9         TGTTCATTGATCGACCTGTGTCAGCCGCGCCTGCTCGGCCGCGGCAGCGTGGACACCTGG
JK143-11        TGTTCATTGATCGACCTGTGTCAGCCGCGCCTGCTCGGCCGCGGCAGCGTGGACACCTGG
JM35            TGTTCATTGATCGACCTGTGTCAGCCGCGCCTGCTCGGCCGCGGCAGCGTGGACACCTGG
JS581           TGTTCATTGATCGACCTGTGTCAGCCGCGCCTGCTCGGCCGCGGCAGCGTGGACACCTGG
JS582           TGTTCATTGATCGACCTGTGTCAGCCGCGCCTGCTCGGCCGCGGCAGCGTGGACACCTGG
JS584           TGTTCATTGATCGACCTGTGTCAGCCGCGCCTGCTCGGCCGCGGCAGCGTGGACACCTGG
LB302           TGTTCATTGATCGACCTGTGTCAGCCGCGCCTGCTCGGCCGCGGCAGCGTGGACACCTGG
LD71a           TGTTCATTGATCGACCTGTGTCAGCCGCGCCTGCTCGGCCGCGGCAGCGTGGACACCTGG
LE3-1           TGTTCATTGATCGACCTGTGTCAGCCGCGCCTGCTCGGCCGCGGCAGCGTGGACACCTGG
LE20-1          TGTTCATTGATCGACCTGTGTCAGCCGCGCCTGCTCGGCCGCGGCAGCGTGGACACCTGG
LG97            TGTTCATTGATCGACCTGTGTCAGCCGCGCCTGCTCGGCCGCGGCAGCGTGGACACCTGG
LG102           TGTTCATTGATCGACCTGTGTCAGCCGCGCCTGCTCGGCCGCGGCAGCGTGGACACCTGG
LG115           TGTTCATTGATCGACCTGTGTCAGCCGCGCCTGCTCGGCCGCGGCAGCGTGGACACCTGG
NCPPB3607       TGTTCATTGATCGACCTGTGTCAGCCGCGCCTGCTCGGCCGCGGCAGCGTGGACACCTGG
NCPPB3608       TGTTCATTGATCGACCTGTGTCAGCCGCGCCTGCTCGGCCGCGGCAGCGTGGACACCTGG
NCPPB3615       TGTTCATTGATCGACCTGTGTCAGCCGCGCCTGCTCGGCCGCGGCAGCGTGGACACCTGG
X2003-3218      TGTTCATTGATCGACCTGTGTCAGCCGCGCCTGCTCGGCCGCGGCAGCGTGGACACCTGG
                ************************************************************
```

```
C40             CTGCGTCGACAACTCGATGCTGAAGAGGAATGA
CFBP2852        CTGCGTCGACAACTCGATGCTGAAGAGGAATGA
FDC217          CTGCGTCGACAACTCGATGCTGAAGAGGAATGA
FDC1083         CTGCGTCGACAACTCGATGCTGAAGAGGAATGA
JJ10-1          CTGCGTCGACAACTCGATGCTGAAGAGGAATGA
JJ238-10        CTGCGTCGACAACTCGATGCTGAAGAGGAATGA
JK4-1           CTGCGTCGACAACTCGATGCTGAAGAGGAATGA
JW160-1         CTGCGTCGACAACTCGATGCTGAAGAGGAATGA
LB100-1         CTGCGTCGACAACTCGATGCTGAAGAGGAATGA
LC80            CTGCGTCGACAACTCGATGCTGAAGAGGAATGA
LD7-1           CTGCGTCGACAACTCGATGCTGAAGAGGAATGA
LE116-1         CTGCGTCGACAACTCGATGCTGAAGAGGAATGA
LG117           CTGCGTCGACAACTCGATGCTGAAGAGGAATGA
LH37-1          CTGCGTCGACAACTCGATGCTGAAGAGGAATGA
LMG9322         CTGCGTCGACAACTCGATGCTGAAGAGGAATGA
NCPPB3562       CTGCGTCGACAACTCGATGCTGAAGAGGAATGA
NCPPB3610       CTGCGTCGACAACTCGATGCTGAAGAGGAATGA
NCPPB3612       CTGCGTCGACAACTCGATGCTGAAGAGGAATGA
CFBP2911        CTGCGTCGACAACTCGATGCTGAAGAGGAATGA
JF90-2          CTGCGTCGACAACTCGATGCTGAAGAGGAATGA
JF90-8          CTGCGTCGACAACTCGATGCTGAAGAGGAATGA
JJ238-24        CTGCGTCGACAACTCGATGCTGAAGAGGAATGA
JK2-10          CTGCGTCGACAACTCGATGCTGAAGAGGAATGA
JK48            CTGCGTCGACAACTCGATGCTGAAGAGGAATGA
JK143-9         CTGCGTCGACAACTCGATGCTGAAGAGGAATGA
JK143-11        CTGCGTCGACAACTCGATGCTGAAGAGGAATGA
JM35            CTGCGTCGACAACTCGATGCTGAAGAGGAATGA
JS581           CTGCGTCGACAACTCGATGCTGAAGAGGAATGA
JS582           CTGCGTCGACAACTCGATGCTGAAGAGGAATGA
JS584           CTGCGTCGACAACTCGATGCTGAAGAGGAATGA
LB302           CTGCGTCGACAACTCGATGCTGAAGAGGAATGA
LD71a           CTGCGTCGACAACTCGATGCTGAAGAGGAATGA
LE3-1           CTGCGTCGACAACTCGATGCTGAAGAGGAATGA
LE20-1          CTGCGTCGACAACTCGATGCTGAAGAGGAATGA
LG97            CTGCGTCGACAACTCGATGCTGAAGAGGAATGA
LG102           CTGCGTCGACAACTCGATGCTGAAGAGGAATGA
LG115           CTGCGTCGACAACTCGATGCTGAAGAGGAATGA
NCPPB3607       CTGCGTCGACAACTCGATGCTGAAGAGGAATGA
NCPPB3608       CTGCGTCGACAACTCGATGCTGAAGAGGAATGA
NCPPB3615       CTGCGTCGACAACTCGATGCTGAAGAGGAATGA
X2003-3218      CTGCGTCGACAACTCGATGCTGAAGAGGAATGA
                *********************************
```

Cas1 (acquisition endonucelase)

```
JF90-8          ATGCGCCGCCAACTCAACACCCTGTATGTCACCACCGATGGTGCCTGGTTGCACAAGGAC
C40             ATGCGCCGCCAACTCAACACCCTGTATGTCACCACCGATGGTGCCTGGTTGCACAAGGAC
CFBP2852        ATGCGCCGCCAACTCAACACCCTGTATGTCACCACCGATGGTGCCTGGTTGCACAAGGAC
CFBP2911        ATGCGCCGCCAACTCAACACCCTGTATGTCACCACCGATGGTGCCTGGTTGCACAAGGAC
FDC217          ATGCGCCGCCAACTCAACACCCTGTATGTCACCACCGATGGTGCCTGGTTGCACAAGGAC
FDC1083         ATGCGCCGCCAACTCAACACCCTGTATGTCACCACCGATGGTGCCTGGTTGCACAAGGAC
JF90-2          ATGCGCCGCCAACTCAACACCCTGTATGTCACCACCGATGGTGCCTGGTTGCACAAGGAC
JJ10-1          ATGCGCCGCCAACTCAACACCCTGTATGTCACCACCGATGGTGCCTGGTTGCACAAGGAC
JJ238-10        ATGCGCCGCCAACTCAACACCCTGTATGTCACCACCGATGGTGCCTGGTTGCACAAGGAC
JJ238-24        ATGCGCCGCCAACTCAACACCCTGTATGTCACCACCGATGGTGCCTGGTTGCACAAGGAC
JK2-10          ATGCGCCGCCAACTCAACACCCTGTATGTCACCACCGATGGTGCCTGGTTGCACAAGGAC
JK4-1           ATGCGCCGCCAACTCAACACCCTGTATGTCACCACCGATGGTGCCTGGTTGCACAAGGAC
JK48            ATGCGCCGCCAACTCAACACCCTGTATGTCACCACCGATGGTGCCTGGTTGCACAAGGAC
JK143-9         ATGCGCCGCCAACTCAACACCCTGTATGTCACCACCGATGGTGCCTGGTTGCACAAGGAC
JK143-11        ATGCGCCGCCAACTCAACACCCTGTATGTCACCACCGATGGTGCCTGGTTGCACAAGGAC
JM35            ATGCGCCGCCAACTCAACACCCTGTATGTCACCACCGATGGTGCCTGGTTGCACAAGGAC
JS581           ATGCGCCGCCAACTCAACACCCTGTATGTCACCACCGATGGTGCCTGGTTGCACAAGGAC
JS582           ATGCGCCGCCAACTCAACACCCTGTATGTCACCACCGATGGTGCCTGGTTGCACAAGGAC
JS584           ATGCGCCGCCAACTCAACACCCTGTATGTCACCACCGATGGTGCCTGGTTGCACAAGGAC
JW160-1         ATGCGCCGCCAACTCAACACCCTGTATGTCACCACCGATGGTGCCTGGTTGCACAAGGAC
LB100-1         ATGCGCCGCCAACTCAACACCCTGTATGTCACCACCGATGGTGCCTGGTTGCACAAGGAC
LC80            ATGCGCCGCCAACTCAACACCCTGTATGTCACCACCGATGGTGCCTGGTTGCACAAGGAC
LD7-1           ATGCGCCGCCAACTCAACACCCTGTATGTCACCACCGATGGTGCCTGGTTGCACAAGGAC
LD71a           ATGCGCCGCCAACTCAACACCCTGTATGTCACCACCGATGGTGCCTGGTTGCACAAGGAC
LE3-1           ATGCGCCGCCAACTCAACACCCTGTATGTCACCACCGATGGTGCCTGGTTGCACAAGGAC
LE20-1          ATGCGCCGCCAACTCAACACCCTGTATGTCACCACCGATGGTGCCTGGTTGCACAAGGAC
LE116-1         ATGCGCCGCCAACTCAACACCCTGTATGTCACCACCGATGGTGCCTGGTTGCACAAGGAC
LG97            ATGCGCCGCCAACTCAACACCCTGTATGTCACCACCGATGGTGCCTGGTTGCACAAGGAC
LG102           ATGCGCCGCCAACTCAACACCCTGTATGTCACCACCGATGGTGCCTGGTTGCACAAGGAC
LG117           ATGCGCCGCCAACTCAACACCCTGTATGTCACCACCGATGGTGCCTGGTTGCACAAGGAC
LH37-1          ATGCGCCGCCAACTCAACACCCTGTATGTCACCACCGATGGTGCCTGGTTGCACAAGGAC
LMG9322         ATGCGCCGCCAACTCAACACCCTGTATGTCACCACCGATGGTGCCTGGTTGCACAAGGAC
NCPPB3562       ATGCGCCGCCAACTCAACACCCTGTATGTCACCACCGATGGTGCCTGGTTGCACAAGGAC
NCPPB3607       ATGCGCCGCCAACTCAACACCCTGTATGTCACCACCGATGGTGCCTGGTTGCACAAGGAC
NCPPB3610       ATGCGCCGCCAACTCAACACCCTGTATGTCACCACCGATGGTGCCTGGTTGCACAAGGAC
NCPPB3612       ATGCGCCGCCAACTCAACACCCTGTATGTCACCACCGATGGTGCCTGGTTGCACAAGGAC
NCPPB3615       ATGCGCCGCCAACTCAACACCCTGTATGTCACCACCGATGGTGCCTGGTTGCACAAGGAC
LB302           ATGCGCCGCCAACTCAACACCCTGTATGTCACCACCGATGGTGCCTGGTTGCACAAGGAC
LG115           ATGCGCCGCCAACTCAACACCCTGTATGTCACCACCGATGGTGCCTGGTTGCACAAGGAC
NCPPB3608       ATGCGCCGCCAACTCAACACCCTGTATGTCACCACCGATGGTGCCTGGTTGCACAAGGAC
X2003-3218      ATGCGCCGCCAACTCAACACCCTGTATGTCACCACCGATGGTGCCTGGTTGCACAAGGAC
                ************************************************************

JF90-8          GGTGCCAATGTCGTCCTGAATGTGGAGCGGCAAGAACGCACACGATTGCCGGTTCACATG
C40             GGTGCCAATGTCGTCCTGAATGTGGAGCGGCAAGAACGCACACGATTGCCGGTTCACATG
CFBP2852        GGTGCCAATGTCGTCCTGAATGTGGAGCGGCAAGAACGCACACGATTGCCGGTTCACATG
CFBP2911        GGTGCCAATGTCGTCCTGAATGTGGAGCGGCAAGAACGCACACGATTGCCGGTTCACATG
FDC217          GGTGCCAATGTCGTCCTGAATGTGGAGCGGCAAGAACGCACACGATTGCCGGTTCACATG
FDC1083         GGTGCCAATGTCGTCCTGAATGTGGAGCGGCAAGAACGCACACGATTGCCGGTTCACATG
JF90-2          GGTGCCAATGTCGTCCTGAATGTGGAGCGGCAAGAACGCACACGATTGCCGGTTCACATG
JJ10-1          GGTGCCAATGTCGTCCTGAATGTGGAGCGGCAAGAACGCACACGATTGCCGGTTCACATG
JJ238-10        GGTGCCAATGTCGTCCTGAATGTGGAGCGGCAAGAACGCACACGATTGCCGGTTCACATG
JJ238-24        GGTGCCAATGTCGTCCTGAATGTGGAGCGGCAAGAACGCACACGATTGCCGGTTCACATG
JK2-10          GGTGCCAATGTCGTCCTGAATGTGGAGCGGCAAGAACGCACACGATTGCCGGTTCACATG
JK4-1           GGTGCCAATGTCGTCCTGAATGTGGAGCGGCAAGAACGCACACGATTGCCGGTTCACATG
JK48            GGTGCCAATGTCGTCCTGAATGTGGAGCGGCAAGAACGCACACGATTGCCGGTTCACATG
JK143-9         GGTGCCAATGTCGTCCTGAATGTGGAGCGGCAAGAACGCACACGATTGCCGGTTCACATG
JK143-11        GGTGCCAATGTCGTCCTGAATGTGGAGCGGCAAGAACGCACACGATTGCCGGTTCACATG
JM35            GGTGCCAATGTCGTCCTGAATGTGGAGCGGCAAGAACGCACACGATTGCCGGTTCACATG
JS581           GGTGCCAATGTCGTCCTGAATGTGGAGCGGCAAGAACGCACACGATTGCCGGTTCACATG
JS582           GGTGCCAATGTCGTCCTGAATGTGGAGCGGCAAGAACGCACACGATTGCCGGTTCACATG
JS584           GGTGCCAATGTCGTCCTGAATGTGGAGCGGCAAGAACGCACACGATTGCCGGTTCACATG
JW160-1         GGTGCCAATGTCGTCCTGAATGTGGAGCGGCAAGAACGCACACGATTGCCGGTTCACATG
LB100-1         GGTGCCAATGTCGTCCTGAATGTGGAGCGGCAAGAACGCACACGATTGCCGGTTCACATG
LC80            GGTGCCAATGTCGTCCTGAATGTGGAGCGGCAAGAACGCACACGATTGCCGGTTCACATG
LD7-1           GGTGCCAATGTCGTCCTGAATGTGGAGCGGCAAGAACGCACACGATTGCCGGTTCACATG
LD71a           GGTGCCAATGTCGTCCTGAATGTGGAGCGGCAAGAACGCACACGATTGCCGGTTCACATG
LE3-1           GGTGCCAATGTCGTCCTGAATGTGGAGCGGCAAGAACGCACACGATTGCCGGTTCACATG
LE20-1          GGTGCCAATGTCGTCCTGAATGTGGAGCGGCAAGAACGCACACGATTGCCGGTTCACATG
LE116-1         GGTGCCAATGTCGTCCTGAATGTGGAGCGGCAAGAACGCACACGATTGCCGGTTCACATG
LG97            GGTGCCAATGTCGTCCTGAATGTGGAGCGGCAAGAACGCACACGATTGCCGGTTCACATG
LG102           GGTGCCAATGTCGTCCTGAATGTGGAGCGGCAAGAACGCACACGATTGCCGGTTCACATG
LG117           GGTGCCAATGTCGTCCTGAATGTGGAGCGGCAAGAACGCACACGATTGCCGGTTCACATG
LH37-1          GGTGCCAATGTCGTCCTGAATGTGGAGCGGCAAGAACGCACACGATTGCCGGTTCACATG
LMG9322         GGTGCCAATGTCGTCCTGAATGTGGAGCGGCAAGAACGCACACGATTGCCGGTTCACATG
NCPPB3562       GGTGCCAATGTCGTCCTGAATGTGGAGCGGCAAGAACGCACACGATTGCCGGTTCACATG
NCPPB3607       GGTGCCAATGTCGTCCTGAATGTGGAGCGGCAAGAACGCACACGATTGCCGGTTCACATG
NCPPB3610       GGTGCCAATGTCGTCCTGAATGTGGAGCGGCAAGAACGCACACGATTGCCGGTTCACATG
NCPPB3612       GGTGCCAATGTCGTCCTGAATGTGGAGCGGCAAGAACGCACACGATTGCCGGTTCACATG
NCPPB3615       GGTGCCAATGTCGTCCTGAATGTGGAGCGGCAAGAACGCACACGATTGCCGGTTCACATG
LB302           GGTGCCAATGTCGTCCTGAATGTGGAGCGGCAAGAACGCACACGATTGCCGGTTCACATG
LG115           GGTGCCAATGTCGTCCTGAATGTGGAGCGGCAAGAACGCACACGATTGCCGGTTCACATG
NCPPB3608       GGTGCCAATGTCGTCCTGAATGTGGAGCGGCAAGAACGCACACGATTGCCGGTTCACATG
X2003-3218      GGTGCCAATGTCGTCCTGAATGTGGAGCGGCAAGAACGCACACGATTGCCGGTTCACATG
                ************************************************************
```

```
JF90-8          CTGGAGAGCATCGTCTGCATCGGACGTGTCGGAGTCTCGCCTCAGTTACTCGGCTTCTGT
C40             CTGGAGAGCATCGTCTGCATCGGACGTGTCGGAGTCTCGCCTCAGTTACTCGGCTTCTGT
CFBP2852        CTGGAGAGCATCGTCTGCATCGGACGTGTCGGAGTCTCGCCTCAGTTACTCGGCTTCTGT
CFBP2911        CTGGAGAGCATCGTCTGCATCGGACGTGTCGGAGTCTCGCCTCAGTTACTCGGCTTCTGT
FDC217          CTGGAGAGCATCGTCTGCATCGGACGTGTCGGAGTCTCGCCTCAGTTACTCGGCTTCTGT
FDC1083         CTGGAGAGCATCGTCTGCATCGGACGTGTCGGAGTCTCGCCTCAGTTACTCGGCTTCTGT
JF90-2          CTGGAGAGCATCGTCTGCATCGGACGTGTCGGAGTCTCGCCTCAGTTACTCGGCTTCTGT
JJ10-1          CTGGAGAGCATCGTCTGCATCGGACGTGTCGGAGTCTCGCCTCAGTTACTCGGCTTCTGT
JJ238-10        CTGGAGAGCATCGTCTGCATCGGACGTGTCGGAGTCTCGCCTCAGTTACTCGGCTTCTGT
JJ238-24        CTGGAGAGCATCGTCTGCATCGGACGTGTCGGAGTCTCGCCTCAGTTACTCGGCTTCTGT
JK2-10          CTGGAGAGCATCGTCTGCATCGGACGTGTCGGAGTCTCGCCTCAGTTACTCGGCTTCTGT
JK4-1           CTGGAGAGCATCGTCTGCATCGGACGTGTCGGAGTCTCGCCTCAGTTACTCGGCTTCTGT
JK48            CTGGAGAGCATCGTCTGCATCGGACGTGTCGGAGTCTCGCCTCAGTTACTCGGCTTCTGT
JK143-9         CTGGAGAGCATCGTCTGCATCGGACGTGTCGGAGTCTCGCCTCAGTTACTCGGCTTCTGT
JK143-11        CTGGAGAGCATCGTCTGCATCGGACGTGTCGGAGTCTCGCCTCAGTTACTCGGCTTCTGT
JM35            CTGGAGAGCATCGTCTGCATCGGACGTGTCGGAGTCTCGCCTCAGTTACTCGGCTTCTGT
JS581           CTGGAGAGCATCGTCTGCATCGGACGTGTCGGAGTCTCGCCTCAGTTACTCGGCTTCTGT
JS582           CTGGAGAGCATCGTCTGCATCGGACGTGTCGGAGTCTCGCCTCAGTTACTCGGCTTCTGT
JS584           CTGGAGAGCATCGTCTGCATCGGACGTGTCGGAGTCTCGCCTCAGTTACTCGGCTTCTGT
JW160-1         CTGGAGAGCATCGTCTGCATCGGACGTGTCGGAGTCTCGCCTCAGTTACTCGGCTTCTGT
LB100-1         CTGGAGAGCATCGTCTGCATCGGACGTGTCGGAGTCTCGCCTCAGTTACTCGGCTTCTGT
LC80            CTGGAGAGCATCGTCTGCATCGGACGTGTCGGAGTCTCGCCTCAGTTACTCGGCTTCTGT
LD7-1           CTGGAGAGCATCGTCTGCATCGGACGTGTCGGAGTCTCGCCTCAGTTACTCGGCTTCTGT
LD71a           CTGGAGAGCATCGTCTGCATCGGACGTGTCGGAGTCTCGCCTCAGTTACTCGGCTTCTGT
LE3-1           CTGGAGAGCATCGTCTGCATCGGACGTGTCGGAGTCTCGCCTCAGTTACTCGGCTTCTGT
LE20-1          CTGGAGAGCATCGTCTGCATCGGACGTGTCGGAGTCTCGCCTCAGTTACTCGGCTTCTGT
LE116-1         CTGGAGAGCATCGTCTGCATCGGACGTGTCGGAGTCTCGCCTCAGTTACTCGGCTTCTGT
LG97            CTGGAGAGCATCGTCTGCATCGGACGTGTCGGAGTCTCGCCTCAGTTACTCGGCTTCTGT
LG102           CTGGAGAGCATCGTCTGCATCGGACGTGTCGGAGTCTCGCCTCAGTTACTCGGCTTCTGT
LG117           CTGGAGAGCATCGTCTGCATCGGACGTGTCGGAGTCTCGCCTCAGTTACTCGGCTTCTGT
LH37-1          CTGGAGAGCATCGTCTGCATCGGACGTGTCGGAGTCTCGCCTCAGTTACTCGGCTTCTGT
LMG9322         CTGGAGAGCATCGTCTGCATCGGACGTGTCGGAGTCTCGCCTCAGTTACTCGGCTTCTGT
NCPPB3562       CTGGAGAGCATCGTCTGCATCGGACGTGTCGGAGTCTCGCCTCAGTTACTCGGCTTCTGT
NCPPB3607       CTGGAGAGCATCGTCTGCATCGGACGTGTCGGAGTCTCGCCTCAGTTACTCGGCTTCTGT
NCPPB3610       CTGGAGAGCATCGTCTGCATCGGACGTGTCGGAGTCTCGCCTCAGTTACTCGGCTTCTGT
NCPPB3612       CTGGAGAGCATCGTCTGCATCGGACGTGTCGGAGTCTCGCCTCAGTTACTCGGCTTCTGT
NCPPB3615       CTGGAGAGCATCGTCTGCATCGGACGTGTCGGAGTCTCGCCTCAGTTACTCGGCTTCTGT
LB302           CTGGAGAGCATCGTCTGCATCGGACGTGTCGGAGTCTCGCCTCAGTTACTCGGCTTCTGT
LG115           CTGGAGAGCATCGTCTGCATCGGACGTGTCGGAGTCTCGCCTCAGTTACTCGGCTTCTGT
NCPPB3608       CTGGAGAGCATCGTCTGCATCGGACGTGTCGGAGTCTCGCCTCAGTTACTCGGCTTCTGT
X2003-3218      CTGGAGAGCATCGTCTGCATCGGACGTGTCGGAGTCTCGCCTCAGTTACTCGGCTTCTGT
                ************************************************************

JF90-8          GCCGAGCAAGGCATCAGCATCTGTTATTTGACTGCGCAAGGGCGCTTTCTCGCGCGTGTC
C40             GCCGAGCAAGGCATCAGCATCTGTTATTTGACTGCGCAAGGGCGCTTTCTCGCGCGTGTC
CFBP2852        GCCGAGCAAGGCATCAGCATCTGTTATTTGACTGCGCAAGGGCGCTTTCTCGCGCGTGTC
CFBP2911        GCCGAGCAAGGCATCAGCATCTGTTATTTGACTGCGCAAGGGCGCTTTCTCGCGCGTGTC
FDC217          GCCGAGCAAGGCATCAGCATCTGTTATTTGACTGCGCAAGGGCGCTTTCTCGCGCGTGTC
FDC1083         GCCGAGCAAGGCATCAGCATCTGTTATTTGACTGCGCAAGGGCGCTTTCTCGCGCGTGTC
JF90-2          GCCGAGCAAGGCATCAGCATCTGTTATTTGACTGCGCAAGGGCGCTTTCTCGCGCGTGTC
JJ10-1          GCCGAGCAAGGCATCAGCATCTGTTATTTGACTGCGCAAGGGCGCTTTCTCGCGCGTGTC
JJ238-10        GCCGAGCAAGGCATCAGCATCTGTTATTTGACTGCGCAAGGGCGCTTTCTCGCGCGTGTC
JJ238-24        GCCGAGCAAGGCATCAGCATCTGTTATTTGACTGCGCAAGGGCGCTTTCTCGCGCGTGTC
JK2-10          GCCGAGCAAGGCATCAGCATCTGTTATTTGACTGCGCAAGGGCGCTTTCTCGCGCGTGTC
JK4-1           GCCGAGCAAGGCATCAGCATCTGTTATTTGACTGCGCAAGGGCGCTTTCTCGCGCGTGTC
JK48            GCCGAGCAAGGCATCAGCATCTGTTATTTGACTGCGCAAGGGCGCTTTCTCGCGCGTGTC
JK143-9         GCCGAGCAAGGCATCAGCATCTGTTATTTGACTGCGCAAGGGCGCTTTCTCGCGCGTGTC
JK143-11        GCCGAGCAAGGCATCAGCATCTGTTATTTGACTGCGCAAGGGCGCTTTCTCGCGCGTGTC
JM35            GCCGAGCAAGGCATCAGCATCTGTTATTTGACTGCGCAAGGGCGCTTTCTCGCGCGTGTC
JS581           GCCGAGCAAGGCATCAGCATCTGTTATTTGACTGCGCAAGGGCGCTTTCTCGCGCGTGTC
JS582           GCCGAGCAAGGCATCAGCATCTGTTATTTGACTGCGCAAGGGCGCTTTCTCGCGCGTGTC
JS584           GCCGAGCAAGGCATCAGCATCTGTTATTTGACTGCGCAAGGGCGCTTTCTCGCGCGTGTC
JW160-1         GCCGAGCAAGGCATCAGCATCTGTTATTTGACTGCGCAAGGGCGCTTTCTCGCGCGTGTC
LB100-1         GCCGAGCAAGGCATCAGCATCTGTTATTTGACTGCGCAAGGGCGCTTTCTCGCGCGTGTC
LC80            GCCGAGCAAGGCATCAGCATCTGTTATTTGACTGCGCAAGGGCGCTTTCTCGCGCGTGTC
LD7-1           GCCGAGCAAGGCATCAGCATCTGTTATTTGACTGCGCAAGGGCGCTTTCTCGCGCGTGTC
LD71a           GCCGAGCAAGGCATCAGCATCTGTTATTTGACTGCGCAAGGGCGCTTTCTCGCGCGTGTC
LE3-1           GCCGAGCAAGGCATCAGCATCTGTTATTTGACTGCGCAAGGGCGCTTTCTCGCGCGTGTC
LE20-1          GCCGAGCAAGGCATCAGCATCTGTTATTTGACTGCGCAAGGGCGCTTTCTCGCGCGTGTC
LE116-1         GCCGAGCAAGGCATCAGCATCTGTTATTTGACTGCGCAAGGGCGCTTTCTCGCGCGTGTC
LG97            GCCGAGCAAGGCATCAGCATCTGTTATTTGACTGCGCAAGGGCGCTTTCTCGCGCGTGTC
LG102           GCCGAGCAAGGCATCAGCATCTGTTATTTGACTGCGCAAGGGCGCTTTCTCGCGCGTGTC
LG117           GCCGAGCAAGGCATCAGCATCTGTTATTTGACTGCGCAAGGGCGCTTTCTCGCGCGTGTC
LH37-1          GCCGAGCAAGGCATCAGCATCTGTTATTTGACTGCGCAAGGGCGCTTTCTCGCGCGTGTC
LMG9322         GCCGAGCAAGGCATCAGCATCTGTTATTTGACTGCGCAAGGGCGCTTTCTCGCGCGTGTC
NCPPB3562       GCCGAGCAAGGCATCAGCATCTGTTATTTGACTGCGCAAGGGCGCTTTCTCGCGCGTGTC
NCPPB3607       GCCGAGCAAGGCATCAGCATCTGTTATTTGACTGCGCAAGGGCGCTTTCTCGCGCGTGTC
NCPPB3610       GCCGAGCAAGGCATCAGCATCTGTTATTTGACTGCGCAAGGGCGCTTTCTCGCGCGTGTC
NCPPB3612       GCCGAGCAAGGCATCAGCATCTGTTATTTGACTGCGCAAGGGCGCTTTCTCGCGCGTGTC
NCPPB3615       GCCGAGCAAGGCATCAGCATCTGTTATTTGACTGCGCAAGGGCGCTTTCTCGCGCGTGTC
LB302           GCCGAGCAAGGCATCAGCATCTGTTATTTGACTGCGCAAGGGCGCTTTCTCGCGCGTGTC
LG115           GCCGAGCAAGGCATCAGCATCTGTTATTTGACTGCGCAAGGGCGCTTTCTCGCGCGTGTC
NCPPB3608       GCCGAGCAAGGCATCAGCATCTGTTATTTGACTGCGCAAGGGCGCTTTCTCGCGCGTGTC
X2003-3218      GCCGAGCAAGGCATCAGCATCTGTTATTTGACTGCGCAAGGGCGCTTTCTCGCGCGTGTC
                ************************************************************
```

```
JF90-8          GAAGGCCCGGTGTCCGGAATGTGCTGCTTCGCCGCGAACAGTACCGATGCAGCGACGAC
C40             GAAGGCCCGGTGTCCGGAATGTGCTGCTTCGCCGCGAACAGTACCGATGCAGCGACGAC
CFBP2852        GAAGGCCCGGTGTCCGGAATGTGCTGCTTCGCCGCGAACAGTACCGATGCAGCGACGAC
CFBP2911        GAAGGCCCGGTGTCCGGAATGTGCTGCTTCGCCGCGAACAGTACCGATGCAGCGACGAC
FDC217          GAAGGCCCGGTGTCCGGAATGTGCTGCTTCGCCGCGAACAGTACCGATGCAGCGACGAC
FDC1083         GAAGGCCCGGTGTCCGGAATGTGCTGCTTCGCCGCGAACAGTACCGATGCAGCGACGAC
JF90-2          GAAGGCCCGGTGTCCGGAATGTGCTGCTTCGCCGCGAACAGTACCGATGCAGCGACGAC
JJ10-1          GAAGGCCCGGTGTCCGGAATGTGCTGCTTCGCCGCGAACAGTACCGATGCAGCGACGAC
JJ238-10        GAAGGCCCGGTGTCCGGAATGTGCTGCTTCGCCGCGAACAGTACCGATGCAGCGACGAC
JJ238-24        GAAGGCCCGGTGTCCGGAATGTGCTGCTTCGCCGCGAACAGTACCGATGCAGCGACGAC
JK2-10          GAAGGCCCGGTGTCCGGAATGTGCTGCTTCGCCGCGAACAGTACCGATGCAGCGACGAC
JK4-1           GAAGGCCCGGTGTCCGGAATGTGCTGCTTCGCCGCGAACAGTACCGATGCAGCGACGAC
JK48            GAAGGCCCGGTGTCCGGAATGTGCTGCTTCGCCGCGAACAGTACCGATGCAGCGACGAC
JK143-9         GAAGGCCCGGTGTCCGGAATGTGCTGCTTCGCCGCGAACAGTACCGATGCAGCGACGAC
JK143-11        GAAGGCCCGGTGTCCGGAATGTGCTGCTTCGCCGCGAACAGTACCGATGCAGCGACGAC
JM35            GAAGGCCCGGTGTCCGGAATGTGCTGCTTCGCCGCGAACAGTACCGATGCAGCGACGAC
JS581           GAAGGCCCGGTGTCCGGAATGTGCTGCTTCGCCGCGAACAGTACCGATGCAGCGACGAC
JS582           GAAGGCCCGGTGTCCGGAATGTGCTGCTTCGCCGCGAACAGTACCGATGCAGCGACGAC
JS584           GAAGGCCCGGTGTCCGGAATGTGCTGCTTCGCCGCGAACAGTACCGATGCAGCGACGAC
JW160-1         GAAGGCCCGGTGTCCGGAATGTGCTGCTTCGCCGCGAACAGTACCGATGCAGCGACGAC
LB100-1         GAAGGCCCGGTGTCCGGAATGTGCTGCTTCGCCGCGAACAGTACCGATGCAGCGACGAC
LC80            GAAGGCCCGGTGTCCGGAATGTGCTGCTTCGCCGCGAACAGTACCGATGCAGCGACGAC
LD7-1           GAAGGCCCGGTGTCCGGAATGTGCTGCTTCGCCGCGAACAGTACCGATGCAGCGACGAC
LD71a           GAAGGCCCGGTGTCCGGAATGTGCTGCTTCGCCGCGAACAGTACCGATGCAGCGACGAC
LE3-1           GAAGGCCCGGTGTCCGGAATGTGCTGCTTCGCCGCGAACAGTACCGATGCAGCGACGAC
LE20-1          GAAGGCCCGGTGTCCGGAATGTGCTGCTTCGCCGCGAACAGTACCGATGCAGCGACGAC
LE116-1         GAAGGCCCGGTGTCCGGAATGTGCTGCTTCGCCGCGAACAGTACCGATGCAGCGACGAC
LG97            GAAGGCCCGGTGTCCGGAATGTGCTGCTTCGCCGCGAACAGTACCGATGCAGCGACGAC
LG102           GAAGGCCCGGTGTCCGGAATGTGCTGCTTCGCCGCGAACAGTACCGATGCAGCGACGAC
LG117           GAAGGCCCGGTGTCCGGAATGTGCTGCTTCGCCGCGAACAGTACCGATGCAGCGACGAC
LH37-1          GAAGGCCCGGTGTCCGGAATGTGCTGCTTCGCCGCGAACAGTACCGATGCAGCGACGAC
LMG9322         GAAGGCCCGGTGTCCGGAATGTGCTGCTTCGCCGCGAACAGTACCGATGCAGCGACGAC
NCPPB3562       GAAGGCCCGGTGTCCGGAATGTGCTGCTTCGCCGCGAACAGTACCGATGCAGCGACGAC
NCPPB3607       GAAGGCCCGGTGTCCGGAATGTGCTGCTTCGCCGCGAACAGTACCGATGCAGCGACGAC
NCPPB3610       GAAGGCCCGGTGTCCGGAATGTGCTGCTTCGCCGCGAACAGTACCGATGCAGCGACGAC
NCPPB3612       GAAGGCCCGGTGTCCGGAATGTGCTGCTTCGCCGCGAACAGTACCGATGCAGCGACGAC
NCPPB3615       GAAGGCCCGGTGTCCGGAATGTGCTGCTTCGCCGCGAACAGTACCGATGCAGCGACGAC
LB302           GAAGGCCCGGTGTCCGGAATGTGCTGCTTCGCCGCGAACAGTACCGATGCAGCGACGAC
LG115           GAAGGCCCGGTGTCCGGAATGTGCTGCTTCGCCGCGAACAGTACCGATGCAGCGACGAC
NCPPB3608       GAAGGCCCGGTGTCCGGAATGTGCTGCTTCGCCGCGAACAGTACCGATGCAGCGACGAC
X2003-3218      GAAGGCCCGGTGTCCGGAATGTGCTGCTTCGCCGCGAACAGTACCGATGCAGCGACGAC
                ************************************************************

JF90-8          CCCGTGCGTTGCGCCGCGATTGTGCGTCACATGCTGGCAGGAAAAATCCACAACCAGCGT
C40             CCCGTGCGTTGCGCCGCGATTGTGCGTCACATGCTGGCAGGAAAAATCCACAACCAGCGT
CFBP2852        CCCGTGCGTTGCGCCGCGATTGTGCGTCACATGCTGGCAGGAAAAATCCACAACCAGCGT
CFBP2911        CCCGTGCGTTGCGCCGCGATTGTGCGTCACATGCTGGCAGGAAAAATCCACAACCAGCGT
FDC217          CCCGTGCGTTGCGCCGCGATTGTGCGTCACATGCTGGCAGGAAAAATCCACAACCAGCGT
FDC1083         CCCGTGCGTTGCGCCGCGATTGTGCGTCACATGCTGGCAGGAAAAATCCACAACCAGCGT
JF90-2          CCCGTGCGTTGCGCCGCGATTGTGCGTCACATGCTGGCAGGAAAAATCCACAACCAGCGT
JJ10-1          CCCGTGCGTTGCGCCGCGATTGTGCGTCACATGCTGGCAGGAAAAATCCACAACCAGCGT
JJ238-10        CCCGTGCGTTGCGCCGCGATTGTGCGTCACATGCTGGCAGGAAAAATCCACAACCAGCGT
JJ238-24        CCCGTGCGTTGCGCCGCGATTGTGCGTCACATGCTGGCAGGAAAAATCCACAACCAGCGT
JK2-10          CCCGTGCGTTGCGCCGCGATTGTGCGTCACATGCTGGCAGGAAAAATCCACAACCAGCGT
JK4-1           CCCGTGCGTTGCGCCGCGATTGTGCGTCACATGCTGGCAGGAAAAATCCACAACCAGCGT
JK48            CCCGTGCGTTGCGCCGCGATTGTGCGTCACATGCTGGCAGGAAAAATCCACAACCAGCGT
JK143-9         CCCGTGCGTTGCGCCGCGATTGTGCGTCACATGCTGGCAGGAAAAATCCACAACCAGCGT
JK143-11        CCCGTGCGTTGCGCCGCGATTGTGCGTCACATGCTGGCAGGAAAAATCCACAACCAGCGT
JM35            CCCGTGCGTTGCGCCGCGATTGTGCGTCACATGCTGGCAGGAAAAATCCACAACCAGCGT
JS581           CCCGTGCGTTGCGCCGCGATTGTGCGTCACATGCTGGCAGGAAAAATCCACAACCAGCGT
JS582           CCCGTGCGTTGCGCCGCGATTGTGCGTCACATGCTGGCAGGAAAAATCCACAACCAGCGT
JS584           CCCGTGCGTTGCGCCGCGATTGTGCGTCACATGCTGGCAGGAAAAATCCACAACCAGCGT
JW160-1         CCCGTGCGTTGCGCCGCGATTGTGCGTCACATGCTGGCAGGAAAAATCCACAACCAGCGT
LB100-1         CCCGTGCGTTGCGCCGCGATTGTGCGTCACATGCTGGCAGGAAAAATCCACAACCAGCGT
LC80            CCCGTGCGTTGCGCCGCGATTGTGCGTCACATGCTGGCAGGAAAAATCCACAACCAGCGT
LD7-1           CCCGTGCGTTGCGCCGCGATTGTGCGTCACATGCTGGCAGGAAAAATCCACAACCAGCGT
LD71a           CCCGTGCGTTGCGCCGCGATTGTGCGTCACATGCTGGCAGGAAAAATCCACAACCAGCGT
LE3-1           CCCGTGCGTTGCGCCGCGATTGTGCGTCACATGCTGGCAGGAAAAATCCACAACCAGCGT
LE20-1          CCCGTGCGTTGCGCCGCGATTGTGCGTCACATGCTGGCAGGAAAAATCCACAACCAGCGT
LE116-1         CCCGTGCGTTGCGCCGCGATTGTGCGTCACATGCTGGCAGGAAAAATCCACAACCAGCGT
LG97            CCCGTGCGTTGCGCCGCGATTGTGCGTCACATGCTGGCAGGAAAAATCCACAACCAGCGT
LG102           CCCGTGCGTTGCGCCGCGATTGTGCGTCACATGCTGGCAGGAAAAATCCACAACCAGCGT
LG117           CCCGTGCGTTGCGCCGCGATTGTGCGTCACATGCTGGCAGGAAAAATCCACAACCAGCGT
LH37-1          CCCGTGCGTTGCGCCGCGATTGTGCGTCACATGCTGGCAGGAAAAATCCACAACCAGCGT
LMG9322         CCCGTGCGTTGCGCCGCGATTGTGCGTCACATGCTGGCAGGAAAAATCCACAACCAGCGT
NCPPB3562       CCCGTGCGTTGCGCCGCGATTGTGCGTCACATGCTGGCAGGAAAAATCCACAACCAGCGT
NCPPB3607       CCCGTGCGTTGCGCCGCGATTGTGCGTCACATGCTGGCAGGAAAAATCCACAACCAGCGT
NCPPB3610       CCCGTGCGTTGCGCCGCGATTGTGCGTCACATGCTGGCAGGAAAAATCCACAACCAGCGT
NCPPB3612       CCCGTGCGTTGCGCCGCGATTGTGCGTCACATGCTGGCAGGAAAAATCCACAACCAGCGT
NCPPB3615       CCCGTGCGTTGCGCCGCGATTGTGCGTCACATGCTGGCAGGAAAAATCCACAACCAGCGT
LB302           CCCGTGCGTTGCGCCGCGATTGTGGGTCACATGCTGGCAGGAAAAATCCACAACCAGCGT
LG115           CCCGTGCGTTGCGCCGCGATTGTGGGTCACATGCTGGCAGGAAAAATCCACAACCAGCGT
NCPPB3608       CCCGTGCGTTGCGCCGCGATTGTGGGTCACATGCTGGCAGGAAAAATCCACAACCAGCGT
X2003-3218      CCCGTGCGTTGCGCCGCGATTGTGGGTCACATGCTGGCAGGAAAAATCCACAACCAGCGT
                ************************ ***********************************
```

```
JF90-8          GCCGTCCTGGCGCGCGGCTGGCGCGACCACGGCGACCGCATGATCGATATCCCCGCCTTT
C40             GCCGTCCTGGCGCGCGGCTGGCGCGACCACGGCGACCGCATGATCGATATCCCCGCCTTT
CFBP2852        GCCGTCCTGGCGCGCGGCTGGCGCGACCACGGCGACCGCATGATCGATATCCCCGCCTTT
CFBP2911        GCCGTCCTGGCGCGCGGCTGGCGCGACCACGGCGACCGCATGATCGATATCCCCGCCTTT
FDC217          GCCGTCCTGGCGCGCGGCTGGCGCGACCACGGCGACCGCATGATCGATATCCCCGCCTTT
FDC1083         GCCGTCCTGGCGCGCGGCTGGCGCGACCACGGCGACCGCATGATCGATATCCCCGCCTTT
JF90-2          GCCGTCCTGGCGCGCGGCTGGCGCGACCACGGCGACCGCATGATCGATATCCCCGCCTTT
JJ10-1          GCCGTCCTGGCGCGCGGCTGGCGCGACCACGGCGACCGCATGATCGATATCCCCGCCTTT
JJ238-10        GCCGTCCTGGCGCGCGGCTGGCGCGACCACGGCGACCGCATGATCGATATCCCCGCCTTT
JJ238-24        GCCGTCCTGGCGCGCGGCTGGCGCGACCACGGCGACCGCATGATCGATATCCCCGCCTTT
JK2-10          GCCGTCCTGGCGCGCGGCTGGCGCGACCACGGCGACCGCATGATCGATATCCCCGCCTTT
JK4-1           GCCGTCCTGGCGCGCGGCTGGCGCGACCACGGCGACCGCATGATCGATATCCCCGCCTTT
JK48            GCCGTCCTGGCGCGCGGCTGGCGCGACCACGGCGACCGCATGATCGATATCCCCGCCTTT
JK143-9         GCCGTCCTGGCGCGCGGCTGGCGCGACCACGGCGACCGCATGATCGATATCCCCGCCTTT
JK143-11        GCCGTCCTGGCGCGCGGCTGGCGCGACCACGGCGACCGCATGATCGATATCCCCGCCTTT
JM35            GCCGTCCTGGCGCGCGGCTGGCGCGACCACGGCGACCGCATGATCGATATCCCCGCCTTT
JS581           GCCGTCCTGGCGCGCGGCTGGCGCGACCACGGCGACCGCATGATCGATATCCCCGCCTTT
JS582           GCCGTCCTGGCGCGCGGCTGGCGCGACCACGGCGACCGCATGATCGATATCCCCGCCTTT
JS584           GCCGTCCTGGCGCGCGGCTGGCGCGACCACGGCGACCGCATGATCGATATCCCCGCCTTT
JW160-1         GCCGTCCTGGCGCGCGGCTGGCGCGACCACGGCGACCGCATGATCGATATCCCCGCCTTT
LB100-1         GCCGTCCTGGCGCGCGGCTGGCGCGACCACGGCGACCGCATGATCGATATCCCCGCCTTT
LC80            GCCGTCCTGGCGCGCGGCTGGCGCGACCACGGCGACCGCATGATCGATATCCCCGCCTTT
LD7-1           GCCGTCCTGGCGCGCGGCTGGCGCGACCACGGCGACCGCATGATCGATATCCCCGCCTTT
LD71a           GCCGTCCTGGCGCGCGGCTGGCGCGACCACGGCGACCGCATGATCGATATCCCCGCCTTT
LE3-1           GCCGTCCTGGCGCGCGGCTGGCGCGACCACGGCGACCGCATGATCGATATCCCCGCCTTT
LE20-1          GCCGTCCTGGCGCGCGGCTGGCGCGACCACGGCGACCGCATGATCGATATCCCCGCCTTT
LE116-1         GCCGTCCTGGCGCGCGGCTGGCGCGACCACGGCGACCGCATGATCGATATCCCCGCCTTT
LG97            GCCGTCCTGGCGCGCGGCTGGCGCGACCACGGCGACCGCATGATCGATATCCCCGCCTTT
LG102           GCCGTCCTGGCGCGCGGCTGGCGCGACCACGGCGACCGCATGATCGATATCCCCGCCTTT
LG117           GCCGTCCTGGCGCGCGGCTGGCGCGACCACGGCGACCGCATGATCGATATCCCCGCCTTT
LH37-1          GCCGTCCTGGCGCGCGGCTGGCGCGACCACGGCGACCGCATGATCGATATCCCCGCCTTT
LMG9322         GCCGTCCTGGCGCGCGGCTGGCGCGACCACGGCGACCGCATGATCGATATCCCCGCCTTT
NCPPB3562       GCCGTCCTGGCGCGCGGCTGGCGCGACCACGGCGACCGCATGATCGATATCCCCGCCTTT
NCPPB3607       GCCGTCCTGGCGCGCGGCTGGCGCGACCACGGCGACCGCATGATCGATATCCCCGCCTTT
NCPPB3610       GCCGTCCTGGCGCGCGGCTGGCGCGACCACGGCGACCGCATGATCGATATCCCCGCCTTT
NCPPB3612       GCCGTCCTGGCGCGCGGCTGGCGCGACCACGGCGACCGCATGATCGATATCCCCGCCTTT
NCPPB3615       GCCGTCCTGGCGCGCGGCTGGCGCGACCACGGCGACCGCATGATCGATATCCCCGCCTTT
LB302           GCCGTCCTGGCGCGCGGCTGGCGCGACCACGGCGACCGCATGATCGATATCCCCGCCTTT
LG115           GCCGTCCTGGCGCGCGGCTGGCGCGACCACGGCGACCGCATGATCGATATCCCCGCCTTT
NCPPB3608       GCCGTCCTGGCGCGCGGCTGGCGCGACCACGGCGACCGCATGATCGATATCCCCGCCTTT
X2003-3218      GCCGTCCTGGCGCGCGGCTGGCGCGACCACGGCGACCGCATGATCGATATCCCCGCCTTT
                ************************************************************

JF90-8          CAACACGCACTCAAGCGACTCAAGCGCATTCCACAGCGCCTGCTCATCGAGACCAGTGTT
C40             CAACACGCACTCAAGCGACTCAAGCGCATTCCACAGCGCCTGCTCATCGAGACCAGTGTT
CFBP2852        CAACACGCACTCAAGCGACTCAAGCGCATTCCACAGCGCCTGCTCATCGAGACCAGTGTT
CFBP2911        CAACACGCACTCAAGCGACTCAAGCGCATTCCACAGCGCCTGCTCATCGAGACCAGTGTT
FDC217          CAACACGCACTCAAGCGACTCAAGCGCATTCCACAGCGCCTGCTCATCGAGACCAGTGTT
FDC1083         CAACACGCACTCAAGCGACTCAAGCGCATTCCACAGCGCCTGCTCATCGAGACCAGTGTT
JF90-2          CAACACGCACTCAAGCGACTCAAGCGCATTCCACAGCGCCTGCTCATCGAGACCAGTGTT
JJ10-1          CAACACGCACTCAAGCGACTCAAGCGCATTCCACAGCGCCTGCTCATCGAGACCAGTGTT
JJ238-10        CAACACGCACTCAAGCGACTCAAGCGCATTCCACAGCGCCTGCTCATCGAGACCAGTGTT
JJ238-24        CAACACGCACTCAAGCGACTCAAGCGCATTCCACAGCGCCTGCTCATCGAGACCAGTGTT
JK2-10          CAACACGCACTCAAGCGACTCAAGCGCATTCCACAGCGCCTGCTCATCGAGACCAGTGTT
JK4-1           CAACACGCACTCAAGCGACTCAAGCGCATTCCACAGCGCCTGCTCATCGAGACCAGTGTT
JK48            CAACACGCACTCAAGCGACTCAAGCGCATTCCACAGCGCCTGCTCATCGAGACCAGTGTT
JK143-9         CAACACGCACTCAAGCGACTCAAGCGCATTCCACAGCGCCTGCTCATCGAGACCAGTGTT
JK143-11        CAACACGCACTCAAGCGACTCAAGCGCATTCCACAGCGCCTGCTCATCGAGACCAGTGTT
JM35            CAACACGCACTCAAGCGACTCAAGCGCATTCCACAGCGCCTGCTCATCGAGACCAGTGTT
JS581           CAACACGCACTCAAGCGACTCAAGCGCATTCCACAGCGCCTGCTCATCGAGACCAGTGTT
JS582           CAACACGCACTCAAGCGACTCAAGCGCATTCCACAGCGCCTGCTCATCGAGACCAGTGTT
JS584           CAACACGCACTCAAGCGACTCAAGCGCATTCCACAGCGCCTGCTCATCGAGACCAGTGTT
JW160-1         CAACACGCACTCAAGCGACTCAAGCGCATTCCACAGCGCCTGCTCATCGAGACCAGTGTT
LB100-1         CAACACGCACTCAAGCGACTCAAGCGCATTCCACAGCGCCTGCTCATCGAGACCAGTGTT
LC80            CAACACGCACTCAAGCGACTCAAGCGCATTCCACAGCGCCTGCTCATCGAGACCAGTGTT
LD7-1           CAACACGCACTCAAGCGACTCAAGCGCATTCCACAGCGCCTGCTCATCGAGACCAGTGTT
LD71a           CAACACGCACTCAAGCGACTCAAGCGCATTCCACAGCGCCTGCTCATCGAGACCAGTGTT
LE3-1           CAACACGCACTCAAGCGACTCAAGCGCATTCCACAGCGCCTGCTCATCGAGACCAGTGTT
LE20-1          CAACACGCACTCAAGCGACTCAAGCGCATTCCACAGCGCCTGCTCATCGAGACCAGTGTT
LE116-1         CAACACGCACTCAAGCGACTCAAGCGCATTCCACAGCGCCTGCTCATCGAGACCAGTGTT
LG97            CAACACGCACTCAAGCGACTCAAGCGCATTCCACAGCGCCTGCTCATCGAGACCAGTGTT
LG102           CAACACGCACTCAAGCGACTCAAGCGCATTCCACAGCGCCTGCTCATCGAGACCAGTGTT
LG117           CAACACGCACTCAAGCGACTCAAGCGCATTCCACAGCGCCTGCTCATCGAGACCAGTGTT
LH37-1          CAACACGCACTCAAGCGACTCAAGCGCATTCCACAGCGCCTGCTCATCGAGACCAGTGTT
LMG9322         CAACACGCACTCAAGCGACTCAAGCGCATTCCACAGCGCCTGCTCATCGAGACCAGTGTT
NCPPB3562       CAACACGCACTCAAGCGACTCAAGCGCATTCCACAGCGCCTGCTCATCGAGACCAGTGTT
NCPPB3607       CAACACGCACTCAAGCGACTCAAGCGCATTCCACAGCGCCTGCTCATCGAGACCAGTGTT
NCPPB3610       CAACACGCACTCAAGCGACTCAAGCGCATTCCACAGCGCCTGCTCATCGAGACCAGTGTT
NCPPB3612       CAACACGCACTCAAGCGACTCAAGCGCATTCCACAGCGCCTGCTCATCGAGACCAGTGTT
NCPPB3615       CAACACGCACTCAAGCGACTCAAGCGCATTCCACAGCGCCTGCTCATCGAGACCAGTGTT
LB302           CAACACGCACTCAAGCGACTCAAGCGCATTCCACAGCGCCTGCTCATCGAGACCAGTGTT
LG115           CAACACGCACTCAAGCGACTCAAGCGCATTCCACAGCGCCTGCTCATCGAGACCAGTGTT
NCPPB3608       CAACACGCACTCAAGCGACTCAAGCGCATTCCACAGCGCCTGCTCATCGAGACCAGTGTT
X2003-3218      CAACACGCACTCAAGCGACTCAAGCGCATTCCACAGCGCCTGCTCATCGAGACCAGTGTT
                ************************************************************
```

```
JF90-8          GACGTGCTGCGTGGCCTGGAAGGCGAGGCAGCGCAGTCGTATTTCGGCGTCTTCGGCCAG
C40             GACGTGCTGCGTGGCCTGGAAGGCGAGGCAGCGCAGTCGTATTTCGGCGTCTTCGGCCAG
CFBP2852        GACGTGCTGCGTGGCCTGGAAGGCGAGGCAGCGCAGTCGTATTTCGGCGTCTTCGGCCAG
CFBP2911        GACGTGCTGCGTGGCCTGGAAGGCGAGGCAGCGCAGTCGTATTTCGGCGTCTTCGGCCAG
FDC217          GACGTGCTGCGTGGCCTGGAAGGCGAGGCAGCGCAGTCGTATTTCGGCGTCTTCGGCCAG
FDC1083         GACGTGCTGCGTGGCCTGGAAGGCGAGGCAGCGCAGTCGTATTTCGGCGTCTTCGGCCAG
JF90-2          GACGTGCTGCGTGGCCTGGAAGGCGAGGCAGCGCAGTCGTATTTCGGCGTCTTCGGCCAG
JJ10-1          GACGTGCTGCGTGGCCTGGAAGGCGAGGCAGCGCAGTCGTATTTCGGCGTCTTCGGCCAG
JJ238-10        GACGTGCTGCGTGGCCTGGAAGGCGAGGCAGCGCAGTCGTATTTCGGCGTCTTCGGCCAG
JJ238-24        GACGTGCTGCGTGGCCTGGAAGGCGAGGCAGCGCAGTCGTATTTCGGCGTCTTCGGCCAG
JK2-10          GACGTGCTGCGTGGCCTGGAAGGCGAGGCAGCGCAGTCGTATTTCGGCGTCTTCGGCCAG
JK4-1           GACGTGCTGCGTGGCCTGGAAGGCGAGGCAGCGCAGTCGTATTTCGGCGTCTTCGGCCAG
JK48            GACGTGCTGCGTGGCCTGGAAGGCGAGGCAGCGCAGTCGTATTTCGGCGTCTTCGGCCAG
JK143-9         GACGTGCTGCGTGGCCTGGAAGGCGAGGCAGCGCAGTCGTATTTCGGCGTCTTCGGCCAG
JK143-11        GACGTGCTGCGTGGCCTGGAAGGCGAGGCAGCGCAGTCGTATTTCGGCGTCTTCGGCCAG
JM35            GACGTGCTGCGTGGCCTGGAAGGCGAGGCAGCGCAGTCGTATTTCGGCGTCTTCGGCCAG
JS581           GACGTGCTGCGTGGCCTGGAAGGCGAGGCAGCGCAGTCGTATTTCGGCGTCTTCGGCCAG
JS582           GACGTGCTGCGTGGCCTGGAAGGCGAGGCAGCGCAGTCGTATTTCGGCGTCTTCGGCCAG
JS584           GACGTGCTGCGTGGCCTGGAAGGCGAGGCAGCGCAGTCGTATTTCGGCGTCTTCGGCCAG
JW160-1         GACGTGCTGCGTGGCCTGGAAGGCGAGGCAGCGCAGTCGTATTTCGGCGTCTTCGGCCAG
LB100-1         GACGTGCTGCGTGGCCTGGAAGGCGAGGCAGCGCAGTCGTATTTCGGCGTCTTCGGCCAG
LC80            GACGTGCTGCGTGGCCTGGAAGGCGAGGCAGCGCAGTCGTATTTCGGCGTCTTCGGCCAG
LD7-1           GACGTGCTGCGTGGCCTGGAAGGCGAGGCAGCGCAGTCGTATTTCGGCGTCTTCGGCCAG
LD71a           GACGTGCTGCGTGGCCTGGAAGGCGAGGCAGCGCAGTCGTATTTCGGCGTCTTCGGCCAG
LE3-1           GACGTGCTGCGTGGCCTGGAAGGCGAGGCAGCGCAGTCGTATTTCGGCGTCTTCGGCCAG
LE20-1          GACGTGCTGCGTGGCCTGGAAGGCGAGGCAGCGCAGTCGTATTTCGGCGTCTTCGGCCAG
LE116-1         GACGTGCTGCGTGGCCTGGAAGGCGAGGCAGCGCAGTCGTATTTCGGCGTCTTCGGCCAG
LG97            GACGTGCTGCGTGGCCTGGAAGGCGAGGCAGCGCAGTCGTATTTCGGCGTCTTCGGCCAG
LG102           GACGTGCTGCGTGGCCTGGAAGGCGAGGCAGCGCAGTCGTATTTCGGCGTCTTCGGCCAG
LG117           GACGTGCTGCGTGGCCTGGAAGGCGAGGCAGCGCAGTCGTATTTCGGCGTCTTCGGCCAG
LH37-1          GACGTGCTGCGTGGCCTGGAAGGCGAGGCAGCGCAGTCGTATTTCGGCGTCTTCGGCCAG
LMG9322         GACGTGCTGCGTGGCCTGGAAGGCGAGGCAGCGCAGTCGTATTTCGGCGTCTTCGGCCAG
NCPPB3562       GACGTGCTGCGTGGCCTGGAAGGCGAGGCAGCGCAGTCGTATTTCGGCGTCTTCGGCCAG
NCPPB3607       GACGTGCTGCGTGGCCTGGAAGGCGAGGCAGCGCAGTCGTATTTCGGCGTCTTCGGCCAG
NCPPB3610       GACGTGCTGCGTGGCCTGGAAGGCGAGGCAGCGCAGTCGTATTTCGGCGTCTTCGGCCAG
NCPPB3612       GACGTGCTGCGTGGCCTGGAAGGCGAGGCAGCGCAGTCGTATTTCGGCGTCTTCGGCCAG
NCPPB3615       GACGTGCTGCGTGGCCTGGAAGGCGAGGCAGCGCAGTCGTATTTCGGCGTCTTCGGCCAG
LB302           GACGTGCTGCGTGGCCTGGAAGGCGAGGCAGCGCAGTCGTATTTCGGCGTCTTCGGCCAG
LG115           GACGTGCTGCGTGGCCTGGAAGGCGAGGCAGCGCAGTCGTATTTCGGCGTCTTCGGCCAG
NCPPB3608       GACGTGCTGCGTGGCCTGGAAGGCGAGGCAGCGCAGTCGTATTTCGGCGTCTTCGGCCAG
X2003-3218      GACGTGCTGCGTGGCCTGGAAGGCGAGGCAGCGCAGTCGTATTTCGGCGTCTTCGGCCAG
                ************************************************************

JF90-8          CTGGTGCGCACCGAGTCGCTGCTGCTGCGCTTTGGCGGACGCAATCGCCGCCCACCGCGC
C40             CTGGTGCGCACCGAGTCGCCGCTGCTGCGCTTTGGCGGACGCAATCGCCGCCCACCGCGC
CFBP2852        CTGGTGCGCACCGAGTCGCCGCTGCTGCGCTTTGGCGGACGCAATCGCCGCCCACCGCGC
CFBP2911        CTGGTGCGCACCGAGTCGCCGCTGCTGCGCTTTGGCGGACGCAATCGCCGCCCACCGCGC
FDC217          CTGGTGCGCACCGAGTCGCCGCTGCTGCGCTTTGGCGGACGCAATCGCCGCCCACCGCGC
FDC1083         CTGGTGCGCACCGAGTCGCCGCTGCTGCGCTTTGGCGGACGCAATCGCCGCCCACCGCGC
JF90-2          CTGGTGCGCACCGAGTCGCCGCTGCTGCGCTTTGGCGGACGCAATCGCCGCCCACCGCGC
JJ10-1          CTGGTGCGCACCGAGTCGCCGCTGCTGCGCTTTGGCGGACGCAATCGCCGCCCACCGCGC
JJ238-10        CTGGTGCGCACCGAGTCGCCGCTGCTGCGCTTTGGCGGACGCAATCGCCGCCCACCGCGC
JJ238-24        CTGGTGCGCACCGAGTCGCCGCTGCTGCGCTTTGGCGGACGCAATCGCCGCCCACCGCGC
JK2-10          CTGGTGCGCACCGAGTCGCCGCTGCTGCGCTTTGGCGGACGCAATCGCCGCCCACCGCGC
JK4-1           CTGGTGCGCACCGAGTCGCCGCTGCTGCGCTTTGGCGGACGCAATCGCCGCCCACCGCGC
JK48            CTGGTGCGCACCGAGTCGCCGCTGCTGCGCTTTGGCGGACGCAATCGCCGCCCACCGCGC
JK143-9         CTGGTGCGCACCGAGTCGCCGCTGCTGCGCTTTGGCGGACGCAATCGCCGCCCACCGCGC
JK143-11        CTGGTGCGCACCGAGTCGCCGCTGCTGCGCTTTGGCGGACGCAATCGCCGCCCACCGCGC
JM35            CTGGTGCGCACCGAGTCGCCGCTGCTGCGCTTTGGCGGACGCAATCGCCGCCCACCGCGC
JS581           CTGGTGCGCACCGAGTCGCCGCTGCTGCGCTTTGGCGGACGCAATCGCCGCCCACCGCGC
JS582           CTGGTGCGCACCGAGTCGCCGCTGCTGCGCTTTGGCGGACGCAATCGCCGCCCACCGCGC
JS584           CTGGTGCGCACCGAGTCGCCGCTGCTGCGCTTTGGCGGACGCAATCGCCGCCCACCGCGC
JW160-1         CTGGTGCGCACCGAGTCGCCGCTGCTGCGCTTTGGCGGACGCAATCGCCGCCCACCGCGC
LB100-1         CTGGTGCGCACCGAGTCGCCGCTGCTGCGCTTTGGCGGACGCAATCGCCGCCCACCGCGC
LC80            CTGGTGCGCACCGAGTCGCCGCTGCTGCGCTTTGGCGGACGCAATCGCCGCCCACCGCGC
LD7-1           CTGGTGCGCACCGAGTCGCCGCTGCTGCGCTTTGGCGGACGCAATCGCCGCCCACCGCGC
LD71a           CTGGTGCGCACCGAGTCGCCGCTGCTGCGCTTTGGCGGACGCAATCGCCGCCCACCGCGC
LE3-1           CTGGTGCGCACCGAGTCGCCGCTGCTGCGCTTTGGCGGACGCAATCGCCGCCCACCGCGC
LE20-1          CTGGTGCGCACCGAGTCGCCGCTGCTGCGCTTTGGCGGACGCAATCGCCGCCCACCGCGC
LE116-1         CTGGTGCGCACCGAGTCGCCGCTGCTGCGCTTTGGCGGACGCAATCGCCGCCCACCGCGC
LG97            CTGGTGCGCACCGAGTCGCCGCTGCTGCGCTTTGGCGGACGCAATCGCCGCCCACCGCGC
LG102           CTGGTGCGCACCGAGTCGCCGCTGCTGCGCTTTGGCGGACGCAATCGCCGCCCACCGCGC
LG117           CTGGTGCGCACCGAGTCGCCGCTGCTGCGCTTTGGCGGACGCAATCGCCGCCCACCGCGC
LH37-1          CTGGTGCGCACCGAGTCGCCGCTGCTGCGCTTTGGCGGACGCAATCGCCGCCCACCGCGC
LMG9322         CTGGTGCGCACCGAGTCGCCGCTGCTGCGCTTTGGCGGACGCAATCGCCGCCCACCGCGC
NCPPB3562       CTGGTGCGCACCGAGTCGCCGCTGCTGCGCTTTGGCGGACGCAATCGCCGCCCACCGCGC
NCPPB3607       CTGGTGCGCACCGAGTCGCCGCTGCTGCGCTTTGGCGGACGCAATCGCCGCCCACCGCGC
NCPPB3610       CTGGTGCGCACCGAGTCGCCGCTGCTGCGCTTTGGCGGACGCAATCGCCGCCCACCGCGC
NCPPB3612       CTGGTGCGCACCGAGTCGCCGCTGCTGCGCTTTGGCGGACGCAATCGCCGCCCACCGCGC
NCPPB3615       CTGGTGCGCACCGAGTCGCCGCTGCTGCGCTTTGGCGGACGCAATCGCCGCCCACCGCGC
LB302           CTGGTGCGCACCGAGTCGCCGCTGCTGCGCTTTGGCGGACGCAATCGCCGCCCACCGCGC
LG115           CTGGTGCGCACCGAGTCGCCGCTGCTGCGCTTTGGCGGACGCAATCGCCGCCCACCGCGC
NCPPB3608       CTGGTGCGCACCGAGTCGCCGCTGCTGCGCTTTGGCGGACGCAATCGCCGCCCACCGCGC
X2003-3218      CTGGTGCGCACCGAGTCGCCGCTGCTGCGCTTTGGCGGACGCAATCGCCGCCCACCGCGC
                ******************* ****************************************
```

```
JF90-8          GACGCCTTCAATGCGTTGCTGTCGTTTCTCTACACCTTGCTGACCCACGACTGCCGTTCG
C40             GACGCCTTCAATGCGTTGCTGTCGTTTCTCTACACCTTGCTGACCCACGACTGCCGTTCG
CFBP2852        GACGCCTTCAATGCGTTGCTGTCGTTTCTCTACACCTTGCTGACCCACGACTGCCGTTCG
CFBP2911        GACGCCTTCAATGCGTTGCTGTCGTTTCTCTACACCTTGCTGACCCACGACTGCCGTTCG
FDC217          GACGCCTTCAATGCGTTGCTGTCGTTTCTCTACACCTTGCTGACCCACGACTGCCGTTCG
FDC1083         GACGCCTTCAATGCGTTGCTGTCGTTTCTCTACACCTTGCTGACCCACGACTGCCGTTCG
JF90-2          GACGCCTTCAATGCGTTGCTGTCGTTTCTCTACACCTTGCTGACCCACGACTGCCGTTCG
JJ10-1          GACGCCTTCAATGCGTTGCTGTCGTTTCTCTACACCTTGCTGACCCACGACTGCCGTTCG
JJ238-10        GACGCCTTCAATGCGTTGCTGTCGTTTCTCTACACCTTGCTGACCCACGACTGCCGTTCG
JJ238-24        GACGCCTTCAATGCGTTGCTGTCGTTTCTCTACACCTTGCTGACCCACGACTGCCGTTCG
JK2-10          GACGCCTTCAATGCGTTGCTGTCGTTTCTCTACACCTTGCTGACCCACGACTGCCGTTCG
JK4-1           GACGCCTTCAATGCGTTGCTGTCGTTTCTCTACACCTTGCTGACCCACGACTGCCGTTCG
JK48            GACGCCTTCAATGCGTTGCTGTCGTTTCTCTACACCTTGCTGACCCACGACTGCCGTTCG
JK143-9         GACGCCTTCAATGCGTTGCTGTCGTTTCTCTACACCTTGCTGACCCACGACTGCCGTTCG
JK143-11        GACGCCTTCAATGCGTTGCTGTCGTTTCTCTACACCTTGCTGACCCACGACTGCCGTTCG
JM35            GACGCCTTCAATGCGTTGCTGTCGTTTCTCTACACCTTGCTGACCCACGACTGCCGTTCG
JS581           GACGCCTTCAATGCGTTGCTGTCGTTTCTCTACACCTTGCTGACCCACGACTGCCGTTCG
JS582           GACGCCTTCAATGCGTTGCTGTCGTTTCTCTACACCTTGCTGACCCACGACTGCCGTTCG
JS584           GACGCCTTCAATGCGTTGCTGTCGTTTCTCTACACCTTGCTGACCCACGACTGCCGTTCG
JW160-1         GACGCCTTCAATGCGTTGCTGTCGTTTCTCTACACCTTGCTGACCCACGACTGCCGTTCG
LB100-1         GACGCCTTCAATGCGTTGCTGTCGTTTCTCTACACCTTGCTGACCCACGACTGCCGTTCG
LC80            GACGCCTTCAATGCGTTGCTGTCGTTTCTCTACACCTTGCTGACCCACGACTGCCGTTCG
LD7-1           GACGCCTTCAATGCGTTGCTGTCGTTTCTCTACACCTTGCTGACCCACGACTGCCGTTCG
LD71a           GACGCCTTCAATGCGTTGCTGTCGTTTCTCTACACCTTGCTGACCCACGACTGCCGTTCG
LE3-1           GACGCCTTCAATGCGTTGCTGTCGTTTCTCTACACCTTGCTGACCCACGACTGCCGTTCG
LE20-1          GACGCCTTCAATGCGTTGCTGTCGTTTCTCTACACCTTGCTGACCCACGACTGCCGTTCG
LE116-1         GACGCCTTCAATGCGTTGCTGTCGTTTCTCTACACCTTGCTGACCCACGACTGCCGTTCG
LG97            GACGCCTTCAATGCGTTGCTGTCGTTTCTCTACACCTTGCTGACCCACGACTGCCGTTCG
LG102           GACGCCTTCAATGCGTTGCTGTCGTTTCTCTACACCTTGCTGACCCACGACTGCCGTTCG
LG117           GACGCCTTCAATGCGTTGCTGTCGTTTCTCTACACCTTGCTGACCCACGACTGCCGTTCG
LH37-1          GACGCCTTCAATGCGTTGCTGTCGTTTCTCTACACCTTGCTGACCCACGACTGCCGTTCG
LMG9322         GACGCCTTCAATGCGTTGCTGTCGTTTCTCTACACCTTGCTGACCCACGACTGCCGTTCG
NCPPB3562       GACGCCTTCAATGCGTTGCTGTCGTTTCTCTACACCTTGCTGACCCACGACTGCCGTTCG
NCPPB3607       GACGCCTTCAATGCGTTGCTGTCGTTTCTCTACACCTTGCTGACCCACGACTGCCGTTCG
NCPPB3610       GACGCCTTCAATGCGTTGCTGTCGTTTCTCTACACCTTGCTGACCCACGACTGCCGTTCG
NCPPB3612       GACGCCTTCAATGCGTTGCTGTCGTTTCTCTACACCTTGCTGACCCACGACTGCCGTTCG
NCPPB3615       GACGCCTTCAATGCGTTGCTGTCGTTTCTCTACACCTTGCTGACCCACGACTGCCGTTCG
LB302           GACGCCTTCAATGCGTTGCTGTCGTTTCTCTACACCTTGCTGACCCACGACTGCCGTTCG
LG115           GACGCCTTCAATGCGTTGCTGTCGTTTCTCTACACCTTGCTGACCCACGACTGCCGTTCG
NCPPB3608       GACGCCTTCAATGCGTTGCTGTCGTTTCTCTACACCTTGCTGACCCACGACTGCCGTTCG
X2003-3218      GACGCCTTCAATGCGTTGCTGTCGTTTCTCTACACCTTGCTGACCCACGACTGCCGTTCG
                ************************************************************

JF90-8          GCCTTGGAAACGGTTGGTCTGGACCCGGCAGTCGGCTTTCTGCATCGCGACCGACCAGGA
C40             GCCTTGGAAACGGTTGGTCTGGACCCGGCAGTCGGCTTTCTGCATCGCGACCGACCAGGA
CFBP2852        GCCTTGGAAACGGTTGGTCTGGACCCGGCAGTCGGCTTTCTGCATCGCGACCGACCAGGA
CFBP2911        GCCTTGGAAACGGTTGGTCTGGACCCGGCAGTCGGCTTTCTGCATCGCGACCGACCAGGA
FDC217          GCCTTGGAAACGGTTGGTCTGGACCCGGCAGTCGGCTTTCTGCATCGCGACCGACCAGGA
FDC1083         GCCTTGGAAACGGTTGGTCTGGACCCGGCAGTCGGCTTTCTGCATCGCGACCGACCAGGA
JF90-2          GCCTTGGAAACGGTTGGTCTGGACCCGGCAGTCGGCTTTCTGCATCGCGACCGACCAGGA
JJ10-1          GCCTTGGAAACGGTTGGTCTGGACCCGGCAGTCGGCTTTCTGCATCGCGACCGACCAGGA
JJ238-10        GCCTTGGAAACGGTTGGTCTGGACCCGGCAGTCGGCTTTCTGCATCGCGACCGACCAGGA
JJ238-24        GCCTTGGAAACGGTTGGTCTGGACCCGGCAGTCGGCTTTCTGCATCGCGACCGACCAGGA
JK2-10          GCCTTGGAAACGGTTGGTCTGGACCCGGCAGTCGGCTTTCTGCATCGCGACCGACCAGGA
JK4-1           GCCTTGGAAACGGTTGGTCTGGACCCGGCAGTCGGCTTTCTGCATCGCGACCGACCAGGA
JK48            GCCTTGGAAACGGTTGGTCTGGACCCGGCAGTCGGCTTTCTGCATCGCGACCGACCAGGA
JK143-9         GCCTTGGAAACGGTTGGTCTGGACCCGGCAGTCGGCTTTCTGCATCGCGACCGACCAGGA
JK143-11        GCCTTGGAAACGGTTGGTCTGGACCCGGCAGTCGGCTTTCTGCATCGCGACCGACCAGGA
JM35            GCCTTGGAAACGGTTGGTCTGGACCCGGCAGTCGGCTTTCTGCATCGCGACCGACCAGGA
JS581           GCCTTGGAAACGGTTGGTCTGGACCCGGCAGTCGGCTTTCTGCATCGCGACCGACCAGGA
JS582           GCCTTGGAAACGGTTGGTCTGGACCCGGCAGTCGGCTTTCTGCATCGCGACCGACCAGGA
JS584           GCCTTGGAAACGGTTGGTCTGGACCCGGCAGTCGGCTTTCTGCATCGCGACCGACCAGGA
JW160-1         GCCTTGGAAACGGTTGGTCTGGACCCGGCAGTCGGCTTTCTGCATCGCGACCGACCAGGA
LB100-1         GCCTTGGAAACGGTTGGTCTGGACCCGGCAGTCGGCTTTCTGCATCGCGACCGACCAGGA
LC80            GCCTTGGAAACGGTTGGTCTGGACCCGGCAGTCGGCTTTCTGCATCGCGACCGACCAGGA
LD7-1           GCCTTGGAAACGGTTGGTCTGGACCCGGCAGTCGGCTTTCTGCATCGCGACCGACCAGGA
LD71a           GCCTTGGAAACGGTTGGTCTGGACCCGGCAGTCGGCTTTCTGCATCGCGACCGACCAGGA
LE3-1           GCCTTGGAAACGGTTGGTCTGGACCCGGCAGTCGGCTTTCTGCATCGCGACCGACCAGGA
LE20-1          GCCTTGGAAACGGTTGGTCTGGACCCGGCAGTCGGCTTTCTGCATCGCGACCGACCAGGA
LE116-1         GCCTTGGAAACGGTTGGTCTGGACCCGGCAGTCGGCTTTCTGCATCGCGACCGACCAGGA
LG97            GCCTTGGAAACGGTTGGTCTGGACCCGGCAGTCGGCTTTCTGCATCGCGACCGACCAGGA
LG102           GCCTTGGAAACGGTTGGTCTGGACCCGGCAGTCGGCTTTCTGCATCGCGACCGACCAGGA
LG117           GCCTTGGAAACGGTTGGTCTGGACCCGGCAGTCGGCTTTCTGCATCGCGACCGACCAGGA
LH37-1          GCCTTGGAAACGGTTGGTCTGGACCCGGCAGTCGGCTTTCTGCATCGCGACCGACCAGGA
LMG9322         GCCTTGGAAACGGTTGGTCTGGACCCGGCAGTCGGCTTTCTGCATCGCGACCGACCAGGA
NCPPB3562       GCCTTGGAAACGGTTGGTCTGGACCCGGCAGTCGGCTTTCTGCATCGCGACCGACCAGGA
NCPPB3607       GCCTTGGAAACGGTTGGTCTGGACCCGGCAGTCGGCTTTCTGCATCGCGACCGACCAGGA
NCPPB3610       GCCTTGGAAACGGTTGGTCTGGACCCGGCAGTCGGCTTTCTGCATCGCGACCGACCAGGA
NCPPB3612       GCCTTGGAAACGGTTGGTCTGGACCCGGCAGTCGGCTTTCTGCATCGCGACCGACCAGGA
NCPPB3615       GCCTTGGAAACGGTTGGTCTGGACCCGGCAGTCGGCTTTCTGCATCGCGACCGACCAGGA
LB302           GCCTTGGAAACGGTTGGTCTGGACCCGGCAGTCGGCTTTCTGCATCGCGACCGACCAGGA
LG115           GCCTTGGAAACGGTTGGTCTGGACCCGGCAGTCGGCTTTCTGCATCGCGACCGACCAGGA
NCPPB3608       GCCTTGGAAACGGTTGGTCTGGACCCGGCAGTCGGCTTTCTGCATCGCGACCGACCAGGA
X2003-3218      GCCTTGGAAACGGTTGGTCTGGACCCGGCAGTCGGCTTTCTGCATCGCGACCGACCAGGA
                ************************************************************
```

```
JF90-8          CGGCCAAGCCTGGCCCTGGACCTTGCAGAGGAATTCAGGCCACTGCTTGGCGAGCGCCTG
C40             CGGCCAAGCCTGGCCCTGGACCTTGCAGAGGAATTCAGGCCACTGCTTGGCGAGCGCCTG
CFBP2852        CGGCCAAGCCTGGCCCTGGACCTTGCAGAGGAATTCAGGCCACTGCTTGGCGAGCGCCTG
CFBP2911        CGGCCAAGCCTGGCCCTGGACCTTGCAGAGGAATTCAGGCCACTGCTTGGCGAGCGCCTG
FDC217          CGGCCAAGCCTGGCCCTGGACCTTGCAGAGGAATTCAGGCCACTGCTTGGCGAGCGCCTG
FDC1083         CGGCCAAGCCTGGCCCTGGACCTTGCAGAGGAATTCAGGCCACTGCTTGGCGAGCGCCTG
JF90-2          CGGCCAAGCCTGGCCCTGGACCTTGCAGAGGAATTCAGGCCACTGCTTGGCGAGCGCCTG
JJ10-1          CGGCCAAGCCTGGCCCTGGACCTTGCAGAGGAATTCAGGCCACTGCTTGGCGAGCGCCTG
JJ238-10        CGGCCAAGCCTGGCCCTGGACCTTGCAGAGGAATTCAGGCCACTGCTTGGCGAGCGCCTG
JJ238-24        CGGCCAAGCCTGGCCCTGGACCTTGCAGAGGAATTCAGGCCACTGCTTGGCGAGCGCCTG
JK2-10          CGGCCAAGCCTGGCCCTGGACCTTGCAGAGGAATTCAGGCCACTGCTTGGCGAGCGCCTG
JK4-1           CGGCCAAGCCTGGCCCTGGACCTTGCAGAGGAATTCAGGCCACTGCTTGGCGAGCGCCTG
JK48            CGGCCAAGCCTGGCCCTGGACCTTGCAGAGGAATTCAGGCCACTGCTTGGCGAGCGCCTG
JK143-9         CGGCCAAGCCTGGCCCTGGACCTTGCAGAGGAATTCAGGCCACTGCTTGGCGAGCGCCTG
JK143-11        CGGCCAAGCCTGGCCCTGGACCTTGCAGAGGAATTCAGGCCACTGCTTGGCGAGCGCCTG
JM35            CGGCCAAGCCTGGCCCTGGACCTTGCAGAGGAATTCAGGCCACTGCTTGGCGAGCGCCTG
JS581           CGGCCAAGCCTGGCCCTGGACCTTGCAGAGGAATTCAGGCCACTGCTTGGCGAGCGCCTG
JS582           CGGCCAAGCCTGGCCCTGGACCTTGCAGAGGAATTCAGGCCACTGCTTGGCGAGCGCCTG
JS584           CGGCCAAGCCTGGCCCTGGACCTTGCAGAGGAATTCAGGCCACTGCTTGGCGAGCGCCTG
JW160-1         CGGCCAAGCCTGGCCCTGGACCTTGCAGAGGAATTCAGGCCACTGCTTGGCGAGCGCCTG
LB100-1         CGGCCAAGCCTGGCCCTGGACCTTGCAGAGGAATTCAGGCCACTGCTTGGCGAGCGCCTG
LC80            CGGCCAAGCCTGGCCCTGGACCTTGCAGAGGAATTCAGGCCACTGCTTGGCGAGCGCCTG
LD7-1           CGGCCAAGCCTGGCCCTGGACCTTGCAGAGGAATTCAGGCCACTGCTTGGCGAGCGCCTG
LD71a           CGGCCAAGCCTGGCCCTGGACCTTGCAGAGGAATTCAGGCCACTGCTTGGCGAGCGCCTG
LE3-1           CGGCCAAGCCTGGCCCTGGACCTTGCAGAGGAATTCAGGCCACTGCTTGGCGAGCGCCTG
LE20-1          CGGCCAAGCCTGGCCCTGGACCTTGCAGAGGAATTCAGGCCACTGCTTGGCGAGCGCCTG
LE116-1         CGGCCAAGCCTGGCCCTGGACCTTGCAGAGGAATTCAGGCCACTGCTTGGCGAGCGCCTG
LG97            CGGCCAAGCCTGGCCCTGGACCTTGCAGAGGAATTCAGGCCACTGCTTGGCGAGCGCCTG
LG102           CGGCCAAGCCTGGCCCTGGACCTTGCAGAGGAATTCAGGCCACTGCTTGGCGAGCGCCTG
LG117           CGGCCAAGCCTGGCCCTGGACCTTGCAGAGGAATTCAGGCCACTGCTTGGCGAGCGCCTG
LH37-1          CGGCCAAGCCTGGCCCTGGACCTTGCAGAGGAATTCAGGCCACTGCTTGGCGAGCGCCTG
LMG9322         CGGCCAAGCCTGGCCCTGGACCTTGCAGAGGAATTCAGGCCACTGCTTGGCGAGCGCCTG
NCPPB3562       CGGCCAAGCCTGGCCCTGGACCTTGCAGAGGAATTCAGGCCACTGCTTGGCGAGCGCCTG
NCPPB3607       CGGCCAAGCCTGGCCCTGGACCTTGCAGAGGAATTCAGGCCACTGCTTGGCGAGCGCCTG
NCPPB3610       CGGCCAAGCCTGGCCCTGGACCTTGCAGAGGAATTCAGGCCACTGCTTGGCGAGCGCCTG
NCPPB3612       CGGCCAAGCCTGGCCCTGGACCTTGCAGAGGAATTCAGGCCACTGCTTGGCGAGCGCCTG
NCPPB3615       CGGCCAAGCCTGGCCCTGGACCTTGCAGAGGAATTCAGGCCACTGCTTGGCGAGCGCCTG
LB302           CGGCCAAGCCTGGCCCTGGACCTTGCAGAGGAATTCAGGCCACTGCTTGGCGAGCGCCTG
LG115           CGGCCAAGCCTGGCCCTGGACCTTGCAGAGGAATTCAGGCCACTGCTTGGCGAGCGCCTG
NCPPB3608       CGGCCAAGCCTGGCCCTGGACCTTGCAGAGGAATTCAGGCCACTGCTTGGCGAGCGCCTG
X2003-3218      CGGCCAAGCCTGGCCCTGGACCTTGCAGAGGAATTCAGGCCACTGCTTGGCGAGCGCCTG
                ************************************************************

JF90-8          GCACTGTCGCTGATCAACCGCCGGCAATTGAACCAGCGCGACTTCCAAGTCTTCGACAAC
C40             GCACTGTCGCTGATCAACCGCCGGCAATTGAACCAGCGCGACTTCCAAGTCTTCGACAAC
CFBP2852        GCACTGTCGCTGATCAACCGCCGGCAATTGAACCAGCGCGACTTCCAAGTCTTCGACAAC
CFBP2911        GCACTGTCGCTGATCAACCGCCGGCAATTGAACCAGCGCGACTTCCAAGTCTTCGACAAC
FDC217          GCACTGTCGCTGATCAACCGCCGGCAATTGAACCAGCGCGACTTCCAAGTCTTCGACAAC
FDC1083         GCACTGTCGCTGATCAACCGCCGGCAATTGAACCAGCGCGACTTCCAAGTCTTCGACAAC
JF90-2          GCACTGTCGCTGATCAACCGCCGGCAATTGAACCAGCGCGACTTCCAAGTCTTCGACAAC
JJ10-1          GCACTGTCGCTGATCAACCGCCGGCAATTGAACCAGCGCGACTTCCAAGTCTTCGACAAC
JJ238-10        GCACTGTCGCTGATCAACCGCCGGCAATTGAACCAGCGCGACTTCCAAGTCTTCGACAAC
JJ238-24        GCACTGTCGCTGATCAACCGCCGGCAATTGAACCAGCGCGACTTCCAAGTCTTCGACAAC
JK2-10          GCACTGTCGCTGATCAACCGCCGGCAATTGAACCAGCGCGACTTCCAAGTCTTCGACAAC
JK4-1           GCACTGTCGCTGATCAACCGCCGGCAATTGAACCAGCGCGACTTCCAAGTCTTCGACAAC
JK48            GCACTGTCGCTGATCAACCGCCGGCAATTGAACCAGCGCGACTTCCAAGTCTTCGACAAC
JK143-9         GCACTGTCGCTGATCAACCGCCGGCAATTGAACCAGCGCGACTTCCAAGTCTTCGACAAC
JK143-11        GCACTGTCGCTGATCAACCGCCGGCAATTGAACCAGCGCGACTTCCAAGTCTTCGACAAC
JM35            GCACTGTCGCTGATCAACCGCCGGCAATTGAACCAGCGCGACTTCCAAGTCTTCGACAAC
JS581           GCACTGTCGCTGATCAACCGCCGGCAATTGAACCAGCGCGACTTCCAAGTCTTCGACAAC
JS582           GCACTGTCGCTGATCAACCGCCGGCAATTGAACCAGCGCGACTTCCAAGTCTTCGACAAC
JS584           GCACTGTCGCTGATCAACCGCCGGCAATTGAACCAGCGCGACTTCCAAGTCTTCGACAAC
JW160-1         GCACTGTCGCTGATCAACCGCCGGCAATTGAACCAGCGCGACTTCCAAGTCTTCGACAAC
LB100-1         GCACTGTCGCTGATCAACCGCCGGCAATTGAACCAGCGCGACTTCCAAGTCTTCGACAAC
LC80            GCACTGTCGCTGATCAACCGCCGGCAATTGAACCAGCGCGACTTCCAAGTCTTCGACAAC
LD7-1           GCACTGTCGCTGATCAACCGCCGGCAATTGAACCAGCGCGACTTCCAAGTCTTCGACAAC
LD71a           GCACTGTCGCTGATCAACCGCCGGCAATTGAACCAGCGCGACTTCCAAGTCTTCGACAAC
LE3-1           GCACTGTCGCTGATCAACCGCCGGCAATTGAACCAGCGCGACTTCCAAGTCTTCGACAAC
LE20-1          GCACTGTCGCTGATCAACCGCCGGCAATTGAACCAGCGCGACTTCCAAGTCTTCGACAAC
LE116-1         GCACTGTCGCTGATCAACCGCCGGCAATTGAACCAGCGCGACTTCCAAGTCTTCGACAAC
LG97            GCACTGTCGCTGATCAACCGCCGGCAATTGAACCAGCGCGACTTCCAAGTCTTCGACAAC
LG102           GCACTGTCGCTGATCAACCGCCGGCAATTGAACCAGCGCGACTTCCAAGTCTTCGACAAC
LG117           GCACTGTCGCTGATCAACCGCCGGCAATTGAACCAGCGCGACTTCCAAGTCTTCGACAAC
LH37-1          GCACTGTCGCTGATCAACCGCCGGCAATTGAACCAGCGCGACTTCCAAGTCTTCGACAAC
LMG9322         GCACTGTCGCTGATCAACCGCCGGCAATTGAACCAGCGCGACTTCCAAGTCTTCGACAAC
NCPPB3562       GCACTGTCGCTGATCAACCGCCGGCAATTGAACCAGCGCGACTTCCAAGTCTTCGACAAC
NCPPB3607       GCACTGTCGCTGATCAACCGCCGGCAATTGAACCAGCGCGACTTCCAAGTCTTCGACAAC
NCPPB3610       GCACTGTCGCTGATCAACCGCCGGCAATTGAACCAGCGCGACTTCCAAGTCTTCGACAAC
NCPPB3612       GCACTGTCGCTGATCAACCGCCGGCAATTGAACCAGCGCGACTTCCAAGTCTTCGACAAC
NCPPB3615       GCACTGTCGCTGATCAACCGCCGGCAATTGAACCAGCGCGACTTCCAAGTCTTCGACAAC
LB302           GCACTGTCGCTGATCAACCGCCGGCAATTGAACCAGCGCGACTTCCAAGTCTTCGACAAC
LG115           GCACTGTCGCTGATCAACCGCCGGCAATTGAACCAGCGCGACTTCCAAGTCTTCGACAAC
NCPPB3608       GCACTGTCGCTGATCAACCGCCGGCAATTGAACCAGCGCGACTTCCAAGTCTTCGACAAC
X2003-3218      GCACTGTCGCTGATCAACCGCCGGCAATTGAACCAGCGCGACTTCCAAGTCTTCGACAAC
                ************************************************************
```

```
JF90-8          GGCGCGGTTCTTCTCAAGGACGATGCGCGCAAGACGGTGCTGATCGCCTACCAGGAGCGC
C40             GGCGCGGTTCTTCTCAAGGACGATGCGCGCAAGACGGTGCTGATCGCCTACCAGGAGCGC
CFBP2852        GGCGCGGTTCTTCTCAAGGACGATGCGCGCAAGACGGTGCTGATCGCCTACCAGGAGCGC
CFBP2911        GGCGCGGTTCTTCTCAAGGACGATGCGCGCAAGACGGTGCTGATCGCCTACCAGGAGCGC
FDC217          GGCGCGGTTCTTCTCAAGGACGATGCGCGCAAGACGGTGCTGATCGCCTACCAGGAGCGC
FDC1083         GGCGCGGTTCTTCTCAAGGACGATGCGCGCAAGACGGTGCTGATCGCCTACCAGGAGCGC
JF90-2          GGCGCGGTTCTTCTCAAGGACGATGCGCGCAAGACGGTGCTGATCGCCTACCAGGAGCGC
JJ10-1          GGCGCGGTTCTTCTCAAGGACGATGCGCGCAAGACGGTGCTGATCGCCTACCAGGAGCGC
JJ238-10        GGCGCGGTTCTTCTCAAGGACGATGCGCGCAAGACGGTGCTGATCGCCTACCAGGAGCGC
JJ238-24        GGCGCGGTTCTTCTCAAGGACGATGCGCGCAAGACGGTGCTGATCGCCTACCAGGAGCGC
JK2-10          GGCGCGGTTCTTCTCAAGGACGATGCGCGCAAGACGGTGCTGATCGCCTACCAGGAGCGC
JK4-1           GGCGCGGTTCTTCTCAAGGACGATGCGCGCAAGACGGTGCTGATCGCCTACCAGGAGCGC
JK48            GGCGCGGTTCTTCTCAAGGACGATGCGCGCAAGACGGTGCTGATCGCCTACCAGGAGCGC
JK143-9         GGCGCGGTTCTTCTCAAGGACGATGCGCGCAAGACGGTGCTGATCGCCTACCAGGAGCGC
JK143-11        GGCGCGGTTCTTCTCAAGGACGATGCGCGCAAGACGGTGCTGATCGCCTACCAGGAGCGC
JM35            GGCGCGGTTCTTCTCAAGGACGATGCGCGCAAGACGGTGCTGATCGCCTACCAGGAGCGC
JS581           GGCGCGGTTCTTCTCAAGGACGATGCGCGCAAGACGGTGCTGATCGCCTACCAGGAGCGC
JS582           GGCGCGGTTCTTCTCAAGGACGATGCGCGCAAGACGGTGCTGATCGCCTACCAGGAGCGC
JS584           GGCGCGGTTCTTCTCAAGGACGATGCGCGCAAGACGGTGCTGATCGCCTACCAGGAGCGC
JW160-1         GGCGCGGTTCTTCTCAAGGACGATGCGCGCAAGACGGTGCTGATCGCCTACCAGGAGCGC
LB100-1         GGCGCGGTTCTTCTCAAGGACGATGCGCGCAAGACGGTGCTGATCGCCTACCAGGAGCGC
LC80            GGCGCGGTTCTTCTCAAGGACGATGCGCGCAAGACGGTGCTGATCGCCTACCAGGAGCGC
LD7-1           GGCGCGGTTCTTCTCAAGGACGATGCGCGCAAGACGGTGCTGATCGCCTACCAGGAGCGC
LD71a           GGCGCGGTTCTTCTCAAGGACGATGCGCGCAAGACGGTGCTGATCGCCTACCAGGAGCGC
LE3-1           GGCGCGGTTCTTCTCAAGGACGATGCGCGCAAGACGGTGCTGATCGCCTACCAGGAGCGC
LE20-1          GGCGCGGTTCTTCTCAAGGACGATGCGCGCAAGACGGTGCTGATCGCCTACCAGGAGCGC
LE116-1         GGCGCGGTTCTTCTCAAGGACGATGCGCGCAAGACGGTGCTGATCGCCTACCAGGAGCGC
LG97            GGCGCGGTTCTTCTCAAGGACGATGCGCGCAAGACGGTGCTGATCGCCTACCAGGAGCGC
LG102           GGCGCGGTTCTTCTCAAGGACGATGCGCGCAAGACGGTGCTGATCGCCTACCAGGAGCGC
LG117           GGCGCGGTTCTTCTCAAGGACGATGCGCGCAAGACGGTGCTGATCGCCTACCAGGAGCGC
LH37-1          GGCGCGGTTCTTCTCAAGGACGATGCGCGCAAGACGGTGCTGATCGCCTACCAGGAGCGC
LMG9322         GGCGCGGTTCTTCTCAAGGACGATGCGCGCAAGACGGTGCTGATCGCCTACCAGGAGCGC
NCPPB3562       GGCGCGGTTCTTCTCAAGGACGATGCGCGCAAGACGGTGCTGATCGCCTACCAGGAGCGC
NCPPB3607       GGCGCGGTTCTTCTCAAGGACGATGCGCGCAAGACGGTGCTGATCGCCTACCAGGAGCGC
NCPPB3610       GGCGCGGTTCTTCTCAAGGACGATGCGCGCAAGACGGTGCTGATCGCCTACCAGGAGCGC
NCPPB3612       GGCGCGGTTCTTCTCAAGGACGATGCGCGCAAGACGGTGCTGATCGCCTACCAGGAGCGC
NCPPB3615       GGCGCGGTTCTTCTCAAGGACGATGCGCGCAAGACGGTGCTGATCGCCTACCAGGAGCGC
LB302           GGCGCGGTTCTTCTCAAGGACGATGCGCGCAAGACGGTGCTGATCGCCTACCAGGAGCGC
LG115           GGCGCGGTTCTTCTCAAGGACGATGCGCGCAAGACGGTGCTGATCGCCTACCAGGAGCGC
NCPPB3608       GGCGCGGTTCTTCTCAAGGACGATGCGCGCAAGACGGTGCTGATCGCCTACCAGGAGCGC
X2003-3218      GGCGCGGTTCTTCTCAAGGACGATGCGCGCAAGACGGTGCTGATCGCCTACCAGGAGCGC
                ************************************************************

JF90-8          AAACGCGAACAGCTTCAACATGCCTTACTGGGTGAAAAGATCGACATCGGCCTACTGCCC
C40             AAACGCGAACAGCTTCAACATGCCTTACTGGGTGAAAAGATCGACATCGGCCTACTGCCC
CFBP2852        AAACGCGAACAGCTTCAACATGCCTTACTGGGTGAAAAGATCGACATCGGCCTACTGCCC
CFBP2911        AAACGCGAACAGCTTCAACATGCCTTACTGGGTGAAAAGATCGACATCGGCCTACTGCCC
FDC217          AAACGCGAACAGCTTCAACATGCCTTACTGGGTGAAAAGATCGACATCGGCCTACTGCCC
FDC1083         AAACGCGAACAGCTTCAACATGCCTTACTGGGTGAAAAGATCGACATCGGCCTACTGCCC
JF90-2          AAACGCGAACAGCTTCAACATGCCTTACTGGGTGAAAAGATCGACATCGGCCTACTGCCC
JJ10-1          AAACGCGAACAGCTTCAACATGCCTTACTGGGTGAAAAGATCGACATCGGCCTACTGCCC
JJ238-10        AAACGCGAACAGCTTCAACATGCCTTACTGGGTGAAAAGATCGACATCGGCCTACTGCCC
JJ238-24        AAACGCGAACAGCTTCAACATGCCTTACTGGGTGAAAAGATCGACATCGGCCTACTGCCC
JK2-10          AAACGCGAACAGCTTCAACATGCCTTACTGGGTGAAAAGATCGACATCGGCCTACTGCCC
JK4-1           AAACGCGAACAGCTTCAACATGCCTTACTGGGTGAAAAGATCGACATCGGCCTACTGCCC
JK48            AAACGCGAACAGCTTCAACATGCCTTACTGGGTGAAAAGATCGACATCGGCCTACTGCCC
JK143-9         AAACGCGAACAGCTTCAACATGCCTTACTGGGTGAAAAGATCGACATCGGCCTACTGCCC
JK143-11        AAACGCGAACAGCTTCAACATGCCTTACTGGGTGAAAAGATCGACATCGGCCTACTGCCC
JM35            AAACGCGAACAGCTTCAACATGCCTTACTGGGTGAAAAGATCGACATCGGCCTACTGCCC
JS581           AAACGCGAACAGCTTCAACATGCCTTACTGGGTGAAAAGATCGACATCGGCCTACTGCCC
JS582           AAACGCGAACAGCTTCAACATGCCTTACTGGGTGAAAAGATCGACATCGGCCTACTGCCC
JS584           AAACGCGAACAGCTTCAACATGCCTTACTGGGTGAAAAGATCGACATCGGCCTACTGCCC
JW160-1         AAACGCGAACAGCTTCAACATGCCTTACTGGGTGAAAAGATCGACATCGGCCTACTGCCC
LB100-1         AAACGCGAACAGCTTCAACATGCCTTACTGGGTGAAAAGATCGACATCGGCCTACTGCCC
LC80            AAACGCGAACAGCTTCAACATGCCTTACTGGGTGAAAAGATCGACATCGGCCTACTGCCC
LD7-1           AAACGCGAACAGCTTCAACATGCCTTACTGGGTGAAAAGATCGACATCGGCCTACTGCCC
LD71a           AAACGCGAACAGCTTCAACATGCCTTACTGGGTGAAAAGATCGACATCGGCCTACTGCCC
LE3-1           AAACGCGAACAGCTTCAACATGCCTTACTGGGTGAAAAGATCGACATCGGCCTACTGCCC
LE20-1          AAACGCGAACAGCTTCAACATGCCTTACTGGGTGAAAAGATCGACATCGGCCTACTGCCC
LE116-1         AAACGCGAACAGCTTCAACATGCCTTACTGGGTGAAAAGATCGACATCGGCCTACTGCCC
LG97            AAACGCGAACAGCTTCAACATGCCTTACTGGGTGAAAAGATCGACATCGGCCTACTGCCC
LG102           AAACGCGAACAGCTTCAACATGCCTTACTGGGTGAAAAGATCGACATCGGCCTACTGCCC
LG117           AAACGCGAACAGCTTCAACATGCCTTACTGGGTGAAAAGATCGACATCGGCCTACTGCCC
LH37-1          AAACGCGAACAGCTTCAACATGCCTTACTGGGTGAAAAGATCGACATCGGCCTACTGCCC
LMG9322         AAACGCGAACAGCTTCAACATGCCTTACTGGGTGAAAAGATCGACATCGGCCTACTGCCC
NCPPB3562       AAACGCGAACAGCTTCAACATGCCTTACTGGGTGAAAAGATCGACATCGGCCTACTGCCC
NCPPB3607       AAACGCGAACAGCTTCAACATGCCTTACTGGGTGAAAAGATCGACATCGGCCTACTGCCC
NCPPB3610       AAACGCGAACAGCTTCAACATGCCTTACTGGGTGAAAAGATCGACATCGGCCTACTGCCC
NCPPB3612       AAACGCGAACAGCTTCAACATGCCTTACTGGGTGAAAAGATCGACATCGGCCTACTGCCC
NCPPB3615       AAACGCGAACAGCTTCAACATGCCTTACTGGGTGAAAAGATCGACATCGGCCTACTGCCC
LB302           AAACGCGAACAGCTTCAACATGCCTTACTGGGTGAAAAGATCGACATCGGCCTACTGCCC
LG115           AAACGCGAACAGCTTCAACATGCCTTACTGGGTGAAAAGATCGACATCGGCCTACTGCCC
NCPPB3608       AAACGCGAACAGCTTCAACATGCCTTACTGGGTGAAAAGATCGACATCGGCCTACTGCCC
X2003-3218      AAACGCGAACAGCTTCAACATGCCTTACTGGGTGAAAAGATCGACATCGGCCTACTGCCC
                ************************************************************
```

```
JF90-8          TTTGTGCAAGCACAGTTGATGGCACGGCACCTGCGTGGCGATCTGGATGGCTACCCCTCC
C40             TTTGTGCAAGCACAGTTGATGGCACGGCACCTGCGTGGCGATCTGGATGGCTACCCCTCC
CFBP2852        TTTGTGCAAGCACAGTTGATGGCACGGCACCTGCGTGGCGATCTGGATGGCTACCCCTCC
CFBP2911        TTTGTGCAAGCACAGTTGATGGCACGGCACCTGCGTGGCGATCTGGATGGCTACCCCTCC
FDC217          TTTGTGCAAGCACAGTTGATGGCACGGCACCTGCGTGGCGATCTGGATGGCTACCCCTCC
FDC1083         TTTGTGCAAGCACAGTTGATGGCACGGCACCTGCGTGGCGATCTGGATGGCTACCCCTCC
JF90-2          TTTGTGCAAGCACAGTTGATGGCACGGCACCTGCGTGGCGATCTGGATGGCTACCCCTCC
JJ10-1          TTTGTGCAAGCACAGTTGATGGCACGGCACCTGCGTGGCGATCTGGATGGCTACCCCTCC
JJ238-10        TTTGTGCAAGCACAGTTGATGGCACGGCACCTGCGTGGCGATCTGGATGGCTACCCCTCC
JJ238-24        TTTGTGCAAGCACAGTTGATGGCACGGCACCTGCGTGGCGATCTGGATGGCTACCCCTCC
JK2-10          TTTGTGCAAGCACAGTTGATGGCACGGCACCTGCGTGGCGATCTGGATGGCTACCCCTCC
JK4-1           TTTGTGCAAGCACAGTTGATGGCACGGCACCTGCGTGGCGATCTGGATGGCTACCCCTCC
JK48            TTTGTGCAAGCACAGTTGATGGCACGGCACCTGCGTGGCGATCTGGATGGCTACCCCTCC
JK143-9         TTTGTGCAAGCACAGTTGATGGCACGGCACCTGCGTGGCGATCTGGATGGCTACCCCTCC
JK143-11        TTTGTGCAAGCACAGTTGATGGCACGGCACCTGCGTGGCGATCTGGATGGCTACCCCTCC
JM35            TTTGTGCAAGCACAGTTGATGGCACGGCACCTGCGTGGCGATCTGGATGGCTACCCCTCC
JS581           TTTGTGCAAGCACAGTTGATGGCACGGCACCTGCGTGGCGATCTGGATGGCTACCCCTCC
JS582           TTTGTGCAAGCACAGTTGATGGCACGGCACCTGCGTGGCGATCTGGATGGCTACCCCTCC
JS584           TTTGTGCAAGCACAGTTGATGGCACGGCACCTGCGTGGCGATCTGGATGGCTACCCCTCC
JW160-1         TTTGTGCAAGCACAGTTGATGGCACGGCACCTGCGTGGCGATCTGGATGGCTACCCCTCC
LB100-1         TTTGTGCAAGCACAGTTGATGGCACGGCACCTGCGTGGCGATCTGGATGGCTACCCCTCC
LC80            TTTGTGCAAGCACAGTTGATGGCACGGCACCTGCGTGGCGATCTGGATGGCTACCCCTCC
LD7-1           TTTGTGCAAGCACAGTTGATGGCACGGCACCTGCGTGGCGATCTGGATGGCTACCCCTCC
LD71a           TTTGTGCAAGCACAGTTGATGGCACGGCACCTGCGTGGCGATCTGGATGGCTACCCCTCC
LE3-1           TTTGTGCAAGCACAGTTGATGGCACGGCACCTGCGTGGCGATCTGGATGGCTACCCCTCC
LE20-1          TTTGTGCAAGCACAGTTGATGGCACGGCACCTGCGTGGCGATCTGGATGGCTACCCCTCC
LE116-1         TTTGTGCAAGCACAGTTGATGGCACGGCACCTGCGTGGCGATCTGGATGGCTACCCCTCC
LG97            TTTGTGCAAGCACAGTTGATGGCACGGCACCTGCGTGGCGATCTGGATGGCTACCCCTCC
LG102           TTTGTGCAAGCACAGTTGATGGCACGGCACCTGCGTGGCGATCTGGATGGCTACCCCTCC
LG117           TTTGTGCAAGCACAGTTGATGGCACGGCACCTGCGTGGCGATCTGGATGGCTACCCCTCC
LH37-1          TTTGTGCAAGCACAGTTGATGGCACGGCACCTGCGTGGCGATCTGGATGGCTACCCCTCC
LMG9322         TTTGTGCAAGCACAGTTGATGGCACGGCACCTGCGTGGCGATCTGGATGGCTACCCCTCC
NCPPB3562       TTTGTGCAAGCACAGTTGATGGCACGGCACCTGCGTGGCGATCTGGATGGCTACCCCTCC
NCPPB3607       TTTGTGCAAGCACAGTTGATGGCACGGCACCTGCGTGGCGATCTGGATGGCTACCCCTCC
NCPPB3610       TTTGTGCAAGCACAGTTGATGGCACGGCACCTGCGTGGCGATCTGGATGGCTACCCCTCC
NCPPB3612       TTTGTGCAAGCACAGTTGATGGCACGGCACCTGCGTGGCGATCTGGATGGCTACCCCTCC
NCPPB3615       TTTGTGCAAGCACAGTTGATGGCACGGCACCTGCGTGGCGATCTGGATGGCTACCCCTCC
LB302           TTTGTGCAAGCACAGTTGATGGCACGGCACCTGCGTGGCGATCTGGATGGCTACCCCTCC
LG115           TTTGTGCAAGCACAGTTGATGGCACGGCACCTGCGTGGCGATCTGGATGGCTACCCCTCC
NCPPB3608       TTTGTGCAAGCACAGTTGATGGCACGGCACCTGCGTGGCGATCTGGATGGCTACCCCTCC
X2003-3218      TTTGTGCAAGCACAGTTGATGGCACGGCACCTGCGTGGCGATCTGGATGGCTACCCCTCC
                ************************************************************

JF90-8          TTCTTCTGGAAATGA
C40             TTCTTCTGGAAATGA
CFBP2852        TTCTTCTGGAAATGA
CFBP2911        TTCTTCTGGAAATGA
FDC217          TTCTTCTGGAAATGA
FDC1083         TTCTTCTGGAAATGA
JF90-2          TTCTTCTGGAAATGA
JJ10-1          TTCTTCTGGAAATGA
JJ238-10        TTCTTCTGGAAATGA
JJ238-24        TTCTTCTGGAAATGA
JK2-10          TTCTTCTGGAAATGA
JK4-1           TTCTTCTGGAAATGA
JK48            TTCTTCTGGAAATGA
JK143-9         TTCTTCTGGAAATGA
JK143-11        TTCTTCTGGAAATGA
JM35            TTCTTCTGGAAATGA
JS581           TTCTTCTGGAAATGA
JS582           TTCTTCTGGAAATGA
JS584           TTCTTCTGGAAATGA
JW160-1         TTCTTCTGGAAATGA
LB100-1         TTCTTCTGGAAATGA
LC80            TTCTTCTGGAAATGA
LD7-1           TTCTTCTGGAAATGA
LD71a           TTCTTCTGGAAATGA
LE3-1           TTCTTCTGGAAATGA
LE20-1          TTCTTCTGGAAATGA
LE116-1         TTCTTCTGGAAATGA
LG97            TTCTTCTGGAAATGA
LG102           TTCTTCTGGAAATGA
LG117           TTCTTCTGGAAATGA
LH37-1          TTCTTCTGGAAATGA
LMG9322         TTCTTCTGGAAATGA
NCPPB3562       TTCTTCTGGAAATGA
NCPPB3607       TTCTTCTGGAAATGA
NCPPB3610       TTCTTCTGGAAATGA
NCPPB3612       TTCTTCTGGAAATGA
NCPPB3615       TTCTTCTGGAAATGA
LB302           TTCTTCTGGAAATGA
LG115           TTCTTCTGGAAATGA
NCPPB3608       TTCTTCTGGAAATGA
X2003-3218      TTCTTCTGGAAATGA
                ***************
```

Cas2 (acquisition dsDNA endonuclease)

```
NCPPB3615      ATGATGATTCTTGTAAGCTACGACGTCAGCACCAGCTCTCCCGGGGGCGAGAAGCGCCTG
C40            ATGATGATTCTTGTAAGCTACGACGTCAGCACCAGCTCTCCCGGGGGCGAGAAGCGCCTG
CFBP2852       ATGATGATTCTTGTAAGCTACGACGTCAGCACCAGCTCTCCCGGGGGCGAGAAGCGCCTG
CFBP2911       ATGATGATTCTTGTAAGCTACGACGTCAGCACCAGCTCTCCCGGGGGCGAGAAGCGCCTG
FDC217         ATGATGATTCTTGTAAGCTACGACGTCAGCACCAGCTCTCCCGGGGGCGAGAAGCGCCTG
FDC1083        ATGATGATTCTTGTAAGCTACGACGTCAGCACCAGCTCTCCCGGGGGCGAGAAGCGCCTG
JF90-2         ATGATGATTCTTGTAAGCTACGACGTCAGCACCAGCTCTCCCGGGGGCGAGAAGCGCCTG
JJ10-1         ATGATGATTCTTGTAAGCTACGACGTCAGCACCAGCTCTCCCGGGGGCGAGAAGCGCCTG
JJ238-10       ATGATGATTCTTGTAAGCTACGACGTCAGCACCAGCTCTCCCGGGGGCGAGAAGCGCCTG
JJ238-24       ATGATGATTCTTGTAAGCTACGACGTCAGCACCAGCTCTCCCGGGGGCGAGAAGCGCCTG
JK2-10         ATGATGATTCTTGTAAGCTACGACGTCAGCACCAGCTCTCCCGGGGGCGAGAAGCGCCTG
JK4-1          ATGATGATTCTTGTAAGCTACGACGTCAGCACCAGCTCTCCCGGGGGCGAGAAGCGCCTG
JK48           ATGATGATTCTTGTAAGCTACGACGTCAGCACCAGCTCTCCCGGGGGCGAGAAGCGCCTG
JK143-9        ATGATGATTCTTGTAAGCTACGACGTCAGCACCAGCTCTCCCGGGGGCGAGAAGCGCCTG
JK143-11       ATGATGATTCTTGTAAGCTACGACGTCAGCACCAGCTCTCCCGGGGGCGAGAAGCGCCTG
JM35           ATGATGATTCTTGTAAGCTACGACGTCAGCACCAGCTCTCCCGGGGGCGAGAAGCGCCTG
JS581          ATGATGATTCTTGTAAGCTACGACGTCAGCACCAGCTCTCCCGGGGGCGAGAAGCGCCTG
JS582          ATGATGATTCTTGTAAGCTACGACGTCAGCACCAGCTCTCCCGGGGGCGAGAAGCGCCTG
JS584          ATGATGATTCTTGTAAGCTACGACGTCAGCACCAGCTCTCCCGGGGGCGAGAAGCGCCTG
JW160-1        ATGATGATTCTTGTAAGCTACGACGTCAGCACCAGCTCTCCCGGGGGCGAGAAGCGCCTG
LB100-1        ATGATGATTCTTGTAAGCTACGACGTCAGCACCAGCTCTCCCGGGGGCGAGAAGCGCCTG
LC80           ATGATGATTCTTGTAAGCTACGACGTCAGCACCAGCTCTCCCGGGGGCGAGAAGCGCCTG
LD7-1          ATGATGATTCTTGTAAGCTACGACGTCAGCACCAGCTCTCCCGGGGGCGAGAAGCGCCTG
LD71a          ATGATGATTCTTGTAAGCTACGACGTCAGCACCAGCTCTCCCGGGGGCGAGAAGCGCCTG
LE3-1          ATGATGATTCTTGTAAGCTACGACGTCAGCACCAGCTCTCCCGGGGGCGAGAAGCGCCTG
LE20-1         ATGATGATTCTTGTAAGCTACGACGTCAGCACCAGCTCTCCCGGGGGCGAGAAGCGCCTG
LE116-1        ATGATGATTCTTGTAAGCTACGACGTCAGCACCAGCTCTCCCGGGGGCGAGAAGCGCCTG
LG117          ATGATGATTCTTGTAAGCTACGACGTCAGCACCAGCTCTCCCGGGGGCGAGAAGCGCCTG
LH37-1         ATGATGATTCTTGTAAGCTACGACGTCAGCACCAGCTCTCCCGGGGGCGAGAAGCGCCTG
LMG9322        ATGATGATTCTTGTAAGCTACGACGTCAGCACCAGCTCTCCCGGGGGCGAGAAGCGCCTG
NCPPB3562      ATGATGATTCTTGTAAGCTACGACGTCAGCACCAGCTCTCCCGGGGGCGAGAAGCGCCTG
NCPPB3607      ATGATGATTCTTGTAAGCTACGACGTCAGCACCAGCTCTCCCGGGGGCGAGAAGCGCCTG
NCPPB3610      ATGATGATTCTTGTAAGCTACGACGTCAGCACCAGCTCTCCCGGGGGCGAGAAGCGCCTG
NCPPB3612      ATGATGATTCTTGTAAGCTACGACGTCAGCACCAGCTCTCCCGGGGGCGAGAAGCGCCTG
LB302          ATGATGATTCTTGTAAGCTAGGACGTCAGCACCAGCTCTCCCGGGGGCCAGAAGCGCCTG
LG115          ATGATGATTCTTGTAAGCTAGGACGTCAGCACCAGCTCTCCCGGGGGCCAGAAGCGCCTG
NCPPB3608      ATGATGATTCTTGTAAGCTAGGACGTCAGCACCAGCTCTCCCGGGGGCCAGAAGCGCCTG
X2003-3218     ATGATGATTCTTGTAAGCTAGGACGTCAGCACCAGCTCTCCCGGGGGCCAGAAGCGCCTG
LG97           ATGATGATTCTTGTAAGCTAGGACGTCAGCACCAGCTCTCCCGGGGGCGAGAAGCGCCTG
LG102          ATGATGATTCTTGTAAGCTAGGACGTCAGCACCAGCTCTCCCGGGGGCGAGAAGCGCCTG
JF90-8         ATGATGATTCTTGTAAGCTAGGACGTCAGCACCAGCTCTCCCGGGGGCGAGAAGCGCCTG
               ******************** *************************** ***********

NCPPB3615      CGCAAGGTCGCCAAAGCCTGCCGGGACCGTGGCCAGCGCGTGCAGTTCTCGGTCTTCGAG
C40            CGCAAGGTCGCCAAAGCCTGCCGGGACCGTGGCCAGCGCGTGCAGTTCTCGGTCTTCGAG
CFBP2852       CGCAAGGTCGCCAAAGCCTGCCGGGACCGTGGCCAGCGCGTGCAGTTCTCGGTCTTCGAG
CFBP2911       CGCAAGGTCGCCAAAGCCTGCCGGGACCGTGGCCAGCGCGTGCAGTTCTCGGTCTTCGAG
FDC217         CGCAAGGTCGCCAAAGCCTGCCGGGACCGTGGCCAGCGCGTGCAGTTCTCGGTCTTCGAG
FDC1083        CGCAAGGTCGCCAAAGCCTGCCGGGACCGTGGCCAGCGCGTGCAGTTCTCGGTCTTCGAG
JF90-2         CGCAAGGTCGCCAAAGCCTGCCGGGACCGTGGCCAGCGCGTGCAGTTCTCGGTCTTCGAG
JJ10-1         CGCAAGGTCGCCAAAGCCTGCCGGGACCGTGGCCAGCGCGTGCAGTTCTCGGTCTTCGAG
JJ238-10       CGCAAGGTCGCCAAAGCCTGCCGGGACCGTGGCCAGCGCGTGCAGTTCTCGGTCTTCGAG
JJ238-24       CGCAAGGTCGCCAAAGCCTGCCGGGACCGTGGCCAGCGCGTGCAGTTCTCGGTCTTCGAG
JK2-10         CGCAAGGTCGCCAAAGCCTGCCGGGACCGTGGCCAGCGCGTGCAGTTCTCGGTCTTCGAG
JK4-1          CGCAAGGTCGCCAAAGCCTGCCGGGACCGTGGCCAGCGCGTGCAGTTCTCGGTCTTCGAG
JK48           CGCAAGGTCGCCAAAGCCTGCCGGGACCGTGGCCAGCGCGTGCAGTTCTCGGTCTTCGAG
JK143-9        CGCAAGGTCGCCAAAGCCTGCCGGGACCGTGGCCAGCGCGTGCAGTTCTCGGTCTTCGAG
JK143-11       CGCAAGGTCGCCAAAGCCTGCCGGGACCGTGGCCAGCGCGTGCAGTTCTCGGTCTTCGAG
JM35           CGCAAGGTCGCCAAAGCCTGCCGGGACCGTGGCCAGCGCGTGCAGTTCTCGGTCTTCGAG
JS581          CGCAAGGTCGCCAAAGCCTGCCGGGACCGTGGCCAGCGCGTGCAGTTCTCGGTCTTCGAG
JS582          CGCAAGGTCGCCAAAGCCTGCCGGGACCGTGGCCAGCGCGTGCAGTTCTCGGTCTTCGAG
JS584          CGCAAGGTCGCCAAAGCCTGCCGGGACCGTGGCCAGCGCGTGCAGTTCTCGGTCTTCGAG
JW160-1        CGCAAGGTCGCCAAAGCCTGCCGGGACCGTGGCCAGCGCGTGCAGTTCTCGGTCTTCGAG
LB100-1        CGCAAGGTCGCCAAAGCCTGCCGGGACCGTGGCCAGCGCGTGCAGTTCTCGGTCTTCGAG
LC80           CGCAAGGTCGCCAAAGCCTGCCGGGACCGTGGCCAGCGCGTGCAGTTCTCGGTCTTCGAG
LD7-1          CGCAAGGTCGCCAAAGCCTGCCGGGACCGTGGCCAGCGCGTGCAGTTCTCGGTCTTCGAG
LD71a          CGCAAGGTCGCCAAAGCCTGCCGGGACCGTGGCCAGCGCGTGCAGTTCTCGGTCTTCGAG
LE3-1          CGCAAGGTCGCCAAAGCCTGCCGGGACCGTGGCCAGCGCGTGCAGTTCTCGGTCTTCGAG
LE20-1         CGCAAGGTCGCCAAAGCCTGCCGGGACCGTGGCCAGCGCGTGCAGTTCTCGGTCTTCGAG
LE116-1        CGCAAGGTCGCCAAAGCCTGCCGGGACCGTGGCCAGCGCGTGCAGTTCTCGGTCTTCGAG
LG117          CGCAAGGTCGCCAAAGCCTGCCGGGACCGTGGCCAGCGCGTGCAGTTCTCGGTCTTCGAG
LH37-1         CGCAAGGTCGCCAAAGCCTGCCGGGACCGTGGCCAGCGCGTGCAGTTCTCGGTCTTCGAG
LMG9322        CGCAAGGTCGCCAAAGCCTGCCGGGACCGTGGCCAGCGCGTGCAGTTCTCGGTCTTCGAG
NCPPB3562      CGCAAGGTCGCCAAAGCCTGCCGGGACCGTGGCCAGCGCGTGCAGTTCTCGGTCTTCGAG
NCPPB3607      CGCAAGGTCGCCAAAGCCTGCCGGGACCGTGGCCAGCGCGTGCAGTTCTCGGTCTTCGAG
NCPPB3610      CGCAAGGTCGCCAAAGCCTGCCGGGACCGTGGCCAGCGCGTGCAGTTCTCGGTCTTCGAG
NCPPB3612      CGCAAGGTCGCCAAAGCCTGCCGGGACCGTGGCCAGCGCGTGCAGTTCTCGGTCTTCGAG
LB302          CGCAAGGTCGCCAAAGCCTGCCGGGACCGTGGCCAGCGCGTGCAGTTCTCGGTCTTCGAG
LG115          CGCAAGGTCGCCAAAGCCTGCCGGGACCGTGGCCAGCGCGTGCAGTTCTCGGTCTTCGAG
NCPPB3608      CGCAAGGTCGCCAAAGCCTGCCGGGACCGTGGCCAGCGCGTGCAGTTCTCGGTCTTCGAG
X2003-3218     CGCAAGGTCGCCAAAGCCTGCCGGGACCGTGGCCAGCGCGTGCAGTTCTCGGTCTTCGAG
LG97           CGCAAGGTCGCCAAAGCCTGCCGGGACCGTGGCCAGCGCGTGCAGTTCTCGGTCTTCGAG
LG102          CGCAAGGTCGCCAAAGCCTGCCGGGACCGTGGCCAGCGCGTGCAGTTCTCGGTCTTCGAG
JF90-8         CGCAAGGTCGCCAAAGCCTGCCGGGACCGTGGCCAGCGCGTGCAGTTCTCGGTCTTCGAG
               ************************************************************
```

```
NCPPB3615      ATCGAAGTCGAGCCTGCCCAATGGACTGAATTACGGCAGCAGCTATGCGACCTGATCGAC
C40            ATCGAAGTCGAGCCTGCCCAATGGACTGAATTACGGCAGCAGCTATGCGACCTGATCGAC
CFBP2852       ATCGAAGTCGAGCCTGCCCAATGGACTGAATTACGGCAGCAGCTATGCGACCTGATCGAC
CFBP2911       ATCGAAGTCGAGCCTGCCCAATGGACTGAATTACGGCAGCAGCTATGCGACCTGATCGAC
FDC217         ATCGAAGTCGAGCCTGCCCAATGGACTGAATTACGGCAGCAGCTATGCGACCTGATCGAC
FDC1083        ATCGAAGTCGAGCCTGCCCAATGGACTGAATTACGGCAGCAGCTATGCGACCTGATCGAC
JF90-2         ATCGAAGTCGAGCCTGCCCAATGGACTGAATTACGGCAGCAGCTATGCGACCTGATCGAC
JJ10-1         ATCGAAGTCGAGCCTGCCCAATGGACTGAATTACGGCAGCAGCTATGCGACCTGATCGAC
JJ238-10       ATCGAAGTCGAGCCTGCCCAATGGACTGAATTACGGCAGCAGCTATGCGACCTGATCGAC
JJ238-24       ATCGAAGTCGAGCCTGCCCAATGGACTGAATTACGGCAGCAGCTATGCGACCTGATCGAC
JK2-10         ATCGAAGTCGAGCCTGCCCAATGGACTGAATTACGGCAGCAGCTATGCGACCTGATCGAC
JK4-1          ATCGAAGTCGAGCCTGCCCAATGGACTGAATTACGGCAGCAGCTATGCGACCTGATCGAC
JK48           ATCGAAGTCGAGCCTGCCCAATGGACTGAATTACGGCAGCAGCTATGCGACCTGATCGAC
JK143-9        ATCGAAGTCGAGCCTGCCCAATGGACTGAATTACGGCAGCAGCTATGCGACCTGATCGAC
JK143-11       ATCGAAGTCGAGCCTGCCCAATGGACTGAATTACGGCAGCAGCTATGCGACCTGATCGAC
JM35           ATCGAAGTCGAGCCTGCCCAATGGACTGAATTACGGCAGCAGCTATGCGACCTGATCGAC
JS581          ATCGAAGTCGAGCCTGCCCAATGGACTGAATTACGGCAGCAGCTATGCGACCTGATCGAC
JS582          ATCGAAGTCGAGCCTGCCCAATGGACTGAATTACGGCAGCAGCTATGCGACCTGATCGAC
JS584          ATCGAAGTCGAGCCTGCCCAATGGACTGAATTACGGCAGCAGCTATGCGACCTGATCGAC
JW160-1        ATCGAAGTCGAGCCTGCCCAATGGACTGAATTACGGCAGCAGCTATGCGACCTGATCGAC
LB100-1        ATCGAAGTCGAGCCTGCCCAATGGACTGAATTACGGCAGCAGCTATGCGACCTGATCGAC
LC80           ATCGAAGTCGAGCCTGCCCAATGGACTGAATTACGGCAGCAGCTATGCGACCTGATCGAC
LD7-1          ATCGAAGTCGAGCCTGCCCAATGGACTGAATTACGGCAGCAGCTATGCGACCTGATCGAC
LD71a          ATCGAAGTCGAGCCTGCCCAATGGACTGAATTACGGCAGCAGCTATGCGACCTGATCGAC
LE3-1          ATCGAAGTCGAGCCTGCCCAATGGACTGAATTACGGCAGCAGCTATGCGACCTGATCGAC
LE20-1         ATCGAAGTCGAGCCTGCCCAATGGACTGAATTACGGCAGCAGCTATGCGACCTGATCGAC
LE116-1        ATCGAAGTCGAGCCTGCCCAATGGACTGAATTACGGCAGCAGCTATGCGACCTGATCGAC
LG117          ATCGAAGTCGAGCCTGCCCAATGGACTGAATTACGGCAGCAGCTATGCGACCTGATCGAC
LH37-1         ATCGAAGTCGAGCCTGCCCAATGGACTGAATTACGGCAGCAGCTATGCGACCTGATCGAC
LMG9322        ATCGAAGTCGAGCCTGCCCAATGGACTGAATTACGGCAGCAGCTATGCGACCTGATCGAC
NCPPB3562      ATCGAAGTCGAGCCTGCCCAATGGACTGAATTACGGCAGCAGCTATGCGACCTGATCGAC
NCPPB3607      ATCGAAGTCGAGCCTGCCCAATGGACTGAATTACGGCAGCAGCTATGCGACCTGATCGAC
NCPPB3610      ATCGAAGTCGAGCCTGCCCAATGGACTGAATTACGGCAGCAGCTATGCGACCTGATCGAC
NCPPB3612      ATCGAAGTCGAGCCTGCCCAATGGACTGAATTACGGCAGCAGCTATGCGACCTGATCGAC
LB302          ATCGAAGTCGAGCCTGCCCAATGGACTGAATTACGGCAGCAGCTATGCGACCTGATCGAC
LG115          ATCGAAGTCGAGCCTGCCCAATGGACTGAATTACGGCAGCAGCTATGCGACCTGATCGAC
NCPPB3608      ATCGAAGTCGAGCCTGCCCAATGGACTGAATTACGGCAGCAGCTATGCGACCTGATCGAC
X2003-3218     ATCGAAGTCGAGCCTGCCCAATGGACTGAATTACGGCAGCAGCTATGCGACCTGATCGAC
LG97           ATCGAAGTCGAGCCTGCTCAATGGACTGAATTACGGCAGCAGCTATGCGACCTGATCGAC
LG102          ATCGAAGTCGAGCCTGCTCAATGGACTGAATTACGGCAGCAGCTATGCGACCTGATCGAC
JF90-8         ATCGAAGTCGAGCCTGCCCAATGGACTGAATTACGGCAGCAGCTATGCGACCTGATCGAC
               ***************** ******************************************

NCPPB3615      CCGGCCCTGGACAGCCTACGGTTCTATCACCTTGGCGCGAAATGGTAGACCCGCGTGGAG
C40            CCGGCCCTGGACAGCCTACGGTTCTATCACCTTGGCGCGAAATGGGAGACCCGCGTGGAG
CFBP2852       CCGGCCCTGGACAGCCTACGGTTCTATCACCTTGGCGCGAAATGGGAGACCCGCGTGGAG
CFBP2911       CCGGCCCTGGACAGCCTACGGTTCTATCACCTTGGCGCGAAATGGGAGACCCGCGTGGAG
FDC217         CCGGCCCTGGACAGCCTACGGTTCTATCACCTTGGCGCGAAATGGGAGACCCGCGTGGAG
FDC1083        CCGGCCCTGGACAGCCTACGGTTCTATCACCTTGGCGCGAAATGGGAGACCCGCGTGGAG
JF90-2         CCGGCCCTGGACAGCCTACGGTTCTATCACCTTGGCGCGAAATGGGAGACCCGCGTGGAG
JJ10-1         CCGGCCCTGGACAGCCTACGGTTCTATCACCTTGGCGCGAAATGGGAGACCCGCGTGGAG
JJ238-10       CCGGCCCTGGACAGCCTACGGTTCTATCACCTTGGCGCGAAATGGGAGACCCGCGTGGAG
JJ238-24       CCGGCCCTGGACAGCCTACGGTTCTATCACCTTGGCGCGAAATGGGAGACCCGCGTGGAG
JK2-10         CCGGCCCTGGACAGCCTACGGTTCTATCACCTTGGCGCGAAATGGGAGACCCGCGTGGAG
JK4-1          CCGGCCCTGGACAGCCTACGGTTCTATCACCTTGGCGCGAAATGGGAGACCCGCGTGGAG
JK48           CCGGCCCTGGACAGCCTACGGTTCTATCACCTTGGCGCGAAATGGGAGACCCGCGTGGAG
JK143-9        CCGGCCCTGGACAGCCTACGGTTCTATCACCTTGGCGCGAAATGGGAGACCCGCGTGGAG
JK143-11       CCGGCCCTGGACAGCCTACGGTTCTATCACCTTGGCGCGAAATGGGAGACCCGCGTGGAG
JM35           CCGGCCCTGGACAGCCTACGGTTCTATCACCTTGGCGCGAAATGGGAGACCCGCGTGGAG
JS581          CCGGCCCTGGACAGCCTACGGTTCTATCACCTTGGCGCGAAATGGGAGACCCGCGTGGAG
JS582          CCGGCCCTGGACAGCCTACGGTTCTATCACCTTGGCGCGAAATGGGAGACCCGCGTGGAG
JS584          CCGGCCCTGGACAGCCTACGGTTCTATCACCTTGGCGCGAAATGGGAGACCCGCGTGGAG
JW160-1        CCGGCCCTGGACAGCCTACGGTTCTATCACCTTGGCGCGAAATGGGAGACCCGCGTGGAG
LB100-1        CCGGCCCTGGACAGCCTACGGTTCTATCACCTTGGCGCGAAATGGGAGACCCGCGTGGAG
LC80           CCGGCCCTGGACAGCCTACGGTTCTATCACCTTGGCGCGAAATGGGAGACCCGCGTGGAG
LD7-1          CCGGCCCTGGACAGCCTACGGTTCTATCACCTTGGCGCGAAATGGGAGACCCGCGTGGAG
LD71a          CCGGCCCTGGACAGCCTACGGTTCTATCACCTTGGCGCGAAATGGGAGACCCGCGTGGAG
LE3-1          CCGGCCCTGGACAGCCTACGGTTCTATCACCTTGGCGCGAAATGGGAGACCCGCGTGGAG
LE20-1         CCGGCCCTGGACAGCCTACGGTTCTATCACCTTGGCGCGAAATGGGAGACCCGCGTGGAG
LE116-1        CCGGCCCTGGACAGCCTACGGTTCTATCACCTTGGCGCGAAATGGGAGACCCGCGTGGAG
LG117          CCGGCCCTGGACAGCCTACGGTTCTATCACCTTGGCGCGAAATGGGAGACCCGCGTGGAG
LH37-1         CCGGCCCTGGACAGCCTACGGTTCTATCACCTTGGCGCGAAATGGGAGACCCGCGTGGAG
LMG9322        CCGGCCCTGGACAGCCTACGGTTCTATCACCTTGGCGCGAAATGGGAGACCCGCGTGGAG
NCPPB3562      CCGGCCCTGGACAGCCTACGGTTCTATCACCTTGGCGCGAAATGGGAGACCCGCGTGGAG
NCPPB3607      CCGGCCCTGGACAGCCTACGGTTCTATCACCTTGGCGCGAAATGGGAGACCCGCGTGGAG
NCPPB3610      CCGGCCCTGGACAGCCTACGGTTCTATCACCTTGGCGCGAAATGGGAGACCCGCGTGGAG
NCPPB3612      CCGGCCCTGGACAGCCTACGGTTCTATCACCTTGGCGCGAAATGGGAGACCCGCGTGGAG
LB302          CCGGCCCTGGACAGCCTACGGTTCTATCACCTTGGCGCGAAATGGGAGACCCGCGTGGAG
LG115          CCGGCCCTGGACAGCCTACGGTTCTATCACCTTGGCGCGAAATGGGAGACCCGCGTGGAG
NCPPB3608      CCGGCCCTGGACAGCCTACGGTTCTATCACCTTGGCGCGAAATGGGAGACCCGCGTGGAG
X2003-3218     CCGGCCCTGGACAGCCTACGGTTCTATCACCTTGGCGCGAAATGGGAGACCCGCGTGGAG
LG97           CCGGCCCTGGACAGCCTACGGTTCTATCACCTTGGCGCGAAATGGGAGACCCGCGTGGAG
LG102          CCGGCCCTGGACAGCCTACGGTTCTATCACCTTGGCGCGAAATGGGAGACCCGCGTGGAG
JF90-8         CCGGCCCTGGACAGCCTACGGTTCTATCACCTTGGCGCGAAATGGGAGACCCGCGTGGAG
               ********************************************* **************
```

```
NCPPB3615        CACATCGGCGCCAAGCCCAGCCTGAACCTCAAAGGCCCACTGATTTTTTGA
C40              CACATCGGCGCCAAGCCCAGCCTGAACCTCAAAGGCCCACTGATTTTTTGA
CFBP2852         CACATCGGCGCCAAGCCCAGCCTGAACCTCAAAGGCCCACTGATTTTTTGA
CFBP2911         CACATCGGCGCCAAGCCCAGCCTGAACCTCAAAGGCCCACTGATTTTTTGA
FDC217           CACATCGGCGCCAAGCCCAGCCTGAACCTCAAAGGCCCACTGATTTTTTGA
FDC1083          CACATCGGCGCCAAGCCCAGCCTGAACCTCAAAGGCCCACTGATTTTTTGA
JF90-2           CACATCGGCGCCAAGCCCAGCCTGAACCTCAAAGGCCCACTGATTTTTTGA
JJ10-1           CACATCGGCGCCAAGCCCAGCCTGAACCTCAAAGGCCCACTGATTTTTTGA
JJ238-10         CACATCGGCGCCAAGCCCAGCCTGAACCTCAAAGGCCCACTGATTTTTTGA
JJ238-24         CACATCGGCGCCAAGCCCAGCCTGAACCTCAAAGGCCCACTGATTTTTTGA
JK2-10           CACATCGGCGCCAAGCCCAGCCTGAACCTCAAAGGCCCACTGATTTTTTGA
JK4-1            CACATCGGCGCCAAGCCCAGCCTGAACCTCAAAGGCCCACTGATTTTTTGA
JK48             CACATCGGCGCCAAGCCCAGCCTGAACCTCAAAGGCCCACTGATTTTTTGA
JK143-9          CACATCGGCGCCAAGCCCAGCCTGAACCTCAAAGGCCCACTGATTTTTTGA
JK143-11         CACATCGGCGCCAAGCCCAGCCTGAACCTCAAAGGCCCACTGATTTTTTGA
JM35             CACATCGGCGCCAAGCCCAGCCTGAACCTCAAAGGCCCACTGATTTTTTGA
JS581            CACATCGGCGCCAAGCCCAGCCTGAACCTCAAAGGCCCACTGATTTTTTGA
JS582            CACATCGGCGCCAAGCCCAGCCTGAACCTCAAAGGCCCACTGATTTTTTGA
JS584            CACATCGGCGCCAAGCCCAGCCTGAACCTCAAAGGCCCACTGATTTTTTGA
JW160-1          CACATCGGCGCCAAGCCCAGCCTGAACCTCAAAGGCCCACTGATTTTTTGA
LB100-1          CACATCGGCGCCAAGCCCAGCCTGAACCTCAAAGGCCCACTGATTTTTTGA
LC80             CACATCGGCGCCAAGCCCAGCCTGAACCTCAAAGGCCCACTGATTTTTTGA
LD7-1            CACATCGGCGCCAAGCCCAGCCTGAACCTCAAAGGCCCACTGATTTTTTGA
LD71a            CACATCGGCGCCAAGCCCAGCCTGAACCTCAAAGGCCCACTGATTTTTTGA
LE3-1            CACATCGGCGCCAAGCCCAGCCTGAACCTCAAAGGCCCACTGATTTTTTGA
LE20-1           CACATCGGCGCCAAGCCCAGCCTGAACCTCAAAGGCCCACTGATTTTTTGA
LE116-1          CACATCGGCGCCAAGCCCAGCCTGAACCTCAAAGGCCCACTGATTTTTTGA
LG117            CACATCGGCGCCAAGCCCAGCCTGAACCTCAAAGGCCCACTGATTTTTTGA
LH37-1           CACATCGGCGCCAAGCCCAGCCTGAACCTCAAAGGCCCACTGATTTTTTGA
LMG9322          CACATCGGCGCCAAGCCCAGCCTGAACCTCAAAGGCCCACTGATTTTTTGA
NCPPB3562        CACATCGGCGCCAAGCCCAGCCTGAACCTCAAAGGCCCACTGATTTTTTGA
NCPPB3607        CACATCGGCGCCAAGCCCAGCCTGAACCTCAAAGGCCCACTGATTTTTTGA
NCPPB3610        CACATCGGCGCCAAGCCCAGCCTGAACCTCAAAGGCCCACTGATTTTTTGA
NCPPB3612        CACATCGGCGCCAAGCCCAGCCTGAACCTCAAAGGCCCACTGATTTTTTGA
LB302            CACATCGGCGCCAAGCCCAGCCTGAACCTCAAAGGCCCACTGATTTTTTGA
LG115            CACATCGGCGCCAAGCCCAGCCTGAACCTCAAAGGCCCACTGATTTTTTGA
NCPPB3608        CACATCGGCGCCAAGCCCAGCCTGAACCTCAAAGGCCCACTGATTTTTTGA
X2003-3218       CACATCGGCGCCAAGCCCAGCCTGAACCTCAAAGGCCCACTGATTTTTTGA
LG97             CACATCGGCGCCAAGCCCAGCCTGAACCTCAAAGGCCCACTGATTTTTTGA
LG102            CACATCGGCGCCAAGCCCAGCCTGAACCTCAAAGGCCCACTGATTTTTTGA
JF90-8           CACATCGGCGCCAAGCCCAGCCTGAACCTCAAAGGCCCACTGATTTTTTGA
                 ***************************************************
```
