# Supplementary material for: CRISPR elements provide a new framework for the genealogy of the citrus canker pathogen Xanthomonas citri pv. citri
Source: BMC Genomics. 2019 Dec 2;20:917. doi: 10.1186/s12864-019-6267-z (PMC6889575; doi:10.1186/s12864-019-6267-z)
Supplement: Supplementary file 9 — Additional file 9: Table S2. List of full-length genome resources used in this study. [file 12864_2019_6267_MOESM9_ESM.docx]

**Table S2.** List of full-length genome resources used in this study.

| No. | Strain | Accession number | Repeat array | Spoligo­type | Patho­type | Reference |
| --- | --- | --- | --- | --- | --- | --- |
| 1 | 03-1638-1-1 | CP023285 | 4634666-4635885 | 14 | A | Gochez et al., 2018 |
| 2a | 306 | AE008923 | 4522475-4523694 | 14 | A | da Silva et al., 2002 |
| 2b | A306 | CP006857 | 4522466-4523685 | 14 | A | Jalan & Wang, unpublished |
| 3 | 5208 (Miami) | CP009028 | 4522473-4523692 | 14 | A | Zhang et al., 2015 |
| 4 | AW13 | CP009031 | 5154998-5158499 ^#^ | 2* | A^w^ | Zhang et al., 2015 |
| 5 | AW14 | CP009034 | 5154998-5158499 ^#^ | 2* | A^w^ | Zhang et al., 2015 |
| 6 | AW15 | CP009037 | 5154991-5158492 ^#^ | 2* | A^w^ | Zhang et al., 2015 |
| 7 | AW16 | CP009040 | 5154921-5158422 ^#^ | 2* | A^w^ | Zhang et al., 2015 |
| 8 | Aw12879 | CP003778 | 5155350-5158851 ^#^ | 2* | A^w^ | Jalan et al., 2013 |
| 9 | BL18 | CP009025 | 4522471-4523690 | 14 | A | Zhang et al., 2015 |
| 10 | FB19 | CP009022 | 4522472-4523626 |  | A | Zhang et al., 2015 |
| 11 | gd2 | CP009019 | 4499512-4500600 |  | A | Zhang et al., 2015 |
| 12 | gd3 | CP009016 | 4499514-4500602 |  | A | Zhang et al., 2015 |
| 13 | jx4 | CP009013 | 4499062-4500277 | 14a | A | Zhang et al., 2015 |
| 14 | jx5 | CP009010 | 4499074-4500293 | 14 | A | Zhang et al., 2015 |
| 15 | jx-6 | CP011827 | 4499059-4500214 |  |  | Chen et al., unpublished |
| 16 | LH201 | CP018858 | 4272755-4274041 ^§^ |  | A | Richard et al., 2017 |
| 17 | LH276 | CP018854 | 4848363-4849649 |  | A | Richard et al., 2017 |
| 18 | LJ207-7 | CP018850 | 2358083-2359366 |  | A | Richard et al., 2017 |
| 19 | LL074-4 | CP018847 | 976634-977920 ^§^ |  | A | Richard et al., 2017 |
| 20 | mf20 | CP009007 | 4522472-4523691 | 14 | A | Zhang et al., 2015 |
| 21 | MN10 | CP009004 | 4499101-4500320 | 14 | A | Zhang et al., 2015 |
| 22 | MN11 | CP009001 | 4497770-4498989 | 14 | A | Zhang et al., 2015 |
| 23 | MN12 | CP008998 | 4499090-4500309 | 14 | A | Zhang et al., 2015 |
| 24 | NT17 | CP008995 | 4522479-4523698 | 14 | A | Zhang et al., 2015 |
| 25 | TX160042 | CP020882 | 702499-706067 ^§#^ | 1* | A^w^ | Munoz Bodnar et al., 2017 |
| 26 | TX160149 | CP020885 | 3901599-3903015 | 4 | A^w^ | Munoz Bodnar et al., 2017 |
| 27 | TX160197 | CP020889 | 2019262-2022830 ^#^ | 1* | A^w^ | Munoz Bodnar et al., 2017 |
| 28 | UI6 | CP008992 | 4498159-4499378 | 14b | A | Zhang et al., 2015 |
| 29 | UI7 | CP008989 | 4498149-4499234 |  | A | Zhang et al., 2015 |
| 30a | Xac29-1 | CP004399 | 4530049-4530804 |  |  | Chen et al., unpublished |
| 30b | Xcc29-1 | CP023661 | 4509073-4509828 |  |  |  |
| 31 | Xcc49 | CP023662 | 4499171-4500390 | 14 |  |  |
| 32 | ICPB 11122 | ACPY01000000 | No CRISPR array |  | XauB | Moreira et al., 2010 |
| 33 | ICPB 10535 | ACPX01000000 | No CRISPR array |  | XauC | Moreira et al., 2010 |

^§^ CRISPR array in opposite direction

^#^ CRISPR array interrupted by an IS element

**References**

1. da Silva AC, Ferro JA, Reinach FC, Farah CS, Furlan LR, Quaggio RB, et al. Comparison of the genomes of two *Xanthomonas* pathogens with differing host specificities. Nature 2002;417(6887):459–463.
2. Gochez AM, Huguet-Tapia JC, Minsavage GV, Shantaraj D, Jalan N, Strauß A, et al. Pacbio sequencing of copper-tolerant *Xanthomonas citri* reveals presence of a chimeric plasmid structure and provides insights into reassortment and shuffling of transcription activator-like effectors among *X. citri* strains. BMC Genomics 2018;19(1):16.
3. Jalan N, Kumar D, Yu F, Jones JB, Graham JH, Wang N. Complete genome sequence of *Xanthomonas citri* subsp. *citri* strain Aw12879, a restricted-host-range citrus canker-causing bacterium. Genome Announc. 2013;1(3):e00235-13.
4. Moreira LM, Almeida NF Jr, Potnis N, Digiampietri LA, Adi SS, Bortolossi JC, et al. Novel insights into the genomic basis of citrus canker based on the genome sequences of two strains of *Xanthomonas fuscans* subsp. *aurantifolii*. BMC Genomics 2010;11:238.
5. Munoz Bodnar A, Santillana G, Mavrodieva V, Liu Z, Nakhla M, Gabriel DW. Complete genome sequences of three *Xanthomonas citri* strains from Texas. Genome Announc. 2017;5(28):e00609-17.
6. Richard D, Boyer C, Vernière C, Canteros BI, Lefeuvre P, Pruvost O. Complete genome sequences of six copper-resistant *Xanthomonas citri* pv. *citri* strains causing Asiatic citrus canker, obtained using long-read technology. Genome Announc. 2017;5(12):e00010-17.
7. Zhang Y, Jalan N, Zhou X, Goss E, Jones JB, Setubal JC, et al. Positive selection is the main driving force for evolution of citrus canker-causing *Xanthomonas*. ISME J. 2015;9(10):2128–2138.
