# Supplementary material for: CRISPR elements provide a new framework for the genealogy of the citrus canker pathogen Xanthomonas citri pv. citri
Source: BMC Genomics. 2019 Dec 2;20:917. doi: 10.1186/s12864-019-6267-z (PMC6889575; doi:10.1186/s12864-019-6267-z)
Supplement: Supplementary file 10 — Additional file 10: Table S3. List of draft genome resources used in this study. [file 12864_2019_6267_MOESM10_ESM.docx]

**Table S3.** List of draft genome resources used in this study.

| No. | Strain | Accession number | Repeat array | Spoligo­type | Patho­type | Reference |
| --- | --- | --- | --- | --- | --- | --- |
| 1 | As8 (A*1609) | JPLO01000002 | 109219-110373 |  | A* | Zhang et al., 2015 |
| 2 | As9 (A*1974) | JPLP01000002 | 109498-110786 |  | A* | Zhang et al., 2015 |
| 3 | As270 (A*270) | JPLN01000002 | 109499-110787 |  | A* | Zhang et al., 2015 |
| 4 | C40 | CCWX01000165 | 25664-26950 | 8 | A | Gordon et al., 2015 |
| 5 | CFBP 2852 | CCWI01000132 | 76103-77453 | 2 | A | Gordon et al., 2015 |
| 6 | CFBP 2911 | CCWD01000161 | 59689-61770 | 11 | A* | Gordon et al., 2015 |
| 7 | FDC217 | CCWY01000114 | 67467-68686 | 14 | A | Gordon et al., 2015 |
| 8 | FDC628 | LAUE01000050 | 79489-80775 | 8 | A | Varani et al., unpublished |
| 9 | FDC636 | LAUQ01000051 | 48675-49894 ^§^ | 14 | A | Varani et al., unpublished |
| 10 | FDC654 | LAUF01000107 | 52750-53901 ^§^ |  | A | Varani et al., unpublished |
| 11 | FDC828 | LAUP01000042 | 52750-53442 ^§^ |  | A | Varani et al., unpublished |
| 12 | FDC1083 | CCVZ01000100 | 67467-68686 | 14 | A | Gordon et al., 2015 |
| 13 | FDC1662 | LAUN01000093 | 52750-53969 ^§^ | 14 | A | Varani et al., unpublished |
| 14 | FDC1682 | LAUG01000027 | 52748-53968 ^§^ | 10 | A* | Varani et al., unpublished |
| 15 | JF090-2 | CCWA01000008 | 69188-70408 | 10 | A* | Gordon et al., 2015 |
| 16 | JF090-8 | CCWB01000009 | 69550-70966 | 4 | A^w^ | Gordon et al., 2015 |
| 17 | JJ010-1 | CDDV01000080 CDDV01000081 | 31530-32649 1-100 | 19 | A | Gordon et al., 2015 |
| 18 | JJ238-10 | CCWC01000116 | 67547-68963 | 4 | A | Gordon et al., 2015 |
| 19 | JJ238-24 | CCVX01000144 | 25945-27095 | 16 | A* | Gordon et al., 2015 |
| 20 | JK002-10 | CCWV01000068 | 15484-16772 | 9 | A* | Gordon et al., 2015 |
| 21 | JK004-1 | CDMR01000156 | 31453-32739 | 8 | A | Gordon et al., 2015 |
| 22 | JK048 | CDAJ01000733 | 6325-7613 | 9 | A* | Gordon et al., 2015 |
| 23 | JK143-09 | CDMQ01001222 | 6318-7468 | 16 | A* | Gordon et al., 2015 |
| 24 | JK143-11 | CDMO01000057 | 65999-67083 ^§^ | 17 | A* | Gordon et al., 2015 |
| 25 | JM035-2 | CDMS01000131 | 31435-32655 | 10 | A* | Gordon et al., 2015 |
| 26 | JS581 | CDAW01000109 | 31485-32507 | 18 | A* | Gordon et al., 2015 |
| 27 | JS582 | CDAP01000132 | 5175-6197 | 18 | A* | Gordon et al., 2015 |
| 28 | JS584 | CCWF01000028 | 69524-70546 | 18 | A* | Gordon et al., 2015 |
| 29 | JW160-1 | CCWH01000145 | 69482-70964 | 2 | A | Gordon et al., 2015 |
| 30 | LB100-1 | CDAV01000117 | 32196-33678 | 2 | A | Gordon et al., 2015 |
| 31 | LB302 | CDAU01000965 CDAU01000966 | 6315-6592 ^#^ 1-1307 ^#^ | 2* | A^w^ | Gordon et al., 2015 |
| 32 | LB305 = X2003-3218 | CCWL01000150 CCWL01000151 | 69336-69637 ^#^ 1-1331 ^#^ | 2* | A^w^ | Gordon et al., 2015 |
| 33 | LC080 | CCWJ01000149 | 67540-68759 | 14 | A | Gordon et al., 2015 |
| 34 | LD007-1 | CDAL01000246 | 4333-5548 | 15 | A | Gordon et al., 2015 |
| 35 | LD71A | CCWE01000144 | 93140-94290 | 16 | A* | Gordon et al., 2015 |
| 36 | LE003-1 | CDAI01000695 | 4319-5543 | 12 | A* | Gordon et al., 2015 |
| 37 | LE020-1 | CCWK01000059 | 69478-70702 | 12 | A* | Gordon et al., 2015 |
| 38 | LE116-1 | CDHD01001308 | 4327-5542 | 15 | A | Gordon et al., 2015 |
| 39 | LG097 | CDAK01001010 CDAK01001011 | 4320-4617 57-1140 | 3* | A | Gordon et al., 2015 |
| 40 | LG098 | CDBA01000158 | 1524-2672 |  | A | Gordon et al., 2015 |
| 41 | LG102 | CDAN01001030 | 6237-7454 | 22 | A | Gordon et al., 2015 |
| 42 | LG115 | CDAY01000139 CDAY01000140 | 27084-27314 40-1257 | 2* | A^w^ | Gordon et al., 2015 |
| 43 | LG117 | CDAX01000128 | 25930-26482 | 25 | A | Gordon et al., 2015 |
| 44 | LH037-1 | CDAS01000149 | 25900-27115 | 15 | A | Gordon et al., 2015 |
| 45 | LM180 | MSQW01000001 | 2343558-2344777 | 14 | A | Richard et al., 2017 |
| 46 | LM199 | MSQV01000001 | 2724438-2725657 ^§^ | 14 | A | Richard et al., 2017 |
| 47a | LMG 9322 | CCVY01000098 | 67470-68756 | 8 | A | Gordon et al., 2015 |
| 47b | LMG 9322 | JPYD01000088 JPYD01000255 | 23021-23170 1-620 | 8 | A | Constantin et al., 2016 |
| 47c | LMG 9322 | MDJT01000185 | 79673-80959 |  |  | Patil et al., unpublished |
| 48 | NCPPB 3562 | CCXZ01000185 | 57-1272 | 15 | A | Gordon et al., 2015 |
| 49 | NCPPB 3607 | CDAT01000231 | 17522-18742 | 10 | A* | Gordon et al., 2015 |
| 50 | NCPPB 3608 | CCWG01000045 CCWG01000047 | 27069-27344 ^#^ 1-1372 ^#^ | 1* | A^w^ | Gordon et al., 2015 |
| 51 | NCPPB 3610 | CDAO01001182 | 4306-5723 | 6 | A | Gordon et al., 2015 |
| 52 | NCPPB 3612 | CDAQ01000143 | 32171-33588 | 5 | A | Gordon et al., 2015 |
| 53 | NCPPB 3615 | CDAM01000157 | 4320-5341 | 24 | A* | Gordon et al., 2015 |
| 54 | NIGEB-88 | LJGA01000004 | 85718-86610 |  | A* | Jalali et al., unpublished |
| 55 | NIGEB-386 | JRON01000038 | 25932-26824 |  |  | Jalali et al., unpublished |
| 56 | NCPPB 3213 | CDHI01000000 | No CRISPR array |  | b | Gordon et al., 2015 |

^§^ CRISPR array in opposite direction

^#^ CRISPR array interrupted by an IS element

**References**

1. Constantin EC, Cleenwerck I, Maes M, Baeyen S, Van Malderghem C, De Vos P, et al. Genetic characterization of strains named as *Xanthomonas axonopodis* pv. *dieffenbachiae* leads to a taxonomic revision of the *X. axonopodis* species complex. Plant Pathol. 2016;65(5):792–806.
2. Gordon JL, Lefeuvre P, Escalon A, Barbe V, Cruveiller S, Gagnevin L, et al. Comparative genomics of 43 strains of *Xanthomonas citri* pv. *citri* reveals the evolutionary events giving rise to pathotypes with different host ranges. BMC Genomics 2015;16:1098.
3. Richard D, Boyer C, Vernière C, Canteros BI, Lefeuvre P, Pruvost O. Complete genome sequences of six copper-resistant *Xanthomonas citri* pv. *citri* strains causing Asiatic citrus canker, obtained using long-read technology. Genome Announc. 2017;5(12):e00010-17.
4. Zhang Y, Jalan N, Zhou X, Goss E, Jones JB, Setubal JC, et al. Positive selection is the main driving force for evolution of citrus canker-causing *Xanthomonas*. ISME J. 2015;9(10):2128–2138.
