# Supplementary material for: CRISPR elements provide a new framework for the genealogy of the citrus canker pathogen Xanthomonas citri pv. citri
Source: BMC Genomics. 2019 Dec 2;20:917. doi: 10.1186/s12864-019-6267-z (PMC6889575; doi:10.1186/s12864-019-6267-z)
Supplement: Supplementary file 11 — Additional file 11: Figure S8. Primer design for PCR amplification of the CRISPR array from X. citri pv. citri. A, amplification of the full-length CRISPR arrays using primers Leader_fw and Terminator_rev. B, amplification to internal regions of the CRISPR arrays using spacer-specific primers. Forward primers are shown as red rectangles with an arrow, reverse primers are represented by blue rectangles with an arrow. [file 12864_2019_6267_MOESM11_ESM.pptx]

## Slide 1
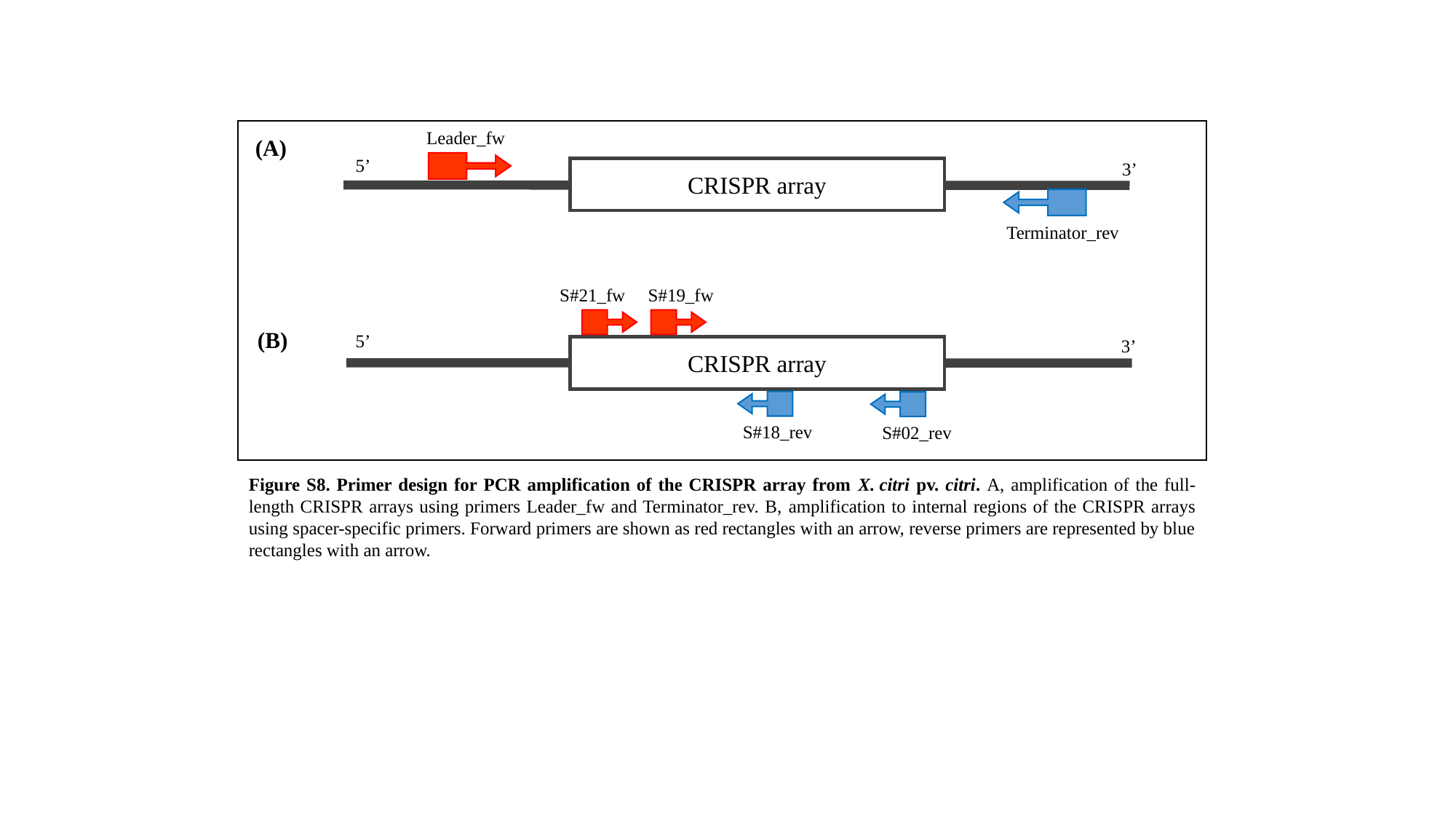

Leader_fw
5’
3’
CRISPR array
Terminator_rev
S#21_fw
S#19_fw
5’
3’
CRISPR array
S#18_rev
S#02_rev
(A)
(B)
Figure S8. Primer design for PCR amplification of the CRISPR array from X. citri pv. citri. A, amplification of the full-length CRISPR arrays using primers Leader_fw and Terminator_rev. B, amplification to internal regions of the CRISPR arrays using spacer-specific primers. Forward primers are shown as red rectangles with an arrow, reverse primers are represented by blue rectangles with an arrow.
