# Supplementary material for: CRISPR elements provide a new framework for the genealogy of the citrus canker pathogen Xanthomonas citri pv. citri
Source: BMC Genomics. 2019 Dec 2;20:917. doi: 10.1186/s12864-019-6267-z (PMC6889575; doi:10.1186/s12864-019-6267-z)
Supplement: Supplementary file 12 — Additional file 12: Figure S9. Primer design for PCR amplification of CRISPR regions adjacent to an IS element. A, primer combinations used for strains LB302, LB305, LG115 and NCPPB 3608; B, primer combinations used for strain LG097. Forward primers are shown as red rectangles with an arrow, reverse primers are represented by blue rectangles with an arrow. The yellow triangle indicates the leader sequence and the red triangle indicates the terminator sequence. Deep blue rectangles indicate CRISPR repeats. CRISPR spacers are represented by green diamonds. Spacers are numbered from 1 to 23, starting with the terminator proximal spacer, which presumably represents the oldest spacer. Orange rectangles indicate IS elements. [file 12864_2019_6267_MOESM12_ESM.pptx]

## Slide 1
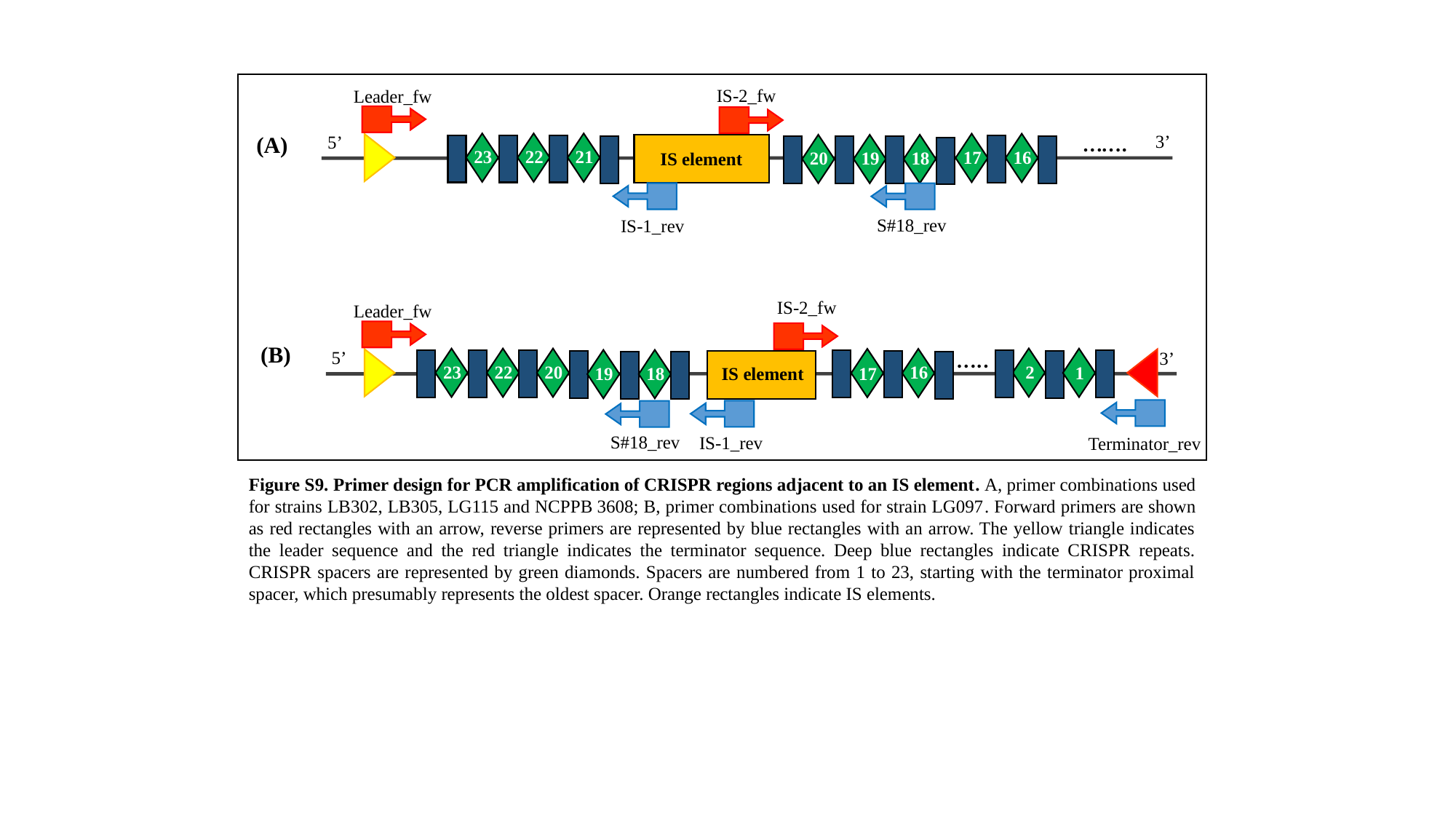

IS-2_fw
Leader_fw
…….
3’
(A)
5’
23
22
21
17
16
20
19
18
IS element
S#18_rev
IS-1_rev
IS-2_fw
Leader_fw
(B)
…..
5’
3’
2
23
22
20
16
1
19
18
17
IS element
S#18_rev
IS-1_rev
Terminator_rev
Figure S9. Primer design for PCR amplification of CRISPR regions adjacent to an IS element. A, primer combinations used for strains LB302, LB305, LG115 and NCPPB 3608; B, primer combinations used for strain LG097. Forward primers are shown as red rectangles with an arrow, reverse primers are represented by blue rectangles with an arrow. The yellow triangle indicates the leader sequence and the red triangle indicates the terminator sequence. Deep blue rectangles indicate CRISPR repeats. CRISPR spacers are represented by green diamonds. Spacers are numbered from 1 to 23, starting with the terminator proximal spacer, which presumably represents the oldest spacer. Orange rectangles indicate IS elements.
